# Health disparities across the counties of Kenya and implications for policy makers, 1990–2016: a systematic analysis for the Global Burden of Disease Study 2016

**DOI:** 10.1016/S2214-109X(18)30472-8

**Published:** 2018-10-25

**Authors:** Tom Achoki, Molly K Miller-Petrie, Scott D Glenn, Nikhila Kalra, Abaleng Lesego, Gladwell K Gathecha, Uzma Alam, Helen W Kiarie, Isabella Wanjiku Maina, Ifedayo M O Adetifa, Hellen C Barsosio, Tizta Tilahun Degfie, Peter Njenga Keiyoro, Daniel N Kiirithio, Yohannes Kinfu, Damaris K Kinyoki, James M Kisia, Varsha Sarah Krish, Abraham K Lagat, Meghan D Mooney, Wilkister Nyaora Moturi, Charles Richard James Newton, Josephine W Ngunjiri, Molly R Nixon, David O Soti, Steven Van De Vijver, Gerald Yonga, Simon I Hay, Christopher J L Murray, Mohsen Naghavi

**Affiliations:** a Sloan Management, Massachusetts Institute of Technology, Cambridge, MA, USA; b Center for Pharmaceutical Policy and Regulation, Utrecht University, Utrecht, Netherlands; c Institute for Health Metrics and Evaluation, University of Washington, Seattle, WA, USA; d Department of Health Metrics Sciences, University of Washington, Seattle, WA, USA; e Strategic Information and Learning, University of Research Company, Gaborone, Botswana; f Policy, Planning, and Healthcare Financing Department, Nairobi, Kenya; g Ministry of Health, Nairobi, Kenya; h International Center for Humanitarian Affairs, Nairobi, Kenya; i Institute of Tropical Medicine, Jomo Kenyatta University of Agriculture and Technology, Nairobi, Kenya; j Department of Infectious Disease Epidemiology, London School of Hygiene & Tropical Medicine, London, UK; k Epidemiology and Demography Department, Kilifi, Kenya; l Malaria Branch, Kilifi, Kenya; m Kenya Medical Research Institute (KEMRI)-Wellcome Trust Collaborative Programme, Kilifi, Kenya; n Department of Psychiatry, University of Oxford, Oxford, UK; o Department of Clinical Sciences, Liverpool School of Tropical Medicine, Liverpool, UK; p Population Dynamics and Reproductive Health Unit, Nairobi, Kenya; q African Population Health Research Centre, Nairobi, Kenya; r ODeL Campus, University of Nairobi, Nairobi, Kenya; s School of Medicine, University of Nairobi, Nairobi, Kenya; t Synotech Consultants, Nairobi, Kenya; u Faculty of Health, University of Canberra, Canberra, ACT, Australia; v Murdoch Children's Research Institute, Royal Children's Hospital, Melbourne, Vic, Australia; w East Africa Center, Humanitarian Leadership Academy, Nairobi, Kenya; x Department of Health Systems and Research Ethics, KEMRI-Wellcome Research Programme, Nairobi, Kenya; y Department of Environmental Science, Egerton University, Egerton, Kenya; z Department of Biological Sciences, University of Embu, Embu, Kenya; aa Eastern Africa Regional Collaborating Centre, African Centre for Disease Control and Prevention, Nairobi, Kenya

## Abstract

**Background:**

The Global Burden of Diseases, Injuries, and Risk Factors Study (GBD) 2016 provided comprehensive estimates of health loss globally. Decision makers in Kenya can use GBD subnational data to target health interventions and address county-level variation in the burden of disease.

**Methods:**

We used GBD 2016 estimates of life expectancy at birth, healthy life expectancy, all-cause and cause-specific mortality, years of life lost, years lived with disability, disability-adjusted life-years, and risk factors to analyse health by age and sex at the national and county levels in Kenya from 1990 to 2016.

**Findings:**

The national all-cause mortality rate decreased from 850·3 (95% uncertainty interval [UI] 829·8–871·1) deaths per 100 000 in 1990 to 579·0 (562·1–596·0) deaths per 100 000 in 2016. Under-5 mortality declined from 95·4 (95% UI 90·1–101·3) deaths per 1000 livebirths in 1990 to 43·4 (36·9–51·2) deaths per 1000 livebirths in 2016, and maternal mortality fell from 315·7 (242·9–399·4) deaths per 100 000 in 1990 to 257·6 (195·1–335·3) deaths per 100 000 in 2016, with steeper declines after 2006 and heterogeneously across counties. Life expectancy at birth increased by 5·4 (95% UI 3·7–7·2) years, with higher gains in females than males in all but ten counties. Unsafe water, sanitation, and handwashing, unsafe sex, and malnutrition were the leading national risk factors in 2016.

**Interpretation:**

Health outcomes have improved in Kenya since 2006. The burden of communicable diseases decreased but continues to predominate the total disease burden in 2016, whereas the non-communicable disease burden increased. Health gains varied strikingly across counties, indicating targeted approaches for health policy are necessary.

**Funding:**

Bill & Melinda Gates Foundation.

## Introduction

The Government of Kenya prioritises health as a strategic sector in its national development agenda. In the national long-term development policy, outlined in *Kenya Vision 2030*,^1^ the government commits to making strategic investments in health service provision to improve the quality of life of its population. Further commitments are elaborated in *Kenya Health Policy 2014–2030*,^2^ which aims to achieve universal health coverage by scaling up priority health services to populations in need. In 2013, Kenya devolved health services to 47 semi-autonomous counties established in response to the new constitution of 2010, increasing local autonomy for managing health services.^3^, ^4^ These and other national policies underscore the need to address existing health inequalities as a pathway towards more rapid and sustainable progress.^5^, ^6^

Kenya has made progress in tackling various health challenges, particularly those linked to priority communicable diseases.^7^, ^8^ Declines in diarrhoea, lower respiratory infections, and vaccine-preventable diseases have occurred alongside decreasing maternal and neonatal mortality, although mortality rates remain comparatively high and unequal across counties.^7^, ^9^, ^10^ The Kenya Expanded Programme on Immunization endorses childhood vaccination for tuberculosis, polio, diphtheria, whooping cough, tetanus, measles, hepatitis B, *Haemophilus influenzae* type b, pneumococcus, and rotavirus.^11^ Kenya has among the highest rates of HIV/AIDS and tuberculosis in the world, although mortality rates have declined since the early 2000s, particularly for HIV/AIDS.^12^, ^13^ Malaria mortality in children declined substantially between 2003 and 2007,^14^, ^15^ although reports indicate increasing incidence in parts of the country since 2011.^16^, ^17^ Despite these isolated reports, to date no comprehensive effort has been made to assess overall population health trends.

Evidence is emerging of a rise in non-communicable diseases (NCDs) and injuries related to rapid urbanisation and adoption of lifestyle changes characterised by high caloric intake, excessive alcohol consumption, and physical inactivity.^18^ Cancers, cardiovascular disease, and cerebrovascular diseases are increasingly the focus of public health intervention efforts in Kenya.^19^, ^20^, ^21^ However, access to preventive care—eg, blood pressure checks and hypertensive diagnoses—and treatment varies.^22^, ^23^ In 2015, more than half the population (56%) had never checked their blood pressure, whereas of those who had been diagnosed previously as hypertensive, only 22% were on treatment.^18^ That same year, approximately 27% of the Kenyan population were either overweight or obese, with the proportion being significantly higher for women (39%) compared with men (18%).^18^ Prevalence of high blood pressure, high body-mass index (BMI), and other cardiovascular risk factors also varies widely between urban and rural areas.^18^, ^24^ These emerging trends have important implications for policy making, and county-level data for NCDs can help guide decisions to prevent the growth of this disease burden.

**Research in context**

**Evidence before this study**

National estimates for Kenya have been included in previous publications of the Global Burden of Diseases, Injuries, and Risk Factors Study (GBD), including GBD 2016, which provided estimates for 333 causes of disease and disability and 84 risk factors in 195 countries and territories from 1990 to 2016. Most existing publications on health in Kenya have focused on specific diseases, locations, or populations and have not provided a comprehensive overview of population health that can be tracked and compared over time.

**Added value of this study**

Data for mortality and morbidity in Kenya are sparse, and the data that are available are often not connected together to create a comprehensive assessment of health status. GBD 2016 estimates provide a basis for universal comparison of health outcomes over time and by sex and age. Our study provides, for the first time, a subnational analysis of health in Kenya, utilising the comprehensive modelling framework of GBD to fill gaps in data and create a complete picture of health.

**Implications of all the available evidence**

Our subnational assessment presents information on health trends and burden that can be applied by policy makers at the county level in Kenya. Health outcomes have improved nationally since 2006, but heterogeneously across counties. Communicable diseases such as HIV/AIDS, diarrhoeal diseases, and lower respiratory infections require continued attention. Non-communicable diseases are a growing concern that will need to be addressed to sustain the health gains. Additional focus on gathering subnational health data in Kenya will be essential to develop a detailed understanding of the health challenges facing this country. Continued assessment of subnational health trends can guide policy making to ensure continued progress.

Understanding local variation in health trends is important because Kenya has a diverse and growing population of 45 million people made up of many ethnic groups inhabiting various counties at different levels of socioeconomic development.^25^ The population's health needs and access to health services are very different across various parts of the country. This diversity is illustrated starkly when considering counties in the arid and semi-arid north of Kenya, the highland regions, the rural northern rift, urban centres (Mombasa, Nairobi, and Kisumu), and the coastal region, Nyanza, and western Kenya.

In view of this complexity, Kenyan decision makers need a nuanced understanding of national and subnational population health trends to implement effective public health measures. The Global Burden of Diseases, Injuries, and Risk Factors Study (GBD) has paid close attention to informational needs for policy making and sought to provide the most comprehensive and up-to-date estimates for health loss and their risk factors globally. For Kenya, this analysis was done at the subnational level for the first time in the GBD 2016 study.^7^, ^26^, ^27^, ^28^, ^29^ The study we report here builds on that analysis to provide a clear basis for the objective assessment of health system performance and to facilitate benchmarking that could be instructive to health system stewards at the national and county levels. Furthermore, understanding trends at the subnational level—at which services are planned, organised, and delivered—is essential to address health inequalities.^30^ In this Article, we have used GBD 2016 estimates to analyse population health trends across the 47 counties of Kenya.

## Methods

### Overview

GBD is the most comprehensive epidemiological study to systematically gather, analyse, and produce comparable estimates of health loss and related risk factors across locations, age groups, and sex categories. The methods used in this study have been described extensively elsewhere.^7^, ^26^, ^27^, ^28^, ^29^, ^31^ Briefly, GBD identifies data sources for different aspects of the estimation process. The quality of these data is assessed systematically and corrected for known biases. Subsequently, data are subject to standardised statistical estimation and cross-validation analyses to assess model performance. All estimates produced for GBD report 95% uncertainty intervals (UIs) that account for sampling and non-sampling error associated with data and various assumptions of the modelling process and are derived from the 2·5th and 97·5th percentiles of 1000 draws. UIs incorporate sample size variability within data sources, different availability of data by age, sex, year, or location, and cause-specific model specifications, but do not currently propagate uncertainty from covariates or so-called garbage code correction.^7^, ^26^, ^27^, ^28^, ^29^ Unless otherwise specified, we report all estimates in terms of age-standardised rates, standardised using GBD world population standards.

Our Article complies with the Guidelines for Accurate and Transparent Health Estimates Reporting (GATHER).^32^ Complete information on data sources used in GBD is available from the Global Health Data Exchange. Related statistical code for Python versions 2.5.4 and 2.7.3, Stata version 13.1, or *R* version 3.1.2 is available at GitHub. Results for all locations and years are available to explore using data visualisations from the Institute for Health Metrics and Evaluation (IHME).

The GBD framework classifies causes of health loss into mutually exclusive and collectively exhaustive categories organised in a four-level hierarchy. GBD 2016 included 333 causes of disease and disability. Causes of health loss are first organised into three primary categories: communicable, maternal, neonatal, and nutritional (CMNN) disorders; NCDs; and injuries. These broad categories are divided further into increasingly more detailed categories in a consistent and comprehensive manner. Standard estimates for different causes of health loss are produced for different sexes, age groups, and locations, enabling useful comparisons. GBD uses various inter-related metrics to measure population health loss, including number of deaths and mortality rates, years of life lost due to premature death (YLLs), years of life lived with disability (YLDs), disability-adjusted life-years (DALYs), life expectancy, and healthy life expectancy (HALE).

### Kenyan data sources, geographical units, and periods

GBD 2016 synthesised many Kenyan input data sources for mortality (appendix pp 45–76), morbidity (appendix pp 77–120), and risk factors (appendix pp 121–26). These sources included population censuses, surveys, sample registration systems, and registries, in addition to published research from various sources. We used localised covariates to customise the estimation for each location for each custom cause or sex model.

We estimated all metrics nationally and individually for each county of Kenya from 1990 to 2016. We chose the years 1990, 2006, and 2016 as primary comparators for this Article, to provide both a historical perspective and a recent assessment that would be more pertinent to policy-making efforts and incorporate recent public health efforts.^33^, ^34^

To account for the reorganisation of Kenyan districts and provinces (the 2010 constitution split provinces into counties), all previous boundaries from 1990 onwards were translated onto the 47 county boundaries from 2016. We then recalculated previous data to conform to this rebuilt map.

### Mortality estimation

Briefly, we estimated all-cause under-5 and adult mortality using updated demographic methods that have been developed for GBD. These methods encompass a multistage process that includes various data sources systematically—eg, surveys, censuses, vital registration systems, birth histories, sibling histories, and household death recall. After correction for biases from different data sources, we generated estimates of under-5 and adult mortality using a combination of spatiotemporal and Gaussian process regressions using covariates (appendix pp 1–44) to help inform data sparse estimation.^28^

For cause-specific mortality, we standardised various data sources—primarily vital registration, verbal autopsy, surveys, and surveillance—and mapped these to GBD cause of death list. Data attributed to causes that could not be underlying causes of death, so-called garbage codes, were redistributed using standard algorithms. The Cause of Death Ensemble model (CODEm) uses country-level covariates and combines and tests various models to provide the most robust estimates for most causes of death.^7^ Individual cause models are then combined and corrected to be internally consistent with estimates of all-cause mortality using the cause of death correction process, CoDCorrect, with uncertainty calculated for detailed causes, aggregate levels, and all-cause mortality.^7^, ^28^

We obtained YLLs by multiplying the number of deaths from each cause in each age group by the reference life expectancy. The reference life table used in GBD estimation is derived from the highest life expectancy observed, currently 86·6 years at birth in Japan.^7^, ^28^

### Morbidity estimation

We used various data sources that capture information on non-fatal outcomes—including published studies, surveillance data, and hospital data—to generate consistent estimates for incidence, prevalence, remission (where feasible), and mortality attributable to different causes, using a Bayesian meta-regression tool, DisMod-MR 2.0. We obtained YLDs by multiplying the prevalence of different sequelae attached to causes with the disability weight attributed to the health state for that sequela. These disability weights were derived consistently from large population surveys across different and representative parts of the world and have been updated continuously through internet-based surveys, making them the most valid measure of disease severity available.^27^, ^35^

### Combined health loss and risk factors

The sum of YLLs and YLDs yields DALYs—a measure of overall health loss.^29^ DALYs combine the health effects of both fatal and non-fatal conditions, providing a common currency to facilitate useful comparisons across different causes of health loss. We calculated HALE using multiple-decrement life tables and YLDs by age, sex, location, and year.^29^

Another useful feature of GBD is the systematic quantification of health loss attributable to priority risk factors selected largely based on relevance to policy making and the availability of data to facilitate valid estimation.^26^ To estimate risk factors, meta-analyses of published research are used in which the relative risk of a specific risk factor for mortality or morbidity was calculated. Subsequently, the distribution of the same risk factor of interest is established across different locations, sexes, and age groups, as defined in GBD systematic hierarchy. We used counterfactual analysis to ascertain the health loss attributable to a specific risk factor by comparing the observed distribution of the risk factor in question with its theoretical minimum risk possible at the population level.^26^

Similar to the causes of health loss, risk factors are organised into a hierarchical structure. Three broad categories of behavioural, metabolic, and environmental and occupational risk factors are divided further into more detailed categories to facilitate useful comparisons. At all levels of aggregation, different ways of combining the effects of various risk factors are espoused, since their combined effects on health loss could be independent, joint, or mediated through a different factor that has to be taken into account.^26^ A GBD comparative risk assessment framework is used to estimate exposure, attributable deaths, and attributable DALYs by age, sex, and location-year.^36^ Data for relative risks and exposure come mainly from published studies, surveys, and censuses that meet quality criteria.

### Subnational estimation

We applied the standard GBD subnational estimation process to produce county-level estimates in Kenya. In short, subnational locations undergo the same estimation process as is used for country-level estimates. In locations with scant data, we used covariate estimation to borrow strength from similar subnational, country, region, and super-region models. The same spatiotemporal Gaussian process regression and ensemble modelling processes are used for subnational locations as for country-level modelling. If subnational locations are estimated, national estimations are based on the sum of the subnational values (the subnational values are not derived from a precalculated national value).

### Socio-demographic Index

The Socio-demographic Index (SDI) is a summary indicator calculated as the mean of the scaled values of total population fertility, educational attainment in the population older than 15 years, and per-capita income.^7^, ^28^ The SDI for Kenya in 2016 was 0·52 on a theoretical scale of 0–1.^7^ We used Gaussian regression methods to estimate the relation between SDI and each age, sex, cause, and health measure, then we used these relations to estimate expected values based on SDI alone for each age, sex, location, and year.

### Role of the funding source

The funder had no role in study design, data collection, data analysis, data interpretation, or writing of the report. All authors had full access to study data and had final responsibility for the decision to submit for publication.

## Results

**Figure 1:**
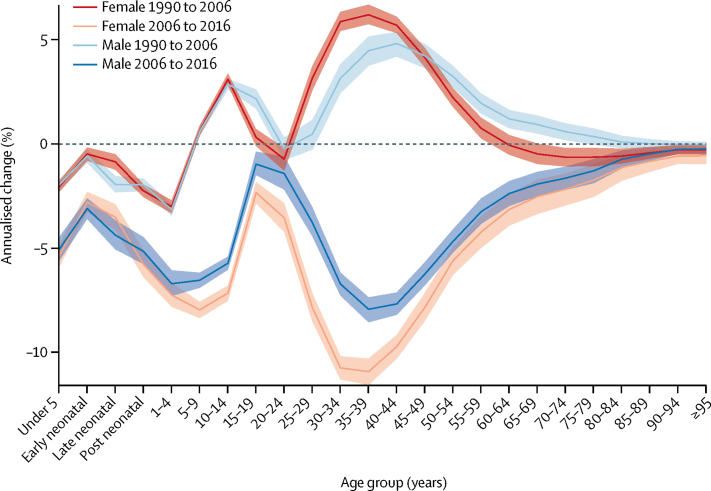
Annualised percentage change in all-cause mortality rates in Kenya, by sex Each coloured line represents the annualised percentage change in all-cause mortality rate for males and females across all age ranges. Shaded areas indicate 95% uncertainty intervals. Early neonatal=age 0–6 days. Late neonatal=age 7–27 days. Post neonatal=age 28–364 days.

Between 1990 and 2006, the all-cause mortality rate in Kenya increased from 850·3 (95% UI 829·8–871·1) deaths per 100 000 to 902·9 (881·6–924·1) deaths per 100 000. This trend was reversed between 2006 and 2016, when the all-cause mortality rate declined to 579·0 (95% UI 562·1–596·0) deaths per 100 000 (appendix p 127). Figure 1 shows the annualised percentage change in all-cause mortality rates by age and sex in both periods. From 1990 to 2006, the highest increase in mortality was estimated for females aged 35–39 years, at 6·2% (95% UI 5·7–6·7), and for males aged 40–44 years, at 4·8% (4·2–5·4). In this same period, changes in mortality for males and females were similar up to age 10–14 years, at which time a wide divergence was noted between sexes; mortality in males aged 15–19 years increased more rapidly than in females in that age group, whereas mortality in females aged 25–44 years increased more rapidly than in males. Mortality trends reconverged later in life, from age 85 and older, as mortality rates declined. From 2006 to 2016, mortality declined across all age groups, although males and females aged 15–19 years and 20–24 years lagged behind. The fastest decline was observed among females aged 35–39 years, with an annualised percentage decrease of 10·9% (95% UI 10·3–11·5).

At the county level, trends in all-cause age-standardised mortality rates varied from 1990 to 2006, but dropped consistently from 2006 to 2016 (appendix pp 128, 129). The fastest annualised decline in mortality between 2006 and 2016 was in Siaya (–6·7% [95% UI −9·1 to −4·4]) whereas the slowest change was in Mandera (–1·7% [–3·1 to −0·2]).

The national under-5 mortality ratio dropped from 95·4 (95% UI 90·1–101·3) deaths per 1000 livebirths in 1990 to 43·4 (36·9–51·2) deaths per 1000 livebirths in 2016 (appendix pp 130, 131). The decline was steeper from 2006 to 2016, with an annualised rate of change of −4·8% (95% UI −6·6 to −3·1), compared with a change of −1·9% (–2·6 to −1·3) from 1990 to 2006. Performance across counties was heterogeneous in both periods, with the fastest changes in mortality in West Pokot from 1990 to 2006 (–7·0% [95% UI −7·8 to −6·2]) and in Siaya from 2006 to 2016 (–7·6% [–10·1 to −5·1]) and the slowest changes in Nyandarua from 1990 to 2006 (2·1% [0·7 to 3·6]) and in Meru from 2006 to 2016 (–0·2% [–1·8 to 1·5]).

The maternal mortality ratio also improved nationally from 2006, increasing from 315·7 (95% UI 242·9–399·4) deaths per 100 000 livebirths in 1990 to 341·7 (272·0–422·8) deaths per 100 000 livebirths in 2006, then decreasing to 257·6 (195·1–335·3) deaths per 100 000 livebirths in 2016 (appendix pp 130, 131). Greater declines were observed after 2006, with an annualised percentage change of 0·5% (95% UI −0·4 to 1·6) from 1990 to 2006 and of −2·8% (–4·5 to −1·3) from 2006 to 2016. At the county level, from 1990 to 2006, the fastest decline in maternal mortality occurred in Kitui, with an annualised percentage change of −3·2% (95% UI −6·0 to −0·4), whereas maternal mortality increased in 25 counties, most substantially in Kirinyaga (4·9% [1·5 to 8·4]). From 2006 to 2016, the fastest decline in maternal mortality was in Siaya, with a percentage change of −8·2% (95% UI −14·5 to −2·4), and small increases in maternal mortality were recorded in Bungoma, Kakamega, Nandi, Taita-Taveta, and Vihiga counties.

**Figure 2:**
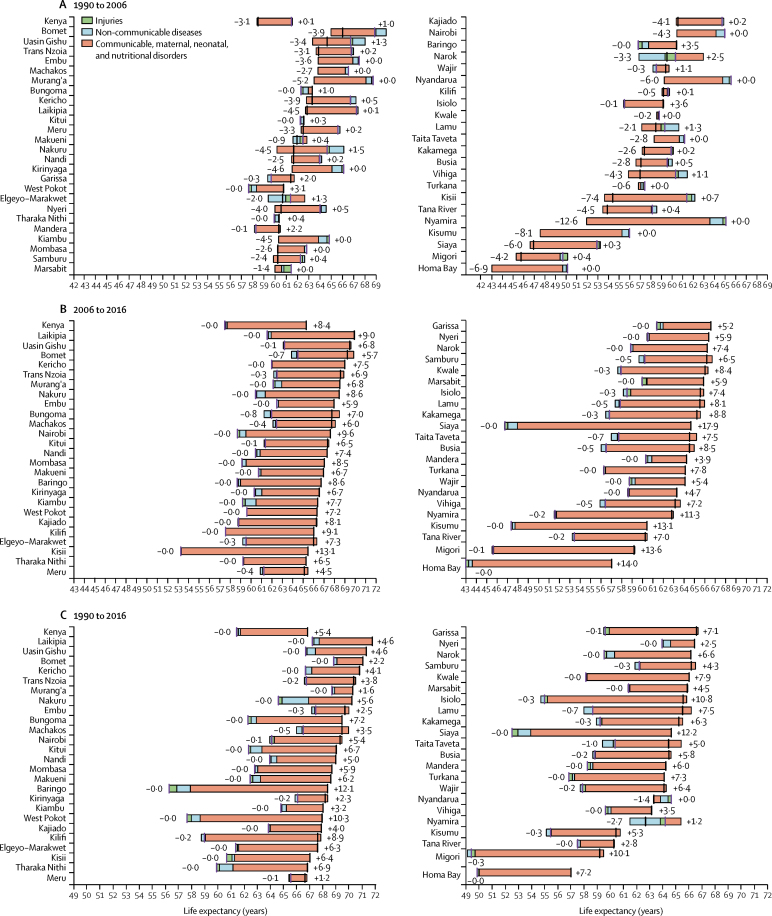
Attribution of changes in life expectancy in Kenya and its counties to changes in major groups of causes of death Periods shown are (A) 1990 to 2006, (B) 2006 to 2016, and (C) 1990 to 2016. Life expectancy is shown for both sexes. Life expectancy at the beginning of each period is indicated by a purple bar; life expectancy at the end of each period is indicated by a black bar. Counties are listed in decreasing order of life expectancy at the end of each period.

Nationally, life expectancy at birth increased by 5·4 (95% UI 3·7–7·2) years between 1990 and 2016, from 61·4 (95% UI 60·8–62·0) years in 1990 to 66·8 (66·1–67·6) years in 2016. This rise in life expectancy at birth was attributable largely to improvements in CMNN disease mortality (figure 2; appendix pp 132, 133). By contrast, nationally, life expectancy at birth decreased by 3·0 (95% UI 2·7–3·3) years between 1990 and 2006, from 61·4 (60·8–62·0) years in 1990 to 58·5 (57·9–59·1) years in 2006, largely due to increasing rates of CMNN disease mortality. The observed improvements in life expectancy between 1990 and 2016 are, thus, attributable to even larger improvements in life expectancy between 2006 and 2016, by 8·4 (95% UI 8·1–8·8) years, from 58·5 years (57·9–59·1) in 2006 to 66·8 (66·1–67·6) years in 2016.

Although this striking pattern is mirrored in many counties, considerable heterogeneity is seen across the period. Nyandarua and Nyamira both showed a decrease in life expectancy between 1990 and 2016, whereas Baringo, West Pokot, Isiolo, and Siaya all increased life expectancy by more than 10 years (appendix pp 132, 133). In 2016, three counties had a life expectancy greater than 71 years, which were Laikipia (71·8 [95% UI 68·2–75·4] years), Uasin Gishu (71·3 [68·6–74·0] years), and Bomet (71·1 [68·4–73·5] years). At the same time, two counties had a life expectancy lower than 60 years, which were Migori (59·2 [95% UI 56·0–63·0] years) and Homa Bay (57·0 [54·4–60·5] years).

Between 1990 and 2006, 38 of 47 counties showed a decrease in life expectancy. In 2006, 22 counties had a life expectancy below 60 years and four counties had a life expectancy below 50 years, which were Kisumu (47·4 [95% UI 44·9–50·3] years), Siaya (46·8 [43·9–49·9] years), Migori (45·7 [42·9–48·7] years), and Homa Bay (43·0 [40·7–45·6] years). Six counties gained more than 10 years in life expectancy between 2006 and 2016. Life expectancy in Siaya increased by 17·9 years between 2006 and 2016, from 46·8 (95% UI 43·9–49·9) years in 2006 to 64·7 (61·9–68·2) years in 2016.

**Figure 3:**
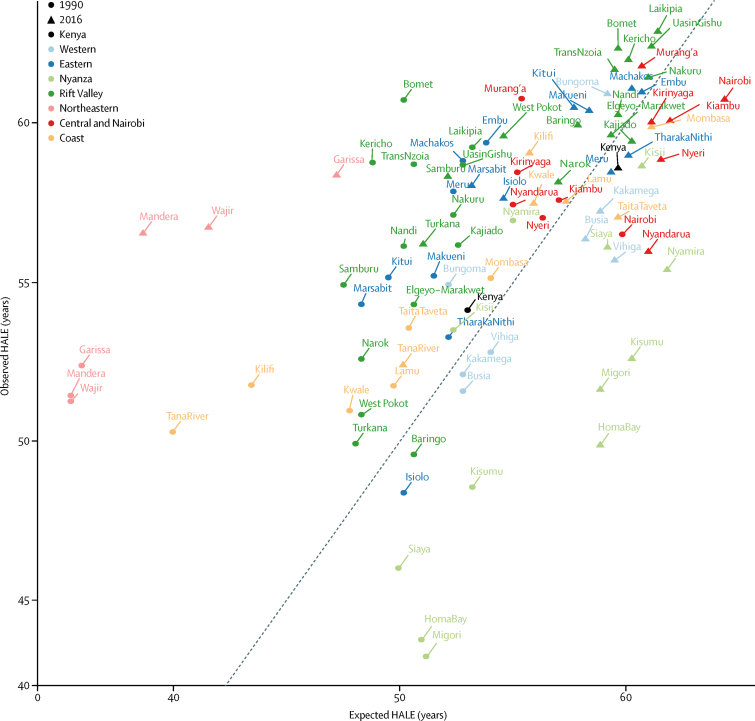
Observed and expected HALE in Kenya and its counties in 1990 and 2016 Observed and expected HALEs are based on SDI for both sexes. Counties are grouped in colours by former provinces. The black line represents 1:1 observed to expected HALE. Points above the line have higher HALE than predicted by SDI; points below the line have lower HALE than predicted by SDI. HALE=healthy average life expectancy. SDI=Socio-demographic Index.

HALE increased nationally between 1990 and 2016, from 54·1 (95% UI 51·8–56·1) years in 1990 to 58·6 (56·0–60·7) years in 2016 (figure 3; appendix pp 134, 135). However, comparing the ratio of observed to expected HALE based on SDI suggests a significant national attainment gap in the year 2016, with a mixed picture at county level. Nairobi and most counties in the former Central province had lower HALE than would be expected in 2016, along with all counties in the former Nyanza and Western provinces, with the exception of Bungoma. All counties in the former Northeastern province had HALE above what would be expected based on SDI in 2016, and HALE was highest overall and higher than expected in 2016 in most counties in the former Rift Valley province.

**Figure 4:**
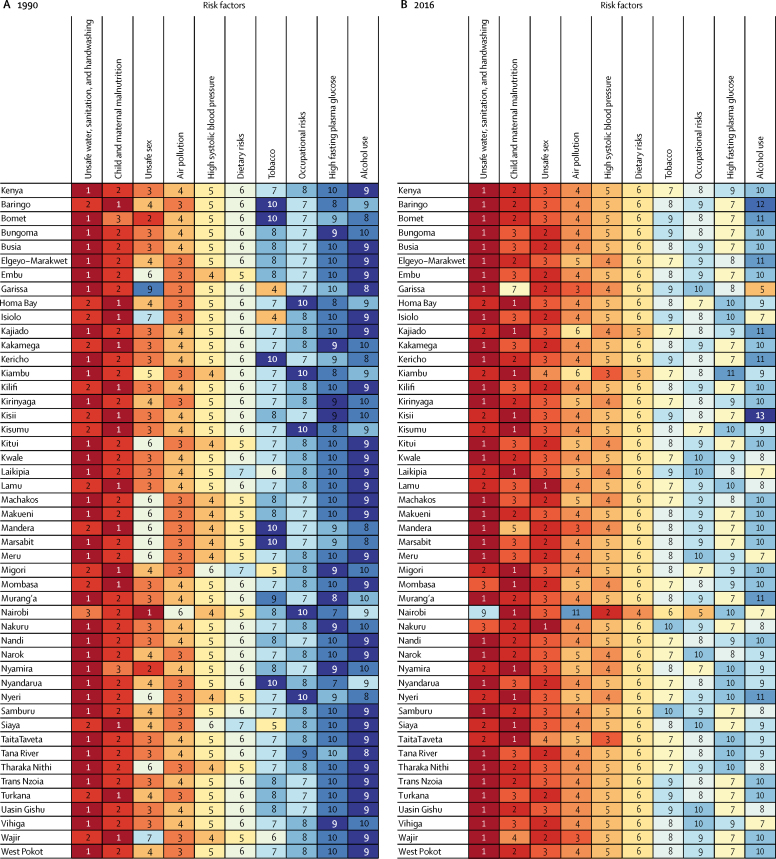
Rankings of leading risk factors attributable to age-standardised DALYs in Kenya and its counties GBD level 2 risk factors in (A) 1990 and (B) 2016 are ranked from 1 (leading) to 10 or more (lowest) and are based on attributable age-standardised DALYs in both sexes. DALYs=disability-adjusted life-years. GBD=Global Burden of Diseases, Injuries, and Risk Factors Study.

At the national level, unsafe water, sanitation, and handwashing, child and maternal malnutrition, and unsafe sex were the leading risk factors contributing to age-standardised DALYs in 1990 (figure 4A; appendix p 136). In 2016, unsafe water, sanitation, and handwashing remained the leading risk factor, with unsafe sex the second leading risk factor and child and maternal malnutrition the third leading risk (figure 4B; appendix p 136). Nairobi showed a different pattern: high systolic blood pressure and alcohol and drug use were the second and third leading risk factors, whereas unsafe water, sanitation, and handwashing and air pollution had less important roles than in other counties. Unsafe sex and alcohol and drug use were much lower risks in Garissa county, whereas tobacco was a much higher risk compared with other counties. Overall, despite the continued predominance of risk factors related to communicable diseases, trends suggest an increasing priority of risk factors related to NCDs over the 26-year period.

**Figure 5:**
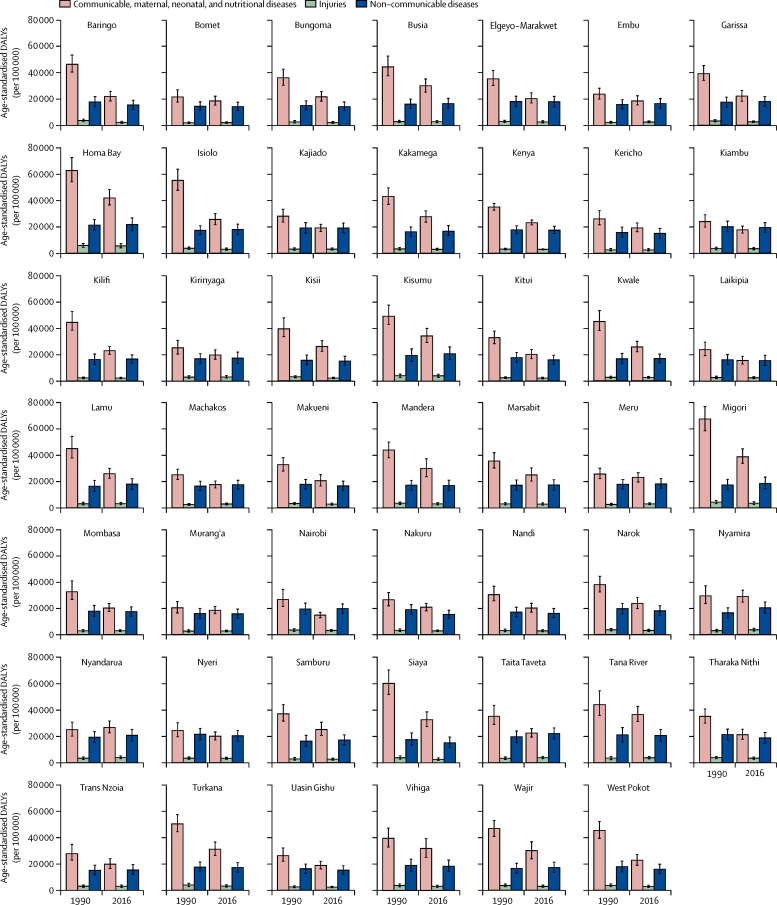
Age-standardised rates of DALYs in Kenyan counties in 1990 and 2016 for both sexes DALYs=disability-adjusted life-years.

DALYs incorporate both morbidity and mortality and, thus, provide an overall assessment of health loss. In 1990, CMNN causes were the largest drivers of rates of DALYs in every county in Kenya (figure 5; appendix pp 137, 138). In 1990, the highest rates of DALYs attributable to CMNN causes were in Migori (68 800 [95% UI 59 600–78 300] per 100 000) and Homa Bay (62 300 [54 000–72 300] per 100 000), and the highest rates of DALYs attributable to NCDs were in Nyeri (21 500 [95% UI 17 100–25 900] per 100 000) and Homa Bay (21 200 [17 200–25 500] per 100 000). By 2016, the highest rate of DALYs attributable to CMNN causes was in Homa Bay, but it had decreased to 41 800 (95% UI 36 300–47 900) per 100 000. In every county, the proportion of DALYs attributable to NCDs increased between 1990 and 2016. The highest rates of DALYs attributable to NCDs in 2016 were in Taita-Taveta (22 000 [95% UI 18 000–26 200] per 100 000) and Homa Bay (21 500 [16 800–26 500] per 100 000). In three counties (Nyeri, Kiambu, and Nairobi) the rates of DALYs attributable to NCDs were higher than those attributable to CMNN causes.

**Figure 6:**
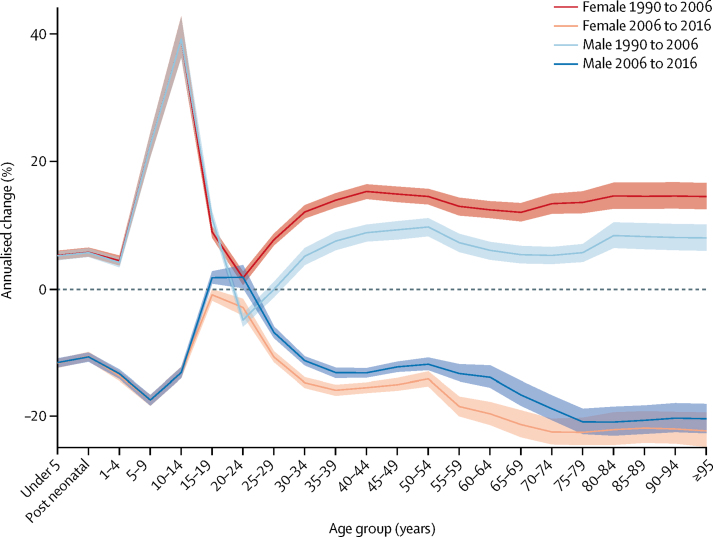
Annualised percentage change in HIV/AIDS-specific mortality rates for males and females Each coloured line represents the annualised percentage change in HIV/AIDS mortality for males and females across all age ranges. Shaded areas indicate 95% uncertainty intervals. Early neonatal and late neonatal are not estimated for HIV/AIDS. Post neonatal=age 28–364 days.

Figure 6 shows the annualised percentage change in HIV/AIDS-specific mortality rates between 1990 and 2006 and between 2006 and 2016. In tandem with all-cause mortality trends, most age groups had increasing HIV/AIDS-specific mortality for the period 1990 to 2006, with the exception of males aged 25–29 years, who had stable rates, and males aged 20–24 years, who saw a decrease in mortality of 4·9% (95% UI −5·9 to −3·9). For both sexes, children aged 10–14 years had the largest increase in HIV/AIDS-specific mortality, at 39·1% (95% UI 36·5 to 42·8) in males and 39·1% (36·6 to 42·8) in females. Trends for males and females were similar up to the age of 20–24 years, at which time they diverged, with females registering higher rates of increasing mortality through older age. By contrast, in the period 2006–16, all age groups had declining rates of HIV/AIDS-specific mortality, with the exception of males aged 15–19 years and 20–24 years, who had an increase in mortality rates of 1·8% (95% UI 0·9 to 2·8) and 1·8% (0·2 to 3·8), respectively.

The HIV/AIDS-specific mortality rate increased in all counties from 1990 to 2006 (appendix pp 128, 129), with the largest annualised increase in Embu (13·0% [95% UI 6·8 to 21·8]) and the smallest increase in Nairobi (6·0% [2·0 to 12·3]). Between 2006 and 2016, a consistent drop in HIV/AIDS-specific mortality rates was noted in all counties, with the fastest change registered in Siaya (–15·3% [–17·3 to −12·2]) and the slowest in Wajir (–4·4% [–6·5 to −2·4]).

**Figure 7:**
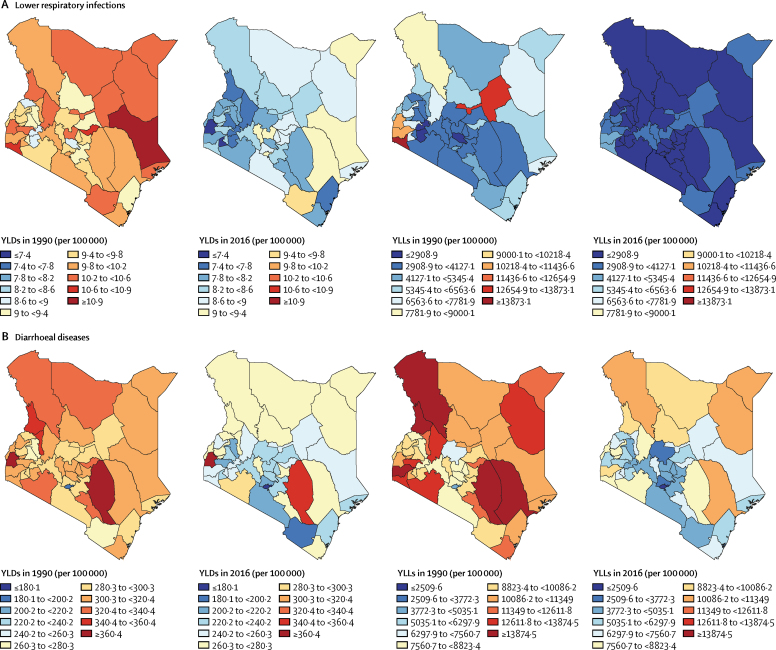
Age-standardised rates of YLLs and YLDs in Kenya Rates of YLLs and YLDs in 1990 and 2016 attributable to (A) lower respiratory infections and (B) diarrhoea are shown for both sexes by county. LRI=lower respiratory infection. YLD=years lived with disability. YLL=years of life lost.

Between 1990 and 2016, rates of YLLs and YLDs for diarrhoea, and rates of YLDs for lower respiratory infections, decreased for every county in Kenya (figure 7; appendix pp 139, 140), whereas YLLs for lower respiratory infections increased in Nyandurya, Meru, Nakura, and Boment counties. Heterogeneity was noted between counties across diseases and metrics. In 2016, age-standardised rates of YLLs for lower respiratory infections ranged from 1690·7 (95% UI 1173·8–2372·7) per 100 000 in Laikipia to 4640·5 (3514·0–5818·2) per 100 000 in Homa Bay (figure 7A); rates of YLLs for diarrhoea were much wider in range, from 1246·9 (95% UI 650·9–1984·0) per 100 000 in Nairobi to 10 779·7 (6335·4–16 413·2) in Wajir (figure 7B). In 2016, Siaya reported the highest rate of YLDs for diarrhoea (364·7 [95% UI 254·2–495·0] per 100 000), but the lowest rate of YLDs for lower respiratory infections (7·2 [4·7–10·3] per 100 000). The highest rate of YLDs attributable to lower respiratory infections in 2016 was reported in Taita-Taveta (9·6 [95% UI 6·3–13·6] per 100 000), and this county also reported the second lowest rate of YLDs attributable to diarrhoea (199·7 [137·1–272·6] per 100 000).

Rates of YLDs and YLLs attributable to malaria decreased steadily in every county in Kenya between 1990 and 2016 (appendix pp 139, 140). However, these rates were higher in counties near Lake Victoria compared with other parts of Kenya.

## Discussion

Our results show that, in the period 2006–16, Kenya made considerable progress in reducing the burden of communicable diseases, including HIV/AIDS, diarrhoeal diseases, lower respiratory infections, and malaria. This trend is especially striking when compared with the period 1990–2006, during which time mortality from CMNN causes rose. This turnaround is testament to many health interventions that have been introduced in the early 2000s and then scaled up, including expansion of HIV/AIDS treatment, malaria control, programmes for maternal and child health (including childhood immunisation and free maternity services), scale-up of rotavirus and pneumococcal immunisation programmes, and slight improvements in access to safe water and sanitation.^11^, ^17^, ^37^, ^38^, ^39^, ^40^ Sustained declines in under-5 mortality further reflect these successes.

The decline in HIV/AIDS mortality since 2006 in Kenya is attributable largely to the successful rollout of prevention and treatment options across the country, in tandem with global efforts.^38^, ^41^ However, HIV/AIDS-specific mortality rates in young men have continued to increase since 2006, which have probably affected overall mortality trends. Low levels of knowledge about sexual behaviours causing exposure to HIV, disempowerment of young women to make decisions about sexual behaviour, and use of alcohol and drugs could contribute to higher rates of HIV/AIDS mortality in younger age groups.^38^ The Kenyan Government's cash transfer programme has successfully reduced the odds of early sexual debut, potentially by keeping children in school, but no effect has been noted on condom use or number of partners.^42^ The fact that HIV/AIDS is still a dominant contributor to overall health loss in Kenya highlights the need for sustained efforts to expand and improve the quality of prevention and treatment to achieve health gains in line with global targets. High rates of co-infection with HIV and tuberculosis indicate that combined interventions might maximise the effect on improving population health outcomes.^43^, ^44^ The declining importance of malaria across endemic counties attests positively to Kenyan efforts to scale up malaria treatment and prevention interventions, although recent trends indicate that further interventions to expand testing and treatment are needed.^17^, ^37^, ^45^, ^46^

Water, sanitation, and hygiene continue to be important contributors to the burden of DALYs, and public health interventions should continue to target these risk factors throughout Kenya. Care should be taken, however, because findings of studies have suggested that the success of targeted interventions might be highly specific to local conditions.^47^, ^48^, ^49^ The rise of unsafe sex as a leading risk also points to the need for increased health education for behaviour change.^50^, ^51^ Without improved practice, it is unlikely that HIV/AIDS-specific mortality will continue to decline, as seen in the trends in younger age groups. While malnutrition remains an important risk, this factor now affects health alongside a growing proportion of metabolic risks contributing to DALYs for NCDs—eg, high systolic blood pressure and dietary risks.

Although the health landscape in Kenya continues to be dominated by communicable diseases, the increase in the relative contribution of NCDs to overall health loss is cause for concern. There is a real risk of erosion of population health gains realised over the past 25 years if these growing burdens are left unchecked—particularly when considering the changing distribution of priority risk factors for NCDs in some counties.

Addressing the burden of NCDs such as ischaemic heart disease and cerebrovascular disease requires an integrated approach, including prevention, treatment, and rehabilitation measures. Tackling related risk factors (eg, obesity, smoking, and hypertension) offers cost-effective ways to combat some of the priority NCDs. Lifestyle changes (eg, promotion of a healthy diet and increased physical activity) are also needed to improve population health.^18^ A 2010 study found high levels of risk for cardiovascular disease in Kenya, including hypertension, diabetes, and cholesterol, particularly in urban areas.^52^ Further, a large proportion of the urban population in Kenya resides in impoverished and informal neighbourhoods where access to health care is limited, presenting an additional challenge for controlling NCDs in this large population.^53^

In addition to health promotion, the health system in Kenya must be reoriented to respond to emerging health needs related to NCDs. In a study of health systems in east Africa, major response gaps were identified at the primary health care level with respect to common NCDs,^54^ whereas findings of other studies from Kenya have identified barriers to optimum health-care provision in hospitals.^55^, ^56^ To be effective, and for Kenya to meet the Sustainable Development Goals in all counties, various components of the health system—ranging from human resources and medical supplies to infrastructure and health data systems—require attention.

Kenya has begun to take steps to address the burden of NCDs and was one of the first countries in sub-Saharan Africa to introduce specific targets for NCDs into health policy.^21^, ^57^ Efforts to achieve new targets will need to be balanced with continued emphasis on the major contributors to the current health burden to maximise total health gains. Moreover, the diversity of risks, health system capacity, and current burden at the county level will require targeted approaches.

Although health outcomes remain heterogeneous at the county level in Kenya, many health metrics have converged across counties from 1990 to 2016. In 1990, a gap of 19 years was recorded between counties with the lowest and highest life expectancy, and by 2016 that gap had narrowed to 15 years.

Maternal mortality—a priority area for the health system—has registered impressive gains over time, particularly since 2006. Gains at the subnational level are heterogeneous, however, revealing relevant performance gaps to be addressed. The same applies to under-5 mortality, with some counties showing declines of greater than 5% every year, whereas in other counties the under-5 mortality rate has largely been stable over time.

The heterogeneous burden of HIV/AIDS, lower respiratory infections, diarrhoea, and malaria across Kenyan counties highlights further the need for localised public health interventions. For example, in 2016, the highest rate of YLDs for diarrhoea was reported in the county of Siaya, but the rate of YLLs for diarrhoea in this county was one of the lowest in 2016. This finding suggests that, although the burden of mild and moderate disease was high, cases tended to resolve instead of progressing to death. In Taita-Taveta county, both the highest rate of YLDs for lower respiratory infections and the second lowest rate of YLDs for diarrhoea were reported in 2016. One-size-fits-all public health policies are unlikely to improve the health of counties facing such divergent challenges. Instead, county governments should develop specific public health interventions tailored to the challenges faced by their local communities.

The lower magnitude of health loss attributable to unsafe sex and alcohol in Garissa and Wajir compared with other counties could reflect beliefs and practices of the majority Muslim population in those counties. Large Muslim populations are associated with lower levels of HIV prevalence in African countries.^58^ Tobacco is a leading risk in Garissa and Isiolo counties, highlighting the need for increased tobacco control efforts in those regions. Overall, although interventions are needed to tackle leading risks at the national level, counties would benefit from more tailored intervention approaches. Benefits of further local burden of disease estimation may be substantial given the large subnational differences observed.^59^, ^60^, ^61^

Health interventions alone do not dictate health outcomes: diverse geography, socioeconomic status, and other social determinants of health all contribute to the heterogeneity in outcomes at the subnational and local levels.^62^, ^63^, ^64^, ^65^, ^66^, ^67^ Infrastructure investments to improve water quality and sanitation and regulations to improve air pollution and workplace safety can have direct effects on the health of citizens without being strict health interventions. In Kenya, special attention will need to be paid to meeting health needs in both urban and rural communities.^53^, ^68^, ^69^, ^70^

Comparison of population health outcomes over time allows decision makers to assess performance objectively across different health delivery units. To date, our analysis is the first and most comprehensive assessment of population health status in Kenya, taking into account trends in mortality, morbidity, and risk factors at the national and subnational levels.

Our study has various limitations to the interpretation of the findings and is subject to all the limitations outlined in GBD 2016.^7^, ^26^, ^27^, ^28^, ^29^ National datasets covering the entire time series and subnational locations were sparse, requiring modelling to fill data gaps, particularly at the county level. Furthermore, some datasets had biases or errors, such as the misclassification of causes of death or assignment of deaths to causes that cannot be primary causes of death, termed garbage codes. Standardised empirical procedures were applied to mitigate for these issues, and these efforts and their potential limitations have been described in detail elsewhere.^7^, ^26^, ^27^, ^28^, ^29^ To compensate for this data scarcity, we have limited our detailed discussions to the diseases for which local data were available specifically (HIV/AIDS, lower respiratory infections, diarrhoea, and malaria).

Our study builds the foundation for further research in benchmarking and health system performance assessment in Kenya. In particular, further research into the effect of the growing economy and increased investment in health, and the effect of disasters related to climate change, could help to identify if and how these factors have contributed to changing health patterns. Moreover, analyses of the effect of devolution on health-care provision and related outcomes could help to identify gaps and improve future services at the county level.

By unmasking the subnational health disparities that are often concealed by aggregate national estimates, our study underscores the need for health system stewards to have an in-depth understanding of detailed population health trends so that they can focus policy attention appropriately. Despite improvements, Kenya faces a large burden of communicable disease, while trends point towards future increases in NCD burden. Focusing on important risk factors associated with health loss—including water, sanitation, and hygiene, unsafe sex, and malnutrition—can enable policy makers to intervene effectively and realise better returns on investment. By pointing to unique opportunities to improve health outcomes at the county level, our study serves as an effective advocacy tool for further investment in population health.
