# Supplementary material for: Health disparities across the counties of Kenya and implications for policy makers, 1990–2016: a systematic analysis for the Global Burden of Disease Study 2016
Source: Lancet Glob Health. 2018 Oct 25;7(1):e81–95. doi: 10.1016/S2214-109X(18)30472-8 (PMC6293072; doi:10.1016/S2214-109X(18)30472-8)
Supplement: Supplementary appendix [file mmc1.pdf]

THE LANCET
Global Health

Supplementary appendix

This appendix formed part of the original submission and has been peer reviewed. We post it as supplied by the authors.

Supplement to: Achoki T, Miller-Petrie MK, Glenn SD, et al. Health disparities across the counties of Kenya and implications for policy makers, 1990–2016: a systematic analysis for the Global Burden of Disease Study 2016. *Lancet Glob Health* 2018; published online Oct 25. http://dx.doi.org/10.1016/S2214-109X(18)30472-8.

Appendix Table 1: CODEm covariates used and expected direction of covariate by cause

Cause	Sex	Age start	Age end	Direction	Covariate
Tuberculosis	Male	28-364 days	95+ years	1	Alcohol (liters per capita)
Tuberculosis	Male	28-364 days	95+ years	1	Diabetes Fasting Plasma Glucose (mmol/L)
Tuberculosis	Male	28-364 days	95+ years	-1	Education (years per capita)
Tuberculosis	Male	28-364 days	95+ years	-1	LDI (I$ per capita)
Tuberculosis	Male	28-364 days	95+ years	1	Indoor Air Pollution (All Cooking Fuels)
Tuberculosis	Male	28-364 days	95+ years	1	Outdoor Air Pollution (PM2.5)
Tuberculosis	Male	28-364 days	95+ years	1	Population Density (500-1000 ppl/sqkm, proportion)
Tuberculosis	Male	28-364 days	95+ years	1	Population Density (over 1000 ppl/sqkm, proportion)
Tuberculosis	Male	28-364 days	95+ years	1	Smoking Prevalence
Tuberculosis	Male	28-364 days	95+ years	1	Log-transformed SEV scalar: TB
Tuberculosis	Male	28-364 days	95+ years	-1	Socio-demographic Index
Tuberculosis	Male	28-364 days	95+ years	-1	Healthcare access and quality index
Tuberculosis	Male	28-364 days	95+ years	1	Age-standardized proportion adult underweight
Tuberculosis	Male	28-364 days	95+ years	1	Tuberculosis infection risk-weighted prevalence (age-standardized)
Tuberculosis	Male	28-364 days	95+ years	1	Tuberculosis prevalence (age-standardized)
Tuberculosis	Female	28-364 days	95+ years	1	Alcohol (liters per capita)
Tuberculosis	Female	28-364 days	95+ years	1	Diabetes Fasting Plasma Glucose (mmol/L)
Tuberculosis	Female	28-364 days	95+ years	-1	Education (years per capita)
Tuberculosis	Female	28-364 days	95+ years	-1	LDI (I$ per capita)
Tuberculosis	Female	28-364 days	95+ years	1	Indoor Air Pollution (All Cooking Fuels)
Tuberculosis	Female	28-364 days	95+ years	1	Outdoor Air Pollution (PM2.5)
Tuberculosis	Female	28-364 days	95+ years	1	Population Density (500-1000 ppl/sqkm, proportion)
Tuberculosis	Female	28-364 days	95+ years	1	Population Density (over 1000 ppl/sqkm, proportion)
Tuberculosis	Female	28-364 days	95+ years	1	Smoking Prevalence
Tuberculosis	Female	28-364 days	95+ years	1	Log-transformed SEV scalar: TB
Tuberculosis	Female	28-364 days	95+ years	-1	Socio-demographic Index
Tuberculosis	Female	28-364 days	95+ years	-1	Healthcare access and quality index
Tuberculosis	Female	28-364 days	95+ years	1	Age-standardized proportion adult underweight
Tuberculosis	Female	28-364 days	95+ years	1	Tuberculosis infection risk-weighted prevalence (age-standardized)
Tuberculosis	Female	28-364 days	95+ years	1	Tuberculosis prevalence (age-standardized)
Diarrheal diseases	Male	5-9 years	95+ years	-1	Education (years per capita)
Diarrheal diseases	Male	5-9 years	95+ years	-1	LDI (I$ per capita)
Diarrheal diseases	Male	5-9 years	95+ years	-1	Mean BMI
Diarrheal diseases	Male	5-9 years	95+ years	0	Population Density (over 1000 ppl/sqkm, proportion)
Diarrheal diseases	Male	5-9 years	95+ years	-1	Sanitation (proportion with access)
Diarrheal diseases	Male	5-9 years	95+ years	-1	Improved Water Source (proportion with access)
Diarrheal diseases	Male	5-9 years	95+ years	1	Log-transformed SEV scalar: Diarrhea
Diarrheal diseases	Male	5-9 years	95+ years	1	SEV unsafe water
Diarrheal diseases	Male	5-9 years	95+ years	1	SEV unsafe sanitation
Diarrheal diseases	Male	5-9 years	95+ years	-1	Socio-demographic Index
Diarrheal diseases	Male	5-9 years	95+ years	-1	Rotavirus coverage (proportion)
Diarrheal diseases	Male	5-9 years	95+ years	-1	Healthcare access and quality index
Diarrheal diseases	Female	5-9 years	95+ years	-1	Education (years per capita)
Diarrheal diseases	Female	5-9 years	95+ years	-1	LDI (I$ per capita)
Diarrheal diseases	Female	5-9 years	95+ years	-1	Mean BMI
Diarrheal diseases	Female	5-9 years	95+ years	0	Population Density (over 1000 ppl/sqkm, proportion)
Diarrheal diseases	Female	5-9 years	95+ years	-1	Sanitation (proportion with access)
Diarrheal diseases	Female	5-9 years	95+ years	-1	Improved Water Source (proportion with access)
Diarrheal diseases	Female	5-9 years	95+ years	1	Log-transformed SEV scalar: Diarrhea
Diarrheal diseases	Female	5-9 years	95+ years	1	SEV unsafe water
Diarrheal diseases	Female	5-9 years	95+ years	1	SEV unsafe sanitation
Diarrheal diseases	Female	5-9 years	95+ years	-1	Socio-demographic Index
Diarrheal diseases	Female	5-9 years	95+ years	-1	Rotavirus coverage (proportion)
Diarrheal diseases	Female	5-9 years	95+ years	-1	Healthcare access and quality index
Diarrheal diseases	Male	0-6 days	1-4 years	-1	LDI (I$ per capita)
Diarrheal diseases	Male	0-6 days	1-4 years	1	Underweight (proportion <2SD weight for age, <5 years)
Diarrheal diseases	Male	0-6 days	1-4 years	0	Population Density (over 1000 ppl/sqkm, proportion)
Diarrheal diseases	Male	0-6 days	1-4 years	0	Population Density (under 150 ppl/sqkm, proportion)
Diarrheal diseases	Male	0-6 days	1-4 years	-1	Sanitation (proportion with access)
Diarrheal diseases	Male	0-6 days	1-4 years	-1	Improved Water Source (proportion with access)
Diarrheal diseases	Male	0-6 days	1-4 years	1	Vitamin A Deficiency Prevalence (age-standardized)
Diarrheal diseases	Male	0-6 days	1-4 years	-1	Maternal education (years per capita)
Diarrheal diseases	Male	0-6 days	1-4 years	1	Log-transformed SEV scalar: Diarrhea
Diarrheal diseases	Male	0-6 days	1-4 years	1	SEV unsafe water
Diarrheal diseases	Male	0-6 days	1-4 years	1	SEV unsafe sanitation
Diarrheal diseases	Male	0-6 days	1-4 years	-1	Socio-demographic Index
Diarrheal diseases	Male	0-6 days	1-4 years	1	Stunting (proportion <2SD height for age, <5 years)
Diarrheal diseases	Male	0-6 days	1-4 years	1	Wasting (proportion <2SD weight for height, <5 years)
Diarrheal diseases	Male	0-6 days	1-4 years	-1	Rotavirus coverage (proportion)
Diarrheal diseases	Male	0-6 days	1-4 years	-1	Healthcare access and quality index
Diarrheal diseases	Male	0-6 days	1-4 years	1	No handwashing with soap
Diarrheal diseases	Male	0-6 days	1-4 years	1	Suboptimal breastfeeding SEV
Diarrheal diseases	Male	0-6 days	1-4 years	1	Zinc deficiency
Diarrheal diseases	Female	0-6 days	1-4 years	-1	LDI (I$ per capita)
Diarrheal diseases	Female	0-6 days	1-4 years	1	Underweight (proportion <2SD weight for age, <5 years)
Diarrheal diseases	Female	0-6 days	1-4 years	0	Population Density (over 1000 ppl/sqkm, proportion)
Diarrheal diseases	Female	0-6 days	1-4 years	0	Population Density (under 150 ppl/sqkm, proportion)
Diarrheal diseases	Female	0-6 days	1-4 years	-1	Sanitation (proportion with access)
Diarrheal diseases	Female	0-6 days	1-4 years	-1	Improved Water Source (proportion with access)
Diarrheal diseases	Female	0-6 days	1-4 years	1	Vitamin A Deficiency Prevalence (age-standardized)
Diarrheal diseases	Female	0-6 days	1-4 years	-1	Maternal education (years per capita)
Diarrheal diseases	Female	0-6 days	1-4 years	1	Log-transformed SEV scalar: Diarrhea
Diarrheal diseases	Female	0-6 days	1-4 years	1	SEV unsafe water
Diarrheal diseases	Female	0-6 days	1-4 years	1	SEV unsafe sanitation
Diarrheal diseases	Female	0-6 days	1-4 years	-1	Socio-demographic Index
Diarrheal diseases	Female	0-6 days	1-4 years	1	Stunting (proportion <2SD height for age, <5 years)
Diarrheal diseases	Female	0-6 days	1-4 years	1	Wasting (proportion <2SD weight for height, <5 years)
Diarrheal diseases	Female	0-6 days	1-4 years	-1	Rotavirus coverage (proportion)
Diarrheal diseases	Female	0-6 days	1-4 years	-1	Healthcare access and quality index
Diarrheal diseases	Female	0-6 days	1-4 years	1	No handwashing with soap
Diarrheal diseases	Female	0-6 days	1-4 years	1	Suboptimal breastfeeding SEV
Diarrheal diseases	Female	0-6 days	1-4 years	1	Zinc deficiency
Lower respiratory infections	Male	5-9 years	95+ years	1	Alcohol (liters per capita)
Lower respiratory infections	Male	5-9 years	95+ years	-1	DTP3 Coverage (proportion)

Cause	Sex	Age start	Age end	Direction	Covariate
Lower respiratory infections	Male	5-9 years	95+ years	-1	Education (years per capita)
Lower respiratory infections	Male	5-9 years	95+ years	-1	LDI (I$ per capita)
Lower respiratory infections	Male	5-9 years	95+ years	-1	Mean BMI
Lower respiratory infections	Male	5-9 years	95+ years	1	Indoor Air Pollution (All Cooking Fuels)
Lower respiratory infections	Male	5-9 years	95+ years	1	Outdoor Air Pollution (PM2.5)
Lower respiratory infections	Male	5-9 years	95+ years	1	Smoking Prevalence
Lower respiratory infections	Male	5-9 years	95+ years	-1	PCV3 Coverage (proportion)
Lower respiratory infections	Male	5-9 years	95+ years	1	Log-transformed SEV scalar: LRI
Lower respiratory infections	Male	5-9 years	95+ years	0	SEV unsafe sanitation
Lower respiratory infections	Male	5-9 years	95+ years	-1	Socio-demographic Index
Lower respiratory infections	Male	5-9 years	95+ years	-1	Healthcare access and quality index
Lower respiratory infections	Female	5-9 years	95+ years	1	Alcohol (liters per capita)
Lower respiratory infections	Female	5-9 years	95+ years	-1	DTP3 Coverage (proportion)
Lower respiratory infections	Female	5-9 years	95+ years	-1	Education (years per capita)
Lower respiratory infections	Female	5-9 years	95+ years	-1	LDI (I$ per capita)
Lower respiratory infections	Female	5-9 years	95+ years	-1	Mean BMI
Lower respiratory infections	Female	5-9 years	95+ years	1	Indoor Air Pollution (All Cooking Fuels)
Lower respiratory infections	Female	5-9 years	95+ years	1	Outdoor Air Pollution (PM2.5)
Lower respiratory infections	Female	5-9 years	95+ years	1	Smoking Prevalence
Lower respiratory infections	Female	5-9 years	95+ years	-1	PCV3 Coverage (proportion)
Lower respiratory infections	Female	5-9 years	95+ years	1	Log-transformed SEV scalar: LRI
Lower respiratory infections	Female	5-9 years	95+ years	0	SEV unsafe sanitation
Lower respiratory infections	Female	5-9 years	95+ years	-1	Socio-demographic Index
Lower respiratory infections	Female	5-9 years	95+ years	-1	Healthcare access and quality index
Lower respiratory infections	Male	0-6 days	1-4 years	-1	DTP3 Coverage (proportion)
Lower respiratory infections	Male	0-6 days	1-4 years	-1	Hib3 Vaccine Coverage (proportion)
Lower respiratory infections	Male	0-6 days	1-4 years	-1	LDI (I$ per capita)
Lower respiratory infections	Male	0-6 days	1-4 years	1	Underweight (proportion <2SD weight for age, <5 years)
Lower respiratory infections	Male	0-6 days	1-4 years	1	Indoor Air Pollution (All Cooking Fuels)
Lower respiratory infections	Male	0-6 days	1-4 years	1	Outdoor Air Pollution (PM2.5)
Lower respiratory infections	Male	0-6 days	1-4 years	-1	PCV3 Coverage (proportion)
Lower respiratory infections	Male	0-6 days	1-4 years	1	Vitamin A Deficiency Prevalence (age-standardized)
Lower respiratory infections	Male	0-6 days	1-4 years	-1	Maternal education (years per capita)
Lower respiratory infections	Male	0-6 days	1-4 years	1	Log-transformed SEV scalar: LRI
Lower respiratory infections	Male	0-6 days	1-4 years	1	SEV unsafe sanitation
Lower respiratory infections	Male	0-6 days	1-4 years	-1	Socio-demographic Index
Lower respiratory infections	Male	0-6 days	1-4 years	1	Stunting (proportion <2SD height for age, <5 years)
Lower respiratory infections	Male	0-6 days	1-4 years	1	Wasting (proportion <2SD weight for height, <5 years)
Lower respiratory infections	Male	0-6 days	1-4 years	-1	Healthcare access and quality index
Lower respiratory infections	Male	0-6 days	1-4 years	1	No handwashing with soap
Lower respiratory infections	Male	0-6 days	1-4 years	1	Suboptimal breastfeeding SEV
Lower respiratory infections	Male	0-6 days	1-4 years	1	Zinc deficiency
Lower respiratory infections	Male	0-6 days	1-4 years	1	Secondhand smoke
Lower respiratory infections	Female	0-6 days	1-4 years	-1	DTP3 Coverage (proportion)
Lower respiratory infections	Female	0-6 days	1-4 years	-1	Hib3 Vaccine Coverage (proportion)
Lower respiratory infections	Female	0-6 days	1-4 years	-1	LDI (I$ per capita)
Lower respiratory infections	Female	0-6 days	1-4 years	1	Underweight (proportion <2SD weight for age, <5 years)
Lower respiratory infections	Female	0-6 days	1-4 years	1	Indoor Air Pollution (All Cooking Fuels)
Lower respiratory infections	Female	0-6 days	1-4 years	1	Outdoor Air Pollution (PM2.5)
Lower respiratory infections	Female	0-6 days	1-4 years	-1	PCV3 Coverage (proportion)
Lower respiratory infections	Female	0-6 days	1-4 years	1	Vitamin A Deficiency Prevalence (age-standardized)
Lower respiratory infections	Female	0-6 days	1-4 years	-1	Maternal education (years per capita)
Lower respiratory infections	Female	0-6 days	1-4 years	1	Log-transformed SEV scalar: LRI
Lower respiratory infections	Female	0-6 days	1-4 years	1	SEV unsafe sanitation
Lower respiratory infections	Female	0-6 days	1-4 years	-1	Socio-demographic Index
Lower respiratory infections	Female	0-6 days	1-4 years	1	Stunting (proportion <2SD height for age, <5 years)
Lower respiratory infections	Female	0-6 days	1-4 years	1	Wasting (proportion <2SD weight for height, <5 years)
Lower respiratory infections	Female	0-6 days	1-4 years	-1	Healthcare access and quality index
Lower respiratory infections	Female	0-6 days	1-4 years	1	No handwashing with soap
Lower respiratory infections	Female	0-6 days	1-4 years	1	Suboptimal breastfeeding SEV
Lower respiratory infections	Female	0-6 days	1-4 years	1	Zinc deficiency
Lower respiratory infections	Female	0-6 days	1-4 years	1	Secondhand smoke
Lower respiratory infections	Male	5-9 years	95+ years	-1	SEV unsafe sanitation
Lower respiratory infections	Female	5-9 years	95+ years	-1	SEV unsafe sanitation
Otitis media	Male	0-6 days	95+ years	-1	Education (years per capita)
Otitis media	Male	0-6 days	95+ years	-1	LDI (I$ per capita)
Otitis media	Male	0-6 days	95+ years	1	Indoor Air Pollution (All Cooking Fuels)
Otitis media	Male	0-6 days	95+ years	1	Outdoor Air Pollution (PM2.5)
Otitis media	Male	0-6 days	95+ years	1	Smoking Prevalence
Otitis media	Male	0-6 days	95+ years	1	Log-transformed SEV scalar: Otitis
Otitis media	Male	0-6 days	95+ years	-1	Socio-demographic Index
Otitis media	Male	0-6 days	95+ years	-1	Healthcare access and quality index
Otitis media	Female	0-6 days	95+ years	-1	Education (years per capita)
Otitis media	Female	0-6 days	95+ years	-1	LDI (I$ per capita)
Otitis media	Female	0-6 days	95+ years	1	Indoor Air Pollution (All Cooking Fuels)
Otitis media	Female	0-6 days	95+ years	1	Outdoor Air Pollution (PM2.5)
Otitis media	Female	0-6 days	95+ years	1	Smoking Prevalence
Otitis media	Female	0-6 days	95+ years	1	Log-transformed SEV scalar: Otitis
Otitis media	Female	0-6 days	95+ years	-1	Socio-demographic Index
Otitis media	Female	0-6 days	95+ years	-1	Healthcare access and quality index
Meningitis	Female	0-6 days	1-4 years	-1	DTP3 Coverage (proportion)
Meningitis	Female	0-6 days	1-4 years	-1	LDI (I$ per capita)
Meningitis	Female	0-6 days	1-4 years	1	Underweight (proportion <2SD weight for age, <5 years)
Meningitis	Female	0-6 days	1-4 years	-1	Sanitation (proportion with access)
Meningitis	Female	0-6 days	1-4 years	-1	Improved Water Source (proportion with access)
Meningitis	Female	0-6 days	1-4 years	-1	Health System Access (capped)
Meningitis	Female	0-6 days	1-4 years	-1	Maternal education (years per capita)
Meningitis	Female	0-6 days	1-4 years	1	meningitis belt (proportion)
Meningitis	Female	0-6 days	1-4 years	-1	Socio-demographic Index
Meningitis	Female	0-6 days	1-4 years	-1	Proportion of total population covered by menafrivac initiative (meningitis meningococcal type A vaccine)
Meningitis	Female	0-6 days	1-4 years	-1	Healthcare access and quality index
Meningitis	Male	0-6 days	1-4 years	-1	DTP3 Coverage (proportion)
Meningitis	Male	0-6 days	1-4 years	-1	LDI (I$ per capita)
Meningitis	Male	0-6 days	1-4 years	1	Underweight (proportion <2SD weight for age, <5 years)
Meningitis	Male	0-6 days	1-4 years	-1	Sanitation (proportion with access)

Cause	Sex	Age start	Age end	Direction	Covariate
Meningitis	Male	0-6 days	1-4 years	-1	Improved Water Source (proportion with access)
Meningitis	Male	0-6 days	1-4 years	-1	Health System Access (capped)
Meningitis	Male	0-6 days	1-4 years	-1	Maternal education (years per capita)
Meningitis	Male	0-6 days	1-4 years	1	meningitis belt (proportion)
Meningitis	Male	0-6 days	1-4 years	-1	Socio-demographic Index
Meningitis	Male	0-6 days	1-4 years	-1	Proportion of total population covered by menafrivac initiative (meningitis meningococcal type A vaccine)
Meningitis	Male	0-6 days	1-4 years	-1	Healthcare access and quality index
Meningitis	Male	5-9 years	95+ years	-1	DTP3 Coverage (proportion)
Meningitis	Male	5-9 years	95+ years	-1	LDI (I$ per capita)
Meningitis	Male	5-9 years	95+ years	1	Underweight (proportion <2SD weight for age, <5 years)
Meningitis	Male	5-9 years	95+ years	-1	Sanitation (proportion with access)
Meningitis	Male	5-9 years	95+ years	-1	Improved Water Source (proportion with access)
Meningitis	Male	5-9 years	95+ years	-1	Health System Access (capped)
Meningitis	Male	5-9 years	95+ years	-1	Maternal education (years per capita)
Meningitis	Male	5-9 years	95+ years	1	meningitis belt (proportion)
Meningitis	Male	5-9 years	95+ years	-1	Socio-demographic Index
Meningitis	Male	5-9 years	95+ years	-1	Proportion of total population covered by menafrivac initiative (meningitis meningococcal type A vaccine)
Meningitis	Male	5-9 years	95+ years	-1	Healthcare access and quality index
Meningitis	Female	5-9 years	95+ years	-1	DTP3 Coverage (proportion)
Meningitis	Female	5-9 years	95+ years	-1	LDI (I$ per capita)
Meningitis	Female	5-9 years	95+ years	1	Underweight (proportion <2SD weight for age, <5 years)
Meningitis	Female	5-9 years	95+ years	-1	Sanitation (proportion with access)
Meningitis	Female	5-9 years	95+ years	-1	Improved Water Source (proportion with access)
Meningitis	Female	5-9 years	95+ years	-1	Health System Access (capped)
Meningitis	Female	5-9 years	95+ years	-1	Maternal education (years per capita)
Meningitis	Female	5-9 years	95+ years	1	meningitis belt (proportion)
Meningitis	Female	5-9 years	95+ years	-1	Socio-demographic Index
Meningitis	Female	5-9 years	95+ years	-1	Proportion of total population covered by menafrivac initiative (meningitis meningococcal type A vaccine)
Meningitis	Female	5-9 years	95+ years	-1	Healthcare access and quality index
Encephalitis	Male	0-6 days	95+ years	-1	In-Facility Delivery (proportion)
Encephalitis	Male	0-6 days	95+ years	-1	LDI (I$ per capita)
Encephalitis	Male	0-6 days	95+ years	1	Underweight (proportion <2SD weight for age, <5 years)
Encephalitis	Male	0-6 days	95+ years	-1	Sanitation (proportion with access)
Encephalitis	Male	0-6 days	95+ years	-1	Improved Water Source (proportion with access)
Encephalitis	Male	0-6 days	95+ years	-1	Health System Access (capped)
Encephalitis	Male	0-6 days	95+ years	-1	Maternal education (years per capita)
Encephalitis	Male	0-6 days	95+ years	-1	Socio-demographic Index
Encephalitis	Male	0-6 days	95+ years	1	Japanese encelphalitis endemic area (binary)
Encephalitis	Male	0-6 days	95+ years	-1	Healthcare access and quality index
Encephalitis	Female	0-6 days	95+ years	-1	In-Facility Delivery (proportion)
Encephalitis	Female	0-6 days	95+ years	-1	LDI (I$ per capita)
Encephalitis	Female	0-6 days	95+ years	1	Underweight (proportion <2SD weight for age, <5 years)
Encephalitis	Female	0-6 days	95+ years	-1	Sanitation (proportion with access)
Encephalitis	Female	0-6 days	95+ years	-1	Improved Water Source (proportion with access)
Encephalitis	Female	0-6 days	95+ years	-1	Health System Access (capped)
Encephalitis	Female	0-6 days	95+ years	-1	Maternal education (years per capita)
Encephalitis	Female	0-6 days	95+ years	-1	Socio-demographic Index
Encephalitis	Female	0-6 days	95+ years	1	Japanese encelphalitis endemic area (binary)
Encephalitis	Female	0-6 days	95+ years	-1	Healthcare access and quality index
Tetanus	Male	0-6 days	28-364 days	-1	DTP3 Coverage (proportion)
Tetanus	Male	0-6 days	28-364 days	-1	Education (years per capita)
Tetanus	Male	0-6 days	28-364 days	-1	In-Facility Delivery (proportion)
Tetanus	Male	0-6 days	28-364 days	-1	LDI (I$ per capita)
Tetanus	Male	0-6 days	28-364 days	-1	Skilled Birth Attendance (proportion)
Tetanus	Male	0-6 days	28-364 days	-1	Tetanus Toxoid Coverage Smooth (proportion)
Tetanus	Male	0-6 days	28-364 days	-1	Health System Access (capped)
Tetanus	Male	0-6 days	28-364 days	-1	Socio-demographic Index
Tetanus	Male	0-6 days	28-364 days	-1	Healthcare access and quality index
Tetanus	Male	1-4 years	95+ years	-1	DTP3 Coverage (proportion)
Tetanus	Male	1-4 years	95+ years	-1	Education (years per capita)
Tetanus	Male	1-4 years	95+ years	-1	LDI (I$ per capita)
Tetanus	Male	1-4 years	95+ years	-1	Sanitation (proportion with access)
Tetanus	Male	1-4 years	95+ years	-1	Health System Access (capped)
Tetanus	Male	1-4 years	95+ years	-1	Socio-demographic Index
Tetanus	Male	1-4 years	95+ years	-1	Healthcare access and quality index
Tetanus	Female	0-6 days	28-364 days	-1	DTP3 Coverage (proportion)
Tetanus	Female	0-6 days	28-364 days	-1	Education (years per capita)
Tetanus	Female	0-6 days	28-364 days	-1	In-Facility Delivery (proportion)
Tetanus	Female	0-6 days	28-364 days	-1	LDI (I$ per capita)
Tetanus	Female	0-6 days	28-364 days	-1	Skilled Birth Attendance (proportion)
Tetanus	Female	0-6 days	28-364 days	-1	Tetanus Toxoid Coverage Smooth (proportion)
Tetanus	Female	0-6 days	28-364 days	-1	Health System Access (capped)
Tetanus	Female	0-6 days	28-364 days	-1	Socio-demographic Index
Tetanus	Female	0-6 days	28-364 days	-1	Healthcare access and quality index
Tetanus	Female	1-4 years	95+ years	-1	DTP3 Coverage (proportion)
Tetanus	Female	1-4 years	95+ years	-1	Education (years per capita)
Tetanus	Female	1-4 years	95+ years	-1	LDI (I$ per capita)
Tetanus	Female	1-4 years	95+ years	-1	Sanitation (proportion with access)
Tetanus	Female	1-4 years	95+ years	-1	Health System Access (capped)
Tetanus	Female	1-4 years	95+ years	-1	Socio-demographic Index
Tetanus	Female	1-4 years	95+ years	-1	Healthcare access and quality index
Dengue	Male	28-364 days	95+ years	0	Education (years per capita)
Dengue	Male	28-364 days	95+ years	0	Health System Access (unitless)
Dengue	Male	28-364 days	95+ years	0	LDI (I$ per capita)
Dengue	Male	28-364 days	95+ years	1	Latitude Under 15 (proportion)
Dengue	Male	28-364 days	95+ years	1	Population Density (over 1000 ppl/sqkm, proportion)
Dengue	Male	28-364 days	95+ years	1	Elevation Under 100m (proportion)
Dengue	Male	28-364 days	95+ years	1	Rainfall Quintile 4 (proportion)
Dengue	Male	28-364 days	95+ years	1	Rainfall Quintile 5 (proportion)
Dengue	Male	28-364 days	95+ years	1	Population weighted probability of dengue transmission
Dengue	Male	28-364 days	95+ years	1	Dengue outbreaks (binary)
Dengue	Male	28-364 days	95+ years	1	Dengue anomalies (deviation from mean dengue incidence rate)
Dengue	Male	28-364 days	95+ years	0	Socio-demographic Index
Dengue	Female	28-364 days	95+ years	0	Education (years per capita)
Dengue	Female	28-364 days	95+ years	0	Health System Access (unitless)

Cause	Sex	Age start	Age end	Direction	Covariate
Dengue	Female	28-364 days	95+ years	0	LDI (I$ per capita)
Dengue	Female	28-364 days	95+ years	1	Latitude Under 15 (proportion)
Dengue	Female	28-364 days	95+ years	1	Population Density (over 1000 ppl/sqkm, proportion)
Dengue	Female	28-364 days	95+ years	1	Elevation Under 100m (proportion)
Dengue	Female	28-364 days	95+ years	1	Rainfall Quintile 4 (proportion)
Dengue	Female	28-364 days	95+ years	1	Rainfall Quintile 5 (proportion)
Dengue	Female	28-364 days	95+ years	1	Population weighted probability of dengue transmission
Dengue	Female	28-364 days	95+ years	1	Dengue outbreaks (binary)
Dengue	Female	28-364 days	95+ years	1	Dengue anomalies (deviation from mean dengue incidence rate)
Dengue	Female	28-364 days	95+ years	0	Socio-demographic Index
Dengue	Male	28-364 days	95+ years	1	Healthcare access and quality index
Dengue	Female	28-364 days	95+ years	1	Healthcare access and quality index
Rabies	Male	28-364 days	95+ years	-1	Antenatal Care (4 visits) Coverage (proportion)
Rabies	Male	28-364 days	95+ years	-1	Health System Access (unitless)
Rabies	Male	28-364 days	95+ years	-1	In-Facility Delivery (proportion)
Rabies	Male	28-364 days	95+ years	0	Population Density (500-1000 ppl/sqkm, proportion)
Rabies	Male	28-364 days	95+ years	0	Population Density (under 150 ppl/sqkm, proportion)
Rabies	Male	28-364 days	95+ years	-1	Skilled Birth Attendance (proportion)
Rabies	Male	28-364 days	95+ years	-1	Socio-demographic Index
Rabies	Male	28-364 days	95+ years	-1	Health System Access (capped)
Rabies	Male	28-364 days	95+ years	-1	Healthcare access and quality index
Rabies	Female	28-364 days	95+ years	-1	Antenatal Care (4 visits) Coverage (proportion)
Rabies	Female	28-364 days	95+ years	-1	Health System Access (unitless)
Rabies	Female	28-364 days	95+ years	-1	In-Facility Delivery (proportion)
Rabies	Female	28-364 days	95+ years	0	Population Density (500-1000 ppl/sqkm, proportion)
Rabies	Female	28-364 days	95+ years	0	Population Density (under 150 ppl/sqkm, proportion)
Rabies	Female	28-364 days	95+ years	-1	Skilled Birth Attendance (proportion)
Rabies	Female	28-364 days	95+ years	-1	Socio-demographic Index
Rabies	Female	28-364 days	95+ years	-1	Health System Access (capped)
Rabies	Female	28-364 days	95+ years	-1	Healthcare access and quality index
Rabies	Male	28-364 days	95+ years	1	Healthcare access and quality index
Rabies	Female	28-364 days	95+ years	1	Healthcare access and quality index
Other neglected tropical diseases	Male	0-6 days	95+ years	-1	Education (years per capita)
Other neglected tropical diseases	Male	0-6 days	95+ years	-1	LDI (I$ per capita)
Other neglected tropical diseases	Male	0-6 days	95+ years	1	Latitude Under 15 (proportion)
Other neglected tropical diseases	Male	0-6 days	95+ years	1	Rainfall Quintile 5 (proportion)
Other neglected tropical diseases	Male	0-6 days	95+ years	-1	Sanitation (proportion with access)
Other neglected tropical diseases	Male	0-6 days	95+ years	-1	Socio-demographic Index
Other neglected tropical diseases	Male	0-6 days	95+ years	-1	Healthcare access and quality index
Other neglected tropical diseases	Female	0-6 days	95+ years	-1	Education (years per capita)
Other neglected tropical diseases	Female	0-6 days	95+ years	-1	LDI (I$ per capita)
Other neglected tropical diseases	Female	0-6 days	95+ years	1	Latitude Under 15 (proportion)
Other neglected tropical diseases	Female	0-6 days	95+ years	1	Rainfall Quintile 5 (proportion)
Other neglected tropical diseases	Female	0-6 days	95+ years	-1	Sanitation (proportion with access)
Other neglected tropical diseases	Female	0-6 days	95+ years	-1	Socio-demographic Index
Other neglected tropical diseases	Female	0-6 days	95+ years	-1	Healthcare access and quality index
Neonatal disorders	Male	0-6 days	1-4 years	-1	Antenatal Care (4 visits) Coverage (proportion)
Neonatal disorders	Male	0-6 days	1-4 years	-1	Education (years per capita)
Neonatal disorders	Male	0-6 days	1-4 years	-1	In-Facility Delivery (proportion)
Neonatal disorders	Male	0-6 days	1-4 years	-1	LDI (I$ per capita)
Neonatal disorders	Male	0-6 days	1-4 years	1	Underweight (proportion <2SD weight for age, <5 years)
Neonatal disorders	Male	0-6 days	1-4 years	1	Live Births 35+ (proportion)
Neonatal disorders	Male	0-6 days	1-4 years	1	Indoor Air Pollution (All Cooking Fuels)
Neonatal disorders	Male	0-6 days	1-4 years	-1	Skilled Birth Attendance (proportion)
Neonatal disorders	Male	0-6 days	1-4 years	1	Smoking Prevalence (Reproductive Age Standardized)
Neonatal disorders	Male	0-6 days	1-4 years	1	Total Fertility Rate
Neonatal disorders	Male	0-6 days	1-4 years	-1	Health System Access (capped)
Neonatal disorders	Male	0-6 days	1-4 years	-1	Socio-demographic Index
Neonatal disorders	Male	0-6 days	1-4 years	-1	Healthcare access and quality index
Neonatal disorders	Female	0-6 days	1-4 years	-1	Antenatal Care (4 visits) Coverage (proportion)
Neonatal disorders	Female	0-6 days	1-4 years	-1	Education (years per capita)
Neonatal disorders	Female	0-6 days	1-4 years	-1	In-Facility Delivery (proportion)
Neonatal disorders	Female	0-6 days	1-4 years	-1	LDI (I$ per capita)
Neonatal disorders	Female	0-6 days	1-4 years	1	Underweight (proportion <2SD weight for age, <5 years)
Neonatal disorders	Female	0-6 days	1-4 years	1	Live Births 35+ (proportion)
Neonatal disorders	Female	0-6 days	1-4 years	1	Indoor Air Pollution (All Cooking Fuels)
Neonatal disorders	Female	0-6 days	1-4 years	-1	Skilled Birth Attendance (proportion)
Neonatal disorders	Female	0-6 days	1-4 years	1	Smoking Prevalence (Reproductive Age Standardized)
Neonatal disorders	Female	0-6 days	1-4 years	1	Total Fertility Rate
Neonatal disorders	Female	0-6 days	1-4 years	-1	Health System Access (capped)
Neonatal disorders	Female	0-6 days	1-4 years	-1	Socio-demographic Index
Neonatal disorders	Female	0-6 days	1-4 years	-1	Healthcare access and quality index
Neonatal preterm birth complications	Male	0-6 days	1-4 years	-1	Antenatal Care (4 visits) Coverage (proportion)
Neonatal preterm birth complications	Male	0-6 days	1-4 years	-1	Education (years per capita)
Neonatal preterm birth complications	Male	0-6 days	1-4 years	-1	In-Facility Delivery (proportion)
Neonatal preterm birth complications	Male	0-6 days	1-4 years	-1	LDI (I$ per capita)
Neonatal preterm birth complications	Male	0-6 days	1-4 years	1	Underweight (proportion <2SD weight for age, <5 years)
Neonatal preterm birth complications	Male	0-6 days	1-4 years	1	Live Births 35+ (proportion)
Neonatal preterm birth complications	Male	0-6 days	1-4 years	1	Indoor Air Pollution (All Cooking Fuels)
Neonatal preterm birth complications	Male	0-6 days	1-4 years	-1	Skilled Birth Attendance (proportion)
Neonatal preterm birth complications	Male	0-6 days	1-4 years	1	Smoking Prevalence (Reproductive Age Standardized)
Neonatal preterm birth complications	Male	0-6 days	1-4 years	1	Total Fertility Rate
Neonatal preterm birth complications	Male	0-6 days	1-4 years	-1	Health System Access (capped)
Neonatal preterm birth complications	Male	0-6 days	1-4 years	-1	Socio-demographic Index
Neonatal preterm birth complications	Male	0-6 days	1-4 years	-1	Healthcare access and quality index
Neonatal preterm birth complications	Female	0-6 days	1-4 years	-1	Antenatal Care (4 visits) Coverage (proportion)
Neonatal preterm birth complications	Female	0-6 days	1-4 years	-1	Education (years per capita)
Neonatal preterm birth complications	Female	0-6 days	1-4 years	-1	In-Facility Delivery (proportion)
Neonatal preterm birth complications	Female	0-6 days	1-4 years	-1	LDI (I$ per capita)
Neonatal preterm birth complications	Female	0-6 days	1-4 years	1	Underweight (proportion <2SD weight for age, <5 years)
Neonatal preterm birth complications	Female	0-6 days	1-4 years	1	Live Births 35+ (proportion)
Neonatal preterm birth complications	Female	0-6 days	1-4 years	1	Indoor Air Pollution (All Cooking Fuels)
Neonatal preterm birth complications	Female	0-6 days	1-4 years	-1	Skilled Birth Attendance (proportion)
Neonatal preterm birth complications	Female	0-6 days	1-4 years	1	Smoking Prevalence (Reproductive Age Standardized)
Neonatal preterm birth complications	Female	0-6 days	1-4 years	1	Total Fertility Rate

Cause	Sex	Age start	Age end	Direction	Covariate
Neonatal preterm birth complications	Female	0-6 days	1-4 years	-1	Health System Access (capped)
Neonatal preterm birth complications	Female	0-6 days	1-4 years	-1	Socio-demographic Index
Neonatal preterm birth complications	Female	0-6 days	1-4 years	-1	Healthcare access and quality index
Neonatal encephalopathy due to birth asphyxia and trauma	Male	0-6 days	1-4 years	-1	Antenatal Care (4 visits) Coverage (proportion)
Neonatal encephalopathy due to birth asphyxia and trauma	Male	0-6 days	1-4 years	-1	Education (years per capita)
Neonatal encephalopathy due to birth asphyxia and trauma	Male	0-6 days	1-4 years	-1	In-Facility Delivery (proportion)
Neonatal encephalopathy due to birth asphyxia and trauma	Male	0-6 days	1-4 years	-1	LDI (I$ per capita)
Neonatal encephalopathy due to birth asphyxia and trauma	Male	0-6 days	1-4 years	1	Underweight (proportion <2SD weight for age, <5 years)
Neonatal encephalopathy due to birth asphyxia and trauma	Male	0-6 days	1-4 years	1	Live Births 35+ (proportion)
Neonatal encephalopathy due to birth asphyxia and trauma	Male	0-6 days	1-4 years	1	Indoor Air Pollution (All Cooking Fuels)
Neonatal encephalopathy due to birth asphyxia and trauma	Male	0-6 days	1-4 years	-1	Skilled Birth Attendance (proportion)
Neonatal encephalopathy due to birth asphyxia and trauma	Male	0-6 days	1-4 years	1	Smoking Prevalence (Reproductive Age Standardized)
Neonatal encephalopathy due to birth asphyxia and trauma	Male	0-6 days	1-4 years	1	Total Fertility Rate
Neonatal encephalopathy due to birth asphyxia and trauma	Male	0-6 days	1-4 years	-1	Health System Access (capped)
Neonatal encephalopathy due to birth asphyxia and trauma	Male	0-6 days	1-4 years	-1	Socio-demographic Index
Neonatal encephalopathy due to birth asphyxia and trauma	Male	0-6 days	1-4 years	-1	Healthcare access and quality index
Neonatal encephalopathy due to birth asphyxia and trauma	Female	0-6 days	1-4 years	-1	Antenatal Care (4 visits) Coverage (proportion)
Neonatal encephalopathy due to birth asphyxia and trauma	Female	0-6 days	1-4 years	-1	Education (years per capita)
Neonatal encephalopathy due to birth asphyxia and trauma	Female	0-6 days	1-4 years	-1	In-Facility Delivery (proportion)
Neonatal encephalopathy due to birth asphyxia and trauma	Female	0-6 days	1-4 years	-1	LDI (I$ per capita)
Neonatal encephalopathy due to birth asphyxia and trauma	Female	0-6 days	1-4 years	1	Underweight (proportion <2SD weight for age, <5 years)
Neonatal encephalopathy due to birth asphyxia and trauma	Female	0-6 days	1-4 years	1	Live Births 35+ (proportion)
Neonatal encephalopathy due to birth asphyxia and trauma	Female	0-6 days	1-4 years	1	Indoor Air Pollution (All Cooking Fuels)
Neonatal encephalopathy due to birth asphyxia and trauma	Female	0-6 days	1-4 years	-1	Skilled Birth Attendance (proportion)
Neonatal encephalopathy due to birth asphyxia and trauma	Female	0-6 days	1-4 years	1	Smoking Prevalence (Reproductive Age Standardized)
Neonatal encephalopathy due to birth asphyxia and trauma	Female	0-6 days	1-4 years	1	Total Fertility Rate
Neonatal encephalopathy due to birth asphyxia and trauma	Female	0-6 days	1-4 years	-1	Health System Access (capped)
Neonatal encephalopathy due to birth asphyxia and trauma	Female	0-6 days	1-4 years	-1	Socio-demographic Index
Neonatal encephalopathy due to birth asphyxia and trauma	Female	0-6 days	1-4 years	-1	Healthcare access and quality index
Neonatal sepsis and other neonatal infections	Male	0-6 days	1-4 years	-1	Antenatal Care (4 visits) Coverage (proportion)
Neonatal sepsis and other neonatal infections	Male	0-6 days	1-4 years	-1	Education (years per capita)
Neonatal sepsis and other neonatal infections	Male	0-6 days	1-4 years	-1	In-Facility Delivery (proportion)
Neonatal sepsis and other neonatal infections	Male	0-6 days	1-4 years	-1	LDI (I$ per capita)
Neonatal sepsis and other neonatal infections	Male	0-6 days	1-4 years	1	Underweight (proportion <2SD weight for age, <5 years)
Neonatal sepsis and other neonatal infections	Male	0-6 days	1-4 years	1	Live Births 35+ (proportion)
Neonatal sepsis and other neonatal infections	Male	0-6 days	1-4 years	1	Indoor Air Pollution (All Cooking Fuels)
Neonatal sepsis and other neonatal infections	Male	0-6 days	1-4 years	-1	Skilled Birth Attendance (proportion)
Neonatal sepsis and other neonatal infections	Male	0-6 days	1-4 years	1	Smoking Prevalence (Reproductive Age Standardized)
Neonatal sepsis and other neonatal infections	Male	0-6 days	1-4 years	1	Total Fertility Rate
Neonatal sepsis and other neonatal infections	Male	0-6 days	1-4 years	-1	Health System Access (capped)
Neonatal sepsis and other neonatal infections	Male	0-6 days	1-4 years	-1	Socio-demographic Index
Neonatal sepsis and other neonatal infections	Male	0-6 days	1-4 years	-1	Healthcare access and quality index
Neonatal sepsis and other neonatal infections	Female	0-6 days	1-4 years	-1	Antenatal Care (4 visits) Coverage (proportion)
Neonatal sepsis and other neonatal infections	Female	0-6 days	1-4 years	-1	Education (years per capita)
Neonatal sepsis and other neonatal infections	Female	0-6 days	1-4 years	-1	In-Facility Delivery (proportion)
Neonatal sepsis and other neonatal infections	Female	0-6 days	1-4 years	-1	LDI (I$ per capita)
Neonatal sepsis and other neonatal infections	Female	0-6 days	1-4 years	1	Underweight (proportion <2SD weight for age, <5 years)
Neonatal sepsis and other neonatal infections	Female	0-6 days	1-4 years	1	Live Births 35+ (proportion)
Neonatal sepsis and other neonatal infections	Female	0-6 days	1-4 years	1	Indoor Air Pollution (All Cooking Fuels)
Neonatal sepsis and other neonatal infections	Female	0-6 days	1-4 years	-1	Skilled Birth Attendance (proportion)
Neonatal sepsis and other neonatal infections	Female	0-6 days	1-4 years	1	Smoking Prevalence (Reproductive Age Standardized)
Neonatal sepsis and other neonatal infections	Female	0-6 days	1-4 years	1	Total Fertility Rate
Neonatal sepsis and other neonatal infections	Female	0-6 days	1-4 years	-1	Health System Access (capped)
Neonatal sepsis and other neonatal infections	Female	0-6 days	1-4 years	-1	Socio-demographic Index
Neonatal sepsis and other neonatal infections	Female	0-6 days	1-4 years	-1	Healthcare access and quality index
Hemolytic disease and other neonatal jaundice	Male	0-6 days	1-4 years	-1	Antenatal Care (4 visits) Coverage (proportion)
Hemolytic disease and other neonatal jaundice	Male	0-6 days	1-4 years	-1	Education (years per capita)
Hemolytic disease and other neonatal jaundice	Male	0-6 days	1-4 years	-1	In-Facility Delivery (proportion)
Hemolytic disease and other neonatal jaundice	Male	0-6 days	1-4 years	-1	LDI (I$ per capita)
Hemolytic disease and other neonatal jaundice	Male	0-6 days	1-4 years	1	Underweight (proportion <2SD weight for age, <5 years)
Hemolytic disease and other neonatal jaundice	Male	0-6 days	1-4 years	1	Live Births 35+ (proportion)
Hemolytic disease and other neonatal jaundice	Male	0-6 days	1-4 years	1	Indoor Air Pollution (All Cooking Fuels)
Hemolytic disease and other neonatal jaundice	Male	0-6 days	1-4 years	-1	Skilled Birth Attendance (proportion)
Hemolytic disease and other neonatal jaundice	Male	0-6 days	1-4 years	1	Smoking Prevalence (Reproductive Age Standardized)
Hemolytic disease and other neonatal jaundice	Male	0-6 days	1-4 years	1	Total Fertility Rate
Hemolytic disease and other neonatal jaundice	Male	0-6 days	1-4 years	-1	Health System Access (capped)
Hemolytic disease and other neonatal jaundice	Male	0-6 days	1-4 years	-1	Socio-demographic Index
Hemolytic disease and other neonatal jaundice	Male	0-6 days	1-4 years	-1	Healthcare access and quality index
Hemolytic disease and other neonatal jaundice	Female	0-6 days	1-4 years	-1	Antenatal Care (4 visits) Coverage (proportion)
Hemolytic disease and other neonatal jaundice	Female	0-6 days	1-4 years	-1	Education (years per capita)
Hemolytic disease and other neonatal jaundice	Female	0-6 days	1-4 years	-1	In-Facility Delivery (proportion)
Hemolytic disease and other neonatal jaundice	Female	0-6 days	1-4 years	-1	LDI (I$ per capita)
Hemolytic disease and other neonatal jaundice	Female	0-6 days	1-4 years	1	Underweight (proportion <2SD weight for age, <5 years)
Hemolytic disease and other neonatal jaundice	Female	0-6 days	1-4 years	1	Live Births 35+ (proportion)
Hemolytic disease and other neonatal jaundice	Female	0-6 days	1-4 years	1	Indoor Air Pollution (All Cooking Fuels)
Hemolytic disease and other neonatal jaundice	Female	0-6 days	1-4 years	-1	Skilled Birth Attendance (proportion)
Hemolytic disease and other neonatal jaundice	Female	0-6 days	1-4 years	1	Smoking Prevalence (Reproductive Age Standardized)
Hemolytic disease and other neonatal jaundice	Female	0-6 days	1-4 years	1	Total Fertility Rate
Hemolytic disease and other neonatal jaundice	Female	0-6 days	1-4 years	-1	Health System Access (capped)
Hemolytic disease and other neonatal jaundice	Female	0-6 days	1-4 years	-1	Socio-demographic Index
Hemolytic disease and other neonatal jaundice	Female	0-6 days	1-4 years	-1	Healthcare access and quality index
Other neonatal disorders	Male	0-6 days	1-4 years	-1	Antenatal Care (4 visits) Coverage (proportion)
Other neonatal disorders	Male	0-6 days	1-4 years	-1	Education (years per capita)
Other neonatal disorders	Male	0-6 days	1-4 years	-1	In-Facility Delivery (proportion)
Other neonatal disorders	Male	0-6 days	1-4 years	-1	LDI (I$ per capita)
Other neonatal disorders	Male	0-6 days	1-4 years	1	Underweight (proportion <2SD weight for age, <5 years)
Other neonatal disorders	Male	0-6 days	1-4 years	1	Live Births 35+ (proportion)
Other neonatal disorders	Male	0-6 days	1-4 years	1	Indoor Air Pollution (All Cooking Fuels)
Other neonatal disorders	Male	0-6 days	1-4 years	-1	Skilled Birth Attendance (proportion)
Other neonatal disorders	Male	0-6 days	1-4 years	1	Smoking Prevalence (Reproductive Age Standardized)
Other neonatal disorders	Male	0-6 days	1-4 years	1	Total Fertility Rate
Other neonatal disorders	Male	0-6 days	1-4 years	-1	Health System Access (capped)
Other neonatal disorders	Male	0-6 days	1-4 years	-1	Socio-demographic Index
Other neonatal disorders	Male	0-6 days	1-4 years	-1	Healthcare access and quality index
Other neonatal disorders	Female	0-6 days	1-4 years	-1	Antenatal Care (4 visits) Coverage (proportion)

Cause	Sex	Age start	Age end	Direction	Covariate
Other neonatal disorders	Female	0-6 days	1-4 years	-1	Education (years per capita)
Other neonatal disorders	Female	0-6 days	1-4 years	-1	In-Facility Delivery (proportion)
Other neonatal disorders	Female	0-6 days	1-4 years	-1	LDI (I$ per capita)
Other neonatal disorders	Female	0-6 days	1-4 years	1	Underweight (proportion <2SD weight for age, <5 years)
Other neonatal disorders	Female	0-6 days	1-4 years	1	Live Births 35+ (proportion)
Other neonatal disorders	Female	0-6 days	1-4 years	1	Indoor Air Pollution (All Cooking Fuels)
Other neonatal disorders	Female	0-6 days	1-4 years	-1	Skilled Birth Attendance (proportion)
Other neonatal disorders	Female	0-6 days	1-4 years	1	Smoking Prevalence (Reproductive Age Standardized)
Other neonatal disorders	Female	0-6 days	1-4 years	1	Total Fertility Rate
Other neonatal disorders	Female	0-6 days	1-4 years	-1	Health System Access (capped)
Other neonatal disorders	Female	0-6 days	1-4 years	-1	Socio-demographic Index
Other neonatal disorders	Female	0-6 days	1-4 years	-1	Healthcare access and quality index
Nutritional deficiencies	Male	28-364 days	95+ years	-1	Antenatal Care (4 visits) Coverage (proportion)
Nutritional deficiencies	Male	28-364 days	95+ years	-1	Education (years per capita)
Nutritional deficiencies	Male	28-364 days	95+ years	-1	Proportion of households using iodized salt (adjusted)
Nutritional deficiencies	Male	28-364 days	95+ years	-1	LDI (I$ per capita)
Nutritional deficiencies	Male	28-364 days	95+ years	1	Underweight (proportion <2SD weight for age, <5 years)
Nutritional deficiencies	Male	28-364 days	95+ years	0	Rainfall Quintile 1 (proportion)
Nutritional deficiencies	Male	28-364 days	95+ years	0	Rainfall Quintile 2 (proportion)
Nutritional deficiencies	Male	28-364 days	95+ years	-1	Sanitation (proportion with access)
Nutritional deficiencies	Male	28-364 days	95+ years	1	Mortality Rate Due to War Shocks (per 1 person)
Nutritional deficiencies	Male	28-364 days	95+ years	-1	Improved Water Source (proportion with access)
Nutritional deficiencies	Male	28-364 days	95+ years	1	Age-Standardize Prevalence of Severe Anemia
Nutritional deficiencies	Male	28-364 days	95+ years	-1	Health System Access (capped)
Nutritional deficiencies	Male	28-364 days	95+ years	-1	Socio-demographic Index
Nutritional deficiencies	Male	28-364 days	95+ years	-1	energy unadjusted(kcal)
Nutritional deficiencies	Male	28-364 days	95+ years	1	Wasting (proportion <2SD weight for height, <5 years)
Nutritional deficiencies	Male	28-364 days	95+ years	-1	Healthcare access and quality index
Nutritional deficiencies	Male	28-364 days	95+ years	1	Malnutrition Shock mortality rate
Nutritional deficiencies	Female	28-364 days	95+ years	-1	Antenatal Care (4 visits) Coverage (proportion)
Nutritional deficiencies	Female	28-364 days	95+ years	-1	Education (years per capita)
Nutritional deficiencies	Female	28-364 days	95+ years	-1	Proportion of households using iodized salt (adjusted)
Nutritional deficiencies	Female	28-364 days	95+ years	-1	LDI (I$ per capita)
Nutritional deficiencies	Female	28-364 days	95+ years	1	Underweight (proportion <2SD weight for age, <5 years)
Nutritional deficiencies	Female	28-364 days	95+ years	0	Rainfall Quintile 1 (proportion)
Nutritional deficiencies	Female	28-364 days	95+ years	0	Rainfall Quintile 2 (proportion)
Nutritional deficiencies	Female	28-364 days	95+ years	-1	Sanitation (proportion with access)
Nutritional deficiencies	Female	28-364 days	95+ years	1	Mortality Rate Due to War Shocks (per 1 person)
Nutritional deficiencies	Female	28-364 days	95+ years	-1	Improved Water Source (proportion with access)
Nutritional deficiencies	Female	28-364 days	95+ years	1	Age-Standardize Prevalence of Severe Anemia
Nutritional deficiencies	Female	28-364 days	95+ years	-1	Health System Access (capped)
Nutritional deficiencies	Female	28-364 days	95+ years	-1	Socio-demographic Index
Nutritional deficiencies	Female	28-364 days	95+ years	-1	energy unadjusted(kcal)
Nutritional deficiencies	Female	28-364 days	95+ years	1	Wasting (proportion <2SD weight for height, <5 years)
Nutritional deficiencies	Female	28-364 days	95+ years	-1	Healthcare access and quality index
Nutritional deficiencies	Female	28-364 days	95+ years	1	Malnutrition Shock mortality rate
Protein-energy malnutrition	Male	5-9 years	95+ years	-1	Antenatal Care (4 visits) Coverage (proportion)
Protein-energy malnutrition	Male	5-9 years	95+ years	-1	Education (years per capita)
Protein-energy malnutrition	Male	5-9 years	95+ years	-1	LDI (I$ per capita)
Protein-energy malnutrition	Male	5-9 years	95+ years	0	Rainfall Quintile 1 (proportion)
Protein-energy malnutrition	Male	5-9 years	95+ years	0	Rainfall Quintile 2 (proportion)
Protein-energy malnutrition	Male	5-9 years	95+ years	-1	Sanitation (proportion with access)
Protein-energy malnutrition	Male	5-9 years	95+ years	1	Mortality Rate Due to War Shocks (per 1 person)
Protein-energy malnutrition	Male	5-9 years	95+ years	-1	Improved Water Source (proportion with access)
Protein-energy malnutrition	Male	5-9 years	95+ years	1	Age-Standardize Prevalence of Severe Anemia
Protein-energy malnutrition	Male	5-9 years	95+ years	-1	Health System Access (capped)
Protein-energy malnutrition	Male	5-9 years	95+ years	-1	Socio-demographic Index
Protein-energy malnutrition	Male	5-9 years	95+ years	-1	energy unadjusted(kcal)
Protein-energy malnutrition	Male	5-9 years	95+ years	-1	Healthcare access and quality index
Protein-energy malnutrition	Male	5-9 years	95+ years	1	Malnutrition Shock mortality rate
Protein-energy malnutrition	Male	28-364 days	1-4 years	-1	Antenatal Care (4 visits) Coverage (proportion)
Protein-energy malnutrition	Male	28-364 days	1-4 years	-1	Education (years per capita)
Protein-energy malnutrition	Male	28-364 days	1-4 years	-1	LDI (I$ per capita)
Protein-energy malnutrition	Male	28-364 days	1-4 years	0	Rainfall Quintile 1 (proportion)
Protein-energy malnutrition	Male	28-364 days	1-4 years	0	Rainfall Quintile 2 (proportion)
Protein-energy malnutrition	Male	28-364 days	1-4 years	-1	Sanitation (proportion with access)
Protein-energy malnutrition	Male	28-364 days	1-4 years	1	Mortality Rate Due to War Shocks (per 1 person)
Protein-energy malnutrition	Male	28-364 days	1-4 years	-1	Improved Water Source (proportion with access)
Protein-energy malnutrition	Male	28-364 days	1-4 years	1	Age-Standardize Prevalence of Severe Anemia
Protein-energy malnutrition	Male	28-364 days	1-4 years	-1	Health System Access (capped)
Protein-energy malnutrition	Male	28-364 days	1-4 years	-1	Socio-demographic Index
Protein-energy malnutrition	Male	28-364 days	1-4 years	-1	energy unadjusted(kcal)
Protein-energy malnutrition	Male	28-364 days	1-4 years	1	Wasting (proportion <2SD weight for height, <5 years)
Protein-energy malnutrition	Male	28-364 days	1-4 years	-1	Healthcare access and quality index
Protein-energy malnutrition	Male	28-364 days	1-4 years	1	Malnutrition Shock mortality rate
Protein-energy malnutrition	Female	28-364 days	1-4 years	-1	Antenatal Care (4 visits) Coverage (proportion)
Protein-energy malnutrition	Female	28-364 days	1-4 years	-1	Education (years per capita)
Protein-energy malnutrition	Female	28-364 days	1-4 years	-1	LDI (I$ per capita)
Protein-energy malnutrition	Female	28-364 days	1-4 years	0	Rainfall Quintile 1 (proportion)
Protein-energy malnutrition	Female	28-364 days	1-4 years	0	Rainfall Quintile 2 (proportion)
Protein-energy malnutrition	Female	28-364 days	1-4 years	-1	Sanitation (proportion with access)
Protein-energy malnutrition	Female	28-364 days	1-4 years	1	Mortality Rate Due to War Shocks (per 1 person)
Protein-energy malnutrition	Female	28-364 days	1-4 years	-1	Improved Water Source (proportion with access)
Protein-energy malnutrition	Female	28-364 days	1-4 years	1	Age-Standardize Prevalence of Severe Anemia
Protein-energy malnutrition	Female	28-364 days	1-4 years	-1	Health System Access (capped)
Protein-energy malnutrition	Female	28-364 days	1-4 years	-1	Socio-demographic Index
Protein-energy malnutrition	Female	28-364 days	1-4 years	-1	energy unadjusted(kcal)
Protein-energy malnutrition	Female	28-364 days	1-4 years	1	Wasting (proportion <2SD weight for height, <5 years)
Protein-energy malnutrition	Female	28-364 days	1-4 years	-1	Healthcare access and quality index
Protein-energy malnutrition	Female	5-9 years	95+ years	-1	Antenatal Care (4 visits) Coverage (proportion)
Protein-energy malnutrition	Female	5-9 years	95+ years	-1	Education (years per capita)
Protein-energy malnutrition	Female	5-9 years	95+ years	-1	LDI (I$ per capita)
Protein-energy malnutrition	Female	5-9 years	95+ years	0	Rainfall Quintile 1 (proportion)
Protein-energy malnutrition	Female	5-9 years	95+ years	0	Rainfall Quintile 2 (proportion)
Protein-energy malnutrition	Female	5-9 years	95+ years	-1	Sanitation (proportion with access)

Cause	Sex	Age start	Age end	Direction	Covariate
Protein-energy malnutrition	Female	5-9 years	95+ years	1	Mortality Rate Due to War Shocks (per 1 person)
Protein-energy malnutrition	Female	5-9 years	95+ years	-1	Improved Water Source (proportion with access)
Protein-energy malnutrition	Female	5-9 years	95+ years	1	Age-Standardize Prevalence of Severe Anemia
Protein-energy malnutrition	Female	5-9 years	95+ years	-1	Health System Access (capped)
Protein-energy malnutrition	Female	5-9 years	95+ years	-1	Socio-demographic Index
Protein-energy malnutrition	Female	5-9 years	95+ years	-1	energy unadjusted(kcal)
Protein-energy malnutrition	Female	5-9 years	95+ years	-1	Healthcare access and quality index
Iron-deficiency anemia	Male	28-364 days	95+ years	-1	Education (years per capita)
Iron-deficiency anemia	Male	28-364 days	95+ years	-1	LDI (I$ per capita)
Iron-deficiency anemia	Male	28-364 days	95+ years	1	Underweight (proportion <2SD weight for age, <5 years)
Iron-deficiency anemia	Male	28-364 days	95+ years	0	Rainfall Quintile 1 (proportion)
Iron-deficiency anemia	Male	28-364 days	95+ years	0	Rainfall Quintile 2 (proportion)
Iron-deficiency anemia	Male	28-364 days	95+ years	-1	Sanitation (proportion with access)
Iron-deficiency anemia	Male	28-364 days	95+ years	-1	Total Calories (kcal per capita)
Iron-deficiency anemia	Male	28-364 days	95+ years	-1	Improved Water Source (proportion with access)
Iron-deficiency anemia	Male	28-364 days	95+ years	1	Age-Standardize Prevalence of Severe Anemia
Iron-deficiency anemia	Male	28-364 days	95+ years	-1	Health System Access (capped)
Iron-deficiency anemia	Male	28-364 days	95+ years	-1	Socio-demographic Index
Iron-deficiency anemia	Male	28-364 days	95+ years	-1	Healthcare access and quality index
Iron-deficiency anemia	Female	28-364 days	95+ years	-1	Education (years per capita)
Iron-deficiency anemia	Female	28-364 days	95+ years	-1	LDI (I$ per capita)
Iron-deficiency anemia	Female	28-364 days	95+ years	1	Underweight (proportion <2SD weight for age, <5 years)
Iron-deficiency anemia	Female	28-364 days	95+ years	0	Rainfall Quintile 1 (proportion)
Iron-deficiency anemia	Female	28-364 days	95+ years	0	Rainfall Quintile 2 (proportion)
Iron-deficiency anemia	Female	28-364 days	95+ years	-1	Sanitation (proportion with access)
Iron-deficiency anemia	Female	28-364 days	95+ years	-1	Total Calories (kcal per capita)
Iron-deficiency anemia	Female	28-364 days	95+ years	-1	Improved Water Source (proportion with access)
Iron-deficiency anemia	Female	28-364 days	95+ years	1	Age-Standardize Prevalence of Severe Anemia
Iron-deficiency anemia	Female	28-364 days	95+ years	-1	Health System Access (capped)
Iron-deficiency anemia	Female	28-364 days	95+ years	-1	Socio-demographic Index
Iron-deficiency anemia	Female	28-364 days	95+ years	-1	Healthcare access and quality index
Iron-deficiency anemia	Male	28-364 days	95+ years	-1	Health System Access 2 (unitless)
Iron-deficiency anemia	Female	28-364 days	95+ years	-1	Health System Access 2 (unitless)
Other nutritional deficiencies	Female	28-364 days	95+ years	-1	Education (years per capita)
Other nutritional deficiencies	Female	28-364 days	95+ years	-1	LDI (I$ per capita)
Other nutritional deficiencies	Female	28-364 days	95+ years	1	Underweight (proportion <2SD weight for age, <5 years)
Other nutritional deficiencies	Female	28-364 days	95+ years	0	Rainfall Quintile 1 (proportion)
Other nutritional deficiencies	Female	28-364 days	95+ years	0	Rainfall Quintile 2 (proportion)
Other nutritional deficiencies	Female	28-364 days	95+ years	-1	Sanitation (proportion with access)
Other nutritional deficiencies	Female	28-364 days	95+ years	1	Mortality Rate Due to War Shocks (per 1 person)
Other nutritional deficiencies	Female	28-364 days	95+ years	-1	Improved Water Source (proportion with access)
Other nutritional deficiencies	Female	28-364 days	95+ years	1	Age-Standardize Prevalence of Severe Anemia
Other nutritional deficiencies	Female	28-364 days	95+ years	-1	Health System Access (capped)
Other nutritional deficiencies	Female	28-364 days	95+ years	-1	Socio-demographic Index
Other nutritional deficiencies	Female	28-364 days	95+ years	-1	energy unadjusted(kcal)
Other nutritional deficiencies	Female	28-364 days	95+ years	-1	Healthcare access and quality index
Other nutritional deficiencies	Female	28-364 days	95+ years	1	Malnutrition Shock mortality rate
Other nutritional deficiencies	Male	28-364 days	95+ years	-1	Education (years per capita)
Other nutritional deficiencies	Male	28-364 days	95+ years	-1	LDI (I$ per capita)
Other nutritional deficiencies	Male	28-364 days	95+ years	1	Underweight (proportion <2SD weight for age, <5 years)
Other nutritional deficiencies	Male	28-364 days	95+ years	0	Rainfall Quintile 1 (proportion)
Other nutritional deficiencies	Male	28-364 days	95+ years	0	Rainfall Quintile 2 (proportion)
Other nutritional deficiencies	Male	28-364 days	95+ years	-1	Sanitation (proportion with access)
Other nutritional deficiencies	Male	28-364 days	95+ years	1	Mortality Rate Due to War Shocks (per 1 person)
Other nutritional deficiencies	Male	28-364 days	95+ years	-1	Improved Water Source (proportion with access)
Other nutritional deficiencies	Male	28-364 days	95+ years	1	Age-Standardize Prevalence of Severe Anemia
Other nutritional deficiencies	Male	28-364 days	95+ years	-1	Health System Access (capped)
Other nutritional deficiencies	Male	28-364 days	95+ years	-1	Socio-demographic Index
Other nutritional deficiencies	Male	28-364 days	95+ years	-1	energy unadjusted(kcal)
Other nutritional deficiencies	Male	28-364 days	95+ years	-1	Healthcare access and quality index
Other nutritional deficiencies	Male	28-364 days	95+ years	1	Malnutrition Shock mortality rate
Sexually transmitted diseases excluding HIV	Male	10-14 years	95+ years	-1	Legality of Abortion
Sexually transmitted diseases excluding HIV	Male	10-14 years	95+ years	-1	Antenatal Care (1 visit) Coverage (proportion)
Sexually transmitted diseases excluding HIV	Male	10-14 years	95+ years	-1	Antenatal Care (4 visits) Coverage (proportion)
Sexually transmitted diseases excluding HIV	Male	10-14 years	95+ years	1	Age-Specific Fertility Rate
Sexually transmitted diseases excluding HIV	Male	10-14 years	95+ years	-1	Education (years per capita)
Sexually transmitted diseases excluding HIV	Male	10-14 years	95+ years	-1	LDI (I$ per capita)
Sexually transmitted diseases excluding HIV	Male	10-14 years	95+ years	1	Total Fertility Rate
Sexually transmitted diseases excluding HIV	Male	10-14 years	95+ years	-1	Health System Access (capped)
Sexually transmitted diseases excluding HIV	Male	10-14 years	95+ years	1	Syphilis prevalence (proportion)
Sexually transmitted diseases excluding HIV	Male	10-14 years	95+ years	-1	Healthcare access and quality index
Sexually transmitted diseases excluding HIV	Female	10-14 years	95+ years	-1	Legality of Abortion
Sexually transmitted diseases excluding HIV	Female	10-14 years	95+ years	-1	Antenatal Care (1 visit) Coverage (proportion)
Sexually transmitted diseases excluding HIV	Female	10-14 years	95+ years	-1	Antenatal Care (4 visits) Coverage (proportion)
Sexually transmitted diseases excluding HIV	Female	10-14 years	95+ years	1	Age-Specific Fertility Rate
Sexually transmitted diseases excluding HIV	Female	10-14 years	95+ years	-1	Education (years per capita)
Sexually transmitted diseases excluding HIV	Female	10-14 years	95+ years	-1	LDI (I$ per capita)
Sexually transmitted diseases excluding HIV	Female	10-14 years	95+ years	1	Total Fertility Rate
Sexually transmitted diseases excluding HIV	Female	10-14 years	95+ years	-1	Health System Access (capped)
Sexually transmitted diseases excluding HIV	Female	10-14 years	95+ years	1	Syphilis prevalence (proportion)
Sexually transmitted diseases excluding HIV	Female	10-14 years	95+ years	-1	Healthcare access and quality index
Hepatitis	Female	28-364 days	95+ years	-1	Education (years per capita)
Hepatitis	Female	28-364 days	95+ years	-1	Health System Access 2 (unitless)
Hepatitis	Female	28-364 days	95+ years	-1	LDI (I$ per capita)
Hepatitis	Female	28-364 days	95+ years	-1	Sanitation (proportion with access)
Hepatitis	Female	28-364 days	95+ years	-1	Improved Water Source (proportion with access)
Hepatitis	Female	28-364 days	95+ years	1	Log-transformed SEV scalar: Hep
Hepatitis	Female	28-364 days	95+ years	-1	Socio-demographic Index
Hepatitis	Female	28-364 days	95+ years	1	Hepatitis B (HBsAg) Seroprevalence
Hepatitis	Female	28-364 days	95+ years	1	Hepatitis C (IgG) Seroprevalence
Hepatitis	Female	28-364 days	95+ years	1	Seroprevalence of anti-HAV (IgG)
Hepatitis	Female	28-364 days	95+ years	1	Seroprevalence of anti-HEV (IgG)
Hepatitis	Male	28-364 days	95+ years	-1	Education (years per capita)
Hepatitis	Male	28-364 days	95+ years	-1	Health System Access 2 (unitless)
Hepatitis	Male	28-364 days	95+ years	-1	LDI (I$ per capita)

Cause	Sex	Age start	Age end	Direction	Covariate
Hepatitis	Male	28-364 days	95+ years	-1	Sanitation (proportion with access)
Hepatitis	Male	28-364 days	95+ years	-1	Improved Water Source (proportion with access)
Hepatitis	Male	28-364 days	95+ years	1	Log-transformed SEV scalar: Hep
Hepatitis	Male	28-364 days	95+ years	-1	Socio-demographic Index
Hepatitis	Male	28-364 days	95+ years	1	Hepatitis B (HBsAg) Seroprevalence
Hepatitis	Male	28-364 days	95+ years	1	Hepatitis C (IgG) Seroprevalence
Hepatitis	Male	28-364 days	95+ years	1	Seroprevalence of anti-HAV (IgG)
Hepatitis	Male	28-364 days	95+ years	1	Seroprevalence of anti-HEV (IgG)
Other infectious diseases	Male	0-6 days	95+ years	1	Underweight (proportion <2SD weight for age, <5 years)
Other infectious diseases	Male	0-6 days	95+ years	-1	Sanitation (proportion with access)
Other infectious diseases	Male	0-6 days	95+ years	1	Rainfall Quintile 5 (proportion)
Other infectious diseases	Male	0-6 days	95+ years	-1	Education (years per capita)
Other infectious diseases	Male	0-6 days	95+ years	-1	DTP3 Coverage (proportion)
Other infectious diseases	Male	0-6 days	95+ years	-1	Health System Access (unitless)
Other infectious diseases	Male	0-6 days	95+ years	0	Latitude 30 to 45 (proportion)
Other infectious diseases	Male	0-6 days	95+ years	-1	Measles Vaccine Coverage (proportion)
Other infectious diseases	Male	0-6 days	95+ years	-1	Latitude Over 45 (proportion)
Other infectious diseases	Male	0-6 days	95+ years	0	Rainfall Quintile 3 (proportion)
Other infectious diseases	Male	0-6 days	95+ years	0	Rainfall Quintile 2 (proportion)
Other infectious diseases	Male	0-6 days	95+ years	-1	Rainfall Quintile 1 (proportion)
Other infectious diseases	Male	0-6 days	95+ years	-1	LDI (I$ per capita)
Other infectious diseases	Male	0-6 days	95+ years	0	Latitude 15 to 30 (proportion)
Other infectious diseases	Male	0-6 days	95+ years	-1	Antenatal Care (1 visit) Coverage (proportion)
Other infectious diseases	Male	0-6 days	95+ years	0	Rainfall Quintile 4 (proportion)
Other infectious diseases	Male	0-6 days	95+ years	-1	Improved Water Source (proportion with access)
Other infectious diseases	Male	0-6 days	95+ years	1	Latitude Under 15 (proportion)
Other infectious diseases	Male	0-6 days	95+ years	-1	Socio-demographic Index
Other infectious diseases	Female	0-6 days	95+ years	1	Underweight (proportion <2SD weight for age, <5 years)
Other infectious diseases	Female	0-6 days	95+ years	-1	Sanitation (proportion with access)
Other infectious diseases	Female	0-6 days	95+ years	1	Rainfall Quintile 5 (proportion)
Other infectious diseases	Female	0-6 days	95+ years	-1	Education (years per capita)
Other infectious diseases	Female	0-6 days	95+ years	-1	DTP3 Coverage (proportion)
Other infectious diseases	Female	0-6 days	95+ years	-1	Health System Access (unitless)
Other infectious diseases	Female	0-6 days	95+ years	0	Latitude 30 to 45 (proportion)
Other infectious diseases	Female	0-6 days	95+ years	-1	Measles Vaccine Coverage (proportion)
Other infectious diseases	Female	0-6 days	95+ years	-1	Latitude Over 45 (proportion)
Other infectious diseases	Female	0-6 days	95+ years	0	Rainfall Quintile 3 (proportion)
Other infectious diseases	Female	0-6 days	95+ years	0	Rainfall Quintile 2 (proportion)
Other infectious diseases	Female	0-6 days	95+ years	-1	Rainfall Quintile 1 (proportion)
Other infectious diseases	Female	0-6 days	95+ years	-1	LDI (I$ per capita)
Other infectious diseases	Female	0-6 days	95+ years	0	Latitude 15 to 30 (proportion)
Other infectious diseases	Female	0-6 days	95+ years	-1	Antenatal Care (1 visit) Coverage (proportion)
Other infectious diseases	Female	0-6 days	95+ years	0	Rainfall Quintile 4 (proportion)
Other infectious diseases	Female	0-6 days	95+ years	-1	Improved Water Source (proportion with access)
Other infectious diseases	Female	0-6 days	95+ years	1	Latitude Under 15 (proportion)
Other infectious diseases	Female	0-6 days	95+ years	-1	Socio-demographic Index
Other infectious diseases	Female	0-6 days	95+ years	-1	Health System Access (capped)
Other infectious diseases	Female	0-6 days	95+ years	-1	Healthcare access and quality index
Esophageal cancer	Male	15-19 years	95+ years	1	Alcohol (liters per capita)
Esophageal cancer	Male	15-19 years	95+ years	1	Tobacco (cigarettes per capita)
Esophageal cancer	Male	15-19 years	95+ years	-1	Education (years per capita)
Esophageal cancer	Male	15-19 years	95+ years	-1	Fruits (kcal per capita)
Esophageal cancer	Male	15-19 years	95+ years	-1	LDI (I$ per capita)
Esophageal cancer	Male	15-19 years	95+ years	1	Mean BMI
Esophageal cancer	Male	15-19 years	95+ years	1	Indoor Air Pollution (All Cooking Fuels)
Esophageal cancer	Male	15-19 years	95+ years	-1	Sanitation (proportion with access)
Esophageal cancer	Male	15-19 years	95+ years	1	Smoking Prevalence
Esophageal cancer	Male	15-19 years	95+ years	-1	Vegetables (kcal per capita)
Esophageal cancer	Male	15-19 years	95+ years	-1	Improved Water Source (proportion with access)
Esophageal cancer	Male	15-19 years	95+ years	1	Log-transformed age-standardized SEV scalar: Esophag C
Esophageal cancer	Male	15-19 years	95+ years	-1	Socio-demographic Index
Esophageal cancer	Male	15-19 years	95+ years	-1	Healthcare access and quality index
Esophageal cancer	Female	15-19 years	95+ years	1	Alcohol (liters per capita)
Esophageal cancer	Female	15-19 years	95+ years	1	Tobacco (cigarettes per capita)
Esophageal cancer	Female	15-19 years	95+ years	-1	Education (years per capita)
Esophageal cancer	Female	15-19 years	95+ years	-1	Fruits (kcal per capita)
Esophageal cancer	Female	15-19 years	95+ years	-1	LDI (I$ per capita)
Esophageal cancer	Female	15-19 years	95+ years	1	Mean BMI
Esophageal cancer	Female	15-19 years	95+ years	1	Indoor Air Pollution (All Cooking Fuels)
Esophageal cancer	Female	15-19 years	95+ years	-1	Sanitation (proportion with access)
Esophageal cancer	Female	15-19 years	95+ years	1	Smoking Prevalence
Esophageal cancer	Female	15-19 years	95+ years	-1	Vegetables (kcal per capita)
Esophageal cancer	Female	15-19 years	95+ years	-1	Improved Water Source (proportion with access)
Esophageal cancer	Female	15-19 years	95+ years	1	Log-transformed age-standardized SEV scalar: Esophag C
Esophageal cancer	Female	15-19 years	95+ years	0	Socio-demographic Index
Esophageal cancer	Female	15-19 years	95+ years	-1	Healthcare access and quality index
Esophageal cancer	Female	15-19 years	95+ years	0	LDI (I$ per capita)
Esophageal cancer	Female	15-19 years	95+ years	-1	Socio-demographic Index
Esophageal cancer	Female	15-19 years	95+ years	-1	fruits adjusted(g)
Esophageal cancer	Female	15-19 years	95+ years	-1	vegetables adjusted(g)
Esophageal cancer	Male	15-19 years	95+ years	0	LDI (I$ per capita)
Esophageal cancer	Male	15-19 years	95+ years	1	Log-transformed SEV scalar: Esophag C
Esophageal cancer	Male	15-19 years	95+ years	-1	fruits adjusted(g)
Esophageal cancer	Male	15-19 years	95+ years	-1	vegetables adjusted(g)
Stomach cancer	Male	15-19 years	95+ years	1	Alcohol (liters per capita)
Stomach cancer	Male	15-19 years	95+ years	1	Tobacco (cigarettes per capita)
Stomach cancer	Male	15-19 years	95+ years	1	Cumulative Cigarettes (10 Years)
Stomach cancer	Male	15-19 years	95+ years	1	Cumulative Cigarettes (15 Years)
Stomach cancer	Male	15-19 years	95+ years	0	LDI (I$ per capita)
Stomach cancer	Male	15-19 years	95+ years	1	Mean BMI
Stomach cancer	Male	15-19 years	95+ years	1	Indoor Air Pollution (All Cooking Fuels)
Stomach cancer	Male	15-19 years	95+ years	1	Outdoor Air Pollution (PM2.5)
Stomach cancer	Male	15-19 years	95+ years	-1	Sanitation (proportion with access)
Stomach cancer	Male	15-19 years	95+ years	1	Smoking Prevalence
Stomach cancer	Male	15-19 years	95+ years	-1	Improved Water Source (proportion with access)

Cause	Sex	Age start	Age end	Direction	Covariate
Stomach cancer	Male	15-19 years	95+ years	1	Log-transformed SEV scalar: Stomach C
Stomach cancer	Male	15-19 years	95+ years	0	Socio-demographic Index
Stomach cancer	Male	15-19 years	95+ years	-1	fruits adjusted(g)
Stomach cancer	Male	15-19 years	95+ years	-1	vegetables adjusted(g)
Stomach cancer	Male	15-19 years	95+ years	-1	Healthcare access and quality index
Stomach cancer	Male	15-19 years	95+ years	1	Diet high in sodium
Stomach cancer	Female	15-19 years	95+ years	1	Alcohol (liters per capita)
Stomach cancer	Female	15-19 years	95+ years	1	Tobacco (cigarettes per capita)
Stomach cancer	Female	15-19 years	95+ years	1	Cumulative Cigarettes (10 Years)
Stomach cancer	Female	15-19 years	95+ years	-1	Education (years per capita)
Stomach cancer	Female	15-19 years	95+ years	0	LDI (I$ per capita)
Stomach cancer	Female	15-19 years	95+ years	1	Mean BMI
Stomach cancer	Female	15-19 years	95+ years	1	Indoor Air Pollution (All Cooking Fuels)
Stomach cancer	Female	15-19 years	95+ years	1	Outdoor Air Pollution (PM2.5)
Stomach cancer	Female	15-19 years	95+ years	-1	Sanitation (proportion with access)
Stomach cancer	Female	15-19 years	95+ years	1	Smoking Prevalence
Stomach cancer	Female	15-19 years	95+ years	-1	Improved Water Source (proportion with access)
Stomach cancer	Female	15-19 years	95+ years	1	Log-transformed SEV scalar: Stomach C
Stomach cancer	Female	15-19 years	95+ years	0	Socio-demographic Index
Stomach cancer	Female	15-19 years	95+ years	-1	fruits adjusted(g)
Stomach cancer	Female	15-19 years	95+ years	-1	vegetables adjusted(g)
Stomach cancer	Female	15-19 years	95+ years	-1	Healthcare access and quality index
Stomach cancer	Female	15-19 years	95+ years	1	Diet high in sodium
Liver cancer	Male	5-9 years	95+ years	1	Alcohol (liters per capita)
Liver cancer	Male	5-9 years	95+ years	1	Tobacco (cigarettes per capita)
Liver cancer	Male	5-9 years	95+ years	1	Cumulative Cigarettes (15 Years)
Liver cancer	Male	5-9 years	95+ years	1	Cumulative Cigarettes (20 Years)
Liver cancer	Male	5-9 years	95+ years	1	Diabetes Age-Standardized Prevalence (proportion)
Liver cancer	Male	5-9 years	95+ years	-1	Education (years per capita)
Liver cancer	Male	5-9 years	95+ years	0	LDI (I$ per capita)
Liver cancer	Male	5-9 years	95+ years	1	Mean BMI
Liver cancer	Male	5-9 years	95+ years	1	Percent of total calories consumed as saturated fat
Liver cancer	Male	5-9 years	95+ years	1	Log-transformed SEV scalar: Liver C
Liver cancer	Male	5-9 years	95+ years	0	Socio-demographic Index
Liver cancer	Male	5-9 years	95+ years	1	red meats adjusted(g)
Liver cancer	Male	5-9 years	95+ years	1	Hepatitis B (HBsAg) Seroprevalence
Liver cancer	Male	5-9 years	95+ years	1	Hepatitis C (IgG) Seroprevalence
Liver cancer	Male	5-9 years	95+ years	-1	Healthcare access and quality index
Liver cancer	Female	5-9 years	95+ years	1	Alcohol (liters per capita)
Liver cancer	Female	5-9 years	95+ years	1	Tobacco (cigarettes per capita)
Liver cancer	Female	5-9 years	95+ years	1	Cumulative Cigarettes (15 Years)
Liver cancer	Female	5-9 years	95+ years	1	Cumulative Cigarettes (20 Years)
Liver cancer	Female	5-9 years	95+ years	1	Diabetes Age-Standardized Prevalence (proportion)
Liver cancer	Female	5-9 years	95+ years	-1	Education (years per capita)
Liver cancer	Female	5-9 years	95+ years	0	LDI (I$ per capita)
Liver cancer	Female	5-9 years	95+ years	1	Mean BMI
Liver cancer	Female	5-9 years	95+ years	1	Percent of total calories consumed as saturated fat
Liver cancer	Female	5-9 years	95+ years	1	Log-transformed SEV scalar: Liver C
Liver cancer	Female	5-9 years	95+ years	0	Socio-demographic Index
Liver cancer	Female	5-9 years	95+ years	1	red meats adjusted(g)
Liver cancer	Female	5-9 years	95+ years	1	Hepatitis B (HBsAg) Seroprevalence
Liver cancer	Female	5-9 years	95+ years	1	Hepatitis C (IgG) Seroprevalence
Liver cancer	Female	5-9 years	95+ years	-1	Healthcare access and quality index
Larynx cancer	Male	15-19 years	95+ years	1	Alcohol (liters per capita)
Larynx cancer	Male	15-19 years	95+ years	1	Tobacco (cigarettes per capita)
Larynx cancer	Male	15-19 years	95+ years	1	Cumulative Cigarettes (10 Years)
Larynx cancer	Male	15-19 years	95+ years	1	Cumulative Cigarettes (15 Years)
Larynx cancer	Male	15-19 years	95+ years	1	Cumulative Cigarettes (20 Years)
Larynx cancer	Male	15-19 years	95+ years	1	Cumulative Cigarettes (5 Years)
Larynx cancer	Male	15-19 years	95+ years	-1	Education (years per capita)
Larynx cancer	Male	15-19 years	95+ years	0	LDI (I$ per capita)
Larynx cancer	Male	15-19 years	95+ years	1	Population Density (over 1000 ppl/sqkm, proportion)
Larynx cancer	Male	15-19 years	95+ years	1	Population Density (under 150 ppl/sqkm, proportion)
Larynx cancer	Male	15-19 years	95+ years	1	Smoking Prevalence
Larynx cancer	Male	15-19 years	95+ years	1	Log-transformed SEV scalar: Larynx C
Larynx cancer	Male	15-19 years	95+ years	0	Socio-demographic Index
Larynx cancer	Male	15-19 years	95+ years	-1	fruits adjusted(g)
Larynx cancer	Male	15-19 years	95+ years	-1	vegetables adjusted(g)
Larynx cancer	Male	15-19 years	95+ years	-1	Healthcare access and quality index
Larynx cancer	Female	15-19 years	95+ years	1	Alcohol (liters per capita)
Larynx cancer	Female	15-19 years	95+ years	1	Tobacco (cigarettes per capita)
Larynx cancer	Female	15-19 years	95+ years	1	Cumulative Cigarettes (10 Years)
Larynx cancer	Female	15-19 years	95+ years	1	Cumulative Cigarettes (15 Years)
Larynx cancer	Female	15-19 years	95+ years	1	Cumulative Cigarettes (20 Years)
Larynx cancer	Female	15-19 years	95+ years	1	Cumulative Cigarettes (5 Years)
Larynx cancer	Female	15-19 years	95+ years	-1	Education (years per capita)
Larynx cancer	Female	15-19 years	95+ years	0	LDI (I$ per capita)
Larynx cancer	Female	15-19 years	95+ years	1	Population Density (over 1000 ppl/sqkm, proportion)
Larynx cancer	Female	15-19 years	95+ years	1	Population Density (under 150 ppl/sqkm, proportion)
Larynx cancer	Female	15-19 years	95+ years	1	Smoking Prevalence
Larynx cancer	Female	15-19 years	95+ years	1	Log-transformed SEV scalar: Larynx C
Larynx cancer	Female	15-19 years	95+ years	0	Socio-demographic Index
Larynx cancer	Female	15-19 years	95+ years	-1	fruits adjusted(g)
Larynx cancer	Female	15-19 years	95+ years	-1	vegetables adjusted(g)
Larynx cancer	Female	15-19 years	95+ years	-1	Healthcare access and quality index
Tracheal, bronchus, and lung cancer	Female	15-19 years	95+ years	1	Tobacco (cigarettes per capita)
Tracheal, bronchus, and lung cancer	Female	15-19 years	95+ years	1	Cumulative Cigarettes (10 Years)
Tracheal, bronchus, and lung cancer	Female	15-19 years	95+ years	1	Cumulative Cigarettes (15 Years)
Tracheal, bronchus, and lung cancer	Female	15-19 years	95+ years	1	Cumulative Cigarettes (20 Years)
Tracheal, bronchus, and lung cancer	Female	15-19 years	95+ years	1	Cumulative Cigarettes (5 Years)
Tracheal, bronchus, and lung cancer	Female	15-19 years	95+ years	0	Education (years per capita)
Tracheal, bronchus, and lung cancer	Female	15-19 years	95+ years	0	LDI (I$ per capita)
Tracheal, bronchus, and lung cancer	Female	15-19 years	95+ years	1	Indoor Air Pollution (All Cooking Fuels)
Tracheal, bronchus, and lung cancer	Female	15-19 years	95+ years	1	Outdoor Air Pollution (PM2.5)
Tracheal, bronchus, and lung cancer	Female	15-19 years	95+ years	1	Smoking Prevalence

Cause	Sex	Age start	Age end	Direction	Covariate
Tracheal, bronchus, and lung cancer	Female	15-19 years	95+ years	1	Log-transformed SEV scalar: Lung C
Tracheal, bronchus, and lung cancer	Female	15-19 years	95+ years	1	Log-transformed age-standardized SEV scalar: Lung C
Tracheal, bronchus, and lung cancer	Female	15-19 years	95+ years	0	Socio-demographic Index
Tracheal, bronchus, and lung cancer	Female	15-19 years	95+ years	-1	Healthcare access and quality index
Tracheal, bronchus, and lung cancer	Male	15-19 years	95+ years	1	Tobacco (cigarettes per capita)
Tracheal, bronchus, and lung cancer	Male	15-19 years	95+ years	1	Cumulative Cigarettes (10 Years)
Tracheal, bronchus, and lung cancer	Male	15-19 years	95+ years	1	Cumulative Cigarettes (15 Years)
Tracheal, bronchus, and lung cancer	Male	15-19 years	95+ years	1	Cumulative Cigarettes (20 Years)
Tracheal, bronchus, and lung cancer	Male	15-19 years	95+ years	1	Cumulative Cigarettes (5 Years)
Tracheal, bronchus, and lung cancer	Male	15-19 years	95+ years	0	Education (years per capita)
Tracheal, bronchus, and lung cancer	Male	15-19 years	95+ years	0	LDI (I$ per capita)
Tracheal, bronchus, and lung cancer	Male	15-19 years	95+ years	1	Indoor Air Pollution (All Cooking Fuels)
Tracheal, bronchus, and lung cancer	Male	15-19 years	95+ years	1	Outdoor Air Pollution (PM2.5)
Tracheal, bronchus, and lung cancer	Male	15-19 years	95+ years	1	Smoking Prevalence
Tracheal, bronchus, and lung cancer	Male	15-19 years	95+ years	1	Log-transformed SEV scalar: Lung C
Tracheal, bronchus, and lung cancer	Male	15-19 years	95+ years	1	Log-transformed age-standardized SEV scalar: Lung C
Tracheal, bronchus, and lung cancer	Male	15-19 years	95+ years	0	Socio-demographic Index
Tracheal, bronchus, and lung cancer	Male	15-19 years	95+ years	-1	Healthcare access and quality index
Breast cancer	Male	15-19 years	95+ years	1	Alcohol (liters per capita)
Breast cancer	Male	15-19 years	95+ years	1	Cumulative Cigarettes (10 Years)
Breast cancer	Male	15-19 years	95+ years	-1	Education (years per capita)
Breast cancer	Male	15-19 years	95+ years	1	Saturated Fats (kcal per capita)
Breast cancer	Male	15-19 years	95+ years	0	LDI (I$ per capita)
Breast cancer	Male	15-19 years	95+ years	1	Mean BMI
Breast cancer	Male	15-19 years	95+ years	1	Log-transformed SEV scalar: Breast C
Breast cancer	Male	15-19 years	95+ years	0	Socio-demographic Index
Breast cancer	Male	15-19 years	95+ years	-1	fruits adjusted(g)
Breast cancer	Male	15-19 years	95+ years	-1	vegetables adjusted(g)
Breast cancer	Male	15-19 years	95+ years	-1	Healthcare access and quality index
Breast cancer	Female	15-19 years	95+ years	1	Alcohol (liters per capita)
Breast cancer	Female	15-19 years	95+ years	-1	Age-Specific Fertility Rate
Breast cancer	Female	15-19 years	95+ years	1	Cumulative Cigarettes (10 Years)
Breast cancer	Female	15-19 years	95+ years	-1	Education (years per capita)
Breast cancer	Female	15-19 years	95+ years	1	Saturated Fats (kcal per capita)
Breast cancer	Female	15-19 years	95+ years	0	LDI (I$ per capita)
Breast cancer	Female	15-19 years	95+ years	1	Mean BMI
Breast cancer	Female	15-19 years	95+ years	-1	Total Fertility Rate
Breast cancer	Female	15-19 years	95+ years	1	Log-transformed SEV scalar: Breast C
Breast cancer	Female	15-19 years	95+ years	0	Socio-demographic Index
Breast cancer	Female	15-19 years	95+ years	-1	fruits adjusted(g)
Breast cancer	Female	15-19 years	95+ years	-1	vegetables adjusted(g)
Breast cancer	Female	15-19 years	95+ years	-1	Healthcare access and quality index
Cervical cancer	Female	15-19 years	95+ years	1	Abortion On-Demand Illegal (binary)
Cervical cancer	Female	15-19 years	95+ years	1	Age-Specific Fertility Rate
Cervical cancer	Female	15-19 years	95+ years	1	Cumulative Cigarettes (10 Years)
Cervical cancer	Female	15-19 years	95+ years	1	Cumulative Cigarettes (15 Years)
Cervical cancer	Female	15-19 years	95+ years	1	Cumulative Cigarettes (5 Years)
Cervical cancer	Female	15-19 years	95+ years	-1	Education (years per capita)
Cervical cancer	Female	15-19 years	95+ years	-1	Health System Access 2 (unitless)
Cervical cancer	Female	15-19 years	95+ years	0	LDI (I$ per capita)
Cervical cancer	Female	15-19 years	95+ years	1	Smoking Prevalence
Cervical cancer	Female	15-19 years	95+ years	1	Total Fertility Rate
Cervical cancer	Female	15-19 years	95+ years	0	Socio-demographic Index
Cervical cancer	Female	15-19 years	95+ years	-1	fruits adjusted(g)
Cervical cancer	Female	15-19 years	95+ years	-1	vegetables adjusted(g)
Cervical cancer	Female	15-19 years	95+ years	1	HIV age-standardized prevalence
Cervical cancer	Female	15-19 years	95+ years	-1	Healthcare access and quality index
Uterine cancer	Female	15-19 years	95+ years	1	Tobacco (cigarettes per capita)
Uterine cancer	Female	15-19 years	95+ years	1	Cumulative Cigarettes (10 Years)
Uterine cancer	Female	15-19 years	95+ years	1	Cumulative Cigarettes (5 Years)
Uterine cancer	Female	15-19 years	95+ years	1	Diabetes Age-Standardized Prevalence (proportion)
Uterine cancer	Female	15-19 years	95+ years	-1	Education (years per capita)
Uterine cancer	Female	15-19 years	95+ years	-1	Health System Access (unitless)
Uterine cancer	Female	15-19 years	95+ years	0	LDI (I$ per capita)
Uterine cancer	Female	15-19 years	95+ years	1	Mean BMI
Uterine cancer	Female	15-19 years	95+ years	1	Smoking Prevalence
Uterine cancer	Female	15-19 years	95+ years	0	Total Fertility Rate
Uterine cancer	Female	15-19 years	95+ years	1	Log-transformed SEV scalar: Uterus C
Uterine cancer	Female	15-19 years	95+ years	0	Socio-demographic Index
Uterine cancer	Female	15-19 years	95+ years	-1	fruits adjusted(g)
Uterine cancer	Female	15-19 years	95+ years	-1	vegetables adjusted(g)
Uterine cancer	Female	15-19 years	95+ years	-1	Healthcare access and quality index
Prostate cancer	Male	15-19 years	95+ years	0	Education (years per capita)
Prostate cancer	Male	15-19 years	95+ years	0	LDI (I$ per capita)
Prostate cancer	Male	15-19 years	95+ years	1	Percent of total calories consumed as saturated fat
Prostate cancer	Male	15-19 years	95+ years	1	Log-transformed SEV scalar: Prostate C
Prostate cancer	Male	15-19 years	95+ years	0	Socio-demographic Index
Prostate cancer	Male	15-19 years	95+ years	-1	Healthcare access and quality index
Colon and rectum cancer	Male	15-19 years	95+ years	1	Alcohol (liters per capita)
Colon and rectum cancer	Male	15-19 years	95+ years	1	Tobacco (cigarettes per capita)
Colon and rectum cancer	Male	15-19 years	95+ years	1	Diabetes Age-Standardized Prevalence (proportion)
Colon and rectum cancer	Male	15-19 years	95+ years	-1	Education (years per capita)
Colon and rectum cancer	Male	15-19 years	95+ years	-1	Health System Access 2 (unitless)
Colon and rectum cancer	Male	15-19 years	95+ years	0	LDI (I$ per capita)
Colon and rectum cancer	Male	15-19 years	95+ years	1	Mean BMI
Colon and rectum cancer	Male	15-19 years	95+ years	1	Smoking Prevalence
Colon and rectum cancer	Male	15-19 years	95+ years	1	Log-transformed SEV scalar: Colorect C
Colon and rectum cancer	Male	15-19 years	95+ years	0	Socio-demographic Index
Colon and rectum cancer	Male	15-19 years	95+ years	-1	fruits adjusted(g)
Colon and rectum cancer	Male	15-19 years	95+ years	-1	milk adjusted(g)
Colon and rectum cancer	Male	15-19 years	95+ years	-1	nuts seeds adjusted(g)
Colon and rectum cancer	Male	15-19 years	95+ years	-1	pufa adjusted(percent)
Colon and rectum cancer	Male	15-19 years	95+ years	1	red meats adjusted(g)
Colon and rectum cancer	Male	15-19 years	95+ years	-1	vegetables adjusted(g)
Colon and rectum cancer	Male	15-19 years	95+ years	-1	whole grains adjusted(g)

Cause	Sex	Age start	Age end	Direction	Covariate
Colon and rectum cancer	Male	15-19 years	95+ years	-1	Healthcare access and quality index
Colon and rectum cancer	Female	15-19 years	95+ years	1	Alcohol (liters per capita)
Colon and rectum cancer	Female	15-19 years	95+ years	1	Tobacco (cigarettes per capita)
Colon and rectum cancer	Female	15-19 years	95+ years	1	Diabetes Age-Standardized Prevalence (proportion)
Colon and rectum cancer	Female	15-19 years	95+ years	-1	Education (years per capita)
Colon and rectum cancer	Female	15-19 years	95+ years	-1	Health System Access 2 (unitless)
Colon and rectum cancer	Female	15-19 years	95+ years	0	LDI (I$ per capita)
Colon and rectum cancer	Female	15-19 years	95+ years	1	Mean BMI
Colon and rectum cancer	Female	15-19 years	95+ years	1	Smoking Prevalence
Colon and rectum cancer	Female	15-19 years	95+ years	1	Log-transformed SEV scalar: Colorect C
Colon and rectum cancer	Female	15-19 years	95+ years	0	Socio-demographic Index
Colon and rectum cancer	Female	15-19 years	95+ years	-1	fruits adjusted(g)
Colon and rectum cancer	Female	15-19 years	95+ years	-1	milk adjusted(g)
Colon and rectum cancer	Female	15-19 years	95+ years	-1	nuts seeds adjusted(g)
Colon and rectum cancer	Female	15-19 years	95+ years	-1	pufa adjusted(percent)
Colon and rectum cancer	Female	15-19 years	95+ years	1	red meats adjusted(g)
Colon and rectum cancer	Female	15-19 years	95+ years	-1	vegetables adjusted(g)
Colon and rectum cancer	Female	15-19 years	95+ years	-1	whole grains adjusted(g)
Colon and rectum cancer	Female	15-19 years	95+ years	-1	Healthcare access and quality index
Lip and oral cavity cancer	Male	15-19 years	95+ years	1	Alcohol (liters per capita)
Lip and oral cavity cancer	Male	15-19 years	95+ years	1	Tobacco (cigarettes per capita)
Lip and oral cavity cancer	Male	15-19 years	95+ years	1	Cumulative Cigarettes (10 Years)
Lip and oral cavity cancer	Male	15-19 years	95+ years	1	Cumulative Cigarettes (15 Years)
Lip and oral cavity cancer	Male	15-19 years	95+ years	1	Cumulative Cigarettes (20 Years)
Lip and oral cavity cancer	Male	15-19 years	95+ years	1	Cumulative Cigarettes (5 Years)
Lip and oral cavity cancer	Male	15-19 years	95+ years	-1	Education (years per capita)
Lip and oral cavity cancer	Male	15-19 years	95+ years	-1	Health System Access 2 (unitless)
Lip and oral cavity cancer	Male	15-19 years	95+ years	0	LDI (I$ per capita)
Lip and oral cavity cancer	Male	15-19 years	95+ years	1	Smoking Prevalence
Lip and oral cavity cancer	Male	15-19 years	95+ years	1	Log-transformed SEV scalar: Mouth C
Lip and oral cavity cancer	Male	15-19 years	95+ years	0	Socio-demographic Index
Lip and oral cavity cancer	Male	15-19 years	95+ years	-1	fruits adjusted(g)
Lip and oral cavity cancer	Male	15-19 years	95+ years	1	red meats adjusted(g)
Lip and oral cavity cancer	Male	15-19 years	95+ years	-1	vegetables adjusted(g)
Lip and oral cavity cancer	Male	15-19 years	95+ years	-1	Healthcare access and quality index
Lip and oral cavity cancer	Female	15-19 years	95+ years	1	Alcohol (liters per capita)
Lip and oral cavity cancer	Female	15-19 years	95+ years	1	Cumulative Cigarettes (10 Years)
Lip and oral cavity cancer	Female	15-19 years	95+ years	1	Cumulative Cigarettes (20 Years)
Lip and oral cavity cancer	Female	15-19 years	95+ years	-1	Education (years per capita)
Lip and oral cavity cancer	Female	15-19 years	95+ years	-1	Fruits (kcal per capita)
Lip and oral cavity cancer	Female	15-19 years	95+ years	-1	Health System Access 2 (unitless)
Lip and oral cavity cancer	Female	15-19 years	95+ years	-1	LDI (I$ per capita)
Lip and oral cavity cancer	Female	15-19 years	95+ years	1	Red Meat (kcal per capita)
Lip and oral cavity cancer	Female	15-19 years	95+ years	1	Smoking Prevalence
Lip and oral cavity cancer	Female	15-19 years	95+ years	-1	Vegetables (kcal per capita)
Lip and oral cavity cancer	Female	15-19 years	95+ years	0	Socio-demographic Index
Lip and oral cavity cancer	Female	15-19 years	95+ years	-1	Healthcare access and quality index
Nasopharynx cancer	Female	5-9 years	95+ years	1	Alcohol (liters per capita)
Nasopharynx cancer	Female	5-9 years	95+ years	1	Tobacco (cigarettes per capita)
Nasopharynx cancer	Female	5-9 years	95+ years	1	Cumulative Cigarettes (10 Years)
Nasopharynx cancer	Female	5-9 years	95+ years	1	Cumulative Cigarettes (15 Years)
Nasopharynx cancer	Female	5-9 years	95+ years	1	Cumulative Cigarettes (20 Years)
Nasopharynx cancer	Female	5-9 years	95+ years	1	Cumulative Cigarettes (5 Years)
Nasopharynx cancer	Female	5-9 years	95+ years	-1	Education (years per capita)
Nasopharynx cancer	Female	5-9 years	95+ years	-1	Health System Access 2 (unitless)
Nasopharynx cancer	Female	5-9 years	95+ years	0	LDI (I$ per capita)
Nasopharynx cancer	Female	5-9 years	95+ years	1	Population Density (over 1000 ppl/sqkm, proportion)
Nasopharynx cancer	Female	5-9 years	95+ years	1	Population Density (under 150 ppl/sqkm, proportion)
Nasopharynx cancer	Female	5-9 years	95+ years	1	Smoking Prevalence
Nasopharynx cancer	Female	5-9 years	95+ years	1	Log-transformed SEV scalar: Nasoph C
Nasopharynx cancer	Female	5-9 years	95+ years	0	Socio-demographic Index
Nasopharynx cancer	Female	5-9 years	95+ years	-1	fruits adjusted(g)
Nasopharynx cancer	Female	5-9 years	95+ years	-1	vegetables adjusted(g)
Nasopharynx cancer	Female	5-9 years	95+ years	-1	whole grains adjusted(g)
Nasopharynx cancer	Male	5-9 years	95+ years	1	Alcohol (liters per capita)
Nasopharynx cancer	Male	5-9 years	95+ years	1	Tobacco (cigarettes per capita)
Nasopharynx cancer	Male	5-9 years	95+ years	1	Cumulative Cigarettes (10 Years)
Nasopharynx cancer	Male	5-9 years	95+ years	1	Cumulative Cigarettes (15 Years)
Nasopharynx cancer	Male	5-9 years	95+ years	1	Cumulative Cigarettes (20 Years)
Nasopharynx cancer	Male	5-9 years	95+ years	1	Cumulative Cigarettes (5 Years)
Nasopharynx cancer	Male	5-9 years	95+ years	-1	Education (years per capita)
Nasopharynx cancer	Male	5-9 years	95+ years	-1	Health System Access 2 (unitless)
Nasopharynx cancer	Male	5-9 years	95+ years	0	LDI (I$ per capita)
Nasopharynx cancer	Male	5-9 years	95+ years	1	Population Density (over 1000 ppl/sqkm, proportion)
Nasopharynx cancer	Male	5-9 years	95+ years	1	Population Density (under 150 ppl/sqkm, proportion)
Nasopharynx cancer	Male	5-9 years	95+ years	1	Smoking Prevalence
Nasopharynx cancer	Male	5-9 years	95+ years	1	Log-transformed SEV scalar: Nasoph C
Nasopharynx cancer	Male	5-9 years	95+ years	0	Socio-demographic Index
Nasopharynx cancer	Male	5-9 years	95+ years	-1	fruits adjusted(g)
Nasopharynx cancer	Male	5-9 years	95+ years	-1	vegetables adjusted(g)
Nasopharynx cancer	Male	5-9 years	95+ years	-1	whole grains adjusted(g)
Other pharynx cancer	Male	15-19 years	95+ years	1	Alcohol (liters per capita)
Other pharynx cancer	Male	15-19 years	95+ years	1	Cumulative Cigarettes (5 Years)
Other pharynx cancer	Male	15-19 years	95+ years	-1	Education (years per capita)
Other pharynx cancer	Male	15-19 years	95+ years	0	LDI (I$ per capita)
Other pharynx cancer	Male	15-19 years	95+ years	1	Population Density (over 1000 ppl/sqkm, proportion)
Other pharynx cancer	Male	15-19 years	95+ years	1	Population Density (under 150 ppl/sqkm, proportion)
Other pharynx cancer	Male	15-19 years	95+ years	1	Smoking Prevalence
Other pharynx cancer	Male	15-19 years	95+ years	-1	Health System Access (capped)
Other pharynx cancer	Male	15-19 years	95+ years	1	Log-transformed SEV scalar: Oth Phar C
Other pharynx cancer	Male	15-19 years	95+ years	0	Socio-demographic Index
Other pharynx cancer	Male	15-19 years	95+ years	-1	fruits adjusted(g)
Other pharynx cancer	Male	15-19 years	95+ years	-1	vegetables adjusted(g)
Other pharynx cancer	Male	15-19 years	95+ years	-1	whole grains adjusted(g)
Other pharynx cancer	Female	15-19 years	95+ years	1	Alcohol (liters per capita)

Cause	Sex	Age start	Age end	Direction	Covariate
Other pharynx cancer	Female	15-19 years	95+ years	1	Cumulative Cigarettes (5 Years)
Other pharynx cancer	Female	15-19 years	95+ years	-1	Education (years per capita)
Other pharynx cancer	Female	15-19 years	95+ years	0	LDI (I$ per capita)
Other pharynx cancer	Female	15-19 years	95+ years	1	Population Density (over 1000 ppl/sqkm, proportion)
Other pharynx cancer	Female	15-19 years	95+ years	1	Population Density (under 150 ppl/sqkm, proportion)
Other pharynx cancer	Female	15-19 years	95+ years	1	Smoking Prevalence
Other pharynx cancer	Female	15-19 years	95+ years	-1	Health System Access (capped)
Other pharynx cancer	Female	15-19 years	95+ years	1	Log-transformed SEV scalar: Oth Phar C
Other pharynx cancer	Female	15-19 years	95+ years	0	Socio-demographic Index
Other pharynx cancer	Female	15-19 years	95+ years	-1	fruits adjusted(g)
Other pharynx cancer	Female	15-19 years	95+ years	-1	vegetables adjusted(g)
Other pharynx cancer	Female	15-19 years	95+ years	-1	whole grains adjusted(g)
Gallbladder and biliary tract cancer	Female	15-19 years	95+ years	1	Alcohol (liters per capita)
Gallbladder and biliary tract cancer	Female	15-19 years	95+ years	1	Tobacco (cigarettes per capita)
Gallbladder and biliary tract cancer	Female	15-19 years	95+ years	1	Cumulative Cigarettes (10 Years)
Gallbladder and biliary tract cancer	Female	15-19 years	95+ years	1	Cumulative Cigarettes (5 Years)
Gallbladder and biliary tract cancer	Female	15-19 years	95+ years	1	Diabetes Age-Standardized Prevalence (proportion)
Gallbladder and biliary tract cancer	Female	15-19 years	95+ years	-1	Education (years per capita)
Gallbladder and biliary tract cancer	Female	15-19 years	95+ years	1	Health System Access 2 (unitless)
Gallbladder and biliary tract cancer	Female	15-19 years	95+ years	0	LDI (I$ per capita)
Gallbladder and biliary tract cancer	Female	15-19 years	95+ years	1	Mean BMI
Gallbladder and biliary tract cancer	Female	15-19 years	95+ years	1	Smoking Prevalence
Gallbladder and biliary tract cancer	Female	15-19 years	95+ years	1	Log-transformed SEV scalar: Gallblad C
Gallbladder and biliary tract cancer	Female	15-19 years	95+ years	0	Socio-demographic Index
Gallbladder and biliary tract cancer	Female	15-19 years	95+ years	-1	fruits adjusted(g)
Gallbladder and biliary tract cancer	Female	15-19 years	95+ years	-1	vegetables adjusted(g)
Gallbladder and biliary tract cancer	Female	15-19 years	95+ years	-1	Healthcare access and quality index
Gallbladder and biliary tract cancer	Female	15-19 years	95+ years	-1	Health System Access (capped)
Gallbladder and biliary tract cancer	Male	15-19 years	95+ years	1	Alcohol (liters per capita)
Gallbladder and biliary tract cancer	Male	15-19 years	95+ years	1	Tobacco (cigarettes per capita)
Gallbladder and biliary tract cancer	Male	15-19 years	95+ years	1	Cumulative Cigarettes (10 Years)
Gallbladder and biliary tract cancer	Male	15-19 years	95+ years	1	Cumulative Cigarettes (5 Years)
Gallbladder and biliary tract cancer	Male	15-19 years	95+ years	1	Diabetes Age-Standardized Prevalence (proportion)
Gallbladder and biliary tract cancer	Male	15-19 years	95+ years	-1	Education (years per capita)
Gallbladder and biliary tract cancer	Male	15-19 years	95+ years	0	LDI (I$ per capita)
Gallbladder and biliary tract cancer	Male	15-19 years	95+ years	1	Mean BMI
Gallbladder and biliary tract cancer	Male	15-19 years	95+ years	1	Smoking Prevalence
Gallbladder and biliary tract cancer	Male	15-19 years	95+ years	-1	Health System Access (capped)
Gallbladder and biliary tract cancer	Male	15-19 years	95+ years	1	Log-transformed SEV scalar: Gallblad C
Gallbladder and biliary tract cancer	Male	15-19 years	95+ years	0	Socio-demographic Index
Gallbladder and biliary tract cancer	Male	15-19 years	95+ years	-1	fruits adjusted(g)
Gallbladder and biliary tract cancer	Male	15-19 years	95+ years	-1	vegetables adjusted(g)
Gallbladder and biliary tract cancer	Male	15-19 years	95+ years	-1	Healthcare access and quality index
Pancreatic cancer	Male	15-19 years	95+ years	1	Alcohol (liters per capita)
Pancreatic cancer	Male	15-19 years	95+ years	1	Tobacco (cigarettes per capita)
Pancreatic cancer	Male	15-19 years	95+ years	1	Cumulative Cigarettes (10 Years)
Pancreatic cancer	Male	15-19 years	95+ years	1	Cumulative Cigarettes (20 Years)
Pancreatic cancer	Male	15-19 years	95+ years	1	Cumulative Cigarettes (5 Years)
Pancreatic cancer	Male	15-19 years	95+ years	1	Diabetes Age-Standardized Prevalence (proportion)
Pancreatic cancer	Male	15-19 years	95+ years	-1	Education (years per capita)
Pancreatic cancer	Male	15-19 years	95+ years	0	LDI (I$ per capita)
Pancreatic cancer	Male	15-19 years	95+ years	1	Mean BMI
Pancreatic cancer	Male	15-19 years	95+ years	1	Percent of total calories consumed as saturated fat
Pancreatic cancer	Male	15-19 years	95+ years	1	Smoking Prevalence
Pancreatic cancer	Male	15-19 years	95+ years	1	Total Calories (kcal per capita)
Pancreatic cancer	Male	15-19 years	95+ years	1	Log-transformed SEV scalar: Pancreas C
Pancreatic cancer	Male	15-19 years	95+ years	0	Socio-demographic Index
Pancreatic cancer	Male	15-19 years	95+ years	-1	fruits adjusted(g)
Pancreatic cancer	Male	15-19 years	95+ years	1	red meats adjusted(g)
Pancreatic cancer	Male	15-19 years	95+ years	-1	vegetables adjusted(g)
Pancreatic cancer	Male	15-19 years	95+ years	-1	Healthcare access and quality index
Pancreatic cancer	Female	15-19 years	95+ years	1	Alcohol (liters per capita)
Pancreatic cancer	Female	15-19 years	95+ years	1	Tobacco (cigarettes per capita)
Pancreatic cancer	Female	15-19 years	95+ years	1	Cumulative Cigarettes (10 Years)
Pancreatic cancer	Female	15-19 years	95+ years	1	Cumulative Cigarettes (20 Years)
Pancreatic cancer	Female	15-19 years	95+ years	1	Cumulative Cigarettes (5 Years)
Pancreatic cancer	Female	15-19 years	95+ years	1	Diabetes Age-Standardized Prevalence (proportion)
Pancreatic cancer	Female	15-19 years	95+ years	-1	Education (years per capita)
Pancreatic cancer	Female	15-19 years	95+ years	0	LDI (I$ per capita)
Pancreatic cancer	Female	15-19 years	95+ years	1	Mean BMI
Pancreatic cancer	Female	15-19 years	95+ years	1	Percent of total calories consumed as saturated fat
Pancreatic cancer	Female	15-19 years	95+ years	1	Smoking Prevalence
Pancreatic cancer	Female	15-19 years	95+ years	1	Log-transformed SEV scalar: Pancreas C
Pancreatic cancer	Female	15-19 years	95+ years	0	Socio-demographic Index
Pancreatic cancer	Female	15-19 years	95+ years	-1	fruits adjusted(g)
Pancreatic cancer	Female	15-19 years	95+ years	1	red meats adjusted(g)
Pancreatic cancer	Female	15-19 years	95+ years	-1	vegetables adjusted(g)
Pancreatic cancer	Female	15-19 years	95+ years	1	energy unadjusted(kcal)
Pancreatic cancer	Female	15-19 years	95+ years	-1	Healthcare access and quality index
Pancreatic cancer	Male	15-19 years	95+ years	1	energy unadjusted(kcal)
Pancreatic cancer	Female	15-19 years	95+ years	1	vegetables adjusted(g)
Pancreatic cancer	Female	15-19 years	95+ years	-1	vegetables unadjusted(g)
Malignant skin melanoma	Male	15-19 years	95+ years	1	Alcohol (liters per capita)
Malignant skin melanoma	Male	15-19 years	95+ years	-1	Education (years per capita)
Malignant skin melanoma	Male	15-19 years	95+ years	0	LDI (I$ per capita)
Malignant skin melanoma	Male	15-19 years	95+ years	0	Latitude Under 15 (proportion)
Malignant skin melanoma	Male	15-19 years	95+ years	0	Latitude 15 to 30 (proportion)
Malignant skin melanoma	Male	15-19 years	95+ years	-1	Latitude 30 to 45 (proportion)
Malignant skin melanoma	Male	15-19 years	95+ years	-1	Latitude Over 45 (proportion)
Malignant skin melanoma	Male	15-19 years	95+ years	0	Socio-demographic Index
Malignant skin melanoma	Male	15-19 years	95+ years	-1	fruits adjusted(g)
Malignant skin melanoma	Male	15-19 years	95+ years	-1	vegetables adjusted(g)
Malignant skin melanoma	Male	15-19 years	95+ years	-1	Healthcare access and quality index
Malignant skin melanoma	Female	15-19 years	95+ years	1	Alcohol (liters per capita)
Malignant skin melanoma	Female	15-19 years	95+ years	-1	Education (years per capita)

Cause	Sex	Age start	Age end	Direction	Covariate
Malignant skin melanoma	Female	15-19 years	95+ years	0	LDI (I$ per capita)
Malignant skin melanoma	Female	15-19 years	95+ years	0	Latitude Under 15 (proportion)
Malignant skin melanoma	Female	15-19 years	95+ years	0	Latitude 15 to 30 (proportion)
Malignant skin melanoma	Female	15-19 years	95+ years	-1	Latitude 30 to 45 (proportion)
Malignant skin melanoma	Female	15-19 years	95+ years	-1	Latitude Over 45 (proportion)
Malignant skin melanoma	Female	15-19 years	95+ years	0	Socio-demographic Index
Malignant skin melanoma	Female	15-19 years	95+ years	-1	fruits adjusted(g)
Malignant skin melanoma	Female	15-19 years	95+ years	-1	vegetables adjusted(g)
Malignant skin melanoma	Female	15-19 years	95+ years	-1	Healthcare access and quality index
Non-melanoma skin cancer	Male	15-19 years	95+ years	1	Cumulative Cigarettes (10 Years)
Non-melanoma skin cancer	Male	15-19 years	95+ years	1	Cumulative Cigarettes (15 Years)
Non-melanoma skin cancer	Male	15-19 years	95+ years	1	Cumulative Cigarettes (5 Years)
Non-melanoma skin cancer	Male	15-19 years	95+ years	-1	Education (years per capita)
Non-melanoma skin cancer	Male	15-19 years	95+ years	0	Average latitude
Non-melanoma skin cancer	Male	15-19 years	95+ years	0	LDI (I$ per capita)
Non-melanoma skin cancer	Male	15-19 years	95+ years	1	Smoking Prevalence
Non-melanoma skin cancer	Male	15-19 years	95+ years	0	Socio-demographic Index
Non-melanoma skin cancer	Male	15-19 years	95+ years	-1	Healthcare access and quality index
Non-melanoma skin cancer	Male	15-19 years	95+ years	-1	Health System Access (capped)
Non-melanoma skin cancer	Female	15-19 years	95+ years	1	Cumulative Cigarettes (10 Years)
Non-melanoma skin cancer	Female	15-19 years	95+ years	1	Cumulative Cigarettes (15 Years)
Non-melanoma skin cancer	Female	15-19 years	95+ years	1	Cumulative Cigarettes (5 Years)
Non-melanoma skin cancer	Female	15-19 years	95+ years	-1	Education (years per capita)
Non-melanoma skin cancer	Female	15-19 years	95+ years	0	Average latitude
Non-melanoma skin cancer	Female	15-19 years	95+ years	0	LDI (I$ per capita)
Non-melanoma skin cancer	Female	15-19 years	95+ years	1	Smoking Prevalence
Non-melanoma skin cancer	Female	15-19 years	95+ years	0	Socio-demographic Index
Non-melanoma skin cancer	Female	15-19 years	95+ years	-1	Healthcare access and quality index
Non-melanoma skin cancer	Female	15-19 years	95+ years	-1	Health System Access (capped)
Ovarian cancer	Female	15-19 years	95+ years	1	Alcohol (liters per capita)
Ovarian cancer	Female	15-19 years	95+ years	1	Tobacco (cigarettes per capita)
Ovarian cancer	Female	15-19 years	95+ years	-1	Contraception (Modern) Prevalence (proportion)
Ovarian cancer	Female	15-19 years	95+ years	1	Cumulative Cigarettes (20 Years)
Ovarian cancer	Female	15-19 years	95+ years	1	Diabetes Age-Standardized Prevalence (proportion)
Ovarian cancer	Female	15-19 years	95+ years	-1	Education (years per capita)
Ovarian cancer	Female	15-19 years	95+ years	0	LDI (I$ per capita)
Ovarian cancer	Female	15-19 years	95+ years	1	Mean BMI
Ovarian cancer	Female	15-19 years	95+ years	1	Percent of total calories consumed as saturated fat
Ovarian cancer	Female	15-19 years	95+ years	1	Smoking Prevalence
Ovarian cancer	Female	15-19 years	95+ years	0	Total Fertility Rate
Ovarian cancer	Female	15-19 years	95+ years	1	Log-transformed SEV scalar: Ovary C
Ovarian cancer	Female	15-19 years	95+ years	0	Socio-demographic Index
Ovarian cancer	Female	15-19 years	95+ years	-1	fruits adjusted(g)
Ovarian cancer	Female	15-19 years	95+ years	-1	vegetables adjusted(g)
Ovarian cancer	Female	15-19 years	95+ years	1	energy unadjusted(kcal)
Ovarian cancer	Female	15-19 years	95+ years	-1	Healthcare access and quality index
Testicular cancer	Male	15-19 years	95+ years	1	Cumulative Cigarettes (10 Years)
Testicular cancer	Male	15-19 years	95+ years	1	Cumulative Cigarettes (15 Years)
Testicular cancer	Male	15-19 years	95+ years	1	Cumulative Cigarettes (5 Years)
Testicular cancer	Male	15-19 years	95+ years	-1	Education (years per capita)
Testicular cancer	Male	15-19 years	95+ years	-1	Fruits (kcal per capita)
Testicular cancer	Male	15-19 years	95+ years	-1	Health System Access 2 (unitless)
Testicular cancer	Male	15-19 years	95+ years	-1	LDI (I$ per capita)
Testicular cancer	Male	15-19 years	95+ years	-1	Vegetables (kcal per capita)
Testicular cancer	Male	15-19 years	95+ years	0	Socio-demographic Index
Testicular cancer	Male	15-19 years	95+ years	-1	Healthcare access and quality index
Kidney cancer	Male	0-6 days	95+ years	1	Alcohol (liters per capita)
Kidney cancer	Male	0-6 days	95+ years	1	Cumulative Cigarettes (10 Years)
Kidney cancer	Male	0-6 days	95+ years	1	Cumulative Cigarettes (15 Years)
Kidney cancer	Male	0-6 days	95+ years	1	Cumulative Cigarettes (5 Years)
Kidney cancer	Male	0-6 days	95+ years	1	Diabetes Age-Standardized Prevalence (proportion)
Kidney cancer	Male	0-6 days	95+ years	-1	Education (years per capita)
Kidney cancer	Male	0-6 days	95+ years	-1	Health System Access 2 (unitless)
Kidney cancer	Male	0-6 days	95+ years	0	LDI (I$ per capita)
Kidney cancer	Male	0-6 days	95+ years	1	Mean BMI
Kidney cancer	Male	0-6 days	95+ years	1	Systolic Blood Pressure (mmHg)
Kidney cancer	Male	0-6 days	95+ years	1	Smoking Prevalence
Kidney cancer	Male	0-6 days	95+ years	1	Log-transformed SEV scalar: Kidney C
Kidney cancer	Male	0-6 days	95+ years	0	Socio-demographic Index
Kidney cancer	Female	0-6 days	95+ years	1	Alcohol (liters per capita)
Kidney cancer	Female	0-6 days	95+ years	1	Cumulative Cigarettes (10 Years)
Kidney cancer	Female	0-6 days	95+ years	1	Cumulative Cigarettes (15 Years)
Kidney cancer	Female	0-6 days	95+ years	1	Cumulative Cigarettes (5 Years)
Kidney cancer	Female	0-6 days	95+ years	1	Diabetes Age-Standardized Prevalence (proportion)
Kidney cancer	Female	0-6 days	95+ years	-1	Education (years per capita)
Kidney cancer	Female	0-6 days	95+ years	-1	Health System Access 2 (unitless)
Kidney cancer	Female	0-6 days	95+ years	-1	LDI (I$ per capita)
Kidney cancer	Female	0-6 days	95+ years	1	Mean BMI
Kidney cancer	Female	0-6 days	95+ years	1	Systolic Blood Pressure (mmHg)
Kidney cancer	Female	0-6 days	95+ years	1	Smoking Prevalence
Kidney cancer	Female	0-6 days	95+ years	0	Total Fertility Rate
Kidney cancer	Female	0-6 days	95+ years	1	Total Calories (kcal per capita)
Kidney cancer	Female	0-6 days	95+ years	1	Log-transformed SEV scalar: Kidney C
Kidney cancer	Female	0-6 days	95+ years	1	Socio-demographic Index
Kidney cancer	Female	0-6 days	95+ years	0	LDI (I$ per capita)
Kidney cancer	Female	0-6 days	95+ years	0	Socio-demographic Index
Bladder cancer	Male	15-19 years	95+ years	1	Alcohol (liters per capita)
Bladder cancer	Male	15-19 years	95+ years	1	Cumulative Cigarettes (10 Years)
Bladder cancer	Male	15-19 years	95+ years	1	Cumulative Cigarettes (15 Years)
Bladder cancer	Male	15-19 years	95+ years	1	Cumulative Cigarettes (5 Years)
Bladder cancer	Male	15-19 years	95+ years	-1	Education (years per capita)
Bladder cancer	Male	15-19 years	95+ years	0	LDI (I$ per capita)
Bladder cancer	Male	15-19 years	95+ years	1	Population Density (over 1000 ppl/sqkm, proportion)
Bladder cancer	Male	15-19 years	95+ years	1	Population Density (under 150 ppl/sqkm, proportion)
Bladder cancer	Male	15-19 years	95+ years	1	Smoking Prevalence

Cause	Sex	Age start	Age end	Direction	Covariate
Bladder cancer	Male	15-19 years	95+ years	1	Log-transformed SEV scalar: Bladder C
Bladder cancer	Male	15-19 years	95+ years	0	Socio-demographic Index
Bladder cancer	Male	15-19 years	95+ years	-1	fruits adjusted(g)
Bladder cancer	Male	15-19 years	95+ years	-1	vegetables adjusted(g)
Bladder cancer	Male	15-19 years	95+ years	-1	Healthcare access and quality index
Bladder cancer	Female	15-19 years	95+ years	1	Alcohol (liters per capita)
Bladder cancer	Female	15-19 years	95+ years	1	Cumulative Cigarettes (10 Years)
Bladder cancer	Female	15-19 years	95+ years	1	Cumulative Cigarettes (15 Years)
Bladder cancer	Female	15-19 years	95+ years	1	Cumulative Cigarettes (5 Years)
Bladder cancer	Female	15-19 years	95+ years	-1	Education (years per capita)
Bladder cancer	Female	15-19 years	95+ years	0	LDI (I$ per capita)
Bladder cancer	Female	15-19 years	95+ years	1	Population Density (over 1000 ppl/sqkm, proportion)
Bladder cancer	Female	15-19 years	95+ years	1	Population Density (under 150 ppl/sqkm, proportion)
Bladder cancer	Female	15-19 years	95+ years	1	Smoking Prevalence
Bladder cancer	Female	15-19 years	95+ years	1	Log-transformed SEV scalar: Bladder C
Bladder cancer	Female	15-19 years	95+ years	0	Socio-demographic Index
Bladder cancer	Female	15-19 years	95+ years	-1	fruits adjusted(g)
Bladder cancer	Female	15-19 years	95+ years	-1	vegetables adjusted(g)
Bladder cancer	Female	15-19 years	95+ years	-1	Healthcare access and quality index
Brain and nervous system cancer	Female	0-6 days	95+ years	1	Alcohol (liters per capita)
Brain and nervous system cancer	Female	0-6 days	95+ years	1	Cumulative Cigarettes (10 Years)
Brain and nervous system cancer	Female	0-6 days	95+ years	1	Cumulative Cigarettes (15 Years)
Brain and nervous system cancer	Female	0-6 days	95+ years	-1	Education (years per capita)
Brain and nervous system cancer	Female	0-6 days	95+ years	0	LDI (I$ per capita)
Brain and nervous system cancer	Female	0-6 days	95+ years	1	Cholesterol (total, mean per capita)
Brain and nervous system cancer	Female	0-6 days	95+ years	1	Systolic Blood Pressure (mmHg)
Brain and nervous system cancer	Female	0-6 days	95+ years	1	Percent of total calories consumed as saturated fat
Brain and nervous system cancer	Female	0-6 days	95+ years	1	Smoking Prevalence
Brain and nervous system cancer	Female	0-6 days	95+ years	0	Socio-demographic Index
Brain and nervous system cancer	Female	0-6 days	95+ years	-1	fruits adjusted(g)
Brain and nervous system cancer	Female	0-6 days	95+ years	1	red meats adjusted(g)
Brain and nervous system cancer	Female	0-6 days	95+ years	-1	vegetables adjusted(g)
Brain and nervous system cancer	Female	0-6 days	95+ years	-1	Healthcare access and quality index
Brain and nervous system cancer	Male	0-6 days	95+ years	1	Alcohol (liters per capita)
Brain and nervous system cancer	Male	0-6 days	95+ years	1	Cumulative Cigarettes (10 Years)
Brain and nervous system cancer	Male	0-6 days	95+ years	1	Cumulative Cigarettes (15 Years)
Brain and nervous system cancer	Male	0-6 days	95+ years	-1	Education (years per capita)
Brain and nervous system cancer	Male	0-6 days	95+ years	0	LDI (I$ per capita)
Brain and nervous system cancer	Male	0-6 days	95+ years	1	Cholesterol (total, mean per capita)
Brain and nervous system cancer	Male	0-6 days	95+ years	1	Systolic Blood Pressure (mmHg)
Brain and nervous system cancer	Male	0-6 days	95+ years	1	Percent of total calories consumed as saturated fat
Brain and nervous system cancer	Male	0-6 days	95+ years	1	Smoking Prevalence
Brain and nervous system cancer	Male	0-6 days	95+ years	-1	Vegetables (kcal per capita)
Brain and nervous system cancer	Male	0-6 days	95+ years	0	Socio-demographic Index
Brain and nervous system cancer	Male	0-6 days	95+ years	-1	fruits adjusted(g)
Brain and nervous system cancer	Male	0-6 days	95+ years	1	red meats adjusted(g)
Brain and nervous system cancer	Male	0-6 days	95+ years	-1	vegetables adjusted(g)
Brain and nervous system cancer	Male	0-6 days	95+ years	-1	Healthcare access and quality index
Thyroid cancer	Female	10-14 years	95+ years	1	Alcohol (liters per capita)
Thyroid cancer	Female	10-14 years	95+ years	1	Tobacco (cigarettes per capita)
Thyroid cancer	Female	10-14 years	95+ years	-1	Education (years per capita)
Thyroid cancer	Female	10-14 years	95+ years	0	LDI (I$ per capita)
Thyroid cancer	Female	10-14 years	95+ years	1	Mean BMI
Thyroid cancer	Female	10-14 years	95+ years	-1	Sanitation (proportion with access)
Thyroid cancer	Female	10-14 years	95+ years	1	Smoking Prevalence
Thyroid cancer	Female	10-14 years	95+ years	-1	Improved Water Source (proportion with access)
Thyroid cancer	Female	10-14 years	95+ years	1	Log-transformed SEV scalar: Thyroid C
Thyroid cancer	Female	10-14 years	95+ years	0	Socio-demographic Index
Thyroid cancer	Female	10-14 years	95+ years	-1	fruits adjusted(g)
Thyroid cancer	Female	10-14 years	95+ years	1	red meats adjusted(g)
Thyroid cancer	Female	10-14 years	95+ years	-1	vegetables adjusted(g)
Thyroid cancer	Female	10-14 years	95+ years	-1	Healthcare access and quality index
Thyroid cancer	Male	10-14 years	95+ years	1	Alcohol (liters per capita)
Thyroid cancer	Male	10-14 years	95+ years	1	Tobacco (cigarettes per capita)
Thyroid cancer	Male	10-14 years	95+ years	-1	Education (years per capita)
Thyroid cancer	Male	10-14 years	95+ years	0	LDI (I$ per capita)
Thyroid cancer	Male	10-14 years	95+ years	1	Mean BMI
Thyroid cancer	Male	10-14 years	95+ years	-1	Sanitation (proportion with access)
Thyroid cancer	Male	10-14 years	95+ years	1	Smoking Prevalence
Thyroid cancer	Male	10-14 years	95+ years	-1	Improved Water Source (proportion with access)
Thyroid cancer	Male	10-14 years	95+ years	1	Log-transformed SEV scalar: Thyroid C
Thyroid cancer	Male	10-14 years	95+ years	0	Socio-demographic Index
Thyroid cancer	Male	10-14 years	95+ years	-1	fruits adjusted(g)
Thyroid cancer	Male	10-14 years	95+ years	1	red meats adjusted(g)
Thyroid cancer	Male	10-14 years	95+ years	-1	vegetables adjusted(g)
Thyroid cancer	Male	10-14 years	95+ years	-1	Healthcare access and quality index
Thyroid cancer	Male	10-14 years	95+ years	2	Smoking Prevalence
Mesothelioma	Female	15-19 years	95+ years	1	Asbestos production (binary)
Mesothelioma	Female	15-19 years	95+ years	1	Asbestos production (kg) per capita
Mesothelioma	Female	15-19 years	95+ years	1	Cumulative Cigarettes (5 Years)
Mesothelioma	Female	15-19 years	95+ years	-1	Education (years per capita)
Mesothelioma	Female	15-19 years	95+ years	1	Gold production (binary)
Mesothelioma	Female	15-19 years	95+ years	1	Gold production (kg) per capita
Mesothelioma	Female	15-19 years	95+ years	0	LDI (I$ per capita)
Mesothelioma	Female	15-19 years	95+ years	1	Indoor Air Pollution (All Cooking Fuels)
Mesothelioma	Female	15-19 years	95+ years	1	Elevation Over 1500m (proportion)
Mesothelioma	Female	15-19 years	95+ years	1	Elevation 500 to 1500m (proportion)
Mesothelioma	Female	15-19 years	95+ years	1	Population Density (over 1000 ppl/sqkm, proportion)
Mesothelioma	Female	15-19 years	95+ years	1	Population Over 65 (proportion)
Mesothelioma	Female	15-19 years	95+ years	1	Smoking Prevalence
Mesothelioma	Female	15-19 years	95+ years	1	Log-transformed SEV scalar: Mesothel
Mesothelioma	Female	15-19 years	95+ years	0	Socio-demographic Index
Mesothelioma	Female	15-19 years	95+ years	1	Asbestos consumption (metric tons per year per capita)
Mesothelioma	Female	15-19 years	95+ years	-1	Healthcare access and quality index
Mesothelioma	Male	15-19 years	95+ years	1	Cumulative Cigarettes (5 Years)

Cause	Sex	Age start	Age end	Direction	Covariate
Mesothelioma	Male	15-19 years	95+ years	-1	Education (years per capita)
Mesothelioma	Male	15-19 years	95+ years	1	Gold production (binary)
Mesothelioma	Male	15-19 years	95+ years	1	Gold production (kg) per capita
Mesothelioma	Male	15-19 years	95+ years	0	LDI (I$ per capita)
Mesothelioma	Male	15-19 years	95+ years	1	Indoor Air Pollution (All Cooking Fuels)
Mesothelioma	Male	15-19 years	95+ years	1	Elevation Over 1500m (proportion)
Mesothelioma	Male	15-19 years	95+ years	1	Elevation 500 to 1500m (proportion)
Mesothelioma	Male	15-19 years	95+ years	1	Population Density (over 1000 ppl/sqkm, proportion)
Mesothelioma	Male	15-19 years	95+ years	1	Population Over 65 (proportion)
Mesothelioma	Male	15-19 years	95+ years	1	Smoking Prevalence
Mesothelioma	Male	15-19 years	95+ years	0	Socio-demographic Index
Mesothelioma	Male	15-19 years	95+ years	1	Asbestos consumption (metric tons per year per capita)
Mesothelioma	Male	15-19 years	95+ years	-1	Healthcare access and quality index
Hodgkin lymphoma	Male	0-6 days	95+ years	-1	Education (years per capita)
Hodgkin lymphoma	Male	0-6 days	95+ years	0	LDI (I$ per capita)
Hodgkin lymphoma	Male	0-6 days	95+ years	0	Socio-demographic Index
Hodgkin lymphoma	Male	0-6 days	95+ years	-1	Healthcare access and quality index
Hodgkin lymphoma	Female	0-6 days	95+ years	-1	Education (years per capita)
Hodgkin lymphoma	Female	0-6 days	95+ years	0	LDI (I$ per capita)
Hodgkin lymphoma	Female	0-6 days	95+ years	0	Socio-demographic Index
Hodgkin lymphoma	Female	0-6 days	95+ years	-1	Healthcare access and quality index
Non-Hodgkin lymphoma	Male	0-6 days	95+ years	1	Alcohol (liters per capita)
Non-Hodgkin lymphoma	Male	0-6 days	95+ years	1	Cumulative Cigarettes (10 Years)
Non-Hodgkin lymphoma	Male	0-6 days	95+ years	-1	Health System Access 2 (unitless)
Non-Hodgkin lymphoma	Male	0-6 days	95+ years	0	LDI (I$ per capita)
Non-Hodgkin lymphoma	Male	0-6 days	95+ years	1	Smoking Prevalence
Non-Hodgkin lymphoma	Male	0-6 days	95+ years	0	Socio-demographic Index
Non-Hodgkin lymphoma	Male	0-6 days	95+ years	-1	Health System Access (capped)
Non-Hodgkin lymphoma	Male	0-6 days	95+ years	-1	Healthcare access and quality index
Non-Hodgkin lymphoma	Female	0-6 days	95+ years	1	Alcohol (liters per capita)
Non-Hodgkin lymphoma	Female	0-6 days	95+ years	1	Cumulative Cigarettes (10 Years)
Non-Hodgkin lymphoma	Female	0-6 days	95+ years	-1	Health System Access 2 (unitless)
Non-Hodgkin lymphoma	Female	0-6 days	95+ years	0	LDI (I$ per capita)
Non-Hodgkin lymphoma	Female	0-6 days	95+ years	1	Smoking Prevalence
Non-Hodgkin lymphoma	Female	0-6 days	95+ years	0	Socio-demographic Index
Non-Hodgkin lymphoma	Female	0-6 days	95+ years	-1	Healthcare access and quality index
Non-Hodgkin lymphoma	Female	0-6 days	95+ years	0	Total Fertility Rate
Multiple myeloma	Male	15-19 years	95+ years	1	Alcohol (liters per capita)
Multiple myeloma	Male	15-19 years	95+ years	1	Tobacco (cigarettes per capita)
Multiple myeloma	Male	15-19 years	95+ years	-1	Education (years per capita)
Multiple myeloma	Male	15-19 years	95+ years	0	LDI (I$ per capita)
Multiple myeloma	Male	15-19 years	95+ years	1	Mean BMI
Multiple myeloma	Male	15-19 years	95+ years	-1	Sanitation (proportion with access)
Multiple myeloma	Male	15-19 years	95+ years	1	Smoking Prevalence
Multiple myeloma	Male	15-19 years	95+ years	-1	Improved Water Source (proportion with access)
Multiple myeloma	Male	15-19 years	95+ years	0	Socio-demographic Index
Multiple myeloma	Male	15-19 years	95+ years	-1	fruits adjusted(g)
Multiple myeloma	Male	15-19 years	95+ years	1	red meats adjusted(g)
Multiple myeloma	Male	15-19 years	95+ years	-1	vegetables adjusted(g)
Multiple myeloma	Male	15-19 years	95+ years	-1	Healthcare access and quality index
Multiple myeloma	Female	15-19 years	95+ years	1	Alcohol (liters per capita)
Multiple myeloma	Female	15-19 years	95+ years	1	Tobacco (cigarettes per capita)
Multiple myeloma	Female	15-19 years	95+ years	-1	Education (years per capita)
Multiple myeloma	Female	15-19 years	95+ years	0	LDI (I$ per capita)
Multiple myeloma	Female	15-19 years	95+ years	1	Mean BMI
Multiple myeloma	Female	15-19 years	95+ years	-1	Sanitation (proportion with access)
Multiple myeloma	Female	15-19 years	95+ years	1	Smoking Prevalence
Multiple myeloma	Female	15-19 years	95+ years	-1	Improved Water Source (proportion with access)
Multiple myeloma	Female	15-19 years	95+ years	0	Socio-demographic Index
Multiple myeloma	Female	15-19 years	95+ years	-1	fruits adjusted(g)
Multiple myeloma	Female	15-19 years	95+ years	1	red meats adjusted(g)
Multiple myeloma	Female	15-19 years	95+ years	-1	vegetables adjusted(g)
Multiple myeloma	Female	15-19 years	95+ years	-1	Healthcare access and quality index
Leukemia	Female	0-6 days	95+ years	1	Alcohol (liters per capita)
Leukemia	Female	0-6 days	95+ years	1	Tobacco (cigarettes per capita)
Leukemia	Female	0-6 days	95+ years	1	Cumulative Cigarettes (10 Years)
Leukemia	Female	0-6 days	95+ years	1	Cumulative Cigarettes (15 Years)
Leukemia	Female	0-6 days	95+ years	1	Cumulative Cigarettes (20 Years)
Leukemia	Female	0-6 days	95+ years	1	Cumulative Cigarettes (5 Years)
Leukemia	Female	0-6 days	95+ years	-1	Education (years per capita)
Leukemia	Female	0-6 days	95+ years	-1	Health System Access 2 (unitless)
Leukemia	Female	0-6 days	95+ years	0	LDI (I$ per capita)
Leukemia	Female	0-6 days	95+ years	1	Smoking Prevalence
Leukemia	Female	0-6 days	95+ years	1	Log-transformed SEV scalar: Leukemia
Leukemia	Female	0-6 days	95+ years	1	Log-transformed age-standardized SEV scalar: Leukemia
Leukemia	Female	0-6 days	95+ years	0	Socio-demographic Index
Leukemia	Male	0-6 days	95+ years	1	Alcohol (liters per capita)
Leukemia	Male	0-6 days	95+ years	1	Tobacco (cigarettes per capita)
Leukemia	Male	0-6 days	95+ years	1	Cumulative Cigarettes (10 Years)
Leukemia	Male	0-6 days	95+ years	1	Cumulative Cigarettes (15 Years)
Leukemia	Male	0-6 days	95+ years	1	Cumulative Cigarettes (20 Years)
Leukemia	Male	0-6 days	95+ years	1	Cumulative Cigarettes (5 Years)
Leukemia	Male	0-6 days	95+ years	-1	Education (years per capita)
Leukemia	Male	0-6 days	95+ years	-1	Health System Access 2 (unitless)
Leukemia	Male	0-6 days	95+ years	0	LDI (I$ per capita)
Leukemia	Male	0-6 days	95+ years	1	Smoking Prevalence
Leukemia	Male	0-6 days	95+ years	1	Log-transformed SEV scalar: Leukemia
Leukemia	Male	0-6 days	95+ years	1	Log-transformed age-standardized SEV scalar: Leukemia
Leukemia	Male	0-6 days	95+ years	0	Socio-demographic Index
Leukemia	Female	0-6 days	95+ years	-1	Health System Access (capped)
Leukemia	Female	0-6 days	95+ years	-1	Healthcare access and quality index
Other neoplasms	Male	0-6 days	95+ years	1	Tobacco (cigarettes per capita)
Other neoplasms	Male	0-6 days	95+ years	-1	Education (years per capita)
Other neoplasms	Male	0-6 days	95+ years	-1	Health System Access 2 (unitless)
Other neoplasms	Male	0-6 days	95+ years	0	LDI (I$ per capita)

Cause	Sex	Age start	Age end	Direction	Covariate
Other neoplasms	Male	0-6 days	95+ years	-1	Nuts & Seeds (kcal per capita)
Other neoplasms	Male	0-6 days	95+ years	1	Smoking Prevalence
Other neoplasms	Male	0-6 days	95+ years	0	Socio-demographic Index
Other neoplasms	Male	0-6 days	95+ years	-1	fruits adjusted(g)
Other neoplasms	Male	0-6 days	95+ years	-1	pufa adjusted(percent)
Other neoplasms	Male	0-6 days	95+ years	-1	vegetables adjusted(g)
Other neoplasms	Male	0-6 days	95+ years	-1	Healthcare access and quality index
Other neoplasms	Female	0-6 days	95+ years	1	Tobacco (cigarettes per capita)
Other neoplasms	Female	0-6 days	95+ years	-1	Education (years per capita)
Other neoplasms	Female	0-6 days	95+ years	-1	Health System Access 2 (unitless)
Other neoplasms	Female	0-6 days	95+ years	0	LDI (I$ per capita)
Other neoplasms	Female	0-6 days	95+ years	1	Smoking Prevalence
Other neoplasms	Female	0-6 days	95+ years	0	Socio-demographic Index
Other neoplasms	Female	0-6 days	95+ years	-1	fruits adjusted(g)
Other neoplasms	Female	0-6 days	95+ years	-1	nuts seeds adjusted(g)
Other neoplasms	Female	0-6 days	95+ years	-1	pufa adjusted(percent)
Other neoplasms	Female	0-6 days	95+ years	-1	vegetables adjusted(g)
Other neoplasms	Female	0-6 days	95+ years	-1	Healthcare access and quality index
Other neoplasms	Male	0-6 days	95+ years	-1	nuts seeds adjusted(g)
Cardiovascular diseases	Female	0-6 days	95+ years	0	Alcohol (liters per capita)
Cardiovascular diseases	Female	0-6 days	95+ years	1	Diabetes Fasting Plasma Glucose (mmol/L)
Cardiovascular diseases	Female	0-6 days	95+ years	-1	LDI (I$ per capita)
Cardiovascular diseases	Female	0-6 days	95+ years	1	Mean BMI
Cardiovascular diseases	Female	0-6 days	95+ years	1	Cholesterol (total, mean per capita)
Cardiovascular diseases	Female	0-6 days	95+ years	1	Systolic Blood Pressure (mmHg)
Cardiovascular diseases	Female	0-6 days	95+ years	1	Indoor Air Pollution (All Cooking Fuels)
Cardiovascular diseases	Female	0-6 days	95+ years	1	Outdoor Air Pollution (PM2.5)
Cardiovascular diseases	Female	0-6 days	95+ years	-1	Elevation Over 1500m (proportion)
Cardiovascular diseases	Female	0-6 days	95+ years	1	Smoking Prevalence
Cardiovascular diseases	Female	0-6 days	95+ years	0	Socio-demographic Index
Cardiovascular diseases	Female	0-6 days	95+ years	-1	omega 3 adjusted(g)
Cardiovascular diseases	Female	0-6 days	95+ years	-1	fruits adjusted(g)
Cardiovascular diseases	Female	0-6 days	95+ years	-1	nuts seeds adjusted(g)
Cardiovascular diseases	Female	0-6 days	95+ years	-1	pufa adjusted(percent)
Cardiovascular diseases	Female	0-6 days	95+ years	-1	pulses legumes adjusted(g)
Cardiovascular diseases	Female	0-6 days	95+ years	-1	vegetables adjusted(g)
Cardiovascular diseases	Female	0-6 days	95+ years	-1	whole grains adjusted(g)
Cardiovascular diseases	Female	0-6 days	95+ years	1	Log-transformed SEV scalar: CVD
Cardiovascular diseases	Female	0-6 days	95+ years	-1	Healthcare access and quality index
Cardiovascular diseases	Female	0-6 days	95+ years	1	Diet high in trans fatty acids
Cardiovascular diseases	Male	0-6 days	95+ years	0	Alcohol (liters per capita)
Cardiovascular diseases	Male	0-6 days	95+ years	1	Diabetes Fasting Plasma Glucose (mmol/L)
Cardiovascular diseases	Male	0-6 days	95+ years	-1	LDI (I$ per capita)
Cardiovascular diseases	Male	0-6 days	95+ years	1	Mean BMI
Cardiovascular diseases	Male	0-6 days	95+ years	1	Cholesterol (total, mean per capita)
Cardiovascular diseases	Male	0-6 days	95+ years	1	Systolic Blood Pressure (mmHg)
Cardiovascular diseases	Male	0-6 days	95+ years	1	Indoor Air Pollution (All Cooking Fuels)
Cardiovascular diseases	Male	0-6 days	95+ years	1	Outdoor Air Pollution (PM2.5)
Cardiovascular diseases	Male	0-6 days	95+ years	-1	Elevation Over 1500m (proportion)
Cardiovascular diseases	Male	0-6 days	95+ years	1	Smoking Prevalence
Cardiovascular diseases	Male	0-6 days	95+ years	0	Socio-demographic Index
Cardiovascular diseases	Male	0-6 days	95+ years	-1	omega 3 adjusted(g)
Cardiovascular diseases	Male	0-6 days	95+ years	-1	fruits adjusted(g)
Cardiovascular diseases	Male	0-6 days	95+ years	-1	nuts seeds adjusted(g)
Cardiovascular diseases	Male	0-6 days	95+ years	-1	pufa adjusted(percent)
Cardiovascular diseases	Male	0-6 days	95+ years	-1	pulses legumes adjusted(g)
Cardiovascular diseases	Male	0-6 days	95+ years	-1	vegetables adjusted(g)
Cardiovascular diseases	Male	0-6 days	95+ years	-1	whole grains adjusted(g)
Cardiovascular diseases	Male	0-6 days	95+ years	1	Log-transformed SEV scalar: CVD
Cardiovascular diseases	Male	0-6 days	95+ years	-1	Healthcare access and quality index
Cardiovascular diseases	Male	0-6 days	95+ years	1	Diet high in trans fatty acids
Rheumatic heart disease	Male	1-4 years	95+ years	-1	Education (years per capita)
Rheumatic heart disease	Male	1-4 years	95+ years	-1	LDI (I$ per capita)
Rheumatic heart disease	Male	1-4 years	95+ years	1	Underweight (proportion <2SD weight for age, <5 years)
Rheumatic heart disease	Male	1-4 years	95+ years	-1	Sanitation (proportion with access)
Rheumatic heart disease	Male	1-4 years	95+ years	-1	Improved Water Source (proportion with access)
Rheumatic heart disease	Male	1-4 years	95+ years	1	Log-transformed SEV scalar: RHD
Rheumatic heart disease	Male	1-4 years	95+ years	-1	Socio-demographic Index
Rheumatic heart disease	Male	1-4 years	95+ years	-1	Healthcare access and quality index
Rheumatic heart disease	Female	1-4 years	95+ years	-1	Education (years per capita)
Rheumatic heart disease	Female	1-4 years	95+ years	-1	LDI (I$ per capita)
Rheumatic heart disease	Female	1-4 years	95+ years	1	Underweight (proportion <2SD weight for age, <5 years)
Rheumatic heart disease	Female	1-4 years	95+ years	-1	Sanitation (proportion with access)
Rheumatic heart disease	Female	1-4 years	95+ years	-1	Improved Water Source (proportion with access)
Rheumatic heart disease	Female	1-4 years	95+ years	1	Log-transformed SEV scalar: RHD
Rheumatic heart disease	Female	1-4 years	95+ years	-1	Socio-demographic Index
Rheumatic heart disease	Female	1-4 years	95+ years	-1	Healthcare access and quality index
Ischemic heart disease	Male	28-364 days	95+ years	0	Alcohol (liters per capita)
Ischemic heart disease	Male	28-364 days	95+ years	1	Diabetes Fasting Plasma Glucose (mmol/L)
Ischemic heart disease	Male	28-364 days	95+ years	-1	LDI (I$ per capita)
Ischemic heart disease	Male	28-364 days	95+ years	1	Mean BMI
Ischemic heart disease	Male	28-364 days	95+ years	1	Cholesterol (total, mean per capita)
Ischemic heart disease	Male	28-364 days	95+ years	1	Systolic Blood Pressure (mmHg)
Ischemic heart disease	Male	28-364 days	95+ years	1	Indoor Air Pollution (All Cooking Fuels)
Ischemic heart disease	Male	28-364 days	95+ years	1	Outdoor Air Pollution (PM2.5)
Ischemic heart disease	Male	28-364 days	95+ years	-1	Elevation Over 1500m (proportion)
Ischemic heart disease	Male	28-364 days	95+ years	1	Smoking Prevalence
Ischemic heart disease	Male	28-364 days	95+ years	1	Log-transformed SEV scalar: IHD
Ischemic heart disease	Male	28-364 days	95+ years	0	Socio-demographic Index
Ischemic heart disease	Male	28-364 days	95+ years	-1	omega 3 adjusted(g)
Ischemic heart disease	Male	28-364 days	95+ years	-1	fruits adjusted(g)
Ischemic heart disease	Male	28-364 days	95+ years	-1	nuts seeds adjusted(g)
Ischemic heart disease	Male	28-364 days	95+ years	-1	pufa adjusted(percent)
Ischemic heart disease	Male	28-364 days	95+ years	-1	pulses legumes adjusted(g)
Ischemic heart disease	Male	28-364 days	95+ years	-1	vegetables adjusted(g)

Cause	Sex	Age start	Age end	Direction	Covariate
Ischemic heart disease	Male	28-364 days	95+ years	-1	whole grains adjusted(g)
Ischemic heart disease	Male	28-364 days	95+ years	-1	Healthcare access and quality index
Ischemic heart disease	Male	28-364 days	95+ years	1	Diet high in trans fatty acids
Ischemic heart disease	Female	28-364 days	95+ years	0	Alcohol (liters per capita)
Ischemic heart disease	Female	28-364 days	95+ years	1	Diabetes Fasting Plasma Glucose (mmol/L)
Ischemic heart disease	Female	28-364 days	95+ years	-1	LDI (I$ per capita)
Ischemic heart disease	Female	28-364 days	95+ years	1	Mean BMI
Ischemic heart disease	Female	28-364 days	95+ years	1	Cholesterol (total, mean per capita)
Ischemic heart disease	Female	28-364 days	95+ years	1	Systolic Blood Pressure (mmHg)
Ischemic heart disease	Female	28-364 days	95+ years	1	Indoor Air Pollution (All Cooking Fuels)
Ischemic heart disease	Female	28-364 days	95+ years	1	Outdoor Air Pollution (PM2.5)
Ischemic heart disease	Female	28-364 days	95+ years	-1	Elevation Over 1500m (proportion)
Ischemic heart disease	Female	28-364 days	95+ years	1	Smoking Prevalence
Ischemic heart disease	Female	28-364 days	95+ years	1	Log-transformed SEV scalar: IHD
Ischemic heart disease	Female	28-364 days	95+ years	0	Socio-demographic Index
Ischemic heart disease	Female	28-364 days	95+ years	-1	omega 3 adjusted(g)
Ischemic heart disease	Female	28-364 days	95+ years	-1	fruits adjusted(g)
Ischemic heart disease	Female	28-364 days	95+ years	-1	nuts seeds adjusted(g)
Ischemic heart disease	Female	28-364 days	95+ years	-1	pufa adjusted(percent)
Ischemic heart disease	Female	28-364 days	95+ years	-1	pulses legumes adjusted(g)
Ischemic heart disease	Female	28-364 days	95+ years	-1	vegetables adjusted(g)
Ischemic heart disease	Female	28-364 days	95+ years	-1	whole grains adjusted(g)
Ischemic heart disease	Female	28-364 days	95+ years	-1	Healthcare access and quality index
Ischemic heart disease	Female	28-364 days	95+ years	1	Diet high in trans fatty acids
Cerebrovascular disease	Male	0-6 days	95+ years	0	Alcohol (liters per capita)
Cerebrovascular disease	Male	0-6 days	95+ years	1	Diabetes Fasting Plasma Glucose (mmol/L)
Cerebrovascular disease	Male	0-6 days	95+ years	-1	LDI (I$ per capita)
Cerebrovascular disease	Male	0-6 days	95+ years	1	Mean BMI
Cerebrovascular disease	Male	0-6 days	95+ years	1	Cholesterol (total, mean per capita)
Cerebrovascular disease	Male	0-6 days	95+ years	1	Systolic Blood Pressure (mmHg)
Cerebrovascular disease	Male	0-6 days	95+ years	1	Indoor Air Pollution (All Cooking Fuels)
Cerebrovascular disease	Male	0-6 days	95+ years	1	Outdoor Air Pollution (PM2.5)
Cerebrovascular disease	Male	0-6 days	95+ years	-1	Elevation Over 1500m (proportion)
Cerebrovascular disease	Male	0-6 days	95+ years	1	Smoking Prevalence
Cerebrovascular disease	Male	0-6 days	95+ years	1	Log-transformed SEV scalar: Stroke
Cerebrovascular disease	Male	0-6 days	95+ years	0	Socio-demographic Index
Cerebrovascular disease	Male	0-6 days	95+ years	-1	omega 3 adjusted(g)
Cerebrovascular disease	Male	0-6 days	95+ years	-1	fruits adjusted(g)
Cerebrovascular disease	Male	0-6 days	95+ years	-1	nuts seeds adjusted(g)
Cerebrovascular disease	Male	0-6 days	95+ years	-1	pufa adjusted(percent)
Cerebrovascular disease	Male	0-6 days	95+ years	-1	pulses legumes adjusted(g)
Cerebrovascular disease	Male	0-6 days	95+ years	-1	vegetables adjusted(g)
Cerebrovascular disease	Male	0-6 days	95+ years	-1	whole grains adjusted(g)
Cerebrovascular disease	Male	0-6 days	95+ years	-1	Healthcare access and quality index
Cerebrovascular disease	Male	0-6 days	95+ years	1	Diet high in trans fatty acids
Cerebrovascular disease	Female	0-6 days	95+ years	0	Alcohol (liters per capita)
Cerebrovascular disease	Female	0-6 days	95+ years	1	Diabetes Fasting Plasma Glucose (mmol/L)
Cerebrovascular disease	Female	0-6 days	95+ years	-1	LDI (I$ per capita)
Cerebrovascular disease	Female	0-6 days	95+ years	1	Mean BMI
Cerebrovascular disease	Female	0-6 days	95+ years	1	Cholesterol (total, mean per capita)
Cerebrovascular disease	Female	0-6 days	95+ years	1	Systolic Blood Pressure (mmHg)
Cerebrovascular disease	Female	0-6 days	95+ years	1	Indoor Air Pollution (All Cooking Fuels)
Cerebrovascular disease	Female	0-6 days	95+ years	1	Outdoor Air Pollution (PM2.5)
Cerebrovascular disease	Female	0-6 days	95+ years	-1	Elevation Over 1500m (proportion)
Cerebrovascular disease	Female	0-6 days	95+ years	1	Smoking Prevalence
Cerebrovascular disease	Female	0-6 days	95+ years	1	Log-transformed SEV scalar: Stroke
Cerebrovascular disease	Female	0-6 days	95+ years	0	Socio-demographic Index
Cerebrovascular disease	Female	0-6 days	95+ years	-1	omega 3 adjusted(g)
Cerebrovascular disease	Female	0-6 days	95+ years	-1	fruits adjusted(g)
Cerebrovascular disease	Female	0-6 days	95+ years	-1	nuts seeds adjusted(g)
Cerebrovascular disease	Female	0-6 days	95+ years	-1	pufa adjusted(percent)
Cerebrovascular disease	Female	0-6 days	95+ years	-1	pulses legumes adjusted(g)
Cerebrovascular disease	Female	0-6 days	95+ years	-1	vegetables adjusted(g)
Cerebrovascular disease	Female	0-6 days	95+ years	-1	whole grains adjusted(g)
Cerebrovascular disease	Female	0-6 days	95+ years	-1	Healthcare access and quality index
Cerebrovascular disease	Female	0-6 days	95+ years	1	Diet high in trans fatty acids
Ischemic stroke	Male	28-364 days	95+ years	0	Alcohol (liters per capita)
Ischemic stroke	Male	28-364 days	95+ years	1	Diabetes Fasting Plasma Glucose (mmol/L)
Ischemic stroke	Male	28-364 days	95+ years	-1	LDI (I$ per capita)
Ischemic stroke	Male	28-364 days	95+ years	1	Mean BMI
Ischemic stroke	Male	28-364 days	95+ years	1	Cholesterol (total, mean per capita)
Ischemic stroke	Male	28-364 days	95+ years	1	Systolic Blood Pressure (mmHg)
Ischemic stroke	Male	28-364 days	95+ years	1	Indoor Air Pollution (All Cooking Fuels)
Ischemic stroke	Male	28-364 days	95+ years	1	Outdoor Air Pollution (PM2.5)
Ischemic stroke	Male	28-364 days	95+ years	-1	Elevation Over 1500m (proportion)
Ischemic stroke	Male	28-364 days	95+ years	1	Smoking Prevalence
Ischemic stroke	Male	28-364 days	95+ years	1	Log-transformed SEV scalar: Isch Stroke
Ischemic stroke	Male	28-364 days	95+ years	0	Socio-demographic Index
Ischemic stroke	Male	28-364 days	95+ years	-1	omega 3 adjusted(g)
Ischemic stroke	Male	28-364 days	95+ years	-1	fruits adjusted(g)
Ischemic stroke	Male	28-364 days	95+ years	-1	nuts seeds adjusted(g)
Ischemic stroke	Male	28-364 days	95+ years	-1	pufa adjusted(percent)
Ischemic stroke	Male	28-364 days	95+ years	-1	pulses legumes adjusted(g)
Ischemic stroke	Male	28-364 days	95+ years	-1	vegetables adjusted(g)
Ischemic stroke	Male	28-364 days	95+ years	-1	whole grains adjusted(g)
Ischemic stroke	Male	28-364 days	95+ years	-1	Healthcare access and quality index
Ischemic stroke	Male	28-364 days	95+ years	1	Diet high in trans fatty acids
Ischemic stroke	Female	28-364 days	95+ years	0	Alcohol (liters per capita)
Ischemic stroke	Female	28-364 days	95+ years	1	Diabetes Fasting Plasma Glucose (mmol/L)
Ischemic stroke	Female	28-364 days	95+ years	-1	LDI (I$ per capita)
Ischemic stroke	Female	28-364 days	95+ years	1	Mean BMI
Ischemic stroke	Female	28-364 days	95+ years	1	Cholesterol (total, mean per capita)
Ischemic stroke	Female	28-364 days	95+ years	1	Systolic Blood Pressure (mmHg)
Ischemic stroke	Female	28-364 days	95+ years	1	Indoor Air Pollution (All Cooking Fuels)
Ischemic stroke	Female	28-364 days	95+ years	1	Outdoor Air Pollution (PM2.5)

Cause	Sex	Age start	Age end	Direction	Covariate
Ischemic stroke	Female	28-364 days	95+ years	-1	Elevation Over 1500m (proportion)
Ischemic stroke	Female	28-364 days	95+ years	1	Smoking Prevalence
Ischemic stroke	Female	28-364 days	95+ years	1	Log-transformed SEV scalar: Isch Stroke
Ischemic stroke	Female	28-364 days	95+ years	0	Socio-demographic Index
Ischemic stroke	Female	28-364 days	95+ years	-1	omega 3 adjusted(g)
Ischemic stroke	Female	28-364 days	95+ years	-1	fruits adjusted(g)
Ischemic stroke	Female	28-364 days	95+ years	-1	nuts seeds adjusted(g)
Ischemic stroke	Female	28-364 days	95+ years	-1	pufa adjusted(percent)
Ischemic stroke	Female	28-364 days	95+ years	-1	pulses legumes adjusted(g)
Ischemic stroke	Female	28-364 days	95+ years	-1	vegetables adjusted(g)
Ischemic stroke	Female	28-364 days	95+ years	-1	whole grains adjusted(g)
Ischemic stroke	Female	28-364 days	95+ years	-1	Healthcare access and quality index
Ischemic stroke	Female	28-364 days	95+ years	1	Diet high in trans fatty acids
Hemorrhagic stroke	Male	0-6 days	95+ years	0	Alcohol (liters per capita)
Hemorrhagic stroke	Male	0-6 days	95+ years	1	Diabetes Fasting Plasma Glucose (mmol/L)
Hemorrhagic stroke	Male	0-6 days	95+ years	-1	LDI (I$ per capita)
Hemorrhagic stroke	Male	0-6 days	95+ years	1	Mean BMI
Hemorrhagic stroke	Male	0-6 days	95+ years	0	Cholesterol (total, mean per capita)
Hemorrhagic stroke	Male	0-6 days	95+ years	1	Systolic Blood Pressure (mmHg)
Hemorrhagic stroke	Male	0-6 days	95+ years	1	Indoor Air Pollution (All Cooking Fuels)
Hemorrhagic stroke	Male	0-6 days	95+ years	1	Outdoor Air Pollution (PM2.5)
Hemorrhagic stroke	Male	0-6 days	95+ years	-1	Elevation Over 1500m (proportion)
Hemorrhagic stroke	Male	0-6 days	95+ years	1	Smoking Prevalence
Hemorrhagic stroke	Male	0-6 days	95+ years	1	Log-transformed SEV scalar: Hem Stroke
Hemorrhagic stroke	Male	0-6 days	95+ years	0	Socio-demographic Index
Hemorrhagic stroke	Male	0-6 days	95+ years	-1	omega 3 adjusted(g)
Hemorrhagic stroke	Male	0-6 days	95+ years	-1	fruits adjusted(g)
Hemorrhagic stroke	Male	0-6 days	95+ years	-1	nuts seeds adjusted(g)
Hemorrhagic stroke	Male	0-6 days	95+ years	-1	pufa adjusted(percent)
Hemorrhagic stroke	Male	0-6 days	95+ years	-1	pulses legumes adjusted(g)
Hemorrhagic stroke	Male	0-6 days	95+ years	-1	vegetables adjusted(g)
Hemorrhagic stroke	Male	0-6 days	95+ years	-1	whole grains adjusted(g)
Hemorrhagic stroke	Male	0-6 days	95+ years	-1	Healthcare access and quality index
Hemorrhagic stroke	Male	0-6 days	95+ years	1	Diet high in trans fatty acids
Hemorrhagic stroke	Female	0-6 days	95+ years	0	Alcohol (liters per capita)
Hemorrhagic stroke	Female	0-6 days	95+ years	1	Diabetes Fasting Plasma Glucose (mmol/L)
Hemorrhagic stroke	Female	0-6 days	95+ years	-1	LDI (I$ per capita)
Hemorrhagic stroke	Female	0-6 days	95+ years	1	Mean BMI
Hemorrhagic stroke	Female	0-6 days	95+ years	0	Cholesterol (total, mean per capita)
Hemorrhagic stroke	Female	0-6 days	95+ years	1	Systolic Blood Pressure (mmHg)
Hemorrhagic stroke	Female	0-6 days	95+ years	1	Indoor Air Pollution (All Cooking Fuels)
Hemorrhagic stroke	Female	0-6 days	95+ years	1	Outdoor Air Pollution (PM2.5)
Hemorrhagic stroke	Female	0-6 days	95+ years	-1	Elevation Over 1500m (proportion)
Hemorrhagic stroke	Female	0-6 days	95+ years	1	Smoking Prevalence
Hemorrhagic stroke	Female	0-6 days	95+ years	1	Log-transformed SEV scalar: Hem Stroke
Hemorrhagic stroke	Female	0-6 days	95+ years	0	Socio-demographic Index
Hemorrhagic stroke	Female	0-6 days	95+ years	-1	omega 3 adjusted(g)
Hemorrhagic stroke	Female	0-6 days	95+ years	-1	fruits adjusted(g)
Hemorrhagic stroke	Female	0-6 days	95+ years	-1	nuts seeds adjusted(g)
Hemorrhagic stroke	Female	0-6 days	95+ years	-1	pufa adjusted(percent)
Hemorrhagic stroke	Female	0-6 days	95+ years	-1	pulses legumes adjusted(g)
Hemorrhagic stroke	Female	0-6 days	95+ years	-1	vegetables adjusted(g)
Hemorrhagic stroke	Female	0-6 days	95+ years	-1	whole grains adjusted(g)
Hemorrhagic stroke	Female	0-6 days	95+ years	-1	Healthcare access and quality index
Hemorrhagic stroke	Female	0-6 days	95+ years	1	Diet high in trans fatty acids
Hypertensive heart disease	Male	28-364 days	95+ years	-1	LDI (I$ per capita)
Hypertensive heart disease	Male	28-364 days	95+ years	1	Mean BMI
Hypertensive heart disease	Male	28-364 days	95+ years	1	Cholesterol (total, mean per capita)
Hypertensive heart disease	Male	28-364 days	95+ years	1	Systolic Blood Pressure (mmHg)
Hypertensive heart disease	Male	28-364 days	95+ years	1	Smoking Prevalence
Hypertensive heart disease	Male	28-364 days	95+ years	0	Socio-demographic Index
Hypertensive heart disease	Male	28-364 days	95+ years	-1	Healthcare access and quality index
Hypertensive heart disease	Female	28-364 days	95+ years	-1	LDI (I$ per capita)
Hypertensive heart disease	Female	28-364 days	95+ years	1	Mean BMI
Hypertensive heart disease	Female	28-364 days	95+ years	1	Cholesterol (total, mean per capita)
Hypertensive heart disease	Female	28-364 days	95+ years	1	Systolic Blood Pressure (mmHg)
Hypertensive heart disease	Female	28-364 days	95+ years	1	Smoking Prevalence
Hypertensive heart disease	Female	28-364 days	95+ years	0	Socio-demographic Index
Hypertensive heart disease	Female	28-364 days	95+ years	-1	Healthcare access and quality index
Cardiomyopathy and myocarditis	Male	0-6 days	95+ years	0	Alcohol (liters per capita)
Cardiomyopathy and myocarditis	Male	0-6 days	95+ years	0	LDI (I$ per capita)
Cardiomyopathy and myocarditis	Male	0-6 days	95+ years	1	Mean BMI
Cardiomyopathy and myocarditis	Male	0-6 days	95+ years	1	Systolic Blood Pressure (mmHg)
Cardiomyopathy and myocarditis	Male	0-6 days	95+ years	1	Smoking Prevalence
Cardiomyopathy and myocarditis	Male	0-6 days	95+ years	1	Log-transformed SEV scalar: CMP
Cardiomyopathy and myocarditis	Male	0-6 days	95+ years	0	Socio-demographic Index
Cardiomyopathy and myocarditis	Male	0-6 days	95+ years	-1	Healthcare access and quality index
Cardiomyopathy and myocarditis	Female	0-6 days	95+ years	0	Alcohol (liters per capita)
Cardiomyopathy and myocarditis	Female	0-6 days	95+ years	0	LDI (I$ per capita)
Cardiomyopathy and myocarditis	Female	0-6 days	95+ years	1	Mean BMI
Cardiomyopathy and myocarditis	Female	0-6 days	95+ years	1	Systolic Blood Pressure (mmHg)
Cardiomyopathy and myocarditis	Female	0-6 days	95+ years	1	Smoking Prevalence
Cardiomyopathy and myocarditis	Female	0-6 days	95+ years	1	Log-transformed SEV scalar: CMP
Cardiomyopathy and myocarditis	Female	0-6 days	95+ years	0	Socio-demographic Index
Cardiomyopathy and myocarditis	Female	0-6 days	95+ years	-1	Healthcare access and quality index
Aortic aneurysm	Male	15-19 years	95+ years	0	Alcohol (liters per capita)
Aortic aneurysm	Male	15-19 years	95+ years	1	Cumulative Cigarettes (10 Years)
Aortic aneurysm	Male	15-19 years	95+ years	-1	LDI (I$ per capita)
Aortic aneurysm	Male	15-19 years	95+ years	1	Mean BMI
Aortic aneurysm	Male	15-19 years	95+ years	1	Cholesterol (total, mean per capita)
Aortic aneurysm	Male	15-19 years	95+ years	1	Systolic Blood Pressure (mmHg)
Aortic aneurysm	Male	15-19 years	95+ years	1	Log-transformed SEV scalar: Aort An
Aortic aneurysm	Male	15-19 years	95+ years	0	Socio-demographic Index
Aortic aneurysm	Male	15-19 years	95+ years	-1	omega 3 adjusted(g)
Aortic aneurysm	Male	15-19 years	95+ years	-1	fruits adjusted(g)

Cause	Sex	Age start	Age end	Direction	Covariate
Aortic aneurysm	Male	15-19 years	95+ years	-1	nuts seeds adjusted(g)
Aortic aneurysm	Male	15-19 years	95+ years	-1	pufa adjusted(percent)
Aortic aneurysm	Male	15-19 years	95+ years	1	pulses legumes adjusted(g)
Aortic aneurysm	Male	15-19 years	95+ years	-1	vegetables adjusted(g)
Aortic aneurysm	Male	15-19 years	95+ years	-1	whole grains adjusted(g)
Aortic aneurysm	Male	15-19 years	95+ years	-1	Healthcare access and quality index
Aortic aneurysm	Female	15-19 years	95+ years	0	Alcohol (liters per capita)
Aortic aneurysm	Female	15-19 years	95+ years	1	Cumulative Cigarettes (10 Years)
Aortic aneurysm	Female	15-19 years	95+ years	-1	LDI (I$ per capita)
Aortic aneurysm	Female	15-19 years	95+ years	1	Mean BMI
Aortic aneurysm	Female	15-19 years	95+ years	1	Cholesterol (total, mean per capita)
Aortic aneurysm	Female	15-19 years	95+ years	1	Systolic Blood Pressure (mmHg)
Aortic aneurysm	Female	15-19 years	95+ years	1	Log-transformed SEV scalar: Aort An
Aortic aneurysm	Female	15-19 years	95+ years	0	Socio-demographic Index
Aortic aneurysm	Female	15-19 years	95+ years	-1	omega 3 adjusted(g)
Aortic aneurysm	Female	15-19 years	95+ years	-1	fruits adjusted(g)
Aortic aneurysm	Female	15-19 years	95+ years	-1	nuts seeds adjusted(g)
Aortic aneurysm	Female	15-19 years	95+ years	-1	pufa adjusted(percent)
Aortic aneurysm	Female	15-19 years	95+ years	1	pulses legumes adjusted(g)
Aortic aneurysm	Female	15-19 years	95+ years	-1	vegetables adjusted(g)
Aortic aneurysm	Female	15-19 years	95+ years	-1	whole grains adjusted(g)
Aortic aneurysm	Female	15-19 years	95+ years	-1	Healthcare access and quality index
Peripheral artery disease	Male	40-44 years	95+ years	0	Alcohol (liters per capita)
Peripheral artery disease	Male	40-44 years	95+ years	-1	LDI (I$ per capita)
Peripheral artery disease	Male	40-44 years	95+ years	1	Mean BMI
Peripheral artery disease	Male	40-44 years	95+ years	1	Cholesterol (total, mean per capita)
Peripheral artery disease	Male	40-44 years	95+ years	1	Systolic Blood Pressure (mmHg)
Peripheral artery disease	Male	40-44 years	95+ years	1	Smoking Prevalence
Peripheral artery disease	Male	40-44 years	95+ years	1	Log-transformed SEV scalar: PVD
Peripheral artery disease	Male	40-44 years	95+ years	0	Socio-demographic Index
Peripheral artery disease	Male	40-44 years	95+ years	-1	omega 3 adjusted(g)
Peripheral artery disease	Male	40-44 years	95+ years	-1	fruits adjusted(g)
Peripheral artery disease	Male	40-44 years	95+ years	-1	nuts seeds adjusted(g)
Peripheral artery disease	Male	40-44 years	95+ years	-1	pufa adjusted(percent)
Peripheral artery disease	Male	40-44 years	95+ years	-1	pulses legumes adjusted(g)
Peripheral artery disease	Male	40-44 years	95+ years	-1	vegetables adjusted(g)
Peripheral artery disease	Male	40-44 years	95+ years	-1	whole grains adjusted(g)
Peripheral artery disease	Male	40-44 years	95+ years	-1	Healthcare access and quality index
Peripheral artery disease	Female	40-44 years	95+ years	0	Alcohol (liters per capita)
Peripheral artery disease	Female	40-44 years	95+ years	-1	LDI (I$ per capita)
Peripheral artery disease	Female	40-44 years	95+ years	1	Mean BMI
Peripheral artery disease	Female	40-44 years	95+ years	1	Cholesterol (total, mean per capita)
Peripheral artery disease	Female	40-44 years	95+ years	1	Systolic Blood Pressure (mmHg)
Peripheral artery disease	Female	40-44 years	95+ years	1	Smoking Prevalence
Peripheral artery disease	Female	40-44 years	95+ years	1	Log-transformed SEV scalar: PVD
Peripheral artery disease	Female	40-44 years	95+ years	0	Socio-demographic Index
Peripheral artery disease	Female	40-44 years	95+ years	-1	omega 3 adjusted(g)
Peripheral artery disease	Female	40-44 years	95+ years	-1	fruits adjusted(g)
Peripheral artery disease	Female	40-44 years	95+ years	-1	nuts seeds adjusted(g)
Peripheral artery disease	Female	40-44 years	95+ years	-1	pufa adjusted(percent)
Peripheral artery disease	Female	40-44 years	95+ years	-1	pulses legumes adjusted(g)
Peripheral artery disease	Female	40-44 years	95+ years	-1	vegetables adjusted(g)
Peripheral artery disease	Female	40-44 years	95+ years	-1	whole grains adjusted(g)
Peripheral artery disease	Female	40-44 years	95+ years	-1	Healthcare access and quality index
Endocarditis	Female	0-6 days	95+ years	-1	LDI (I$ per capita)
Endocarditis	Female	0-6 days	95+ years	-1	Sanitation (proportion with access)
Endocarditis	Female	0-6 days	95+ years	-1	Improved Water Source (proportion with access)
Endocarditis	Female	0-6 days	95+ years	1	Log-transformed SEV scalar: Endocar
Endocarditis	Female	0-6 days	95+ years	0	Socio-demographic Index
Endocarditis	Female	0-6 days	95+ years	-1	Healthcare access and quality index
Endocarditis	Male	0-6 days	95+ years	-1	LDI (I$ per capita)
Endocarditis	Male	0-6 days	95+ years	-1	Sanitation (proportion with access)
Endocarditis	Male	0-6 days	95+ years	-1	Improved Water Source (proportion with access)
Endocarditis	Male	0-6 days	95+ years	1	Log-transformed SEV scalar: Endocar
Endocarditis	Male	0-6 days	95+ years	0	Socio-demographic Index
Endocarditis	Male	0-6 days	95+ years	-1	Healthcare access and quality index
Other cardiovascular and circulatory diseases	Male	0-6 days	95+ years	0	Alcohol (liters per capita)
Other cardiovascular and circulatory diseases	Male	0-6 days	95+ years	1	Diabetes Fasting Plasma Glucose (mmol/L)
Other cardiovascular and circulatory diseases	Male	0-6 days	95+ years	-1	LDI (I$ per capita)
Other cardiovascular and circulatory diseases	Male	0-6 days	95+ years	1	Mean BMI
Other cardiovascular and circulatory diseases	Male	0-6 days	95+ years	1	Cholesterol (total, mean per capita)
Other cardiovascular and circulatory diseases	Male	0-6 days	95+ years	1	Systolic Blood Pressure (mmHg)
Other cardiovascular and circulatory diseases	Male	0-6 days	95+ years	1	Indoor Air Pollution (All Cooking Fuels)
Other cardiovascular and circulatory diseases	Male	0-6 days	95+ years	1	Outdoor Air Pollution (PM2.5)
Other cardiovascular and circulatory diseases	Male	0-6 days	95+ years	-1	Elevation Over 1500m (proportion)
Other cardiovascular and circulatory diseases	Male	0-6 days	95+ years	1	Smoking Prevalence
Other cardiovascular and circulatory diseases	Male	0-6 days	95+ years	1	Log-transformed SEV scalar: Oth Cardio
Other cardiovascular and circulatory diseases	Male	0-6 days	95+ years	0	Socio-demographic Index
Other cardiovascular and circulatory diseases	Male	0-6 days	95+ years	-1	omega 3 adjusted(g)
Other cardiovascular and circulatory diseases	Male	0-6 days	95+ years	-1	fruits adjusted(g)
Other cardiovascular and circulatory diseases	Male	0-6 days	95+ years	-1	nuts seeds adjusted(g)
Other cardiovascular and circulatory diseases	Male	0-6 days	95+ years	-1	pufa adjusted(percent)
Other cardiovascular and circulatory diseases	Male	0-6 days	95+ years	-1	pulses legumes adjusted(g)
Other cardiovascular and circulatory diseases	Male	0-6 days	95+ years	-1	vegetables adjusted(g)
Other cardiovascular and circulatory diseases	Male	0-6 days	95+ years	-1	whole grains adjusted(g)
Other cardiovascular and circulatory diseases	Male	0-6 days	95+ years	-1	Healthcare access and quality index
Other cardiovascular and circulatory diseases	Female	0-6 days	95+ years	0	Alcohol (liters per capita)
Other cardiovascular and circulatory diseases	Female	0-6 days	95+ years	1	Diabetes Fasting Plasma Glucose (mmol/L)
Other cardiovascular and circulatory diseases	Female	0-6 days	95+ years	-1	LDI (I$ per capita)
Other cardiovascular and circulatory diseases	Female	0-6 days	95+ years	1	Mean BMI
Other cardiovascular and circulatory diseases	Female	0-6 days	95+ years	1	Cholesterol (total, mean per capita)
Other cardiovascular and circulatory diseases	Female	0-6 days	95+ years	1	Systolic Blood Pressure (mmHg)
Other cardiovascular and circulatory diseases	Female	0-6 days	95+ years	1	Indoor Air Pollution (All Cooking Fuels)
Other cardiovascular and circulatory diseases	Female	0-6 days	95+ years	1	Outdoor Air Pollution (PM2.5)
Other cardiovascular and circulatory diseases	Female	0-6 days	95+ years	-1	Elevation Over 1500m (proportion)

Cause	Sex	Age start	Age end	Direction	Covariate
Other cardiovascular and circulatory diseases	Female	0-6 days	95+ years	1	Smoking Prevalence
Other cardiovascular and circulatory diseases	Female	0-6 days	95+ years	1	Log-transformed SEV scalar: Oth Cardio
Other cardiovascular and circulatory diseases	Female	0-6 days	95+ years	0	Socio-demographic Index
Other cardiovascular and circulatory diseases	Female	0-6 days	95+ years	-1	omega 3 adjusted(g)
Other cardiovascular and circulatory diseases	Female	0-6 days	95+ years	-1	fruits adjusted(g)
Other cardiovascular and circulatory diseases	Female	0-6 days	95+ years	-1	nuts seeds adjusted(g)
Other cardiovascular and circulatory diseases	Female	0-6 days	95+ years	-1	pufa adjusted(percent)
Other cardiovascular and circulatory diseases	Female	0-6 days	95+ years	-1	pulses legumes adjusted(g)
Other cardiovascular and circulatory diseases	Female	0-6 days	95+ years	-1	vegetables adjusted(g)
Other cardiovascular and circulatory diseases	Female	0-6 days	95+ years	-1	whole grains adjusted(g)
Other cardiovascular and circulatory diseases	Female	0-6 days	95+ years	-1	Healthcare access and quality index
Chronic respiratory diseases	Male	1-4 years	95+ years	1	Cumulative Cigarettes (10 Years)
Chronic respiratory diseases	Male	1-4 years	95+ years	1	Cumulative Cigarettes (5 Years)
Chronic respiratory diseases	Male	1-4 years	95+ years	-1	Education (years per capita)
Chronic respiratory diseases	Male	1-4 years	95+ years	-1	LDI (I$ per capita)
Chronic respiratory diseases	Male	1-4 years	95+ years	1	Indoor Air Pollution (All Cooking Fuels)
Chronic respiratory diseases	Male	1-4 years	95+ years	1	Outdoor Air Pollution (PM2.5)
Chronic respiratory diseases	Male	1-4 years	95+ years	1	Elevation Over 1500m (proportion)
Chronic respiratory diseases	Male	1-4 years	95+ years	1	Elevation 500 to 1500m (proportion)
Chronic respiratory diseases	Male	1-4 years	95+ years	1	Population Density (over 1000 ppl/sqkm, proportion)
Chronic respiratory diseases	Male	1-4 years	95+ years	1	Smoking Prevalence
Chronic respiratory diseases	Male	1-4 years	95+ years	0	Socio-demographic Index
Chronic respiratory diseases	Male	1-4 years	95+ years	1	Log-transformed SEV scalar: Chr Resp
Chronic respiratory diseases	Male	1-4 years	95+ years	-1	Healthcare access and quality index
Chronic respiratory diseases	Female	1-4 years	95+ years	1	Cumulative Cigarettes (10 Years)
Chronic respiratory diseases	Female	1-4 years	95+ years	1	Cumulative Cigarettes (5 Years)
Chronic respiratory diseases	Female	1-4 years	95+ years	-1	Education (years per capita)
Chronic respiratory diseases	Female	1-4 years	95+ years	-1	LDI (I$ per capita)
Chronic respiratory diseases	Female	1-4 years	95+ years	1	Indoor Air Pollution (All Cooking Fuels)
Chronic respiratory diseases	Female	1-4 years	95+ years	1	Outdoor Air Pollution (PM2.5)
Chronic respiratory diseases	Female	1-4 years	95+ years	1	Elevation Over 1500m (proportion)
Chronic respiratory diseases	Female	1-4 years	95+ years	1	Elevation 500 to 1500m (proportion)
Chronic respiratory diseases	Female	1-4 years	95+ years	1	Population Density (over 1000 ppl/sqkm, proportion)
Chronic respiratory diseases	Female	1-4 years	95+ years	1	Smoking Prevalence
Chronic respiratory diseases	Female	1-4 years	95+ years	0	Socio-demographic Index
Chronic respiratory diseases	Female	1-4 years	95+ years	1	Log-transformed SEV scalar: Chr Resp
Chronic respiratory diseases	Female	1-4 years	95+ years	1	Healthcare access and quality index
Chronic obstructive pulmonary disease	Male	1-4 years	95+ years	1	Cumulative Cigarettes (10 Years)
Chronic obstructive pulmonary disease	Male	1-4 years	95+ years	1	Cumulative Cigarettes (20 Years)
Chronic obstructive pulmonary disease	Male	1-4 years	95+ years	1	Cumulative Cigarettes (5 Years)
Chronic obstructive pulmonary disease	Male	1-4 years	95+ years	-1	Education (years per capita)
Chronic obstructive pulmonary disease	Male	1-4 years	95+ years	-1	LDI (I$ per capita)
Chronic obstructive pulmonary disease	Male	1-4 years	95+ years	1	Indoor Air Pollution (All Cooking Fuels)
Chronic obstructive pulmonary disease	Male	1-4 years	95+ years	1	Outdoor Air Pollution (PM2.5)
Chronic obstructive pulmonary disease	Male	1-4 years	95+ years	1	Elevation Over 1500m (proportion)
Chronic obstructive pulmonary disease	Male	1-4 years	95+ years	1	Smoking Prevalence
Chronic obstructive pulmonary disease	Male	1-4 years	95+ years	1	Log-transformed SEV scalar: COPD
Chronic obstructive pulmonary disease	Male	1-4 years	95+ years	0	Socio-demographic Index
Chronic obstructive pulmonary disease	Male	1-4 years	95+ years	-1	Healthcare access and quality index
Chronic obstructive pulmonary disease	Female	1-4 years	95+ years	1	Cumulative Cigarettes (10 Years)
Chronic obstructive pulmonary disease	Female	1-4 years	95+ years	1	Cumulative Cigarettes (5 Years)
Chronic obstructive pulmonary disease	Female	1-4 years	95+ years	-1	Education (years per capita)
Chronic obstructive pulmonary disease	Female	1-4 years	95+ years	-1	LDI (I$ per capita)
Chronic obstructive pulmonary disease	Female	1-4 years	95+ years	1	Indoor Air Pollution (All Cooking Fuels)
Chronic obstructive pulmonary disease	Female	1-4 years	95+ years	1	Outdoor Air Pollution (PM2.5)
Chronic obstructive pulmonary disease	Female	1-4 years	95+ years	1	Elevation Over 1500m (proportion)
Chronic obstructive pulmonary disease	Female	1-4 years	95+ years	1	Smoking Prevalence
Chronic obstructive pulmonary disease	Female	1-4 years	95+ years	1	Log-transformed SEV scalar: COPD
Chronic obstructive pulmonary disease	Female	1-4 years	95+ years	0	Socio-demographic Index
Chronic obstructive pulmonary disease	Female	1-4 years	95+ years	1	Healthcare access and quality index
Pneumoconiosis	Female	1-4 years	95+ years	1	Coal Production (per capita)
Pneumoconiosis	Female	1-4 years	95+ years	1	Cumulative Cigarettes (5 Years)
Pneumoconiosis	Female	1-4 years	95+ years	-1	Education (years per capita)
Pneumoconiosis	Female	1-4 years	95+ years	1	Gold production (kg) per capita
Pneumoconiosis	Female	1-4 years	95+ years	-1	LDI (I$ per capita)
Pneumoconiosis	Female	1-4 years	95+ years	1	Indoor Air Pollution (All Cooking Fuels)
Pneumoconiosis	Female	1-4 years	95+ years	1	Elevation Over 1500m (proportion)
Pneumoconiosis	Female	1-4 years	95+ years	1	Elevation 500 to 1500m (proportion)
Pneumoconiosis	Female	1-4 years	95+ years	1	Smoking Prevalence
Pneumoconiosis	Female	1-4 years	95+ years	1	Log-transformed SEV scalar: Pneumocon
Pneumoconiosis	Female	1-4 years	95+ years	-1	Socio-demographic Index
Pneumoconiosis	Female	1-4 years	95+ years	-1	Healthcare access and quality index
Pneumoconiosis	Female	1-4 years	95+ years	1	Asbestos consumption (metric tons per year per capita)
Pneumoconiosis	Male	1-4 years	95+ years	1	Coal Production (per capita)
Pneumoconiosis	Male	1-4 years	95+ years	1	Cumulative Cigarettes (5 Years)
Pneumoconiosis	Male	1-4 years	95+ years	-1	Education (years per capita)
Pneumoconiosis	Male	1-4 years	95+ years	1	Gold production (kg) per capita
Pneumoconiosis	Male	1-4 years	95+ years	-1	LDI (I$ per capita)
Pneumoconiosis	Male	1-4 years	95+ years	1	Indoor Air Pollution (All Cooking Fuels)
Pneumoconiosis	Male	1-4 years	95+ years	1	Elevation Over 1500m (proportion)
Pneumoconiosis	Male	1-4 years	95+ years	1	Elevation 500 to 1500m (proportion)
Pneumoconiosis	Male	1-4 years	95+ years	1	Smoking Prevalence
Pneumoconiosis	Male	1-4 years	95+ years	1	Log-transformed SEV scalar: Pneumocon
Pneumoconiosis	Male	1-4 years	95+ years	-1	Socio-demographic Index
Pneumoconiosis	Male	1-4 years	95+ years	-1	Healthcare access and quality index
Pneumoconiosis	Male	1-4 years	95+ years	1	Asbestos consumption (metric tons per year per capita)
Silicosis	Female	1-4 years	95+ years	1	Cumulative Cigarettes (5 Years)
Silicosis	Female	1-4 years	95+ years	-1	Education (years per capita)
Silicosis	Female	1-4 years	95+ years	1	Gold production (kg) per capita
Silicosis	Female	1-4 years	95+ years	-1	LDI (I$ per capita)
Silicosis	Female	1-4 years	95+ years	1	Indoor Air Pollution (All Cooking Fuels)
Silicosis	Female	1-4 years	95+ years	1	Elevation Over 1500m (proportion)
Silicosis	Female	1-4 years	95+ years	1	Elevation 500 to 1500m (proportion)
Silicosis	Female	1-4 years	95+ years	1	Smoking Prevalence
Silicosis	Female	1-4 years	95+ years	1	Log-transformed SEV scalar: Silicosis

Cause	Sex	Age start	Age end	Direction	Covariate
Silicosis	Female	1-4 years	95+ years	-1	Socio-demographic Index
Silicosis	Female	1-4 years	95+ years	-1	Healthcare access and quality index
Silicosis	Male	1-4 years	95+ years	1	Cumulative Cigarettes (5 Years)
Silicosis	Male	1-4 years	95+ years	-1	Education (years per capita)
Silicosis	Male	1-4 years	95+ years	1	Gold production (kg) per capita
Silicosis	Male	1-4 years	95+ years	-1	LDI (I$ per capita)
Silicosis	Male	1-4 years	95+ years	1	Indoor Air Pollution (All Cooking Fuels)
Silicosis	Male	1-4 years	95+ years	1	Elevation Over 1500m (proportion)
Silicosis	Male	1-4 years	95+ years	1	Elevation 500 to 1500m (proportion)
Silicosis	Male	1-4 years	95+ years	1	Smoking Prevalence
Silicosis	Male	1-4 years	95+ years	1	Log-transformed SEV scalar: Silicosis
Silicosis	Male	1-4 years	95+ years	-1	Socio-demographic Index
Silicosis	Male	1-4 years	95+ years	-1	Healthcare access and quality index
Asbestosis	Female	1-4 years	95+ years	1	Cumulative Cigarettes (5 Years)
Asbestosis	Female	1-4 years	95+ years	-1	Education (years per capita)
Asbestosis	Female	1-4 years	95+ years	-1	LDI (I$ per capita)
Asbestosis	Female	1-4 years	95+ years	1	Indoor Air Pollution (All Cooking Fuels)
Asbestosis	Female	1-4 years	95+ years	1	Elevation Over 1500m (proportion)
Asbestosis	Female	1-4 years	95+ years	1	Elevation 500 to 1500m (proportion)
Asbestosis	Female	1-4 years	95+ years	1	Smoking Prevalence
Asbestosis	Female	1-4 years	95+ years	1	Log-transformed SEV scalar: Asbestosis
Asbestosis	Female	1-4 years	95+ years	-1	Socio-demographic Index
Asbestosis	Female	1-4 years	95+ years	-1	Healthcare access and quality index
Asbestosis	Female	1-4 years	95+ years	1	Asbestos consumption (metric tons per year per capita)
Asbestosis	Male	1-4 years	95+ years	1	Cumulative Cigarettes (5 Years)
Asbestosis	Male	1-4 years	95+ years	-1	Education (years per capita)
Asbestosis	Male	1-4 years	95+ years	-1	LDI (I$ per capita)
Asbestosis	Male	1-4 years	95+ years	1	Indoor Air Pollution (All Cooking Fuels)
Asbestosis	Male	1-4 years	95+ years	1	Elevation Over 1500m (proportion)
Asbestosis	Male	1-4 years	95+ years	1	Elevation 500 to 1500m (proportion)
Asbestosis	Male	1-4 years	95+ years	1	Smoking Prevalence
Asbestosis	Male	1-4 years	95+ years	1	Log-transformed SEV scalar: Asbestosis
Asbestosis	Male	1-4 years	95+ years	-1	Socio-demographic Index
Asbestosis	Male	1-4 years	95+ years	-1	Healthcare access and quality index
Asbestosis	Male	1-4 years	95+ years	1	Asbestos consumption (metric tons per year per capita)
Asbestosis	Male	1-4 years	95+ years	0	Socio-demographic Index
Coal workers pneumoconiosis	Male	1-4 years	95+ years	1	Coal Production (per capita)
Coal workers pneumoconiosis	Male	1-4 years	95+ years	1	Cumulative Cigarettes (5 Years)
Coal workers pneumoconiosis	Male	1-4 years	95+ years	-1	Education (years per capita)
Coal workers pneumoconiosis	Male	1-4 years	95+ years	-1	LDI (I$ per capita)
Coal workers pneumoconiosis	Male	1-4 years	95+ years	1	Indoor Air Pollution (All Cooking Fuels)
Coal workers pneumoconiosis	Male	1-4 years	95+ years	1	Elevation Over 1500m (proportion)
Coal workers pneumoconiosis	Male	1-4 years	95+ years	1	Elevation 500 to 1500m (proportion)
Coal workers pneumoconiosis	Male	1-4 years	95+ years	1	Smoking Prevalence
Coal workers pneumoconiosis	Male	1-4 years	95+ years	1	Log-transformed SEV scalar: Coal W
Coal workers pneumoconiosis	Male	1-4 years	95+ years	-1	Socio-demographic Index
Coal workers pneumoconiosis	Male	1-4 years	95+ years	-1	Healthcare access and quality index
Coal workers pneumoconiosis	Female	1-4 years	95+ years	1	Coal Production (per capita)
Coal workers pneumoconiosis	Female	1-4 years	95+ years	1	Cumulative Cigarettes (5 Years)
Coal workers pneumoconiosis	Female	1-4 years	95+ years	-1	Education (years per capita)
Coal workers pneumoconiosis	Female	1-4 years	95+ years	-1	LDI (I$ per capita)
Coal workers pneumoconiosis	Female	1-4 years	95+ years	1	Indoor Air Pollution (All Cooking Fuels)
Coal workers pneumoconiosis	Female	1-4 years	95+ years	1	Elevation Over 1500m (proportion)
Coal workers pneumoconiosis	Female	1-4 years	95+ years	1	Elevation 500 to 1500m (proportion)
Coal workers pneumoconiosis	Female	1-4 years	95+ years	1	Smoking Prevalence
Coal workers pneumoconiosis	Female	1-4 years	95+ years	1	Log-transformed SEV scalar: Coal W
Coal workers pneumoconiosis	Female	1-4 years	95+ years	-1	Socio-demographic Index
Coal workers pneumoconiosis	Female	1-4 years	95+ years	-1	Healthcare access and quality index
Other pneumoconiosis	Male	1-4 years	95+ years	1	Cumulative Cigarettes (5 Years)
Other pneumoconiosis	Male	1-4 years	95+ years	-1	Education (years per capita)
Other pneumoconiosis	Male	1-4 years	95+ years	-1	LDI (I$ per capita)
Other pneumoconiosis	Male	1-4 years	95+ years	1	Indoor Air Pollution (All Cooking Fuels)
Other pneumoconiosis	Male	1-4 years	95+ years	1	Elevation Over 1500m (proportion)
Other pneumoconiosis	Male	1-4 years	95+ years	1	Elevation 500 to 1500m (proportion)
Other pneumoconiosis	Male	1-4 years	95+ years	1	Smoking Prevalence
Other pneumoconiosis	Male	1-4 years	95+ years	1	Log-transformed SEV scalar: Oth Pneum
Other pneumoconiosis	Male	1-4 years	95+ years	-1	Socio-demographic Index
Other pneumoconiosis	Male	1-4 years	95+ years	-1	Healthcare access and quality index
Other pneumoconiosis	Female	1-4 years	95+ years	1	Cumulative Cigarettes (5 Years)
Other pneumoconiosis	Female	1-4 years	95+ years	-1	Education (years per capita)
Other pneumoconiosis	Female	1-4 years	95+ years	-1	LDI (I$ per capita)
Other pneumoconiosis	Female	1-4 years	95+ years	1	Indoor Air Pollution (All Cooking Fuels)
Other pneumoconiosis	Female	1-4 years	95+ years	1	Elevation Over 1500m (proportion)
Other pneumoconiosis	Female	1-4 years	95+ years	1	Elevation 500 to 1500m (proportion)
Other pneumoconiosis	Female	1-4 years	95+ years	1	Smoking Prevalence
Other pneumoconiosis	Female	1-4 years	95+ years	1	Log-transformed SEV scalar: Oth Pneum
Other pneumoconiosis	Female	1-4 years	95+ years	-1	Socio-demographic Index
Other pneumoconiosis	Female	1-4 years	95+ years	-1	Healthcare access and quality index
Asthma	Female	1-4 years	95+ years	1	Cumulative Cigarettes (10 Years)
Asthma	Female	1-4 years	95+ years	1	Cumulative Cigarettes (5 Years)
Asthma	Female	1-4 years	95+ years	-1	Education (years per capita)
Asthma	Female	1-4 years	95+ years	-1	LDI (I$ per capita)
Asthma	Female	1-4 years	95+ years	1	Indoor Air Pollution (All Cooking Fuels)
Asthma	Female	1-4 years	95+ years	1	Outdoor Air Pollution (PM2.5)
Asthma	Female	1-4 years	95+ years	1	Smoking Prevalence
Asthma	Female	1-4 years	95+ years	1	Log-transformed SEV scalar: Asthma
Asthma	Female	1-4 years	95+ years	-1	Socio-demographic Index
Asthma	Female	1-4 years	95+ years	-1	Healthcare access and quality index
Asthma	Male	1-4 years	95+ years	1	Cumulative Cigarettes (10 Years)
Asthma	Male	1-4 years	95+ years	1	Cumulative Cigarettes (5 Years)
Asthma	Male	1-4 years	95+ years	-1	Education (years per capita)
Asthma	Male	1-4 years	95+ years	-1	LDI (I$ per capita)
Asthma	Male	1-4 years	95+ years	1	Indoor Air Pollution (All Cooking Fuels)
Asthma	Male	1-4 years	95+ years	1	Outdoor Air Pollution (PM2.5)
Asthma	Male	1-4 years	95+ years	1	Smoking Prevalence

Cause	Sex	Age start	Age end	Direction	Covariate
Asthma	Male	1-4 years	95+ years	1	Log-transformed SEV scalar: Asthma
Asthma	Male	1-4 years	95+ years	-1	Socio-demographic Index
Asthma	Male	1-4 years	95+ years	-1	Healthcare access and quality index
Interstitial lung disease and pulmonary sarcoidosis	Female	1-4 years	95+ years	1	Cumulative Cigarettes (5 Years)
Interstitial lung disease and pulmonary sarcoidosis	Female	1-4 years	95+ years	-1	Education (years per capita)
Interstitial lung disease and pulmonary sarcoidosis	Female	1-4 years	95+ years	-1	LDI (I$ per capita)
Interstitial lung disease and pulmonary sarcoidosis	Female	1-4 years	95+ years	1	Indoor Air Pollution (All Cooking Fuels)
Interstitial lung disease and pulmonary sarcoidosis	Female	1-4 years	95+ years	1	Outdoor Air Pollution (PM2.5)
Interstitial lung disease and pulmonary sarcoidosis	Female	1-4 years	95+ years	1	Elevation Over 1500m (proportion)
Interstitial lung disease and pulmonary sarcoidosis	Female	1-4 years	95+ years	1	Elevation 500 to 1500m (proportion)
Interstitial lung disease and pulmonary sarcoidosis	Female	1-4 years	95+ years	1	Population Density (over 1000 ppl/sqkm, proportion)
Interstitial lung disease and pulmonary sarcoidosis	Female	1-4 years	95+ years	1	Smoking Prevalence
Interstitial lung disease and pulmonary sarcoidosis	Female	1-4 years	95+ years	1	Log-transformed SEV scalar: ILD
Interstitial lung disease and pulmonary sarcoidosis	Female	1-4 years	95+ years	0	Socio-demographic Index
Interstitial lung disease and pulmonary sarcoidosis	Female	1-4 years	95+ years	-1	Healthcare access and quality index
Interstitial lung disease and pulmonary sarcoidosis	Male	1-4 years	95+ years	1	Cumulative Cigarettes (5 Years)
Interstitial lung disease and pulmonary sarcoidosis	Male	1-4 years	95+ years	-1	Education (years per capita)
Interstitial lung disease and pulmonary sarcoidosis	Male	1-4 years	95+ years	-1	LDI (I$ per capita)
Interstitial lung disease and pulmonary sarcoidosis	Male	1-4 years	95+ years	1	Indoor Air Pollution (All Cooking Fuels)
Interstitial lung disease and pulmonary sarcoidosis	Male	1-4 years	95+ years	1	Outdoor Air Pollution (PM2.5)
Interstitial lung disease and pulmonary sarcoidosis	Male	1-4 years	95+ years	1	Elevation Over 1500m (proportion)
Interstitial lung disease and pulmonary sarcoidosis	Male	1-4 years	95+ years	1	Elevation 500 to 1500m (proportion)
Interstitial lung disease and pulmonary sarcoidosis	Male	1-4 years	95+ years	1	Population Density (over 1000 ppl/sqkm, proportion)
Interstitial lung disease and pulmonary sarcoidosis	Male	1-4 years	95+ years	1	Smoking Prevalence
Interstitial lung disease and pulmonary sarcoidosis	Male	1-4 years	95+ years	1	Log-transformed SEV scalar: ILD
Interstitial lung disease and pulmonary sarcoidosis	Male	1-4 years	95+ years	0	Socio-demographic Index
Interstitial lung disease and pulmonary sarcoidosis	Male	1-4 years	95+ years	-1	Healthcare access and quality index
Other chronic respiratory diseases	Female	1-4 years	95+ years	1	Cumulative Cigarettes (5 Years)
Other chronic respiratory diseases	Female	1-4 years	95+ years	-1	Education (years per capita)
Other chronic respiratory diseases	Female	1-4 years	95+ years	-1	LDI (I$ per capita)
Other chronic respiratory diseases	Female	1-4 years	95+ years	1	Indoor Air Pollution (All Cooking Fuels)
Other chronic respiratory diseases	Female	1-4 years	95+ years	1	Outdoor Air Pollution (PM2.5)
Other chronic respiratory diseases	Female	1-4 years	95+ years	1	Elevation Over 1500m (proportion)
Other chronic respiratory diseases	Female	1-4 years	95+ years	1	Elevation 500 to 1500m (proportion)
Other chronic respiratory diseases	Female	1-4 years	95+ years	1	Population Density (over 1000 ppl/sqkm, proportion)
Other chronic respiratory diseases	Female	1-4 years	95+ years	1	Smoking Prevalence
Other chronic respiratory diseases	Female	1-4 years	95+ years	1	Log-transformed SEV scalar: Oth Resp
Other chronic respiratory diseases	Female	1-4 years	95+ years	-1	Socio-demographic Index
Other chronic respiratory diseases	Female	1-4 years	95+ years	-1	Healthcare access and quality index
Other chronic respiratory diseases	Male	1-4 years	95+ years	1	Cumulative Cigarettes (5 Years)
Other chronic respiratory diseases	Male	1-4 years	95+ years	-1	Education (years per capita)
Other chronic respiratory diseases	Male	1-4 years	95+ years	-1	LDI (I$ per capita)
Other chronic respiratory diseases	Male	1-4 years	95+ years	1	Indoor Air Pollution (All Cooking Fuels)
Other chronic respiratory diseases	Male	1-4 years	95+ years	1	Outdoor Air Pollution (PM2.5)
Other chronic respiratory diseases	Male	1-4 years	95+ years	1	Elevation Over 1500m (proportion)
Other chronic respiratory diseases	Male	1-4 years	95+ years	1	Elevation 500 to 1500m (proportion)
Other chronic respiratory diseases	Male	1-4 years	95+ years	1	Population Density (over 1000 ppl/sqkm, proportion)
Other chronic respiratory diseases	Male	1-4 years	95+ years	1	Smoking Prevalence
Other chronic respiratory diseases	Male	1-4 years	95+ years	1	Log-transformed SEV scalar: Oth Resp
Other chronic respiratory diseases	Male	1-4 years	95+ years	-1	Socio-demographic Index
Other chronic respiratory diseases	Male	1-4 years	95+ years	-1	Healthcare access and quality index
Cirrhosis and other chronic liver diseases	Male	1-4 years	95+ years	1	Alcohol (liters per capita)
Cirrhosis and other chronic liver diseases	Male	1-4 years	95+ years	1	Diabetes Age-Standardized Prevalence (proportion)
Cirrhosis and other chronic liver diseases	Male	1-4 years	95+ years	-1	Education (years per capita)
Cirrhosis and other chronic liver diseases	Male	1-4 years	95+ years	-1	Health System Access 2 (unitless)
Cirrhosis and other chronic liver diseases	Male	1-4 years	95+ years	-1	LDI (I$ per capita)
Cirrhosis and other chronic liver diseases	Male	1-4 years	95+ years	1	Mean BMI
Cirrhosis and other chronic liver diseases	Male	1-4 years	95+ years	1	Schistosomiasis Prevalence (proportion)
Cirrhosis and other chronic liver diseases	Male	1-4 years	95+ years	0	Socio-demographic Index
Cirrhosis and other chronic liver diseases	Male	1-4 years	95+ years	1	Hepatitis B (HBsAg) Seroprevalence
Cirrhosis and other chronic liver diseases	Male	1-4 years	95+ years	1	Hepatitis C (IgG) Seroprevalence
Cirrhosis and other chronic liver diseases	Male	1-4 years	95+ years	-1	Healthcare access and quality index
Cirrhosis and other chronic liver diseases	Female	1-4 years	95+ years	1	Alcohol (liters per capita)
Cirrhosis and other chronic liver diseases	Female	1-4 years	95+ years	1	Diabetes Age-Standardized Prevalence (proportion)
Cirrhosis and other chronic liver diseases	Female	1-4 years	95+ years	-1	Education (years per capita)
Cirrhosis and other chronic liver diseases	Female	1-4 years	95+ years	-1	Health System Access 2 (unitless)
Cirrhosis and other chronic liver diseases	Female	1-4 years	95+ years	-1	LDI (I$ per capita)
Cirrhosis and other chronic liver diseases	Female	1-4 years	95+ years	1	Mean BMI
Cirrhosis and other chronic liver diseases	Female	1-4 years	95+ years	1	Schistosomiasis Prevalence (proportion)
Cirrhosis and other chronic liver diseases	Female	1-4 years	95+ years	0	Socio-demographic Index
Cirrhosis and other chronic liver diseases	Female	1-4 years	95+ years	1	Hepatitis B (HBsAg) Seroprevalence
Cirrhosis and other chronic liver diseases	Female	1-4 years	95+ years	1	Hepatitis C (IgG) Seroprevalence
Cirrhosis and other chronic liver diseases	Female	1-4 years	95+ years	-1	Healthcare access and quality index
Digestive diseases	Female	0-6 days	95+ years	1	Alcohol (liters per capita)
Digestive diseases	Female	0-6 days	95+ years	1	Cumulative Cigarettes (5 Years)
Digestive diseases	Female	0-6 days	95+ years	-1	Education (years per capita)
Digestive diseases	Female	0-6 days	95+ years	-1	LDI (I$ per capita)
Digestive diseases	Female	0-6 days	95+ years	-1	Sanitation (proportion with access)
Digestive diseases	Female	0-6 days	95+ years	-1	Socio-demographic Index
Digestive diseases	Female	0-6 days	95+ years	-1	fruits adjusted(g)
Digestive diseases	Female	0-6 days	95+ years	1	red meats adjusted(g)
Digestive diseases	Female	0-6 days	95+ years	-1	Healthcare access and quality index
Digestive diseases	Male	0-6 days	95+ years	1	Alcohol (liters per capita)
Digestive diseases	Male	0-6 days	95+ years	1	Cumulative Cigarettes (5 Years)
Digestive diseases	Male	0-6 days	95+ years	-1	Education (years per capita)
Digestive diseases	Male	0-6 days	95+ years	-1	LDI (I$ per capita)
Digestive diseases	Male	0-6 days	95+ years	-1	Sanitation (proportion with access)
Digestive diseases	Male	0-6 days	95+ years	-1	Socio-demographic Index
Digestive diseases	Male	0-6 days	95+ years	-1	fruits adjusted(g)
Digestive diseases	Male	0-6 days	95+ years	1	red meats adjusted(g)
Digestive diseases	Male	0-6 days	95+ years	-1	Healthcare access and quality index
Peptic ulcer disease	Female	1-4 years	95+ years	1	Alcohol (liters per capita)
Peptic ulcer disease	Female	1-4 years	95+ years	1	Cumulative Cigarettes (10 Years)
Peptic ulcer disease	Female	1-4 years	95+ years	1	Cumulative Cigarettes (5 Years)
Peptic ulcer disease	Female	1-4 years	95+ years	-1	LDI (I$ per capita)

Cause	Sex	Age start	Age end	Direction	Covariate
Peptic ulcer disease	Female	1-4 years	95+ years	-1	Sanitation (proportion with access)
Peptic ulcer disease	Female	1-4 years	95+ years	1	Smoking Prevalence
Peptic ulcer disease	Female	1-4 years	95+ years	-1	Maternal education (years per capita)
Peptic ulcer disease	Female	1-4 years	95+ years	1	SEV unsafe water
Peptic ulcer disease	Female	1-4 years	95+ years	-1	Socio-demographic Index
Peptic ulcer disease	Female	1-4 years	95+ years	0	vegetables adjusted(g)
Peptic ulcer disease	Female	1-4 years	95+ years	-1	Healthcare access and quality index
Peptic ulcer disease	Male	1-4 years	95+ years	1	Alcohol (liters per capita)
Peptic ulcer disease	Male	1-4 years	95+ years	1	Cumulative Cigarettes (10 Years)
Peptic ulcer disease	Male	1-4 years	95+ years	1	Cumulative Cigarettes (5 Years)
Peptic ulcer disease	Male	1-4 years	95+ years	-1	LDI (I$ per capita)
Peptic ulcer disease	Male	1-4 years	95+ years	-1	Sanitation (proportion with access)
Peptic ulcer disease	Male	1-4 years	95+ years	1	Smoking Prevalence
Peptic ulcer disease	Male	1-4 years	95+ years	-1	Maternal education (years per capita)
Peptic ulcer disease	Male	1-4 years	95+ years	1	SEV unsafe water
Peptic ulcer disease	Male	1-4 years	95+ years	-1	Socio-demographic Index
Peptic ulcer disease	Male	1-4 years	95+ years	0	vegetables adjusted(g)
Peptic ulcer disease	Male	1-4 years	95+ years	-1	Healthcare access and quality index
Gastritis and duodenitis	Male	1-4 years	95+ years	1	Alcohol (liters per capita)
Gastritis and duodenitis	Male	1-4 years	95+ years	1	Cumulative Cigarettes (10 Years)
Gastritis and duodenitis	Male	1-4 years	95+ years	1	Cumulative Cigarettes (5 Years)
Gastritis and duodenitis	Male	1-4 years	95+ years	-1	Education (years per capita)
Gastritis and duodenitis	Male	1-4 years	95+ years	-1	LDI (I$ per capita)
Gastritis and duodenitis	Male	1-4 years	95+ years	-1	Sanitation (proportion with access)
Gastritis and duodenitis	Male	1-4 years	95+ years	1	Smoking Prevalence
Gastritis and duodenitis	Male	1-4 years	95+ years	1	SEV unsafe water
Gastritis and duodenitis	Male	1-4 years	95+ years	-1	Socio-demographic Index
Gastritis and duodenitis	Male	1-4 years	95+ years	0	vegetables adjusted(g)
Gastritis and duodenitis	Male	1-4 years	95+ years	-1	Healthcare access and quality index
Gastritis and duodenitis	Female	1-4 years	95+ years	1	Alcohol (liters per capita)
Gastritis and duodenitis	Female	1-4 years	95+ years	1	Cumulative Cigarettes (10 Years)
Gastritis and duodenitis	Female	1-4 years	95+ years	1	Cumulative Cigarettes (5 Years)
Gastritis and duodenitis	Female	1-4 years	95+ years	-1	Education (years per capita)
Gastritis and duodenitis	Female	1-4 years	95+ years	-1	LDI (I$ per capita)
Gastritis and duodenitis	Female	1-4 years	95+ years	-1	Sanitation (proportion with access)
Gastritis and duodenitis	Female	1-4 years	95+ years	1	Smoking Prevalence
Gastritis and duodenitis	Female	1-4 years	95+ years	1	SEV unsafe water
Gastritis and duodenitis	Female	1-4 years	95+ years	-1	Socio-demographic Index
Gastritis and duodenitis	Female	1-4 years	95+ years	0	vegetables adjusted(g)
Gastritis and duodenitis	Female	1-4 years	95+ years	-1	Healthcare access and quality index
Appendicitis	Male	1-4 years	95+ years	-1	Education (years per capita)
Appendicitis	Male	1-4 years	95+ years	-1	LDI (I$ per capita)
Appendicitis	Male	1-4 years	95+ years	-1	Health System Access (capped)
Appendicitis	Male	1-4 years	95+ years	-1	Socio-demographic Index
Appendicitis	Male	1-4 years	95+ years	-1	fruits adjusted(g)
Appendicitis	Male	1-4 years	95+ years	-1	vegetables adjusted(g)
Appendicitis	Male	1-4 years	95+ years	-1	Healthcare access and quality index
Appendicitis	Female	1-4 years	95+ years	-1	Education (years per capita)
Appendicitis	Female	1-4 years	95+ years	-1	LDI (I$ per capita)
Appendicitis	Female	1-4 years	95+ years	-1	Health System Access (capped)
Appendicitis	Female	1-4 years	95+ years	-1	Socio-demographic Index
Appendicitis	Female	1-4 years	95+ years	-1	fruits adjusted(g)
Appendicitis	Female	1-4 years	95+ years	-1	vegetables adjusted(g)
Appendicitis	Female	1-4 years	95+ years	-1	Healthcare access and quality index
Paralytic ileus and intestinal obstruction	Male	0-6 days	95+ years	-1	Education (years per capita)
Paralytic ileus and intestinal obstruction	Male	0-6 days	95+ years	-1	LDI (I$ per capita)
Paralytic ileus and intestinal obstruction	Male	0-6 days	95+ years	-1	Health System Access (capped)
Paralytic ileus and intestinal obstruction	Male	0-6 days	95+ years	-1	Socio-demographic Index
Paralytic ileus and intestinal obstruction	Male	0-6 days	95+ years	-1	fruits adjusted(g)
Paralytic ileus and intestinal obstruction	Male	0-6 days	95+ years	-1	vegetables adjusted(g)
Paralytic ileus and intestinal obstruction	Male	0-6 days	95+ years	-1	Healthcare access and quality index
Paralytic ileus and intestinal obstruction	Female	0-6 days	95+ years	-1	Education (years per capita)
Paralytic ileus and intestinal obstruction	Female	0-6 days	95+ years	-1	LDI (I$ per capita)
Paralytic ileus and intestinal obstruction	Female	0-6 days	95+ years	-1	Health System Access (capped)
Paralytic ileus and intestinal obstruction	Female	0-6 days	95+ years	-1	Socio-demographic Index
Paralytic ileus and intestinal obstruction	Female	0-6 days	95+ years	-1	fruits adjusted(g)
Paralytic ileus and intestinal obstruction	Female	0-6 days	95+ years	-1	vegetables adjusted(g)
Paralytic ileus and intestinal obstruction	Female	0-6 days	95+ years	-1	Healthcare access and quality index
Inguinal, femoral, and abdominal hernia	Female	1-4 years	95+ years	-1	Education (years per capita)
Inguinal, femoral, and abdominal hernia	Female	1-4 years	95+ years	-1	LDI (I$ per capita)
Inguinal, femoral, and abdominal hernia	Female	1-4 years	95+ years	0	Socio-demographic Index
Inguinal, femoral, and abdominal hernia	Female	1-4 years	95+ years	-1	Healthcare access and quality index
Inguinal, femoral, and abdominal hernia	Male	1-4 years	95+ years	-1	Education (years per capita)
Inguinal, femoral, and abdominal hernia	Male	1-4 years	95+ years	-1	LDI (I$ per capita)
Inguinal, femoral, and abdominal hernia	Male	1-4 years	95+ years	0	Socio-demographic Index
Inguinal, femoral, and abdominal hernia	Male	1-4 years	95+ years	-1	Healthcare access and quality index
Inflammatory bowel disease	Male	1-4 years	95+ years	-1	Education (years per capita)
Inflammatory bowel disease	Male	1-4 years	95+ years	0	LDI (I$ per capita)
Inflammatory bowel disease	Male	1-4 years	95+ years	-1	Latitude 15 to 30 (proportion)
Inflammatory bowel disease	Male	1-4 years	95+ years	1	Latitude 30 to 45 (proportion)
Inflammatory bowel disease	Male	1-4 years	95+ years	1	Latitude Over 45 (proportion)
Inflammatory bowel disease	Male	1-4 years	95+ years	0	Socio-demographic Index
Inflammatory bowel disease	Male	1-4 years	95+ years	-1	fruits adjusted(g)
Inflammatory bowel disease	Male	1-4 years	95+ years	1	red meats adjusted(g)
Inflammatory bowel disease	Male	1-4 years	95+ years	1	saturated fats adjusted(percent)
Inflammatory bowel disease	Male	1-4 years	95+ years	-1	vegetables adjusted(g)
Inflammatory bowel disease	Male	1-4 years	95+ years	-1	Healthcare access and quality index
Inflammatory bowel disease	Female	1-4 years	95+ years	-1	Education (years per capita)
Inflammatory bowel disease	Female	1-4 years	95+ years	0	LDI (I$ per capita)
Inflammatory bowel disease	Female	1-4 years	95+ years	-1	Latitude 15 to 30 (proportion)
Inflammatory bowel disease	Female	1-4 years	95+ years	1	Latitude 30 to 45 (proportion)
Inflammatory bowel disease	Female	1-4 years	95+ years	1	Latitude Over 45 (proportion)
Inflammatory bowel disease	Female	1-4 years	95+ years	0	Socio-demographic Index
Inflammatory bowel disease	Female	1-4 years	95+ years	-1	fruits adjusted(g)
Inflammatory bowel disease	Female	1-4 years	95+ years	1	red meats adjusted(g)

Cause	Sex	Age start	Age end	Direction	Covariate
Inflammatory bowel disease	Female	1-4 years	95+ years	1	saturated fats adjusted(percent)
Inflammatory bowel disease	Female	1-4 years	95+ years	-1	vegetables adjusted(g)
Inflammatory bowel disease	Female	1-4 years	95+ years	-1	Healthcare access and quality index
Vascular intestinal disorders	Female	1-4 years	95+ years	1	Alcohol (liters per capita)
Vascular intestinal disorders	Female	1-4 years	95+ years	1	Diabetes Fasting Plasma Glucose (mmol/L)
Vascular intestinal disorders	Female	1-4 years	95+ years	1	Diabetes Age-Standardized Prevalence (proportion)
Vascular intestinal disorders	Female	1-4 years	95+ years	-1	Education (years per capita)
Vascular intestinal disorders	Female	1-4 years	95+ years	-1	LDI (I$ per capita)
Vascular intestinal disorders	Female	1-4 years	95+ years	1	Cholesterol (total, mean per capita)
Vascular intestinal disorders	Female	1-4 years	95+ years	1	Systolic Blood Pressure (mmHg)
Vascular intestinal disorders	Female	1-4 years	95+ years	1	Latitude Over 45 (proportion)
Vascular intestinal disorders	Female	1-4 years	95+ years	0	Socio-demographic Index
Vascular intestinal disorders	Female	1-4 years	95+ years	-1	fruits adjusted(g)
Vascular intestinal disorders	Female	1-4 years	95+ years	1	saturated fats adjusted(percent)
Vascular intestinal disorders	Female	1-4 years	95+ years	-1	vegetables adjusted(g)
Vascular intestinal disorders	Female	1-4 years	95+ years	-1	Healthcare access and quality index
Vascular intestinal disorders	Male	1-4 years	95+ years	1	Alcohol (liters per capita)
Vascular intestinal disorders	Male	1-4 years	95+ years	1	Diabetes Fasting Plasma Glucose (mmol/L)
Vascular intestinal disorders	Male	1-4 years	95+ years	1	Diabetes Age-Standardized Prevalence (proportion)
Vascular intestinal disorders	Male	1-4 years	95+ years	-1	Education (years per capita)
Vascular intestinal disorders	Male	1-4 years	95+ years	-1	LDI (I$ per capita)
Vascular intestinal disorders	Male	1-4 years	95+ years	1	Cholesterol (total, mean per capita)
Vascular intestinal disorders	Male	1-4 years	95+ years	1	Systolic Blood Pressure (mmHg)
Vascular intestinal disorders	Male	1-4 years	95+ years	1	Latitude Over 45 (proportion)
Vascular intestinal disorders	Male	1-4 years	95+ years	0	Socio-demographic Index
Vascular intestinal disorders	Male	1-4 years	95+ years	-1	fruits adjusted(g)
Vascular intestinal disorders	Male	1-4 years	95+ years	1	saturated fats adjusted(percent)
Vascular intestinal disorders	Male	1-4 years	95+ years	-1	vegetables adjusted(g)
Vascular intestinal disorders	Male	1-4 years	95+ years	-1	Healthcare access and quality index
Gallbladder and biliary diseases	Female	1-4 years	95+ years	1	Alcohol (liters per capita)
Gallbladder and biliary diseases	Female	1-4 years	95+ years	0	Education (years per capita)
Gallbladder and biliary diseases	Female	1-4 years	95+ years	0	LDI (I$ per capita)
Gallbladder and biliary diseases	Female	1-4 years	95+ years	1	Mean BMI
Gallbladder and biliary diseases	Female	1-4 years	95+ years	1	Population Over 65 (proportion)
Gallbladder and biliary diseases	Female	1-4 years	95+ years	0	Socio-demographic Index
Gallbladder and biliary diseases	Female	1-4 years	95+ years	1	red meats adjusted(g)
Gallbladder and biliary diseases	Female	1-4 years	95+ years	1	saturated fats adjusted(percent)
Gallbladder and biliary diseases	Female	1-4 years	95+ years	-1	Healthcare access and quality index
Gallbladder and biliary diseases	Male	1-4 years	95+ years	1	Alcohol (liters per capita)
Gallbladder and biliary diseases	Male	1-4 years	95+ years	0	Education (years per capita)
Gallbladder and biliary diseases	Male	1-4 years	95+ years	0	LDI (I$ per capita)
Gallbladder and biliary diseases	Male	1-4 years	95+ years	1	Mean BMI
Gallbladder and biliary diseases	Male	1-4 years	95+ years	1	Population Over 65 (proportion)
Gallbladder and biliary diseases	Male	1-4 years	95+ years	0	Socio-demographic Index
Gallbladder and biliary diseases	Male	1-4 years	95+ years	1	red meats adjusted(g)
Gallbladder and biliary diseases	Male	1-4 years	95+ years	1	saturated fats adjusted(percent)
Gallbladder and biliary diseases	Male	1-4 years	95+ years	-1	Healthcare access and quality index
Gallbladder and biliary diseases	Male	1-4 years	95+ years	-1	Health System Access (capped)
Pancreatitis	Female	1-4 years	95+ years	1	Alcohol (liters per capita)
Pancreatitis	Female	1-4 years	95+ years	-1	Education (years per capita)
Pancreatitis	Female	1-4 years	95+ years	0	LDI (I$ per capita)
Pancreatitis	Female	1-4 years	95+ years	1	Mean BMI
Pancreatitis	Female	1-4 years	95+ years	1	Log-transformed SEV scalar: Pancreatit
Pancreatitis	Female	1-4 years	95+ years	0	Socio-demographic Index
Pancreatitis	Female	1-4 years	95+ years	-1	Healthcare access and quality index
Pancreatitis	Male	1-4 years	95+ years	1	Alcohol (liters per capita)
Pancreatitis	Male	1-4 years	95+ years	-1	Education (years per capita)
Pancreatitis	Male	1-4 years	95+ years	0	LDI (I$ per capita)
Pancreatitis	Male	1-4 years	95+ years	1	Mean BMI
Pancreatitis	Male	1-4 years	95+ years	1	Log-transformed SEV scalar: Pancreatit
Pancreatitis	Male	1-4 years	95+ years	0	Socio-demographic Index
Pancreatitis	Male	1-4 years	95+ years	-1	Healthcare access and quality index
Pancreatitis	Male	1-4 years	95+ years	-1	Health System Access (capped)
Other digestive diseases	Male	1-4 years	95+ years	1	Alcohol (liters per capita)
Other digestive diseases	Male	1-4 years	95+ years	1	Cumulative Cigarettes (10 Years)
Other digestive diseases	Male	1-4 years	95+ years	1	Cumulative Cigarettes (5 Years)
Other digestive diseases	Male	1-4 years	95+ years	1	Diabetes Age-Standardized Prevalence (proportion)
Other digestive diseases	Male	1-4 years	95+ years	-1	Education (years per capita)
Other digestive diseases	Male	1-4 years	95+ years	-1	Health System Access 2 (unitless)
Other digestive diseases	Male	1-4 years	95+ years	-1	LDI (I$ per capita)
Other digestive diseases	Male	1-4 years	95+ years	1	Mean BMI
Other digestive diseases	Male	1-4 years	95+ years	-1	Sanitation (proportion with access)
Other digestive diseases	Male	1-4 years	95+ years	1	Smoking Prevalence
Other digestive diseases	Male	1-4 years	95+ years	-1	Improved Water Source (proportion with access)
Other digestive diseases	Male	1-4 years	95+ years	0	Socio-demographic Index
Other digestive diseases	Male	1-4 years	95+ years	-1	fruits adjusted(g)
Other digestive diseases	Male	1-4 years	95+ years	1	red meats adjusted(g)
Other digestive diseases	Male	1-4 years	95+ years	1	saturated fats adjusted(percent)
Other digestive diseases	Male	1-4 years	95+ years	0	vegetables adjusted(g)
Other digestive diseases	Male	1-4 years	95+ years	-1	Healthcare access and quality index
Other digestive diseases	Female	1-4 years	95+ years	1	Alcohol (liters per capita)
Other digestive diseases	Female	1-4 years	95+ years	1	Cumulative Cigarettes (10 Years)
Other digestive diseases	Female	1-4 years	95+ years	1	Cumulative Cigarettes (5 Years)
Other digestive diseases	Female	1-4 years	95+ years	1	Diabetes Age-Standardized Prevalence (proportion)
Other digestive diseases	Female	1-4 years	95+ years	-1	Education (years per capita)
Other digestive diseases	Female	1-4 years	95+ years	-1	Health System Access 2 (unitless)
Other digestive diseases	Female	1-4 years	95+ years	-1	LDI (I$ per capita)
Other digestive diseases	Female	1-4 years	95+ years	1	Mean BMI
Other digestive diseases	Female	1-4 years	95+ years	-1	Sanitation (proportion with access)
Other digestive diseases	Female	1-4 years	95+ years	1	Smoking Prevalence
Other digestive diseases	Female	1-4 years	95+ years	-1	Improved Water Source (proportion with access)
Other digestive diseases	Female	1-4 years	95+ years	0	Socio-demographic Index
Other digestive diseases	Female	1-4 years	95+ years	-1	fruits adjusted(g)
Other digestive diseases	Female	1-4 years	95+ years	1	red meats adjusted(g)
Other digestive diseases	Female	1-4 years	95+ years	1	saturated fats adjusted(percent)

Cause	Sex	Age start	Age end	Direction	Covariate
Other digestive diseases	Female	1-4 years	95+ years	0	vegetables adjusted(g)
Other digestive diseases	Female	1-4 years	95+ years	-1	Healthcare access and quality index
Epilepsy	Male	28-364 days	95+ years	1	Cumulative Cigarettes (10 Years)
Epilepsy	Male	28-364 days	95+ years	1	Cumulative Cigarettes (5 Years)
Epilepsy	Male	28-364 days	95+ years	-1	Education (years per capita)
Epilepsy	Male	28-364 days	95+ years	-1	LDI (I$ per capita)
Epilepsy	Male	28-364 days	95+ years	1	Mean BMI
Epilepsy	Male	28-364 days	95+ years	1	Cholesterol (total, mean per capita)
Epilepsy	Male	28-364 days	95+ years	1	Systolic Blood Pressure (mmHg)
Epilepsy	Male	28-364 days	95+ years	1	Pig Meat (kg per capita)
Epilepsy	Male	28-364 days	95+ years	1	Pigs (per capita)
Epilepsy	Male	28-364 days	95+ years	1	Log-transformed SEV scalar: Epilepsy
Epilepsy	Male	28-364 days	95+ years	-1	Socio-demographic Index
Epilepsy	Male	28-364 days	95+ years	-1	Healthcare access and quality index
Epilepsy	Female	28-364 days	95+ years	1	Cumulative Cigarettes (10 Years)
Epilepsy	Female	28-364 days	95+ years	1	Cumulative Cigarettes (5 Years)
Epilepsy	Female	28-364 days	95+ years	-1	Education (years per capita)
Epilepsy	Female	28-364 days	95+ years	-1	LDI (I$ per capita)
Epilepsy	Female	28-364 days	95+ years	1	Mean BMI
Epilepsy	Female	28-364 days	95+ years	1	Cholesterol (total, mean per capita)
Epilepsy	Female	28-364 days	95+ years	1	Systolic Blood Pressure (mmHg)
Epilepsy	Female	28-364 days	95+ years	1	Pig Meat (kg per capita)
Epilepsy	Female	28-364 days	95+ years	1	Pigs (per capita)
Epilepsy	Female	28-364 days	95+ years	1	Log-transformed SEV scalar: Epilepsy
Epilepsy	Female	28-364 days	95+ years	-1	Socio-demographic Index
Epilepsy	Female	28-364 days	95+ years	-1	Healthcare access and quality index
Multiple sclerosis	Female	20-24 years	95+ years	1	Absolute value of average latitude
Multiple sclerosis	Female	20-24 years	95+ years	1	Cumulative Cigarettes (10 Years)
Multiple sclerosis	Female	20-24 years	95+ years	1	Cumulative Cigarettes (5 Years)
Multiple sclerosis	Female	20-24 years	95+ years	-1	Education (years per capita)
Multiple sclerosis	Female	20-24 years	95+ years	-1	LDI (I$ per capita)
Multiple sclerosis	Female	20-24 years	95+ years	1	Cholesterol (total, mean per capita)
Multiple sclerosis	Female	20-24 years	95+ years	1	Smoking Prevalence
Multiple sclerosis	Female	20-24 years	95+ years	1	Socio-demographic Index
Multiple sclerosis	Female	20-24 years	95+ years	-1	Healthcare access and quality index
Multiple sclerosis	Male	20-24 years	95+ years	1	Absolute value of average latitude
Multiple sclerosis	Male	20-24 years	95+ years	1	Cumulative Cigarettes (10 Years)
Multiple sclerosis	Male	20-24 years	95+ years	1	Cumulative Cigarettes (5 Years)
Multiple sclerosis	Male	20-24 years	95+ years	-1	Education (years per capita)
Multiple sclerosis	Male	20-24 years	95+ years	-1	LDI (I$ per capita)
Multiple sclerosis	Male	20-24 years	95+ years	1	Cholesterol (total, mean per capita)
Multiple sclerosis	Male	20-24 years	95+ years	1	Smoking Prevalence
Multiple sclerosis	Male	20-24 years	95+ years	1	Socio-demographic Index
Multiple sclerosis	Male	20-24 years	95+ years	-1	Healthcare access and quality index
Motor neuron disease	Male	0-6 days	95+ years	1	Absolute value of average latitude
Motor neuron disease	Male	0-6 days	95+ years	1	Asbestos production (kg) per capita
Motor neuron disease	Male	0-6 days	95+ years	0	Education (years per capita)
Motor neuron disease	Male	0-6 days	95+ years	0	LDI (I$ per capita)
Motor neuron disease	Male	0-6 days	95+ years	0	Cholesterol (total, mean per capita)
Motor neuron disease	Male	0-6 days	95+ years	0	Sanitation (proportion with access)
Motor neuron disease	Male	0-6 days	95+ years	0	Improved Water Source (proportion with access)
Motor neuron disease	Male	0-6 days	95+ years	0	Socio-demographic Index
Motor neuron disease	Male	0-6 days	95+ years	0	fruits adjusted(g)
Motor neuron disease	Male	0-6 days	95+ years	-1	Healthcare access and quality index
Motor neuron disease	Female	0-6 days	95+ years	1	Absolute value of average latitude
Motor neuron disease	Female	0-6 days	95+ years	1	Asbestos production (kg) per capita
Motor neuron disease	Female	0-6 days	95+ years	0	Education (years per capita)
Motor neuron disease	Female	0-6 days	95+ years	0	LDI (I$ per capita)
Motor neuron disease	Female	0-6 days	95+ years	0	Cholesterol (total, mean per capita)
Motor neuron disease	Female	0-6 days	95+ years	0	Sanitation (proportion with access)
Motor neuron disease	Female	0-6 days	95+ years	0	Improved Water Source (proportion with access)
Motor neuron disease	Female	0-6 days	95+ years	0	Socio-demographic Index
Motor neuron disease	Female	0-6 days	95+ years	0	fruits adjusted(g)
Motor neuron disease	Female	0-6 days	95+ years	-1	Healthcare access and quality index
Other neurological disorders	Male	28-364 days	95+ years	1	Alcohol (liters per capita)
Other neurological disorders	Male	28-364 days	95+ years	1	Animal Fats (kcal per capita)
Other neurological disorders	Male	28-364 days	95+ years	1	Cumulative Cigarettes (10 Years)
Other neurological disorders	Male	28-364 days	95+ years	1	Cumulative Cigarettes (5 Years)
Other neurological disorders	Male	28-364 days	95+ years	-1	Education (years per capita)
Other neurological disorders	Male	28-364 days	95+ years	-1	LDI (I$ per capita)
Other neurological disorders	Male	28-364 days	95+ years	1	Underweight (proportion <2SD weight for age, <5 years)
Other neurological disorders	Male	28-364 days	95+ years	1	Mean BMI
Other neurological disorders	Male	28-364 days	95+ years	1	Cholesterol (total, mean per capita)
Other neurological disorders	Male	28-364 days	95+ years	1	Systolic Blood Pressure (mmHg)
Other neurological disorders	Male	28-364 days	95+ years	1	Pig Meat (kg per capita)
Other neurological disorders	Male	28-364 days	95+ years	1	Population Density (over 1000 ppl/sqkm, proportion)
Other neurological disorders	Male	28-364 days	95+ years	1	Smoking Prevalence
Other neurological disorders	Male	28-364 days	95+ years	0	Socio-demographic Index
Other neurological disorders	Male	28-364 days	95+ years	-1	fruits adjusted(g)
Other neurological disorders	Male	28-364 days	95+ years	1	red meats adjusted(g)
Other neurological disorders	Male	28-364 days	95+ years	-1	Healthcare access and quality index
Other neurological disorders	Female	28-364 days	95+ years	1	Alcohol (liters per capita)
Other neurological disorders	Female	28-364 days	95+ years	1	Animal Fats (kcal per capita)
Other neurological disorders	Female	28-364 days	95+ years	1	Cumulative Cigarettes (10 Years)
Other neurological disorders	Female	28-364 days	95+ years	1	Cumulative Cigarettes (5 Years)
Other neurological disorders	Female	28-364 days	95+ years	-1	Education (years per capita)
Other neurological disorders	Female	28-364 days	95+ years	-1	LDI (I$ per capita)
Other neurological disorders	Female	28-364 days	95+ years	1	Underweight (proportion <2SD weight for age, <5 years)
Other neurological disorders	Female	28-364 days	95+ years	1	Mean BMI
Other neurological disorders	Female	28-364 days	95+ years	1	Cholesterol (total, mean per capita)
Other neurological disorders	Female	28-364 days	95+ years	1	Systolic Blood Pressure (mmHg)
Other neurological disorders	Female	28-364 days	95+ years	1	Pig Meat (kg per capita)
Other neurological disorders	Female	28-364 days	95+ years	1	Population Density (over 1000 ppl/sqkm, proportion)
Other neurological disorders	Female	28-364 days	95+ years	1	Smoking Prevalence
Other neurological disorders	Female	28-364 days	95+ years	0	Socio-demographic Index

Cause	Sex	Age start	Age end	Direction	Covariate
Other neurological disorders	Female	28-364 days	95+ years	-1	fruits adjusted(g)
Other neurological disorders	Female	28-364 days	95+ years	1	red meats adjusted(g)
Other neurological disorders	Female	28-364 days	95+ years	-1	Healthcare access and quality index
Schizophrenia	Male	25-29 years	95+ years	0	Alcohol (liters per capita)
Schizophrenia	Male	25-29 years	95+ years	0	Cumulative Cigarettes (20 Years)
Schizophrenia	Male	25-29 years	95+ years	0	Education (years per capita)
Schizophrenia	Male	25-29 years	95+ years	0	Health System Access 2 (unitless)
Schizophrenia	Male	25-29 years	95+ years	0	LDI (I$ per capita)
Schizophrenia	Male	25-29 years	95+ years	1	Underweight (proportion <2SD weight for age, <5 years)
Schizophrenia	Male	25-29 years	95+ years	0	Smoking Prevalence
Schizophrenia	Male	25-29 years	95+ years	0	Socio-demographic Index
Schizophrenia	Female	25-29 years	95+ years	0	Alcohol (liters per capita)
Schizophrenia	Female	25-29 years	95+ years	0	Cumulative Cigarettes (20 Years)
Schizophrenia	Female	25-29 years	95+ years	0	Education (years per capita)
Schizophrenia	Female	25-29 years	95+ years	0	Health System Access 2 (unitless)
Schizophrenia	Female	25-29 years	95+ years	0	LDI (I$ per capita)
Schizophrenia	Female	25-29 years	95+ years	1	Underweight (proportion <2SD weight for age, <5 years)
Schizophrenia	Female	25-29 years	95+ years	0	Smoking Prevalence
Schizophrenia	Female	25-29 years	95+ years	0	Socio-demographic Index
Alcohol use disorders	Male	15-19 years	95+ years	1	Alcohol (liters per capita)
Alcohol use disorders	Male	15-19 years	95+ years	0	Cumulative Cigarettes (10 Years)
Alcohol use disorders	Male	15-19 years	95+ years	-1	Education (years per capita)
Alcohol use disorders	Male	15-19 years	95+ years	-1	Health System Access 2 (unitless)
Alcohol use disorders	Male	15-19 years	95+ years	-1	LDI (I$ per capita)
Alcohol use disorders	Male	15-19 years	95+ years	-1	Religion (binary, >50% Muslim)
Alcohol use disorders	Male	15-19 years	95+ years	0	Smoking Prevalence
Alcohol use disorders	Male	15-19 years	95+ years	1	Prevalence of binge drinking
Alcohol use disorders	Male	15-19 years	95+ years	0	Socio-demographic Index
Alcohol use disorders	Male	15-19 years	95+ years	-1	Healthcare access and quality index
Alcohol use disorders	Female	15-19 years	95+ years	1	Alcohol (liters per capita)
Alcohol use disorders	Female	15-19 years	95+ years	0	Cumulative Cigarettes (10 Years)
Alcohol use disorders	Female	15-19 years	95+ years	-1	Education (years per capita)
Alcohol use disorders	Female	15-19 years	95+ years	-1	Health System Access 2 (unitless)
Alcohol use disorders	Female	15-19 years	95+ years	-1	LDI (I$ per capita)
Alcohol use disorders	Female	15-19 years	95+ years	-1	Religion (binary, >50% Muslim)
Alcohol use disorders	Female	15-19 years	95+ years	0	Smoking Prevalence
Alcohol use disorders	Female	15-19 years	95+ years	1	Prevalence of binge drinking
Alcohol use disorders	Female	15-19 years	95+ years	0	Socio-demographic Index
Alcohol use disorders	Female	15-19 years	95+ years	-1	Healthcare access and quality index
Drug use disorders	Male	15-19 years	95+ years	1	Alcohol (liters per capita)
Drug use disorders	Male	15-19 years	95+ years	1	Cumulative Cigarettes (10 Years)
Drug use disorders	Male	15-19 years	95+ years	1	Cumulative Cigarettes (5 Years)
Drug use disorders	Male	15-19 years	95+ years	0	Education (years per capita)
Drug use disorders	Male	15-19 years	95+ years	0	LDI (I$ per capita)
Drug use disorders	Male	15-19 years	95+ years	1	Opium Cultivation (binary)
Drug use disorders	Male	15-19 years	95+ years	1	Smoking Prevalence
Drug use disorders	Male	15-19 years	95+ years	0	Socio-demographic Index
Drug use disorders	Male	15-19 years	95+ years	-1	Healthcare access and quality index
Drug use disorders	Female	15-19 years	95+ years	1	Alcohol (liters per capita)
Drug use disorders	Female	15-19 years	95+ years	1	Cumulative Cigarettes (10 Years)
Drug use disorders	Female	15-19 years	95+ years	1	Cumulative Cigarettes (5 Years)
Drug use disorders	Female	15-19 years	95+ years	0	Education (years per capita)
Drug use disorders	Female	15-19 years	95+ years	0	LDI (I$ per capita)
Drug use disorders	Female	15-19 years	95+ years	1	Opium Cultivation (binary)
Drug use disorders	Female	15-19 years	95+ years	1	Smoking Prevalence
Drug use disorders	Female	15-19 years	95+ years	0	Socio-demographic Index
Drug use disorders	Female	15-19 years	95+ years	-1	Healthcare access and quality index
Drug use disorders	Male	0-6 days	7-27 days	1	Alcohol (liters per capita)
Drug use disorders	Male	0-6 days	7-27 days	1	Cumulative Cigarettes (10 Years)
Drug use disorders	Male	0-6 days	7-27 days	1	Cumulative Cigarettes (5 Years)
Drug use disorders	Male	0-6 days	7-27 days	0	Education (years per capita)
Drug use disorders	Male	0-6 days	7-27 days	0	Health System Access 2 (unitless)
Drug use disorders	Male	0-6 days	7-27 days	0	LDI (I$ per capita)
Drug use disorders	Male	0-6 days	7-27 days	1	Opium Cultivation (binary)
Drug use disorders	Male	0-6 days	7-27 days	1	Smoking Prevalence
Drug use disorders	Male	0-6 days	7-27 days	0	Socio-demographic Index
Drug use disorders	Male	0-6 days	7-27 days	-1	Healthcare access and quality index
Drug use disorders	Female	0-6 days	7-27 days	1	Alcohol (liters per capita)
Drug use disorders	Female	0-6 days	7-27 days	1	Cumulative Cigarettes (10 Years)
Drug use disorders	Female	0-6 days	7-27 days	1	Cumulative Cigarettes (5 Years)
Drug use disorders	Female	0-6 days	7-27 days	0	Education (years per capita)
Drug use disorders	Female	0-6 days	7-27 days	0	Health System Access 2 (unitless)
Drug use disorders	Female	0-6 days	7-27 days	0	LDI (I$ per capita)
Drug use disorders	Female	0-6 days	7-27 days	1	Opium Cultivation (binary)
Drug use disorders	Female	0-6 days	7-27 days	1	Smoking Prevalence
Drug use disorders	Female	0-6 days	7-27 days	0	Socio-demographic Index
Drug use disorders	Female	0-6 days	7-27 days	-1	Healthcare access and quality index
Opioid use disorders	Male	15-19 years	95+ years	1	Alcohol (liters per capita)
Opioid use disorders	Male	15-19 years	95+ years	1	Cumulative Cigarettes (10 Years)
Opioid use disorders	Male	15-19 years	95+ years	1	Cumulative Cigarettes (5 Years)
Opioid use disorders	Male	15-19 years	95+ years	0	Education (years per capita)
Opioid use disorders	Male	15-19 years	95+ years	0	LDI (I$ per capita)
Opioid use disorders	Male	15-19 years	95+ years	1	Opium Cultivation (binary)
Opioid use disorders	Male	15-19 years	95+ years	1	Smoking Prevalence
Opioid use disorders	Male	15-19 years	95+ years	0	Socio-demographic Index
Opioid use disorders	Male	15-19 years	95+ years	-1	Healthcare access and quality index
Opioid use disorders	Female	15-19 years	95+ years	1	Alcohol (liters per capita)
Opioid use disorders	Female	15-19 years	95+ years	1	Cumulative Cigarettes (10 Years)
Opioid use disorders	Female	15-19 years	95+ years	1	Cumulative Cigarettes (5 Years)
Opioid use disorders	Female	15-19 years	95+ years	0	Education (years per capita)
Opioid use disorders	Female	15-19 years	95+ years	0	LDI (I$ per capita)
Opioid use disorders	Female	15-19 years	95+ years	1	Opium Cultivation (binary)
Opioid use disorders	Female	15-19 years	95+ years	1	Smoking Prevalence
Opioid use disorders	Female	15-19 years	95+ years	0	Socio-demographic Index
Opioid use disorders	Female	15-19 years	95+ years	-1	Healthcare access and quality index

Cause	Sex	Age start	Age end	Direction	Covariate
Opioid use disorders	Male	0-6 days	7-27 days	1	Alcohol (liters per capita)
Opioid use disorders	Male	0-6 days	7-27 days	1	Cumulative Cigarettes (10 Years)
Opioid use disorders	Male	0-6 days	7-27 days	1	Cumulative Cigarettes (5 Years)
Opioid use disorders	Male	0-6 days	7-27 days	0	Education (years per capita)
Opioid use disorders	Male	0-6 days	7-27 days	0	LDI (I$ per capita)
Opioid use disorders	Male	0-6 days	7-27 days	1	Opium Cultivation (binary)
Opioid use disorders	Male	0-6 days	7-27 days	1	Smoking Prevalence
Opioid use disorders	Male	0-6 days	7-27 days	0	Socio-demographic Index
Opioid use disorders	Male	0-6 days	7-27 days	-1	Healthcare access and quality index
Opioid use disorders	Female	0-6 days	7-27 days	1	Alcohol (liters per capita)
Opioid use disorders	Female	0-6 days	7-27 days	1	Cumulative Cigarettes (10 Years)
Opioid use disorders	Female	0-6 days	7-27 days	1	Cumulative Cigarettes (5 Years)
Opioid use disorders	Female	0-6 days	7-27 days	0	Education (years per capita)
Opioid use disorders	Female	0-6 days	7-27 days	0	LDI (I$ per capita)
Opioid use disorders	Female	0-6 days	7-27 days	1	Opium Cultivation (binary)
Opioid use disorders	Female	0-6 days	7-27 days	1	Smoking Prevalence
Opioid use disorders	Female	0-6 days	7-27 days	0	Socio-demographic Index
Opioid use disorders	Female	0-6 days	7-27 days	-1	Healthcare access and quality index
Opioid use disorders	Male	0-6 days	7-27 days	0	Health System Access 2 (unitless)
Opioid use disorders	Female	0-6 days	7-27 days	0	Health System Access 2 (unitless)
Cocaine use disorders	Male	15-19 years	95+ years	1	Alcohol (liters per capita)
Cocaine use disorders	Male	15-19 years	95+ years	1	Cumulative Cigarettes (10 Years)
Cocaine use disorders	Male	15-19 years	95+ years	1	Cumulative Cigarettes (5 Years)
Cocaine use disorders	Male	15-19 years	95+ years	0	Education (years per capita)
Cocaine use disorders	Male	15-19 years	95+ years	0	LDI (I$ per capita)
Cocaine use disorders	Male	15-19 years	95+ years	1	Smoking Prevalence
Cocaine use disorders	Male	15-19 years	95+ years	1	Socio-demographic Index
Cocaine use disorders	Male	15-19 years	95+ years	-1	Healthcare access and quality index
Cocaine use disorders	Female	15-19 years	95+ years	1	Alcohol (liters per capita)
Cocaine use disorders	Female	15-19 years	95+ years	1	Cumulative Cigarettes (10 Years)
Cocaine use disorders	Female	15-19 years	95+ years	1	Cumulative Cigarettes (5 Years)
Cocaine use disorders	Female	15-19 years	95+ years	0	Education (years per capita)
Cocaine use disorders	Female	15-19 years	95+ years	0	LDI (I$ per capita)
Cocaine use disorders	Female	15-19 years	95+ years	1	Smoking Prevalence
Cocaine use disorders	Female	15-19 years	95+ years	1	Socio-demographic Index
Cocaine use disorders	Female	15-19 years	95+ years	-1	Healthcare access and quality index
Amphetamine use disorders	Male	15-19 years	95+ years	1	Alcohol (liters per capita)
Amphetamine use disorders	Male	15-19 years	95+ years	1	Cumulative Cigarettes (10 Years)
Amphetamine use disorders	Male	15-19 years	95+ years	1	Cumulative Cigarettes (5 Years)
Amphetamine use disorders	Male	15-19 years	95+ years	0	Education (years per capita)
Amphetamine use disorders	Male	15-19 years	95+ years	0	LDI (I$ per capita)
Amphetamine use disorders	Male	15-19 years	95+ years	1	Smoking Prevalence
Amphetamine use disorders	Male	15-19 years	95+ years	1	Socio-demographic Index
Amphetamine use disorders	Male	15-19 years	95+ years	-1	Healthcare access and quality index
Amphetamine use disorders	Female	15-19 years	95+ years	1	Alcohol (liters per capita)
Amphetamine use disorders	Female	15-19 years	95+ years	1	Cumulative Cigarettes (10 Years)
Amphetamine use disorders	Female	15-19 years	95+ years	1	Cumulative Cigarettes (5 Years)
Amphetamine use disorders	Female	15-19 years	95+ years	0	Education (years per capita)
Amphetamine use disorders	Female	15-19 years	95+ years	0	LDI (I$ per capita)
Amphetamine use disorders	Female	15-19 years	95+ years	1	Smoking Prevalence
Amphetamine use disorders	Female	15-19 years	95+ years	1	Socio-demographic Index
Amphetamine use disorders	Female	15-19 years	95+ years	-1	Healthcare access and quality index
Other drug use disorders	Male	15-19 years	95+ years	1	Alcohol (liters per capita)
Other drug use disorders	Male	15-19 years	95+ years	1	Cumulative Cigarettes (10 Years)
Other drug use disorders	Male	15-19 years	95+ years	1	Cumulative Cigarettes (5 Years)
Other drug use disorders	Male	15-19 years	95+ years	0	Education (years per capita)
Other drug use disorders	Male	15-19 years	95+ years	0	LDI (I$ per capita)
Other drug use disorders	Male	15-19 years	95+ years	1	Smoking Prevalence
Other drug use disorders	Male	15-19 years	95+ years	0	Socio-demographic Index
Other drug use disorders	Male	15-19 years	95+ years	-1	Healthcare access and quality index
Other drug use disorders	Female	15-19 years	95+ years	1	Alcohol (liters per capita)
Other drug use disorders	Female	15-19 years	95+ years	1	Cumulative Cigarettes (10 Years)
Other drug use disorders	Female	15-19 years	95+ years	1	Cumulative Cigarettes (5 Years)
Other drug use disorders	Female	15-19 years	95+ years	0	Education (years per capita)
Other drug use disorders	Female	15-19 years	95+ years	0	LDI (I$ per capita)
Other drug use disorders	Female	15-19 years	95+ years	1	Smoking Prevalence
Other drug use disorders	Female	15-19 years	95+ years	0	Socio-demographic Index
Other drug use disorders	Female	15-19 years	95+ years	-1	Healthcare access and quality index
Eating disorders	Male	5-9 years	45-49 years	1	Education (years per capita)
Eating disorders	Male	5-9 years	45-49 years	1	LDI (I$ per capita)
Eating disorders	Male	5-9 years	45-49 years	-1	Underweight (proportion <2SD weight for age, <5 years)
Eating disorders	Male	5-9 years	45-49 years	1	Sanitation (proportion with access)
Eating disorders	Male	5-9 years	45-49 years	1	Maternal education (years per capita)
Eating disorders	Male	5-9 years	45-49 years	1	Socio-demographic Index
Eating disorders	Male	5-9 years	45-49 years	-1	Healthcare access and quality index
Eating disorders	Female	5-9 years	45-49 years	1	Education (years per capita)
Eating disorders	Female	5-9 years	45-49 years	1	LDI (I$ per capita)
Eating disorders	Female	5-9 years	45-49 years	-1	Underweight (proportion <2SD weight for age, <5 years)
Eating disorders	Female	5-9 years	45-49 years	1	Sanitation (proportion with access)
Eating disorders	Female	5-9 years	45-49 years	1	Maternal education (years per capita)
Eating disorders	Female	5-9 years	45-49 years	1	Socio-demographic Index
Eating disorders	Female	5-9 years	45-49 years	-1	Healthcare access and quality index
Anorexia nervosa	Male	5-9 years	45-49 years	1	Education (years per capita)
Anorexia nervosa	Male	5-9 years	45-49 years	1	LDI (I$ per capita)
Anorexia nervosa	Male	5-9 years	45-49 years	-1	Underweight (proportion <2SD weight for age, <5 years)
Anorexia nervosa	Male	5-9 years	45-49 years	1	Sanitation (proportion with access)
Anorexia nervosa	Male	5-9 years	45-49 years	1	Maternal education (years per capita)
Anorexia nervosa	Male	5-9 years	45-49 years	1	Socio-demographic Index
Anorexia nervosa	Male	5-9 years	45-49 years	-1	Healthcare access and quality index
Anorexia nervosa	Female	5-9 years	45-49 years	1	Education (years per capita)
Anorexia nervosa	Female	5-9 years	45-49 years	1	LDI (I$ per capita)
Anorexia nervosa	Female	5-9 years	45-49 years	-1	Underweight (proportion <2SD weight for age, <5 years)
Anorexia nervosa	Female	5-9 years	45-49 years	1	Sanitation (proportion with access)
Anorexia nervosa	Female	5-9 years	45-49 years	1	Maternal education (years per capita)
Anorexia nervosa	Female	5-9 years	45-49 years	1	Socio-demographic Index

Cause	Sex	Age start	Age end	Direction	Covariate
Anorexia nervosa	Female	5-9 years	45-49 years	-1	Healthcare access and quality index
Bulimia nervosa	Male	5-9 years	45-49 years	1	Education (years per capita)
Bulimia nervosa	Male	5-9 years	45-49 years	1	LDI (I$ per capita)
Bulimia nervosa	Male	5-9 years	45-49 years	-1	Underweight (proportion <2SD weight for age, <5 years)
Bulimia nervosa	Male	5-9 years	45-49 years	1	Sanitation (proportion with access)
Bulimia nervosa	Male	5-9 years	45-49 years	1	Maternal education (years per capita)
Bulimia nervosa	Male	5-9 years	45-49 years	1	Socio-demographic Index
Bulimia nervosa	Male	5-9 years	45-49 years	-1	Healthcare access and quality index
Bulimia nervosa	Female	5-9 years	45-49 years	1	Education (years per capita)
Bulimia nervosa	Female	5-9 years	45-49 years	1	LDI (I$ per capita)
Bulimia nervosa	Female	5-9 years	45-49 years	-1	Underweight (proportion <2SD weight for age, <5 years)
Bulimia nervosa	Female	5-9 years	45-49 years	1	Sanitation (proportion with access)
Bulimia nervosa	Female	5-9 years	45-49 years	1	Maternal education (years per capita)
Bulimia nervosa	Female	5-9 years	45-49 years	1	Socio-demographic Index
Bulimia nervosa	Female	5-9 years	45-49 years	-1	Healthcare access and quality index
Diabetes mellitus	Female	0-6 days	20-24 years	0	Animal Fats (kcal per capita)
Diabetes mellitus	Female	0-6 days	20-24 years	1	Diabetes Fasting Plasma Glucose (mmol/L)
Diabetes mellitus	Female	0-6 days	20-24 years	1	Diabetes Age-Standardized Prevalence (proportion)
Diabetes mellitus	Female	0-6 days	20-24 years	0	Education (years per capita)
Diabetes mellitus	Female	0-6 days	20-24 years	0	LDI (I$ per capita)
Diabetes mellitus	Female	0-6 days	20-24 years	0	Mean BMI
Diabetes mellitus	Female	0-6 days	20-24 years	0	Cholesterol (total, mean per capita)
Diabetes mellitus	Female	0-6 days	20-24 years	0	Systolic Blood Pressure (mmHg)
Diabetes mellitus	Female	0-6 days	20-24 years	0	fruits adjusted(g)
Diabetes mellitus	Female	0-6 days	20-24 years	0	vegetables adjusted(g)
Diabetes mellitus	Female	0-6 days	20-24 years	0	whole grains adjusted(g)
Diabetes mellitus	Female	0-6 days	20-24 years	0	energy unadjusted(kcal)
Diabetes mellitus	Female	0-6 days	20-24 years	0	Healthcare access and quality index
Diabetes mellitus	Male	0-6 days	20-24 years	0	Animal Fats (kcal per capita)
Diabetes mellitus	Male	0-6 days	20-24 years	1	Diabetes Fasting Plasma Glucose (mmol/L)
Diabetes mellitus	Male	0-6 days	20-24 years	1	Diabetes Age-Standardized Prevalence (proportion)
Diabetes mellitus	Male	0-6 days	20-24 years	0	Education (years per capita)
Diabetes mellitus	Male	0-6 days	20-24 years	0	LDI (I$ per capita)
Diabetes mellitus	Male	0-6 days	20-24 years	0	Mean BMI
Diabetes mellitus	Male	0-6 days	20-24 years	0	Cholesterol (total, mean per capita)
Diabetes mellitus	Male	0-6 days	20-24 years	0	Systolic Blood Pressure (mmHg)
Diabetes mellitus	Male	0-6 days	20-24 years	0	fruits adjusted(g)
Diabetes mellitus	Male	0-6 days	20-24 years	0	vegetables adjusted(g)
Diabetes mellitus	Male	0-6 days	20-24 years	0	whole grains adjusted(g)
Diabetes mellitus	Male	0-6 days	20-24 years	0	energy unadjusted(kcal)
Diabetes mellitus	Male	0-6 days	20-24 years	0	Healthcare access and quality index
Diabetes mellitus	Male	25-29 years	95+ years	0	Animal Fats (kcal per capita)
Diabetes mellitus	Male	25-29 years	95+ years	1	Diabetes Fasting Plasma Glucose (mmol/L)
Diabetes mellitus	Male	25-29 years	95+ years	1	Diabetes Age-Standardized Prevalence (proportion)
Diabetes mellitus	Male	25-29 years	95+ years	0	Education (years per capita)
Diabetes mellitus	Male	25-29 years	95+ years	0	LDI (I$ per capita)
Diabetes mellitus	Male	25-29 years	95+ years	0	Mean BMI
Diabetes mellitus	Male	25-29 years	95+ years	0	Cholesterol (total, mean per capita)
Diabetes mellitus	Male	25-29 years	95+ years	0	Systolic Blood Pressure (mmHg)
Diabetes mellitus	Male	25-29 years	95+ years	0	fruits adjusted(g)
Diabetes mellitus	Male	25-29 years	95+ years	0	vegetables adjusted(g)
Diabetes mellitus	Male	25-29 years	95+ years	0	whole grains adjusted(g)
Diabetes mellitus	Male	25-29 years	95+ years	0	energy unadjusted(kcal)
Diabetes mellitus	Male	25-29 years	95+ years	0	Healthcare access and quality index
Diabetes mellitus	Female	25-29 years	95+ years	0	Animal Fats (kcal per capita)
Diabetes mellitus	Female	25-29 years	95+ years	1	Diabetes Fasting Plasma Glucose (mmol/L)
Diabetes mellitus	Female	25-29 years	95+ years	1	Diabetes Age-Standardized Prevalence (proportion)
Diabetes mellitus	Female	25-29 years	95+ years	0	Education (years per capita)
Diabetes mellitus	Female	25-29 years	95+ years	0	LDI (I$ per capita)
Diabetes mellitus	Female	25-29 years	95+ years	0	Mean BMI
Diabetes mellitus	Female	25-29 years	95+ years	0	Cholesterol (total, mean per capita)
Diabetes mellitus	Female	25-29 years	95+ years	0	Systolic Blood Pressure (mmHg)
Diabetes mellitus	Female	25-29 years	95+ years	0	fruits adjusted(g)
Diabetes mellitus	Female	25-29 years	95+ years	0	vegetables adjusted(g)
Diabetes mellitus	Female	25-29 years	95+ years	0	whole grains adjusted(g)
Diabetes mellitus	Female	25-29 years	95+ years	0	energy unadjusted(kcal)
Diabetes mellitus	Female	25-29 years	95+ years	0	Healthcare access and quality index
Acute glomerulonephritis	Female	28-364 days	95+ years	1	Diabetes Age-Standardized Prevalence (proportion)
Acute glomerulonephritis	Female	28-364 days	95+ years	-1	Education (years per capita)
Acute glomerulonephritis	Female	28-364 days	95+ years	-1	LDI (I$ per capita)
Acute glomerulonephritis	Female	28-364 days	95+ years	1	Systolic Blood Pressure (mmHg)
Acute glomerulonephritis	Female	28-364 days	95+ years	-1	Sanitation (proportion with access)
Acute glomerulonephritis	Female	28-364 days	95+ years	-1	Improved Water Source (proportion with access)
Acute glomerulonephritis	Female	28-364 days	95+ years	-1	Socio-demographic Index
Acute glomerulonephritis	Female	28-364 days	95+ years	-1	Healthcare access and quality index
Acute glomerulonephritis	Male	28-364 days	95+ years	1	Diabetes Age-Standardized Prevalence (proportion)
Acute glomerulonephritis	Male	28-364 days	95+ years	-1	Education (years per capita)
Acute glomerulonephritis	Male	28-364 days	95+ years	-1	LDI (I$ per capita)
Acute glomerulonephritis	Male	28-364 days	95+ years	1	Systolic Blood Pressure (mmHg)
Acute glomerulonephritis	Male	28-364 days	95+ years	-1	Sanitation (proportion with access)
Acute glomerulonephritis	Male	28-364 days	95+ years	-1	Improved Water Source (proportion with access)
Acute glomerulonephritis	Male	28-364 days	95+ years	-1	Socio-demographic Index
Acute glomerulonephritis	Male	28-364 days	95+ years	-1	Healthcare access and quality index
Chronic kidney disease	Female	28-364 days	95+ years	1	Diabetes Fasting Plasma Glucose (mmol/L)
Chronic kidney disease	Female	28-364 days	95+ years	1	Diabetes Age-Standardized Prevalence (proportion)
Chronic kidney disease	Female	28-364 days	95+ years	-1	Education (years per capita)
Chronic kidney disease	Female	28-364 days	95+ years	-1	LDI (I$ per capita)
Chronic kidney disease	Female	28-364 days	95+ years	1	Mean BMI
Chronic kidney disease	Female	28-364 days	95+ years	1	Cholesterol (total, mean per capita)
Chronic kidney disease	Female	28-364 days	95+ years	1	Systolic Blood Pressure (mmHg)
Chronic kidney disease	Female	28-364 days	95+ years	0	Socio-demographic Index
Chronic kidney disease	Female	28-364 days	95+ years	0	red meats adjusted(g)
Chronic kidney disease	Female	28-364 days	95+ years	0	whole grains adjusted(g)
Chronic kidney disease	Female	28-364 days	95+ years	1	energy unadjusted(kcal)
Chronic kidney disease	Female	28-364 days	95+ years	-1	Healthcare access and quality index

Cause	Sex	Age start	Age end	Direction	Covariate
Chronic kidney disease	Male	28-364 days	95+ years	1	Diabetes Fasting Plasma Glucose (mmol/L)
Chronic kidney disease	Male	28-364 days	95+ years	1	Diabetes Age-Standardized Prevalence (proportion)
Chronic kidney disease	Male	28-364 days	95+ years	-1	Education (years per capita)
Chronic kidney disease	Male	28-364 days	95+ years	-1	LDI (I$ per capita)
Chronic kidney disease	Male	28-364 days	95+ years	1	Mean BMI
Chronic kidney disease	Male	28-364 days	95+ years	1	Cholesterol (total, mean per capita)
Chronic kidney disease	Male	28-364 days	95+ years	1	Systolic Blood Pressure (mmHg)
Chronic kidney disease	Male	28-364 days	95+ years	0	Socio-demographic Index
Chronic kidney disease	Male	28-364 days	95+ years	0	red meats adjusted(g)
Chronic kidney disease	Male	28-364 days	95+ years	0	whole grains adjusted(g)
Chronic kidney disease	Male	28-364 days	95+ years	1	energy unadjusted(kcal)
Chronic kidney disease	Male	28-364 days	95+ years	-1	Healthcare access and quality index
Urinary diseases and male infertility	Male	0-6 days	95+ years	-1	Education (years per capita)
Urinary diseases and male infertility	Male	0-6 days	95+ years	-1	LDI (I$ per capita)
Urinary diseases and male infertility	Male	0-6 days	95+ years	1	Mean BMI
Urinary diseases and male infertility	Male	0-6 days	95+ years	0	Latitude Under 15 (proportion)
Urinary diseases and male infertility	Male	0-6 days	95+ years	0	Latitude 15 to 30 (proportion)
Urinary diseases and male infertility	Male	0-6 days	95+ years	0	Latitude 30 to 45 (proportion)
Urinary diseases and male infertility	Male	0-6 days	95+ years	0	Latitude Over 45 (proportion)
Urinary diseases and male infertility	Male	0-6 days	95+ years	0	Socio-demographic Index
Urinary diseases and male infertility	Male	0-6 days	95+ years	-1	Healthcare access and quality index
Urinary diseases and male infertility	Female	0-6 days	95+ years	-1	Education (years per capita)
Urinary diseases and male infertility	Female	0-6 days	95+ years	-1	LDI (I$ per capita)
Urinary diseases and male infertility	Female	0-6 days	95+ years	1	Mean BMI
Urinary diseases and male infertility	Female	0-6 days	95+ years	0	Latitude Under 15 (proportion)
Urinary diseases and male infertility	Female	0-6 days	95+ years	0	Latitude 15 to 30 (proportion)
Urinary diseases and male infertility	Female	0-6 days	95+ years	0	Latitude 30 to 45 (proportion)
Urinary diseases and male infertility	Female	0-6 days	95+ years	0	Latitude Over 45 (proportion)
Urinary diseases and male infertility	Female	0-6 days	95+ years	0	Socio-demographic Index
Urinary diseases and male infertility	Female	0-6 days	95+ years	-1	Healthcare access and quality index
Interstitial nephritis and urinary tract infections	Female	0-6 days	95+ years	-1	Education (years per capita)
Interstitial nephritis and urinary tract infections	Female	0-6 days	95+ years	-1	LDI (I$ per capita)
Interstitial nephritis and urinary tract infections	Female	0-6 days	95+ years	1	Sanitation (proportion with access)
Interstitial nephritis and urinary tract infections	Female	0-6 days	95+ years	0	Socio-demographic Index
Interstitial nephritis and urinary tract infections	Female	0-6 days	95+ years	-1	Healthcare access and quality index
Interstitial nephritis and urinary tract infections	Male	0-6 days	95+ years	-1	Education (years per capita)
Interstitial nephritis and urinary tract infections	Male	0-6 days	95+ years	-1	LDI (I$ per capita)
Interstitial nephritis and urinary tract infections	Male	0-6 days	95+ years	1	Sanitation (proportion with access)
Interstitial nephritis and urinary tract infections	Male	0-6 days	95+ years	-1	Health System Access (capped)
Interstitial nephritis and urinary tract infections	Male	0-6 days	95+ years	0	Socio-demographic Index
Interstitial nephritis and urinary tract infections	Male	0-6 days	95+ years	-1	Healthcare access and quality index
Urolithiasis	Female	5-9 years	95+ years	-1	Education (years per capita)
Urolithiasis	Female	5-9 years	95+ years	-1	LDI (I$ per capita)
Urolithiasis	Female	5-9 years	95+ years	1	90th percentile climatic temperature in the given country-year.
Urolithiasis	Female	5-9 years	95+ years	0	Socio-demographic Index
Urolithiasis	Female	5-9 years	95+ years	-1	fruits adjusted(g)
Urolithiasis	Female	5-9 years	95+ years	1	red meats adjusted(g)
Urolithiasis	Female	5-9 years	95+ years	-1	vegetables adjusted(g)
Urolithiasis	Female	5-9 years	95+ years	1	Healthcare access and quality index
Urolithiasis	Male	5-9 years	95+ years	-1	Education (years per capita)
Urolithiasis	Male	5-9 years	95+ years	-1	LDI (I$ per capita)
Urolithiasis	Male	5-9 years	95+ years	1	90th percentile climatic temperature in the given country-year.
Urolithiasis	Male	5-9 years	95+ years	0	Socio-demographic Index
Urolithiasis	Male	5-9 years	95+ years	-1	fruits adjusted(g)
Urolithiasis	Male	5-9 years	95+ years	1	red meats adjusted(g)
Urolithiasis	Male	5-9 years	95+ years	-1	vegetables adjusted(g)
Urolithiasis	Male	5-9 years	95+ years	-1	Healthcare access and quality index
Other urinary diseases	Male	0-6 days	95+ years	-1	Education (years per capita)
Other urinary diseases	Male	0-6 days	95+ years	-1	LDI (I$ per capita)
Other urinary diseases	Male	0-6 days	95+ years	1	Mean BMI
Other urinary diseases	Male	0-6 days	95+ years	0	Socio-demographic Index
Other urinary diseases	Male	0-6 days	95+ years	-1	Healthcare access and quality index
Other urinary diseases	Female	0-6 days	95+ years	1	Education (years per capita)
Other urinary diseases	Female	0-6 days	95+ years	1	LDI (I$ per capita)
Other urinary diseases	Female	0-6 days	95+ years	1	Mean BMI
Other urinary diseases	Female	0-6 days	95+ years	0	Socio-demographic Index
Other urinary diseases	Female	0-6 days	95+ years	-1	Healthcare access and quality index
Gynecological diseases	Female	15-19 years	95+ years	-1	Education (years per capita)
Gynecological diseases	Female	15-19 years	95+ years	-1	LDI (I$ per capita)
Gynecological diseases	Female	15-19 years	95+ years	1	Live Births 35+ (proportion)
Gynecological diseases	Female	15-19 years	95+ years	-1	Skilled Birth Attendance (proportion)
Gynecological diseases	Female	15-19 years	95+ years	0	Smoking Prevalence
Gynecological diseases	Female	15-19 years	95+ years	1	Total Fertility Rate
Gynecological diseases	Female	15-19 years	95+ years	-1	Health System Access (capped)
Gynecological diseases	Female	15-19 years	95+ years	-1	Socio-demographic Index
Gynecological diseases	Female	15-19 years	95+ years	-1	Healthcare access and quality index
Uterine fibroids	Female	15-19 years	95+ years	-1	Education (years per capita)
Uterine fibroids	Female	15-19 years	95+ years	-1	LDI (I$ per capita)
Uterine fibroids	Female	15-19 years	95+ years	1	Live Births 35+ (proportion)
Uterine fibroids	Female	15-19 years	95+ years	-1	Skilled Birth Attendance (proportion)
Uterine fibroids	Female	15-19 years	95+ years	0	Smoking Prevalence
Uterine fibroids	Female	15-19 years	95+ years	1	Total Fertility Rate
Uterine fibroids	Female	15-19 years	95+ years	-1	Health System Access (capped)
Uterine fibroids	Female	15-19 years	95+ years	-1	Socio-demographic Index
Uterine fibroids	Female	15-19 years	95+ years	-1	Healthcare access and quality index
Polycystic ovarian syndrome	Female	15-19 years	50-54 years	-1	Education (years per capita)
Polycystic ovarian syndrome	Female	15-19 years	50-54 years	-1	LDI (I$ per capita)
Polycystic ovarian syndrome	Female	15-19 years	50-54 years	1	Live Births 35+ (proportion)
Polycystic ovarian syndrome	Female	15-19 years	50-54 years	-1	Skilled Birth Attendance (proportion)
Polycystic ovarian syndrome	Female	15-19 years	50-54 years	0	Smoking Prevalence
Polycystic ovarian syndrome	Female	15-19 years	50-54 years	1	Total Fertility Rate
Polycystic ovarian syndrome	Female	15-19 years	50-54 years	-1	Health System Access (capped)
Polycystic ovarian syndrome	Female	15-19 years	50-54 years	-1	Socio-demographic Index
Polycystic ovarian syndrome	Female	15-19 years	50-54 years	-1	Healthcare access and quality index
Endometriosis	Female	15-19 years	50-54 years	-1	Education (years per capita)

Cause	Sex	Age start	Age end	Direction	Covariate
Endometriosis	Female	15-19 years	50-54 years	-1	LDI (I$ per capita)
Endometriosis	Female	15-19 years	50-54 years	1	Live Births 35+ (proportion)
Endometriosis	Female	15-19 years	50-54 years	-1	Skilled Birth Attendance (proportion)
Endometriosis	Female	15-19 years	50-54 years	0	Smoking Prevalence
Endometriosis	Female	15-19 years	50-54 years	1	Total Fertility Rate
Endometriosis	Female	15-19 years	50-54 years	-1	Health System Access (capped)
Endometriosis	Female	15-19 years	50-54 years	-1	Socio-demographic Index
Endometriosis	Female	15-19 years	50-54 years	-1	Healthcare access and quality index
Genital prolapse	Female	15-19 years	95+ years	-1	Education (years per capita)
Genital prolapse	Female	15-19 years	95+ years	-1	LDI (I$ per capita)
Genital prolapse	Female	15-19 years	95+ years	1	Live Births 35+ (proportion)
Genital prolapse	Female	15-19 years	95+ years	-1	Skilled Birth Attendance (proportion)
Genital prolapse	Female	15-19 years	95+ years	0	Smoking Prevalence
Genital prolapse	Female	15-19 years	95+ years	1	Total Fertility Rate
Genital prolapse	Female	15-19 years	95+ years	-1	Health System Access (capped)
Genital prolapse	Female	15-19 years	95+ years	-1	Socio-demographic Index
Genital prolapse	Female	15-19 years	95+ years	-1	Healthcare access and quality index
Other gynecological diseases	Female	15-19 years	95+ years	-1	Education (years per capita)
Other gynecological diseases	Female	15-19 years	95+ years	-1	LDI (I$ per capita)
Other gynecological diseases	Female	15-19 years	95+ years	1	Live Births 35+ (proportion)
Other gynecological diseases	Female	15-19 years	95+ years	-1	Skilled Birth Attendance (proportion)
Other gynecological diseases	Female	15-19 years	95+ years	0	Smoking Prevalence
Other gynecological diseases	Female	15-19 years	95+ years	1	Total Fertility Rate
Other gynecological diseases	Female	15-19 years	95+ years	-1	Health System Access (capped)
Other gynecological diseases	Female	15-19 years	95+ years	-1	Socio-demographic Index
Other gynecological diseases	Female	15-19 years	95+ years	-1	Healthcare access and quality index
Hemoglobinopathies and hemolytic anemias	Male	0-6 days	5-9 years	-1	Education (years per capita)
Hemoglobinopathies and hemolytic anemias	Male	0-6 days	5-9 years	-1	LDI (I$ per capita)
Hemoglobinopathies and hemolytic anemias	Male	0-6 days	5-9 years	1	Latitude Under 15 (proportion)
Hemoglobinopathies and hemolytic anemias	Male	0-6 days	5-9 years	1	Latitude 15 to 30 (proportion)
Hemoglobinopathies and hemolytic anemias	Male	0-6 days	5-9 years	0	Latitude 30 to 45 (proportion)
Hemoglobinopathies and hemolytic anemias	Male	0-6 days	5-9 years	-1	Latitude Over 45 (proportion)
Hemoglobinopathies and hemolytic anemias	Male	0-6 days	5-9 years	1	Malaria Lysenko PFPR 1 (Holoendemic)
Hemoglobinopathies and hemolytic anemias	Male	0-6 days	5-9 years	1	Hemoglobinopathies Prevalence x Excess Mortality
Hemoglobinopathies and hemolytic anemias	Male	0-6 days	5-9 years	-1	Health System Access (capped)
Hemoglobinopathies and hemolytic anemias	Male	0-6 days	5-9 years	1	Hemoglobinopathies Prevalence x Excess Mortality (excluding G6PD deficiency)
Hemoglobinopathies and hemolytic anemias	Male	0-6 days	5-9 years	-1	Socio-demographic Index
Hemoglobinopathies and hemolytic anemias	Male	0-6 days	5-9 years	-1	Healthcare access and quality index
Hemoglobinopathies and hemolytic anemias	Female	0-6 days	5-9 years	-1	Education (years per capita)
Hemoglobinopathies and hemolytic anemias	Female	0-6 days	5-9 years	-1	LDI (I$ per capita)
Hemoglobinopathies and hemolytic anemias	Female	0-6 days	5-9 years	1	Latitude Under 15 (proportion)
Hemoglobinopathies and hemolytic anemias	Female	0-6 days	5-9 years	1	Latitude 15 to 30 (proportion)
Hemoglobinopathies and hemolytic anemias	Female	0-6 days	5-9 years	0	Latitude 30 to 45 (proportion)
Hemoglobinopathies and hemolytic anemias	Female	0-6 days	5-9 years	-1	Latitude Over 45 (proportion)
Hemoglobinopathies and hemolytic anemias	Female	0-6 days	5-9 years	1	Malaria Lysenko PFPR 1 (Holoendemic)
Hemoglobinopathies and hemolytic anemias	Female	0-6 days	5-9 years	1	Hemoglobinopathies Prevalence x Excess Mortality
Hemoglobinopathies and hemolytic anemias	Female	0-6 days	5-9 years	-1	Health System Access (capped)
Hemoglobinopathies and hemolytic anemias	Female	0-6 days	5-9 years	1	Hemoglobinopathies Prevalence x Excess Mortality (excluding G6PD deficiency)
Hemoglobinopathies and hemolytic anemias	Female	0-6 days	5-9 years	-1	Socio-demographic Index
Hemoglobinopathies and hemolytic anemias	Female	0-6 days	5-9 years	-1	Healthcare access and quality index
Hemoglobinopathies and hemolytic anemias	Male	10-14 years	95+ years	-1	Education (years per capita)
Hemoglobinopathies and hemolytic anemias	Male	10-14 years	95+ years	-1	LDI (I$ per capita)
Hemoglobinopathies and hemolytic anemias	Male	10-14 years	95+ years	1	Latitude Under 15 (proportion)
Hemoglobinopathies and hemolytic anemias	Male	10-14 years	95+ years	1	Latitude 15 to 30 (proportion)
Hemoglobinopathies and hemolytic anemias	Male	10-14 years	95+ years	0	Latitude 30 to 45 (proportion)
Hemoglobinopathies and hemolytic anemias	Male	10-14 years	95+ years	-1	Latitude Over 45 (proportion)
Hemoglobinopathies and hemolytic anemias	Male	10-14 years	95+ years	1	Malaria Lysenko PFPR 1 (Holoendemic)
Hemoglobinopathies and hemolytic anemias	Male	10-14 years	95+ years	1	Hemoglobinopathies Prevalence x Excess Mortality
Hemoglobinopathies and hemolytic anemias	Male	10-14 years	95+ years	-1	Health System Access (capped)
Hemoglobinopathies and hemolytic anemias	Male	10-14 years	95+ years	1	Hemoglobinopathies Prevalence x Excess Mortality (excluding G6PD deficiency)
Hemoglobinopathies and hemolytic anemias	Male	10-14 years	95+ years	-1	Socio-demographic Index
Hemoglobinopathies and hemolytic anemias	Male	10-14 years	95+ years	-1	Healthcare access and quality index
Hemoglobinopathies and hemolytic anemias	Female	10-14 years	95+ years	-1	Education (years per capita)
Hemoglobinopathies and hemolytic anemias	Female	10-14 years	95+ years	-1	LDI (I$ per capita)
Hemoglobinopathies and hemolytic anemias	Female	10-14 years	95+ years	1	Latitude Under 15 (proportion)
Hemoglobinopathies and hemolytic anemias	Female	10-14 years	95+ years	1	Latitude 15 to 30 (proportion)
Hemoglobinopathies and hemolytic anemias	Female	10-14 years	95+ years	0	Latitude 30 to 45 (proportion)
Hemoglobinopathies and hemolytic anemias	Female	10-14 years	95+ years	-1	Latitude Over 45 (proportion)
Hemoglobinopathies and hemolytic anemias	Female	10-14 years	95+ years	1	Malaria Lysenko PFPR 1 (Holoendemic)
Hemoglobinopathies and hemolytic anemias	Female	10-14 years	95+ years	1	Hemoglobinopathies Prevalence x Excess Mortality
Hemoglobinopathies and hemolytic anemias	Female	10-14 years	95+ years	-1	Health System Access (capped)
Hemoglobinopathies and hemolytic anemias	Female	10-14 years	95+ years	1	Hemoglobinopathies Prevalence x Excess Mortality (excluding G6PD deficiency)
Hemoglobinopathies and hemolytic anemias	Female	10-14 years	95+ years	-1	Socio-demographic Index
Hemoglobinopathies and hemolytic anemias	Female	10-14 years	95+ years	-1	Healthcare access and quality index
Endocrine, metabolic, blood, and immune disorders	Female	0-6 days	95+ years	1	Alcohol (liters per capita)
Endocrine, metabolic, blood, and immune disorders	Female	0-6 days	95+ years	1	Animal Fats (kcal per capita)
Endocrine, metabolic, blood, and immune disorders	Female	0-6 days	95+ years	-1	Education (years per capita)
Endocrine, metabolic, blood, and immune disorders	Female	0-6 days	95+ years	-1	LDI (I$ per capita)
Endocrine, metabolic, blood, and immune disorders	Female	0-6 days	95+ years	1	Mean BMI
Endocrine, metabolic, blood, and immune disorders	Female	0-6 days	95+ years	1	Cholesterol (total, mean per capita)
Endocrine, metabolic, blood, and immune disorders	Female	0-6 days	95+ years	1	Total Calories (kcal per capita)
Endocrine, metabolic, blood, and immune disorders	Female	0-6 days	95+ years	0	Socio-demographic Index
Endocrine, metabolic, blood, and immune disorders	Female	0-6 days	95+ years	-1	Healthcare access and quality index
Endocrine, metabolic, blood, and immune disorders	Male	0-6 days	95+ years	1	Alcohol (liters per capita)
Endocrine, metabolic, blood, and immune disorders	Male	0-6 days	95+ years	1	Animal Fats (kcal per capita)
Endocrine, metabolic, blood, and immune disorders	Male	0-6 days	95+ years	-1	Education (years per capita)
Endocrine, metabolic, blood, and immune disorders	Male	0-6 days	95+ years	-1	LDI (I$ per capita)
Endocrine, metabolic, blood, and immune disorders	Male	0-6 days	95+ years	1	Mean BMI
Endocrine, metabolic, blood, and immune disorders	Male	0-6 days	95+ years	1	Cholesterol (total, mean per capita)
Endocrine, metabolic, blood, and immune disorders	Male	0-6 days	95+ years	1	Total Calories (kcal per capita)
Endocrine, metabolic, blood, and immune disorders	Male	0-6 days	95+ years	0	Socio-demographic Index
Endocrine, metabolic, blood, and immune disorders	Male	0-6 days	95+ years	-1	Healthcare access and quality index
Musculoskeletal disorders	Male	5-9 years	95+ years	1	Alcohol (liters per capita)
Musculoskeletal disorders	Male	5-9 years	95+ years	1	Cumulative Cigarettes (10 Years)
Musculoskeletal disorders	Male	5-9 years	95+ years	1	Cumulative Cigarettes (5 Years)

Cause	Sex	Age start	Age end	Direction	Covariate
Musculoskeletal disorders	Male	5-9 years	95+ years	0	Education (years per capita)
Musculoskeletal disorders	Male	5-9 years	95+ years	0	LDI (I$ per capita)
Musculoskeletal disorders	Male	5-9 years	95+ years	1	Mean BMI
Musculoskeletal disorders	Male	5-9 years	95+ years	1	Cholesterol (total, mean per capita)
Musculoskeletal disorders	Male	5-9 years	95+ years	1	Smoking Prevalence
Musculoskeletal disorders	Male	5-9 years	95+ years	0	Socio-demographic Index
Musculoskeletal disorders	Male	5-9 years	95+ years	0	vegetables adjusted(g)
Musculoskeletal disorders	Male	5-9 years	95+ years	-1	Healthcare access and quality index
Musculoskeletal disorders	Female	5-9 years	95+ years	1	Alcohol (liters per capita)
Musculoskeletal disorders	Female	5-9 years	95+ years	1	Cumulative Cigarettes (10 Years)
Musculoskeletal disorders	Female	5-9 years	95+ years	1	Cumulative Cigarettes (5 Years)
Musculoskeletal disorders	Female	5-9 years	95+ years	0	Education (years per capita)
Musculoskeletal disorders	Female	5-9 years	95+ years	0	LDI (I$ per capita)
Musculoskeletal disorders	Female	5-9 years	95+ years	1	Mean BMI
Musculoskeletal disorders	Female	5-9 years	95+ years	1	Cholesterol (total, mean per capita)
Musculoskeletal disorders	Female	5-9 years	95+ years	1	Smoking Prevalence
Musculoskeletal disorders	Female	5-9 years	95+ years	0	Socio-demographic Index
Musculoskeletal disorders	Female	5-9 years	95+ years	0	vegetables adjusted(g)
Musculoskeletal disorders	Female	5-9 years	95+ years	-1	Healthcare access and quality index
Rheumatoid arthritis	Female	5-9 years	95+ years	1	Alcohol (liters per capita)
Rheumatoid arthritis	Female	5-9 years	95+ years	1	Cumulative Cigarettes (10 Years)
Rheumatoid arthritis	Female	5-9 years	95+ years	1	Cumulative Cigarettes (5 Years)
Rheumatoid arthritis	Female	5-9 years	95+ years	-1	Education (years per capita)
Rheumatoid arthritis	Female	5-9 years	95+ years	-1	LDI (I$ per capita)
Rheumatoid arthritis	Female	5-9 years	95+ years	1	Mean BMI
Rheumatoid arthritis	Female	5-9 years	95+ years	1	Cholesterol (total, mean per capita)
Rheumatoid arthritis	Female	5-9 years	95+ years	1	Smoking Prevalence
Rheumatoid arthritis	Female	5-9 years	95+ years	0	Socio-demographic Index
Rheumatoid arthritis	Female	5-9 years	95+ years	0	vegetables adjusted(g)
Rheumatoid arthritis	Female	5-9 years	95+ years	-1	Healthcare access and quality index
Rheumatoid arthritis	Male	5-9 years	95+ years	1	Alcohol (liters per capita)
Rheumatoid arthritis	Male	5-9 years	95+ years	1	Cumulative Cigarettes (10 Years)
Rheumatoid arthritis	Male	5-9 years	95+ years	1	Cumulative Cigarettes (5 Years)
Rheumatoid arthritis	Male	5-9 years	95+ years	-1	Education (years per capita)
Rheumatoid arthritis	Male	5-9 years	95+ years	-1	LDI (I$ per capita)
Rheumatoid arthritis	Male	5-9 years	95+ years	1	Mean BMI
Rheumatoid arthritis	Male	5-9 years	95+ years	1	Cholesterol (total, mean per capita)
Rheumatoid arthritis	Male	5-9 years	95+ years	1	Smoking Prevalence
Rheumatoid arthritis	Male	5-9 years	95+ years	0	Socio-demographic Index
Rheumatoid arthritis	Male	5-9 years	95+ years	0	vegetables adjusted(g)
Rheumatoid arthritis	Male	5-9 years	95+ years	-1	Healthcare access and quality index
Other musculoskeletal disorders	Male	5-9 years	95+ years	1	Alcohol (liters per capita)
Other musculoskeletal disorders	Male	5-9 years	95+ years	1	Cumulative Cigarettes (10 Years)
Other musculoskeletal disorders	Male	5-9 years	95+ years	1	Cumulative Cigarettes (5 Years)
Other musculoskeletal disorders	Male	5-9 years	95+ years	0	Education (years per capita)
Other musculoskeletal disorders	Male	5-9 years	95+ years	0	LDI (I$ per capita)
Other musculoskeletal disorders	Male	5-9 years	95+ years	1	Mean BMI
Other musculoskeletal disorders	Male	5-9 years	95+ years	1	Cholesterol (total, mean per capita)
Other musculoskeletal disorders	Male	5-9 years	95+ years	1	Smoking Prevalence
Other musculoskeletal disorders	Male	5-9 years	95+ years	0	Socio-demographic Index
Other musculoskeletal disorders	Male	5-9 years	95+ years	0	vegetables adjusted(g)
Other musculoskeletal disorders	Male	5-9 years	95+ years	-1	Healthcare access and quality index
Other musculoskeletal disorders	Female	5-9 years	95+ years	1	Alcohol (liters per capita)
Other musculoskeletal disorders	Female	5-9 years	95+ years	1	Cumulative Cigarettes (10 Years)
Other musculoskeletal disorders	Female	5-9 years	95+ years	1	Cumulative Cigarettes (5 Years)
Other musculoskeletal disorders	Female	5-9 years	95+ years	0	Education (years per capita)
Other musculoskeletal disorders	Female	5-9 years	95+ years	0	LDI (I$ per capita)
Other musculoskeletal disorders	Female	5-9 years	95+ years	1	Mean BMI
Other musculoskeletal disorders	Female	5-9 years	95+ years	1	Cholesterol (total, mean per capita)
Other musculoskeletal disorders	Female	5-9 years	95+ years	1	Smoking Prevalence
Other musculoskeletal disorders	Female	5-9 years	95+ years	0	Socio-demographic Index
Other musculoskeletal disorders	Female	5-9 years	95+ years	0	vegetables adjusted(g)
Other musculoskeletal disorders	Female	5-9 years	95+ years	-1	Healthcare access and quality index
Congenital birth defects	Male	0-6 days	65-69 years	-1	Legality of Abortion
Congenital birth defects	Male	0-6 days	65-69 years	1	Alcohol (liters per capita)
Congenital birth defects	Male	0-6 days	65-69 years	0	Antenatal Care (1 visit) Coverage (proportion)
Congenital birth defects	Male	0-6 days	65-69 years	-1	Antenatal Care (4 visits) Coverage (proportion)
Congenital birth defects	Male	0-6 days	65-69 years	-1	Education (years per capita)
Congenital birth defects	Male	0-6 days	65-69 years	-1	In-Facility Delivery (proportion)
Congenital birth defects	Male	0-6 days	65-69 years	1	Live Births 35+ (proportion)
Congenital birth defects	Male	0-6 days	65-69 years	1	Indoor Air Pollution (All Cooking Fuels)
Congenital birth defects	Male	0-6 days	65-69 years	1	Outdoor Air Pollution (PM2.5)
Congenital birth defects	Male	0-6 days	65-69 years	1	Smoking Prevalence (Reproductive Age Standardized)
Congenital birth defects	Male	0-6 days	65-69 years	-1	Socio-demographic Index
Congenital birth defects	Male	0-6 days	65-69 years	1	fruits unadjusted(g)
Congenital birth defects	Male	0-6 days	65-69 years	1	vegetables unadjusted(g)
Congenital birth defects	Male	0-6 days	65-69 years	1	Maternal alcohol consumption during pregnancy (proportion)
Congenital birth defects	Male	0-6 days	65-69 years	-1	Healthcare access and quality index
Congenital birth defects	Male	0-6 days	65-69 years	-1	Folic acid unadjusted (ug)
Congenital birth defects	Female	0-6 days	65-69 years	-1	Legality of Abortion
Congenital birth defects	Female	0-6 days	65-69 years	1	Alcohol (liters per capita)
Congenital birth defects	Female	0-6 days	65-69 years	0	Antenatal Care (1 visit) Coverage (proportion)
Congenital birth defects	Female	0-6 days	65-69 years	-1	Antenatal Care (4 visits) Coverage (proportion)
Congenital birth defects	Female	0-6 days	65-69 years	-1	Education (years per capita)
Congenital birth defects	Female	0-6 days	65-69 years	-1	In-Facility Delivery (proportion)
Congenital birth defects	Female	0-6 days	65-69 years	1	Live Births 35+ (proportion)
Congenital birth defects	Female	0-6 days	65-69 years	1	Indoor Air Pollution (All Cooking Fuels)
Congenital birth defects	Female	0-6 days	65-69 years	1	Outdoor Air Pollution (PM2.5)
Congenital birth defects	Female	0-6 days	65-69 years	1	Smoking Prevalence (Reproductive Age Standardized)
Congenital birth defects	Female	0-6 days	65-69 years	-1	Socio-demographic Index
Congenital birth defects	Female	0-6 days	65-69 years	1	fruits unadjusted(g)
Congenital birth defects	Female	0-6 days	65-69 years	1	vegetables unadjusted(g)
Congenital birth defects	Female	0-6 days	65-69 years	1	Maternal alcohol consumption during pregnancy (proportion)
Congenital birth defects	Female	0-6 days	65-69 years	-1	Healthcare access and quality index
Congenital birth defects	Female	0-6 days	65-69 years	-1	Folic acid unadjusted (ug)

Cause	Sex	Age start	Age end	Direction	Covariate
Neural tube defects	Male	0-6 days	65-69 years	-1	Legality of Abortion
Neural tube defects	Male	0-6 days	65-69 years	-1	Antenatal Care (1 visit) Coverage (proportion)
Neural tube defects	Male	0-6 days	65-69 years	-1	Antenatal Care (4 visits) Coverage (proportion)
Neural tube defects	Male	0-6 days	65-69 years	1	Diabetes Age-Standardized Prevalence (proportion)
Neural tube defects	Male	0-6 days	65-69 years	-1	Education (years per capita)
Neural tube defects	Male	0-6 days	65-69 years	-1	In-Facility Delivery (proportion)
Neural tube defects	Male	0-6 days	65-69 years	1	Indoor Air Pollution (All Cooking Fuels)
Neural tube defects	Male	0-6 days	65-69 years	1	Outdoor Air Pollution (PM2.5)
Neural tube defects	Male	0-6 days	65-69 years	1	Smoking Prevalence (Reproductive Age Standardized)
Neural tube defects	Male	0-6 days	65-69 years	-1	Socio-demographic Index
Neural tube defects	Male	0-6 days	65-69 years	0	fruits unadjusted(g)
Neural tube defects	Male	0-6 days	65-69 years	-1	vegetables unadjusted(g)
Neural tube defects	Male	0-6 days	65-69 years	1	Maternal alcohol consumption during pregnancy (proportion)
Neural tube defects	Male	0-6 days	65-69 years	-1	Healthcare access and quality index
Neural tube defects	Male	0-6 days	65-69 years	-1	Folic acid unadjusted (ug)
Neural tube defects	Female	0-6 days	65-69 years	-1	Legality of Abortion
Neural tube defects	Female	0-6 days	65-69 years	-1	Antenatal Care (1 visit) Coverage (proportion)
Neural tube defects	Female	0-6 days	65-69 years	-1	Antenatal Care (4 visits) Coverage (proportion)
Neural tube defects	Female	0-6 days	65-69 years	1	Diabetes Age-Standardized Prevalence (proportion)
Neural tube defects	Female	0-6 days	65-69 years	-1	Education (years per capita)
Neural tube defects	Female	0-6 days	65-69 years	-1	In-Facility Delivery (proportion)
Neural tube defects	Female	0-6 days	65-69 years	1	Indoor Air Pollution (All Cooking Fuels)
Neural tube defects	Female	0-6 days	65-69 years	1	Outdoor Air Pollution (PM2.5)
Neural tube defects	Female	0-6 days	65-69 years	1	Smoking Prevalence (Reproductive Age Standardized)
Neural tube defects	Female	0-6 days	65-69 years	-1	Socio-demographic Index
Neural tube defects	Female	0-6 days	65-69 years	0	fruits unadjusted(g)
Neural tube defects	Female	0-6 days	65-69 years	-1	vegetables unadjusted(g)
Neural tube defects	Female	0-6 days	65-69 years	1	Maternal alcohol consumption during pregnancy (proportion)
Neural tube defects	Female	0-6 days	65-69 years	-1	Healthcare access and quality index
Neural tube defects	Female	0-6 days	65-69 years	-1	Folic acid unadjusted (ug)
Congenital heart anomalies	Male	0-6 days	65-69 years	-1	Legality of Abortion
Congenital heart anomalies	Male	0-6 days	65-69 years	1	Alcohol (liters per capita)
Congenital heart anomalies	Male	0-6 days	65-69 years	-1	Antenatal Care (1 visit) Coverage (proportion)
Congenital heart anomalies	Male	0-6 days	65-69 years	-1	Antenatal Care (4 visits) Coverage (proportion)
Congenital heart anomalies	Male	0-6 days	65-69 years	1	Diabetes Age-Standardized Prevalence (proportion)
Congenital heart anomalies	Male	0-6 days	65-69 years	-1	Education (years per capita)
Congenital heart anomalies	Male	0-6 days	65-69 years	-1	In-Facility Delivery (proportion)
Congenital heart anomalies	Male	0-6 days	65-69 years	1	Live Births 35+ (proportion)
Congenital heart anomalies	Male	0-6 days	65-69 years	-1	Skilled Birth Attendance (proportion)
Congenital heart anomalies	Male	0-6 days	65-69 years	1	Smoking Prevalence (Reproductive Age Standardized)
Congenital heart anomalies	Male	0-6 days	65-69 years	-1	Socio-demographic Index
Congenital heart anomalies	Male	0-6 days	65-69 years	1	Maternal alcohol consumption during pregnancy (proportion)
Congenital heart anomalies	Male	0-6 days	65-69 years	-1	Healthcare access and quality index
Congenital heart anomalies	Female	0-6 days	65-69 years	-1	Legality of Abortion
Congenital heart anomalies	Female	0-6 days	65-69 years	1	Alcohol (liters per capita)
Congenital heart anomalies	Female	0-6 days	65-69 years	-1	Antenatal Care (1 visit) Coverage (proportion)
Congenital heart anomalies	Female	0-6 days	65-69 years	-1	Antenatal Care (4 visits) Coverage (proportion)
Congenital heart anomalies	Female	0-6 days	65-69 years	1	Diabetes Age-Standardized Prevalence (proportion)
Congenital heart anomalies	Female	0-6 days	65-69 years	-1	Education (years per capita)
Congenital heart anomalies	Female	0-6 days	65-69 years	-1	In-Facility Delivery (proportion)
Congenital heart anomalies	Female	0-6 days	65-69 years	1	Live Births 35+ (proportion)
Congenital heart anomalies	Female	0-6 days	65-69 years	-1	Skilled Birth Attendance (proportion)
Congenital heart anomalies	Female	0-6 days	65-69 years	1	Smoking Prevalence (Reproductive Age Standardized)
Congenital heart anomalies	Female	0-6 days	65-69 years	-1	Socio-demographic Index
Congenital heart anomalies	Female	0-6 days	65-69 years	1	Maternal alcohol consumption during pregnancy (proportion)
Congenital heart anomalies	Female	0-6 days	65-69 years	-1	Healthcare access and quality index
Orofacial clefts	Male	0-6 days	1-4 years	-1	Legality of Abortion
Orofacial clefts	Male	0-6 days	1-4 years	1	Alcohol (liters per capita)
Orofacial clefts	Male	0-6 days	1-4 years	-1	Antenatal Care (1 visit) Coverage (proportion)
Orofacial clefts	Male	0-6 days	1-4 years	-1	Antenatal Care (4 visits) Coverage (proportion)
Orofacial clefts	Male	0-6 days	1-4 years	1	Diabetes Age-Standardized Prevalence (proportion)
Orofacial clefts	Male	0-6 days	1-4 years	-1	Education (years per capita)
Orofacial clefts	Male	0-6 days	1-4 years	1	Indoor Air Pollution (All Cooking Fuels)
Orofacial clefts	Male	0-6 days	1-4 years	1	Outdoor Air Pollution (PM2.5)
Orofacial clefts	Male	0-6 days	1-4 years	-1	Skilled Birth Attendance (proportion)
Orofacial clefts	Male	0-6 days	1-4 years	1	Smoking Prevalence (Reproductive Age Standardized)
Orofacial clefts	Male	0-6 days	1-4 years	-1	Socio-demographic Index
Orofacial clefts	Male	0-6 days	1-4 years	0	fruits unadjusted(g)
Orofacial clefts	Male	0-6 days	1-4 years	0	vegetables unadjusted(g)
Orofacial clefts	Male	0-6 days	1-4 years	1	Maternal alcohol consumption during pregnancy (proportion)
Orofacial clefts	Male	0-6 days	1-4 years	-1	Healthcare access and quality index
Orofacial clefts	Male	0-6 days	1-4 years	-1	Folic acid unadjusted (ug)
Orofacial clefts	Female	0-6 days	1-4 years	-1	Legality of Abortion
Orofacial clefts	Female	0-6 days	1-4 years	1	Alcohol (liters per capita)
Orofacial clefts	Female	0-6 days	1-4 years	-1	Antenatal Care (1 visit) Coverage (proportion)
Orofacial clefts	Female	0-6 days	1-4 years	-1	Antenatal Care (4 visits) Coverage (proportion)
Orofacial clefts	Female	0-6 days	1-4 years	1	Diabetes Age-Standardized Prevalence (proportion)
Orofacial clefts	Female	0-6 days	1-4 years	-1	Education (years per capita)
Orofacial clefts	Female	0-6 days	1-4 years	1	Indoor Air Pollution (All Cooking Fuels)
Orofacial clefts	Female	0-6 days	1-4 years	1	Outdoor Air Pollution (PM2.5)
Orofacial clefts	Female	0-6 days	1-4 years	-1	Skilled Birth Attendance (proportion)
Orofacial clefts	Female	0-6 days	1-4 years	1	Smoking Prevalence (Reproductive Age Standardized)
Orofacial clefts	Female	0-6 days	1-4 years	-1	Socio-demographic Index
Orofacial clefts	Female	0-6 days	1-4 years	0	fruits unadjusted(g)
Orofacial clefts	Female	0-6 days	1-4 years	0	vegetables unadjusted(g)
Orofacial clefts	Female	0-6 days	1-4 years	1	Maternal alcohol consumption during pregnancy (proportion)
Orofacial clefts	Female	0-6 days	1-4 years	-1	Healthcare access and quality index
Down syndrome	Male	0-6 days	65-69 years	-1	Legality of Abortion
Down syndrome	Male	0-6 days	65-69 years	-1	Antenatal Care (1 visit) Coverage (proportion)
Down syndrome	Male	0-6 days	65-69 years	-1	Antenatal Care (4 visits) Coverage (proportion)
Down syndrome	Male	0-6 days	65-69 years	-1	Education (years per capita)
Down syndrome	Male	0-6 days	65-69 years	-1	In-Facility Delivery (proportion)
Down syndrome	Male	0-6 days	65-69 years	-1	LDI (I$ per capita)
Down syndrome	Male	0-6 days	65-69 years	1	Live Births 35+ (proportion)
Down syndrome	Male	0-6 days	65-69 years	1	Live Births 40+ (proportion)

Cause	Sex	Age start	Age end	Direction	Covariate
Down syndrome	Male	0-6 days	65-69 years	1	Indoor Air Pollution (All Cooking Fuels)
Down syndrome	Male	0-6 days	65-69 years	1	Smoking Prevalence (Reproductive Age Standardized)
Down syndrome	Male	0-6 days	65-69 years	-1	Socio-demographic Index
Down syndrome	Male	0-6 days	65-69 years	-1	vegetables unadjusted(g)
Down syndrome	Male	0-6 days	65-69 years	1	Maternal alcohol consumption during pregnancy (proportion)
Down syndrome	Male	0-6 days	65-69 years	-1	Healthcare access and quality index
Down syndrome	Female	0-6 days	65-69 years	-1	Legality of Abortion
Down syndrome	Female	0-6 days	65-69 years	-1	Antenatal Care (1 visit) Coverage (proportion)
Down syndrome	Female	0-6 days	65-69 years	-1	Antenatal Care (4 visits) Coverage (proportion)
Down syndrome	Female	0-6 days	65-69 years	-1	Education (years per capita)
Down syndrome	Female	0-6 days	65-69 years	-1	In-Facility Delivery (proportion)
Down syndrome	Female	0-6 days	65-69 years	-1	LDI (I$ per capita)
Down syndrome	Female	0-6 days	65-69 years	1	Live Births 35+ (proportion)
Down syndrome	Female	0-6 days	65-69 years	1	Live Births 40+ (proportion)
Down syndrome	Female	0-6 days	65-69 years	1	Indoor Air Pollution (All Cooking Fuels)
Down syndrome	Female	0-6 days	65-69 years	1	Smoking Prevalence (Reproductive Age Standardized)
Down syndrome	Female	0-6 days	65-69 years	-1	Socio-demographic Index
Down syndrome	Female	0-6 days	65-69 years	-1	vegetables unadjusted(g)
Down syndrome	Female	0-6 days	65-69 years	1	Maternal alcohol consumption during pregnancy (proportion)
Down syndrome	Female	0-6 days	65-69 years	-1	Healthcare access and quality index
Other chromosomal abnormalities	Male	0-6 days	65-69 years	-1	Legality of Abortion
Other chromosomal abnormalities	Male	0-6 days	65-69 years	1	Alcohol (liters per capita)
Other chromosomal abnormalities	Male	0-6 days	65-69 years	-1	Antenatal Care (1 visit) Coverage (proportion)
Other chromosomal abnormalities	Male	0-6 days	65-69 years	-1	Antenatal Care (4 visits) Coverage (proportion)
Other chromosomal abnormalities	Male	0-6 days	65-69 years	-1	Education (years per capita)
Other chromosomal abnormalities	Male	0-6 days	65-69 years	-1	In-Facility Delivery (proportion)
Other chromosomal abnormalities	Male	0-6 days	65-69 years	-1	LDI (I$ per capita)
Other chromosomal abnormalities	Male	0-6 days	65-69 years	1	Live Births 35+ (proportion)
Other chromosomal abnormalities	Male	0-6 days	65-69 years	1	Live Births 40+ (proportion)
Other chromosomal abnormalities	Male	0-6 days	65-69 years	1	Indoor Air Pollution (All Cooking Fuels)
Other chromosomal abnormalities	Male	0-6 days	65-69 years	-1	Skilled Birth Attendance (proportion)
Other chromosomal abnormalities	Male	0-6 days	65-69 years	1	Smoking Prevalence (Reproductive Age Standardized)
Other chromosomal abnormalities	Male	0-6 days	65-69 years	0	Socio-demographic Index
Other chromosomal abnormalities	Male	0-6 days	65-69 years	1	Maternal alcohol consumption during pregnancy (proportion)
Other chromosomal abnormalities	Male	0-6 days	65-69 years	-1	Healthcare access and quality index
Other chromosomal abnormalities	Female	0-6 days	65-69 years	-1	Legality of Abortion
Other chromosomal abnormalities	Female	0-6 days	65-69 years	1	Alcohol (liters per capita)
Other chromosomal abnormalities	Female	0-6 days	65-69 years	-1	Antenatal Care (1 visit) Coverage (proportion)
Other chromosomal abnormalities	Female	0-6 days	65-69 years	-1	Antenatal Care (4 visits) Coverage (proportion)
Other chromosomal abnormalities	Female	0-6 days	65-69 years	-1	Education (years per capita)
Other chromosomal abnormalities	Female	0-6 days	65-69 years	-1	In-Facility Delivery (proportion)
Other chromosomal abnormalities	Female	0-6 days	65-69 years	-1	LDI (I$ per capita)
Other chromosomal abnormalities	Female	0-6 days	65-69 years	1	Live Births 35+ (proportion)
Other chromosomal abnormalities	Female	0-6 days	65-69 years	1	Live Births 40+ (proportion)
Other chromosomal abnormalities	Female	0-6 days	65-69 years	1	Indoor Air Pollution (All Cooking Fuels)
Other chromosomal abnormalities	Female	0-6 days	65-69 years	-1	Skilled Birth Attendance (proportion)
Other chromosomal abnormalities	Female	0-6 days	65-69 years	1	Smoking Prevalence (Reproductive Age Standardized)
Other chromosomal abnormalities	Female	0-6 days	65-69 years	0	Socio-demographic Index
Other chromosomal abnormalities	Female	0-6 days	65-69 years	1	Maternal alcohol consumption during pregnancy (proportion)
Other chromosomal abnormalities	Female	0-6 days	65-69 years	-1	Healthcare access and quality index
Congenital musculoskeletal and limb anomalies	Male	0-6 days	65-69 years	-1	Legality of Abortion
Congenital musculoskeletal and limb anomalies	Male	0-6 days	65-69 years	1	Alcohol (liters per capita)
Congenital musculoskeletal and limb anomalies	Male	0-6 days	65-69 years	-1	Antenatal Care (1 visit) Coverage (proportion)
Congenital musculoskeletal and limb anomalies	Male	0-6 days	65-69 years	-1	Antenatal Care (4 visits) Coverage (proportion)
Congenital musculoskeletal and limb anomalies	Male	0-6 days	65-69 years	1	Diabetes Age-Standardized Prevalence (proportion)
Congenital musculoskeletal and limb anomalies	Male	0-6 days	65-69 years	-1	Education (years per capita)
Congenital musculoskeletal and limb anomalies	Male	0-6 days	65-69 years	-1	In-Facility Delivery (proportion)
Congenital musculoskeletal and limb anomalies	Male	0-6 days	65-69 years	-1	LDI (I$ per capita)
Congenital musculoskeletal and limb anomalies	Male	0-6 days	65-69 years	1	Indoor Air Pollution (All Cooking Fuels)
Congenital musculoskeletal and limb anomalies	Male	0-6 days	65-69 years	1	Smoking Prevalence (Reproductive Age Standardized)
Congenital musculoskeletal and limb anomalies	Male	0-6 days	65-69 years	-1	Socio-demographic Index
Congenital musculoskeletal and limb anomalies	Male	0-6 days	65-69 years	0	fruits unadjusted(g)
Congenital musculoskeletal and limb anomalies	Male	0-6 days	65-69 years	0	vegetables unadjusted(g)
Congenital musculoskeletal and limb anomalies	Male	0-6 days	65-69 years	1	Maternal alcohol consumption during pregnancy (proportion)
Congenital musculoskeletal and limb anomalies	Male	0-6 days	65-69 years	-1	Healthcare access and quality index
Congenital musculoskeletal and limb anomalies	Female	0-6 days	65-69 years	-1	Legality of Abortion
Congenital musculoskeletal and limb anomalies	Female	0-6 days	65-69 years	1	Alcohol (liters per capita)
Congenital musculoskeletal and limb anomalies	Female	0-6 days	65-69 years	-1	Antenatal Care (1 visit) Coverage (proportion)
Congenital musculoskeletal and limb anomalies	Female	0-6 days	65-69 years	-1	Antenatal Care (4 visits) Coverage (proportion)
Congenital musculoskeletal and limb anomalies	Female	0-6 days	65-69 years	1	Diabetes Age-Standardized Prevalence (proportion)
Congenital musculoskeletal and limb anomalies	Female	0-6 days	65-69 years	-1	Education (years per capita)
Congenital musculoskeletal and limb anomalies	Female	0-6 days	65-69 years	-1	In-Facility Delivery (proportion)
Congenital musculoskeletal and limb anomalies	Female	0-6 days	65-69 years	-1	LDI (I$ per capita)
Congenital musculoskeletal and limb anomalies	Female	0-6 days	65-69 years	1	Indoor Air Pollution (All Cooking Fuels)
Congenital musculoskeletal and limb anomalies	Female	0-6 days	65-69 years	1	Smoking Prevalence (Reproductive Age Standardized)
Congenital musculoskeletal and limb anomalies	Female	0-6 days	65-69 years	-1	Socio-demographic Index
Congenital musculoskeletal and limb anomalies	Female	0-6 days	65-69 years	0	fruits unadjusted(g)
Congenital musculoskeletal and limb anomalies	Female	0-6 days	65-69 years	0	vegetables unadjusted(g)
Congenital musculoskeletal and limb anomalies	Female	0-6 days	65-69 years	1	Maternal alcohol consumption during pregnancy (proportion)
Congenital musculoskeletal and limb anomalies	Female	0-6 days	65-69 years	-1	Healthcare access and quality index
Urogenital congenital anomalies	Male	0-6 days	65-69 years	1	Alcohol (liters per capita)
Urogenital congenital anomalies	Male	0-6 days	65-69 years	-1	Antenatal Care (1 visit) Coverage (proportion)
Urogenital congenital anomalies	Male	0-6 days	65-69 years	-1	Antenatal Care (4 visits) Coverage (proportion)
Urogenital congenital anomalies	Male	0-6 days	65-69 years	1	Diabetes Age-Standardized Prevalence (proportion)
Urogenital congenital anomalies	Male	0-6 days	65-69 years	-1	Education (years per capita)
Urogenital congenital anomalies	Male	0-6 days	65-69 years	-1	In-Facility Delivery (proportion)
Urogenital congenital anomalies	Male	0-6 days	65-69 years	-1	LDI (I$ per capita)
Urogenital congenital anomalies	Male	0-6 days	65-69 years	1	Indoor Air Pollution (All Cooking Fuels)
Urogenital congenital anomalies	Male	0-6 days	65-69 years	1	Outdoor Air Pollution (PM2.5)
Urogenital congenital anomalies	Male	0-6 days	65-69 years	1	Smoking Prevalence (Reproductive Age Standardized)
Urogenital congenital anomalies	Male	0-6 days	65-69 years	-1	Socio-demographic Index
Urogenital congenital anomalies	Male	0-6 days	65-69 years	1	Maternal alcohol consumption during pregnancy (proportion)
Urogenital congenital anomalies	Male	0-6 days	65-69 years	-1	Healthcare access and quality index
Urogenital congenital anomalies	Female	0-6 days	65-69 years	1	Alcohol (liters per capita)
Urogenital congenital anomalies	Female	0-6 days	65-69 years	-1	Antenatal Care (1 visit) Coverage (proportion)

Cause	Sex	Age start	Age end	Direction	Covariate
Urogenital congenital anomalies	Female	0-6 days	65-69 years	-1	Antenatal Care (4 visits) Coverage (proportion)
Urogenital congenital anomalies	Female	0-6 days	65-69 years	1	Diabetes Age-Standardized Prevalence (proportion)
Urogenital congenital anomalies	Female	0-6 days	65-69 years	-1	Education (years per capita)
Urogenital congenital anomalies	Female	0-6 days	65-69 years	-1	In-Facility Delivery (proportion)
Urogenital congenital anomalies	Female	0-6 days	65-69 years	-1	LDI (I$ per capita)
Urogenital congenital anomalies	Female	0-6 days	65-69 years	1	Indoor Air Pollution (All Cooking Fuels)
Urogenital congenital anomalies	Female	0-6 days	65-69 years	1	Outdoor Air Pollution (PM2.5)
Urogenital congenital anomalies	Female	0-6 days	65-69 years	1	Smoking Prevalence (Reproductive Age Standardized)
Urogenital congenital anomalies	Female	0-6 days	65-69 years	-1	Socio-demographic Index
Urogenital congenital anomalies	Female	0-6 days	65-69 years	1	Maternal alcohol consumption during pregnancy (proportion)
Urogenital congenital anomalies	Female	0-6 days	65-69 years	-1	Healthcare access and quality index
Digestive congenital anomalies	Female	0-6 days	65-69 years	1	Alcohol (liters per capita)
Digestive congenital anomalies	Female	0-6 days	65-69 years	-1	Antenatal Care (1 visit) Coverage (proportion)
Digestive congenital anomalies	Female	0-6 days	65-69 years	-1	Antenatal Care (4 visits) Coverage (proportion)
Digestive congenital anomalies	Female	0-6 days	65-69 years	1	Diabetes Age-Standardized Prevalence (proportion)
Digestive congenital anomalies	Female	0-6 days	65-69 years	-1	Education (years per capita)
Digestive congenital anomalies	Female	0-6 days	65-69 years	-1	In-Facility Delivery (proportion)
Digestive congenital anomalies	Female	0-6 days	65-69 years	-1	LDI (I$ per capita)
Digestive congenital anomalies	Female	0-6 days	65-69 years	1	Indoor Air Pollution (All Cooking Fuels)
Digestive congenital anomalies	Female	0-6 days	65-69 years	1	Smoking Prevalence (Reproductive Age Standardized)
Digestive congenital anomalies	Female	0-6 days	65-69 years	-1	Health System Access (capped)
Digestive congenital anomalies	Female	0-6 days	65-69 years	1	Prevalence of obesity (age-standardized)
Digestive congenital anomalies	Female	0-6 days	65-69 years	-1	Socio-demographic Index
Digestive congenital anomalies	Female	0-6 days	65-69 years	0	fruits unadjusted(g)
Digestive congenital anomalies	Female	0-6 days	65-69 years	0	vegetables unadjusted(g)
Digestive congenital anomalies	Female	0-6 days	65-69 years	1	Maternal alcohol consumption during pregnancy (proportion)
Digestive congenital anomalies	Female	0-6 days	65-69 years	-1	Healthcare access and quality index
Digestive congenital anomalies	Male	0-6 days	65-69 years	1	Alcohol (liters per capita)
Digestive congenital anomalies	Male	0-6 days	65-69 years	-1	Antenatal Care (1 visit) Coverage (proportion)
Digestive congenital anomalies	Male	0-6 days	65-69 years	-1	Antenatal Care (4 visits) Coverage (proportion)
Digestive congenital anomalies	Male	0-6 days	65-69 years	1	Diabetes Age-Standardized Prevalence (proportion)
Digestive congenital anomalies	Male	0-6 days	65-69 years	-1	Education (years per capita)
Digestive congenital anomalies	Male	0-6 days	65-69 years	-1	In-Facility Delivery (proportion)
Digestive congenital anomalies	Male	0-6 days	65-69 years	-1	LDI (I$ per capita)
Digestive congenital anomalies	Male	0-6 days	65-69 years	1	Indoor Air Pollution (All Cooking Fuels)
Digestive congenital anomalies	Male	0-6 days	65-69 years	1	Smoking Prevalence (Reproductive Age Standardized)
Digestive congenital anomalies	Male	0-6 days	65-69 years	-1	Health System Access (capped)
Digestive congenital anomalies	Male	0-6 days	65-69 years	1	Prevalence of obesity (age-standardized)
Digestive congenital anomalies	Male	0-6 days	65-69 years	-1	Socio-demographic Index
Digestive congenital anomalies	Male	0-6 days	65-69 years	0	fruits unadjusted(g)
Digestive congenital anomalies	Male	0-6 days	65-69 years	0	vegetables unadjusted(g)
Digestive congenital anomalies	Male	0-6 days	65-69 years	1	Maternal alcohol consumption during pregnancy (proportion)
Digestive congenital anomalies	Male	0-6 days	65-69 years	-1	Healthcare access and quality index
Other congenital birth defects	Female	0-6 days	65-69 years	-1	Legality of Abortion
Other congenital birth defects	Female	0-6 days	65-69 years	1	Alcohol (liters per capita)
Other congenital birth defects	Female	0-6 days	65-69 years	-1	Antenatal Care (1 visit) Coverage (proportion)
Other congenital birth defects	Female	0-6 days	65-69 years	-1	Antenatal Care (4 visits) Coverage (proportion)
Other congenital birth defects	Female	0-6 days	65-69 years	1	Diabetes Age-Standardized Prevalence (proportion)
Other congenital birth defects	Female	0-6 days	65-69 years	-1	Education (years per capita)
Other congenital birth defects	Female	0-6 days	65-69 years	-1	In-Facility Delivery (proportion)
Other congenital birth defects	Female	0-6 days	65-69 years	-1	LDI (I$ per capita)
Other congenital birth defects	Female	0-6 days	65-69 years	1	Live Births 35+ (proportion)
Other congenital birth defects	Female	0-6 days	65-69 years	1	Indoor Air Pollution (All Cooking Fuels)
Other congenital birth defects	Female	0-6 days	65-69 years	1	Smoking Prevalence (Reproductive Age Standardized)
Other congenital birth defects	Female	0-6 days	65-69 years	-1	Socio-demographic Index
Other congenital birth defects	Female	0-6 days	65-69 years	1	Maternal alcohol consumption during pregnancy (proportion)
Other congenital birth defects	Female	0-6 days	65-69 years	-1	Healthcare access and quality index
Other congenital birth defects	Male	0-6 days	65-69 years	-1	Legality of Abortion
Other congenital birth defects	Male	0-6 days	65-69 years	1	Alcohol (liters per capita)
Other congenital birth defects	Male	0-6 days	65-69 years	-1	Antenatal Care (1 visit) Coverage (proportion)
Other congenital birth defects	Male	0-6 days	65-69 years	-1	Antenatal Care (4 visits) Coverage (proportion)
Other congenital birth defects	Male	0-6 days	65-69 years	1	Diabetes Age-Standardized Prevalence (proportion)
Other congenital birth defects	Male	0-6 days	65-69 years	-1	Education (years per capita)
Other congenital birth defects	Male	0-6 days	65-69 years	-1	In-Facility Delivery (proportion)
Other congenital birth defects	Male	0-6 days	65-69 years	-1	LDI (I$ per capita)
Other congenital birth defects	Male	0-6 days	65-69 years	1	Live Births 35+ (proportion)
Other congenital birth defects	Male	0-6 days	65-69 years	1	Indoor Air Pollution (All Cooking Fuels)
Other congenital birth defects	Male	0-6 days	65-69 years	1	Smoking Prevalence (Reproductive Age Standardized)
Other congenital birth defects	Male	0-6 days	65-69 years	-1	Socio-demographic Index
Other congenital birth defects	Male	0-6 days	65-69 years	1	Maternal alcohol consumption during pregnancy (proportion)
Other congenital birth defects	Male	0-6 days	65-69 years	-1	Healthcare access and quality index
Skin and subcutaneous diseases	Male	28-364 days	95+ years	1	Alcohol (liters per capita)
Skin and subcutaneous diseases	Male	28-364 days	95+ years	1	Cumulative Cigarettes (10 Years)
Skin and subcutaneous diseases	Male	28-364 days	95+ years	1	Cumulative Cigarettes (5 Years)
Skin and subcutaneous diseases	Male	28-364 days	95+ years	-1	Education (years per capita)
Skin and subcutaneous diseases	Male	28-364 days	95+ years	-1	LDI (I$ per capita)
Skin and subcutaneous diseases	Male	28-364 days	95+ years	1	Smoking Prevalence
Skin and subcutaneous diseases	Male	28-364 days	95+ years	-1	Improved Water Source (proportion with access)
Skin and subcutaneous diseases	Male	28-364 days	95+ years	1	SEV unsafe sanitation
Skin and subcutaneous diseases	Male	28-364 days	95+ years	0	Socio-demographic Index
Skin and subcutaneous diseases	Male	28-364 days	95+ years	-1	Healthcare access and quality index
Skin and subcutaneous diseases	Female	28-364 days	95+ years	1	Alcohol (liters per capita)
Skin and subcutaneous diseases	Female	28-364 days	95+ years	1	Cumulative Cigarettes (10 Years)
Skin and subcutaneous diseases	Female	28-364 days	95+ years	1	Cumulative Cigarettes (5 Years)
Skin and subcutaneous diseases	Female	28-364 days	95+ years	-1	Education (years per capita)
Skin and subcutaneous diseases	Female	28-364 days	95+ years	-1	LDI (I$ per capita)
Skin and subcutaneous diseases	Female	28-364 days	95+ years	1	Smoking Prevalence
Skin and subcutaneous diseases	Female	28-364 days	95+ years	-1	Improved Water Source (proportion with access)
Skin and subcutaneous diseases	Female	28-364 days	95+ years	1	SEV unsafe sanitation
Skin and subcutaneous diseases	Female	28-364 days	95+ years	0	Socio-demographic Index
Skin and subcutaneous diseases	Female	28-364 days	95+ years	-1	Healthcare access and quality index
Cellulitis	Male	28-364 days	95+ years	0	Education (years per capita)
Cellulitis	Male	28-364 days	95+ years	0	LDI (I$ per capita)
Cellulitis	Male	28-364 days	95+ years	-1	Healthcare access and quality index
Cellulitis	Female	28-364 days	95+ years	0	Education (years per capita)

Cause	Sex	Age start	Age end	Direction	Covariate
Cellulitis	Female	28-364 days	95+ years	0	LDI (I$ per capita)
Cellulitis	Female	28-364 days	95+ years	-1	Healthcare access and quality index
Pyoderma	Female	0-6 days	95+ years	1	Alcohol (liters per capita)
Pyoderma	Female	0-6 days	95+ years	1	Cumulative Cigarettes (10 Years)
Pyoderma	Female	0-6 days	95+ years	1	Cumulative Cigarettes (5 Years)
Pyoderma	Female	0-6 days	95+ years	-1	Education (years per capita)
Pyoderma	Female	0-6 days	95+ years	-1	LDI (I$ per capita)
Pyoderma	Female	0-6 days	95+ years	1	Smoking Prevalence
Pyoderma	Female	0-6 days	95+ years	-1	Improved Water Source (proportion with access)
Pyoderma	Female	0-6 days	95+ years	1	SEV unsafe sanitation
Pyoderma	Female	0-6 days	95+ years	0	Socio-demographic Index
Pyoderma	Female	0-6 days	95+ years	-1	Healthcare access and quality index
Pyoderma	Male	0-6 days	95+ years	1	Alcohol (liters per capita)
Pyoderma	Male	0-6 days	95+ years	1	Cumulative Cigarettes (10 Years)
Pyoderma	Male	0-6 days	95+ years	1	Cumulative Cigarettes (5 Years)
Pyoderma	Male	0-6 days	95+ years	-1	Education (years per capita)
Pyoderma	Male	0-6 days	95+ years	-1	LDI (I$ per capita)
Pyoderma	Male	0-6 days	95+ years	1	Smoking Prevalence
Pyoderma	Male	0-6 days	95+ years	-1	Improved Water Source (proportion with access)
Pyoderma	Male	0-6 days	95+ years	1	SEV unsafe sanitation
Pyoderma	Male	0-6 days	95+ years	0	Socio-demographic Index
Pyoderma	Male	0-6 days	95+ years	-1	Healthcare access and quality index
Decubitus ulcer	Male	1-4 years	95+ years	1	Alcohol (liters per capita)
Decubitus ulcer	Male	1-4 years	95+ years	1	Cumulative Cigarettes (10 Years)
Decubitus ulcer	Male	1-4 years	95+ years	1	Cumulative Cigarettes (5 Years)
Decubitus ulcer	Male	1-4 years	95+ years	1	Diabetes Fasting Plasma Glucose (mmol/L)
Decubitus ulcer	Male	1-4 years	95+ years	-1	Education (years per capita)
Decubitus ulcer	Male	1-4 years	95+ years	-1	Health System Access 2 (unitless)
Decubitus ulcer	Male	1-4 years	95+ years	-1	LDI (I$ per capita)
Decubitus ulcer	Male	1-4 years	95+ years	1	Smoking Prevalence
Decubitus ulcer	Male	1-4 years	95+ years	-1	Improved Water Source (proportion with access)
Decubitus ulcer	Male	1-4 years	95+ years	1	Prevalence of obesity
Decubitus ulcer	Male	1-4 years	95+ years	1	SEV unsafe sanitation
Decubitus ulcer	Male	1-4 years	95+ years	0	Socio-demographic Index
Decubitus ulcer	Male	1-4 years	95+ years	-1	Healthcare access and quality index
Decubitus ulcer	Female	1-4 years	95+ years	1	Alcohol (liters per capita)
Decubitus ulcer	Female	1-4 years	95+ years	1	Cumulative Cigarettes (10 Years)
Decubitus ulcer	Female	1-4 years	95+ years	1	Cumulative Cigarettes (5 Years)
Decubitus ulcer	Female	1-4 years	95+ years	1	Diabetes Fasting Plasma Glucose (mmol/L)
Decubitus ulcer	Female	1-4 years	95+ years	-1	Education (years per capita)
Decubitus ulcer	Female	1-4 years	95+ years	-1	Health System Access 2 (unitless)
Decubitus ulcer	Female	1-4 years	95+ years	-1	LDI (I$ per capita)
Decubitus ulcer	Female	1-4 years	95+ years	1	Smoking Prevalence
Decubitus ulcer	Female	1-4 years	95+ years	-1	Improved Water Source (proportion with access)
Decubitus ulcer	Female	1-4 years	95+ years	1	Prevalence of obesity
Decubitus ulcer	Female	1-4 years	95+ years	1	SEV unsafe sanitation
Decubitus ulcer	Female	1-4 years	95+ years	0	Socio-demographic Index
Decubitus ulcer	Female	1-4 years	95+ years	-1	Healthcare access and quality index
Other skin and subcutaneous diseases	Male	28-364 days	95+ years	1	Alcohol (liters per capita)
Other skin and subcutaneous diseases	Male	28-364 days	95+ years	1	Cumulative Cigarettes (10 Years)
Other skin and subcutaneous diseases	Male	28-364 days	95+ years	1	Cumulative Cigarettes (5 Years)
Other skin and subcutaneous diseases	Male	28-364 days	95+ years	-1	Education (years per capita)
Other skin and subcutaneous diseases	Male	28-364 days	95+ years	-1	Health System Access 2 (unitless)
Other skin and subcutaneous diseases	Male	28-364 days	95+ years	-1	LDI (I$ per capita)
Other skin and subcutaneous diseases	Male	28-364 days	95+ years	1	Underweight (proportion <2SD weight for age, <5 years)
Other skin and subcutaneous diseases	Male	28-364 days	95+ years	1	Smoking Prevalence
Other skin and subcutaneous diseases	Male	28-364 days	95+ years	-1	Improved Water Source (proportion with access)
Other skin and subcutaneous diseases	Male	28-364 days	95+ years	1	SEV unsafe sanitation
Other skin and subcutaneous diseases	Male	28-364 days	95+ years	0	Socio-demographic Index
Other skin and subcutaneous diseases	Male	28-364 days	95+ years	-1	Healthcare access and quality index
Other skin and subcutaneous diseases	Female	28-364 days	95+ years	1	Alcohol (liters per capita)
Other skin and subcutaneous diseases	Female	28-364 days	95+ years	1	Cumulative Cigarettes (10 Years)
Other skin and subcutaneous diseases	Female	28-364 days	95+ years	1	Cumulative Cigarettes (5 Years)
Other skin and subcutaneous diseases	Female	28-364 days	95+ years	-1	Education (years per capita)
Other skin and subcutaneous diseases	Female	28-364 days	95+ years	-1	Health System Access 2 (unitless)
Other skin and subcutaneous diseases	Female	28-364 days	95+ years	-1	LDI (I$ per capita)
Other skin and subcutaneous diseases	Female	28-364 days	95+ years	1	Underweight (proportion <2SD weight for age, <5 years)
Other skin and subcutaneous diseases	Female	28-364 days	95+ years	1	Smoking Prevalence
Other skin and subcutaneous diseases	Female	28-364 days	95+ years	-1	Improved Water Source (proportion with access)
Other skin and subcutaneous diseases	Female	28-364 days	95+ years	1	SEV unsafe sanitation
Other skin and subcutaneous diseases	Female	28-364 days	95+ years	0	Socio-demographic Index
Other skin and subcutaneous diseases	Female	28-364 days	95+ years	-1	Healthcare access and quality index
Sudden infant death syndrome	Male	7-27 days	28-364 days	-1	Education (years per capita)
Sudden infant death syndrome	Male	7-27 days	28-364 days	-1	In-Facility Delivery (proportion)
Sudden infant death syndrome	Male	7-27 days	28-364 days	0	LDI (I$ per capita)
Sudden infant death syndrome	Male	7-27 days	28-364 days	1	Underweight (proportion <2SD weight for age, <5 years)
Sudden infant death syndrome	Male	7-27 days	28-364 days	1	Indoor Air Pollution (All Cooking Fuels)
Sudden infant death syndrome	Male	7-27 days	28-364 days	-1	Skilled Birth Attendance (proportion)
Sudden infant death syndrome	Male	7-27 days	28-364 days	1	Smoking Prevalence (Reproductive Age Standardized)
Sudden infant death syndrome	Male	7-27 days	28-364 days	1	Total Fertility Rate
Sudden infant death syndrome	Male	7-27 days	28-364 days	-1	Health System Access (capped)
Sudden infant death syndrome	Male	7-27 days	28-364 days	0	Socio-demographic Index
Sudden infant death syndrome	Male	7-27 days	28-364 days	-1	Healthcare access and quality index
Sudden infant death syndrome	Female	7-27 days	28-364 days	-1	Education (years per capita)
Sudden infant death syndrome	Female	7-27 days	28-364 days	-1	In-Facility Delivery (proportion)
Sudden infant death syndrome	Female	7-27 days	28-364 days	0	LDI (I$ per capita)
Sudden infant death syndrome	Female	7-27 days	28-364 days	1	Underweight (proportion <2SD weight for age, <5 years)
Sudden infant death syndrome	Female	7-27 days	28-364 days	1	Indoor Air Pollution (All Cooking Fuels)
Sudden infant death syndrome	Female	7-27 days	28-364 days	-1	Skilled Birth Attendance (proportion)
Sudden infant death syndrome	Female	7-27 days	28-364 days	1	Smoking Prevalence (Reproductive Age Standardized)
Sudden infant death syndrome	Female	7-27 days	28-364 days	1	Total Fertility Rate
Sudden infant death syndrome	Female	7-27 days	28-364 days	0	Socio-demographic Index
Transport injuries	Female	0-6 days	95+ years	1	Alcohol (liters per capita)
Transport injuries	Female	0-6 days	95+ years	-1	Education (years per capita)
Transport injuries	Female	0-6 days	95+ years	0	LDI (I$ per capita)

Cause	Sex	Age start	Age end	Direction	Covariate
Transport injuries	Female	0-6 days	95+ years	0	Population Density (300-500 ppl/sqkm, proportion)
Transport injuries	Female	0-6 days	95+ years	0	Population Density (500-1000 ppl/sqkm, proportion)
Transport injuries	Female	0-6 days	95+ years	1	Rainfall Quintile 5 (proportion)
Transport injuries	Female	0-6 days	95+ years	1	Vehicles - 2+4 wheels (per capita)
Transport injuries	Female	0-6 days	95+ years	1	Vehicles - 2 wheels fraction (proportion)
Transport injuries	Female	0-6 days	95+ years	-1	Socio-demographic Index
Transport injuries	Female	0-6 days	95+ years	-1	Healthcare access and quality index
Transport injuries	Male	0-6 days	95+ years	1	Alcohol (liters per capita)
Transport injuries	Male	0-6 days	95+ years	-1	Education (years per capita)
Transport injuries	Male	0-6 days	95+ years	0	LDI (I$ per capita)
Transport injuries	Male	0-6 days	95+ years	0	Population Density (300-500 ppl/sqkm, proportion)
Transport injuries	Male	0-6 days	95+ years	0	Population Density (500-1000 ppl/sqkm, proportion)
Transport injuries	Male	0-6 days	95+ years	1	Rainfall Quintile 5 (proportion)
Transport injuries	Male	0-6 days	95+ years	1	Vehicles - 2+4 wheels (per capita)
Transport injuries	Male	0-6 days	95+ years	1	Vehicles - 2 wheels fraction (proportion)
Transport injuries	Male	0-6 days	95+ years	-1	Socio-demographic Index
Transport injuries	Male	0-6 days	95+ years	-1	Healthcare access and quality index
Road injuries	Male	0-6 days	95+ years	1	Alcohol (liters per capita)
Road injuries	Male	0-6 days	95+ years	-1	Education (years per capita)
Road injuries	Male	0-6 days	95+ years	0	LDI (I$ per capita)
Road injuries	Male	0-6 days	95+ years	1	Population 15 to 30 (proportion)
Road injuries	Male	0-6 days	95+ years	0	Population Density (300-500 ppl/sqkm, proportion)
Road injuries	Male	0-6 days	95+ years	0	Population Density (500-1000 ppl/sqkm, proportion)
Road injuries	Male	0-6 days	95+ years	1	Vehicles - 2+4 wheels (per capita)
Road injuries	Male	0-6 days	95+ years	1	Vehicles - 2 wheels (per capita)
Road injuries	Male	0-6 days	95+ years	1	Vehicles - 4 wheels (per capita)
Road injuries	Male	0-6 days	95+ years	1	Vehicles - 2 wheels fraction (proportion)
Road injuries	Male	0-6 days	95+ years	1	Log-transformed SEV scalar: Road Inj
Road injuries	Male	0-6 days	95+ years	-1	Socio-demographic Index
Road injuries	Male	0-6 days	95+ years	-1	Healthcare access and quality index
Road injuries	Female	0-6 days	95+ years	1	Alcohol (liters per capita)
Road injuries	Female	0-6 days	95+ years	-1	Education (years per capita)
Road injuries	Female	0-6 days	95+ years	0	LDI (I$ per capita)
Road injuries	Female	0-6 days	95+ years	1	Population 15 to 30 (proportion)
Road injuries	Female	0-6 days	95+ years	0	Population Density (300-500 ppl/sqkm, proportion)
Road injuries	Female	0-6 days	95+ years	0	Population Density (500-1000 ppl/sqkm, proportion)
Road injuries	Female	0-6 days	95+ years	1	Vehicles - 2+4 wheels (per capita)
Road injuries	Female	0-6 days	95+ years	1	Vehicles - 2 wheels (per capita)
Road injuries	Female	0-6 days	95+ years	1	Vehicles - 4 wheels (per capita)
Road injuries	Female	0-6 days	95+ years	1	Vehicles - 2 wheels fraction (proportion)
Road injuries	Female	0-6 days	95+ years	1	Log-transformed SEV scalar: Road Inj
Road injuries	Female	0-6 days	95+ years	-1	Socio-demographic Index
Road injuries	Female	0-6 days	95+ years	-1	Healthcare access and quality index
Pedestrian road injuries	Female	0-6 days	95+ years	1	Alcohol (liters per capita)
Pedestrian road injuries	Female	0-6 days	95+ years	-1	Education (years per capita)
Pedestrian road injuries	Female	0-6 days	95+ years	0	LDI (I$ per capita)
Pedestrian road injuries	Female	0-6 days	95+ years	0	Population Density (300-500 ppl/sqkm, proportion)
Pedestrian road injuries	Female	0-6 days	95+ years	0	Population Density (500-1000 ppl/sqkm, proportion)
Pedestrian road injuries	Female	0-6 days	95+ years	1	Rainfall Quintile 5 (proportion)
Pedestrian road injuries	Female	0-6 days	95+ years	1	Vehicles - 2+4 wheels (per capita)
Pedestrian road injuries	Female	0-6 days	95+ years	1	Vehicles - 2 wheels fraction (proportion)
Pedestrian road injuries	Female	0-6 days	95+ years	1	Log-transformed SEV scalar: Pedest
Pedestrian road injuries	Female	0-6 days	95+ years	-1	Socio-demographic Index
Pedestrian road injuries	Female	0-6 days	95+ years	-1	Healthcare access and quality index
Pedestrian road injuries	Male	0-6 days	95+ years	1	Alcohol (liters per capita)
Pedestrian road injuries	Male	0-6 days	95+ years	-1	Education (years per capita)
Pedestrian road injuries	Male	0-6 days	95+ years	0	LDI (I$ per capita)
Pedestrian road injuries	Male	0-6 days	95+ years	0	Population Density (300-500 ppl/sqkm, proportion)
Pedestrian road injuries	Male	0-6 days	95+ years	0	Population Density (500-1000 ppl/sqkm, proportion)
Pedestrian road injuries	Male	0-6 days	95+ years	1	Rainfall Quintile 5 (proportion)
Pedestrian road injuries	Male	0-6 days	95+ years	1	Vehicles - 2+4 wheels (per capita)
Pedestrian road injuries	Male	0-6 days	95+ years	1	Vehicles - 2 wheels fraction (proportion)
Pedestrian road injuries	Male	0-6 days	95+ years	1	Log-transformed SEV scalar: Pedest
Pedestrian road injuries	Male	0-6 days	95+ years	-1	Socio-demographic Index
Pedestrian road injuries	Male	0-6 days	95+ years	-1	Healthcare access and quality index
Cyclist road injuries	Female	1-4 years	95+ years	1	Alcohol (liters per capita)
Cyclist road injuries	Female	1-4 years	95+ years	-1	Education (years per capita)
Cyclist road injuries	Female	1-4 years	95+ years	0	LDI (I$ per capita)
Cyclist road injuries	Female	1-4 years	95+ years	0	Population Density (300-500 ppl/sqkm, proportion)
Cyclist road injuries	Female	1-4 years	95+ years	0	Population Density (500-1000 ppl/sqkm, proportion)
Cyclist road injuries	Female	1-4 years	95+ years	1	Vehicles - 2+4 wheels (per capita)
Cyclist road injuries	Female	1-4 years	95+ years	1	Vehicles - 2 wheels fraction (proportion)
Cyclist road injuries	Female	1-4 years	95+ years	1	Log-transformed SEV scalar: Cyclist
Cyclist road injuries	Female	1-4 years	95+ years	0	Socio-demographic Index
Cyclist road injuries	Female	1-4 years	95+ years	-1	Healthcare access and quality index
Cyclist road injuries	Male	1-4 years	95+ years	1	Alcohol (liters per capita)
Cyclist road injuries	Male	1-4 years	95+ years	-1	Education (years per capita)
Cyclist road injuries	Male	1-4 years	95+ years	0	LDI (I$ per capita)
Cyclist road injuries	Male	1-4 years	95+ years	0	Population Density (300-500 ppl/sqkm, proportion)
Cyclist road injuries	Male	1-4 years	95+ years	0	Population Density (500-1000 ppl/sqkm, proportion)
Cyclist road injuries	Male	1-4 years	95+ years	1	Vehicles - 2+4 wheels (per capita)
Cyclist road injuries	Male	1-4 years	95+ years	1	Vehicles - 2 wheels fraction (proportion)
Cyclist road injuries	Male	1-4 years	95+ years	1	Log-transformed SEV scalar: Cyclist
Cyclist road injuries	Male	1-4 years	95+ years	0	Socio-demographic Index
Cyclist road injuries	Male	1-4 years	95+ years	-1	Healthcare access and quality index
Motorcyclist road injuries	Female	0-6 days	95+ years	1	Alcohol (liters per capita)
Motorcyclist road injuries	Female	0-6 days	95+ years	-1	Education (years per capita)
Motorcyclist road injuries	Female	0-6 days	95+ years	0	LDI (I$ per capita)
Motorcyclist road injuries	Female	0-6 days	95+ years	0	Population Density (300-500 ppl/sqkm, proportion)
Motorcyclist road injuries	Female	0-6 days	95+ years	0	Population Density (500-1000 ppl/sqkm, proportion)
Motorcyclist road injuries	Female	0-6 days	95+ years	1	Rainfall Quintile 5 (proportion)
Motorcyclist road injuries	Female	0-6 days	95+ years	1	Vehicles - 2 wheels (per capita)
Motorcyclist road injuries	Female	0-6 days	95+ years	1	Log-transformed SEV scalar: Mot Cyc
Motorcyclist road injuries	Female	0-6 days	95+ years	0	Socio-demographic Index
Motorcyclist road injuries	Female	0-6 days	95+ years	-1	Healthcare access and quality index

Cause	Sex	Age start	Age end	Direction	Covariate
Motorcyclist road injuries	Male	0-6 days	95+ years	1	Alcohol (liters per capita)
Motorcyclist road injuries	Male	0-6 days	95+ years	-1	Education (years per capita)
Motorcyclist road injuries	Male	0-6 days	95+ years	0	LDI (I$ per capita)
Motorcyclist road injuries	Male	0-6 days	95+ years	0	Population Density (300-500 ppl/sqkm, proportion)
Motorcyclist road injuries	Male	0-6 days	95+ years	0	Population Density (500-1000 ppl/sqkm, proportion)
Motorcyclist road injuries	Male	0-6 days	95+ years	1	Rainfall Quintile 5 (proportion)
Motorcyclist road injuries	Male	0-6 days	95+ years	1	Vehicles - 2 wheels (per capita)
Motorcyclist road injuries	Male	0-6 days	95+ years	1	Log-transformed SEV scalar: Mot Cyc
Motorcyclist road injuries	Male	0-6 days	95+ years	0	Socio-demographic Index
Motorcyclist road injuries	Male	0-6 days	95+ years	-1	Healthcare access and quality index
Motor vehicle road injuries	Female	0-6 days	95+ years	1	Alcohol (liters per capita)
Motor vehicle road injuries	Female	0-6 days	95+ years	0	Education (years per capita)
Motor vehicle road injuries	Female	0-6 days	95+ years	0	LDI (I$ per capita)
Motor vehicle road injuries	Female	0-6 days	95+ years	0	Population Density (300-500 ppl/sqkm, proportion)
Motor vehicle road injuries	Female	0-6 days	95+ years	0	Population Density (500-1000 ppl/sqkm, proportion)
Motor vehicle road injuries	Female	0-6 days	95+ years	1	Rainfall Quintile 5 (proportion)
Motor vehicle road injuries	Female	0-6 days	95+ years	1	Vehicles - 4 wheels (per capita)
Motor vehicle road injuries	Female	0-6 days	95+ years	1	Log-transformed SEV scalar: Mot Veh
Motor vehicle road injuries	Female	0-6 days	95+ years	0	Socio-demographic Index
Motor vehicle road injuries	Female	0-6 days	95+ years	-1	Healthcare access and quality index
Motor vehicle road injuries	Male	0-6 days	95+ years	1	Alcohol (liters per capita)
Motor vehicle road injuries	Male	0-6 days	95+ years	0	Education (years per capita)
Motor vehicle road injuries	Male	0-6 days	95+ years	0	LDI (I$ per capita)
Motor vehicle road injuries	Male	0-6 days	95+ years	0	Population Density (300-500 ppl/sqkm, proportion)
Motor vehicle road injuries	Male	0-6 days	95+ years	0	Population Density (500-1000 ppl/sqkm, proportion)
Motor vehicle road injuries	Male	0-6 days	95+ years	1	Rainfall Quintile 5 (proportion)
Motor vehicle road injuries	Male	0-6 days	95+ years	1	Vehicles - 4 wheels (per capita)
Motor vehicle road injuries	Male	0-6 days	95+ years	1	Log-transformed SEV scalar: Mot Veh
Motor vehicle road injuries	Male	0-6 days	95+ years	0	Socio-demographic Index
Motor vehicle road injuries	Male	0-6 days	95+ years	-1	Healthcare access and quality index
Other road injuries	Female	0-6 days	95+ years	1	Alcohol (liters per capita)
Other road injuries	Female	0-6 days	95+ years	0	LDI (I$ per capita)
Other road injuries	Female	0-6 days	95+ years	1	Rainfall Quintile 5 (proportion)
Other road injuries	Female	0-6 days	95+ years	1	Vehicles - 2+4 wheels (per capita)
Other road injuries	Female	0-6 days	95+ years	1	Vehicles - 2 wheels fraction (proportion)
Other road injuries	Female	0-6 days	95+ years	1	Log-transformed SEV scalar: Oth Road
Other road injuries	Female	0-6 days	95+ years	-1	Socio-demographic Index
Other road injuries	Female	0-6 days	95+ years	-1	Healthcare access and quality index
Other road injuries	Male	0-6 days	95+ years	1	Alcohol (liters per capita)
Other road injuries	Male	0-6 days	95+ years	0	LDI (I$ per capita)
Other road injuries	Male	0-6 days	95+ years	1	Rainfall Quintile 5 (proportion)
Other road injuries	Male	0-6 days	95+ years	1	Vehicles - 2+4 wheels (per capita)
Other road injuries	Male	0-6 days	95+ years	1	Vehicles - 2 wheels fraction (proportion)
Other road injuries	Male	0-6 days	95+ years	1	Log-transformed SEV scalar: Oth Road
Other road injuries	Male	0-6 days	95+ years	-1	Socio-demographic Index
Other road injuries	Male	0-6 days	95+ years	-1	Healthcare access and quality index
Other transport injuries	Female	0-6 days	95+ years	1	Alcohol (liters per capita)
Other transport injuries	Female	0-6 days	95+ years	0	Education (years per capita)
Other transport injuries	Female	0-6 days	95+ years	0	LDI (I$ per capita)
Other transport injuries	Female	0-6 days	95+ years	0	Population Density (300-500 ppl/sqkm, proportion)
Other transport injuries	Female	0-6 days	95+ years	0	Population Density (500-1000 ppl/sqkm, proportion)
Other transport injuries	Female	0-6 days	95+ years	1	Rainfall Quintile 5 (proportion)
Other transport injuries	Female	0-6 days	95+ years	1	Vehicles - 2+4 wheels (per capita)
Other transport injuries	Female	0-6 days	95+ years	1	Vehicles - 2 wheels fraction (proportion)
Other transport injuries	Female	0-6 days	95+ years	1	Log-transformed SEV scalar: Oth Trans
Other transport injuries	Female	0-6 days	95+ years	0	Socio-demographic Index
Other transport injuries	Female	0-6 days	95+ years	-1	Healthcare access and quality index
Other transport injuries	Male	0-6 days	95+ years	1	Alcohol (liters per capita)
Other transport injuries	Male	0-6 days	95+ years	0	Education (years per capita)
Other transport injuries	Male	0-6 days	95+ years	1	LDI (I$ per capita)
Other transport injuries	Male	0-6 days	95+ years	0	Population Density (300-500 ppl/sqkm, proportion)
Other transport injuries	Male	0-6 days	95+ years	0	Population Density (500-1000 ppl/sqkm, proportion)
Other transport injuries	Male	0-6 days	95+ years	1	Rainfall Quintile 5 (proportion)
Other transport injuries	Male	0-6 days	95+ years	1	Vehicles - 2+4 wheels (per capita)
Other transport injuries	Male	0-6 days	95+ years	1	Vehicles - 2 wheels fraction (proportion)
Other transport injuries	Male	0-6 days	95+ years	1	Log-transformed SEV scalar: Oth Trans
Other transport injuries	Male	0-6 days	95+ years	0	Socio-demographic Index
Other transport injuries	Male	0-6 days	95+ years	-1	Healthcare access and quality index
Unintentional injuries	Female	28-364 days	95+ years	1	Alcohol (liters per capita)
Unintentional injuries	Female	28-364 days	95+ years	1	Cumulative Cigarettes (5 Years)
Unintentional injuries	Female	28-364 days	95+ years	1	Diabetes Fasting Plasma Glucose (mmol/L)
Unintentional injuries	Female	28-364 days	95+ years	-1	Education (years per capita)
Unintentional injuries	Female	28-364 days	95+ years	-1	Health System Access 2 (unitless)
Unintentional injuries	Female	28-364 days	95+ years	-1	LDI (I$ per capita)
Unintentional injuries	Female	28-364 days	95+ years	1	Underweight (proportion <2SD weight for age, <5 years)
Unintentional injuries	Female	28-364 days	95+ years	1	Indoor Air Pollution (All Cooking Fuels)
Unintentional injuries	Female	28-364 days	95+ years	1	Population Density (500-1000 ppl/sqkm, proportion)
Unintentional injuries	Female	28-364 days	95+ years	1	Population Density (over 1000 ppl/sqkm, proportion)
Unintentional injuries	Female	28-364 days	95+ years	1	Smoking Prevalence
Unintentional injuries	Male	28-364 days	95+ years	1	Alcohol (liters per capita)
Unintentional injuries	Male	28-364 days	95+ years	1	Cumulative Cigarettes (5 Years)
Unintentional injuries	Male	28-364 days	95+ years	1	Diabetes Fasting Plasma Glucose (mmol/L)
Unintentional injuries	Male	28-364 days	95+ years	-1	Education (years per capita)
Unintentional injuries	Male	28-364 days	95+ years	-1	Health System Access 2 (unitless)
Unintentional injuries	Male	28-364 days	95+ years	-1	LDI (I$ per capita)
Unintentional injuries	Male	28-364 days	95+ years	1	Underweight (proportion <2SD weight for age, <5 years)
Unintentional injuries	Male	28-364 days	95+ years	1	Indoor Air Pollution (All Cooking Fuels)
Unintentional injuries	Male	28-364 days	95+ years	1	Population Density (500-1000 ppl/sqkm, proportion)
Unintentional injuries	Male	28-364 days	95+ years	1	Population Density (over 1000 ppl/sqkm, proportion)
Unintentional injuries	Male	28-364 days	95+ years	1	Smoking Prevalence
Falls	Female	0-6 days	95+ years	1	Alcohol (liters per capita)
Falls	Female	0-6 days	95+ years	0	LDI (I$ per capita)
Falls	Female	0-6 days	95+ years	-1	ln-Milk (kcal per capita)
Falls	Female	0-6 days	95+ years	1	Elevation Over 1500m (proportion)
Falls	Female	0-6 days	95+ years	1	Log-transformed SEV scalar: Falls

Cause	Sex	Age start	Age end	Direction	Covariate
Falls	Female	0-6 days	95+ years	0	Socio-demographic Index
Falls	Female	0-6 days	95+ years	-1	Healthcare access and quality index
Falls	Male	0-6 days	95+ years	1	Alcohol (liters per capita)
Falls	Male	0-6 days	95+ years	0	LDI (I$ per capita)
Falls	Male	0-6 days	95+ years	-1	ln-Milk (kcal per capita)
Falls	Male	0-6 days	95+ years	1	Elevation Over 1500m (proportion)
Falls	Male	0-6 days	95+ years	1	Log-transformed SEV scalar: Falls
Falls	Male	0-6 days	95+ years	0	Socio-demographic Index
Falls	Male	0-6 days	95+ years	-1	Healthcare access and quality index
Drowning	Female	0-6 days	95+ years	1	Alcohol (liters per capita)
Drowning	Female	0-6 days	95+ years	1	Coastal Population within 10km (proportion)
Drowning	Female	0-6 days	95+ years	-1	Education (years per capita)
Drowning	Female	0-6 days	95+ years	-1	Landlocked Nation (binary)
Drowning	Female	0-6 days	95+ years	0	LDI (I$ per capita)
Drowning	Female	0-6 days	95+ years	1	Elevation Under 100m (proportion)
Drowning	Female	0-6 days	95+ years	-1	Rainfall Quintile 1 (proportion)
Drowning	Female	0-6 days	95+ years	1	Rainfall Quintile 5 (proportion)
Drowning	Female	0-6 days	95+ years	1	Log-transformed SEV scalar: Drown
Drowning	Female	0-6 days	95+ years	-1	Socio-demographic Index
Drowning	Male	0-6 days	95+ years	1	Alcohol (liters per capita)
Drowning	Male	0-6 days	95+ years	1	Coastal Population within 10km (proportion)
Drowning	Male	0-6 days	95+ years	-1	Education (years per capita)
Drowning	Male	0-6 days	95+ years	-1	Landlocked Nation (binary)
Drowning	Male	0-6 days	95+ years	0	LDI (I$ per capita)
Drowning	Male	0-6 days	95+ years	1	Elevation Under 100m (proportion)
Drowning	Male	0-6 days	95+ years	-1	Rainfall Quintile 1 (proportion)
Drowning	Male	0-6 days	95+ years	1	Rainfall Quintile 5 (proportion)
Drowning	Male	0-6 days	95+ years	1	Log-transformed SEV scalar: Drown
Drowning	Male	0-6 days	95+ years	-1	Socio-demographic Index
Fire, heat, and hot substances	Female	0-6 days	95+ years	1	Alcohol (liters per capita)
Fire, heat, and hot substances	Female	0-6 days	95+ years	1	Tobacco (cigarettes per capita)
Fire, heat, and hot substances	Female	0-6 days	95+ years	-1	Education (years per capita)
Fire, heat, and hot substances	Female	0-6 days	95+ years	0	LDI (I$ per capita)
Fire, heat, and hot substances	Female	0-6 days	95+ years	1	Indoor Air Pollution (Biomass Cooking)
Fire, heat, and hot substances	Female	0-6 days	95+ years	0	Population Density (over 1000 ppl/sqkm, proportion)
Fire, heat, and hot substances	Female	0-6 days	95+ years	1	Log-transformed SEV scalar: Fire
Fire, heat, and hot substances	Female	0-6 days	95+ years	-1	Socio-demographic Index
Fire, heat, and hot substances	Female	0-6 days	95+ years	-1	Healthcare access and quality index
Fire, heat, and hot substances	Male	0-6 days	95+ years	1	Alcohol (liters per capita)
Fire, heat, and hot substances	Male	0-6 days	95+ years	1	Tobacco (cigarettes per capita)
Fire, heat, and hot substances	Male	0-6 days	95+ years	-1	Education (years per capita)
Fire, heat, and hot substances	Male	0-6 days	95+ years	0	LDI (I$ per capita)
Fire, heat, and hot substances	Male	0-6 days	95+ years	1	Indoor Air Pollution (Biomass Cooking)
Fire, heat, and hot substances	Male	0-6 days	95+ years	0	Population Density (over 1000 ppl/sqkm, proportion)
Fire, heat, and hot substances	Male	0-6 days	95+ years	1	Log-transformed SEV scalar: Fire
Fire, heat, and hot substances	Male	0-6 days	95+ years	-1	Socio-demographic Index
Fire, heat, and hot substances	Male	0-6 days	95+ years	-1	Healthcare access and quality index
Poisonings	Female	0-6 days	95+ years	-1	Education (years per capita)
Poisonings	Female	0-6 days	95+ years	0	LDI (I$ per capita)
Poisonings	Female	0-6 days	95+ years	1	Opium Cultivation (binary)
Poisonings	Female	0-6 days	95+ years	0	Population Density (over 1000 ppl/sqkm, proportion)
Poisonings	Female	0-6 days	95+ years	0	Population Density (under 150 ppl/sqkm, proportion)
Poisonings	Female	0-6 days	95+ years	1	Log-transformed SEV scalar: Poison
Poisonings	Female	0-6 days	95+ years	-1	Socio-demographic Index
Poisonings	Female	0-6 days	95+ years	-1	Healthcare access and quality index
Poisonings	Male	0-6 days	95+ years	-1	Education (years per capita)
Poisonings	Male	0-6 days	95+ years	0	LDI (I$ per capita)
Poisonings	Male	0-6 days	95+ years	1	Opium Cultivation (binary)
Poisonings	Male	0-6 days	95+ years	0	Population Density (over 1000 ppl/sqkm, proportion)
Poisonings	Male	0-6 days	95+ years	0	Population Density (under 150 ppl/sqkm, proportion)
Poisonings	Male	0-6 days	95+ years	1	Log-transformed SEV scalar: Poison
Poisonings	Male	0-6 days	95+ years	-1	Socio-demographic Index
Poisonings	Male	0-6 days	95+ years	-1	Healthcare access and quality index
Exposure to mechanical forces	Female	0-6 days	95+ years	1	Alcohol (liters per capita)
Exposure to mechanical forces	Female	0-6 days	95+ years	-1	Education (years per capita)
Exposure to mechanical forces	Female	0-6 days	95+ years	0	LDI (I$ per capita)
Exposure to mechanical forces	Female	0-6 days	95+ years	0	Population Density (over 1000 ppl/sqkm, proportion)
Exposure to mechanical forces	Female	0-6 days	95+ years	0	Population Density (under 150 ppl/sqkm, proportion)
Exposure to mechanical forces	Female	0-6 days	95+ years	-1	Socio-demographic Index
Exposure to mechanical forces	Female	0-6 days	95+ years	-1	Healthcare access and quality index
Exposure to mechanical forces	Male	0-6 days	95+ years	1	Alcohol (liters per capita)
Exposure to mechanical forces	Male	0-6 days	95+ years	-1	Education (years per capita)
Exposure to mechanical forces	Male	0-6 days	95+ years	0	LDI (I$ per capita)
Exposure to mechanical forces	Male	0-6 days	95+ years	0	Population Density (over 1000 ppl/sqkm, proportion)
Exposure to mechanical forces	Male	0-6 days	95+ years	0	Population Density (under 150 ppl/sqkm, proportion)
Exposure to mechanical forces	Male	0-6 days	95+ years	-1	Socio-demographic Index
Exposure to mechanical forces	Male	0-6 days	95+ years	-1	Healthcare access and quality index
Unintentional firearm injuries	Female	0-6 days	95+ years	1	Alcohol (liters per capita)
Unintentional firearm injuries	Female	0-6 days	95+ years	-1	Education (years per capita)
Unintentional firearm injuries	Female	0-6 days	95+ years	-1	Health System Access (unitless)
Unintentional firearm injuries	Female	0-6 days	95+ years	0	LDI (I$ per capita)
Unintentional firearm injuries	Female	0-6 days	95+ years	0	Population Density (over 1000 ppl/sqkm, proportion)
Unintentional firearm injuries	Female	0-6 days	95+ years	0	Population Density (under 150 ppl/sqkm, proportion)
Unintentional firearm injuries	Female	0-6 days	95+ years	1	Log-transformed SEV scalar: Mech Gun
Unintentional firearm injuries	Female	0-6 days	95+ years	-1	Socio-demographic Index
Unintentional firearm injuries	Female	0-6 days	95+ years	-1	Healthcare access and quality index
Unintentional firearm injuries	Male	0-6 days	95+ years	1	Alcohol (liters per capita)
Unintentional firearm injuries	Male	0-6 days	95+ years	-1	Education (years per capita)
Unintentional firearm injuries	Male	0-6 days	95+ years	-1	Health System Access (unitless)
Unintentional firearm injuries	Male	0-6 days	95+ years	0	LDI (I$ per capita)
Unintentional firearm injuries	Male	0-6 days	95+ years	0	Population Density (over 1000 ppl/sqkm, proportion)
Unintentional firearm injuries	Male	0-6 days	95+ years	0	Population Density (under 150 ppl/sqkm, proportion)
Unintentional firearm injuries	Male	0-6 days	95+ years	1	Log-transformed SEV scalar: Mech Gun
Unintentional firearm injuries	Male	0-6 days	95+ years	-1	Socio-demographic Index
Unintentional firearm injuries	Male	0-6 days	95+ years	-1	Healthcare access and quality index

Cause	Sex	Age start	Age end	Direction	Covariate
Unintentional suffocation	Female	0-6 days	95+ years	1	Alcohol (liters per capita)
Unintentional suffocation	Female	0-6 days	95+ years	-1	Education (years per capita)
Unintentional suffocation	Female	0-6 days	95+ years	0	LDI (I$ per capita)
Unintentional suffocation	Female	0-6 days	95+ years	0	Population Density (over 1000 ppl/sqkm, proportion)
Unintentional suffocation	Female	0-6 days	95+ years	0	Population Density (under 150 ppl/sqkm, proportion)
Unintentional suffocation	Female	0-6 days	95+ years	1	Log-transformed SEV scalar: Mech Suff
Unintentional suffocation	Female	0-6 days	95+ years	0	Socio-demographic Index
Unintentional suffocation	Female	0-6 days	95+ years	-1	Healthcare access and quality index
Unintentional suffocation	Male	0-6 days	95+ years	1	Alcohol (liters per capita)
Unintentional suffocation	Male	0-6 days	95+ years	-1	Education (years per capita)
Unintentional suffocation	Male	0-6 days	95+ years	0	LDI (I$ per capita)
Unintentional suffocation	Male	0-6 days	95+ years	0	Population Density (over 1000 ppl/sqkm, proportion)
Unintentional suffocation	Male	0-6 days	95+ years	0	Population Density (under 150 ppl/sqkm, proportion)
Unintentional suffocation	Male	0-6 days	95+ years	1	Log-transformed SEV scalar: Mech Suff
Unintentional suffocation	Male	0-6 days	95+ years	0	Socio-demographic Index
Unintentional suffocation	Male	0-6 days	95+ years	-1	Healthcare access and quality index
Other exposure to mechanical forces	Female	0-6 days	95+ years	1	Alcohol (liters per capita)
Other exposure to mechanical forces	Female	0-6 days	95+ years	-1	Education (years per capita)
Other exposure to mechanical forces	Female	0-6 days	95+ years	-1	Health System Access (unitless)
Other exposure to mechanical forces	Female	0-6 days	95+ years	0	LDI (I$ per capita)
Other exposure to mechanical forces	Female	0-6 days	95+ years	0	Population Density (over 1000 ppl/sqkm, proportion)
Other exposure to mechanical forces	Female	0-6 days	95+ years	0	Population Density (under 150 ppl/sqkm, proportion)
Other exposure to mechanical forces	Female	0-6 days	95+ years	1	Log-transformed SEV scalar: Oth Mech
Other exposure to mechanical forces	Female	0-6 days	95+ years	-1	Socio-demographic Index
Other exposure to mechanical forces	Female	0-6 days	95+ years	-1	Healthcare access and quality index
Other exposure to mechanical forces	Male	0-6 days	95+ years	1	Alcohol (liters per capita)
Other exposure to mechanical forces	Male	0-6 days	95+ years	-1	Education (years per capita)
Other exposure to mechanical forces	Male	0-6 days	95+ years	-1	Health System Access (unitless)
Other exposure to mechanical forces	Male	0-6 days	95+ years	0	LDI (I$ per capita)
Other exposure to mechanical forces	Male	0-6 days	95+ years	0	Population Density (over 1000 ppl/sqkm, proportion)
Other exposure to mechanical forces	Male	0-6 days	95+ years	0	Population Density (under 150 ppl/sqkm, proportion)
Other exposure to mechanical forces	Male	0-6 days	95+ years	1	Log-transformed SEV scalar: Oth Mech
Other exposure to mechanical forces	Male	0-6 days	95+ years	-1	Socio-demographic Index
Other exposure to mechanical forces	Male	0-6 days	95+ years	-1	Healthcare access and quality index
Adverse effects of medical treatment	Female	0-6 days	95+ years	0	LDI (I$ per capita)
Adverse effects of medical treatment	Female	0-6 days	95+ years	0	Socio-demographic Index
Adverse effects of medical treatment	Female	0-6 days	95+ years	0	Healthcare access and quality index
Adverse effects of medical treatment	Male	0-6 days	95+ years	0	LDI (I$ per capita)
Adverse effects of medical treatment	Male	0-6 days	95+ years	0	Socio-demographic Index
Adverse effects of medical treatment	Male	0-6 days	95+ years	0	Healthcare access and quality index
Animal contact	Female	0-6 days	95+ years	1	Alcohol (liters per capita)
Animal contact	Female	0-6 days	95+ years	-1	Education (years per capita)
Animal contact	Female	0-6 days	95+ years	0	LDI (I$ per capita)
Animal contact	Female	0-6 days	95+ years	0	Elevation Over 1500m (proportion)
Animal contact	Female	0-6 days	95+ years	1	Population 15 to 30 (proportion)
Animal contact	Female	0-6 days	95+ years	0	Population Density (over 1000 ppl/sqkm, proportion)
Animal contact	Female	0-6 days	95+ years	0	Population Density (under 150 ppl/sqkm, proportion)
Animal contact	Female	0-6 days	95+ years	0	Elevation Under 100m (proportion)
Animal contact	Female	0-6 days	95+ years	1	Log-transformed SEV scalar: Animal
Animal contact	Female	0-6 days	95+ years	-1	Socio-demographic Index
Animal contact	Female	0-6 days	95+ years	-1	Healthcare access and quality index
Animal contact	Male	0-6 days	95+ years	1	Alcohol (liters per capita)
Animal contact	Male	0-6 days	95+ years	-1	Education (years per capita)
Animal contact	Male	0-6 days	95+ years	0	LDI (I$ per capita)
Animal contact	Male	0-6 days	95+ years	0	Elevation Over 1500m (proportion)
Animal contact	Male	0-6 days	95+ years	1	Population 15 to 30 (proportion)
Animal contact	Male	0-6 days	95+ years	0	Population Density (over 1000 ppl/sqkm, proportion)
Animal contact	Male	0-6 days	95+ years	0	Population Density (under 150 ppl/sqkm, proportion)
Animal contact	Male	0-6 days	95+ years	0	Elevation Under 100m (proportion)
Animal contact	Male	0-6 days	95+ years	1	Log-transformed SEV scalar: Animal
Animal contact	Male	0-6 days	95+ years	-1	Socio-demographic Index
Animal contact	Male	0-6 days	95+ years	-1	Healthcare access and quality index
Venomous animal contact	Female	0-6 days	95+ years	1	Alcohol (liters per capita)
Venomous animal contact	Female	0-6 days	95+ years	-1	Education (years per capita)
Venomous animal contact	Female	0-6 days	95+ years	0	LDI (I$ per capita)
Venomous animal contact	Female	0-6 days	95+ years	0	Elevation Over 1500m (proportion)
Venomous animal contact	Female	0-6 days	95+ years	0	Population Density (over 1000 ppl/sqkm, proportion)
Venomous animal contact	Female	0-6 days	95+ years	0	Population Density (under 150 ppl/sqkm, proportion)
Venomous animal contact	Female	0-6 days	95+ years	0	Elevation Under 100m (proportion)
Venomous animal contact	Female	0-6 days	95+ years	1	Log-transformed SEV scalar: Venom
Venomous animal contact	Female	0-6 days	95+ years	-1	Socio-demographic Index
Venomous animal contact	Female	0-6 days	95+ years	-1	Healthcare access and quality index
Venomous animal contact	Male	0-6 days	95+ years	1	Alcohol (liters per capita)
Venomous animal contact	Male	0-6 days	95+ years	-1	Education (years per capita)
Venomous animal contact	Male	0-6 days	95+ years	0	LDI (I$ per capita)
Venomous animal contact	Male	0-6 days	95+ years	0	Elevation Over 1500m (proportion)
Venomous animal contact	Male	0-6 days	95+ years	0	Population Density (over 1000 ppl/sqkm, proportion)
Venomous animal contact	Male	0-6 days	95+ years	0	Population Density (under 150 ppl/sqkm, proportion)
Venomous animal contact	Male	0-6 days	95+ years	0	Elevation Under 100m (proportion)
Venomous animal contact	Male	0-6 days	95+ years	1	Log-transformed SEV scalar: Venom
Venomous animal contact	Male	0-6 days	95+ years	-1	Socio-demographic Index
Venomous animal contact	Male	0-6 days	95+ years	-1	Healthcare access and quality index
Non-venomous animal contact	Female	0-6 days	95+ years	1	Alcohol (liters per capita)
Non-venomous animal contact	Female	0-6 days	95+ years	-1	Education (years per capita)
Non-venomous animal contact	Female	0-6 days	95+ years	0	LDI (I$ per capita)
Non-venomous animal contact	Female	0-6 days	95+ years	0	Elevation Over 1500m (proportion)
Non-venomous animal contact	Female	0-6 days	95+ years	0	Population Density (over 1000 ppl/sqkm, proportion)
Non-venomous animal contact	Female	0-6 days	95+ years	0	Population Density (under 150 ppl/sqkm, proportion)
Non-venomous animal contact	Female	0-6 days	95+ years	0	Elevation Under 100m (proportion)
Non-venomous animal contact	Female	0-6 days	95+ years	1	Log-transformed SEV scalar: Non Ven
Non-venomous animal contact	Female	0-6 days	95+ years	-1	Socio-demographic Index
Non-venomous animal contact	Female	0-6 days	95+ years	-1	Healthcare access and quality index
Non-venomous animal contact	Male	0-6 days	95+ years	1	Alcohol (liters per capita)
Non-venomous animal contact	Male	0-6 days	95+ years	-1	Education (years per capita)
Non-venomous animal contact	Male	0-6 days	95+ years	0	LDI (I$ per capita)

Cause	Sex	Age start	Age end	Direction	Covariate
Non-venomous animal contact	Male	0-6 days	95+ years	0	Elevation Over 1500m (proportion)
Non-venomous animal contact	Male	0-6 days	95+ years	0	Population Density (over 1000 ppl/sqkm, proportion)
Non-venomous animal contact	Male	0-6 days	95+ years	0	Population Density (under 150 ppl/sqkm, proportion)
Non-venomous animal contact	Male	0-6 days	95+ years	0	Elevation Under 100m (proportion)
Non-venomous animal contact	Male	0-6 days	95+ years	1	Log-transformed SEV scalar: Non Ven
Non-venomous animal contact	Male	0-6 days	95+ years	-1	Socio-demographic Index
Non-venomous animal contact	Male	0-6 days	95+ years	-1	Healthcare access and quality index
Foreign body	Female	0-6 days	95+ years	1	Education (years per capita)
Foreign body	Female	0-6 days	95+ years	1	LDI (I$ per capita)
Foreign body	Female	0-6 days	95+ years	1	Indoor Air Pollution (Coal Cooking)
Foreign body	Female	0-6 days	95+ years	1	Population Density (over 1000 ppl/sqkm, proportion)
Foreign body	Female	0-6 days	95+ years	1	Population Over 65 (proportion)
Foreign body	Female	0-6 days	95+ years	0	Socio-demographic Index
Foreign body	Female	0-6 days	95+ years	-1	Healthcare access and quality index
Foreign body	Male	0-6 days	95+ years	1	Education (years per capita)
Foreign body	Male	0-6 days	95+ years	1	LDI (I$ per capita)
Foreign body	Male	0-6 days	95+ years	1	Indoor Air Pollution (Coal Cooking)
Foreign body	Male	0-6 days	95+ years	1	Population Density (over 1000 ppl/sqkm, proportion)
Foreign body	Male	0-6 days	95+ years	1	Population Over 65 (proportion)
Foreign body	Male	0-6 days	95+ years	0	Socio-demographic Index
Foreign body	Male	0-6 days	95+ years	-1	Healthcare access and quality index
Pulmonary aspiration and foreign body in airway	Female	0-6 days	95+ years	1	Alcohol (liters per capita)
Pulmonary aspiration and foreign body in airway	Female	0-6 days	95+ years	0	LDI (I$ per capita)
Pulmonary aspiration and foreign body in airway	Female	0-6 days	95+ years	1	Mean BMI
Pulmonary aspiration and foreign body in airway	Female	0-6 days	95+ years	1	Log-transformed SEV scalar: F Body Asp
Pulmonary aspiration and foreign body in airway	Female	0-6 days	95+ years	0	Socio-demographic Index
Pulmonary aspiration and foreign body in airway	Female	0-6 days	95+ years	-1	Healthcare access and quality index
Pulmonary aspiration and foreign body in airway	Male	0-6 days	95+ years	1	Alcohol (liters per capita)
Pulmonary aspiration and foreign body in airway	Male	0-6 days	95+ years	0	LDI (I$ per capita)
Pulmonary aspiration and foreign body in airway	Male	0-6 days	95+ years	1	Mean BMI
Pulmonary aspiration and foreign body in airway	Male	0-6 days	95+ years	1	Log-transformed SEV scalar: F Body Asp
Pulmonary aspiration and foreign body in airway	Male	0-6 days	95+ years	0	Socio-demographic Index
Pulmonary aspiration and foreign body in airway	Male	0-6 days	95+ years	-1	Healthcare access and quality index
Foreign body in other body part	Female	0-6 days	95+ years	1	Alcohol (liters per capita)
Foreign body in other body part	Female	0-6 days	95+ years	-1	Education (years per capita)
Foreign body in other body part	Female	0-6 days	95+ years	0	LDI (I$ per capita)
Foreign body in other body part	Female	0-6 days	95+ years	0	Elevation Over 1500m (proportion)
Foreign body in other body part	Female	0-6 days	95+ years	0	Population Density (over 1000 ppl/sqkm, proportion)
Foreign body in other body part	Female	0-6 days	95+ years	0	Population Density (under 150 ppl/sqkm, proportion)
Foreign body in other body part	Female	0-6 days	95+ years	0	Elevation Under 100m (proportion)
Foreign body in other body part	Female	0-6 days	95+ years	1	Log-transformed SEV scalar: Oth F Body
Foreign body in other body part	Female	0-6 days	95+ years	-1	Socio-demographic Index
Foreign body in other body part	Female	0-6 days	95+ years	-1	Healthcare access and quality index
Foreign body in other body part	Male	0-6 days	95+ years	1	Alcohol (liters per capita)
Foreign body in other body part	Male	0-6 days	95+ years	-1	Education (years per capita)
Foreign body in other body part	Male	0-6 days	95+ years	0	LDI (I$ per capita)
Foreign body in other body part	Male	0-6 days	95+ years	0	Elevation Over 1500m (proportion)
Foreign body in other body part	Male	0-6 days	95+ years	0	Population Density (over 1000 ppl/sqkm, proportion)
Foreign body in other body part	Male	0-6 days	95+ years	0	Population Density (under 150 ppl/sqkm, proportion)
Foreign body in other body part	Male	0-6 days	95+ years	0	Elevation Under 100m (proportion)
Foreign body in other body part	Male	0-6 days	95+ years	1	Log-transformed SEV scalar: Oth F Body
Foreign body in other body part	Male	0-6 days	95+ years	-1	Socio-demographic Index
Foreign body in other body part	Male	0-6 days	95+ years	-1	Healthcare access and quality index
Other unintentional injuries	Female	0-6 days	95+ years	1	Alcohol (liters per capita)
Other unintentional injuries	Female	0-6 days	95+ years	-1	Education (years per capita)
Other unintentional injuries	Female	0-6 days	95+ years	0	LDI (I$ per capita)
Other unintentional injuries	Female	0-6 days	95+ years	0	Elevation Over 1500m (proportion)
Other unintentional injuries	Female	0-6 days	95+ years	0	Population Density (over 1000 ppl/sqkm, proportion)
Other unintentional injuries	Female	0-6 days	95+ years	0	Population Density (under 150 ppl/sqkm, proportion)
Other unintentional injuries	Female	0-6 days	95+ years	0	Elevation Under 100m (proportion)
Other unintentional injuries	Female	0-6 days	95+ years	1	Vehicles - 2 wheels (per capita)
Other unintentional injuries	Female	0-6 days	95+ years	0	Vehicles - 4 wheels (per capita)
Other unintentional injuries	Female	0-6 days	95+ years	1	Log-transformed SEV scalar: Oth Unint
Other unintentional injuries	Female	0-6 days	95+ years	0	Socio-demographic Index
Other unintentional injuries	Female	0-6 days	95+ years	-1	Healthcare access and quality index
Other unintentional injuries	Male	0-6 days	95+ years	1	Alcohol (liters per capita)
Other unintentional injuries	Male	0-6 days	95+ years	-1	Education (years per capita)
Other unintentional injuries	Male	0-6 days	95+ years	0	LDI (I$ per capita)
Other unintentional injuries	Male	0-6 days	95+ years	0	Elevation Over 1500m (proportion)
Other unintentional injuries	Male	0-6 days	95+ years	0	Population Density (over 1000 ppl/sqkm, proportion)
Other unintentional injuries	Male	0-6 days	95+ years	0	Population Density (under 150 ppl/sqkm, proportion)
Other unintentional injuries	Male	0-6 days	95+ years	0	Elevation Under 100m (proportion)
Other unintentional injuries	Male	0-6 days	95+ years	1	Vehicles - 2 wheels (per capita)
Other unintentional injuries	Male	0-6 days	95+ years	0	Vehicles - 4 wheels (per capita)
Other unintentional injuries	Male	0-6 days	95+ years	1	Log-transformed SEV scalar: Oth Unint
Other unintentional injuries	Male	0-6 days	95+ years	0	Socio-demographic Index
Other unintentional injuries	Male	0-6 days	95+ years	-1	Healthcare access and quality index
Self-harm and interpersonal violence	Female	0-6 days	95+ years	1	Alcohol (liters per capita)
Self-harm and interpersonal violence	Female	0-6 days	95+ years	-1	Education (years per capita)
Self-harm and interpersonal violence	Female	0-6 days	95+ years	0	LDI (I$ per capita)
Self-harm and interpersonal violence	Female	0-6 days	95+ years	0	Elevation Over 1500m (proportion)
Self-harm and interpersonal violence	Female	0-6 days	95+ years	0	Population Density (over 1000 ppl/sqkm, proportion)
Self-harm and interpersonal violence	Female	0-6 days	95+ years	0	Population Density (under 150 ppl/sqkm, proportion)
Self-harm and interpersonal violence	Female	0-6 days	95+ years	0	Elevation Under 100m (proportion)
Self-harm and interpersonal violence	Female	0-6 days	95+ years	1	Log-transformed SEV scalar: Oth Unint
Self-harm and interpersonal violence	Female	0-6 days	95+ years	1	Healthcare access and quality index
Self-harm and interpersonal violence	Male	0-6 days	95+ years	1	Alcohol (liters per capita)
Self-harm and interpersonal violence	Male	0-6 days	95+ years	-1	Education (years per capita)
Self-harm and interpersonal violence	Male	0-6 days	95+ years	0	LDI (I$ per capita)
Self-harm and interpersonal violence	Male	0-6 days	95+ years	0	Elevation Over 1500m (proportion)
Self-harm and interpersonal violence	Male	0-6 days	95+ years	0	Population Density (over 1000 ppl/sqkm, proportion)
Self-harm and interpersonal violence	Male	0-6 days	95+ years	0	Population Density (under 150 ppl/sqkm, proportion)
Self-harm and interpersonal violence	Male	0-6 days	95+ years	0	Elevation Under 100m (proportion)
Self-harm and interpersonal violence	Male	0-6 days	95+ years	1	Log-transformed SEV scalar: Oth Unint
Self-harm and interpersonal violence	Male	0-6 days	95+ years	1	Healthcare access and quality index

Cause	Sex	Age start	Age end	Direction	Covariate
Self-harm	Female	10-14 years	95+ years	1	Alcohol (liters per capita)
Self-harm	Female	10-14 years	95+ years	0	Education (years per capita)
Self-harm	Female	10-14 years	95+ years	0	LDI (I$ per capita)
Self-harm	Female	10-14 years	95+ years	0	Population Density (150-300 ppl/sqkm, proportion)
Self-harm	Female	10-14 years	95+ years	0	Population Density (300-500 ppl/sqkm, proportion)
Self-harm	Female	10-14 years	95+ years	0	Population Density (500-1000 ppl/sqkm, proportion)
Self-harm	Female	10-14 years	95+ years	0	Population Density (over 1000 ppl/sqkm, proportion)
Self-harm	Female	10-14 years	95+ years	0	Population Density (under 150 ppl/sqkm, proportion)
Self-harm	Female	10-14 years	95+ years	-1	Religion (binary, >50% Muslim)
Self-harm	Female	10-14 years	95+ years	1	Log-transformed SEV scalar: Self Harm
Self-harm	Female	10-14 years	95+ years	0	Socio-demographic Index
Self-harm	Female	10-14 years	95+ years	1	Major depressive disorder
Self-harm	Female	10-14 years	95+ years	-1	Healthcare access and quality index
Self-harm	Male	10-14 years	95+ years	1	Alcohol (liters per capita)
Self-harm	Male	10-14 years	95+ years	0	Education (years per capita)
Self-harm	Male	10-14 years	95+ years	0	LDI (I$ per capita)
Self-harm	Male	10-14 years	95+ years	0	Population Density (150-300 ppl/sqkm, proportion)
Self-harm	Male	10-14 years	95+ years	0	Population Density (300-500 ppl/sqkm, proportion)
Self-harm	Male	10-14 years	95+ years	0	Population Density (500-1000 ppl/sqkm, proportion)
Self-harm	Male	10-14 years	95+ years	0	Population Density (over 1000 ppl/sqkm, proportion)
Self-harm	Male	10-14 years	95+ years	0	Population Density (under 150 ppl/sqkm, proportion)
Self-harm	Male	10-14 years	95+ years	-1	Religion (binary, >50% Muslim)
Self-harm	Male	10-14 years	95+ years	0	Socio-demographic Index
Self-harm	Male	10-14 years	95+ years	-1	Healthcare access and quality index
Self-harm by firearm	Female	10-14 years	95+ years	1	Alcohol (liters per capita)
Self-harm by firearm	Female	10-14 years	95+ years	0	Education (years per capita)
Self-harm by firearm	Female	10-14 years	95+ years	0	LDI (I$ per capita)
Self-harm by firearm	Female	10-14 years	95+ years	0	Population Density (150-300 ppl/sqkm, proportion)
Self-harm by firearm	Female	10-14 years	95+ years	0	Population Density (300-500 ppl/sqkm, proportion)
Self-harm by firearm	Female	10-14 years	95+ years	0	Population Density (500-1000 ppl/sqkm, proportion)
Self-harm by firearm	Female	10-14 years	95+ years	0	Population Density (over 1000 ppl/sqkm, proportion)
Self-harm by firearm	Female	10-14 years	95+ years	0	Population Density (under 150 ppl/sqkm, proportion)
Self-harm by firearm	Female	10-14 years	95+ years	-1	Religion (binary, >50% Muslim)
Self-harm by firearm	Female	10-14 years	95+ years	1	Log-transformed SEV scalar: Self Harm
Self-harm by firearm	Female	10-14 years	95+ years	0	Socio-demographic Index
Self-harm by firearm	Female	10-14 years	95+ years	1	Major depressive disorder
Self-harm by firearm	Female	10-14 years	95+ years	-1	Healthcare access and quality index
Self-harm by firearm	Male	10-14 years	95+ years	1	Alcohol (liters per capita)
Self-harm by firearm	Male	10-14 years	95+ years	0	Education (years per capita)
Self-harm by firearm	Male	10-14 years	95+ years	0	LDI (I$ per capita)
Self-harm by firearm	Male	10-14 years	95+ years	0	Population Density (150-300 ppl/sqkm, proportion)
Self-harm by firearm	Male	10-14 years	95+ years	0	Population Density (300-500 ppl/sqkm, proportion)
Self-harm by firearm	Male	10-14 years	95+ years	0	Population Density (500-1000 ppl/sqkm, proportion)
Self-harm by firearm	Male	10-14 years	95+ years	0	Population Density (over 1000 ppl/sqkm, proportion)
Self-harm by firearm	Male	10-14 years	95+ years	0	Population Density (under 150 ppl/sqkm, proportion)
Self-harm by firearm	Male	10-14 years	95+ years	-1	Religion (binary, >50% Muslim)
Self-harm by firearm	Male	10-14 years	95+ years	1	Log-transformed SEV scalar: Self Harm
Self-harm by firearm	Male	10-14 years	95+ years	0	Socio-demographic Index
Self-harm by firearm	Male	10-14 years	95+ years	1	Major depressive disorder
Self-harm by firearm	Male	10-14 years	95+ years	-1	Healthcare access and quality index
Self-harm by other specified means	Female	10-14 years	95+ years	1	Alcohol (liters per capita)
Self-harm by other specified means	Female	10-14 years	95+ years	0	Education (years per capita)
Self-harm by other specified means	Female	10-14 years	95+ years	0	LDI (I$ per capita)
Self-harm by other specified means	Female	10-14 years	95+ years	0	Population Density (150-300 ppl/sqkm, proportion)
Self-harm by other specified means	Female	10-14 years	95+ years	0	Population Density (300-500 ppl/sqkm, proportion)
Self-harm by other specified means	Female	10-14 years	95+ years	0	Population Density (500-1000 ppl/sqkm, proportion)
Self-harm by other specified means	Female	10-14 years	95+ years	0	Population Density (over 1000 ppl/sqkm, proportion)
Self-harm by other specified means	Female	10-14 years	95+ years	0	Population Density (under 150 ppl/sqkm, proportion)
Self-harm by other specified means	Female	10-14 years	95+ years	-1	Religion (binary, >50% Muslim)
Self-harm by other specified means	Female	10-14 years	95+ years	1	Log-transformed SEV scalar: Self Harm
Self-harm by other specified means	Female	10-14 years	95+ years	0	Socio-demographic Index
Self-harm by other specified means	Female	10-14 years	95+ years	1	Major depressive disorder
Self-harm by other specified means	Female	10-14 years	95+ years	-1	Healthcare access and quality index
Self-harm by other specified means	Male	10-14 years	95+ years	1	Alcohol (liters per capita)
Self-harm by other specified means	Male	10-14 years	95+ years	0	Education (years per capita)
Self-harm by other specified means	Male	10-14 years	95+ years	0	LDI (I$ per capita)
Self-harm by other specified means	Male	10-14 years	95+ years	0	Population Density (150-300 ppl/sqkm, proportion)
Self-harm by other specified means	Male	10-14 years	95+ years	0	Population Density (300-500 ppl/sqkm, proportion)
Self-harm by other specified means	Male	10-14 years	95+ years	0	Population Density (500-1000 ppl/sqkm, proportion)
Self-harm by other specified means	Male	10-14 years	95+ years	0	Population Density (over 1000 ppl/sqkm, proportion)
Self-harm by other specified means	Male	10-14 years	95+ years	0	Population Density (under 150 ppl/sqkm, proportion)
Self-harm by other specified means	Male	10-14 years	95+ years	-1	Religion (binary, >50% Muslim)
Self-harm by other specified means	Male	10-14 years	95+ years	1	Log-transformed SEV scalar: Self Harm
Self-harm by other specified means	Male	10-14 years	95+ years	0	Socio-demographic Index
Self-harm by other specified means	Male	10-14 years	95+ years	1	Major depressive disorder
Self-harm by other specified means	Male	10-14 years	95+ years	-1	Healthcare access and quality index
Interpersonal violence	Female	0-6 days	95+ years	1	Alcohol (liters per capita)
Interpersonal violence	Female	0-6 days	95+ years	0	Education (years per capita)
Interpersonal violence	Female	0-6 days	95+ years	0	LDI (I$ per capita)
Interpersonal violence	Female	0-6 days	95+ years	1	Opium Cultivation (binary)
Interpersonal violence	Female	0-6 days	95+ years	1	Population Density (over 1000 ppl/sqkm, proportion)
Interpersonal violence	Female	0-6 days	95+ years	1	Log-transformed SEV scalar: Violence
Interpersonal violence	Female	0-6 days	95+ years	0	Socio-demographic Index
Interpersonal violence	Female	0-6 days	95+ years	-1	Healthcare access and quality index
Interpersonal violence	Male	0-6 days	95+ years	1	Alcohol (liters per capita)
Interpersonal violence	Male	0-6 days	95+ years	0	Education (years per capita)
Interpersonal violence	Male	0-6 days	95+ years	0	LDI (I$ per capita)
Interpersonal violence	Male	0-6 days	95+ years	1	Opium Cultivation (binary)
Interpersonal violence	Male	0-6 days	95+ years	1	Population Density (over 1000 ppl/sqkm, proportion)
Interpersonal violence	Male	0-6 days	95+ years	1	Log-transformed SEV scalar: Violence
Interpersonal violence	Male	0-6 days	95+ years	0	Socio-demographic Index
Interpersonal violence	Male	0-6 days	95+ years	-1	Healthcare access and quality index
Physical violence by firearm	Female	0-6 days	95+ years	1	Alcohol (liters per capita)
Physical violence by firearm	Female	0-6 days	95+ years	0	Education (years per capita)
Physical violence by firearm	Female	0-6 days	95+ years	0	LDI (I$ per capita)

Cause	Sex	Age start	Age end	Direction	Covariate
Physical violence by firearm	Female	0-6 days	95+ years	1	Opium Cultivation (binary)
Physical violence by firearm	Female	0-6 days	95+ years	1	Population Density (over 1000 ppl/sqkm, proportion)
Physical violence by firearm	Female	0-6 days	95+ years	1	Log-transformed SEV scalar: Viol Gun
Physical violence by firearm	Female	0-6 days	95+ years	0	Socio-demographic Index
Physical violence by firearm	Female	0-6 days	95+ years	-1	Healthcare access and quality index
Physical violence by firearm	Male	0-6 days	95+ years	1	Alcohol (liters per capita)
Physical violence by firearm	Male	0-6 days	95+ years	0	Education (years per capita)
Physical violence by firearm	Male	0-6 days	95+ years	0	LDI (I$ per capita)
Physical violence by firearm	Male	0-6 days	95+ years	1	Opium Cultivation (binary)
Physical violence by firearm	Male	0-6 days	95+ years	1	Population Density (over 1000 ppl/sqkm, proportion)
Physical violence by firearm	Male	0-6 days	95+ years	1	Log-transformed SEV scalar: Viol Gun
Physical violence by firearm	Male	0-6 days	95+ years	0	Socio-demographic Index
Physical violence by firearm	Male	0-6 days	95+ years	-1	Healthcare access and quality index
Physical violence by sharp object	Female	0-6 days	95+ years	1	Alcohol (liters per capita)
Physical violence by sharp object	Female	0-6 days	95+ years	0	Education (years per capita)
Physical violence by sharp object	Female	0-6 days	95+ years	0	LDI (I$ per capita)
Physical violence by sharp object	Female	0-6 days	95+ years	1	Opium Cultivation (binary)
Physical violence by sharp object	Female	0-6 days	95+ years	1	Population Density (over 1000 ppl/sqkm, proportion)
Physical violence by sharp object	Female	0-6 days	95+ years	1	Log-transformed SEV scalar: Viol Knife
Physical violence by sharp object	Female	0-6 days	95+ years	0	Socio-demographic Index
Physical violence by sharp object	Female	0-6 days	95+ years	-1	Healthcare access and quality index
Physical violence by sharp object	Male	0-6 days	95+ years	1	Alcohol (liters per capita)
Physical violence by sharp object	Male	0-6 days	95+ years	0	Education (years per capita)
Physical violence by sharp object	Male	0-6 days	95+ years	0	LDI (I$ per capita)
Physical violence by sharp object	Male	0-6 days	95+ years	1	Opium Cultivation (binary)
Physical violence by sharp object	Male	0-6 days	95+ years	1	Population Density (over 1000 ppl/sqkm, proportion)
Physical violence by sharp object	Male	0-6 days	95+ years	1	Log-transformed SEV scalar: Viol Knife
Physical violence by sharp object	Male	0-6 days	95+ years	0	Socio-demographic Index
Physical violence by sharp object	Male	0-6 days	95+ years	-1	Healthcare access and quality index
Physical violence by other means	Female	0-6 days	95+ years	1	Alcohol (liters per capita)
Physical violence by other means	Female	0-6 days	95+ years	0	Education (years per capita)
Physical violence by other means	Female	0-6 days	95+ years	0	LDI (I$ per capita)
Physical violence by other means	Female	0-6 days	95+ years	1	Opium Cultivation (binary)
Physical violence by other means	Female	0-6 days	95+ years	1	Population Density (over 1000 ppl/sqkm, proportion)
Physical violence by other means	Female	0-6 days	95+ years	1	Log-transformed SEV scalar: Oth Viol
Physical violence by other means	Female	0-6 days	95+ years	0	Socio-demographic Index
Physical violence by other means	Female	0-6 days	95+ years	-1	Healthcare access and quality index
Physical violence by other means	Male	0-6 days	95+ years	1	Alcohol (liters per capita)
Physical violence by other means	Male	0-6 days	95+ years	0	Education (years per capita)
Physical violence by other means	Male	0-6 days	95+ years	0	LDI (I$ per capita)
Physical violence by other means	Male	0-6 days	95+ years	1	Opium Cultivation (binary)
Physical violence by other means	Male	0-6 days	95+ years	1	Population Density (over 1000 ppl/sqkm, proportion)
Physical violence by other means	Male	0-6 days	95+ years	1	Log-transformed SEV scalar: Oth Viol
Physical violence by other means	Male	0-6 days	95+ years	0	Socio-demographic Index
Physical violence by other means	Male	0-6 days	95+ years	-1	Healthcare access and quality index
Environmental heat and cold exposure	Female	0-6 days	95+ years	-1	Education (years per capita)
Environmental heat and cold exposure	Female	0-6 days	95+ years	0	LDI (I$ per capita)
Environmental heat and cold exposure	Female	0-6 days	95+ years	0	Population-weighted mean temperature
Environmental heat and cold exposure	Female	0-6 days	95+ years	0	Elevation Over 1500m (proportion)
Environmental heat and cold exposure	Female	0-6 days	95+ years	0	Elevation 500 to 1500m (proportion)
Environmental heat and cold exposure	Female	0-6 days	95+ years	0	Population Density (150-300 ppl/sqkm, proportion)
Environmental heat and cold exposure	Female	0-6 days	95+ years	0	Rainfall (Quintiles 4-5)
Environmental heat and cold exposure	Female	0-6 days	95+ years	0	Sanitation (proportion with access)
Environmental heat and cold exposure	Female	0-6 days	95+ years	0	90th percentile climatic temperature in the given country-year.
Environmental heat and cold exposure	Female	0-6 days	95+ years	-1	Socio-demographic Index
Environmental heat and cold exposure	Female	0-6 days	95+ years	-1	Healthcare access and quality index
Environmental heat and cold exposure	Male	0-6 days	95+ years	-1	Education (years per capita)
Environmental heat and cold exposure	Male	0-6 days	95+ years	0	LDI (I$ per capita)
Environmental heat and cold exposure	Male	0-6 days	95+ years	0	Population-weighted mean temperature
Environmental heat and cold exposure	Male	0-6 days	95+ years	0	Elevation Over 1500m (proportion)
Environmental heat and cold exposure	Male	0-6 days	95+ years	0	Elevation 500 to 1500m (proportion)
Environmental heat and cold exposure	Male	0-6 days	95+ years	0	Population Density (150-300 ppl/sqkm, proportion)
Environmental heat and cold exposure	Male	0-6 days	95+ years	0	Rainfall (Quintiles 4-5)
Environmental heat and cold exposure	Male	0-6 days	95+ years	0	Sanitation (proportion with access)
Environmental heat and cold exposure	Male	0-6 days	95+ years	0	90th percentile climatic temperature in the given country-year.
Environmental heat and cold exposure	Male	0-6 days	95+ years	-1	Socio-demographic Index
Environmental heat and cold exposure	Male	0-6 days	95+ years	-1	Healthcare access and quality index
Acute lymphoid leukemia	Female	0-6 days	95+ years	1	Alcohol (liters per capita)
Acute lymphoid leukemia	Female	0-6 days	95+ years	1	Tobacco (cigarettes per capita)
Acute lymphoid leukemia	Female	0-6 days	95+ years	1	Cumulative Cigarettes (10 Years)
Acute lymphoid leukemia	Female	0-6 days	95+ years	1	Cumulative Cigarettes (15 Years)
Acute lymphoid leukemia	Female	0-6 days	95+ years	1	Cumulative Cigarettes (20 Years)
Acute lymphoid leukemia	Female	0-6 days	95+ years	1	Cumulative Cigarettes (5 Years)
Acute lymphoid leukemia	Female	0-6 days	95+ years	-1	Education (years per capita)
Acute lymphoid leukemia	Female	0-6 days	95+ years	-1	Health System Access 2 (unitless)
Acute lymphoid leukemia	Female	0-6 days	95+ years	0	LDI (I$ per capita)
Acute lymphoid leukemia	Female	0-6 days	95+ years	1	Smoking Prevalence
Acute lymphoid leukemia	Female	0-6 days	95+ years	1	Log-transformed SEV scalar: Leukemia
Acute lymphoid leukemia	Female	0-6 days	95+ years	1	Log-transformed age-standardized SEV scalar: Leukemia
Acute lymphoid leukemia	Female	0-6 days	95+ years	0	Socio-demographic Index
Acute lymphoid leukemia	Male	0-6 days	95+ years	1	Alcohol (liters per capita)
Acute lymphoid leukemia	Male	0-6 days	95+ years	1	Tobacco (cigarettes per capita)
Acute lymphoid leukemia	Male	0-6 days	95+ years	1	Cumulative Cigarettes (10 Years)
Acute lymphoid leukemia	Male	0-6 days	95+ years	1	Cumulative Cigarettes (15 Years)
Acute lymphoid leukemia	Male	0-6 days	95+ years	1	Cumulative Cigarettes (20 Years)
Acute lymphoid leukemia	Male	0-6 days	95+ years	1	Cumulative Cigarettes (5 Years)
Acute lymphoid leukemia	Male	0-6 days	95+ years	-1	Education (years per capita)
Acute lymphoid leukemia	Male	0-6 days	95+ years	-1	Health System Access 2 (unitless)
Acute lymphoid leukemia	Male	0-6 days	95+ years	0	LDI (I$ per capita)
Acute lymphoid leukemia	Male	0-6 days	95+ years	1	Smoking Prevalence
Acute lymphoid leukemia	Male	0-6 days	95+ years	1	Log-transformed SEV scalar: Leukemia
Acute lymphoid leukemia	Male	0-6 days	95+ years	1	Log-transformed age-standardized SEV scalar: Leukemia
Acute lymphoid leukemia	Male	0-6 days	95+ years	0	Socio-demographic Index
Chronic lymphoid leukemia	Male	15-19 years	95+ years	1	Alcohol (liters per capita)
Chronic lymphoid leukemia	Male	15-19 years	95+ years	1	Tobacco (cigarettes per capita)

Cause	Sex	Age start	Age end	Direction	Covariate
Chronic lymphoid leukemia	Male	15-19 years	95+ years	1	Cumulative Cigarettes (10 Years)
Chronic lymphoid leukemia	Male	15-19 years	95+ years	1	Cumulative Cigarettes (15 Years)
Chronic lymphoid leukemia	Male	15-19 years	95+ years	1	Cumulative Cigarettes (20 Years)
Chronic lymphoid leukemia	Male	15-19 years	95+ years	1	Cumulative Cigarettes (5 Years)
Chronic lymphoid leukemia	Male	15-19 years	95+ years	-1	Education (years per capita)
Chronic lymphoid leukemia	Male	15-19 years	95+ years	-1	Health System Access 2 (unitless)
Chronic lymphoid leukemia	Male	15-19 years	95+ years	0	LDI (I$ per capita)
Chronic lymphoid leukemia	Male	15-19 years	95+ years	1	Smoking Prevalence
Chronic lymphoid leukemia	Male	15-19 years	95+ years	1	Log-transformed SEV scalar: Leukemia
Chronic lymphoid leukemia	Male	15-19 years	95+ years	1	Log-transformed age-standardized SEV scalar: Leukemia
Chronic lymphoid leukemia	Male	15-19 years	95+ years	0	Socio-demographic Index
Chronic lymphoid leukemia	Female	15-19 years	95+ years	1	Alcohol (liters per capita)
Chronic lymphoid leukemia	Female	15-19 years	95+ years	1	Tobacco (cigarettes per capita)
Chronic lymphoid leukemia	Female	15-19 years	95+ years	1	Cumulative Cigarettes (10 Years)
Chronic lymphoid leukemia	Female	15-19 years	95+ years	1	Cumulative Cigarettes (15 Years)
Chronic lymphoid leukemia	Female	15-19 years	95+ years	1	Cumulative Cigarettes (20 Years)
Chronic lymphoid leukemia	Female	15-19 years	95+ years	1	Cumulative Cigarettes (5 Years)
Chronic lymphoid leukemia	Female	15-19 years	95+ years	-1	Education (years per capita)
Chronic lymphoid leukemia	Female	15-19 years	95+ years	-1	Health System Access 2 (unitless)
Chronic lymphoid leukemia	Female	15-19 years	95+ years	0	LDI (I$ per capita)
Chronic lymphoid leukemia	Female	15-19 years	95+ years	1	Smoking Prevalence
Chronic lymphoid leukemia	Female	15-19 years	95+ years	1	Log-transformed SEV scalar: Leukemia
Chronic lymphoid leukemia	Female	15-19 years	95+ years	1	Log-transformed age-standardized SEV scalar: Leukemia
Chronic lymphoid leukemia	Female	15-19 years	95+ years	0	Socio-demographic Index
Acute myeloid leukemia	Female	0-6 days	95+ years	1	Alcohol (liters per capita)
Acute myeloid leukemia	Female	0-6 days	95+ years	1	Tobacco (cigarettes per capita)
Acute myeloid leukemia	Female	0-6 days	95+ years	1	Cumulative Cigarettes (10 Years)
Acute myeloid leukemia	Female	0-6 days	95+ years	1	Cumulative Cigarettes (15 Years)
Acute myeloid leukemia	Female	0-6 days	95+ years	1	Cumulative Cigarettes (20 Years)
Acute myeloid leukemia	Female	0-6 days	95+ years	1	Cumulative Cigarettes (5 Years)
Acute myeloid leukemia	Female	0-6 days	95+ years	-1	Education (years per capita)
Acute myeloid leukemia	Female	0-6 days	95+ years	-1	Health System Access 2 (unitless)
Acute myeloid leukemia	Female	0-6 days	95+ years	0	LDI (I$ per capita)
Acute myeloid leukemia	Female	0-6 days	95+ years	1	Smoking Prevalence
Acute myeloid leukemia	Female	0-6 days	95+ years	1	Log-transformed SEV scalar: Leukemia
Acute myeloid leukemia	Female	0-6 days	95+ years	1	Log-transformed age-standardized SEV scalar: Leukemia
Acute myeloid leukemia	Female	0-6 days	95+ years	0	Socio-demographic Index
Acute myeloid leukemia	Male	0-6 days	95+ years	1	Alcohol (liters per capita)
Acute myeloid leukemia	Male	0-6 days	95+ years	1	Tobacco (cigarettes per capita)
Acute myeloid leukemia	Male	0-6 days	95+ years	1	Cumulative Cigarettes (10 Years)
Acute myeloid leukemia	Male	0-6 days	95+ years	1	Cumulative Cigarettes (15 Years)
Acute myeloid leukemia	Male	0-6 days	95+ years	1	Cumulative Cigarettes (20 Years)
Acute myeloid leukemia	Male	0-6 days	95+ years	1	Cumulative Cigarettes (5 Years)
Acute myeloid leukemia	Male	0-6 days	95+ years	-1	Education (years per capita)
Acute myeloid leukemia	Male	0-6 days	95+ years	-1	Health System Access 2 (unitless)
Acute myeloid leukemia	Male	0-6 days	95+ years	0	LDI (I$ per capita)
Acute myeloid leukemia	Male	0-6 days	95+ years	1	Smoking Prevalence
Acute myeloid leukemia	Male	0-6 days	95+ years	1	Log-transformed SEV scalar: Leukemia
Acute myeloid leukemia	Male	0-6 days	95+ years	1	Log-transformed age-standardized SEV scalar: Leukemia
Acute myeloid leukemia	Male	0-6 days	95+ years	0	Socio-demographic Index
Acute myeloid leukemia	Male	0-6 days	95+ years	-1	Health System Access (capped)
Acute myeloid leukemia	Male	0-6 days	95+ years	-1	Healthcare access and quality index
Chronic myeloid leukemia	Female	28-364 days	95+ years	1	Alcohol (liters per capita)
Chronic myeloid leukemia	Female	28-364 days	95+ years	1	Tobacco (cigarettes per capita)
Chronic myeloid leukemia	Female	28-364 days	95+ years	1	Cumulative Cigarettes (10 Years)
Chronic myeloid leukemia	Female	28-364 days	95+ years	1	Cumulative Cigarettes (15 Years)
Chronic myeloid leukemia	Female	28-364 days	95+ years	1	Cumulative Cigarettes (20 Years)
Chronic myeloid leukemia	Female	28-364 days	95+ years	1	Cumulative Cigarettes (5 Years)
Chronic myeloid leukemia	Female	28-364 days	95+ years	-1	Education (years per capita)
Chronic myeloid leukemia	Female	28-364 days	95+ years	1	Health System Access 2 (unitless)
Chronic myeloid leukemia	Female	28-364 days	95+ years	0	LDI (I$ per capita)
Chronic myeloid leukemia	Female	28-364 days	95+ years	1	Smoking Prevalence
Chronic myeloid leukemia	Female	28-364 days	95+ years	1	Log-transformed age-standardized SEV scalar: Leukemia
Chronic myeloid leukemia	Female	28-364 days	95+ years	0	Socio-demographic Index
Chronic myeloid leukemia	Female	28-364 days	95+ years	-1	Healthcare access and quality index
Chronic myeloid leukemia	Female	28-364 days	95+ years	-1	Health System Access 2 (unitless)
Chronic myeloid leukemia	Male	28-364 days	95+ years	1	Alcohol (liters per capita)
Chronic myeloid leukemia	Male	28-364 days	95+ years	1	Tobacco (cigarettes per capita)
Chronic myeloid leukemia	Male	28-364 days	95+ years	1	Cumulative Cigarettes (10 Years)
Chronic myeloid leukemia	Male	28-364 days	95+ years	1	Cumulative Cigarettes (15 Years)
Chronic myeloid leukemia	Male	28-364 days	95+ years	1	Cumulative Cigarettes (20 Years)
Chronic myeloid leukemia	Male	28-364 days	95+ years	1	Cumulative Cigarettes (5 Years)
Chronic myeloid leukemia	Male	28-364 days	95+ years	-1	Education (years per capita)
Chronic myeloid leukemia	Male	28-364 days	95+ years	1	Health System Access 2 (unitless)
Chronic myeloid leukemia	Male	28-364 days	95+ years	0	LDI (I$ per capita)
Chronic myeloid leukemia	Male	28-364 days	95+ years	1	Smoking Prevalence
Chronic myeloid leukemia	Male	28-364 days	95+ years	1	Log-transformed age-standardized SEV scalar: Leukemia
Chronic myeloid leukemia	Male	28-364 days	95+ years	0	Socio-demographic Index
Chronic myeloid leukemia	Male	28-364 days	95+ years	-1	Healthcare access and quality index
Non-melanoma skin cancer (squamous-cell carcinoma)	Male	28-364 days	95+ years	1	Cumulative Cigarettes (10 Years)
Non-melanoma skin cancer (squamous-cell carcinoma)	Male	28-364 days	95+ years	1	Cumulative Cigarettes (15 Years)
Non-melanoma skin cancer (squamous-cell carcinoma)	Male	28-364 days	95+ years	1	Cumulative Cigarettes (5 Years)
Non-melanoma skin cancer (squamous-cell carcinoma)	Male	28-364 days	95+ years	-1	Education (years per capita)
Non-melanoma skin cancer (squamous-cell carcinoma)	Male	28-364 days	95+ years	0	Average latitude
Non-melanoma skin cancer (squamous-cell carcinoma)	Male	28-364 days	95+ years	0	LDI (I$ per capita)
Non-melanoma skin cancer (squamous-cell carcinoma)	Male	28-364 days	95+ years	1	Smoking Prevalence
Non-melanoma skin cancer (squamous-cell carcinoma)	Male	28-364 days	95+ years	0	Socio-demographic Index
Non-melanoma skin cancer (squamous-cell carcinoma)	Male	28-364 days	95+ years	-1	Healthcare access and quality index
Non-melanoma skin cancer (squamous-cell carcinoma)	Male	28-364 days	95+ years	-1	Health System Access (capped)
Non-melanoma skin cancer (squamous-cell carcinoma)	Female	28-364 days	95+ years	1	Cumulative Cigarettes (10 Years)
Non-melanoma skin cancer (squamous-cell carcinoma)	Female	28-364 days	95+ years	1	Cumulative Cigarettes (15 Years)
Non-melanoma skin cancer (squamous-cell carcinoma)	Female	28-364 days	95+ years	1	Cumulative Cigarettes (5 Years)
Non-melanoma skin cancer (squamous-cell carcinoma)	Female	28-364 days	95+ years	-1	Education (years per capita)
Non-melanoma skin cancer (squamous-cell carcinoma)	Female	28-364 days	95+ years	0	Average latitude
Non-melanoma skin cancer (squamous-cell carcinoma)	Female	28-364 days	95+ years	0	LDI (I$ per capita)

Cause	Sex	Age start	Age end	Direction	Covariate
Non-melanoma skin cancer (squamous-cell carcinoma)	Female	28-364 days	95+ years	1	Smoking Prevalence
Non-melanoma skin cancer (squamous-cell carcinoma)	Female	28-364 days	95+ years	0	Socio-demographic Index
Non-melanoma skin cancer (squamous-cell carcinoma)	Female	28-364 days	95+ years	-1	Healthcare access and quality index
Non-melanoma skin cancer (squamous-cell carcinoma)	Female	28-364 days	95+ years	-1	Health System Access (capped)
Executions and police conflict	Female	28-364 days	95+ years	1	Alcohol (liters per capita)
Executions and police conflict	Female	28-364 days	95+ years	1	Education (years per capita)
Executions and police conflict	Female	28-364 days	95+ years	0	LDI (I$ per capita)
Executions and police conflict	Female	28-364 days	95+ years	1	Population Density (over 1000 ppl/sqkm, proportion)
Executions and police conflict	Female	28-364 days	95+ years	0	Socio-demographic Index
Executions and police conflict	Female	28-364 days	95+ years	-1	Healthcare access and quality index
Executions and police conflict	Male	28-364 days	95+ years	1	Alcohol (liters per capita)
Executions and police conflict	Male	28-364 days	95+ years	1	Education (years per capita)
Executions and police conflict	Male	28-364 days	95+ years	0	LDI (I$ per capita)
Executions and police conflict	Male	28-364 days	95+ years	1	Population Density (over 1000 ppl/sqkm, proportion)
Executions and police conflict	Male	28-364 days	95+ years	0	Socio-demographic Index
Executions and police conflict	Male	28-364 days	95+ years	-1	Healthcare access and quality index
Alcoholic cardiomyopathy	Male	15-19 years	95+ years	1	Alcohol (liters per capita)
Alcoholic cardiomyopathy	Male	15-19 years	95+ years	-1	LDI (I$ per capita)
Alcoholic cardiomyopathy	Male	15-19 years	95+ years	1	Smoking Prevalence
Alcoholic cardiomyopathy	Male	15-19 years	95+ years	1	Log-transformed SEV scalar: CMP
Alcoholic cardiomyopathy	Male	15-19 years	95+ years	0	Socio-demographic Index
Alcoholic cardiomyopathy	Male	15-19 years	95+ years	-1	Healthcare access and quality index
Alcoholic cardiomyopathy	Female	15-19 years	95+ years	1	Alcohol (liters per capita)
Alcoholic cardiomyopathy	Female	15-19 years	95+ years	-1	LDI (I$ per capita)
Alcoholic cardiomyopathy	Female	15-19 years	95+ years	1	Smoking Prevalence
Alcoholic cardiomyopathy	Female	15-19 years	95+ years	1	Log-transformed SEV scalar: CMP
Alcoholic cardiomyopathy	Female	15-19 years	95+ years	0	Socio-demographic Index
Alcoholic cardiomyopathy	Female	15-19 years	95+ years	-1	Healthcare access and quality index
Myocarditis	Male	0-6 days	95+ years	-1	LDI (I$ per capita)
Myocarditis	Male	0-6 days	95+ years	1	Systolic Blood Pressure (mmHg)
Myocarditis	Male	0-6 days	95+ years	1	Log-transformed SEV scalar: CMP
Myocarditis	Male	0-6 days	95+ years	0	Socio-demographic Index
Myocarditis	Male	0-6 days	95+ years	-1	Healthcare access and quality index
Myocarditis	Female	0-6 days	95+ years	-1	LDI (I$ per capita)
Myocarditis	Female	0-6 days	95+ years	1	Systolic Blood Pressure (mmHg)
Myocarditis	Female	0-6 days	95+ years	1	Log-transformed SEV scalar: CMP
Myocarditis	Female	0-6 days	95+ years	0	Socio-demographic Index
Myocarditis	Female	0-6 days	95+ years	-1	Healthcare access and quality index
Other leukemia	Female	0-6 days	95+ years	1	Alcohol (liters per capita)
Other leukemia	Female	0-6 days	95+ years	1	Tobacco (cigarettes per capita)
Other leukemia	Female	0-6 days	95+ years	1	Cumulative Cigarettes (10 Years)
Other leukemia	Female	0-6 days	95+ years	1	Cumulative Cigarettes (15 Years)
Other leukemia	Female	0-6 days	95+ years	1	Cumulative Cigarettes (20 Years)
Other leukemia	Female	0-6 days	95+ years	1	Cumulative Cigarettes (5 Years)
Other leukemia	Female	0-6 days	95+ years	-1	Education (years per capita)
Other leukemia	Female	0-6 days	95+ years	-1	Health System Access 2 (unitless)
Other leukemia	Female	0-6 days	95+ years	0	LDI (I$ per capita)
Other leukemia	Female	0-6 days	95+ years	1	Smoking Prevalence
Other leukemia	Female	0-6 days	95+ years	1	Log-transformed SEV scalar: Leukemia
Other leukemia	Female	0-6 days	95+ years	0	Socio-demographic Index
Other leukemia	Male	0-6 days	95+ years	1	Alcohol (liters per capita)
Other leukemia	Male	0-6 days	95+ years	1	Tobacco (cigarettes per capita)
Other leukemia	Male	0-6 days	95+ years	1	Cumulative Cigarettes (10 Years)
Other leukemia	Male	0-6 days	95+ years	1	Cumulative Cigarettes (15 Years)
Other leukemia	Male	0-6 days	95+ years	1	Cumulative Cigarettes (20 Years)
Other leukemia	Male	0-6 days	95+ years	1	Cumulative Cigarettes (5 Years)
Other leukemia	Male	0-6 days	95+ years	-1	Education (years per capita)
Other leukemia	Male	0-6 days	95+ years	-1	Health System Access 2 (unitless)
Other leukemia	Male	0-6 days	95+ years	0	LDI (I$ per capita)
Other leukemia	Male	0-6 days	95+ years	1	Smoking Prevalence
Other leukemia	Male	0-6 days	95+ years	1	Log-transformed SEV scalar: Leukemia
Other leukemia	Male	0-6 days	95+ years	0	Socio-demographic Index
Other cardiomyopathy	Male	0-6 days	95+ years	0	LDI (I$ per capita)
Other cardiomyopathy	Male	0-6 days	95+ years	1	Mean BMI
Other cardiomyopathy	Male	0-6 days	95+ years	1	Systolic Blood Pressure (mmHg)
Other cardiomyopathy	Male	0-6 days	95+ years	1	Smoking Prevalence
Other cardiomyopathy	Male	0-6 days	95+ years	1	Log-transformed SEV scalar: CMP
Other cardiomyopathy	Male	0-6 days	95+ years	0	Socio-demographic Index
Other cardiomyopathy	Male	0-6 days	95+ years	-1	Healthcare access and quality index
Other cardiomyopathy	Female	0-6 days	95+ years	0	LDI (I$ per capita)
Other cardiomyopathy	Female	0-6 days	95+ years	1	Mean BMI
Other cardiomyopathy	Female	0-6 days	95+ years	1	Systolic Blood Pressure (mmHg)
Other cardiomyopathy	Female	0-6 days	95+ years	1	Smoking Prevalence
Other cardiomyopathy	Female	0-6 days	95+ years	1	Log-transformed SEV scalar: CMP
Other cardiomyopathy	Female	0-6 days	95+ years	0	Socio-demographic Index
Other cardiomyopathy	Female	0-6 days	95+ years	-1	Healthcare access and quality index

Note: Only causes modeled in CODEm are included in this table.

Appendix Table 2. Data used in Kenya mortality estimation, GBD 2016

Adaptive Integrated Malaria Vector Management at Malindi, Kenya--Final Report (May 2005-April 2006) as it appears in Malaria Atlas Project. Malaria Atlas Project Plasmodium Falciparum Parasite Rate Database. Oxford, United Kingdom: Malaria Atlas Project

African Population and Health Research Center, INDEPTH. Kenya - Nairobi Urban Health and Demographic Surveillance System

Akhwale WS, Lum JK, Kaneko A, Eto H, Obonyo C, Björkman A, Kobayakawa T. Anemia and malaria at different altitudes in the western highlands of Kenya. Addict Behav Rep. 2004; 91(2): 167-75

Amollo DA, Kihara JH, Kombe Y, Karanja SM. PREVALENCE AND INTENSITY OF SINGLE AND MIXED SCHISTOSOMA MANSONI AND SCHISTOSOMA HAEMATOBIUM INFECTIONS IN PRIMARY SCHOOL CHILDREN IN RACHUONYO NORTH DISTRICT, HOMABAY COUNTY, WESTERN KENYA. East Afr Med J. 2013; 90(2): 36–44

Annual Report for Dertu, Kenya, Millennium Village. Year 1: February 2006-February 2007 as it appears in Malaria Atlas Project. Malaria Atlas Project Plasmodium Falciparum Parasite Rate Database. Oxford, United Kingdom: Malaria Atlas Project

Armah GE, Sow SO, Breiman RF, Dallas MJ, Tapia MD, Feikin DR, Binka FN, Steele AD, Laserson KF, Ansah NA, Levine MM, Lewis K, Coia ML, Attah-Poku M, Ojwando J, Rivers SB, Victor JC, Nyambane G, Hodgson A, Schödel F, Ciarlet M, Neuzil KM. Efficacy of pentavalent rotavirus vaccine against severe rotavirus gastroenteritis in infants in developing countries in sub-Saharan Africa: a randomised, double-blind, placebo-controlled trial. Lancet. 2010; 376(9741): 606–14

Ashford R, Craig P, Oppenheimer S. Polyparasitism on the Kenya coast. 2. Spatial heterogeneity in parasite distributions. Ann Trop Med Parasitol. 1993; 87(3): 283-93 as it appears in Malaria Atlas Project. Malaria Atlas Project Plasmodium Falciparum Parasite Rate Database. Oxford, United Kingdom: Malaria Atlas Project

Ashford RW, Craig PS, Oppenheimer SJ. Polyparasitism on the Kenya coast. 1. Prevalence, and association between parasitic infections. Ann Trop Med Parasitol. 1992; 86(6): 671-9 as it appears in Malaria Atlas Project. Malaria Atlas Project Plasmodium Falciparum Parasite Rate Database. Oxford, United Kingdom: Malaria Atlas Project

Baliraine FN, Afrane YA, Amenya DA, Bonizzoni M, Menge DM, Zhou G, Zhong D, Vardo-Zalik AM, Githeko AK, Yan G. High Prevalence of Asymptomatic Plasmodium falciparum Infections in a Highland Area of Western Kenya: A Cohort Study. J Infect Dis. 2009; 200(1): 66-74 as it appears in Malaria Atlas Project. Malaria Atlas Project Plasmodium Falciparum Parasite Rate Database. Oxford, United Kingdom: Malaria Atlas Project

Baseline Report: Millenium Research Village Sauri, Kenya as it appears in Malaria Atlas Project. Malaria Atlas Project Plasmodium Falciparum Parasite Rate Database. Oxford, United Kingdom: Malaria Atlas Project

Beatty ME, Ochieng JB, Chege W, Kumar L, Okoth G, Shapiro RL, Wells JG, Parsons MB, Bopp C, Chiller T, Vulule J, Mintz E, Slutsker L, Brooks JT. Sporadic paediatric diarrhoeal illness in urban and rural sites in Nyanza Province, Kenya. East Afr Med J. 2009; 86(8): 387-98

Beier J, Oster C, Onyango F, Bales J, Sherwood J, Perkins P, Chumo D, Koech dv, Whitmire R, Roberts C. Plasmodium falciparum incidence relative to entomologic inoculation rates at a site proposed for testing malaria vaccines in western Kenya. Am J Trop Med Hyg. 1994; 50(5): 529-36 as it appears in Malaria Atlas Project. Malaria Atlas Project Plasmodium Falciparum Parasite Rate Database. Oxford, United Kingdom: Malaria Atlas Project

Bejon P, Lusingu J, Olotu A, Leach A, Lievens M, Vekemans J, Mshamu S, Lang T, Gould J, Dubois MC, Demoitié MA, Stallaert JF, Vansadia P, Carter T, Njuguna P, Awuondo KO, Malabeja A, Abdul

O, Gesase S, Mturi N, Drakeley CJ, Savarese B, Villafana T, Ballou WR, Cohen J, Riley EM, Lemnge MM, Marsh K, von Seidlein L. Efficacy of RTS,S/AS01E Vaccine against Malaria in Children 5 to 17 Months of Age. N Engl J Med. 2008; 359(24): 2521-32 as it appears in Malaria Atlas Project. Malaria Atlas Project Plasmodium Falciparum Parasite Rate Database. Oxford, United Kingdom: Malaria Atlas Project

Bejon P, Mwacharo J, Kai O, Mwangi T, Milligan P, Todryk S, Keating S, Lang T, Lowe B, Gikonyo C, Molyneux C, Fegan G, Gilbert SC, Peshu N, Marsh K, Hill AVS. A Phase 2b Randomised Trial of the Candidate Malaria Vaccines FP9 ME-TRAP and MVA ME-TRAP among Children in Kenya. PLoS Clin Trials. 2006; 1(6): e29 as it appears in Malaria Atlas Project. Malaria Atlas Project Plasmodium Falciparum Parasite Rate Database. Oxford, United Kingdom: Malaria Atlas Project

Berkley JA, Munywoki P, Ngama M, Kazungu S, Abwao J, Bett A, Lassauniére R, Kresfelder T, Cane PA, Venter M, Scott JAG, Nokes DJ. Viral etiology of severe pneumonia among Kenyan infants and children. JAMA. 2010; 303(20): 2051-7

Bloland PB, Boriga DA, Ruebush TK, McCormick JB, Roberts JM, Oloo AJ, Hawley W, Lal A, Nahlen B, Campbell CC. Longitudinal cohort study of the epidemiology of malaria infections in an area of intense malaria transmission II. Descriptive epidemiology of malaria infection and disease among children. Am J Trop Med Hyg. 1999; 60(4): 641-8 as it appears in Malaria Atlas Project. Malaria Atlas Project Plasmodium Falciparum Parasite Rate Database. Oxford, United Kingdom: Malaria Atlas Project

Bonizzoni M, Afrane Y, Baliraine FN, Amenya DA, Githeko AK, Yan G. Genetic structure of Plasmodium falciparum populations between lowland and highland sites and antimalarial drug resistance in Western Kenya. Infect Genet Evol. 2009; 9(5): 806-12 as it appears in Malaria Atlas Project. Malaria Atlas Project Plasmodium Falciparum Parasite Rate Database. Oxford, United Kingdom: Malaria Atlas Project

Bousema JT, Gouagna LC, Drakeley CJ, Meutstege AM, Okech BA, Akim IN, Beier JC, Githure JI, Sauerwein RW. Plasmodium falciparum gametocyte carriage in asymptomatic children in western Kenya. Malar J. 2004; 3: 18 as it appears in Malaria Atlas Project. Malaria Atlas Project Plasmodium Falciparum Parasite Rate Database. Oxford, United Kingdom: Malaria Atlas Project

Bowry TR, Pade J, Omari M, Chemtai A. A pilot study of hepatitis B virus seroepidemiology suggests widespread immunosuppression in the nomadic inhabitants of Turkana District of Kenya. East Afr Med J. 1985; 62(7): 501-6

Breiman RF, Cosmas L, Audi A, Mwiti W, Njuguna H, Bigogo GM, Olack B, Ochieng JB, Wamola N, Montgomery JM, Williamson J, Parashar UD, Burton DC, Tate JE, Feikin DR. Use of population-based surveillance to determine the incidence of rotavirus gastroenteritis in an urban slum and a rural setting in Kenya. Pediatr Infect Dis J. 2014; S54-61

Breiman RF, Cosmas L, Njenga M, Williamson J, Mott JA, Katz MA, Erdman DD, Schneider E, Oberste M, Neatherlin JC, Njuguna H, Ondari DM, Odero K, Okoth GO, Olack B, Wamola N, Montgomery JM, Fields BS, Feikin DR. Severe acute respiratory infection in children in a densely populated urban slum in Kenya, 2007-2011. BMC Infect Dis. 2015; 15: 95

Breiman RF, Cosmas L, Njuguna H, Audi A, Olack B, Ochieng JB, Wamola N, Bigogo GM, Awiti G, Tabu CW, Burke H, Williamson J, Oundo JO, Mintz ED, Feikin DR. Population-based incidence of typhoid fever in an urban informal settlement and a rural area in Kenya: implications for typhoid vaccine use in Africa. PLoS One. 2012; 7(1): e29119

Brinkhof MWG, Dabis F, Myer L, Bangsberg DR, Boulle A, Nash D, Schechter M, Laurent C, Keiser O, May M, Sprinz E, Egger M, Anglaret X, ART-LINC, IeDEA. Early loss of HIV-infected patients on

potent antiretroviral therapy programmes in lower-income countries. Bull World Health Organ. 2008; 86(7): 559-67
Brooker S, Miguel EA, Moulin S, Luoba AI, Bundy DAP, Kremer M. Epidemiology of single and multiple species of helminth infections among school children in Busia District, Kenya. East Afr Med J. 2000; 77(3): 157-61
Brooker SJ, Pullan RL, Gitonga CW, Ashton RA, Kolaczinski JH, Kabatereine NB, Snow RW. Plasmodium-Helminth Coinfection and Its Sources of Heterogeneity Across East Africa. J Infect Dis. 2012; 205(5): 841-52
Burström B, Aaby P, Mutie DM, Kimani G, Bjerregaard P. Severe measles outbreak in western Kenya. East Afr Med J. 1992; 69(8): 419-23
Burström B, Aaby P, Mutie DM. Child mortality impact of a measles outbreak in a partially vaccinated rural African community. Scand J Infect Dis. 1993; 25(6): 763-9
Burström B, Aaby P, Mutie DM. Validity of measles mortality data using hospital registers and community surveys. Int J Epidemiol. 1995; 24(3): 625-9
Bustinduy AL, Parraga IM, Thomas CL, Mungai PL, Mutuku F, Muchiri EM, Kitron U, King CH. Impact of polyparasitic infections on anemia and undernutrition among Kenyan children living in a Schistosoma haematobium-endemic area. Am J Trop Med Hyg. 2013; 88(3): 433-40 as it appears in Malaria Atlas Project. Malaria Atlas Project Plasmodium Falciparum Parasite Rate Database. Oxford, United Kingdom: Malaria Atlas Project
Bustinduy AL, Sutherland LJ, Chang-Cojulun A, Malhotra I, DuVall AS, Fairley JK, Mungai PL, Muchiri EM, Mutuku FM, Kitron U, King CH. Age-Stratified Profiles of Serum IL-6, IL-10, and TNF-a Cytokines Among Kenyan Children with Schistosoma haematobium, Plasmodium falciparum, and Other Chronic Parasitic Co-Infections. Am J Trop Med Hyg. 2015; 92(5): 945-51 as it appears in Malaria Atlas Project. Malaria Atlas Project Plasmodium Falciparum Parasite Rate Database. Oxford, United Kingdom: Malaria Atlas Project
Bustinduy AL, Thomas CL, Fiutem JJ, Parraga IM, Mungai PL, Muchiri EM, Mutuku F, Kitron U, King CH. Measuring fitness of Kenyan children with polyparasitic infections using the 20-meter shuttle run test as a morbidity metric. PLoS Negl Trop Dis. 2011; 5(7): e1213
Campbell GH, Collins FH, Brandling-Bennett AD, Schwartz IK, Roberts JM. Age-specific prevalence of antibody to a synthetic peptide of the circumsporozoite protein of Plasmodium falciparum in children from three villages in Kenya. Am J Trop Med Hyg. 1987; 37(2): 220-4 as it appears in Malaria Atlas Project. Malaria Atlas Project Plasmodium Falciparum Parasite Rate Database. Oxford, United Kingdom: Malaria Atlas Project
Case definitions of clinical malaria under different transmission conditions in Kilifi District, Kenya and Clinical algorithms for malaria diagnosis lack utility among people of different age groups as it appears in Battle KE, Guerra CA, Golding N, Duda KA, Cameron E, Howes RE, Elyazar IRF, Baird JK, Reiner Jr. RC, Gething PW, Smith DL, Hay SI. Data from: Global database of matched Plasmodium falciparum and P. vivax incidence and prevalence records from 1985–2013. Dryad Digital Repository. 2015
Center for Vaccine Development (Chile), Center for Vaccine Development, University of Maryland, Centers for Disease Control and Prevention (CDC), Department of Medical Microbiology and Immunology, Göteborg University, International Vaccine Institute, Kenya Medical Research Institute (KEMRI), Perry Point Cooperative Studies Program Coordinating Center, U.S. Department of Veterans Affairs, School of Medicine, University of Virginia, University of Chile. Kenya - Nyanza Global Enteric Multicenter Study 2011-2012. Baltimore, MD, United States: Center for Vaccine Development, University of Maryland
Centers for Disease Control and Prevention (CDC), Central Bureau of Statistics (Kenya), Macro International, Inc, Ministry of Health (Kenya), National Council for Population and

Development (Kenya). Kenya Demographic and Health Survey 2003. Calverton, United States: Macro International, Inc
Centers for Disease Control and Prevention (CDC), ICF Macro, Kenya Medical Research Institute (KEMRI), Kenya National Bureau of Statistics, Ministry of Public Health and Sanitation (Kenya), National AIDS and STI Control Program (Kenya), National Aids Control Council (NACC), National Coordinating Agency for Population and Development (Kenya). Kenya Demographic and Health Survey - Maternal Mortality Data
Centers for Disease Control and Prevention (CDC), Kenya Medical Research Institute (KEMRI). Kenya KEMRI/CDC Health and Demographic Surveillance System
Central Bureau of Statistics (Kenya), Macro International, Inc, National Council for Population Development (NCPD). Kenya Demographic and Health Survey 1993. Calverton, United States: Macro International, Inc
Central Bureau of Statistics (Kenya), Macro International, Inc, National Council for Population Development (NCPD). Kenya Demographic and Health Survey 1998. Calverton, United States: Macro International, Inc
Centre for Research on the Epidemiology of Disasters (CRED). EM-DAT: The OFDA/CRED International Disaster Database. Brussels, Belgium: Catholic University of Leuven
Chunge RN, Karumba N, Ouma JH, Thiongo FW, Sturrock RF, Butterworth AE. Polyparasitism in two rural communities with endemic Schistosoma mansoni infection in Machakos District, Kenya. J Trop Med Hyg. 1995; 98(6): 440-4
Clarke SE, Brooker S, Njagi JK, Njau E, Estambale B, Muchiri E, Magnussen P. Malaria morbidity among school children living in two areas of contrasting transmission in western Kenya. Am J Trop Med Hyg. 2004; 71(6): 732-8 as it appears in Malaria Atlas Project. Malaria Atlas Project Plasmodium Falciparum Parasite Rate Database. Oxford, United Kingdom: Malaria Atlas Project
Clarke SE, Jukes MCH, Njagi JK, Khasakhala L, Cundill B, Otido J, Crudder C, Estambale BBA, Brooker S. Effect of intermittent preventive treatment of malaria on health and education in schoolchildren: a cluster-randomised, double-blind, placebo-controlled trial. Lancet. 2008; 372(9633): 127-38 as it appears in Malaria Atlas Project. Malaria Atlas Project Plasmodium Falciparum Parasite Rate Database. Oxford, United Kingdom: Malaria Atlas Project
Clennon JA, King CH, Muchiri EM, Kariuki HC, Ouma JH, Mungai P, Kitron U. Spatial patterns of urinary schistosomiasis infection in a highly endemic area of coastal Kenya. Am J Trop Med Hyg. 2004; 70(4): 443-8
Climate Change and African Political Stability Project (CCAPS). Armed Conflict Location and Event Dataset, Realtime - Robert S. Strauss Center as referenced in Raleigh, Clionadh, Andrew Linke, Havard Hegre and Joakim Karlsen. 2010. Introducing ACLED-Armed Conflict Location and Event Data. Journal of Peace Research 47(5), 651-60
Clinical Epidemiology of Malaria Under Differing Levels of Transmission as it appears in Malaria Atlas Project. Malaria Atlas Project Plasmodium Falciparum Parasite Rate Database. Oxford, United Kingdom: Malaria Atlas Project
Coles GC, Mutahi WT, Kinoti GK, Bruce JI, Katz N. Tolerance of Kenyan Schistosoma mansoni to oxamniquine. Trans R Soc Trop Med Hyg. 1987; 81(5): 782-5
Comparative Studies on Malaria Transmssion Potential at a Lakeshore and a Nearby Inland Site in Western Kenya as it appears in Malaria Atlas Project. Malaria Atlas Project Plasmodium Falciparum Parasite Rate Database. Oxford, United Kingdom: Malaria Atlas Project
Corbett EL, Butterworth AE, Fulford AJ, Ouma JH, Sturrock RF. Nutritional status of children with schistosomiasis mansoni in two different areas of Machakos District, Kenya. Trans R Soc Trop Med Hyg. 1992; 86(3): 266-73

Deloron P, Campbell GH, Brandling-Bennett D, Roberts JM, Schwartz IK, Odera JS, Lal AA, Osanga CO, de la Cruz V, McCutchan TM. Antibodies to Plasmodium falciparum ring-infected erythrocyte surface antigen and P. falciparum and P. malariae circumsporozoite proteins: seasonal prevalence in Kenyan villages. Am J Trop Med Hyg. 1989; 41(4): 395-9 as it appears in Malaria Atlas Project. Malaria Atlas Project Plasmodium Falciparum Parasite Rate Database. Oxford, United Kingdom: Malaria Atlas Project

Dent AE, Chelimo K, Sumba PO, Spring MD, Crabb BS, Moormann AM, Tisch DJ, Kazura JW. Temporal stability of naturally acquired immunity to Merozoite Surface Protein-1 in Kenyan Adults. Malar J. 2009; 8(1): 162 as it appears in Malaria Atlas Project. Malaria Atlas Project Plasmodium Falciparum Parasite Rate Database. Oxford, United Kingdom: Malaria Atlas Project

Dent AE, Moormann AM, Yohn CT, Kimmel RJ, Sumba PO, Vulule J, Long CA, Narum DL, Crabb BS, Kazura JW, Tisch DJ. Broadly reactive antibodies specific for Plasmodium falciparum MSP-1(19) are associated with the protection of naturally exposed children against infection. Malar J. 2012; 11(287) as it appears in Malaria Atlas Project. Malaria Atlas Project Plasmodium Falciparum Parasite Rate Database. Oxford, United Kingdom: Malaria Atlas Project

Department of Peace and Conflict Research, Uppsala University. UCDP/PRIO Armed Conflict Database Version 4, 2015 - UCDP. Uppsala, Sweden: Department of Peace and Conflict Research, Uppsala University, 2014

Department of Peace and Conflict Research, Uppsala University. UCDP One-Sided Violence Dataset, Version 1.4, 2015. Uppsala, Sweden: Department of Peace and Conflict Research, Uppsala University, 2015

Department of Peace and Conflict Research, Uppsala University. UCDP Georeferenced Event Dataset, Version 5.0, 2016. Uppsala, Sweden: Department of Peace and Conflict Research, Uppsala University, 2016

Department of Peace and Conflict Research, Uppsala University. UCDP Battle-Related Deaths Database, Version 5, 2016. Uppsala, Sweden: Department of Peace and Conflict Research, Uppsala University, 2015

Department of Peace and Conflict Research, Uppsala University. UCDP Nonstate Conflict Dataset, Version 2.5, 2016. Uppsala, Sweden: Department of Peace and Conflict Research, Uppsala University, 2013

Desai M, Phillips-Howard PA, Odhiambo FO, Katana A, Ouma P, Hamel MJ, Omoto J, Macharia S, van Eijk A, Ogwang S, Slutsker L, Laserson KF. An analysis of pregnancy-related mortality in the KEMRI/CDC health and demographic surveillance system in western Kenya. PLoS One. 2013; 8(7): e68733

Doenhoff MJ, Butterworth AE, Hayes RJ, Sturrock RF, Ouma JH, Koech D, Prentice M, Bain J. Seroepidemiology and serodiagnosis of schistosomiasis in Kenya using crude and purified egg antigens of Schistosoma mansoni in ELISA. Trans R Soc Trop Med Hyg. 1993; 87(1): 42-8

Dynamics of Malaria Transmission and its Epidemiology among Children Population of Kilifi District, Coast Province, Kenya as it appears in Malaria Atlas Project. Malaria Atlas Project Plasmodium Falciparum Parasite Rate Database. Oxford, United Kingdom: Malaria Atlas Project

Environmental Factors Affecting the Development of Plasmodium Falciparum in Anopheles Gambaie Mosquitoes as it appears in Malaria Atlas Project. Malaria Atlas Project Plasmodium Falciparum Parasite Rate Database. Oxford, United Kingdom: Malaria Atlas Project

Estimation of Attributable Risk of Anemia Due to Schistosomiasis in Western Kenya as it appears in Malaria Atlas Project. Malaria Atlas Project Plasmodium Falciparum Parasite Rate Database. Oxford, United Kingdom: Malaria Atlas Project

Evaluation of Malaria Control in Kisumu Municipality, Kenya: A Case Study as it appears in Malaria Atlas Project. Malaria Atlas Project Plasmodium Falciparum Parasite Rate Database. Oxford, United Kingdom: Malaria Atlas Project

Evidence of Plasmodium Species Interactions in an Endemic Population in Coastal Kenya as it appears in Malaria Atlas Project. Malaria Atlas Project Plasmodium Falciparum Parasite Rate Database. Oxford, United Kingdom: Malaria Atlas Project

Feikin DR, Njenga MK, Bigogo G, Aura B, Aol G, Audi A, Jagero G, Muluare PO, Gikunju S, Nderitu L, Balish A, Winchell J, Schneider E, Erdman D, Oberste MS, Katz MA, Breiman RF. Etiology and Incidence of viral and bacterial acute respiratory illness among older children and adults in rural western Kenya, 2007-2010. PLoS One. 2012; 7(8): e43656

Feikin DR, Njenga MK, Bigogo G, Aura B, Aol G, Audi A, Jagero G, Muluare PO, Gikunju S, Nderitu L, Winchell JM, Schneider E, Erdman DD, Oberste MS, Katz MA, Breiman RF. Viral and bacterial causes of severe acute respiratory illness among children aged less than 5 years in a high malaria prevalence area of western Kenya, 2007-2010. Pediatr Infect Dis J. 2013; 32(1): e14-9

Fillinger U, Ndenga B, Githeko A, Lindsay SW. Integrated malaria vector control with microbial larvicides and insecticide-treated nets in western Kenya: a controlled trial. Bull World Health Organ. 2009; 87(9): 655-65 as it appears in Malaria Atlas Project. Malaria Atlas Project Plasmodium Falciparum Parasite Rate Database. Oxford, United Kingdom: Malaria Atlas Project

Freeman MC, Clasen T, Brooker SJ, Akoko DO, Rheingans R. The impact of a school-based hygiene, water quality and sanitation intervention on soil-transmitted helminth reinfection: a cluster-randomized trial. Am J Trop Med Hyg. 2013; 89(5): 875-83

Friis H, Mwaniki D, Omondi B, Muniu E, Magnussen P, Geissler W, Thiong'o F, Michaelsen KF. Serum retinol concentrations and Schistosoma mansoni, intestinal helminths, and malarial parasitemia: a cross-sectional study in Kenyan preschool and primary school children. Am J Clin Nutr. 1997; 66(3): 665-71 as it appears in Malaria Atlas Project. Malaria Atlas Project Plasmodium Falciparum Parasite Rate Database. Oxford, United Kingdom: Malaria Atlas Project

Fujii Y, Kaneko S, Nzou SM, Mwau M, Njenga SM, Tanigawa C, Kimotho J, Mwangi AW, Kiche I, Matsumoto S, Niki M, Osada-Oka M, Ichinose Y, Inoue M, Itoh M, Tachibana H, Ishii K, Tsuboi T, Yoshida LM, Mondal D, Haque R, Hamano S, Changoma M, Hoshi T, Kamo K-I, Karama M, Miura M, Hirayama K. Serological surveillance development for tropical infectious diseases using simultaneous microsphere-based multiplex assays and finite mixture models.. PLoS Negl Trop Dis. 2014; 8(7): e3040

Fulford AJ, Mbugua GG, Ouma JH, Kariuki HC, Sturrock RF, Butterworth AE. Differences in the rate of hepatosplenomegaly due to Schistosoma mansoni infection between two areas in Machakos District, Kenya. Trans R Soc Trop Med Hyg. 1991; 85(4): 481-8 as it appears in Malaria Atlas Project. Malaria Atlas Project Plasmodium Falciparum Parasite Rate Database. Oxford, United Kingdom: Malaria Atlas Project

Gatei W, Wamae CN, Mbae C, Waruru A, Mulinge E, Waithera T, Gatika SM, Kamwati SK, Revathi G, Hart CA. Cryptosporidiosis: Prevalence, Genotype Analysis, and Symptoms Associated with Infections in Children in Kenya. Am J Trop Med Hyg. 2006; 75(1): 78–82

Githonga A. Oil Truck Fireball Kills at Least 42 in Central Kenya: Aid Worker. Reuters [Internet]. 2016 Dec 11. World News

Gitonga CW, Karanja PN, Kihara J, Mwanje M, Juma E, Snow RW, Noor AM, Brooker S. Implementing school malaria surveys in Kenya: towards a national surveillance system. Malar J. 2010; 9: 306 as it appears in Malaria Atlas Project. Malaria Atlas Project Plasmodium Falciparum Parasite Rate Database. Oxford, United Kingdom: Malaria Atlas Project

Gouvras AN, Kariuki C, Koukounari A, Norton A, Lange CN, Ireri E, Fenwick A, Mkoji GM, Webster JP. The impact of single versus mixed Schistosoma haematobium and S. mansoni infections on morbidity profiles amongst school-children in Taveta, Kenya. Addict Behav Rep. 2013; 128(2): 309-17

Greene JA, Moormann AM, Vulule J, Bockarie MJ, Zimmerman PA, Kazura JW. Toll-like receptor polymorphisms in malaria-endemic populations. Malar J. 2009; 8: 50 as it appears in Malaria Atlas Project. Malaria Atlas Project Plasmodium Falciparum Parasite Rate Database. Oxford, United Kingdom: Malaria Atlas Project

Greenham R. Anaemia and Schistosoma haematobium infection in the North-Eastern Province of Kenya. Trans R Soc Trop Med Hyg. 1978; 72(1): 72-5

Grimsrud A, Balkan S, Casas EC, Lujan J, Van Cutsem G, Poulet E, Myer L, Pujades-Rodriguez M. Outcomes of antiretroviral therapy over a 10-year period of expansion: a multicohort analysis of African and Asian HIV programs. J Acquir Immune Defic Syndr. 2014; 67(2): e55–66

Guyatt HL, Corlett SK, Robinson TP, Ochola SA, Snow RW. Malaria prevention in highland Kenya: indoor residual house-spraying vs. insecticide-treated bednets. Trop Med Int Health. 2002; 7(4): 298-303 as it appears in Malaria Atlas Project. Malaria Atlas Project Plasmodium Falciparum Parasite Rate Database. Oxford, United Kingdom: Malaria Atlas Project

Hagos B, Khan B, Ofulla AV, Kariuki D, Martin SK. Response of falciparum malaria to chloroquine and three second line antimalarial drugs in a Kenyan coastal school age population. East Afr Med J. 1993; 70(10): 620-3 as it appears in Malaria Atlas Project. Malaria Atlas Project Plasmodium Falciparum Parasite Rate Database. Oxford, United Kingdom: Malaria Atlas Project

Halliday KE, Karanja P, Turner EL, Okello G, Njagi K, Dubeck MM, Allen E, Jukes MCH, Brooker SJ. Plasmodium falciparum, anaemia and cognitive and educational performance among school children in an area of moderate malaria transmission: baseline results of a cluster randomized trial on the coast of Kenya. Trop Med Int Health. 2012; 17(5): 532-49 as it appears in Malaria Atlas Project. Malaria Atlas Project Plasmodium Falciparum Parasite Rate Database. Oxford, United Kingdom: Malaria Atlas Project

Hamel MJ, Adazu K, Obor D, Sewe M, Vulule J, Williamson JM, Slutsker L, Feikin DR, Laserson KF. A reversal in reductions of child mortality in western Kenya, 2003-2009. Am J Trop Med Hyg. 2011; 85(4): 597-605

Hammitt LL, Kazungu S, Morpeth SC, Gibson DG, Mvera B, Brent AJ, Mwarumba S, Onyango CO, Bett A, Akech DO, Murdoch DR, Nokes DJ, Scott JAG. A preliminary study of pneumonia etiology among hospitalized children in Kenya. Clin Infect Dis. 2012; 54(Supp 2): S190-199

Handzel T, Karanja DM, Addiss DG, Hightower AW, Rosen DH, Colley DG, Andove J, Slutsker L, Secor WE. Geographic distribution of schistosomiasis and soil-transmitted helminths in Western Kenya: implications for anthelminthic mass treatment. Am J Trop Med Hyg. 2003; 69(3): 318-23

Hodder SL, Mahmoud AA, Sorenson K, Weinert DM, Stein RL, Ouma JH, Koech D, King CH. Predisposition to urinary tract epithelial metaplasia in Schistosoma haematobium infection. Am J Trop Med Hyg. 2000; 63(3-4): 133-8

Human African Trypanosomiasis At-Risk Population Estimates 1980-2015, as provided by the Global Burden of Disease 2010 Nematode expert group. [Unpublished]

ICF International, Kenya Medical Research Institute (KEMRI), Kenya National Bureau of Statistics, Ministry of Health (Kenya), National AIDS Control Council (Kenya), National Council for Population and Development (Kenya). Kenya Demographic and Health Survey 2014. Fairfax, United States: ICF International

ICF Macro, Kenya Medical Research Institute (KEMRI), Kenya National Bureau of Statistics, Ministry of Public Health and Sanitation (Kenya), National AIDS and STI Control Program (Kenya), National Aids Control Council (NACC), National Coordinating Agency for Population and Development (Kenya). Kenya Demographic and Health Survey 2008-2009. Calverton, United States: ICF Macro

Ilako FM, McLigeyo SO, Riyat MS, Lule GN, Okoth FA, Kaptich D. The prevalence of hepatitis C virus antibodies in renal patients, blood donors and patients with chronic liver disease in Kenya. East Afr Med J. 1995; 72(6): 362-4

Imbahale SS, Fillinger U, Githeko A, Mukabana WR, Takken W. An exploratory survey of malaria prevalence and people's knowledge, attitudes and practices of mosquito larval source management for malaria control in western Kenya. Addict Behav Rep. 2010; 115(3): 248-56 as it appears in Malaria Atlas Project. Malaria Atlas Project Plasmodium Falciparum Parasite Rate Database. Oxford, United Kingdom: Malaria Atlas Project

INDEPTH. Africa, Asia, Oceania - INDEPTH Network Cause-Specific Mortality - Release 2014. Accra, Ghana: INDEPTH, 2014

Insecticide Treated Net Procurement Data, Personal Communication with the World Health Organization 2016 as it appears in Malaria Atlas Project. Malaria Atlas Project Interventions Database

International Vaccine Institute. Typhoid Fever Surveillance in Africa Program (TSAP) Typhoid & Salmonella Tabulations

Isozumi R, Uemura H, Kimata I, Ichinose Y, Logedi J, Omar AH, Kaneko A. Novel mutations in K13 propeller gene of artemisinin-resistant plasmodium falciparum.(DISPATCHES). Emerg Infect Dis. 2015; 21(3): 490–2 as it appears in Malaria Atlas Project. Malaria Atlas Project Plasmodium Falciparum Parasite Rate Database. Oxford, United Kingdom: Malaria Atlas Project

Jaoko WG, Muchemi G, Oguya FO. Praziquantel side effects during treatment of Schistosoma mansoni infected pupils in Kibwezi, Kenya. East Afr Med J. 1996; 73(8): 499-501

Johansen MV, Simonsen PE, Butterworth AE, Ouma JH, Mbugua GG, Sturrock RF, Orinda DA, Christensen NO. A survey of Schistosoma mansoni induced kidney disease in children in an endemic area of Machakos District, Kenya. Addict Behav Rep. 1994; 58(1): 21-8

John CC, McHugh MM, Moormann AM, Sumba PO, Ofulla AV. Low prevalence of Plasmodium falciparum infection among asymptomatic individuals in a highland area of Kenya. Trans R Soc Trop Med Hyg. 2005; 99(10): 780-6 as it appears in Malaria Atlas Project. Malaria Atlas Project Plasmodium Falciparum Parasite Rate Database. Oxford, United Kingdom: Malaria Atlas Project

Joint United Nations Program on HIV/AIDS (UNAIDS). UNAIDS Spectrum - National HIV Estimates 2016. Geneva, Switzerland: Joint United Nations Program on HIV/AIDS (UNAIDS)

Joint WHO/Ministry of Health Report: Malaria Training Programme and Malaria Endemicity in Kenya - Base-line Data as it appears in Malaria Atlas Project. Malaria Atlas Project Plasmodium Falciparum Parasite Rate Database. Oxford, United Kingdom: Malaria Atlas Project

Kabiru EW, Gachare JW, Mbaabu DA, Ngindu AM, Siongok TK. In-vivo falciparum malaria response to chloroquine in Kisumu-Kenya. East Afr Med J. 1987; 64(9): 606-10 as it appears in Malaria Atlas Project. Malaria Atlas Project Plasmodium Falciparum Parasite Rate Database. Oxford, United Kingdom: Malaria Atlas Project

Kakai R, Wamola IA, Bwayo JJ, Ndinya-Achola JO. Enteric pathogens in malnourished children with diarrhoea. East Afr Med J. 1995; 72(5): 288-9

Kakai R, Wamola IA, Bwayo JJ. Association of human rotavirus infection and intestinal rotavirus-specific immunoglobulin A in children with diarrhoea. East Afr Med J. 1995; 72(4): 217-9

Karcher H, Omondi A, Odera J, Kunz A, Harms G. Risk factors for treatment denial and loss to follow-up in an antiretroviral treatment cohort in Kenya. Trop Med Int Health. 2007; 12(5): 687-94

Katamine D, Arap Siongok TK, Kawashima K, Nakajima Y, Nojima H, Imai J. Prevalence of human Schistosomiasis in the Taveta area of Kenya, East Africa. Japan J Trop Med Hyg. 1978; 6(3, 4): 167-80

Kenya - Chemase Entomology and Parasitology Report July 1981 as it appears in Malaria Atlas Project. Malaria Atlas Project Plasmodium Falciparum Parasite Rate Database. Oxford, United Kingdom: Malaria Atlas Project

Kenya - Chemase Entomology and Parasitology Report June 1981 as it appears in Malaria Atlas Project. Malaria Atlas Project Plasmodium Falciparum Parasite Rate Database. Oxford, United Kingdom: Malaria Atlas Project

Kenya - Division of Vector-Borne Diseases Monthly Report September 1984 as it appears in Malaria Atlas Project. Malaria Atlas Project Plasmodium Falciparum Parasite Rate Database. Oxford, United Kingdom: Malaria Atlas Project

Kenya - Kisii Malariometric Survey, May/June 2007 as it appears in Malaria Atlas Project. Malaria Atlas Project Plasmodium Falciparum Parasite Rate Database. Oxford, United Kingdom: Malaria Atlas Project

Kenya – Mombasa District Division of Vector-Borne Diseases Activities Report 1996 as it appears in Malaria Atlas Project. Malaria Atlas Project Plasmodium Falciparum Parasite Rate Database. Oxford, United Kingdom: Malaria Atlas Project

Kenya - Nyando Integrated Child Health and Education (NICHE) Project Baseline Survey 2007 as it appears in Malaria Atlas Project. Malaria Atlas Project Plasmodium Falciparum Parasite Rate Database. Oxford, United Kingdom: Malaria Atlas Project

Kenya AIDS Indicator Survey 2007 as it appears in Malaria Atlas Project. Malaria Atlas Project Interventions Database

Kenya Division of Vector-Borne Diseases - Field Activities Report for the Financial Year 95/96 as it appears in Malaria Atlas Project. Malaria Atlas Project Plasmodium Falciparum Parasite Rate Database. Oxford, United Kingdom: Malaria Atlas Project

Kenya Division of Vector-Borne Diseases Annual and Monthly Reports for 1975-1998 as it appears in Malaria Atlas Project. Malaria Atlas Project Plasmodium Falciparum Parasite Rate Database. Oxford, United Kingdom: Malaria Atlas Project

Kenya Division of Vector-Borne Diseases Annual Report 1979 as it appears in Malaria Atlas Project. Malaria Atlas Project Plasmodium Falciparum Parasite Rate Database. Oxford, United Kingdom: Malaria Atlas Project

Kenya Division of Vector-Borne Diseases Annual Report 1999 as it appears in Malaria Atlas Project. Malaria Atlas Project Plasmodium Falciparum Parasite Rate Database. Oxford, United Kingdom: Malaria Atlas Project

Kenya Division of Vector-Borne Diseases Malariometric Report 1987-1996 as it appears in Malaria Atlas Project. Malaria Atlas Project Plasmodium Falciparum Parasite Rate Database. Oxford, United Kingdom: Malaria Atlas Project

Kenya Division of Vector-Borne Diseases Monthly Report April - June 1996 as it appears in Malaria Atlas Project. Malaria Atlas Project Plasmodium Falciparum Parasite Rate Database. Oxford, United Kingdom: Malaria Atlas Project

Kenya Division of Vector-Borne Diseases Monthly Report April 1976 as it appears in Malaria Atlas Project. Malaria Atlas Project Plasmodium Falciparum Parasite Rate Database. Oxford, United Kingdom: Malaria Atlas Project
Kenya Division of Vector-Borne Diseases Monthly Report April 1978 as it appears in Malaria Atlas Project. Malaria Atlas Project Plasmodium Falciparum Parasite Rate Database. Oxford, United Kingdom: Malaria Atlas Project
Kenya Division of Vector-Borne Diseases Monthly Report April 1979 as it appears in Malaria Atlas Project. Malaria Atlas Project Plasmodium Falciparum Parasite Rate Database. Oxford, United Kingdom: Malaria Atlas Project
Kenya Division of Vector-Borne Diseases Monthly Report April 1981 as it appears in Malaria Atlas Project. Malaria Atlas Project Plasmodium Falciparum Parasite Rate Database. Oxford, United Kingdom: Malaria Atlas Project
Kenya Division of Vector-Borne Diseases Monthly Report April 1984 as it appears in Malaria Atlas Project. Malaria Atlas Project Plasmodium Falciparum Parasite Rate Database. Oxford, United Kingdom: Malaria Atlas Project
Kenya Division of Vector-Borne Diseases Monthly Report April 1985 as it appears in Malaria Atlas Project. Malaria Atlas Project Plasmodium Falciparum Parasite Rate Database. Oxford, United Kingdom: Malaria Atlas Project
Kenya Division of Vector-Borne Diseases Monthly Report April 1991 as it appears in Malaria Atlas Project. Malaria Atlas Project Plasmodium Falciparum Parasite Rate Database. Oxford, United Kingdom: Malaria Atlas Project
Kenya Division of Vector-Borne Diseases Monthly Report April 1993 as it appears in Malaria Atlas Project. Malaria Atlas Project Plasmodium Falciparum Parasite Rate Database. Oxford, United Kingdom: Malaria Atlas Project
Kenya Division of Vector-Borne Diseases Monthly Report August - October 2002 as it appears in Malaria Atlas Project. Malaria Atlas Project Plasmodium Falciparum Parasite Rate Database. Oxford, United Kingdom: Malaria Atlas Project
Kenya Division of Vector-Borne Diseases Monthly Report August 1977 as it appears in Malaria Atlas Project. Malaria Atlas Project Plasmodium Falciparum Parasite Rate Database. Oxford, United Kingdom: Malaria Atlas Project
Kenya Division of Vector-Borne Diseases Monthly Report August 1979 as it appears in Malaria Atlas Project. Malaria Atlas Project Plasmodium Falciparum Parasite Rate Database. Oxford, United Kingdom: Malaria Atlas Project
Kenya Division of Vector-Borne Diseases Monthly Report August 1975 as it appears in Malaria Atlas Project. Malaria Atlas Project Plasmodium Falciparum Parasite Rate Database. Oxford, United Kingdom: Malaria Atlas Project
Kenya Division of Vector-Borne Diseases Monthly Report August 1985 as it appears in Malaria Atlas Project. Malaria Atlas Project Plasmodium Falciparum Parasite Rate Database. Oxford, United Kingdom: Malaria Atlas Project
Kenya Division of Vector-Borne Diseases Monthly Report August 1988 as it appears in Malaria Atlas Project. Malaria Atlas Project Plasmodium Falciparum Parasite Rate Database. Oxford, United Kingdom: Malaria Atlas Project
Kenya Division of Vector-Borne Diseases Monthly Report August 1982 as it appears in Malaria Atlas Project. Malaria Atlas Project Plasmodium Falciparum Parasite Rate Database. Oxford, United Kingdom: Malaria Atlas Project
Kenya Division of Vector-Borne Diseases Monthly Report December 1977 as it appears in Malaria Atlas Project. Malaria Atlas Project Plasmodium Falciparum Parasite Rate Database. Oxford, United Kingdom: Malaria Atlas Project

Kenya Division of Vector-Borne Diseases Monthly Report December 1982 as it appears in Malaria Atlas Project. Malaria Atlas Project Plasmodium Falciparum Parasite Rate Database. Oxford, United Kingdom: Malaria Atlas Project
Kenya Division of Vector-Borne Diseases Monthly Report February 1979 as it appears in Malaria Atlas Project. Malaria Atlas Project Plasmodium Falciparum Parasite Rate Database. Oxford, United Kingdom: Malaria Atlas Project
Kenya Division of Vector-Borne Diseases Monthly Report February 1976 as it appears in Malaria Atlas Project. Malaria Atlas Project Plasmodium Falciparum Parasite Rate Database. Oxford, United Kingdom: Malaria Atlas Project
Kenya Division of Vector-Borne Diseases Monthly Report February 1981 as it appears in Malaria Atlas Project. Malaria Atlas Project Plasmodium Falciparum Parasite Rate Database. Oxford, United Kingdom: Malaria Atlas Project
Kenya Division of Vector-Borne Diseases Monthly Report February 1975 as it appears in Malaria Atlas Project. Malaria Atlas Project Plasmodium Falciparum Parasite Rate Database. Oxford, United Kingdom: Malaria Atlas Project
Kenya Division of Vector-Borne Diseases Monthly Report January - February 1989 as it appears in Malaria Atlas Project. Malaria Atlas Project Plasmodium Falciparum Parasite Rate Database. Oxford, United Kingdom: Malaria Atlas Project
Kenya Division of Vector-Borne Diseases Monthly Report January 1988 as it appears in Malaria Atlas Project. Malaria Atlas Project Plasmodium Falciparum Parasite Rate Database. Oxford, United Kingdom: Malaria Atlas Project
Kenya Division of Vector-Borne Diseases Monthly Report January 1983 as it appears in Malaria Atlas Project. Malaria Atlas Project Plasmodium Falciparum Parasite Rate Database. Oxford, United Kingdom: Malaria Atlas Project
Kenya Division of Vector-Borne Diseases Monthly Report January 1985 as it appears in Malaria Atlas Project. Malaria Atlas Project Plasmodium Falciparum Parasite Rate Database. Oxford, United Kingdom: Malaria Atlas Project
Kenya Division of Vector-Borne Diseases Monthly Report January 1986 as it appears in Malaria Atlas Project. Malaria Atlas Project Plasmodium Falciparum Parasite Rate Database. Oxford, United Kingdom: Malaria Atlas Project
Kenya Division of Vector-Borne Diseases Monthly Report January 1997 as it appears in Malaria Atlas Project. Malaria Atlas Project Plasmodium Falciparum Parasite Rate Database. Oxford, United Kingdom: Malaria Atlas Project
Kenya Division of Vector-Borne Diseases Monthly Report July - August 2001 as it appears in Malaria Atlas Project. Malaria Atlas Project Plasmodium Falciparum Parasite Rate Database. Oxford, United Kingdom: Malaria Atlas Project
Kenya Division of Vector-Borne Diseases Monthly Report July - September 1998 as it appears in Malaria Atlas Project. Malaria Atlas Project Plasmodium Falciparum Parasite Rate Database. Oxford, United Kingdom: Malaria Atlas Project
Kenya Division of Vector-Borne Diseases Monthly Report July 1975 as it appears in Malaria Atlas Project. Malaria Atlas Project Plasmodium Falciparum Parasite Rate Database. Oxford, United Kingdom: Malaria Atlas Project
Kenya Division of Vector-Borne Diseases Monthly Report July 1976 as it appears in Malaria Atlas Project. Malaria Atlas Project Plasmodium Falciparum Parasite Rate Database. Oxford, United Kingdom: Malaria Atlas Project
Kenya Division of Vector-Borne Diseases Monthly Report July 1977 as it appears in Malaria Atlas Project. Malaria Atlas Project Plasmodium Falciparum Parasite Rate Database. Oxford, United Kingdom: Malaria Atlas Project

Kenya Division of Vector-Borne Diseases Monthly Report July 1982 as it appears in Malaria Atlas Project. Malaria Atlas Project Plasmodium Falciparum Parasite Rate Database. Oxford, United Kingdom: Malaria Atlas Project
Kenya Division of Vector-Borne Diseases Monthly Report July 1986 as it appears in Malaria Atlas Project. Malaria Atlas Project Plasmodium Falciparum Parasite Rate Database. Oxford, United Kingdom: Malaria Atlas Project
Kenya Division of Vector-Borne Diseases Monthly Report July 1988 as it appears in Malaria Atlas Project. Malaria Atlas Project Plasmodium Falciparum Parasite Rate Database. Oxford, United Kingdom: Malaria Atlas Project
Kenya Division of Vector-Borne Diseases Monthly Report July 1989 as it appears in Malaria Atlas Project. Malaria Atlas Project Plasmodium Falciparum Parasite Rate Database. Oxford, United Kingdom: Malaria Atlas Project
Kenya Division of Vector-Borne Diseases Monthly Report July 1991 as it appears in Malaria Atlas Project. Malaria Atlas Project Plasmodium Falciparum Parasite Rate Database. Oxford, United Kingdom: Malaria Atlas Project
Kenya Division of Vector-Borne Diseases Monthly Report July 1997 as it appears in Malaria Atlas Project. Malaria Atlas Project Plasmodium Falciparum Parasite Rate Database. Oxford, United Kingdom: Malaria Atlas Project
Kenya Division of Vector-Borne Diseases Monthly Report June - July 2004 as it appears in Malaria Atlas Project. Malaria Atlas Project Plasmodium Falciparum Parasite Rate Database. Oxford, United Kingdom: Malaria Atlas Project
Kenya Division of Vector-Borne Diseases Monthly Report June 1975 as it appears in Malaria Atlas Project. Malaria Atlas Project Plasmodium Falciparum Parasite Rate Database. Oxford, United Kingdom: Malaria Atlas Project
Kenya Division of Vector-Borne Diseases Monthly Report June 1976 as it appears in Malaria Atlas Project. Malaria Atlas Project Plasmodium Falciparum Parasite Rate Database. Oxford, United Kingdom: Malaria Atlas Project
Kenya Division of Vector-Borne Diseases Monthly Report June 1978 as it appears in Malaria Atlas Project. Malaria Atlas Project Plasmodium Falciparum Parasite Rate Database. Oxford, United Kingdom: Malaria Atlas Project
Kenya Division of Vector-Borne Diseases Monthly Report June 1979 as it appears in Malaria Atlas Project. Malaria Atlas Project Plasmodium Falciparum Parasite Rate Database. Oxford, United Kingdom: Malaria Atlas Project
Kenya Division of Vector-Borne Diseases Monthly Report June 1981 as it appears in Malaria Atlas Project. Malaria Atlas Project Plasmodium Falciparum Parasite Rate Database. Oxford, United Kingdom: Malaria Atlas Project
Kenya Division of Vector-Borne Diseases Monthly Report June 1983 as it appears in Malaria Atlas Project. Malaria Atlas Project Plasmodium Falciparum Parasite Rate Database. Oxford, United Kingdom: Malaria Atlas Project
Kenya Division of Vector-Borne Diseases Monthly Report June 1985 as it appears in Malaria Atlas Project. Malaria Atlas Project Plasmodium Falciparum Parasite Rate Database. Oxford, United Kingdom: Malaria Atlas Project
Kenya Division of Vector-Borne Diseases Monthly Report June 1991 as it appears in Malaria Atlas Project. Malaria Atlas Project Plasmodium Falciparum Parasite Rate Database. Oxford, United Kingdom: Malaria Atlas Project
Kenya Division of Vector-Borne Diseases Monthly Report June 2008 as it appears in Malaria Atlas Project. Malaria Atlas Project Plasmodium Falciparum Parasite Rate Database. Oxford, United Kingdom: Malaria Atlas Project

Kenya Division of Vector-Borne Diseases Monthly Report March 1976 as it appears in Malaria Atlas Project. Malaria Atlas Project Plasmodium Falciparum Parasite Rate Database. Oxford, United Kingdom: Malaria Atlas Project
Kenya Division of Vector-Borne Diseases Monthly Report March 1979 as it appears in Malaria Atlas Project. Malaria Atlas Project Plasmodium Falciparum Parasite Rate Database. Oxford, United Kingdom: Malaria Atlas Project
Kenya Division of Vector-Borne Diseases Monthly Report March 1980 as it appears in Malaria Atlas Project. Malaria Atlas Project Plasmodium Falciparum Parasite Rate Database. Oxford, United Kingdom: Malaria Atlas Project
Kenya Division of Vector-Borne Diseases Monthly Report March 1983 as it appears in Malaria Atlas Project. Malaria Atlas Project Plasmodium Falciparum Parasite Rate Database. Oxford, United Kingdom: Malaria Atlas Project
Kenya Division of Vector-Borne Diseases Monthly Report March 1986 as it appears in Malaria Atlas Project. Malaria Atlas Project Plasmodium Falciparum Parasite Rate Database. Oxford, United Kingdom: Malaria Atlas Project
Kenya Division of Vector-Borne Diseases Monthly Report March 1998 as it appears in Malaria Atlas Project. Malaria Atlas Project Plasmodium Falciparum Parasite Rate Database. Oxford, United Kingdom: Malaria Atlas Project
Kenya Division of Vector-Borne Diseases Monthly Report May 1975 as it appears in Malaria Atlas Project. Malaria Atlas Project Plasmodium Falciparum Parasite Rate Database. Oxford, United Kingdom: Malaria Atlas Project
Kenya Division of Vector-Borne Diseases Monthly Report May 1976 as it appears in Malaria Atlas Project. Malaria Atlas Project Plasmodium Falciparum Parasite Rate Database. Oxford, United Kingdom: Malaria Atlas Project
Kenya Division of Vector-Borne Diseases Monthly Report May 1978 as it appears in Malaria Atlas Project. Malaria Atlas Project Plasmodium Falciparum Parasite Rate Database. Oxford, United Kingdom: Malaria Atlas Project
Kenya Division of Vector-Borne Diseases Monthly Report May 1979 as it appears in Malaria Atlas Project. Malaria Atlas Project Plasmodium Falciparum Parasite Rate Database. Oxford, United Kingdom: Malaria Atlas Project
Kenya Division of Vector-Borne Diseases Monthly Report May 1988 as it appears in Malaria Atlas Project. Malaria Atlas Project Plasmodium Falciparum Parasite Rate Database. Oxford, United Kingdom: Malaria Atlas Project
Kenya Division of Vector-Borne Diseases Monthly Report May 1990 as it appears in Malaria Atlas Project. Malaria Atlas Project Plasmodium Falciparum Parasite Rate Database. Oxford, United Kingdom: Malaria Atlas Project
Kenya Division of Vector-Borne Diseases Monthly Report May 1992 as it appears in Malaria Atlas Project. Malaria Atlas Project Plasmodium Falciparum Parasite Rate Database. Oxford, United Kingdom: Malaria Atlas Project
Kenya Division of Vector-Borne Diseases Monthly Report May 1995 as it appears in Malaria Atlas Project. Malaria Atlas Project Plasmodium Falciparum Parasite Rate Database. Oxford, United Kingdom: Malaria Atlas Project
Kenya Division of Vector-Borne Diseases Monthly Report November 1977 as it appears in Malaria Atlas Project. Malaria Atlas Project Plasmodium Falciparum Parasite Rate Database. Oxford, United Kingdom: Malaria Atlas Project
Kenya Division of Vector-Borne Diseases Monthly Report November 1978 as it appears in Malaria Atlas Project. Malaria Atlas Project Plasmodium Falciparum Parasite Rate Database. Oxford, United Kingdom: Malaria Atlas Project

Kenya Division of Vector-Borne Diseases Monthly Report November 1979 as it appears in Malaria Atlas Project. Malaria Atlas Project Plasmodium Falciparum Parasite Rate Database. Oxford, United Kingdom: Malaria Atlas Project
Kenya Division of Vector-Borne Diseases Monthly Report November 1980 as it appears in Malaria Atlas Project. Malaria Atlas Project Plasmodium Falciparum Parasite Rate Database. Oxford, United Kingdom: Malaria Atlas Project
Kenya Division of Vector-Borne Diseases Monthly Report November 1985 as it appears in Malaria Atlas Project. Malaria Atlas Project Plasmodium Falciparum Parasite Rate Database. Oxford, United Kingdom: Malaria Atlas Project
Kenya Division of Vector-Borne Diseases Monthly Report November 1996 as it appears in Malaria Atlas Project. Malaria Atlas Project Plasmodium Falciparum Parasite Rate Database. Oxford, United Kingdom: Malaria Atlas Project
Kenya Division of Vector-Borne Diseases Monthly Report November 1987 as it appears in Malaria Atlas Project. Malaria Atlas Project Plasmodium Falciparum Parasite Rate Database. Oxford, United Kingdom: Malaria Atlas Project
Kenya Division of Vector-Borne Diseases Monthly Report November 1975 as it appears in Malaria Atlas Project. Malaria Atlas Project Plasmodium Falciparum Parasite Rate Database. Oxford, United Kingdom: Malaria Atlas Project
Kenya Division of Vector-Borne Diseases Monthly Report November 1983 as it appears in Malaria Atlas Project. Malaria Atlas Project Plasmodium Falciparum Parasite Rate Database. Oxford, United Kingdom: Malaria Atlas Project
Kenya Division of Vector-Borne Diseases Monthly Report November 1980 and 1982 as it appears in Malaria Atlas Project. Malaria Atlas Project Plasmodium Falciparum Parasite Rate Database. Oxford, United Kingdom: Malaria Atlas Project
Kenya Division of Vector-Borne Diseases Monthly Report October - December 1998 as it appears in Malaria Atlas Project. Malaria Atlas Project Plasmodium Falciparum Parasite Rate Database. Oxford, United Kingdom: Malaria Atlas Project
Kenya Division of Vector-Borne Diseases Monthly Report October 1978 as it appears in Malaria Atlas Project. Malaria Atlas Project Plasmodium Falciparum Parasite Rate Database. Oxford, United Kingdom: Malaria Atlas Project
Kenya Division of Vector-Borne Diseases Monthly Report October 1977 as it appears in Malaria Atlas Project. Malaria Atlas Project Plasmodium Falciparum Parasite Rate Database. Oxford, United Kingdom: Malaria Atlas Project
Kenya Division of Vector-Borne Diseases Monthly Report October 1980 as it appears in Malaria Atlas Project. Malaria Atlas Project Plasmodium Falciparum Parasite Rate Database. Oxford, United Kingdom: Malaria Atlas Project
Kenya Division of Vector-Borne Diseases Monthly Report October 1985 as it appears in Malaria Atlas Project. Malaria Atlas Project Plasmodium Falciparum Parasite Rate Database. Oxford, United Kingdom: Malaria Atlas Project
Kenya Division of Vector-Borne Diseases Monthly Report October 1995 as it appears in Malaria Atlas Project. Malaria Atlas Project Plasmodium Falciparum Parasite Rate Database. Oxford, United Kingdom: Malaria Atlas Project
Kenya Division of Vector-Borne Diseases Monthly Report October 1993 as it appears in Malaria Atlas Project. Malaria Atlas Project Plasmodium Falciparum Parasite Rate Database. Oxford, United Kingdom: Malaria Atlas Project
Kenya Division of Vector-Borne Diseases Monthly Report October 1987 as it appears in Malaria Atlas Project. Malaria Atlas Project Plasmodium Falciparum Parasite Rate Database. Oxford, United Kingdom: Malaria Atlas Project

Kenya Division of Vector-Borne Diseases Monthly Report October 1992 as it appears in Malaria Atlas Project. Malaria Atlas Project Plasmodium Falciparum Parasite Rate Database. Oxford, United Kingdom: Malaria Atlas Project
Kenya Division of Vector-Borne Diseases Monthly Report October 2007 as it appears in Malaria Atlas Project. Malaria Atlas Project Plasmodium Falciparum Parasite Rate Database. Oxford, United Kingdom: Malaria Atlas Project
Kenya Division of Vector-Borne Diseases Monthly Report September 1978 as it appears in Malaria Atlas Project. Malaria Atlas Project Plasmodium Falciparum Parasite Rate Database. Oxford, United Kingdom: Malaria Atlas Project
Kenya Division of Vector-Borne Diseases Monthly Report September 1976 as it appears in Malaria Atlas Project. Malaria Atlas Project Plasmodium Falciparum Parasite Rate Database. Oxford, United Kingdom: Malaria Atlas Project
Kenya Division of Vector-Borne Diseases Monthly Report September 1977 as it appears in Malaria Atlas Project. Malaria Atlas Project Plasmodium Falciparum Parasite Rate Database. Oxford, United Kingdom: Malaria Atlas Project
Kenya Division of Vector-Borne Diseases Monthly Report September 1995 as it appears in Malaria Atlas Project. Malaria Atlas Project Plasmodium Falciparum Parasite Rate Database. Oxford, United Kingdom: Malaria Atlas Project
Kenya Division of Vector-Borne Diseases Monthly Report September 1987 as it appears in Malaria Atlas Project. Malaria Atlas Project Plasmodium Falciparum Parasite Rate Database. Oxford, United Kingdom: Malaria Atlas Project
Kenya Division of Vector-Borne Diseases Monthly Report September 1975 as it appears in Malaria Atlas Project. Malaria Atlas Project Plasmodium Falciparum Parasite Rate Database. Oxford, United Kingdom: Malaria Atlas Project
Kenya Division of Vector-Borne Diseases Monthly Report September 1982 as it appears in Malaria Atlas Project. Malaria Atlas Project Plasmodium Falciparum Parasite Rate Database. Oxford, United Kingdom: Malaria Atlas Project
Kenya Division of Vector-Borne Diseases Monthly Report, Garsen, February 1978 as it appears in Malaria Atlas Project. Malaria Atlas Project Plasmodium Falciparum Parasite Rate Database. Oxford, United Kingdom: Malaria Atlas Project
Kenya Division of Vector-Borne Diseases Monthly Report, Hola District Hospital February 1978 as it appears in Malaria Atlas Project. Malaria Atlas Project Plasmodium Falciparum Parasite Rate Database. Oxford, United Kingdom: Malaria Atlas Project
Kenya Division of Vector-Borne Diseases Return for March 1990 as it appears in Malaria Atlas Project. Malaria Atlas Project Plasmodium Falciparum Parasite Rate Database. Oxford, United Kingdom: Malaria Atlas Project
Kenya Division of Vector-Borne Diseases Return for November 1989 as it appears in Malaria Atlas Project. Malaria Atlas Project Plasmodium Falciparum Parasite Rate Database. Oxford, United Kingdom: Malaria Atlas Project
Kenya Lohuk and Kampi Ya Samaki Malariometric Survey 1996 as it appears in Malaria Atlas Project. Malaria Atlas Project Plasmodium Falciparum Parasite Rate Database. Oxford, United Kingdom: Malaria Atlas Project
Kenya Malaria Indicator Survey 2010 as it appears in Malaria Atlas Project. Malaria Atlas Project Interventions Database
Kenya Malaria Indicator Survey 2015 as it appears in Malaria Atlas Project. Malaria Atlas Project Plasmodium Falciparum Parasite Rate Database. Oxford, United Kingdom: Malaria Atlas Project

Kenya Malaria Survey 1996 as it appears in Malaria Atlas Project. Malaria Atlas Project Plasmodium Falciparum Parasite Rate Database. Oxford, United Kingdom: Malaria Atlas Project
Kenya Malariometric Survey 1987 as it appears in Malaria Atlas Project. Malaria Atlas Project Plasmodium Falciparum Parasite Rate Database. Oxford, United Kingdom: Malaria Atlas Project
Kenya Malariometric Survey 1999 as it appears in Malaria Atlas Project. Malaria Atlas Project Plasmodium Falciparum Parasite Rate Database. Oxford, United Kingdom: Malaria Atlas Project
Kenya Malariometric Survey 2001 as it appears in Malaria Atlas Project. Malaria Atlas Project Plasmodium Falciparum Parasite Rate Database. Oxford, United Kingdom: Malaria Atlas Project
Kenya Malariometric/Entomological Report 2000 as it appears in Malaria Atlas Project. Malaria Atlas Project Plasmodium Falciparum Parasite Rate Database. Oxford, United Kingdom: Malaria Atlas Project
Kenya Medical Research Institute (KEMRI). Kenya - Nairobi Cancer Incidence Report 2000-2002. Kenya Medical Research Institute (KEMRI), 2006
Kenya Monthly Reports From Field Stations on Community-based Malaria Prevalence Surveys 1985–1997 as it appears in Malaria Atlas Project. Malaria Atlas Project Plasmodium Falciparum Parasite Rate Database. Oxford, United Kingdom: Malaria Atlas Project
Kenya National Bureau of Statistics, USAID, United Nations Population Fund (UNFPA), United States Census Bureau. Kenya Population and Housing Census 2009
Kenya National Micronutrient Survey 1999 as it appears in Malaria Atlas Project. Malaria Atlas Project Plasmodium Falciparum Parasite Rate Database. Oxford, United Kingdom: Malaria Atlas Project
Kenya Plasmodium Falciparum Parasite Rate Data 1979, Personal Communication with Masaba, Ministry of Health 1981 as it appears in Malaria Atlas Project. Malaria Atlas Project Plasmodium Falciparum Parasite Rate Database. Oxford, United Kingdom: Malaria Atlas Project
Kenya Plasmodium Falciparum Parasite Rate Data 1982-1983, Personal Communication with Murigi, Ministry of Health 1985 as it appears in Malaria Atlas Project. Malaria Atlas Project Plasmodium Falciparum Parasite Rate Database. Oxford, United Kingdom: Malaria Atlas Project
Kenya Plasmodium Falciparum Parasite Rate Data 1982-1984, Personal Communication with Murigi, Ministry of Health 1984 as it appears in Malaria Atlas Project. Malaria Atlas Project Plasmodium Falciparum Parasite Rate Database. Oxford, United Kingdom: Malaria Atlas Project
Kenya Plasmodium Falciparum Parasite Rate Data 1983, Personal Communication with Murigi, Ministry of Health 1990 as it appears in Malaria Atlas Project. Malaria Atlas Project Plasmodium Falciparum Parasite Rate Database. Oxford, United Kingdom: Malaria Atlas Project
Kenya Plasmodium Falciparum Parasite Rate Data 1983-1984, Personal Communication with Owino 1984 as it appears in Malaria Atlas Project. Malaria Atlas Project Plasmodium Falciparum Parasite Rate Database. Oxford, United Kingdom: Malaria Atlas Project
Kenya Plasmodium Falciparum Parasite Rate Data 1984, Personal Communication with Roemer 1985 as it appears in Malaria Atlas Project. Malaria Atlas Project Plasmodium Falciparum Parasite Rate Database. Oxford, United Kingdom: Malaria Atlas Project
Kenya Plasmodium Falciparum Parasite Rate Data 2008-2009, Personal Communication with the Division of Vector Borne Diseases 2009 as it appears in Malaria Atlas Project. Malaria Atlas

Project Plasmodium Falciparum Parasite Rate Database. Oxford, United Kingdom: Malaria Atlas Project

Kenya Plasmodium Falciparum Parasite Rate Data, A.A. Obala, Paper 46/91, 1991 as it appears in Malaria Atlas Project. Malaria Atlas Project Plasmodium Falciparum Parasite Rate Database. Oxford, United Kingdom: Malaria Atlas Project

Kenya Plasmodium Falciparum Parasite Rate Data, Adungo 1992 as it appears in Malaria Atlas Project. Malaria Atlas Project Plasmodium Falciparum Parasite Rate Database. Oxford, United Kingdom: Malaria Atlas Project

Kenya Plasmodium Falciparum Parasite Rate Data, B.A. Rapuoda 1995 as it appears in Malaria Atlas Project. Malaria Atlas Project Plasmodium Falciparum Parasite Rate Database. Oxford, United Kingdom: Malaria Atlas Project

Kenya Plasmodium Falciparum Parasite Rate Data, C.M. Mbogo 1990 as it appears in Malaria Atlas Project. Malaria Atlas Project Plasmodium Falciparum Parasite Rate Database. Oxford, United Kingdom: Malaria Atlas Project

Kenya Plasmodium Falciparum Parasite Rate Data, D. Ongore 1985 as it appears in Malaria Atlas Project. Malaria Atlas Project Plasmodium Falciparum Parasite Rate Database. Oxford, United Kingdom: Malaria Atlas Project

Kenya Plasmodium Falciparum Parasite Rate Data, E.O. Oongo, Division of Vector-Borne Diseases, Ministry of Health 1996 as it appears in Malaria Atlas Project. Malaria Atlas Project Plasmodium Falciparum Parasite Rate Database. Oxford, United Kingdom: Malaria Atlas Project

Kenya Plasmodium Falciparum Parasite Rate Data, K.M. K'Omollo, Division of Vector-Borne Diseases, Ministry of Health 1994 as it appears in Malaria Atlas Project. Malaria Atlas Project Plasmodium Falciparum Parasite Rate Database. Oxford, United Kingdom: Malaria Atlas Project

Kenya Plasmodium Falciparum Parasite Rate Data, MSc Thesis submitted by Kaseje DC, Kenyatta University 1989 as it appears in Malaria Atlas Project. Malaria Atlas Project Plasmodium Falciparum Parasite Rate Database. Oxford, United Kingdom: Malaria Atlas Project

Kenya Plasmodium Falciparum Parasite Rate Data, Personal Communication with U. Fillinger 2006 as it appears in Malaria Atlas Project. Malaria Atlas Project Plasmodium Falciparum Parasite Rate Database. Oxford, United Kingdom: Malaria Atlas Project

Kenya Plasmodium Falciparum Parasite Rate Data, Personal Communication with KEMRI-Welcome Trust Research Programme, Kilifi, 2008 as it appears in Malaria Atlas Project. Malaria Atlas Project Plasmodium Falciparum Parasite Rate Database. Oxford, United Kingdom: Malaria Atlas Project

Kenya Plasmodium Falciparum Parasite Rate Data, Personal Communication with KEMRI-Welcome Trust Research Programme, Kilifi, 2009 as it appears in Malaria Atlas Project. Malaria Atlas Project Plasmodium Falciparum Parasite Rate Database. Oxford, United Kingdom: Malaria Atlas Project

Kenya Plasmodium Falciparum Parasite Rate Data, Personal Communication with C.L. King 2009 as it appears in Malaria Atlas Project. Malaria Atlas Project Plasmodium Falciparum Parasite Rate Database. Oxford, United Kingdom: Malaria Atlas Project

Kenya Plasmodium Falciparum Parasite Rate Data, Personal Communication with H. Manda and L.C. Gouagna, International Centre for Insect Physiology and Ecology, 2009 as it appears in Malaria Atlas Project. Malaria Atlas Project Plasmodium Falciparum Parasite Rate Database. Oxford, United Kingdom: Malaria Atlas Project

Kenya Plasmodium Falciparum Parasite Rate Data, Personal Communication with K. Marsh 1995 as it appears in Malaria Atlas Project. Malaria Atlas Project Plasmodium Falciparum Parasite Rate Database. Oxford, United Kingdom: Malaria Atlas Project

Kenya Plasmodium Falciparum Parasite Rate Data, Personal Communication with V.M. Marsh and T. Abuya 2004 as it appears in Malaria Atlas Project. Malaria Atlas Project Plasmodium Falciparum Parasite Rate Database. Oxford, United Kingdom: Malaria Atlas Project

Kenya Plasmodium Falciparum Parasite Rate Data, Personal Communication with C.G. Nevill and R.W. Snow 1993 as it appears in Malaria Atlas Project. Malaria Atlas Project Plasmodium Falciparum Parasite Rate Database. Oxford, United Kingdom: Malaria Atlas Project

Kenya Plasmodium Falciparum Parasite Rate Data, Personal Communication with C.G. Nevill and R.W. Snow 1988 as it appears in Malaria Atlas Project. Malaria Atlas Project Plasmodium Falciparum Parasite Rate Database. Oxford, United Kingdom: Malaria Atlas Project

Kenya Plasmodium Falciparum Parasite Rate Data, Personal Communication with R.W. Snow 1991 as it appears in Malaria Atlas Project. Malaria Atlas Project Plasmodium Falciparum Parasite Rate Database. Oxford, United Kingdom: Malaria Atlas Project

Kenya Plasmodium Falciparum Parasite Rate Data, Personal Communication with D. Zurovac 2000 as it appears in Malaria Atlas Project. Malaria Atlas Project Plasmodium Falciparum Parasite Rate Database. Oxford, United Kingdom: Malaria Atlas Project

Kenya Plasmodium Falciparum Parasite Rate Data, Personal Communication with C. Gitonga and S. Brooker 2009 as it appears in Malaria Atlas Project. Malaria Atlas Project Plasmodium Falciparum Parasite Rate Database. Oxford, United Kingdom: Malaria Atlas Project

Kenya Plasmodium Falciparum Parasite Rate Data, Personal Communication with S.S. Imbahale, A. Githeko, and W. Takken 2009 as it appears in Malaria Atlas Project. Malaria Atlas Project Plasmodium Falciparum Parasite Rate Database. Oxford, United Kingdom: Malaria Atlas Project

Kenya Plasmodium Falciparum Parasite Rate Data, Personal Communication with R.W. Snow, A. Noor, and C. Gitonga, Nairobi KEMRI-Wellcome Trust Research Programme 2009 as it appears in Malaria Atlas Project. Malaria Atlas Project Plasmodium Falciparum Parasite Rate Database. Oxford, United Kingdom: Malaria Atlas Project

Kenya Plasmodium Falciparum Parasite Rate Data, Personal Communication with W. Takken, S. Imbahale, A. Githeko, and A. Busula, Medical and Veterinary Entomology at Wageningen University and Research Centre 2007 as it appears in Malaria Atlas Project. Malaria Atlas Project Plasmodium Falciparum Parasite Rate Database. Oxford, United Kingdom: Malaria Atlas Project

Kenya Plasmodium Falciparum Parasite Rate Data, Personal Communication with the Division of Malaria Control 2007 as it appears in Malaria Atlas Project. Malaria Atlas Project Plasmodium Falciparum Parasite Rate Database. Oxford, United Kingdom: Malaria Atlas Project

Kenya Plasmodium Falciparum Parasite Rate Data, Personal Communication with J. Kihara 2007 as it appears in Malaria Atlas Project. Malaria Atlas Project Plasmodium Falciparum Parasite Rate Database. Oxford, United Kingdom: Malaria Atlas Project

Kenya Plasmodium Falciparum Parasite Rate Data, Personal Communication with J. Kahara 2008 as it appears in Malaria Atlas Project. Malaria Atlas Project Plasmodium Falciparum Parasite Rate Database. Oxford, United Kingdom: Malaria Atlas Project

Kenya Plasmodium Falciparum Parasite Rate Data, Personal Communication with N. Minakawa 2009 as it appears in Malaria Atlas Project. Malaria Atlas Project Plasmodium Falciparum Parasite Rate Database. Oxford, United Kingdom: Malaria Atlas Project

Kenya Plasmodium Falciparum Parasite Rate Data, Personal Communication with the Division of Vector Borne Diseases 2008 as it appears in Malaria Atlas Project. Malaria Atlas Project

Plasmodium Falciparum Parasite Rate Database. Oxford, United Kingdom: Malaria Atlas Project
Kenya Plasmodium Falciparum Parasite Rate Data, Personal Communication with Cox 2009 as it appears in Malaria Atlas Project. Malaria Atlas Project Plasmodium Falciparum Parasite Rate Database. Oxford, United Kingdom: Malaria Atlas Project
Kenya Plasmodium Falciparum Parasite Rate Data, Personal Communication with KEMRI/CDC 2009 as it appears in Malaria Atlas Project. Malaria Atlas Project Plasmodium Falciparum Parasite Rate Database. Oxford, United Kingdom: Malaria Atlas Project
Kenya Plasmodium Falciparum Parasite Rate Data, Personal Communication with KEMRI/CDC 2007 as it appears in Malaria Atlas Project. Malaria Atlas Project Plasmodium Falciparum Parasite Rate Database. Oxford, United Kingdom: Malaria Atlas Project
Kenya Plasmodium Falciparum Parasite Rate Data, Personal Communication with Merlin 1999 as it appears in Malaria Atlas Project. Malaria Atlas Project Plasmodium Falciparum Parasite Rate Database. Oxford, United Kingdom: Malaria Atlas Project
Kenya Plasmodium Falciparum Parasite Rate Data, Personal Communication with Muigai 2006 as it appears in Malaria Atlas Project. Malaria Atlas Project Plasmodium Falciparum Parasite Rate Database. Oxford, United Kingdom: Malaria Atlas Project
Kenya Plasmodium Falciparum Parasite Rate Data, Personal Communication with Mui'gau 2004 as it appears in Malaria Atlas Project. Malaria Atlas Project Plasmodium Falciparum Parasite Rate Database. Oxford, United Kingdom: Malaria Atlas Project
Kenya Plasmodium Falciparum Parasite Rate Data, Personal Communication with Mwamguza 2002 as it appears in Malaria Atlas Project. Malaria Atlas Project Plasmodium Falciparum Parasite Rate Database. Oxford, United Kingdom: Malaria Atlas Project
Kenya Plasmodium Falciparum Parasite Rate Data, Personal Communication with Kanyi 2005 as it appears in Malaria Atlas Project. Malaria Atlas Project Plasmodium Falciparum Parasite Rate Database. Oxford, United Kingdom: Malaria Atlas Project
Kenya Plasmodium Falciparum Parasite Rate Data, Personal Communication with Otunge 2004 as it appears in Malaria Atlas Project. Malaria Atlas Project Plasmodium Falciparum Parasite Rate Database. Oxford, United Kingdom: Malaria Atlas Project
Kenya Plasmodium Falciparum Parasite Rate Data, Personal Communication with Namolo 2006 as it appears in Malaria Atlas Project. Malaria Atlas Project Plasmodium Falciparum Parasite Rate Database. Oxford, United Kingdom: Malaria Atlas Project
Kenya Plasmodium Falciparum Parasite Rate Data, Personal Communication with Onyango 2006 as it appears in Malaria Atlas Project. Malaria Atlas Project Plasmodium Falciparum Parasite Rate Database. Oxford, United Kingdom: Malaria Atlas Project
Kenya Plasmodium Falciparum Parasite Rate Data, Personal Communication with Nyago 2006 as it appears in Malaria Atlas Project. Malaria Atlas Project Plasmodium Falciparum Parasite Rate Database. Oxford, United Kingdom: Malaria Atlas Project
Kenya Plasmodium Falciparum Parasite Rate Data, Personal Communication with Nyago 2007 as it appears in Malaria Atlas Project. Malaria Atlas Project Plasmodium Falciparum Parasite Rate Database. Oxford, United Kingdom: Malaria Atlas Project
Kenya Plasmodium Falciparum Parasite Rate Data, Personal Communication with Oloo 2006 as it appears in Malaria Atlas Project. Malaria Atlas Project Plasmodium Falciparum Parasite Rate Database. Oxford, United Kingdom: Malaria Atlas Project
Kenya Plasmodium Falciparum Parasite Rate Data, Personal Communication with Otieno 2005 as it appears in Malaria Atlas Project. Malaria Atlas Project Plasmodium Falciparum Parasite Rate Database. Oxford, United Kingdom: Malaria Atlas Project

Kenya Plasmodium Falciparum Parasite Rate Data, Personal Communication with Otieno 2006 as it appears in Malaria Atlas Project. Malaria Atlas Project Plasmodium Falciparum Parasite Rate Database. Oxford, United Kingdom: Malaria Atlas Project

Kenya Plasmodium Falciparum Parasite Rate Data, Personal Communication with Onjore 2001 as it appears in Malaria Atlas Project. Malaria Atlas Project Plasmodium Falciparum Parasite Rate Database. Oxford, United Kingdom: Malaria Atlas Project

Kenya Plasmodium Falciparum Parasite Rate Data, Personal Communication with Odour 2006 as it appears in Malaria Atlas Project. Malaria Atlas Project Plasmodium Falciparum Parasite Rate Database. Oxford, United Kingdom: Malaria Atlas Project

Kenya Plasmodium Falciparum Parasite Rate Data, Personal Communication with Koech 2002 as it appears in Malaria Atlas Project. Malaria Atlas Project Plasmodium Falciparum Parasite Rate Database. Oxford, United Kingdom: Malaria Atlas Project

Kenya Plasmodium Falciparum Parasite Rate Data, Personal Communication with Rotich 2004 as it appears in Malaria Atlas Project. Malaria Atlas Project Plasmodium Falciparum Parasite Rate Database. Oxford, United Kingdom: Malaria Atlas Project

Kenya Plasmodium Falciparum Parasite Rate Data, Personal Communication with Boiyon 2004 as it appears in Malaria Atlas Project. Malaria Atlas Project Plasmodium Falciparum Parasite Rate Database. Oxford, United Kingdom: Malaria Atlas Project

Kenya Plasmodium Falciparum Parasite Rate Data, Personal Communication with Wangonyi 2002 as it appears in Malaria Atlas Project. Malaria Atlas Project Plasmodium Falciparum Parasite Rate Database. Oxford, United Kingdom: Malaria Atlas Project

Kenya Plasmodium Falciparum Parasite Rate Data, Personal Communication with Kakai 2005 as it appears in Malaria Atlas Project. Malaria Atlas Project Plasmodium Falciparum Parasite Rate Database. Oxford, United Kingdom: Malaria Atlas Project

Kenya Plasmodium Falciparum Parasite Rate Data, Personal Communication with Kakai 2006 as it appears in Malaria Atlas Project. Malaria Atlas Project Plasmodium Falciparum Parasite Rate Database. Oxford, United Kingdom: Malaria Atlas Project

Kenya Plasmodium Falciparum Parasite Rate Data, Personal Communication with Chemuliti 2005 as it appears in Malaria Atlas Project. Malaria Atlas Project Plasmodium Falciparum Parasite Rate Database. Oxford, United Kingdom: Malaria Atlas Project

Kenya Plasmodium Falciparum Parasite Rate Data, Personal Communication with Agak, Ministry of Health 1980 as it appears in Malaria Atlas Project. Malaria Atlas Project Plasmodium Falciparum Parasite Rate Database. Oxford, United Kingdom: Malaria Atlas Project

Kenya Plasmodium Falciparum Parasite Rate Data, Personal Communication with Agak, Ministry of Health 1981 as it appears in Malaria Atlas Project. Malaria Atlas Project Plasmodium Falciparum Parasite Rate Database. Oxford, United Kingdom: Malaria Atlas Project

Kenya Plasmodium Falciparum Parasite Rate Data, Personal Communication with Makazi, Ministry of Health 1976 as it appears in Malaria Atlas Project. Malaria Atlas Project Plasmodium Falciparum Parasite Rate Database. Oxford, United Kingdom: Malaria Atlas Project

Kenya Plasmodium Falciparum Parasite Rate Data, Personal Communication with Makazi, Ministry of Health 1975 as it appears in Malaria Atlas Project. Malaria Atlas Project Plasmodium Falciparum Parasite Rate Database. Oxford, United Kingdom: Malaria Atlas Project

Kenya Plasmodium Falciparum Parasite Rate Data, Personal Communication with Makazi, Ministry of Health 1981 as it appears in Malaria Atlas Project. Malaria Atlas Project Plasmodium Falciparum Parasite Rate Database. Oxford, United Kingdom: Malaria Atlas Project

Kenya Plasmodium Falciparum Parasite Rate Data, Personal Communication with Mugo, Ministry of Health 1981 as it appears in Malaria Atlas Project. Malaria Atlas Project Plasmodium Falciparum Parasite Rate Database. Oxford, United Kingdom: Malaria Atlas Project

Kenya Plasmodium Falciparum Parasite Rate Data, Personal Communication with Mugo, Ministry of Health 1983 as it appears in Malaria Atlas Project. Malaria Atlas Project Plasmodium Falciparum Parasite Rate Database. Oxford, United Kingdom: Malaria Atlas Project

Kenya Plasmodium Falciparum Parasite Rate Data, Personal Communication with Muthinja, Ministry of Health 1984 as it appears in Malaria Atlas Project. Malaria Atlas Project Plasmodium Falciparum Parasite Rate Database. Oxford, United Kingdom: Malaria Atlas Project

Kenya Plasmodium Falciparum Parasite Rate Data, Personal Communication with Ndzovu, Ministry of Health 1981 as it appears in Malaria Atlas Project. Malaria Atlas Project Plasmodium Falciparum Parasite Rate Database. Oxford, United Kingdom: Malaria Atlas Project

Kenya Plasmodium Falciparum Parasite Rate Data, Personal Communication with Omar, Ministry of Health 1982 as it appears in Malaria Atlas Project. Malaria Atlas Project Plasmodium Falciparum Parasite Rate Database. Oxford, United Kingdom: Malaria Atlas Project

Kenya Plasmodium Falciparum Parasite Rate Data, S.B. Tosha, Division of Vector-Borne Diseases, Ministry of Health 1996 as it appears in Malaria Atlas Project. Malaria Atlas Project Plasmodium Falciparum Parasite Rate Database. Oxford, United Kingdom: Malaria Atlas Project

Kenya Plasmodium Falciparum Parasite Rate Data, T. Mugo, Division of Vector-Borne Diseases, Ministry of Health 1994 as it appears in Malaria Atlas Project. Malaria Atlas Project Plasmodium Falciparum Parasite Rate Database. Oxford, United Kingdom: Malaria Atlas Project

Kenya Ramada and Pumwani Primary Schools Malaria and Schistosomiasis Baseline Survey 1993 as it appears in Malaria Atlas Project. Malaria Atlas Project Plasmodium Falciparum Parasite Rate Database. Oxford, United Kingdom: Malaria Atlas Project

Khagayi S, Burton DC, Onkoba R, Ochieng B, Ismail A, Mutonga D, Muthoni J, Feikin DR, Breiman RF, Mwenda JM, Odhiambo F, Laserson KF. High burden of rotavirus gastroenteritis in young children in rural western Kenya, 2010-2011. Pediatr Infect Dis J. 2014; S34-40

Kimani EW, Vulule JM, Kuria IW, Mugisha F. Use of insecticide-treated clothes for personal protection against malaria: a community trial. Malar J. 2006; 5: 63 as it appears in Malaria Atlas Project. Malaria Atlas Project Plasmodium Falciparum Parasite Rate Database. Oxford, United Kingdom: Malaria Atlas Project

King CH, Keating CE, Muruka JF, Ouma JH, Houser H, Siongok TK, Mahmoud AA. Urinary tract morbidity in schistosomiasis haematobia: associations with age and intensity of infection in an endemic area of Coast Province, Kenya. Am J Trop Med Hyg. 1988; 39(4): 361-8 as it appears in Malaria Atlas Project. Malaria Atlas Project Plasmodium Falciparum Parasite Rate Database. Oxford, United Kingdom: Malaria Atlas Project

King CH, Lombardi G, Lombardi C, Greenblatt R, Hodder S, Kinyanjui H, Ouma J, Odiambo O, Bryan PJ, Muruka J. Chemotherapy-based control of schistosomiasis haematobia. I. Metrifonate versus praziquantel in control of intensity and prevalence of infection. Am J Trop Med Hyg. 1988; 39(3): 295-305

King CH, Muchiri EM, Mungai P, Ouma JH, Kadzo H, Magak P, Koech DK. Randomized comparison of low-dose versus standard-dose praziquantel therapy in treatment of urinary tract morbidity due to Schistosoma haema tobium infection. Am J Trop Med Hyg. 2002; 66(6): 725-30

Kiulia NM, Peenze I, Dewar J, Nyachieo A, Galo M, Omolo E, Steele AD, Mwenda JM. Molecular characterisation of the rotavirus strains prevalent in Maua, Meru North, Kenya. East Afr Med J. 2006; 83(7): 360-5

Kloos H, Fulford AJC, Butterworth AE, Sturrock RF, Ouma JH, Kariuki HC, Thiongo FW, Dalton PR, Klumpp RK. Spatial patterns of human water contact and Schistosoma mansoni transmission

and infection in four rural areas in Machakos District, Kenya. Soc Sci Med. 1997; 44(7): 949-68

Koenraadt CJ, Paaijmans KP, Schneider P, Githeko AK, Takken W. Low larval vector survival explains unstable malaria in the western Kenya highlands. Trop Med Int Health. 2006; 11(8): 1195-205 as it appears in Malaria Atlas Project. Malaria Atlas Project Plasmodium Falciparum Parasite Rate Database. Oxford, United Kingdom: Malaria Atlas Project

Kolaczinski JH, Reithinger R, Worku DT, Ocheng A, Kasimiro J, Kabatereine N, Brooker S. Risk factors of visceral leishmaniasis in East Africa: a case-control study in Pokot territory of Kenya and Uganda. Int J Epidemiol. 2008; 37(2): 344-52 as it appears in Malaria Atlas Project. Malaria Atlas Project Plasmodium Falciparum Parasite Rate Database. Oxford, United Kingdom: Malaria Atlas Project

Kotloff KL, Nataro JP, Blackwelder WC, Nasrin D, Farag TH, Panchalingam S, Wu Y, Sow SO, Sur D, Breiman RF, Faruque AS, Zaidi AK, Saha D, Alonso PL, Tamboura B, Sanogo D, Onwuchekwa U, Manna B, Ramamurthy T, Kanungo S, Ochieng JB, Omore R, Oundo JO, Hossain A, Das SK, Ahmed S, Qureshi S, Quadri F, Adegbola RA, Antonio M, Hossain MJ, Akinsola A, Mandomando I, Nhampossa T, Acácio S, Biswas K, O'Reilly CE, Mintz ED, Berkeley LY, Muhsen K, Sommerfelt H, Robins-Browne RM, Levine MM. Burden and aetiology of diarrhoeal disease in infants and young children in developing countries (the Global Enteric Multicenter Study, GEMS): a prospective, case-control study. Lancet. 2013; 382(9888): 209–22

Koukounari A, Estambale BBA, Njagi JK, Cundill B, Ajanga A, Crudder C, Otido J, Jukes M, Clarke SE, Brooker S. Relationships between anaemia and parasitic infections in Kenyan schoolchildren: a Bayesian hierarchical modelling approach. Int J Parasitol. 2008; 38(14): 1663-71

Lawless JW, Latham MC, Stephenson LS, Kinoti SN, Pertet AM. Iron supplementation improves appetite and growth in anemic Kenyan primary school children. J Nutr. 1994; 124(5): 645-54 as it appears in Malaria Atlas Project. Malaria Atlas Project Plasmodium Falciparum Parasite Rate Database. Oxford, United Kingdom: Malaria Atlas Project

Le Sueur D, Binka F, Lengeler C, De Savigny D, Snow B, Teuscher T, Toure Y. An atlas of malaria in Africa. Afr Health. 1997; 19(2): 23-4 as it appears in Malaria Atlas Project. Malaria Atlas Project Plasmodium Falciparum Parasite Rate Database. Oxford, United Kingdom: Malaria Atlas Project

Leenstra T, Kariuki SK, Kurtis JD, Oloo AJ, Kager PA, ter Kuile FO. Prevalence and severity of anemia and iron deficiency: cross-sectional studies in adolescent schoolgirls in western Kenya. Eur J Clin Nutr. 2004; 58(4): 681-91

Leenstra T, Petersen LT, Kariuki SK, Oloo AJ, Kager PA, ter Kuile FO. Prevalence and severity of malnutrition and age at menarche; cross-sectional studies in adolescent schoolgirls in western Kenya. Eur J Clin Nutr. 2005; 59(1): 41-8 as it appears in Malaria Atlas Project. Malaria Atlas Project Plasmodium Falciparum Parasite Rate Database. Oxford, United Kingdom: Malaria Atlas Project

Lo E, Zhou G, Oo W, Afrane Y, Githeko A, Yan G. Low parasitemia in submicroscopic infections significantly impacts malaria diagnostic sensitivity in the highlands of Western Kenya. PLoS One. 2015; 10(3): e0121763 as it appears in Malaria Atlas Project. Malaria Atlas Project Plasmodium Falciparum Parasite Rate Database. Oxford, United Kingdom: Malaria Atlas Project

London School of Hygiene and Tropical Medicine. Global Atlas of Helminth Infections - Schistosomiasis. London, United Kingdom: London School of Hygiene and Tropical Medicine

Magnussen P, Muchiri E, Mungai P, Ndzovu M, Ouma J, Tosha S. A school-based approach to the control of urinary schistosomiasis and intestinal helminth infections in children in Matuga,

Kenya: impact of a two-year chemotherapy programme on prevalence and intensity of infections. Trop Med Int Health. 1997; 2(9): 825-31

Mahamud A, Burton A, Hassan M, Ahmed JA, Wagacha JB, Spiegel P, Haskew C, Eidex RB, Shetty S, Cookson S, Navarro-Colorado C, Goodson JL. Risk factors for measles mortality among hospitalized Somali refugees displaced by famine, Kenya, 2011. Clin Infect Dis. 2013; 57(8): e160-6

Mahieu JM, Muller AS, Voorhoeve AM, Dikken H. Pertussis in a rural area of Kenya: epidemiology and a preliminary report on a vaccine trial. Bull World Health Organ. 1978; 56(5): 773-80

Makino Y, Matsumoto I, Chiba Y, Mohammed OA, Ogaja PO, Kibue AM, Muli JM, Nakitare GW. Virological survey of children in Nyeri and Mombasa. Monthly survey of rotavirus in faeces. East Afr Med J. 1983; 60(8): 536-41

Malaria 1982-1997 as it appears in Malaria Atlas Project. Malaria Atlas Project Annual Parasite Incidence Database

Malaria Endemicity in Central Division, Kitui District, Kenya, October, 1983 as it appears in Malaria Atlas Project. Malaria Atlas Project Plasmodium Falciparum Parasite Rate Database. Oxford, United Kingdom: Malaria Atlas Project

Malaria Epidemiology and Drug Resistance in Kisii, Kenya as it appears in Malaria Atlas Project. Malaria Atlas Project Plasmodium Falciparum Parasite Rate Database. Oxford, United Kingdom: Malaria Atlas Project

Malariometric Surveillance Report - Tot Division as it appears in Malaria Atlas Project. Malaria Atlas Project Plasmodium Falciparum Parasite Rate Database. Oxford, United Kingdom: Malaria Atlas Project

Malawi Plasmodium Falciparum Parasite Rate Data, Personal Communication with D.P. Mathanga, S. Brooker, and K.E. Halliday 2013 as it appears in Malaria Atlas Project. Malaria Atlas Project Plasmodium Falciparum Parasite Rate Database. Oxford, United Kingdom: Malaria Atlas Project

Maman D, Pujades-Rodriguez M, Nicholas S, McGuire M, Szumilin E, Ecochard R, Etard J-F. Response to antiretroviral therapy: improved survival associated with CD4 above 500?cells/?l. AIDS. 2012; 26(11): 1393-8

Marston BJ, Macharia DK, Nga'nga L, Wangai M, Ilako F, Muhenje O, Kjaer M, Isavwa A, Kim A, Chebet K, Decock KM, Weidle PJ. A program to provide antiretroviral therapy to residents of an urban slum in nairobi, kenya. J Int Assoc Physicians AIDS Care (Chic). 2007; 6(2): 106-12

Masaba S. Schistosomiasis in Bunyala and Samia locations of Western Kenya. East Afr Med J. 1978; 55(11): 497-500

Masaba SC, Awiti IE, Muruka JF. Morbidity in urinary schistosomiasis in relation to the intensity of infection in Kisumu, Kenya. J Trop Med Hyg. 1983; 86(2): 65-6

Mbogo CM, Mwangangi JM, Nzovu J, Gu W, Yan G, Gunter JT, Swalm C, Keating J, Regens JL, Shililu JI, Githure JI, Beier JC. Spatial and temporal heterogeneity of Anopheles mosquitoes and Plasmodium falciparum transmission along the Kenyan coast. Am J Trop Med Hyg. 2003; 68(6): 734-42 as it appears in Malaria Atlas Project. Malaria Atlas Project Plasmodium Falciparum Parasite Rate Database. Oxford, United Kingdom: Malaria Atlas Project

McMorrow ML, Emukule GO, Njuguna HN, Bigogo G, Montgomery JM, Nyawanda B, Audi A, Breiman RF, Katz MA, Cosmas L, Waiboci LW, Duque J, Widdowson MA, Mott JA. The Unrecognized Burden of Influenza in Young Kenyan Children, 2008-2012. PLoS One. 2015; 10(9): e0138272

Menge I, Esamai F, van Reken D, Anabwani G. Paediatric morbidity and mortality at the Eldoret District Hospital, Kenya. East Afr Med J. 1995; 72(3): 165-9

Microscopy Versus Home-based Presumptive Diagnosis of Malaria in a Rural Community in Western Kenya as it appears in Malaria Atlas Project. Malaria Atlas Project Plasmodium Falciparum Parasite Rate Database. Oxford, United Kingdom: Malaria Atlas Project

Mirza NM, Macharia WM, Wafula EM, Agwanda R, Onyango FE. Mortality patterns in a rural Kenyan community. East Afr Med J. 1990; 67(11): 823-9

Mkoji GM, Muchemi GK, Kipesha FS, Mungai BN, Machai P. Schistosoma mansoni ova in urine of children from an endemic area of Kenya: a short report. East Afr Med J. 1998; 75(9): 558–9

Muller AS, Leeuwenburg J, Voorhoeve AM. Pertussis in a rural area of Kenya: epidemiology and results of a vaccine trial. Bull World Health Organ. 1984; 62(6): 899-908

Munyekenye OG, Githeko AK, Zhou G, Mushinzimana E, Minakawa N, Yan G. Plasmodium falciparum: Spatial Analysis, Western Kenya Highlands. Emerg Infect Dis. 2005; 11(10): 1571-7 as it appears in Malaria Atlas Project. Malaria Atlas Project Plasmodium Falciparum Parasite Rate Database. Oxford, United Kingdom: Malaria Atlas Project

Mutahi WT, Thiong'o FW. Prevalence and intensity of Schistosomiasis mansoni in irrigation and non-irrigation areas of central Kenya. East Afr Med J. 2005; 82(11): 586-91

Mutanda LN, Kangethe SK, Juma R, Lichenga EO, Gathecha C. Aetiology of diarrhoea in malnourished children at Kenyatta National Hospital. East Afr Med J. 1985; 62(12): 835-41

Mutanda LN, Kinoti SN, Gemert W, Lichenga EO. Age distribution and seasonal pattern of rotavirus infection in children in Kenya. J Diarrhoeal Dis Res. 1984; 2(3): 147-50

Mutanda LN. Epidemiology of acute gastroenteritis in early childhood in Kenya. III. Distribution of the aetiological agents. East Afr Med J. 1980; 57(5): 317-26

Mutanda LN. Epidemiology of acute gastroenteritis in early childhood in Kenya: aetiological agents. Trop Geogr Med. 1980; 32(2): 138-44

Mutero CM, Kabutha C, Kimani V, Kabuage L, Gitau G, Ssennyonga J, Githure J, Muthami L, Kaida A, Musyoka L, Kiarie E, Oganda M. A transdisciplinary perspective on the links between malaria and agroecosystems in Kenya. Addict Behav Rep. 2004; 89(2): 171-86 as it appears in Malaria Atlas Project. Malaria Atlas Project Plasmodium Falciparum Parasite Rate Database. Oxford, United Kingdom: Malaria Atlas Project

Mutero CM, Mutinga MJ, Ngindu AM, Kenya PR, Amimo FA. Visceral leishmaniasis and malaria prevalence in West Pokot District, Kenya. East Afr Med J. 1992; 69(1): 3-8 as it appears in Malaria Atlas Project. Malaria Atlas Project Plasmodium Falciparum Parasite Rate Database. Oxford, United Kingdom: Malaria Atlas Project

Mutero CM, Ouma JH, Agak BK, Wanderi JA, Copeland RS. Malaria prevalence and use of self-protection measures against mosquitoes in Suba District, Kenya. East Afr Med J. 1998; 75(1): 11-5 as it appears in Malaria Atlas Project. Malaria Atlas Project Plasmodium Falciparum Parasite Rate Database. Oxford, United Kingdom: Malaria Atlas Project

Mutinga MJ, Mnzava A, Kimokoti R, Nyamori M, Ngindu AM. Malaria prevalence and morbidity in relation to the use of permethrin-treated wall cloths in Kenya. East Afr Med J. 1993; 70(12): 756-62 as it appears in Malaria Atlas Project. Malaria Atlas Project Plasmodium Falciparum Parasite Rate Database. Oxford, United Kingdom: Malaria Atlas Project

Mutonga D, Langat D, Mwangi D, Tonui J, Njeru M, Abade A, Irura Z, Njeru I, Dahlke M. National surveillance data on the epidemiology of cholera in Kenya, 1997-2010. J Infect Dis. 2013; S55–61

Mutuku FM, King CH, Bustinduy AL, Mungai PL, Muchiri EM, Kitron U. Impact of drought on the spatial pattern of transmission of Schistosoma haematobium in coastal Kenya. Am J Trop Med Hyg. 2011; 85(6): 1065-70

Mwangi J, Gatei DG. Hepatitis B virus, hepatocellular carcinoma and liver cirrhosis in Kenya. East Afr Med J. 1993; 70(4 Suppl): 34-6

Mwangi JW. Viral markers in a blood donor population. East Afr Med J. 1999; 76(1): 35-7
Mwaniki D, Omondi B, Muniu E, Thiong'o F, Ouma J, Magnussen P, Geissler PW, Michaelsen KF, Friis H. Effects on serum retinol of multi-micronutrient supplementation and multi-helminth chemotherapy: a randomised, controlled trial in Kenyan school children. Eur J Clin Nutr. 2002; 56(7): 666-73
Mwenda JM, Ntoto KM, Abebe A, Enweronu-Laryea C, Amina I, Mchomvu J, Kisakye A, Mpabalwani EM, Pazvakavambwa I, Armah GE, Seheri LM, Kiulia NM, Page N, Widdowson M-A, Steele AD. Burden and epidemiology of rotavirus diarrhea in selected African countries: preliminary results from the African Rotavirus Surveillance Network. J Infect Dis. 2010; 202(Suppl): S5-11
Nabakwe EC, Lichtenbelt WVM, Ngare DK, Wierik M, Westerterp KR, Owino OC. Vitamin a deficiency and anaemia in young children living in a malaria endemic district of western Kenya. East Afr Med J. 2005; 82(6): 300-6 as it appears in Malaria Atlas Project. Malaria Atlas Project Plasmodium Falciparum Parasite Rate Database. Oxford, United Kingdom: Malaria Atlas Project
Nagi S, Chadeka EA, Sunahara T, Mutungi F, Justin YK, Kaneko S, Ichinose Y, Matsumoto S, Njenga SM, Hashizume M, Shimada M, Hamano S. Risk factors and spatial distribution of Schistosoma mansoni infection among primary school children in Mbita District, Western Kenya. PLoS Negl Trop Dis. 2014; 8(7): e2991 as it appears in Malaria Atlas Project. Malaria Atlas Project Plasmodium Falciparum Parasite Rate Database. Oxford, United Kingdom: Malaria Atlas Project
Nakata S, Gatheru Z, Ukae S, Adachi N, Kobayashi N, Honma S, Muli J, Ogaja P, Nyangao J, Kiplagat E, Tukei PM, Chiba S. Epidemiological study of the G serotype distribution of group A rotaviruses in Kenya from 1991 to 1994. J Med Virol. 1999; 58(3): 296-303
Nasrin D, Wu Y, Blackwelder WC, Farag TH, Saha D, Sow SO, Alonso PL, Breiman RF, Sur D, Faruque ASG, Zaidi AKM, Biswas K, Van Eijk AM, Walker DG, Levine MM, Kotloff KL. Health care seeking for childhood diarrhea in developing countries: evidence from seven sites in Africa and Asia. Am J Trop Med Hyg. 2013; 89(1 Suppl): 3-12
Navarro-Colorado C, Mahamud A, Burton A, Haskew C, Maina GK, Wagacha JB, Ahmed JA, Shetty S, Cookson S, Goodson JL, Schilperoord M, Spiegel P. Measles outbreak response among adolescent and adult Somali refugees displaced by famine in Kenya and Ethiopia, 2011. J Infect Dis. 2014; 210(12): 1863-70
Ndegwa LK, Katz MA, McCormick K, Nganga Z, Mungai A, Emukule G, Kollmann MKHM, Mayieka L, Otieno J, Breiman RF, Mott JA, Ellingson K. Surveillance for respiratory health care-associated infections among inpatients in 3 Kenyan hospitals, 2010-2012. Am J Infect Control. 2014; 42(9): 985–90
Nevill CG, Lury JD, Mosobo MK, Watkins HM, Watkins WM. Daily chlorproguanil is an effective alternative to daily proguanil in the prevention of Plasmodium falciparum malaria in Kenya. Trans R Soc Trop Med Hyg. 1994; 88(3): 319-20 as it appears in Malaria Atlas Project. Malaria Atlas Project Plasmodium Falciparum Parasite Rate Database. Oxford, United Kingdom: Malaria Atlas Project
Nevill CG, Watkins WM, Carter JY, Munafu CG. Comparison of mosquito nets, proguanil hydrochloride, and placebo to prevent malaria. Br Med J (Clin Res Ed). 1988; 297(6645): 401-3 as it appears in Malaria Atlas Project. Malaria Atlas Project Plasmodium Falciparum Parasite Rate Database. Oxford, United Kingdom: Malaria Atlas Project
Ng'etich AI, Rawago FO, Jura WGZO, Mwinzi PN, Won KY, Odiere MR. A cross-sectional study on schistosomiasis and soil-transmitted helminths in Mbita district, western Kenya using different copromicroscopic techniques. Parasit Vectors. 2016; 9: 87

Njenga SM, Mwandawiro CS, Muniu E, Mwanje MT, Haji FM, Bockarie MJ. Adult population as potential reservoir of NTD infections in rural villages of Kwale district, Coastal Kenya: implications for preventive chemotherapy interventions policy. Parasit Vectors. 2011; 4: 175

Noland GS, Hendel-Paterson B, Min XM, Moormann AM, Vulule JM, Narum DL, Lanar DE, Kazura JW, John CC. Low prevalence of antibodies to preerythrocytic but not blood-stage Plasmodium falciparum antigens in an area of unstable malaria transmission compared to prevalence in an area of stable malaria transmission. Infect Immun. 2008; 76(12): 5721-8 as it appears in Malaria Atlas Project. Malaria Atlas Project Plasmodium Falciparum Parasite Rate Database. Oxford, United Kingdom: Malaria Atlas Project

Odhiambo FO, Beynon CM, Ogwang S, Hamel MJ, Howland O, van Eijk AM, Norton R, Amek N, Slutsker L, Laserson KF, De Cock KM, Phillips-Howard PA. Trauma-Related Mortality among Adults in Rural Western Kenya: Characterising Deaths Using Data from a Health and Demographic Surveillance System. PLoS One. 2013; 8(11): e79840

Odiere MR, Rawago FO, Ombok M, Secor WE, Karanja DM, Mwinzi PN, Lammie PJ, Won K. High prevalence of schistosomiasis in Mbita and its adjacent islands of Lake Victoria, western Kenya. Parasit Vectors. 2012; 2: 278

Ofulla AV, Moormann AM, Embury PE, Kazura JW, Sumba PO, John CC. Age-related differences in the detection of Plasmodium falciparum infection by PCR and microscopy, in an area of Kenya with holo-endemic malaria. Ann Trop Med Parasitol. 2005; 99(4): 431-5 as it appears in Malaria Atlas Project. Malaria Atlas Project Plasmodium Falciparum Parasite Rate Database. Oxford, United Kingdom: Malaria Atlas Project

Ogutu BR, Apollo OJ, McKinney D, Okoth W, Siangla J, Dubovsky F, Tucker K, Waitumbi JN, Diggs C, Wittes J, Malkin E, Leach A, Soisson LA, Milman JB, Otieno L, Holland CA, Polhemus M, Remich SA, Ockenhouse CF, Cohen J, Ballou WR, Martin SK, Angov E, Stewart VA, Lyon JA, Heppner DG Jr, Withers MR, for the MSP-1 Malaria Vaccine Working Group. Blood Stage Malaria Vaccine Eliciting High Antigen-Specific Antibody Concentrations Confers No Protection to Young Children in Western Kenya. PLoS One. 2009; 4(3): e4708 as it appears in Malaria Atlas Project. Malaria Atlas Project Plasmodium Falciparum Parasite Rate Database. Oxford, United Kingdom: Malaria Atlas Project

Okech BA, Mwobobia IK, Kamau A, Muiruri S, Mutiso N, Nyambura J, Mwatele C, Amano T, Mwandawiro CS. Use of Integrated Malaria Management Reduces Malaria in Kenya. PLoS One. 2008; 3(12): e4050 as it appears in Malaria Atlas Project. Malaria Atlas Project Plasmodium Falciparum Parasite Rate Database. Oxford, United Kingdom: Malaria Atlas Project

Okoth F, Mbuthia J, Gatheru Z, Murila F, Kanyingi F, Mugo F, Esamai F, Alavi Z, Otieno J, Kiambati H, Wanjuki N. Seroprevalence of hepatitis B markers in pregnant women in Kenya. East Afr Med J. 2006; 83(9): 485-93

Okoth FA, Kobayashi M, Kaptich DC, Kaiguri PM, Tukei PM, Takayanagi T, Yamanaka T. Seroepidemiological study for HBV markers and anti-delta in Kenya. East Afr Med J. 1991; 68(7): 515-25

Olds GR, King C, Hewlett J, Olveda R, Wu G, Ouma J, Peters P, McGarvey S, Odhiambo O, Koech D, Liu CY, Aligui G, Gachihi G, Kombe Y, Parraga I, Ramirez B, Whalen C, Horton RJ, Reeve P. Double-blind placebo-controlled study of concurrent administration of albendazole and praziquantel in schoolchildren with schistosomiasis and geohelminths. J Infect Dis. 1999; 179(4): 996-1003

Oloo A, Githeko A, Adungo N, Karanja D, Vulule J, Kisia-Abok I, Seroney I, Ayisi J, Ondijo S, Koech DK, Abdullah MS. Field trial of permethrin impregnated sisal curtains in malaria control in western Kenya. East Afr Med J. 1996; 73(11): 735-40 as it appears in Malaria Atlas Project.

Malaria Atlas Project Plasmodium Falciparum Parasite Rate Database. Oxford, United Kingdom: Malaria Atlas Project
Olsen A. The proportion of helminth infections in a community in western Kenya which would be treated by mass chemotherapy of schoolchildren. Trans R Soc Trop Med Hyg. 1998; 92(2): 144-8
Onyango CO, Njeru R, Kazungu S, Achilla R, Bulimo W, Welch SR, Cane PA, Gunson RN, Hammitt LL, Scott JAG, Berkley JA, Nokes DJ. Influenza surveillance among children with pneumonia admitted to a district hospital in coastal Kenya, 2007-2010. J Infect Dis. 2012; 206(Suppl 1): S61-67
O'Reilly CE, Jaron P, Ochieng B, Nyaguara A, Tate JE, Parsons MB, Bopp CA, Williams KA, Vinjé J, Blanton E, Wannemuehler KA, Vulule J, Laserson KF, Breiman RF, Feikin DR, Widdowson M-A, Mintz E. Risk factors for death among children less than 5 years old hospitalized with diarrhea in rural western Kenya, 2005-2007: a cohort study. PLoS Med. 2012; 9(7): e1001256
Oti SO, Kyobutungi C. Verbal autopsy interpretation: a comparative analysis of the InterVA model versus physician review in determining causes of death in the Nairobi DSS. Popul Health Metr. 2010; 8: 21
Ouma JH, Waithaka F. Prevalence of Schistosoma mansoni and Schistosoma haematobium in Kitui District, Kenya. East Afr Med J. 1978; 55(2): 54-60
Outbreak of Epidemic Malaria in Uasin Gishu District-1988 as it appears in Malaria Atlas Project. Malaria Atlas Project Plasmodium Falciparum Parasite Rate Database. Oxford, United Kingdom: Malaria Atlas Project
Oyieke JBO, Obore S, Kigondu CS. Millennium development goal 5: a review of maternal mortality at the Kenyatta National Hospital, Nairobi. East Afr Med J. 2006; 83(1): 4-9
Pinoges L, Schramm B, Poulet E, Balkan S, Szumilin E, Ferreyra C, Pujades-Rodríguez M. Risk factors and mortality associated with resistance to first-line antiretroviral therapy: multicentric cross-sectional and longitudinal analyses. J Acquir Immune Defic Syndr. 2015; 68(5): 527–35
Porter K, Zaba B. The empirical evidence for the impact of HIV on adult mortality in the developing world: data from serological studies. AIDS. 2004; 18(Suppl 2): S9-S17
Prevalence of Concomitant Infections of Plasmodium falciparum and Wuchereria bancrofti in Mosquito and Human Populations in Malindi, Kenya as it appears in Malaria Atlas Project. Malaria Atlas Project Plasmodium Falciparum Parasite Rate Database. Oxford, United Kingdom: Malaria Atlas Project
Radin JM, Katz MA, Tempia S, Talla Nzussouo N, Davis R, Duque J, Adedeji A, Adjabeng MJ, Ampofo WK, Ayele W, Bakamutumaho B, Barakat A, Cohen AL, Cohen C, Dalhatu IT, Daouda C, Dueger E, Francisco M, Heraud J-M, Jima D, Kabanda A, Kadjo H, Kandeel A, Bi Shamamba SK, Kasolo F, Kronmann KC, Mazaba Liwewe ML, Lutwama JJ, Matonya M, Mmbaga V, Mott JA, Muhimpundu MA, Muthoka P, Njuguna H, Randrianasolo L, Refaey S, Sanders C, Talaat M, Theo A, Valente F, Venter M, Woodfill C, Bresee J, Moen A, Widdowson M-A. Influenza surveillance in 15 countries in Africa, 2006-2010. J Infect Dis. 2012; S14–21
Reniers G, Slaymaker E, Nakiyingi-Miiro J, Nyamukapa C, Crampin AC, Herbst K, Urassa M, Otieno F, Gregson S, Sewe M, Michael D, Lutalo T, Hosegood V, Kasamba I, Price A, Nabukalu D, Mclean E, Zaba B, ALPHA Network. Mortality trends in the era of antiretroviral therapy: evidence from the Network for Analysing Longitudinal Population based HIV/AIDS data on Africa (ALPHA). AIDS. 2014; S533–42
Report on the Malaria Prevalence Survey in Malindi as it appears in Malaria Atlas Project. Malaria Atlas Project Plasmodium Falciparum Parasite Rate Database. Oxford, United Kingdom: Malaria Atlas Project

Rijpstra AC. Results of duplicated series of stool-examinations for all intestinal parasites by five different methods in school-children in East Africa with remarks on serological aspects of amoebiasis and schistosomiasis. Ann Soc Belg Med Trop. 1975; 55(5): 415-25

Rowe AK, Rowe SY, Snow RW, Korenromp EL, Schellenberg JR, Stein C, Nahlen BL, Bryce J, Black RE, Steketee RW. The burden of malaria mortality among African children in the year 2000. Int J Epidemiol. 2006; 35(3): 691-704

Rowland M, Bouma M, Ducornez D, Durrani N, Rozendaal J, Schapira A, Sondorp E. Pyrethroid-impregnated bed nets for personal protection against malaria for Afghan refugees. Trans R Soc Trop Med Hyg. 1996; 90(4): 357-61 as it appears in Malaria Atlas Project. Malaria Atlas Project Plasmodium Falciparum Parasite Rate Database. Oxford, United Kingdom: Malaria Atlas Project

Ruto JJ, Karuga JW. Temporal and spatial epidemiology of sleeping sickness and use of geographical information system (GIS) in Kenya. J Vector Borne Dis. 2009; 46(1): 18-25

Saidi SM, Iijima Y, Sang WK, Mwangudza AK, Oundo JO, Taga K, Aihara M, Nagayama K, Yamamoto H, Waiyaki PG, Honda T. Epidemiological study on infectious diarrheal diseases in children in a coastal rural area of Kenya. Microbiol Immunol. 1997; 41(10): 773-8

Salehyan I, Hendrix CS, Hamner J, Case C, Linebarger C, Stull E, Williams J, Robert S. Strauss Center for International Security and Law. Social Conflict in Africa: A New Database. Int Interact. 2012; 38(4): 503-511

Sang WK, Oundo V, Schnabel D. Prevalence and antibiotic resistance of bacterial pathogens isolated from childhood diarrhoea in four provinces of Kenya. J Infect Dev Ctries. 2012; 6(7): 572–8

Satayathum SA, Muchiri EM, Ouma JH, Whalen CC, King CH. Factors affecting infection or reinfection with Schistosoma haematobium in coastal Kenya: survival analysis during a nine-year, school-based treatment program. Am J Trop Med Hyg. 2006; 75(1): 83-92

Sato K, Shimada M, Noda S, Muhoho ND, Katsumata T, Sato A, Aoki Y. Efficacy of metrifonate in a highly endemic area of urinary schistosomiasis in Kenya. Am J Trop Med Hyg. 1988; 38(1): 81-5

Schaefer KU, Khan B, Gachihi GS, Kager PA, Muller AS, Verhave JP, Mcneill KM. Splenomegaly in Baringo District, Kenya, an area endemic for visceral leishmaniasis and malaria. Trop Geogr Med. 1995; 47(3): 111–4 as it appears in Malaria Atlas Project. Malaria Atlas Project Plasmodium Falciparum Parasite Rate Database. Oxford, United Kingdom: Malaria Atlas Project

Seal A, Creeke P, Mirghani Z, Abdalla F, McBurney R, Pratt L, Brookes D, Ruth L, Marchand E. Iron and vitamin A deficiency in long-term African refugees. J Nutr. 2005; 135(4): 808-13 as it appears in Malaria Atlas Project. Malaria Atlas Project Plasmodium Falciparum Parasite Rate Database. Oxford, United Kingdom: Malaria Atlas Project

Sexton JD, Ruebush TK, Brandling-Bennett AD, Breman JG, Roberts JM, Odera JS, Were JB. Permethrin-impregnated curtains and bed-nets prevent malaria in western Kenya. Am J Trop Med Hyg. 1990; 43(1): 11-8 as it appears in Malaria Atlas Project. Malaria Atlas Project Plasmodium Falciparum Parasite Rate Database. Oxford, United Kingdom: Malaria Atlas Project

Shanks GD, Biomndo K, Guyatt HL, Snow RW. Travel as a risk factor for uncomplicated Plasmodium falciparum malaria in the highlands of western Kenya. Trans R Soc Trop Med Hyg. 2005; 99(1): 71-4 as it appears in Malaria Atlas Project. Malaria Atlas Project Plasmodium Falciparum Parasite Rate Database. Oxford, United Kingdom: Malaria Atlas Project

Shapiro RL, Otieno MR, Adcock PM, Phillips-Howard PA, Hawley WA, Kumar L, Waiyaki P, Nahlen BL, Slutsker L. Transmission of epidemic Vibrio cholerae O1 in rural western Kenya associated

with drinking water from Lake Victoria: an environmental reservoir for cholera?. Am J Trop Med Hyg. 1999; 60(2): 271–6

Shililu, Maier, Seitz, Orago. Seasonal density, sporozoite rates and entomological inoculation rates of Anopheles gambiae and Anopheles funestus in a high-altitude sugarcane growing zone in western kenya. Trop Med Int Health. 1998; 3(9): 706–10 as it appears in Malaria Atlas Project. Malaria Atlas Project Plasmodium Falciparum Parasite Rate Database. Oxford, United Kingdom: Malaria Atlas Project

Shimada M, Hirata M, Ouma JH, Wambayi E, Thiongo FW, Aoki Y. Epidemiological study of Schistosoma haematobium infection in the coastal area of Kenya. Japan J Trop Med Hyg. 1987; 15(3): 173-84

Slutsker L, Tipple M, Keane V, McCance C, Campbell CC. Malaria in east African refugees resettling to the United States: development of strategies to reduce the risk of imported malaria. J Infect Dis. 1995; 171(2): 489-93 as it appears in Malaria Atlas Project. Malaria Atlas Project Plasmodium Falciparum Parasite Rate Database. Oxford, United Kingdom: Malaria Atlas Project

Smith DH, Warren KS, Mahmoud AA. Morbidity in schistosomiasis mansoni in relation to intensity of infection: study of a community in Kisumu, Kenya. Am J Trop Med Hyg. 1979; 28(2): 220-9

Snow RW, Omumbo JA, Lowe B, Molyneux CS, Obiero JO, Palmer A, Weber MW, Pinder M, Nahlen B, Obonyo C, Newbold C, Gupta S, Marsh K. Relation between severe malaria morbidity in children and level of Plasmodium falciparum transmission in Africa. Lancet. 1997; 349(9066): 1650-4 as it appears in Malaria Atlas Project. Malaria Atlas Project Plasmodium Falciparum Parasite Rate Database. Oxford, United Kingdom: Malaria Atlas Project

Spencer HC, Kaseje DC, Collins WE, Shehata MG, Turner A, Stanfill PS, Huong AY, Roberts JM, Villinski M, Koech DK. Community-based malaria control in Saradidi, Kenya: description of the programme and impact on parasitaemia rates and antimalarial antibodies. Ann Trop Med Parasitol. 1987; 81 Suppl 1: 13–23 as it appears in Malaria Atlas Project. Malaria Atlas Project Plasmodium Falciparum Parasite Rate Database. Oxford, United Kingdom: Malaria Atlas Project

Stephenson LS, Latham MC, Crompton DWT, Schulpen TWJ, Jansen AA. Nutritional status and stool examinations for intestinal parasites in Kenyan preschool children in Machakos District. East Afr Med J. 1979; 56(1): 1-9

Stephenson LS, Latham MC, Kinoti SN, Oduori ML. Regression of Splenomegaly and Hepatomegaly in Children Treated for Schistosoma haematobium Infection. Am J Trop Med Hyg. 1985; 34(1): 119–23 as it appears in Malaria Atlas Project. Malaria Atlas Project Plasmodium Falciparum Parasite Rate Database. Oxford, United Kingdom: Malaria Atlas Project

Stevenson JC, Stresman GH, Gitonga CW, Gillig J, Owaga C, Marube E, Odongo W, Okoth A, China P, Oriango R, Brooker SJ, Bousema T, Drakeley C, Cox J. Reliability of School Surveys in Estimating Geographic Variation in Malaria Transmission in the Western Kenyan Highlands. PLoS One. 2013; 8(10): e77641 as it appears in Malaria Atlas Project. Malaria Atlas Project Plasmodium Falciparum Parasite Rate Database. Oxford, United Kingdom: Malaria Atlas Project

Studies on Malaria and its Vectors in Nairobi: a Review of the Distribution of the Vectors and the Prevalence of the Disease as it appears in Malaria Atlas Project. Malaria Atlas Project Plasmodium Falciparum Parasite Rate Database. Oxford, United Kingdom: Malaria Atlas Project

Sturrock RF, Kariuki HC, Thiongo FW, Gachare JW, Omondi BG, Ouma JH, Mbugua G, Butterworth AE. Schistosomiasis mansoni in Kenya: relationship between infection and anaemia in schoolchildren at the community level. Trans R Soc Trop Med Hyg. 1996; 90(1): 48-54

Suchdev PS, Davis SM, Bartoces M, Ruth LJ, Worrell CM, Kanyi H, Odero K, Wiegand RE, Njenga SM, Montgomery JM, Fox LM. Soil-transmitted helminth infection and nutritional status among urban slum children in Kenya. Am J Trop Med Hyg. 2014; 90(2): 229-305 as it appears in Malaria Atlas Project. Malaria Atlas Project Plasmodium Falciparum Parasite Rate Database. Oxford, United Kingdom: Malaria Atlas Project

Swierczewski BE, Odundo EA, Koech MC, Ndonye JN, Kirera RK, Odhiambo CP, Cheruiyot EK, Shaffer DN, Ombogo AN, Oaks EV. Enteric pathogen surveillance in a case-control study of acute diarrhoea in the town of Kisii, Kenya. J Med Microbiol. 2013; 62(Pt 11): 1774-6

Talbert A, Thuo N, Karisa J, Chesaro C, Ohuma E, Ignas J, Berkley JA, Toromo C, Atkinson S, Maitland K. Diarrhoea complicating severe acute malnutrition in Kenyan children: a prospective descriptive study of risk factors and outcome. PLoS One. 2012; 7(6): e38321

The Malaria Parasite Prevalence Rates in Settled Villages of the Mwea Tebere Irrigation Scheme as it appears in Malaria Atlas Project. Malaria Atlas Project Plasmodium Falciparum Parasite Rate Database. Oxford, United Kingdom: Malaria Atlas Project

Ukachukwu VE, Unger H, Onoka C, Nduka C, Maina S, Ngugi N. Maternal morbidity and mortality in peri-urban Kenya--assessing progress in improving maternal healthcare. East Afr J Public Health. 2009; 6(2): 112-8

United Nations Children's Fund (UNICEF), World Health Organization (WHO). WHO and UNICEF Reported Disease Incidence Time Series. Geneva, Switzerland: World Health Organization (WHO)

United Nations Office on Drugs and Crime (UNODC). United Nations Office on Drugs and Crime Global Study on Homicide 2011. Vienna, Austria: United Nations Office on Drugs and Crime (UNODC), 2011

van Eijk AM, Adazu K, Ofware P, Vulule J, Hamel M, Slutsker L. Causes of deaths using verbal autopsy among adolescents and adults in rural western Kenya. Trop Med Int Health. 2008; 13(10): 1314-24

Veenemans J, Andang'O PEA, Mbugi EV, Kraaijenhagen RJ, Mwaniki DL, Mockenhaupt FP, Roewer S, Olomi RM, Shao JF, Van Der Meer JWM, Savelkoul HFJ, Verhoef H. Alpha+ -thalassemia protects against anemia associated with asymptomatic malaria: evidence from community-based surveys in Tanzania and Kenya. J Infect Dis. 2008; 198(3): 401–8 as it appears in Malaria Atlas Project. Malaria Atlas Project Plasmodium Falciparum Parasite Rate Database. Oxford, United Kingdom: Malaria Atlas Project

Verani JR, Abudho B, Montgomery SP, Mwinzi PNM, Shane HL, Butler SE, Karanja DMS, Secor WE. Schistosomiasis among young children in Usoma, Kenya. Am J Trop Med Hyg. 2011; 84(5): 787–91

Verhoef H, Hodgins E, Eggelte TA, Carter JY, Lema O, West CE, Kok FJ. Anti-malarial drug use among preschool children in an area of seasonal malaria transmission in Kenya. Am J Trop Med Hyg. 1999; 61(5): 770–5 as it appears in Malaria Atlas Project. Malaria Atlas Project Plasmodium Falciparum Parasite Rate Database. Oxford, United Kingdom: Malaria Atlas Project

Voorhoeve AM, Muller AS, Schulpen TW, t' Mannetje W, van Rens M. Machakos project studies. Agents affecting health of mother and child in a rural area of Kenya. IV: Theepidemiology of pertussis. Trop Geogr Med. 1978; 30(1): 125-39

Wamae CN, Lammie PJ. Haematuria in coastal Kenya is associated with Schistosoma haematobium but not Wuchereria bancrofti infection. Trans R Soc Trop Med Hyg. 1998; 92(1): 63-4

Wamala JF, Malimbo M, Okot CL, Atai-Omoruto AD, Tenywa E, Miller JR, Balinandi S, Shoemaker T, Oyoo C, Omony EO, Kagirita A, Musenero MM, Makumbi I, Nanyunja M, Lutwama JJ, Downing R, Mbonye AK. Epidemiological and Laboratory Characterization of a Yellow Fever

Outbreak in Northern Uganda, October 2010–January 2011. Int J Infect Dis. 2012; 16(7): e536-42
Wanjala CL, Waitumbi J, Zhou G, Githeko AK. Identification of malaria transmission and epidemic hotspots in the western Kenya highlands: its application to malaria epidemic prediction. Parasit Vectors. 2011; 4: 81 as it appears in Malaria Atlas Project. Malaria Atlas Project Plasmodium Falciparum Parasite Rate Database. Oxford, United Kingdom: Malaria Atlas Project
Warren KS, Mahmoud AA, Muruka JF, Whittaker LR, Ouma JH, Arap Siongok TK. Schistosomiasis haematobia in coast province Kenya. Am J Trop Med Hyg. 1979; 28(5): 864-70
Watkins WM, Howells RE, Brandling-Bennett AD, Koech DK. In Vitro Susceptibility of Plasmodium falciparum Isolates from Jilore, Kenya, to Antimalarial Drugs. Am J Trop Med Hyg. 1987; 37(3): 445–51 as it appears in Malaria Atlas Project. Malaria Atlas Project Plasmodium Falciparum Parasite Rate Database. Oxford, United Kingdom: Malaria Atlas Project
Watkins WM, Oloo JA, Lury JD, Mosoba M, Kariuki D, Mjomba M, Koech DK, Gilles HM. Efficacy of multiple-dose halofantrine in treatment of chloroquine-resistant falciparum malaria in children in Kenya. Lancet. 1988; 2(8605): 247-50 as it appears in Malaria Atlas Project. Malaria Atlas Project Plasmodium Falciparum Parasite Rate Database. Oxford, United Kingdom: Malaria Atlas Project
WHO Department of Communicable Disease Surveillance and Response. WHO Report on Global Surveillance of Epidemic-prone Infectious Diseases 2000
Whooping Cough Case Fatality Rate Estimates as provided by the Global Burden of Disease 2010 Pertussis Expert Group. [Unpublished]
Wikipedia. 2015 Mina Stampede. San Francisco, United States: Wikipedia; [updated 2016]
Wikipedia. List of Terrorist Incidents in 2016. San Francisco, United States: Wikipedia
Wilson S, Booth M, Jones FM, Mwatha JK, Kimani G, Kariuki HC, Vennervald BJ, Ouma JH, Muchiri E, Dunne DW. Age-adjusted Plasmodium falciparum antibody levels in school-aged children are a stable marker of microgeographical variations in exposure to Plasmodium infection. BMC Infect Dis. 2007; 7(1): 67 as it appears in Malaria Atlas Project. Malaria Atlas Project Plasmodium Falciparum Parasite Rate Database. Oxford, United Kingdom: Malaria Atlas Project
Wilson S, Vennervald BJ, Kadzo H, Ireri E, Amaganga C, Booth M, Kariuki HC, Mwatha JK, Kimani G, Ouma JH, Muchiri E, Dunne DW. Hepatosplenomegaly in Kenyan schoolchildren: exacerbation by concurrent chronic exposure to malaria and Schistosoma mansoni infection. Trop Med Int Health. 2007; 12(12): 1442-9 as it appears in Malaria Atlas Project. Malaria Atlas Project Plasmodium Falciparum Parasite Rate Database. Oxford, United Kingdom: Malaria Atlas Project
World Health Organization (WHO). Kenya World Health Survey 2004. Geneva, Switzerland: World Health Organization (WHO), 2005
World Health Organization (WHO). WHO Global Health Observatory - Cholera: Number of Reported Cases by Country. Geneva, Switzerland: World Health Organization (WHO)
World Health Organization (WHO). WHO Global Health Observatory - Number of new reported cases (T.b. rhodesiense), Data by country. Geneva, Switzerland: World Health Organization (WHO)
World Health Organization (WHO). WHO Global Project on Anti-Tuberculosis Drug Resistance Surveillance Data 1988-2015
World Health Organization (WHO). WHO Tuberculosis Case Notifications. Geneva, Switzerland: World Health Organization (WHO)
World Malaria Report 2005 as it appears in Malaria Atlas Project. Malaria Atlas Project Interventions Database

World Malaria Report 2008 as it appears in Malaria Atlas Project. Malaria Atlas Project Interventions Database
World Malaria Report 2009 as it appears in Malaria Atlas Project. Malaria Atlas Project Interventions Database
World Malaria Report 2012 as it appears in Malaria Atlas Project. Malaria Atlas Project Interventions Database
World Malaria Report 2013 as it appears in Malaria Atlas Project. Malaria Atlas Project Annual Parasite Incidence Database
World Malaria Report 2015 as it appears in Malaria Atlas Project. Malaria Atlas Project Annual Parasite Incidence Database
World Malaria Report 2016 as it appears in Malaria Atlas Project. Malaria Atlas Project Annual Parasite Incidence Database
Ye Y, Madise N, Ndugwa R, Ochola S, Snow RW. Fever treatment in the absence of malaria transmission in an urban informal settlement in Nairobi, Kenya. Malar J. 2009; 8(1): 160 as it appears in Malaria Atlas Project. Malaria Atlas Project Plasmodium Falciparum Parasite Rate Database. Oxford, United Kingdom: Malaria Atlas Project

Appendix Table 3. Data used in Kenya morbidity estimation, GBD 2016

Abt Associates Inc., Kenya National Bureau of Statistics, Ministry of Health (Kenya). Kenya Household Health Expenditure and Utilization Survey 2007. Nairobi, Kenya: Kenya National Bureau of Statistics

Abu-Aisha H, Elamin S. Peritoneal dialysis in Africa. Perit Dial Int. 2010; 30(1): 23–8

Action Africa Help International (AAH-I), Institute for Health Metrics and Evaluation (IHME), Ministry of Medical Services (Kenya), Ministry of Public Health and Sanitation (Kenya). Access, Bottlenecks, Costs, and Equity (ABCE) project in Kenya, 2012. Seattle, United States: Institute for Health Metrics and Evaluation (IHME), 2015

Adam AM. Multiple sclerosis: epidemic in Kenya. East Afr Med J. 1989; 66(8): 503-6

Adaptive Integrated Malaria Vector Management at Malindi, Kenya--Final Report (May 2005-April 2006) as it appears in Malaria Atlas Project. Malaria Atlas Project Plasmodium Falciparum Parasite Rate Database. Oxford, United Kingdom: Malaria Atlas Project

Aidoo M, Terlouw DJ, Kolczak MS, McElroy PD, ter Kuile FO, Kariuki S, Nahlen BL, Lal AA, Udhayakumar V. Protective effects of the sickle cell gene against malaria morbidity and mortality. Lancet. 2002; 359(9314): 1311-2

Akhwale WS, Lum JK, Kaneko A, Eto H, Obonyo C, Björkman A, Kobayakawa T. Anemia and malaria at different altitudes in the western highlands of Kenya. Addict Behav Rep. 2004; 91(2): 167-75

Allison AC. Glucose-6-phosphate dehydrogenase deficiency in red blood cells of East Africans. Nature. 1960; 531-2

Amollo DA, Kihara JH, Kombe Y, Karanja SM. PREVALENCE AND INTENSITY OF SINGLE AND MIXED SCHISTOSOMA MANSONI AND SCHISTOSOMA HAEMATOBIUM INFECTIONS IN PRIMARY SCHOOL CHILDREN IN RACHUONYO NORTH DISTRICT, HOMABAY COUNTY, WESTERN KENYA. East Afr Med J. 2013; 90(2): 36–44

Amornkul PN, Vandenhoudt H, Nasokho P, Odhiambo F, Mwaengo D, Hightower A, Buvé A, Misore A, Vulule J, Vitek C, Glynn J, Greenberg A, Slutsker L, De Cock KM. HIV prevalence and associated risk factors among individuals aged 13-34 years in Rural Western Kenya. PLoS One. 2009; 4(7): e6470

Anabwani GM, Bonhoeffer P. Prevalence of heart disease in school children in rural Kenya using colour-flow echocardiography. East Afr Med J. 1996; 73(4): 215-7

Annual Report for Dertu, Kenya, Millennium Village. Year 1: February 2006-February 2007 as it appears in Malaria Atlas Project. Malaria Atlas Project Plasmodium Falciparum Parasite Rate Database. Oxford, United Kingdom: Malaria Atlas Project

Arap Siongok TK, Mahmoud AA, Ouma JH, Warren KS, Muller AS, Handa AK, Houser HB. Morbidity in Schistosomiasis mansoni in relation to intensity of infection: study of a community in Machakos, Kenya. Am J Trop Med Hyg. 1976; 25(2): 273-84

Armah GE, Sow SO, Breiman RF, Dallas MJ, Tapia MD, Feikin DR, Binka FN, Steele AD, Laserson KF, Ansah NA, Levine MM, Lewis K, Coia ML, Attah-Poku M, Ojwando J, Rivers SB, Victor JC, Nyambane G, Hodgson A, Schödel F, Ciarlet M, Neuzil KM. Efficacy of pentavalent rotavirus vaccine against severe rotavirus gastroenteritis in infants in developing countries in sub-Saharan Africa: a randomised, double-blind, placebo-controlled trial. Lancet. 2010; 376(9741): 606–14

Ashford R, Craig P, Oppenheimer S. Polyparasitism on the Kenya coast. 2. Spatial heterogeneity in parasite distributions. Ann Trop Med Parasitol. 1993; 87(3): 283-93 as it appears in Malaria Atlas Project. Malaria Atlas Project Plasmodium Falciparum Parasite Rate Database. Oxford, United Kingdom: Malaria Atlas Project

Ashford RW, Craig PS, Oppenheimer SJ. Polyparasitism on the Kenya coast. 1. Prevalence, and association between parasitic infections. Ann Trop Med Parasitol. 1992; 86(6): 671-9 as it appears in Malaria Atlas Project. Malaria Atlas Project Plasmodium Falciparum Parasite Rate Database. Oxford, United Kingdom: Malaria Atlas Project

Ayisi JG, van Eijk AM, ter Kuile FO, Kolczak MS, Otieno JA, Misore AO, Kager PA, Steketee RW, Nahlen BL. The effect of dual infection with HIV and malaria on pregnancy outcome in western Kenya. AIDS. 2003; 17(4): 585-94

Baelum V, Manji F, Fejerskov O, Wanzala P. Validity of CPITN's assumptions of hierarchical occurrence of periodontal conditions in a Kenyan population aged 15-65 years. Community Dent Oral Epidemiol. 1993; 21(6): 347-53

Baeten JM, McClelland RS, Corey L, Overbaugh J, Lavreys L, Richardson BA, Wald A, Mandaliya K, Bwayo JJ, Kreiss JK. Vitamin A supplementation and genital shedding of herpes simplex virus among HIV-1-infected women: a randomized clinical trial. J Infect Dis. 2004; 189(8): 1466-71

Baliraine FN, Afrane YA, Amenya DA, Bonizzoni M, Menge DM, Zhou G, Zhong D, Vardo-Zalik AM, Githeko AK, Yan G. High Prevalence of Asymptomatic Plasmodium falciparum Infections in a Highland Area of Western Kenya: A Cohort Study. J Infect Dis. 2009; 200(1): 66-74 as it appears in Malaria Atlas Project. Malaria Atlas Project Plasmodium Falciparum Parasite Rate Database. Oxford, United Kingdom: Malaria Atlas Project

Baseline Report: Millenium Research Village Sauri, Kenya as it appears in Malaria Atlas Project. Malaria Atlas Project Plasmodium Falciparum Parasite Rate Database. Oxford, United Kingdom: Malaria Atlas Project

Beatty ME, Ochieng JB, Chege W, Kumar L, Okoth G, Shapiro RL, Wells JG, Parsons MB, Bopp C, Chiller T, Vulule J, Mintz E, Slutsker L, Brooks JT. Sporadic paediatric diarrhoeal illness in urban and rural sites in Nyanza Province, Kenya. East Afr Med J. 2009; 86(8): 387-98

Beier J, Oster C, Onyango F, Bales J, Sherwood J, Perkins P, Chumo D, Koech dv, Whitmire R, Roberts C. Plasmodium falciparum incidence relative to entomologic inoculation rates at a site proposed for testing malaria vaccines in western Kenya. Am J Trop Med Hyg. 1994; 50(5): 529-36 as it appears in Malaria Atlas Project. Malaria Atlas Project Plasmodium Falciparum Parasite Rate Database. Oxford, United Kingdom: Malaria Atlas Project

Bejon P, Lusingu J, Olotu A, Leach A, Lievens M, Vekemans J, Mshamu S, Lang T, Gould J, Dubois MC, Demoitié MA, Stallaert JF, Vansadia P, Carter T, Njuguna P, Awuondo KO, Malabeja A, Abdul O, Gesase S, Mturi N, Drakeley CJ, Savarese B, Villafana T, Ballou WR, Cohen J, Riley EM, Lemnge MM, Marsh K, von Seidlein L. Efficacy of RTS,S/AS01E Vaccine against Malaria in Children 5 to 17 Months of Age. N Engl J Med. 2008; 359(24): 2521-32 as it appears in Malaria Atlas Project. Malaria Atlas Project Plasmodium Falciparum Parasite Rate Database. Oxford, United Kingdom: Malaria Atlas Project

Bejon P, Mwacharo J, Kai O, Mwangi T, Milligan P, Todryk S, Keating S, Lang T, Lowe B, Gikonyo C, Molyneux C, Fegan G, Gilbert SC, Peshu N, Marsh K, Hill AVS. A Phase 2b Randomised Trial of the Candidate Malaria Vaccines FP9 ME-TRAP and MVA ME-TRAP among Children in Kenya. PLoS Clin Trials. 2006; 1(6): e29 as it appears in Malaria Atlas Project. Malaria Atlas Project Plasmodium Falciparum Parasite Rate Database. Oxford, United Kingdom: Malaria Atlas Project

Bellizzi S, Ali MM, Abalos E, Betran AP, Kapila J, Pileggi-Castro C, Vogel JP, Merialdi M. Are hypertensive disorders in pregnancy associated with congenital malformations in offspring? Evidence from the WHO Multicountry cross sectional survey on maternal and newborn health. BMC Pregnancy Childbirth. 2016; 16(1): 198

Berkley JA, Munywoki P, Ngama M, Kazungu S, Abwao J, Bett A, Lassauniére R, Kresfelder T, Cane PA, Venter M, Scott JAG, Nokes DJ. Viral etiology of severe pneumonia among Kenyan infants and children. JAMA. 2010; 303(20): 2051-7

Bhutani VK, Zipursky A, Blencowe H, Khanna R, Sgro M, Ebbesen F, Bell J, Mori R, Slusher TM, Fahmy N, Paul VK, Du L, Okolo AA, de Almeida MF, Olusanya BO, Kumar P, Cousens S, Lawn JE. Neonatal hyperbilirubinemia and Rhesus disease of the newborn: incidence and impairment estimates for 2010 at regional and global levels. Pediatr Res. 2013; 74(Suppl 1): 86-100

Blaylock JM, Maranich A, Bauer K, Nyakoe N, Waitumbi J, Martinez LJ, Lynch J. The seroprevalence and seroincidence of dengue virus infection in western Kenya. Travel Med Infect Dis. 2011; 9(5): 246-8

Bloland PB, Boriga DA, Ruebush TK, McCormick JB, Roberts JM, Oloo AJ, Hawley W, Lal A, Nahlen B, Campbell CC. Longitudinal cohort study of the epidemiology of malaria infections in an area of intense malaria transmission II. Descriptive epidemiology of malaria infection and disease among children. Am J Trop Med Hyg. 1999; 60(4): 641-8 as it appears in Malaria Atlas Project. Malaria Atlas Project Plasmodium Falciparum Parasite Rate Database. Oxford, United Kingdom: Malaria Atlas Project

Bonizzoni M, Afrane Y, Baliraine FN, Amenya DA, Githeko AK, Yan G. Genetic structure of Plasmodium falciparum populations between lowland and highland sites and antimalarial drug resistance in Western Kenya. Infect Genet Evol. 2009; 9(5): 806-12 as it appears in Malaria Atlas Project. Malaria Atlas Project Plasmodium Falciparum Parasite Rate Database. Oxford, United Kingdom: Malaria Atlas Project

Bosman MC, Swai OB, Kwamanga DO, Agwanda R, Idukitta G, Misljenovic O. National tuberculin survey of Kenya, 1986-1990. Int J Tuberc Lung Dis. 1998; 2(4): 272–80

Bousema JT, Gouagna LC, Drakeley CJ, Meutstege AM, Okech BA, Akim IN, Beier JC, Githure JI, Sauerwein RW. Plasmodium falciparum gametocyte carriage in asymptomatic children in western Kenya. Malar J. 2004; 3: 18 as it appears in Malaria Atlas Project. Malaria Atlas Project Plasmodium Falciparum Parasite Rate Database. Oxford, United Kingdom: Malaria Atlas Project

Bowry TR, Pade J, Omari M, Chemtai A. A pilot study of hepatitis B virus seroepidemiology suggests widespread immunosuppression in the nomadic inhabitants of Turkana District of Kenya. East Afr Med J. 1985; 62(7): 501-6

Breiman RF, Cosmas L, Audi A, Mwiti W, Njuguna H, Bigogo GM, Olack B, Ochieng JB, Wamola N, Montgomery JM, Williamson J, Parashar UD, Burton DC, Tate JE, Feikin DR. Use of population-based surveillance to determine the incidence of rotavirus gastroenteritis in an urban slum and a rural setting in Kenya. Pediatr Infect Dis J. 2014; S54-61

Breiman RF, Cosmas L, Njenga M, Williamson J, Mott JA, Katz MA, Erdman DD, Schneider E, Oberste M, Neatherlin JC, Njuguna H, Ondari DM, Odero K, Okoth GO, Olack B, Wamola N, Montgomery JM, Fields BS, Feikin DR. Severe acute respiratory infection in children in a densely populated urban slum in Kenya, 2007-2011. BMC Infect Dis. 2015; 15: 95

Breiman RF, Cosmas L, Njuguna H, Audi A, Olack B, Ochieng JB, Wamola N, Bigogo GM, Awiti G, Tabu CW, Burke H, Williamson J, Oundo JO, Mintz ED, Feikin DR. Population-based incidence of typhoid fever in an urban informal settlement and a rural area in Kenya: implications for typhoid vaccine use in Africa. PLoS One. 2012; 7(1): e29119

Brinkhof MWG, Dabis F, Myer L, Bangsberg DR, Boulle A, Nash D, Schechter M, Laurent C, Keiser O, May M, Sprinz E, Egger M, Anglaret X, ART-LINC, IeDEA. Early loss of HIV-infected patients on potent antiretroviral therapy programmes in lower-income countries. Bull World Health Organ. 2008; 86(7): 559-67

Brooker S, Hotez PJ, Bundy DAP. Hookworm-related anaemia among pregnant women: a systematic review. PLoS Negl Trop Dis. 2008; 2(9): e291

Brooker S, Miguel EA, Moulin S, Luoba AI, Bundy DAP, Kremer M. Epidemiology of single and multiple species of helminth infections among school children in Busia District, Kenya. East Afr Med J. 2000; 77(3): 157-61

Brooker S, Peshu N, Warn PA, Mosobo M, Guyatt HL, Marsh K, Snow RW. The epidemiology of hookworm infection and its contribution to anaemia among pre-school children on the Kenyan coast. Trans R Soc Trop Med Hyg. 1999; 93(3): 240-6 as it appears in World Health Organization (WHO). WHO Global Database on Anemia, Nutrition Landscape Information System. Geneva, Switzerland: World Health Organization (WHO)

Brooker SJ, Pullan RL, Gitonga CW, Ashton RA, Kolaczinski JH, Kabatereine NB, Snow RW. Plasmodium-Helminth Coinfection and Its Sources of Heterogeneity Across East Africa. J Infect Dis. 2012; 205(5): 841-52

Bustinduy AL, Parraga IM, Thomas C, Mungai PL, Mutuku F, Muchiri EM, Kitron U, King CH. Anemia, impaired growth and exercise intolerence in Kenyan children: the role of schistosomiasis and polyparasitism [Abstract]. In Abstract Book. American Society of Tropical Medicine and Hygiene 59th Annual Meeting; 2010 Nov 3-7; Atlanta, United States. Am J Trop Med Hyg. 2010; 83(Suppl 5): 318

Bustinduy AL, Parraga IM, Thomas CL, Mungai PL, Mutuku F, Muchiri EM, Kitron U, King CH. Impact of polyparasitic infections on anemia and undernutrition among Kenyan children living in a Schistosoma haematobium-endemic area. Am J Trop Med Hyg. 2013; 88(3): 433-40 as it appears in Malaria Atlas Project. Malaria Atlas Project Plasmodium Falciparum Parasite Rate Database. Oxford, United Kingdom: Malaria Atlas Project

Bustinduy AL, Sutherland LJ, Chang-Cojulun A, Malhotra I, DuVall AS, Fairley JK, Mungai PL, Muchiri EM, Mutuku FM, Kitron U, King CH. Age-Stratified Profiles of Serum IL-6, IL-10, and TNF-a Cytokines Among Kenyan Children with Schistosoma haematobium, Plasmodium falciparum, and Other Chronic Parasitic Co-Infections. Am J Trop Med Hyg. 2015; 92(5): 945-51 as it appears in Malaria Atlas Project. Malaria Atlas Project Plasmodium Falciparum Parasite Rate Database. Oxford, United Kingdom: Malaria Atlas Project

Bustinduy AL, Thomas CL, Fiutem JJ, Parraga IM, Mungai PL, Muchiri EM, Mutuku F, Kitron U, King CH. Measuring fitness of Kenyan children with polyparasitic infections using the 20-meter shuttle run test as a morbidity metric. PLoS Negl Trop Dis. 2011; 5(7): e1213

Buvé A, Weiss HA, Laga M, Van Dyck E, Musonda R, Zekeng L, Kahindo M, Anagonou S, Morison L, Robinson NJ, Hayes RJ. The epidemiology of gonorrhoea, chlamydial infection and syphilis in four African cities. AIDS. 2001; S79-88

Buvé A, Weiss HA, Laga M, Van Dyck E, Musonda R, Zekeng L, Kahindo M, Anagonou S, Morison L, Robinson NJ, Hayes RJ, Study Group on Heterogeneity of HIV Epidemics in African Cities. The epidemiology of trichomoniasis in women in four African cities. AIDS. 2001; S89–96

Campbell GH, Collins FH, Brandling-Bennett AD, Schwartz IK, Roberts JM. Age-specific prevalence of antibody to a synthetic peptide of the circumsporozoite protein of Plasmodium falciparum in children from three villages in Kenya. Am J Trop Med Hyg. 1987; 37(2): 220-4 as it appears in Malaria Atlas Project. Malaria Atlas Project Plasmodium Falciparum Parasite Rate Database. Oxford, United Kingdom: Malaria Atlas Project

Carter Center, International Trachoma Initiative, London School of Hygiene and Tropical Medicine. Global Atlas of Trachoma. Decatur, United States: International Trachoma Initiative

Case definitions of clinical malaria under different transmission conditions in Kilifi District, Kenya and Clinical algorithms for malaria diagnosis lack utility among people of different age groups as it appears in Battle KE, Guerra CA, Golding N, Duda KA, Cameron E, Howes RE, Elyazar IRF, Baird

JK, Reiner Jr. RC, Gething PW, Smith DL, Hay SI. Data from: Global database of matched Plasmodium falciparum and P. vivax incidence and prevalence records from 1985–2013. Dryad Digital Repository. 2015

Center for Vaccine Development (Chile), Center for Vaccine Development, University of Maryland, Centers for Disease Control and Prevention (CDC), Department of Medical Microbiology and Immunology, Göteborg University, International Vaccine Institute, Kenya Medical Research Institute (KEMRI), Perry Point Cooperative Studies Program Coordinating Center, U.S. Department of Veterans Affairs, School of Medicine, University of Virginia, University of Chile. Kenya - Nyanza Global Enteric Multicenter Study 2008-2011. Baltimore, MD, United States: Center for Vaccine Development, University of Maryland

Center for Vaccine Development (Chile), Center for Vaccine Development, University of Maryland, Centers for Disease Control and Prevention (CDC), Department of Medical Microbiology and Immunology, Göteborg University, International Vaccine Institute, Kenya Medical Research Institute (KEMRI), Perry Point Cooperative Studies Program Coordinating Center, U.S. Department of Veterans Affairs, School of Medicine, University of Virginia, University of Chile. Kenya - Nyanza Global Enteric Multicenter Study 2011-2012. Baltimore, MD, United States: Center for Vaccine Development, University of Maryland

Centers for Disease Control and Prevention (CDC), Central Bureau of Statistics (Kenya), Macro International, Inc, Ministry of Health (Kenya), National Council for Population and Development (Kenya). Kenya Demographic and Health Survey 2003. Calverton, United States: Macro International, Inc

Centers for Disease Control and Prevention (CDC), ICF Macro, Kenya Medical Research Institute (KEMRI), Kenya National Bureau of Statistics, Measure DHS, Ministry of Public Health and Sanitation (Kenya), Population Services International (PSI), President's Malaria Initiative (PMI), United Nations Children's Fund (UNICEF), Walter Reed Project, World Health Organization (WHO). Kenya Malaria Indicator Survey 2010. Nairobi, Kenya: Kenya National Bureau of Statistics

Centers for Disease Control and Prevention (CDC), ICF Macro, Kenya Medical Research Institute (KEMRI), Kenya National Bureau of Statistics, Ministry of Public Health and Sanitation (Kenya), National AIDS and STI Control Program (Kenya), National Aids Control Council (NACC), National Coordinating Agency for Population and Development (Kenya). Kenya Demographic and Health Survey - Complete Birth History Data

Centers for Disease Control and Prevention (CDC), Kenya Medical Research Institute (KEMRI), Kenya National Bureau of Statistics, Ministry of Public Health and Sanitation (Kenya), National AIDS Control Council (Kenya), National AIDS and STI Control Program (Kenya), National Coordinating Agency for Population and Development (Kenya), National Public Health Laboratory Services, Ministry of Public Health and Sanitation (Kenya), USAID. Kenya AIDS Indicator Survey 2007. Nairobi, Kenya: Kenya National Bureau of Statistics

Centers for Disease Control and Prevention (CDC), Kenya National Bureau of Statistics, United Nations Children's Fund (UNICEF). Kenya Violence Against Children Study 2010. Nairobi, Kenya: Kenya National Bureau of Statistics

Central Bureau of Statistics (Kenya), International Statistical Institute. Kenya World Fertility Survey 1977-1978. Voorburg, Netherlands: International Statistical Institute

Central Bureau of Statistics (Kenya), Macro International, Inc, National Council for Population Development (NCPD). Kenya Demographic and Health Survey 1993. Calverton, United States: Macro International, Inc

Central Bureau of Statistics (Kenya), Macro International, Inc, National Council for Population Development (NCPD). Kenya Demographic and Health Survey 1998. Calverton, United States: Macro International, Inc

Central Bureau of Statistics (Kenya), United Nations Children's Fund (UNICEF). Kenya Multiple Indicator Cluster Survey 2000. New York, United States: United Nations Children's Fund (UNICEF)

Central Bureau of Statistics (Kenya). Kenya Welfare Monitoring Survey III 1997. Nairobi, Kenya: Kenya National Bureau of Statistics

Centre for Research on the Epidemiology of Disasters (CRED). EM-DAT: The OFDA/CRED International Disaster Database. Brussels, Belgium: Catholic University of Leuven

Chang Cojulun A, Bustinduy AL, Sutherland LJ, Mungai PL, Mutuku F, Muchiri E, Kitron U, King CH. Anemia Among Children Exposed to Polyparasitism in Coastal Kenya. Am J Trop Med Hyg. 2015; 93(5): 1099-105 as it appears in London School of Hygiene and Tropical Medicine. Global Atlas of Helminth Infections - Lymphatic Filariasis. London, United Kingdom: London School of Hygiene and Tropical Medicine

Chege W, Pals SL, McLellan-Lemal E, Shinde S, Nyambura M, Otieno FO, Gust DA, Chen RT, Thomas T. Baseline findings of an HIV incidence cohort study to prepare for future HIV prevention clinical trials in Kisumu, Kenya. J Infect Dev Ctries. 2012; 6(12): 870–80

Chepchirchir A, Bii C, Ndinya-Achola JO. Dermatophyte infections in primary school children in Kibera slums of Nairobi. East Afr Med J. 2009; 86(2): 59-68

Cherutich P, Kaiser R, Galbraith J, Williamson J, Shiraishi RW, Ngare C, Mermin J, Marum E, Bunnell R, KAIS Study Group. Lack of knowledge of HIV status a major barrier to HIV prevention, care and treatment efforts in Kenya: results from a nationally representative study. PLoS One. 2012; 7(5): e36797

Chunge RN, Karumba N, Ouma JH, Thiongo FW, Sturrock RF, Butterworth AE. Polyparasitism in two rural communities with endemic Schistosoma mansoni infection in Machakos District, Kenya. J Trop Med Hyg. 1995; 98(6): 440-4

Clarke SE, Brooker S, Njagi JK, Njau E, Estambale B, Muchiri E, Magnussen P. Malaria morbidity among school children living in two areas of contrasting transmission in western Kenya. Am J Trop Med Hyg. 2004; 71(6): 732-8 as it appears in Malaria Atlas Project. Malaria Atlas Project Plasmodium Falciparum Parasite Rate Database. Oxford, United Kingdom: Malaria Atlas Project

Clarke SE, Jukes MCH, Njagi JK, Khasakhala L, Cundill B, Otido J, Crudder C, Estambale BBA, Brooker S. Effect of intermittent preventive treatment of malaria on health and education in schoolchildren: a cluster-randomised, double-blind, placebo-controlled trial. Lancet. 2008; 372(9633): 127-38 as it appears in Malaria Atlas Project. Malaria Atlas Project Plasmodium Falciparum Parasite Rate Database. Oxford, United Kingdom: Malaria Atlas Project

Clennon JA, King CH, Muchiri EM, Kariuki HC, Ouma JH, Mungai P, Kitron U. Spatial patterns of urinary schistosomiasis infection in a highly endemic area of coastal Kenya. Am J Trop Med Hyg. 2004; 70(4): 443-8

Clinical Epidemiology of Malaria Under Differing Levels of Transmission as it appears in Malaria Atlas Project. Malaria Atlas Project Plasmodium Falciparum Parasite Rate Database. Oxford, United Kingdom: Malaria Atlas Project

Coles GC, Mutahi WT, Kinoti GK, Bruce JI, Katz N. Tolerance of Kenyan Schistosoma mansoni to oxamniquine. Trans R Soc Trop Med Hyg. 1987; 81(5): 782-5

Community-Based Management of Acute Malnutrition (CMAM) Forum. Putting Child Kwarshiorkor on the Map. Community-Based Management of Acute Malnutrition (CMAM) Forum, 2016

Comparative Studies on Malaria Transmssion Potential at a Lakeshore and a Nearby Inland Site in Western Kenya as it appears in Malaria Atlas Project. Malaria Atlas Project Plasmodium Falciparum Parasite Rate Database. Oxford, United Kingdom: Malaria Atlas Project

Cooney RM, Flanagan KP, Zehyle E. Review of surgical management of cystic hydatid disease in a resource limited setting: Turkana, Kenya. Eur J Gastroenterol Hepatol. 2004; 16(11): 1233-6

Corbett EL, Butterworth AE, Fulford AJ, Ouma JH, Sturrock RF. Nutritional status of children with schistosomiasis mansoni in two different areas of Machakos District, Kenya. Trans R Soc Trop Med Hyg. 1992; 86(3): 266-73

Cystic Echinococcosis Endemicity Estimates identified through systematic review and personal communication, as provided by GBD 2015 expert. [Unpublished]

Daly CC, Maggwa N, Mati JK, Solomon M, Mbugua S, Tukei PM, Hunter DJ. Risk factors for gonorrhoea, syphilis, and trichomonas infections among women attending family planning clinics in Nairobi, Kenya. Genitourin Med. 1994; 70(3): 155-61

Damasceno A, Mayosi BM, Sani M, Ogah OS, Mondo C, Ojji D, Dzudie A, Kouam CK, Suliman A, Schrueder N, Yonga G, Ba SA, Maru F, Alemayehu B, Edwards C, Davison BA, Cotter G, Sliwa K. The causes, treatment, and outcome of acute heart failure in 1006 Africans from 9 countries. Arch Intern Med. 2012; 172(18): 1386-94

Deloron P, Campbell GH, Brandling-Bennett D, Roberts JM, Schwartz IK, Odera JS, Lal AA, Osanga CO, de la Cruz V, McCutchan TM. Antibodies to Plasmodium falciparum ring-infected erythrocyte surface antigen and P. falciparum and P. malariae circumsporozoite proteins: seasonal prevalence in Kenyan villages. Am J Trop Med Hyg. 1989; 41(4): 395-9 as it appears in Malaria Atlas Project. Malaria Atlas Project Plasmodium Falciparum Parasite Rate Database. Oxford, United Kingdom: Malaria Atlas Project

Dent AE, Chelimo K, Sumba PO, Spring MD, Crabb BS, Moormann AM, Tisch DJ, Kazura JW. Temporal stability of naturally acquired immunity to Merozoite Surface Protein-1 in Kenyan Adults. Malar J. 2009; 8(1): 162 as it appears in Malaria Atlas Project. Malaria Atlas Project Plasmodium Falciparum Parasite Rate Database. Oxford, United Kingdom: Malaria Atlas Project

Dent AE, Moormann AM, Yohn CT, Kimmel RJ, Sumba PO, Vulule J, Long CA, Narum DL, Crabb BS, Kazura JW, Tisch DJ. Broadly reactive antibodies specific for Plasmodium falciparum MSP-1(19) are associated with the protection of naturally exposed children against infection. Malar J. 2012; 11(287) as it appears in Malaria Atlas Project. Malaria Atlas Project Plasmodium Falciparum Parasite Rate Database. Oxford, United Kingdom: Malaria Atlas Project

Doenhoff MJ, Butterworth AE, Hayes RJ, Sturrock RF, Ouma JH, Koech D, Prentice M, Bain J. Seroepidemiology and serodiagnosis of schistosomiasis in Kenya using crude and purified egg antigens of Schistosoma mansoni in ELISA. Trans R Soc Trop Med Hyg. 1993; 87(1): 42-8

Drake AL, Kinuthia J, Matemo D, McClelland RS, Unger J, John-Stewart G. P3.079 Prevalence and Cofactors For STIs Among Pregnant Adolescents in Western Kenya. Sex Transm Infect. 2013; 89(Suppl 1): A172-A172

Dynamics of Malaria Transmission and its Epidemiology among Children Population of Kilifi District, Coast Province, Kenya as it appears in Malaria Atlas Project. Malaria Atlas Project Plasmodium Falciparum Parasite Rate Database. Oxford, United Kingdom: Malaria Atlas Project

Edwards T, Scott A, Munyoki G, Odera V, Chengo E, Bauni E, Kwasa T, Sander L, Neville B, Newton C. Active convulsive epilepsy in a rural district of Kenya: a study of prevalence and possible risk factors. Lancet Neurol. 2008; 7(1): 50-6

Environmental Factors Affecting the Development of Plasmodium Falciparum in Anopheles Gambaie Mosquitoes as it appears in Malaria Atlas Project. Malaria Atlas Project Plasmodium Falciparum Parasite Rate Database. Oxford, United Kingdom: Malaria Atlas Project

Esamai F, Ayaya S, Nyandiko W. Prevalence of Asthma, Allergic Rhinitis and Dermatitis in primary school children in Uasin Gishu District, Kenya. East Afr Med J. 2002; 79(10): 514–8

Estambale BB, Simonsen PE, Knight R, Bwayo JJ. Bancroftian filariasis in Kwale District of Kenya. I. Clinical and parasitological survey in an endemic community. Ann Trop Med Parasitol. 1994; 88(2): 145-51 as it appears in London School of Hygiene and Tropical Medicine. Global Atlas of Helminth Infections - Lymphatic Filariasis. London, United Kingdom: London School of Hygiene and Tropical Medicine

Estimation of Attributable Risk of Anemia Due to Schistosomiasis in Western Kenya as it appears in Malaria Atlas Project. Malaria Atlas Project Plasmodium Falciparum Parasite Rate Database. Oxford, United Kingdom: Malaria Atlas Project

Evaluation of Malaria Control in Kisumu Municipality, Kenya: A Case Study as it appears in Malaria Atlas Project. Malaria Atlas Project Plasmodium Falciparum Parasite Rate Database. Oxford, United Kingdom: Malaria Atlas Project

Evidence of Plasmodium Species Interactions in an Endemic Population in Coastal Kenya as it appears in Malaria Atlas Project. Malaria Atlas Project Plasmodium Falciparum Parasite Rate Database. Oxford, United Kingdom: Malaria Atlas Project

Feikin DR, Njenga MK, Bigogo G, Aura B, Aol G, Audi A, Jagero G, Muluare PO, Gikunju S, Nderitu L, Balish A, Winchell J, Schneider E, Erdman D, Oberste MS, Katz MA, Breiman RF. Etiology and Incidence of viral and bacterial acute respiratory illness among older children and adults in rural western Kenya, 2007-2010. PLoS One. 2012; 7(8): e43656

Feikin DR, Njenga MK, Bigogo G, Aura B, Aol G, Audi A, Jagero G, Muluare PO, Gikunju S, Nderitu L, Winchell JM, Schneider E, Erdman DD, Oberste MS, Katz MA, Breiman RF. Viral and bacterial causes of severe acute respiratory illness among children aged less than 5 years in a high malaria prevalence area of western Kenya, 2007-2010. Pediatr Infect Dis J. 2013; 32(1): e14-9

Feikin DR, Olack B, Bigogo GM, Audi A, Cosmas L, Aura B, Burke H, Njenga MK, Williamson J, Breiman RF. The Burden of Common Infectious Disease Syndromes at the Clinic and Household Level from Population-Based Surveillance in Rural and Urban Kenya. PLoS One. 2011; 6(1): e16085

Feksi AT, Kaamugisha J, Gatiti S, Sander JW, Shorvon SD. A comprehensive community epilepsy programme: the Nakuru project. Epilepsy Res. 1991; 8(3): 252-9

Feksi AT, Kaamugisha J, Sander JW, Gatiti S, Shorvon SD. Comprehensive primary health care antiepileptic drug treatment programme in rural and semi-urban Kenya. ICBERG (International Community-based Epilepsy Research Group). Lancet. 1991; 337(8738): 406-9

Fillinger U, Ndenga B, Githeko A, Lindsay SW. Integrated malaria vector control with microbial larvicides and insecticide-treated nets in western Kenya: a controlled trial. Bull World Health Organ. 2009; 87(9): 655-65 as it appears in Malaria Atlas Project. Malaria Atlas Project Plasmodium Falciparum Parasite Rate Database. Oxford, United Kingdom: Malaria Atlas Project

Foote EM, Sullivan KM, Ruth LJ, Oremo J, Sadumah I, Williams TN, Suchdev PS. Determinants of anemia among preschool children in rural, western Kenya. Am J Trop Med Hyg. 2013; 88(4): 757-64

Freeman MC, Clasen T, Brooker SJ, Akoko DO, Rheingans R. The impact of a school-based hygiene, water quality and sanitation intervention on soil-transmitted helminth reinfection: a cluster-randomized trial. Am J Trop Med Hyg. 2013; 89(5): 875-83

Frencken J, Manji F, Mosha H. Dental caries prevalence amongst 12-year-old urban children in East Africa. Community Dent Oral Epidemiol. 1986; 14(2): 94-8

Friis H, Mwaniki D, Omondi B, Muniu E, Magnussen P, Geissler W, Thiong'o F, Michaelsen KF. Serum retinol concentrations and Schistosoma mansoni, intestinal helminths, and malarial parasitemia: a cross-sectional study in Kenyan preschool and primary school children. Am J

Clin Nutr. 1997; 66(3): 665-71 as it appears in Malaria Atlas Project. Malaria Atlas Project Plasmodium Falciparum Parasite Rate Database. Oxford, United Kingdom: Malaria Atlas Project

Friis H, Mwaniki D, Omondi B, Muniu E, Thiong'o F, Ouma J, Magnussen P, Geissler PW, Michaelsen KF. Effects on haemoglobin of multi-micronutrient supplementation and multi-helminth chemotherapy: a randomized, controlled trial in Kenyan school children. Eur J Clin Nutr. 2003; 57(4): 573-9

Fujii Y, Kaneko S, Nzou SM, Mwau M, Njenga SM, Tanigawa C, Kimotho J, Mwangi AW, Kiche I, Matsumoto S, Niki M, Osada-Oka M, Ichinose Y, Inoue M, Itoh M, Tachibana H, Ishii K, Tsuboi T, Yoshida LM, Mondal D, Haque R, Hamano S, Changoma M, Hoshi T, Kamo K-I, Karama M, Miura M, Hirayama K. Serological surveillance development for tropical infectious diseases using simultaneous microsphere-based multiplex assays and finite mixture models.. PLoS Negl Trop Dis. 2014; 8(7): e3040

Fulford AJ, Mbugua GG, Ouma JH, Kariuki HC, Sturrock RF, Butterworth AE. Differences in the rate of hepatosplenomegaly due to Schistosoma mansoni infection between two areas in Machakos District, Kenya. Trans R Soc Trop Med Hyg. 1991; 85(4): 481-8 as it appears in Malaria Atlas Project. Malaria Atlas Project Plasmodium Falciparum Parasite Rate Database. Oxford, United Kingdom: Malaria Atlas Project

Gatei W, Wamae CN, Mbae C, Waruru A, Mulinge E, Waithera T, Gatika SM, Kamwati SK, Revathi G, Hart CA. Cryptosporidiosis: Prevalence, Genotype Analysis, and Symptoms Associated with Infections in Children in Kenya. Am J Trop Med Hyg. 2006; 75(1): 78–82

Gathecha G, Makokha A, Wanzala P, Omolo J, Smith P. Dental caries and oral health practices among 12 year old children in Nairobi West and Mathira West Districts, Kenya. Pan Afr Med J. 2012; 42

Geissler PW, Mwaniki DL, Thiong'o F, Michaelsen KF, Friis H. Geophagy, iron status and anaemia among primary school children in Western Kenya. Trop Med Int Health. 1998; 3(7): 529-34 as it appears in World Health Organization (WHO). WHO Global Database on Anemia, Nutrition Landscape Information System. Geneva, Switzerland: World Health Organization (WHO)

Gichangi P, Renterghem LV, Karanja J, Bwayo J, Kiragu D, Temmerman M. Congenital syphilis in a Nairobi maternity hospital. East Afr Med J. 2004; 81(11): 589-93

Gitonga CW, Karanja PN, Kihara J, Mwanje M, Juma E, Snow RW, Noor AM, Brooker S. Implementing school malaria surveys in Kenya: towards a national surveillance system. Malar J. 2010; 9: 306 as it appears in Malaria Atlas Project. Malaria Atlas Project Plasmodium Falciparum Parasite Rate Database. Oxford, United Kingdom: Malaria Atlas Project

Gouvras AN, Kariuki C, Koukounari A, Norton A, Lange CN, Ireri E, Fenwick A, Mkoji GM, Webster JP. The impact of single versus mixed Schistosoma haematobium and S. mansoni infections on morbidity profiles amongst school-children in Taveta, Kenya. Addict Behav Rep. 2013; 128(2): 309-17

Greene JA, Moormann AM, Vulule J, Bockarie MJ, Zimmerman PA, Kazura JW. Toll-like receptor polymorphisms in malaria-endemic populations. Malar J. 2009; 8: 50 as it appears in Malaria Atlas Project. Malaria Atlas Project Plasmodium Falciparum Parasite Rate Database. Oxford, United Kingdom: Malaria Atlas Project

Greenham R. Anaemia and Schistosoma haematobium infection in the North-Eastern Province of Kenya. Trans R Soc Trop Med Hyg. 1978; 72(1): 72-5

Grimsrud A, Balkan S, Casas EC, Lujan J, Van Cutsem G, Poulet E, Myer L, Pujades-Rodriguez M. Outcomes of antiretroviral therapy over a 10-year period of expansion: a multicohort analysis of African and Asian HIV programs. J Acquir Immune Defic Syndr. 2014; 67(2): e55–66

Guyatt HL, Corlett SK, Robinson TP, Ochola SA, Snow RW. Malaria prevention in highland Kenya: indoor residual house-spraying vs. insecticide-treated bednets. Trop Med Int Health. 2002; 7(4): 298-303 as it appears in Malaria Atlas Project. Malaria Atlas Project Plasmodium Falciparum Parasite Rate Database. Oxford, United Kingdom: Malaria Atlas Project

Hagos B, Khan B, Ofulla AV, Kariuki D, Martin SK. Response of falciparum malaria to chloroquine and three second line antimalarial drugs in a Kenyan coastal school age population. East Afr Med J. 1993; 70(10): 620-3 as it appears in Malaria Atlas Project. Malaria Atlas Project Plasmodium Falciparum Parasite Rate Database. Oxford, United Kingdom: Malaria Atlas Project

Halliday KE, Karanja P, Turner EL, Okello G, Njagi K, Dubeck MM, Allen E, Jukes MCH, Brooker SJ. Plasmodium falciparum, anaemia and cognitive and educational performance among school children in an area of moderate malaria transmission: baseline results of a cluster randomized trial on the coast of Kenya. Trop Med Int Health. 2012; 17(5): 532-49 as it appears in Malaria Atlas Project. Malaria Atlas Project Plasmodium Falciparum Parasite Rate Database. Oxford, United Kingdom: Malaria Atlas Project

Hammitt LL, Kazungu S, Morpeth SC, Gibson DG, Mvera B, Brent AJ, Mwarumba S, Onyango CO, Bett A, Akech DO, Murdoch DR, Nokes DJ, Scott JAG. A preliminary study of pneumonia etiology among hospitalized children in Kenya. Clin Infect Dis. 2012; 54(Supp 2): S190-199

Handzel T, Karanja DM, Addiss DG, Hightower AW, Rosen DH, Colley DG, Andove J, Slutsker L, Secor WE. Geographic distribution of schistosomiasis and soil-transmitted helminths in Western Kenya: implications for anthelminthic mass treatment. Am J Trop Med Hyg. 2003; 69(3): 318-23

Hatcher J, Smith A, Mackenzie I, Thompson S, Bal I, Macharia I, Mugwe P, Okoth-Olende C, Oburra H, Wanjohi Z. A prevalence study of ear problems in school children in Kiambu district, Kenya, May 1992. Int J Pediatr Otorhinolaryngol. 1995; 33(3): 197-205

Hodder SL, Mahmoud AA, Sorenson K, Weinert DM, Stein RL, Ouma JH, Koech D, King CH. Predisposition to urinary tract epithelial metaplasia in Schistosoma haematobium infection. Am J Trop Med Hyg. 2000; 63(3-4): 133-8

Hogrefe W, Su X, Song J, Ashley R, Kong L. Detection of herpes simplex virus type 2-specific immunoglobulin G antibodies in African sera by using recombinant gG2, Western blotting, and gG2 inhibition. J Clin Microbiol. 2002; 40(10): 3635-40

Hopkins DR, Ruiz-Tiben E, Downs P, Withers PC Jr, Maguire JH. Dracunculiasis eradication: the final inch. Am J Trop Med Hyg. 2005; 73(4): 669-675

Hopkins DR, Ruiz-Tiben E, Ruebush TK, Diallo N, Agle A, Withers PC Jr. Dracunculiasis eradication: delayed, not denied.. Am J Trop Med Hyg. 2000; 62(2): 163-8

Human African Trypanosomiasis At-Risk Population Estimates 1980-2015, as provided by the Global Burden of Disease 2010 Nematode expert group. [Unpublished]

Ibinda F, Bauni E, Kariuki SM, Fegan G, Lewa J, Mwikamba M, Boga M, Odhiambo R, Mwagandi K, Seale AC, Berkley JA, Dorfman JR, Newton CR. Incidence and risk factors for neonatal tetanus in admissions to Kilifi County Hospital, Kenya. PLoS One. 2015; 10(4): e0122606

ICF International, Kenya Medical Research Institute (KEMRI), Kenya National Bureau of Statistics, Ministry of Health (Kenya), National AIDS Control Council (Kenya), National Council for Population and Development (Kenya). Kenya Demographic and Health Survey 2014. Fairfax, United States: ICF International

ICF Macro, Kenya Medical Research Institute (KEMRI), Kenya National Bureau of Statistics, Ministry of Public Health and Sanitation (Kenya), National AIDS and STI Control Program (Kenya), National Aids Control Council (NACC), National Coordinating Agency for Population and Development (Kenya). Kenya Demographic and Health Survey 2008-2009. Calverton, United States: ICF Macro

Ilako FM, McLigeyo SO, Riyat MS, Lule GN, Okoth FA, Kaptich D. The prevalence of hepatitis C virus antibodies in renal patients, blood donors and patients with chronic liver disease in Kenya. East Afr Med J. 1995; 72(6): 362-4

Imbahale SS, Fillinger U, Githeko A, Mukabana WR, Takken W. An exploratory survey of malaria prevalence and people's knowledge, attitudes and practices of mosquito larval source management for malaria control in western Kenya. Addict Behav Rep. 2010; 115(3): 248-56 as it appears in Malaria Atlas Project. Malaria Atlas Project Plasmodium Falciparum Parasite Rate Database. Oxford, United Kingdom: Malaria Atlas Project

Insecticide Treated Net Procurement Data, Personal Communication with the World Health Organization 2016 as it appears in Malaria Atlas Project. Malaria Atlas Project Interventions Database

International Centre for Eye Health (ICEH). Kenya - Embu Rapid Assessment of Avoidable Blindness 2007. Grootebroek, Netherlands: RAAB Repository

International Centre for Eye Health (ICEH). Kenya - Kericho Rapid Assessment of Avoidable Blindness 2007. Grootebroek, Netherlands: RAAB Repository

International Centre for Eye Health (ICEH). Kenya - Kwale Rapid Assessment of Avoidable Blindness 2011. Grootebroek, Netherlands: RAAB Repository

International Centre for Eye Health (ICEH). Kenya - Nakuru Rapid Assessment of Avoidable Blindness 2005. Grootebroek, Netherlands: RAAB Repository

International Vaccine Institute. Typhoid Fever Surveillance in Africa Program (TSAP) Typhoid & Salmonella Tabulations

Iodine Nutrition Situation in Kenya and Trends in the Control of Iodine Deficiency as it appears in World Health Organization (WHO). WHO Global Database on Iodine Deficiency. Geneva, Switzerland: World Health Organization (WHO)

Isozumi R, Uemura H, Kimata I, Ichinose Y, Logedi J, Omar AH, Kaneko A. Novel mutations in K13 propeller gene of artemisinin-resistant plasmodium falciparum.(DISPATCHES). Emerg Infect Dis. 2015; 21(3): 490–2 as it appears in Malaria Atlas Project. Malaria Atlas Project Plasmodium Falciparum Parasite Rate Database. Oxford, United Kingdom: Malaria Atlas Project

J. L. Barlow JL, Mung'ala-Odera V, Gona J, Newton CRJC. Brain damage after neonatal tetanus in a rural Kenyan hospital. Trop Med Int Health. 2001; 6(4): 305-308

Jaoko WG, Michael E, Meyrowitsch DW, Estambale BBA, Malecela MN, Simonsen PE. Immunoepidemiology of Wuchereria bancrofti infection: parasite transmission intensity, filaria-specific antibodies, and host immunity in two East African communities. Infect Immun. 2007; 75(12): 5651-62

Jaoko WG, Muchemi G, Oguya FO. Praziquantel side effects during treatment of Schistosoma mansoni infected pupils in Kibwezi, Kenya. East Afr Med J. 1996; 73(8): 499-501

Jenkins R, Othieno C, Ongeri L, Kiima D, Sifuna P, Kingora J, Omollo R, Ogutu B. Alcohol consumption and hazardous drinking in western Kenya--a household survey in a health and demographic surveillance site. BMC Psychiatry. 2015; 15: 230

Jenkins R, Othieno C, Ongeri L, Sifuna P, Ongecha M, Kingora J, Kiima D, Omollo R, Ogutu B. Common mental disorder in Nyanza province, Kenya in 2013 and its associated risk factors –an assessment of change since 2004, using a repeat household survey in a demographic surveillance site. BMC Psychiatry. 2015; 15: 1-12

Jenniskens F, Obwaka E, Kirisuah S, Moses S, Yusufali FM, Achola JO, Fransen L, Laga M, Temmerman M. Syphilis control in pregnancy: decentralization of screening facilities to primary care level, a demonstration project in Nairobi, Kenya. Int J Gynaecol Obstet. 1995; S121–8

Johansen MV, Simonsen PE, Butterworth AE, Ouma JH, Mbugua GG, Sturrock RF, Orinda DA, Christensen NO. A survey of Schistosoma mansoni induced kidney disease in children in an endemic area of Machakos District, Kenya. Addict Behav Rep. 1994; 58(1): 21-8

John CC, McHugh MM, Moormann AM, Sumba PO, Ofulla AV. Low prevalence of Plasmodium falciparum infection among asymptomatic individuals in a highland area of Kenya. Trans R Soc Trop Med Hyg. 2005; 99(10): 780-6 as it appears in Malaria Atlas Project. Malaria Atlas Project Plasmodium Falciparum Parasite Rate Database. Oxford, United Kingdom: Malaria Atlas Project

Joint United Nations Program on HIV/AIDS (UNAIDS), United Nations Children's Fund (UNICEF), World Health Organization (WHO). Kenya Global AIDS Response Progress Reporting (GARPR) System - Antenatal Care Attendees Positive for Syphilis

Joint United Nations Program on HIV/AIDS (UNAIDS). UNAIDS Spectrum - National HIV Estimates 2016. Geneva, Switzerland: Joint United Nations Program on HIV/AIDS (UNAIDS)

Joint WHO/Ministry of Health Report: Malaria Training Programme and Malaria Endemicity in Kenya - Base-line Data as it appears in Malaria Atlas Project. Malaria Atlas Project Plasmodium Falciparum Parasite Rate Database. Oxford, United Kingdom: Malaria Atlas Project

Kaamugisha J, Feksi AT. Determining the prevalence of epilepsy in the semi-urban population of Nakuru, Kenya, comparing two independent methods not apparently used before in epilepsy studies. Neuroepidemiology. 1988; 7(3): 115-21

Kabiru EW, Gachare JW, Mbaabu DA, Ngindu AM, Siongok TK. In-vivo falciparum malaria response to chloroquine in Kisumu-Kenya. East Afr Med J. 1987; 64(9): 606-10 as it appears in Malaria Atlas Project. Malaria Atlas Project Plasmodium Falciparum Parasite Rate Database. Oxford, United Kingdom: Malaria Atlas Project

Kagai JM, Mpoke S, Muli F, Hamburger J, Kenya EU. Molecular technique utilising sputum for detecting Wuchereria bancrofti infections in Malindi, Kenya. East Afr Med J. 2008; 85(3): 118-22 as it appears in London School of Hygiene and Tropical Medicine. Global Atlas of Helminth Infections - Lymphatic Filariasis. London, United Kingdom: London School of Hygiene and Tropical Medicine

Kakai R, Wamola IA, Bwayo JJ, Ndinya-Achola JO. Enteric pathogens in malnourished children with diarrhoea. East Afr Med J. 1995; 72(5): 288-9

Kakai R, Wamola IA, Bwayo JJ. Association of human rotavirus infection and intestinal rotavirus-specific immunoglobulin A in children with diarrhoea. East Afr Med J. 1995; 72(4): 217-9

Karcher H, Omondi A, Odera J, Kunz A, Harms G. Risk factors for treatment denial and loss to follow-up in an antiretroviral treatment cohort in Kenya. Trop Med Int Health. 2007; 12(5): 687-94

Karimurio J, Sheila M, Gichangi M, Adala H, Huguet P. Rapid Assessment of Cataract Surgical Services in Embu District Kenya. East Afr J Ophthalmol. 2007; 13: 19-25

Katamine D, Arap Siongok TK, Kawashima K, Nakajima Y, Nojima H, Imai J. Prevalence of human Schistosomiasis in the Taveta area of Kenya, East Africa. Japan J Trop Med Hyg. 1978; 6(3, 4): 167-80

Kaul R, Kimani J, Nagelkerke NJ, Fonck K, Ngugi EN, Keli F, MacDonald KS, Maclean IW, Bwayo JJ, Temmerman M, Ronald AR, Moses S, Kibera HIV Study Group. Monthly antibiotic chemoprophylaxis and incidence of sexually transmitted infections and HIV-1 infection in Kenyan sex workers: a randomized controlled trial. JAMA. 2004; 291(21): 2555-62

Kenny JV, MacCabe RJ. Sero-epidemiology of hydatid disease in the non-intervention area of north-east Turkana. Ann Trop Med Parasitol. 1993; 87(5): 451-7

Kenya - Chemase Entomology and Parasitology Report July 1981 as it appears in Malaria Atlas Project. Malaria Atlas Project Plasmodium Falciparum Parasite Rate Database. Oxford, United Kingdom: Malaria Atlas Project

Kenya - Chemase Entomology and Parasitology Report June 1981 as it appears in Malaria Atlas Project. Malaria Atlas Project Plasmodium Falciparum Parasite Rate Database. Oxford, United Kingdom: Malaria Atlas Project

Kenya - Division of Vector-Borne Diseases Monthly Report September 1984 as it appears in Malaria Atlas Project. Malaria Atlas Project Plasmodium Falciparum Parasite Rate Database. Oxford, United Kingdom: Malaria Atlas Project

Kenya - Kisii Malariometric Survey, May/June 2007 as it appears in Malaria Atlas Project. Malaria Atlas Project Plasmodium Falciparum Parasite Rate Database. Oxford, United Kingdom: Malaria Atlas Project

Kenya – Mombasa District Division of Vector-Borne Diseases Activities Report 1996 as it appears in Malaria Atlas Project. Malaria Atlas Project Plasmodium Falciparum Parasite Rate Database. Oxford, United Kingdom: Malaria Atlas Project

Kenya - Nyando Integrated Child Health and Education (NICHE) Project Baseline Survey 2007 as it appears in Malaria Atlas Project. Malaria Atlas Project Plasmodium Falciparum Parasite Rate Database. Oxford, United Kingdom: Malaria Atlas Project

Kenya Anthropometric and Micronutrient Nutrition Survey in Kakuma Camp 2001 as it appears in World Health Organization (WHO). WHO Global Database on Child Growth and Malnutrition - Historical. Geneva, Switzerland: World Health Organization (WHO)

Kenya Baseline Survey on Nutrition and Health for the Marsabit Development Program 1994 as it appears in World Health Organization (WHO). WHO Global Database on Child Growth and Malnutrition - Historical. Geneva, Switzerland: World Health Organization (WHO)

Kenya Division of Vector-Borne Diseases - Field Activities Report for the Financial Year 95/96 as it appears in Malaria Atlas Project. Malaria Atlas Project Plasmodium Falciparum Parasite Rate Database. Oxford, United Kingdom: Malaria Atlas Project

Kenya Division of Vector-Borne Diseases Annual and Monthly Reports for 1975-1998 as it appears in Malaria Atlas Project. Malaria Atlas Project Plasmodium Falciparum Parasite Rate Database. Oxford, United Kingdom: Malaria Atlas Project

Kenya Division of Vector-Borne Diseases Annual Report 1979 as it appears in Malaria Atlas Project. Malaria Atlas Project Plasmodium Falciparum Parasite Rate Database. Oxford, United Kingdom: Malaria Atlas Project

Kenya Division of Vector-Borne Diseases Annual Report 1999 as it appears in Malaria Atlas Project. Malaria Atlas Project Plasmodium Falciparum Parasite Rate Database. Oxford, United Kingdom: Malaria Atlas Project

Kenya Division of Vector-Borne Diseases Malariometric Report 1987-1996 as it appears in Malaria Atlas Project. Malaria Atlas Project Plasmodium Falciparum Parasite Rate Database. Oxford, United Kingdom: Malaria Atlas Project

Kenya Division of Vector-Borne Diseases Monthly Report April - June 1996 as it appears in Malaria Atlas Project. Malaria Atlas Project Plasmodium Falciparum Parasite Rate Database. Oxford, United Kingdom: Malaria Atlas Project

Kenya Division of Vector-Borne Diseases Monthly Report April 1976 as it appears in Malaria Atlas Project. Malaria Atlas Project Plasmodium Falciparum Parasite Rate Database. Oxford, United Kingdom: Malaria Atlas Project

Kenya Division of Vector-Borne Diseases Monthly Report April 1978 as it appears in Malaria Atlas Project. Malaria Atlas Project Plasmodium Falciparum Parasite Rate Database. Oxford, United Kingdom: Malaria Atlas Project

Kenya Division of Vector-Borne Diseases Monthly Report April 1979 as it appears in Malaria Atlas Project. Malaria Atlas Project Plasmodium Falciparum Parasite Rate Database. Oxford, United Kingdom: Malaria Atlas Project

Kenya Division of Vector-Borne Diseases Monthly Report April 1981 as it appears in Malaria Atlas Project. Malaria Atlas Project Plasmodium Falciparum Parasite Rate Database. Oxford, United Kingdom: Malaria Atlas Project

Kenya Division of Vector-Borne Diseases Monthly Report April 1984 as it appears in Malaria Atlas Project. Malaria Atlas Project Plasmodium Falciparum Parasite Rate Database. Oxford, United Kingdom: Malaria Atlas Project

Kenya Division of Vector-Borne Diseases Monthly Report April 1985 as it appears in Malaria Atlas Project. Malaria Atlas Project Plasmodium Falciparum Parasite Rate Database. Oxford, United Kingdom: Malaria Atlas Project

Kenya Division of Vector-Borne Diseases Monthly Report April 1991 as it appears in Malaria Atlas Project. Malaria Atlas Project Plasmodium Falciparum Parasite Rate Database. Oxford, United Kingdom: Malaria Atlas Project

Kenya Division of Vector-Borne Diseases Monthly Report April 1993 as it appears in Malaria Atlas Project. Malaria Atlas Project Plasmodium Falciparum Parasite Rate Database. Oxford, United Kingdom: Malaria Atlas Project

Kenya Division of Vector-Borne Diseases Monthly Report August - October 2002 as it appears in Malaria Atlas Project. Malaria Atlas Project Plasmodium Falciparum Parasite Rate Database. Oxford, United Kingdom: Malaria Atlas Project

Kenya Division of Vector-Borne Diseases Monthly Report August 1977 as it appears in Malaria Atlas Project. Malaria Atlas Project Plasmodium Falciparum Parasite Rate Database. Oxford, United Kingdom: Malaria Atlas Project

Kenya Division of Vector-Borne Diseases Monthly Report August 1979 as it appears in Malaria Atlas Project. Malaria Atlas Project Plasmodium Falciparum Parasite Rate Database. Oxford, United Kingdom: Malaria Atlas Project

Kenya Division of Vector-Borne Diseases Monthly Report August 1975 as it appears in Malaria Atlas Project. Malaria Atlas Project Plasmodium Falciparum Parasite Rate Database. Oxford, United Kingdom: Malaria Atlas Project

Kenya Division of Vector-Borne Diseases Monthly Report August 1985 as it appears in Malaria Atlas Project. Malaria Atlas Project Plasmodium Falciparum Parasite Rate Database. Oxford, United Kingdom: Malaria Atlas Project

Kenya Division of Vector-Borne Diseases Monthly Report August 1988 as it appears in Malaria Atlas Project. Malaria Atlas Project Plasmodium Falciparum Parasite Rate Database. Oxford, United Kingdom: Malaria Atlas Project

Kenya Division of Vector-Borne Diseases Monthly Report August 1982 as it appears in Malaria Atlas Project. Malaria Atlas Project Plasmodium Falciparum Parasite Rate Database. Oxford, United Kingdom: Malaria Atlas Project

Kenya Division of Vector-Borne Diseases Monthly Report December 1977 as it appears in Malaria Atlas Project. Malaria Atlas Project Plasmodium Falciparum Parasite Rate Database. Oxford, United Kingdom: Malaria Atlas Project

Kenya Division of Vector-Borne Diseases Monthly Report December 1982 as it appears in Malaria Atlas Project. Malaria Atlas Project Plasmodium Falciparum Parasite Rate Database. Oxford, United Kingdom: Malaria Atlas Project

Kenya Division of Vector-Borne Diseases Monthly Report February 1979 as it appears in Malaria Atlas Project. Malaria Atlas Project Plasmodium Falciparum Parasite Rate Database. Oxford, United Kingdom: Malaria Atlas Project

Kenya Division of Vector-Borne Diseases Monthly Report February 1976 as it appears in Malaria Atlas Project. Malaria Atlas Project Plasmodium Falciparum Parasite Rate Database. Oxford, United Kingdom: Malaria Atlas Project

Kenya Division of Vector-Borne Diseases Monthly Report February 1981 as it appears in Malaria Atlas Project. Malaria Atlas Project Plasmodium Falciparum Parasite Rate Database. Oxford, United Kingdom: Malaria Atlas Project

Kenya Division of Vector-Borne Diseases Monthly Report February 1975 as it appears in Malaria Atlas Project. Malaria Atlas Project Plasmodium Falciparum Parasite Rate Database. Oxford, United Kingdom: Malaria Atlas Project

Kenya Division of Vector-Borne Diseases Monthly Report January - February 1989 as it appears in Malaria Atlas Project. Malaria Atlas Project Plasmodium Falciparum Parasite Rate Database. Oxford, United Kingdom: Malaria Atlas Project

Kenya Division of Vector-Borne Diseases Monthly Report January 1988 as it appears in Malaria Atlas Project. Malaria Atlas Project Plasmodium Falciparum Parasite Rate Database. Oxford, United Kingdom: Malaria Atlas Project

Kenya Division of Vector-Borne Diseases Monthly Report January 1983 as it appears in Malaria Atlas Project. Malaria Atlas Project Plasmodium Falciparum Parasite Rate Database. Oxford, United Kingdom: Malaria Atlas Project

Kenya Division of Vector-Borne Diseases Monthly Report January 1985 as it appears in Malaria Atlas Project. Malaria Atlas Project Plasmodium Falciparum Parasite Rate Database. Oxford, United Kingdom: Malaria Atlas Project

Kenya Division of Vector-Borne Diseases Monthly Report January 1986 as it appears in Malaria Atlas Project. Malaria Atlas Project Plasmodium Falciparum Parasite Rate Database. Oxford, United Kingdom: Malaria Atlas Project

Kenya Division of Vector-Borne Diseases Monthly Report January 1997 as it appears in Malaria Atlas Project. Malaria Atlas Project Plasmodium Falciparum Parasite Rate Database. Oxford, United Kingdom: Malaria Atlas Project

Kenya Division of Vector-Borne Diseases Monthly Report July - August 2001 as it appears in Malaria Atlas Project. Malaria Atlas Project Plasmodium Falciparum Parasite Rate Database. Oxford, United Kingdom: Malaria Atlas Project

Kenya Division of Vector-Borne Diseases Monthly Report July - September 1998 as it appears in Malaria Atlas Project. Malaria Atlas Project Plasmodium Falciparum Parasite Rate Database. Oxford, United Kingdom: Malaria Atlas Project

Kenya Division of Vector-Borne Diseases Monthly Report July 1975 as it appears in Malaria Atlas Project. Malaria Atlas Project Plasmodium Falciparum Parasite Rate Database. Oxford, United Kingdom: Malaria Atlas Project

Kenya Division of Vector-Borne Diseases Monthly Report July 1976 as it appears in Malaria Atlas Project. Malaria Atlas Project Plasmodium Falciparum Parasite Rate Database. Oxford, United Kingdom: Malaria Atlas Project

Kenya Division of Vector-Borne Diseases Monthly Report July 1977 as it appears in Malaria Atlas Project. Malaria Atlas Project Plasmodium Falciparum Parasite Rate Database. Oxford, United Kingdom: Malaria Atlas Project

Kenya Division of Vector-Borne Diseases Monthly Report July 1982 as it appears in Malaria Atlas Project. Malaria Atlas Project Plasmodium Falciparum Parasite Rate Database. Oxford, United Kingdom: Malaria Atlas Project

Kenya Division of Vector-Borne Diseases Monthly Report July 1986 as it appears in Malaria Atlas Project. Malaria Atlas Project Plasmodium Falciparum Parasite Rate Database. Oxford, United Kingdom: Malaria Atlas Project

Kenya Division of Vector-Borne Diseases Monthly Report July 1988 as it appears in Malaria Atlas Project. Malaria Atlas Project Plasmodium Falciparum Parasite Rate Database. Oxford, United Kingdom: Malaria Atlas Project

Kenya Division of Vector-Borne Diseases Monthly Report July 1989 as it appears in Malaria Atlas Project. Malaria Atlas Project Plasmodium Falciparum Parasite Rate Database. Oxford, United Kingdom: Malaria Atlas Project
Kenya Division of Vector-Borne Diseases Monthly Report July 1991 as it appears in Malaria Atlas Project. Malaria Atlas Project Plasmodium Falciparum Parasite Rate Database. Oxford, United Kingdom: Malaria Atlas Project
Kenya Division of Vector-Borne Diseases Monthly Report July 1997 as it appears in Malaria Atlas Project. Malaria Atlas Project Plasmodium Falciparum Parasite Rate Database. Oxford, United Kingdom: Malaria Atlas Project
Kenya Division of Vector-Borne Diseases Monthly Report June - July 2004 as it appears in Malaria Atlas Project. Malaria Atlas Project Plasmodium Falciparum Parasite Rate Database. Oxford, United Kingdom: Malaria Atlas Project
Kenya Division of Vector-Borne Diseases Monthly Report June 1975 as it appears in Malaria Atlas Project. Malaria Atlas Project Plasmodium Falciparum Parasite Rate Database. Oxford, United Kingdom: Malaria Atlas Project
Kenya Division of Vector-Borne Diseases Monthly Report June 1976 as it appears in Malaria Atlas Project. Malaria Atlas Project Plasmodium Falciparum Parasite Rate Database. Oxford, United Kingdom: Malaria Atlas Project
Kenya Division of Vector-Borne Diseases Monthly Report June 1978 as it appears in Malaria Atlas Project. Malaria Atlas Project Plasmodium Falciparum Parasite Rate Database. Oxford, United Kingdom: Malaria Atlas Project
Kenya Division of Vector-Borne Diseases Monthly Report June 1979 as it appears in Malaria Atlas Project. Malaria Atlas Project Plasmodium Falciparum Parasite Rate Database. Oxford, United Kingdom: Malaria Atlas Project
Kenya Division of Vector-Borne Diseases Monthly Report June 1981 as it appears in Malaria Atlas Project. Malaria Atlas Project Plasmodium Falciparum Parasite Rate Database. Oxford, United Kingdom: Malaria Atlas Project
Kenya Division of Vector-Borne Diseases Monthly Report June 1983 as it appears in Malaria Atlas Project. Malaria Atlas Project Plasmodium Falciparum Parasite Rate Database. Oxford, United Kingdom: Malaria Atlas Project
Kenya Division of Vector-Borne Diseases Monthly Report June 1985 as it appears in Malaria Atlas Project. Malaria Atlas Project Plasmodium Falciparum Parasite Rate Database. Oxford, United Kingdom: Malaria Atlas Project
Kenya Division of Vector-Borne Diseases Monthly Report June 1991 as it appears in Malaria Atlas Project. Malaria Atlas Project Plasmodium Falciparum Parasite Rate Database. Oxford, United Kingdom: Malaria Atlas Project
Kenya Division of Vector-Borne Diseases Monthly Report June 2008 as it appears in Malaria Atlas Project. Malaria Atlas Project Plasmodium Falciparum Parasite Rate Database. Oxford, United Kingdom: Malaria Atlas Project
Kenya Division of Vector-Borne Diseases Monthly Report March 1976 as it appears in Malaria Atlas Project. Malaria Atlas Project Plasmodium Falciparum Parasite Rate Database. Oxford, United Kingdom: Malaria Atlas Project
Kenya Division of Vector-Borne Diseases Monthly Report March 1979 as it appears in Malaria Atlas Project. Malaria Atlas Project Plasmodium Falciparum Parasite Rate Database. Oxford, United Kingdom: Malaria Atlas Project
Kenya Division of Vector-Borne Diseases Monthly Report March 1980 as it appears in Malaria Atlas Project. Malaria Atlas Project Plasmodium Falciparum Parasite Rate Database. Oxford, United Kingdom: Malaria Atlas Project

Kenya Division of Vector-Borne Diseases Monthly Report March 1983 as it appears in Malaria Atlas Project. Malaria Atlas Project Plasmodium Falciparum Parasite Rate Database. Oxford, United Kingdom: Malaria Atlas Project
Kenya Division of Vector-Borne Diseases Monthly Report March 1986 as it appears in Malaria Atlas Project. Malaria Atlas Project Plasmodium Falciparum Parasite Rate Database. Oxford, United Kingdom: Malaria Atlas Project
Kenya Division of Vector-Borne Diseases Monthly Report March 1998 as it appears in Malaria Atlas Project. Malaria Atlas Project Plasmodium Falciparum Parasite Rate Database. Oxford, United Kingdom: Malaria Atlas Project
Kenya Division of Vector-Borne Diseases Monthly Report May 1975 as it appears in Malaria Atlas Project. Malaria Atlas Project Plasmodium Falciparum Parasite Rate Database. Oxford, United Kingdom: Malaria Atlas Project
Kenya Division of Vector-Borne Diseases Monthly Report May 1976 as it appears in Malaria Atlas Project. Malaria Atlas Project Plasmodium Falciparum Parasite Rate Database. Oxford, United Kingdom: Malaria Atlas Project
Kenya Division of Vector-Borne Diseases Monthly Report May 1978 as it appears in Malaria Atlas Project. Malaria Atlas Project Plasmodium Falciparum Parasite Rate Database. Oxford, United Kingdom: Malaria Atlas Project
Kenya Division of Vector-Borne Diseases Monthly Report May 1979 as it appears in Malaria Atlas Project. Malaria Atlas Project Plasmodium Falciparum Parasite Rate Database. Oxford, United Kingdom: Malaria Atlas Project
Kenya Division of Vector-Borne Diseases Monthly Report May 1988 as it appears in Malaria Atlas Project. Malaria Atlas Project Plasmodium Falciparum Parasite Rate Database. Oxford, United Kingdom: Malaria Atlas Project
Kenya Division of Vector-Borne Diseases Monthly Report May 1990 as it appears in Malaria Atlas Project. Malaria Atlas Project Plasmodium Falciparum Parasite Rate Database. Oxford, United Kingdom: Malaria Atlas Project
Kenya Division of Vector-Borne Diseases Monthly Report May 1992 as it appears in Malaria Atlas Project. Malaria Atlas Project Plasmodium Falciparum Parasite Rate Database. Oxford, United Kingdom: Malaria Atlas Project
Kenya Division of Vector-Borne Diseases Monthly Report May 1995 as it appears in Malaria Atlas Project. Malaria Atlas Project Plasmodium Falciparum Parasite Rate Database. Oxford, United Kingdom: Malaria Atlas Project
Kenya Division of Vector-Borne Diseases Monthly Report November 1977 as it appears in Malaria Atlas Project. Malaria Atlas Project Plasmodium Falciparum Parasite Rate Database. Oxford, United Kingdom: Malaria Atlas Project
Kenya Division of Vector-Borne Diseases Monthly Report November 1978 as it appears in Malaria Atlas Project. Malaria Atlas Project Plasmodium Falciparum Parasite Rate Database. Oxford, United Kingdom: Malaria Atlas Project
Kenya Division of Vector-Borne Diseases Monthly Report November 1979 as it appears in Malaria Atlas Project. Malaria Atlas Project Plasmodium Falciparum Parasite Rate Database. Oxford, United Kingdom: Malaria Atlas Project
Kenya Division of Vector-Borne Diseases Monthly Report November 1980 as it appears in Malaria Atlas Project. Malaria Atlas Project Plasmodium Falciparum Parasite Rate Database. Oxford, United Kingdom: Malaria Atlas Project
Kenya Division of Vector-Borne Diseases Monthly Report November 1985 as it appears in Malaria Atlas Project. Malaria Atlas Project Plasmodium Falciparum Parasite Rate Database. Oxford, United Kingdom: Malaria Atlas Project

Kenya Division of Vector-Borne Diseases Monthly Report November 1996 as it appears in Malaria Atlas Project. Malaria Atlas Project Plasmodium Falciparum Parasite Rate Database. Oxford, United Kingdom: Malaria Atlas Project
Kenya Division of Vector-Borne Diseases Monthly Report November 1987 as it appears in Malaria Atlas Project. Malaria Atlas Project Plasmodium Falciparum Parasite Rate Database. Oxford, United Kingdom: Malaria Atlas Project
Kenya Division of Vector-Borne Diseases Monthly Report November 1975 as it appears in Malaria Atlas Project. Malaria Atlas Project Plasmodium Falciparum Parasite Rate Database. Oxford, United Kingdom: Malaria Atlas Project
Kenya Division of Vector-Borne Diseases Monthly Report November 1983 as it appears in Malaria Atlas Project. Malaria Atlas Project Plasmodium Falciparum Parasite Rate Database. Oxford, United Kingdom: Malaria Atlas Project
Kenya Division of Vector-Borne Diseases Monthly Report November 1980 and 1982 as it appears in Malaria Atlas Project. Malaria Atlas Project Plasmodium Falciparum Parasite Rate Database. Oxford, United Kingdom: Malaria Atlas Project
Kenya Division of Vector-Borne Diseases Monthly Report October - December 1998 as it appears in Malaria Atlas Project. Malaria Atlas Project Plasmodium Falciparum Parasite Rate Database. Oxford, United Kingdom: Malaria Atlas Project
Kenya Division of Vector-Borne Diseases Monthly Report October 1978 as it appears in Malaria Atlas Project. Malaria Atlas Project Plasmodium Falciparum Parasite Rate Database. Oxford, United Kingdom: Malaria Atlas Project
Kenya Division of Vector-Borne Diseases Monthly Report October 1977 as it appears in Malaria Atlas Project. Malaria Atlas Project Plasmodium Falciparum Parasite Rate Database. Oxford, United Kingdom: Malaria Atlas Project
Kenya Division of Vector-Borne Diseases Monthly Report October 1980 as it appears in Malaria Atlas Project. Malaria Atlas Project Plasmodium Falciparum Parasite Rate Database. Oxford, United Kingdom: Malaria Atlas Project
Kenya Division of Vector-Borne Diseases Monthly Report October 1985 as it appears in Malaria Atlas Project. Malaria Atlas Project Plasmodium Falciparum Parasite Rate Database. Oxford, United Kingdom: Malaria Atlas Project
Kenya Division of Vector-Borne Diseases Monthly Report October 1995 as it appears in Malaria Atlas Project. Malaria Atlas Project Plasmodium Falciparum Parasite Rate Database. Oxford, United Kingdom: Malaria Atlas Project
Kenya Division of Vector-Borne Diseases Monthly Report October 1993 as it appears in Malaria Atlas Project. Malaria Atlas Project Plasmodium Falciparum Parasite Rate Database. Oxford, United Kingdom: Malaria Atlas Project
Kenya Division of Vector-Borne Diseases Monthly Report October 1987 as it appears in Malaria Atlas Project. Malaria Atlas Project Plasmodium Falciparum Parasite Rate Database. Oxford, United Kingdom: Malaria Atlas Project
Kenya Division of Vector-Borne Diseases Monthly Report October 1992 as it appears in Malaria Atlas Project. Malaria Atlas Project Plasmodium Falciparum Parasite Rate Database. Oxford, United Kingdom: Malaria Atlas Project
Kenya Division of Vector-Borne Diseases Monthly Report October 2007 as it appears in Malaria Atlas Project. Malaria Atlas Project Plasmodium Falciparum Parasite Rate Database. Oxford, United Kingdom: Malaria Atlas Project
Kenya Division of Vector-Borne Diseases Monthly Report September 1978 as it appears in Malaria Atlas Project. Malaria Atlas Project Plasmodium Falciparum Parasite Rate Database. Oxford, United Kingdom: Malaria Atlas Project

Kenya Division of Vector-Borne Diseases Monthly Report September 1976 as it appears in Malaria Atlas Project. Malaria Atlas Project Plasmodium Falciparum Parasite Rate Database. Oxford, United Kingdom: Malaria Atlas Project
Kenya Division of Vector-Borne Diseases Monthly Report September 1977 as it appears in Malaria Atlas Project. Malaria Atlas Project Plasmodium Falciparum Parasite Rate Database. Oxford, United Kingdom: Malaria Atlas Project
Kenya Division of Vector-Borne Diseases Monthly Report September 1995 as it appears in Malaria Atlas Project. Malaria Atlas Project Plasmodium Falciparum Parasite Rate Database. Oxford, United Kingdom: Malaria Atlas Project
Kenya Division of Vector-Borne Diseases Monthly Report September 1987 as it appears in Malaria Atlas Project. Malaria Atlas Project Plasmodium Falciparum Parasite Rate Database. Oxford, United Kingdom: Malaria Atlas Project
Kenya Division of Vector-Borne Diseases Monthly Report September 1975 as it appears in Malaria Atlas Project. Malaria Atlas Project Plasmodium Falciparum Parasite Rate Database. Oxford, United Kingdom: Malaria Atlas Project
Kenya Division of Vector-Borne Diseases Monthly Report September 1982 as it appears in Malaria Atlas Project. Malaria Atlas Project Plasmodium Falciparum Parasite Rate Database. Oxford, United Kingdom: Malaria Atlas Project
Kenya Division of Vector-Borne Diseases Monthly Report, Garsen, February 1978 as it appears in Malaria Atlas Project. Malaria Atlas Project Plasmodium Falciparum Parasite Rate Database. Oxford, United Kingdom: Malaria Atlas Project
Kenya Division of Vector-Borne Diseases Monthly Report, Hola District Hospital February 1978 as it appears in Malaria Atlas Project. Malaria Atlas Project Plasmodium Falciparum Parasite Rate Database. Oxford, United Kingdom: Malaria Atlas Project
Kenya Division of Vector-Borne Diseases Return for March 1990 as it appears in Malaria Atlas Project. Malaria Atlas Project Plasmodium Falciparum Parasite Rate Database. Oxford, United Kingdom: Malaria Atlas Project
Kenya Division of Vector-Borne Diseases Return for November 1989 as it appears in Malaria Atlas Project. Malaria Atlas Project Plasmodium Falciparum Parasite Rate Database. Oxford, United Kingdom: Malaria Atlas Project
Kenya Integrated Household Budget Survey 2005-2006 as it appears in World Health Organization (WHO). WHO Global Database on Child Growth and Malnutrition. Geneva, Switzerland: World Health Organization (WHO)
Kenya Lohuk and Kampi Ya Samaki Malariometric Survey 1996 as it appears in Malaria Atlas Project. Malaria Atlas Project Plasmodium Falciparum Parasite Rate Database. Oxford, United Kingdom: Malaria Atlas Project
Kenya Malaria Indicator Survey 2015 as it appears in Malaria Atlas Project. Malaria Atlas Project Plasmodium Falciparum Parasite Rate Database. Oxford, United Kingdom: Malaria Atlas Project
Kenya Malaria Survey 1996 as it appears in Malaria Atlas Project. Malaria Atlas Project Plasmodium Falciparum Parasite Rate Database. Oxford, United Kingdom: Malaria Atlas Project
Kenya Malariometric Survey 1987 as it appears in Malaria Atlas Project. Malaria Atlas Project Plasmodium Falciparum Parasite Rate Database. Oxford, United Kingdom: Malaria Atlas Project
Kenya Malariometric Survey 1999 as it appears in Malaria Atlas Project. Malaria Atlas Project Plasmodium Falciparum Parasite Rate Database. Oxford, United Kingdom: Malaria Atlas Project

Kenya Malariometric Survey 2001 as it appears in Malaria Atlas Project. Malaria Atlas Project Plasmodium Falciparum Parasite Rate Database. Oxford, United Kingdom: Malaria Atlas Project

Kenya Malariometric/Entomological Report 2000 as it appears in Malaria Atlas Project. Malaria Atlas Project Plasmodium Falciparum Parasite Rate Database. Oxford, United Kingdom: Malaria Atlas Project

Kenya Medical Research Institute (KEMRI), Ministry of Health (Kenya), Social Sciences and Medicine Africa Network (SOMA-NET), United Nations Children's Fund (UNICEF), University of Nairobi. Kenya National Micronutrient Survey 1999

Kenya Medical Research Institute (KEMRI). Kenya - Nairobi Cancer Incidence Report 2000-2002. Kenya Medical Research Institute (KEMRI), 2006

Kenya Monthly Reports From Field Stations on Community-based Malaria Prevalence Surveys 1985–1997 as it appears in Malaria Atlas Project. Malaria Atlas Project Plasmodium Falciparum Parasite Rate Database. Oxford, United Kingdom: Malaria Atlas Project

Kenya National Bureau of Statistics, Ministry of Health (Kenya), World Health Organization (WHO). Kenya STEPS Noncommunicable Disease Risk Factors Survey 2015

Kenya National Bureau of Statistics, Population Studies and Research Institute, University of Nairobi (Kenya), United Nations Children's Fund (UNICEF). Kenya - Bungoma County Multiple Indicator Survey 2013-2014. New York, United States: United Nations Children's Fund (UNICEF), 2015

Kenya National Bureau of Statistics, Population Studies and Research Institute, University of Nairobi (Kenya), United Nations Children's Fund (UNICEF). Kenya - Kakamega County Multiple Indicator Survey 2013-2014. New York, United States: United Nations Children's Fund (UNICEF), 2015

Kenya National Bureau of Statistics, Population Studies and Research Institute, University of Nairobi (Kenya), United Nations Children's Fund (UNICEF). Kenya - Turkana County Multiple Indicator Survey 2013-2014. New York, United States: United Nations Children's Fund (UNICEF), 2015

Kenya National Bureau of Statistics, United Nations Children's Fund (UNICEF). Kenya - Eastern Province Multiple Indicator Cluster Survey 2008. Nairobi, Kenya: Kenya National Bureau of Statistics

Kenya National Bureau of Statistics, United Nations Children's Fund (UNICEF). Kenya - Coast Multiple Indicator Cluster Survey 2009. New York, United States: United Nations Children's Fund (UNICEF), 2014

Kenya National Bureau of Statistics, United Nations Children's Fund (UNICEF). Kenya - Nyanza Province Multiple Indicator Cluster Survey 2011. Nairobi, Kenya: Kenya National Bureau of Statistics

Kenya National Bureau of Statistics, United Nations Children's Fund (UNICEF). Kenya - North Eastern Province Multiple Indicator Cluster Survey 2007. Nairobi, Kenya: Kenya National Bureau of Statistics

Kenya National Bureau of Statistics, United Nations Children's Fund (UNICEF). Kenya Multiple Indicator Cluster Survey 1996

Kenya National Micronutrient Survey 2011 as it appears in Petry N, Olofin I, Hurrell RF, Boy E, Wirth JP, Moursi M, Angel MD, Rohner F. The Proportion of Anemia Associated with Iron Deficiency in Low, Medium, and High Human Development Index Countries: A Systematic Analysis of National Surveys. Nutrients. 2016; 8(11)

Kenya Nutrition and Immunization Coverage Survey in Kakuma Camp 1997 as it appears in World Health Organization (WHO). WHO Global Database on Child Growth and Malnutrition - Historical. Geneva, Switzerland: World Health Organization (WHO)

Kenya Plasmodium Falciparum Parasite Rate Data 1979, Personal Communication with Masaba, Ministry of Health 1981 as it appears in Malaria Atlas Project. Malaria Atlas Project Plasmodium Falciparum Parasite Rate Database. Oxford, United Kingdom: Malaria Atlas Project

Kenya Plasmodium Falciparum Parasite Rate Data 1982-1983, Personal Communication with Murigi, Ministry of Health 1985 as it appears in Malaria Atlas Project. Malaria Atlas Project Plasmodium Falciparum Parasite Rate Database. Oxford, United Kingdom: Malaria Atlas Project

Kenya Plasmodium Falciparum Parasite Rate Data 1982-1984, Personal Communication with Murigi, Ministry of Health 1984 as it appears in Malaria Atlas Project. Malaria Atlas Project Plasmodium Falciparum Parasite Rate Database. Oxford, United Kingdom: Malaria Atlas Project

Kenya Plasmodium Falciparum Parasite Rate Data 1983, Personal Communication with Murigi, Ministry of Health 1990 as it appears in Malaria Atlas Project. Malaria Atlas Project Plasmodium Falciparum Parasite Rate Database. Oxford, United Kingdom: Malaria Atlas Project

Kenya Plasmodium Falciparum Parasite Rate Data 1983-1984, Personal Communication with Owino 1984 as it appears in Malaria Atlas Project. Malaria Atlas Project Plasmodium Falciparum Parasite Rate Database. Oxford, United Kingdom: Malaria Atlas Project

Kenya Plasmodium Falciparum Parasite Rate Data 1984, Personal Communication with Roemer 1985 as it appears in Malaria Atlas Project. Malaria Atlas Project Plasmodium Falciparum Parasite Rate Database. Oxford, United Kingdom: Malaria Atlas Project

Kenya Plasmodium Falciparum Parasite Rate Data 2008-2009, Personal Communication with the Division of Vector Borne Diseases 2009 as it appears in Malaria Atlas Project. Malaria Atlas Project Plasmodium Falciparum Parasite Rate Database. Oxford, United Kingdom: Malaria Atlas Project

Kenya Plasmodium Falciparum Parasite Rate Data, A.A. Obala, Paper 46/91, 1991 as it appears in Malaria Atlas Project. Malaria Atlas Project Plasmodium Falciparum Parasite Rate Database. Oxford, United Kingdom: Malaria Atlas Project

Kenya Plasmodium Falciparum Parasite Rate Data, Adungo 1992 as it appears in Malaria Atlas Project. Malaria Atlas Project Plasmodium Falciparum Parasite Rate Database. Oxford, United Kingdom: Malaria Atlas Project

Kenya Plasmodium Falciparum Parasite Rate Data, B.A. Rapuoda 1995 as it appears in Malaria Atlas Project. Malaria Atlas Project Plasmodium Falciparum Parasite Rate Database. Oxford, United Kingdom: Malaria Atlas Project

Kenya Plasmodium Falciparum Parasite Rate Data, C.M. Mbogo 1990 as it appears in Malaria Atlas Project. Malaria Atlas Project Plasmodium Falciparum Parasite Rate Database. Oxford, United Kingdom: Malaria Atlas Project

Kenya Plasmodium Falciparum Parasite Rate Data, D. Ongore 1985 as it appears in Malaria Atlas Project. Malaria Atlas Project Plasmodium Falciparum Parasite Rate Database. Oxford, United Kingdom: Malaria Atlas Project

Kenya Plasmodium Falciparum Parasite Rate Data, E.O. Oongo, Division of Vector-Borne Diseases, Ministry of Health 1996 as it appears in Malaria Atlas Project. Malaria Atlas Project Plasmodium Falciparum Parasite Rate Database. Oxford, United Kingdom: Malaria Atlas Project

Kenya Plasmodium Falciparum Parasite Rate Data, K.M. K'Omollo, Division of Vector-Borne Diseases, Ministry of Health 1994 as it appears in Malaria Atlas Project. Malaria Atlas Project

Plasmodium Falciparum Parasite Rate Database. Oxford, United Kingdom: Malaria Atlas Project

Kenya Plasmodium Falciparum Parasite Rate Data, MSc Thesis submitted by Kaseje DC, Kenyatta University 1989 as it appears in Malaria Atlas Project. Malaria Atlas Project Plasmodium Falciparum Parasite Rate Database. Oxford, United Kingdom: Malaria Atlas Project

Kenya Plasmodium Falciparum Parasite Rate Data, Personal Communication with U. Fillinger 2006 as it appears in Malaria Atlas Project. Malaria Atlas Project Plasmodium Falciparum Parasite Rate Database. Oxford, United Kingdom: Malaria Atlas Project

Kenya Plasmodium Falciparum Parasite Rate Data, Personal Communication with KEMRI-Welcome Trust Research Programme, Kilifi, 2008 as it appears in Malaria Atlas Project. Malaria Atlas Project Plasmodium Falciparum Parasite Rate Database. Oxford, United Kingdom: Malaria Atlas Project

Kenya Plasmodium Falciparum Parasite Rate Data, Personal Communication with KEMRI-Welcome Trust Research Programme, Kilifi, 2009 as it appears in Malaria Atlas Project. Malaria Atlas Project Plasmodium Falciparum Parasite Rate Database. Oxford, United Kingdom: Malaria Atlas Project

Kenya Plasmodium Falciparum Parasite Rate Data, Personal Communication with C.L. King 2009 as it appears in Malaria Atlas Project. Malaria Atlas Project Plasmodium Falciparum Parasite Rate Database. Oxford, United Kingdom: Malaria Atlas Project

Kenya Plasmodium Falciparum Parasite Rate Data, Personal Communication with H. Manda and L.C. Gouagna, International Centre for Insect Physiology and Ecology, 2009 as it appears in Malaria Atlas Project. Malaria Atlas Project Plasmodium Falciparum Parasite Rate Database. Oxford, United Kingdom: Malaria Atlas Project

Kenya Plasmodium Falciparum Parasite Rate Data, Personal Communication with K. Marsh 1995 as it appears in Malaria Atlas Project. Malaria Atlas Project Plasmodium Falciparum Parasite Rate Database. Oxford, United Kingdom: Malaria Atlas Project

Kenya Plasmodium Falciparum Parasite Rate Data, Personal Communication with V.M. Marsh and T. Abuya 2004 as it appears in Malaria Atlas Project. Malaria Atlas Project Plasmodium Falciparum Parasite Rate Database. Oxford, United Kingdom: Malaria Atlas Project

Kenya Plasmodium Falciparum Parasite Rate Data, Personal Communication with C.G. Nevill and R.W. Snow 1993 as it appears in Malaria Atlas Project. Malaria Atlas Project Plasmodium Falciparum Parasite Rate Database. Oxford, United Kingdom: Malaria Atlas Project

Kenya Plasmodium Falciparum Parasite Rate Data, Personal Communication with C.G. Nevill and R.W. Snow 1988 as it appears in Malaria Atlas Project. Malaria Atlas Project Plasmodium Falciparum Parasite Rate Database. Oxford, United Kingdom: Malaria Atlas Project

Kenya Plasmodium Falciparum Parasite Rate Data, Personal Communication with R.W. Snow 1991 as it appears in Malaria Atlas Project. Malaria Atlas Project Plasmodium Falciparum Parasite Rate Database. Oxford, United Kingdom: Malaria Atlas Project

Kenya Plasmodium Falciparum Parasite Rate Data, Personal Communication with D. Zurovac 2000 as it appears in Malaria Atlas Project. Malaria Atlas Project Plasmodium Falciparum Parasite Rate Database. Oxford, United Kingdom: Malaria Atlas Project

Kenya Plasmodium Falciparum Parasite Rate Data, Personal Communication with C. Gitonga and S. Brooker 2009 as it appears in Malaria Atlas Project. Malaria Atlas Project Plasmodium Falciparum Parasite Rate Database. Oxford, United Kingdom: Malaria Atlas Project

Kenya Plasmodium Falciparum Parasite Rate Data, Personal Communication with S.S. Imbahale, A. Githeko, and W. Takken 2009 as it appears in Malaria Atlas Project. Malaria Atlas Project Plasmodium Falciparum Parasite Rate Database. Oxford, United Kingdom: Malaria Atlas Project

Kenya Plasmodium Falciparum Parasite Rate Data, Personal Communication with R.W. Snow, A. Noor, and C. Gitonga, Nairobi KEMRI-Wellcome Trust Research Programme 2009 as it appears in Malaria Atlas Project. Malaria Atlas Project Plasmodium Falciparum Parasite Rate Database. Oxford, United Kingdom: Malaria Atlas Project

Kenya Plasmodium Falciparum Parasite Rate Data, Personal Communication with W. Takken, S. Imbahale, A. Githeko, and A. Busula, Medical and Veterinary Entomology at Wageningen University and Research Centre 2007 as it appears in Malaria Atlas Project. Malaria Atlas Project Plasmodium Falciparum Parasite Rate Database. Oxford, United Kingdom: Malaria Atlas Project

Kenya Plasmodium Falciparum Parasite Rate Data, Personal Communication with the Division of Malaria Control 2007 as it appears in Malaria Atlas Project. Malaria Atlas Project Plasmodium Falciparum Parasite Rate Database. Oxford, United Kingdom: Malaria Atlas Project

Kenya Plasmodium Falciparum Parasite Rate Data, Personal Communication with J. Kihara 2007 as it appears in Malaria Atlas Project. Malaria Atlas Project Plasmodium Falciparum Parasite Rate Database. Oxford, United Kingdom: Malaria Atlas Project

Kenya Plasmodium Falciparum Parasite Rate Data, Personal Communication with J. Kahara 2008 as it appears in Malaria Atlas Project. Malaria Atlas Project Plasmodium Falciparum Parasite Rate Database. Oxford, United Kingdom: Malaria Atlas Project

Kenya Plasmodium Falciparum Parasite Rate Data, Personal Communication with N. Minakawa 2009 as it appears in Malaria Atlas Project. Malaria Atlas Project Plasmodium Falciparum Parasite Rate Database. Oxford, United Kingdom: Malaria Atlas Project

Kenya Plasmodium Falciparum Parasite Rate Data, Personal Communication with the Division of Vector Borne Diseases 2008 as it appears in Malaria Atlas Project. Malaria Atlas Project Plasmodium Falciparum Parasite Rate Database. Oxford, United Kingdom: Malaria Atlas Project

Kenya Plasmodium Falciparum Parasite Rate Data, Personal Communication with Cox 2009 as it appears in Malaria Atlas Project. Malaria Atlas Project Plasmodium Falciparum Parasite Rate Database. Oxford, United Kingdom: Malaria Atlas Project

Kenya Plasmodium Falciparum Parasite Rate Data, Personal Communication with KEMRI/CDC 2009 as it appears in Malaria Atlas Project. Malaria Atlas Project Plasmodium Falciparum Parasite Rate Database. Oxford, United Kingdom: Malaria Atlas Project

Kenya Plasmodium Falciparum Parasite Rate Data, Personal Communication with KEMRI/CDC 2007 as it appears in Malaria Atlas Project. Malaria Atlas Project Plasmodium Falciparum Parasite Rate Database. Oxford, United Kingdom: Malaria Atlas Project

Kenya Plasmodium Falciparum Parasite Rate Data, Personal Communication with Merlin 1999 as it appears in Malaria Atlas Project. Malaria Atlas Project Plasmodium Falciparum Parasite Rate Database. Oxford, United Kingdom: Malaria Atlas Project

Kenya Plasmodium Falciparum Parasite Rate Data, Personal Communication with Muigai 2006 as it appears in Malaria Atlas Project. Malaria Atlas Project Plasmodium Falciparum Parasite Rate Database. Oxford, United Kingdom: Malaria Atlas Project

Kenya Plasmodium Falciparum Parasite Rate Data, Personal Communication with Mui'gau 2004 as it appears in Malaria Atlas Project. Malaria Atlas Project Plasmodium Falciparum Parasite Rate Database. Oxford, United Kingdom: Malaria Atlas Project

Kenya Plasmodium Falciparum Parasite Rate Data, Personal Communication with Mwamguza 2002 as it appears in Malaria Atlas Project. Malaria Atlas Project Plasmodium Falciparum Parasite Rate Database. Oxford, United Kingdom: Malaria Atlas Project

Kenya Plasmodium Falciparum Parasite Rate Data, Personal Communication with Kanyi 2005 as it appears in Malaria Atlas Project. Malaria Atlas Project Plasmodium Falciparum Parasite Rate Database. Oxford, United Kingdom: Malaria Atlas Project

Kenya Plasmodium Falciparum Parasite Rate Data, Personal Communication with Otunge 2004 as it appears in Malaria Atlas Project. Malaria Atlas Project Plasmodium Falciparum Parasite Rate Database. Oxford, United Kingdom: Malaria Atlas Project

Kenya Plasmodium Falciparum Parasite Rate Data, Personal Communication with Namolo 2006 as it appears in Malaria Atlas Project. Malaria Atlas Project Plasmodium Falciparum Parasite Rate Database. Oxford, United Kingdom: Malaria Atlas Project

Kenya Plasmodium Falciparum Parasite Rate Data, Personal Communication with Onyango 2006 as it appears in Malaria Atlas Project. Malaria Atlas Project Plasmodium Falciparum Parasite Rate Database. Oxford, United Kingdom: Malaria Atlas Project

Kenya Plasmodium Falciparum Parasite Rate Data, Personal Communication with Nyago 2006 as it appears in Malaria Atlas Project. Malaria Atlas Project Plasmodium Falciparum Parasite Rate Database. Oxford, United Kingdom: Malaria Atlas Project

Kenya Plasmodium Falciparum Parasite Rate Data, Personal Communication with Nyago 2007 as it appears in Malaria Atlas Project. Malaria Atlas Project Plasmodium Falciparum Parasite Rate Database. Oxford, United Kingdom: Malaria Atlas Project

Kenya Plasmodium Falciparum Parasite Rate Data, Personal Communication with Oloo 2006 as it appears in Malaria Atlas Project. Malaria Atlas Project Plasmodium Falciparum Parasite Rate Database. Oxford, United Kingdom: Malaria Atlas Project

Kenya Plasmodium Falciparum Parasite Rate Data, Personal Communication with Otieno 2005 as it appears in Malaria Atlas Project. Malaria Atlas Project Plasmodium Falciparum Parasite Rate Database. Oxford, United Kingdom: Malaria Atlas Project

Kenya Plasmodium Falciparum Parasite Rate Data, Personal Communication with Otieno 2006 as it appears in Malaria Atlas Project. Malaria Atlas Project Plasmodium Falciparum Parasite Rate Database. Oxford, United Kingdom: Malaria Atlas Project

Kenya Plasmodium Falciparum Parasite Rate Data, Personal Communication with Onjore 2001 as it appears in Malaria Atlas Project. Malaria Atlas Project Plasmodium Falciparum Parasite Rate Database. Oxford, United Kingdom: Malaria Atlas Project

Kenya Plasmodium Falciparum Parasite Rate Data, Personal Communication with Odour 2006 as it appears in Malaria Atlas Project. Malaria Atlas Project Plasmodium Falciparum Parasite Rate Database. Oxford, United Kingdom: Malaria Atlas Project

Kenya Plasmodium Falciparum Parasite Rate Data, Personal Communication with Koech 2002 as it appears in Malaria Atlas Project. Malaria Atlas Project Plasmodium Falciparum Parasite Rate Database. Oxford, United Kingdom: Malaria Atlas Project

Kenya Plasmodium Falciparum Parasite Rate Data, Personal Communication with Rotich 2004 as it appears in Malaria Atlas Project. Malaria Atlas Project Plasmodium Falciparum Parasite Rate Database. Oxford, United Kingdom: Malaria Atlas Project

Kenya Plasmodium Falciparum Parasite Rate Data, Personal Communication with Boiyon 2004 as it appears in Malaria Atlas Project. Malaria Atlas Project Plasmodium Falciparum Parasite Rate Database. Oxford, United Kingdom: Malaria Atlas Project

Kenya Plasmodium Falciparum Parasite Rate Data, Personal Communication with Wangonyi 2002 as it appears in Malaria Atlas Project. Malaria Atlas Project Plasmodium Falciparum Parasite Rate Database. Oxford, United Kingdom: Malaria Atlas Project

Kenya Plasmodium Falciparum Parasite Rate Data, Personal Communication with Kakai 2005 as it appears in Malaria Atlas Project. Malaria Atlas Project Plasmodium Falciparum Parasite Rate Database. Oxford, United Kingdom: Malaria Atlas Project

Kenya Plasmodium Falciparum Parasite Rate Data, Personal Communication with Kakai 2006 as it appears in Malaria Atlas Project. Malaria Atlas Project Plasmodium Falciparum Parasite Rate Database. Oxford, United Kingdom: Malaria Atlas Project

Kenya Plasmodium Falciparum Parasite Rate Data, Personal Communication with Chemuliti 2005 as it appears in Malaria Atlas Project. Malaria Atlas Project Plasmodium Falciparum Parasite Rate Database. Oxford, United Kingdom: Malaria Atlas Project

Kenya Plasmodium Falciparum Parasite Rate Data, Personal Communication with Agak, Ministry of Health 1980 as it appears in Malaria Atlas Project. Malaria Atlas Project Plasmodium Falciparum Parasite Rate Database. Oxford, United Kingdom: Malaria Atlas Project

Kenya Plasmodium Falciparum Parasite Rate Data, Personal Communication with Agak, Ministry of Health 1981 as it appears in Malaria Atlas Project. Malaria Atlas Project Plasmodium Falciparum Parasite Rate Database. Oxford, United Kingdom: Malaria Atlas Project

Kenya Plasmodium Falciparum Parasite Rate Data, Personal Communication with Makazi, Ministry of Health 1976 as it appears in Malaria Atlas Project. Malaria Atlas Project Plasmodium Falciparum Parasite Rate Database. Oxford, United Kingdom: Malaria Atlas Project

Kenya Plasmodium Falciparum Parasite Rate Data, Personal Communication with Makazi, Ministry of Health 1975 as it appears in Malaria Atlas Project. Malaria Atlas Project Plasmodium Falciparum Parasite Rate Database. Oxford, United Kingdom: Malaria Atlas Project

Kenya Plasmodium Falciparum Parasite Rate Data, Personal Communication with Makazi, Ministry of Health 1981 as it appears in Malaria Atlas Project. Malaria Atlas Project Plasmodium Falciparum Parasite Rate Database. Oxford, United Kingdom: Malaria Atlas Project

Kenya Plasmodium Falciparum Parasite Rate Data, Personal Communication with Mugo, Ministry of Health 1981 as it appears in Malaria Atlas Project. Malaria Atlas Project Plasmodium Falciparum Parasite Rate Database. Oxford, United Kingdom: Malaria Atlas Project

Kenya Plasmodium Falciparum Parasite Rate Data, Personal Communication with Mugo, Ministry of Health 1983 as it appears in Malaria Atlas Project. Malaria Atlas Project Plasmodium Falciparum Parasite Rate Database. Oxford, United Kingdom: Malaria Atlas Project

Kenya Plasmodium Falciparum Parasite Rate Data, Personal Communication with Muthinja, Ministry of Health 1984 as it appears in Malaria Atlas Project. Malaria Atlas Project Plasmodium Falciparum Parasite Rate Database. Oxford, United Kingdom: Malaria Atlas Project

Kenya Plasmodium Falciparum Parasite Rate Data, Personal Communication with Ndzovu, Ministry of Health 1981 as it appears in Malaria Atlas Project. Malaria Atlas Project Plasmodium Falciparum Parasite Rate Database. Oxford, United Kingdom: Malaria Atlas Project

Kenya Plasmodium Falciparum Parasite Rate Data, Personal Communication with Omar, Ministry of Health 1982 as it appears in Malaria Atlas Project. Malaria Atlas Project Plasmodium Falciparum Parasite Rate Database. Oxford, United Kingdom: Malaria Atlas Project

Kenya Plasmodium Falciparum Parasite Rate Data, S.B. Tosha, Division of Vector-Borne Diseases, Ministry of Health 1996 as it appears in Malaria Atlas Project. Malaria Atlas Project Plasmodium Falciparum Parasite Rate Database. Oxford, United Kingdom: Malaria Atlas Project

Kenya Plasmodium Falciparum Parasite Rate Data, T. Mugo, Division of Vector-Borne Diseases, Ministry of Health 1994 as it appears in Malaria Atlas Project. Malaria Atlas Project Plasmodium Falciparum Parasite Rate Database. Oxford, United Kingdom: Malaria Atlas Project

Kenya Ramada and Pumwani Primary Schools Malaria and Schistosomiasis Baseline Survey 1993 as it appears in Malaria Atlas Project. Malaria Atlas Project Plasmodium Falciparum Parasite Rate Database. Oxford, United Kingdom: Malaria Atlas Project

Kenya Rural Child Nutrition Survey 1987 as it appears in World Health Organization (WHO). WHO Global Database on Child Growth and Malnutrition. Geneva, Switzerland: World Health Organization (WHO)

Kenya Society for Deaf Children. Kenya Survey on Prevention of Deafness in Children 1996

Kenya Welfare Monitoring Survey II 1994 as it appears in World Health Organization (WHO). WHO Global Database on Child Growth and Malnutrition. Geneva, Switzerland: World Health Organization (WHO)

Khagayi S, Burton DC, Onkoba R, Ochieng B, Ismail A, Mutonga D, Muthoni J, Feikin DR, Breiman RF, Mwenda JM, Odhiambo F, Laserson KF. High burden of rotavirus gastroenteritis in young children in rural western Kenya, 2010-2011. Pediatr Infect Dis J. 2014; S34-40

Kimani D, Mwangi J, Mwangi M, Bunnell R, Kellogg TA, Oluoch T, Gichangi A, Kaiser R, Mugo N, Odongo T, Oduor M, Marum L, KAIS Study Group. Blood donors in Kenya: a comparison of voluntary and family replacement donors based on a population-based survey. Vox Sang. 2011; 100(2): 212–8

Kimani EW, Vulule JM, Kuria IW, Mugisha F. Use of insecticide-treated clothes for personal protection against malaria: a community trial. Malar J. 2006; 5: 63 as it appears in Malaria Atlas Project. Malaria Atlas Project Plasmodium Falciparum Parasite Rate Database. Oxford, United Kingdom: Malaria Atlas Project

King CH, Dickman K, Tisch DJ. Reassessment of the cost of chronic helmintic infection: a meta-analysis of disability-related outcomes in endemic schistosomiasis. Lancet. 2005; 365(9470): 1561–9

King CH, Keating CE, Muruka JF, Ouma JH, Houser H, Siongok TK, Mahmoud AA. Urinary tract morbidity in schistosomiasis haematobia: associations with age and intensity of infection in an endemic area of Coast Province, Kenya. Am J Trop Med Hyg. 1988; 39(4): 361-8 as it appears in Malaria Atlas Project. Malaria Atlas Project Plasmodium Falciparum Parasite Rate Database. Oxford, United Kingdom: Malaria Atlas Project

King CH, Lombardi G, Lombardi C, Greenblatt R, Hodder S, Kinyanjui H, Ouma J, Odiambo O, Bryan PJ, Muruka J. Chemotherapy-based control of schistosomiasis haematobia. I. Metrifonate versus praziquantel in control of intensity and prevalence of infection. Am J Trop Med Hyg. 1988; 39(3): 295-305

King CH, Muchiri EM, Mungai P, Ouma JH, Kadzo H, Magak P, Koech DK. Randomized comparison of low-dose versus standard-dose praziquantel therapy in treatment of urinary tract morbidity due to Schistosoma haema tobium infection. Am J Trop Med Hyg. 2002; 66(6): 725-30

Kiulia NM, Peenze I, Dewar J, Nyachieo A, Galo M, Omolo E, Steele AD, Mwenda JM. Molecular characterisation of the rotavirus strains prevalent in Maua, Meru North, Kenya. East Afr Med J. 2006; 83(7): 360-5

Kloos H, Fulford AJC, Butterworth AE, Sturrock RF, Ouma JH, Kariuki HC, Thiongo FW, Dalton PR, Klumpp RK. Spatial patterns of human water contact and Schistosoma mansoni transmission and infection in four rural areas in Machakos District, Kenya. Soc Sci Med. 1997; 44(7): 949-68

Koenraadt CJ, Paaijmans KP, Schneider P, Githeko AK, Takken W. Low larval vector survival explains unstable malaria in the western Kenya highlands. Trop Med Int Health. 2006; 11(8): 1195-205 as it appears in Malaria Atlas Project. Malaria Atlas Project Plasmodium Falciparum Parasite Rate Database. Oxford, United Kingdom: Malaria Atlas Project

Kohli R, Konya WP, Obura T, Stones W, Revathi G. Prevalence of genital Chlamydia infection in urban women of reproductive age, Nairobi, Kenya. BMC Res Notes. 2013; 44

Kolaczinski JH, Reithinger R, Worku DT, Ocheng A, Kasimiro J, Kabatereine N, Brooker S. Risk factors of visceral leishmaniasis in East Africa: a case-control study in Pokot territory of Kenya and Uganda. Int J Epidemiol. 2008; 37(2): 344-52 as it appears in Malaria Atlas Project. Malaria

Atlas Project Plasmodium Falciparum Parasite Rate Database. Oxford, United Kingdom: Malaria Atlas Project

Kotloff KL, Nataro JP, Blackwelder WC, Nasrin D, Farag TH, Panchalingam S, Wu Y, Sow SO, Sur D, Breiman RF, Faruque AS, Zaidi AK, Saha D, Alonso PL, Tamboura B, Sanogo D, Onwuchekwa U, Manna B, Ramamurthy T, Kanungo S, Ochieng JB, Omore R, Oundo JO, Hossain A, Das SK, Ahmed S, Qureshi S, Quadri F, Adegbola RA, Antonio M, Hossain MJ, Akinsola A, Mandomando I, Nhampossa T, Acácio S, Biswas K, O'Reilly CE, Mintz ED, Berkeley LY, Muhsen K, Sommerfelt H, Robins-Browne RM, Levine MM. Burden and aetiology of diarrhoeal disease in infants and young children in developing countries (the Global Enteric Multicenter Study, GEMS): a prospective, case-control study. Lancet. 2013; 382(9888): 209–22

Koukounari A, Estambale BBA, Njagi JK, Cundill B, Ajanga A, Crudder C, Otido J, Jukes M, Clarke SE, Brooker S. Relationships between anaemia and parasitic infections in Kenyan schoolchildren: a Bayesian hierarchical modelling approach. Int J Parasitol. 2008; 38(14): 1663-71

Kwamanga D, Chakaya J, Sitienei J, Kalisvaart N, L'herminez R, Van der Werf MJ. Tuberculosis transmission in Kenya: results of the third National Tuberculin Survey. Int J Tuberc Lung Dis. 2010; 14(6): 695–700

Lai CK, Beasley R, Crane J, Foliaki S, Shah J, Weiland S. Global variation in the prevalence and severity of asthma symptoms: phase three of the International Study of Asthma and Allergies in Childhood (ISAAC). Thorax. 2009; 64(6): 476-483

Latham MC, Stephenson LS, Hall A, Wolgemuth JC, Elliot TC, Crompton DW. Parasitic infections, anaemia and nutritional status: a study of their interrelationships and the effect of prophylaxis and treatment on workers in Kwale District, Kenya. Trans R Soc Trop Med Hyg. 1983; 77(1): 41-8

Latham MC, Stephenson LS, Hall A, Wolgemuth JC, Elliott TC, Crompton DW. A comparative study of the nutritional status, parasitic infections and health of male roadworkers in four areas of Kenya. Trans R Soc Trop Med Hyg. 1982; 76(6): 734-40

Latham MC, Stephenson LS, Kurz KM, Kinoti SN. Metrifonate or praziquantel treatment improves physical fitness and appetite of Kenyan schoolboys with Schistosoma haematobium and hookworm infections. Am J Trop Med Hyg. 1990; 43(2): 170-9

Lawless JW, Latham MC, Stephenson LS, Kinoti SN, Pertet AM. Iron supplementation improves appetite and growth in anemic Kenyan primary school children. J Nutr. 1994; 124(5): 645-54 as it appears in Malaria Atlas Project. Malaria Atlas Project Plasmodium Falciparum Parasite Rate Database. Oxford, United Kingdom: Malaria Atlas Project

Le Sueur D, Binka F, Lengeler C, De Savigny D, Snow B, Teuscher T, Toure Y. An atlas of malaria in Africa. Afr Health. 1997; 19(2): 23-4 as it appears in Malaria Atlas Project. Malaria Atlas Project Plasmodium Falciparum Parasite Rate Database. Oxford, United Kingdom: Malaria Atlas Project

Leenstra T, Kariuki SK, Kurtis JD, Oloo AJ, Kager PA, ter Kuile FO. Prevalence and severity of anemia and iron deficiency: cross-sectional studies in adolescent schoolgirls in western Kenya. Eur J Clin Nutr. 2004; 58(4): 681-91

Leenstra T, Petersen LT, Kariuki SK, Oloo AJ, Kager PA, ter Kuile FO. Prevalence and severity of malnutrition and age at menarche; cross-sectional studies in adolescent schoolgirls in western Kenya. Eur J Clin Nutr. 2005; 59(1): 41-8 as it appears in Malaria Atlas Project. Malaria Atlas Project Plasmodium Falciparum Parasite Rate Database. Oxford, United Kingdom: Malaria Atlas Project

Lo E, Zhou G, Oo W, Afrane Y, Githeko A, Yan G. Low parasitemia in submicroscopic infections significantly impacts malaria diagnostic sensitivity in the highlands of Western Kenya. PLoS One. 2015; 10(3): e0121763 as it appears in Malaria Atlas Project. Malaria Atlas Project

Plasmodium Falciparum Parasite Rate Database. Oxford, United Kingdom: Malaria Atlas Project
London School of Hygiene and Tropical Medicine. Global Atlas of Helminth Infections - Lymphatic Filariasis. London, United Kingdom: London School of Hygiene and Tropical Medicine
London School of Hygiene and Tropical Medicine. Global Atlas of Helminth Infections - Schistosomiasis. London, United Kingdom: London School of Hygiene and Tropical Medicine
Mabeya H. Characteristics of women with obstetric fistula in the rural hospitals in West Pokot, Kenya. Paper presented at: Postgraduate Training in Research Methodology and Reproductive Health; 2004 Mar; Geneva, Switzerland
Macro Systems, Inc.; Institute for Resource Development, National Council for Population Development (NCPD). Kenya Demographic and Health Survey 1988-1989. Columbia, United States: Macro Systems, Inc
Magak P, King CH, Ireri E, Kadzo H, Ouma JH, Muchiri EM. High prevalence of ectopic kidney in Coast Province, Kenya. Trop Med Int Health. 2004; 9(5): 595-600
Magnussen P, Muchiri E, Mungai P, Ndzovu M, Ouma J, Tosha S. A school-based approach to the control of urinary schistosomiasis and intestinal helminth infections in children in Matuga, Kenya: impact of a two-year chemotherapy programme on prevalence and intensity of infections. Trop Med Int Health. 1997; 2(9): 825-31
Makino Y, Matsumoto I, Chiba Y, Mohammed OA, Ogaja PO, Kibue AM, Muli JM, Nakitare GW. Virological survey of children in Nyeri and Mombasa. Monthly survey of rotavirus in faeces. East Afr Med J. 1983; 60(8): 536-41
Malaria 1982-1997 as it appears in Malaria Atlas Project. Malaria Atlas Project Annual Parasite Incidence Database
Malaria Endemicity in Central Division, Kitui District, Kenya, October, 1983 as it appears in Malaria Atlas Project. Malaria Atlas Project Plasmodium Falciparum Parasite Rate Database. Oxford, United Kingdom: Malaria Atlas Project
Malaria Epidemiology and Drug Resistance in Kisii, Kenya as it appears in Malaria Atlas Project. Malaria Atlas Project Plasmodium Falciparum Parasite Rate Database. Oxford, United Kingdom: Malaria Atlas Project
Malariometric Surveillance Report - Tot Division as it appears in Malaria Atlas Project. Malaria Atlas Project Plasmodium Falciparum Parasite Rate Database. Oxford, United Kingdom: Malaria Atlas Project
Malawi Plasmodium Falciparum Parasite Rate Data, Personal Communication with D.P. Mathanga, S. Brooker, and K.E. Halliday 2013 as it appears in Malaria Atlas Project. Malaria Atlas Project Plasmodium Falciparum Parasite Rate Database. Oxford, United Kingdom: Malaria Atlas Project
Maman D, Pujades-Rodriguez M, Nicholas S, McGuire M, Szumilin E, Ecochard R, Etard J-F. Response to antiretroviral therapy: improved survival associated with CD4 above 500?cells/?l. AIDS. 2012; 26(11): 1393-8
Manji F. Gingivitis, dental fluorosis, and dental caries in primary school children of Nairobi, Kenya. East Afr Med J. 1984; 61(7): 524-32
Marston BJ, Macharia DK, Nga'nga L, Wangai M, Ilako F, Muhenje O, Kjaer M, Isavwa A, Kim A, Chebet K, Decock KM, Weidle PJ. A program to provide antiretroviral therapy to residents of an urban slum in nairobi, kenya. J Int Assoc Physicians AIDS Care (Chic). 2007; 6(2): 106-12
Masaba S. Schistosomiasis in Bunyala and Samia locations of Western Kenya. East Afr Med J. 1978; 55(11): 497-500
Masaba SC, Awiti IE, Muruka JF. Morbidity in urinary schistosomiasis in relation to the intensity of infection in Kisumu, Kenya. J Trop Med Hyg. 1983; 86(2): 65-6

Mathenge W, Kuper H, Limburg H, Polack S, Onyango O, Nyaga G, Foster A. Rapid assessment of avoidable blindness in Nakuru district, Kenya. Ophthalmology. 2007; 114(3): 599-605

Mbogo CM, Mwangangi JM, Nzovu J, Gu W, Yan G, Gunter JT, Swalm C, Keating J, Regens JL, Shililu JI, Githure JI, Beier JC. Spatial and temporal heterogeneity of Anopheles mosquitoes and Plasmodium falciparum transmission along the Kenyan coast. Am J Trop Med Hyg. 2003; 68(6): 734-42 as it appears in Malaria Atlas Project. Malaria Atlas Project Plasmodium Falciparum Parasite Rate Database. Oxford, United Kingdom: Malaria Atlas Project

Mbuba CK, Ngugi AK, Fegan G, Ibinda F, Muchohi SN, Nyundo C, Odhiambo R, Edwards T, Odermatt P, Carter JA, Newton CR. Risk factors associated with the epilepsy treatment gap in Kilifi, Kenya: a cross-sectional study. Lancet Neurol. 2012; 11(8): 688-96

McMorrow ML, Emukule GO, Njuguna HN, Bigogo G, Montgomery JM, Nyawanda B, Audi A, Breiman RF, Katz MA, Cosmas L, Waiboci LW, Duque J, Widdowson MA, Mott JA. The Unrecognized Burden of Influenza in Young Kenyan Children, 2008-2012. PLoS One. 2015; 10(9): e0138272

Mehta SD, Gaydos C, Maclean I, Odoyo-June E, Moses S, Agunda L, Quinn N, Bailey RC. The effect of medical male circumcision on urogenital Mycoplasma genitalium among men in Kisumu, Kenya. Sex Transm Dis. 2012; 39(4): 276–80

Mehta SD, Moses S, Agot K, Agingu W, Parker C, Ndinya-Achola JO, Bailey RC. Herpes simplex virus type 2 infection among young uncircumcised men in Kisumu, Kenya. Sex Transm Infect. 2008; 84(1): 42–8

Mehta SD, Moses S, Parker CB, Agot K, Maclean I, Bailey RC. Circumcision status and incident herpes simplex virus type 2 infection, genital ulcer disease, and HIV infection. AIDS. 2012; 26(9): 1141–9

Menge I, Esamai F, van Reken D, Anabwani G. Paediatric morbidity and mortality at the Eldoret District Hospital, Kenya. East Afr Med J. 1995; 72(3): 165-9

Microscopy Versus Home-based Presumptive Diagnosis of Malaria in a Rural Community in Western Kenya as it appears in Malaria Atlas Project. Malaria Atlas Project Plasmodium Falciparum Parasite Rate Database. Oxford, United Kingdom: Malaria Atlas Project

Ministry of Health (Kenya). Kenya National Inpatient Morbidity and Mortality Statistics 1999

Mirza NM, Caulfield LE, Black RE, Macharia WM. Risk factors for diarrheal duration. Am J Epidemiol. 1997; 146(9): 776-85

Mkoji GM, Muchemi GK, Kipesha FS, Mungai BN, Machai P. Schistosoma mansoni ova in urine of children from an endemic area of Kenya: a short report. East Afr Med J. 1998; 75(9): 558–9

Moisi JC, Saha SK, Falade AG, Njanpop-Lafourcade B-M, Oundo J, Zaidi AKM, Afroj S, Bakare RA, Buss JK, Lasi R, Mueller J, Odekanmi AA, Sangare L, Scott JAG, Knoll MD, Levine OS, Gessner BD. Enhanced diagnosis of pneumococcal meningitis with use of the binax NOW immunochromatographic test of Streptococcus pneumoniae antigen: A multisite study. Clin Infect Dis. 2009; 48(Suppl 2): S49-S56

Moses S, Ngugi EN, Costigan A, Kariuki C, Plummer FA. Declining STDs and HIV prevalences among ANC attenders in Nairobi, Kenya from 1992-2001. The XIV International AIDS Conference; 2002; Barcelona, Spain. (Oral Abstract: The XIV International AIDS Conference: Abstract no. WeOrC1272)

Mugo N, Dadabhai SS, Bunnell R, Williamson J, Bennett E, Baya I, Akinyi N, Mohamed I, Kaiser R. Prevalence of herpes simplex virus type 2 infection, human immunodeficiency virus/herpes simplex virus type 2 coinfection, and associated risk factors in a national, population-based survey in Kenya. Sex Transm Dis. 2011; 38(11): 1059–66

Mukoko DAN, Pedersen EM, Masese NN, Estambale BBA, Ouma JH. Bancroftian filariasis in 12 villages in Kwale district, Coast province, Kenya - variation in clinical and parasitological patterns. Ann Trop Med Parasitol. 2004; 98(8): 801-15

Mung'ala-Odera V, Meehan R, Njuguna P, Mturi N, Alcock KJ, Newton CRJC. Prevalence and risk factors of neurological disability and impairment in children living in rural Kenya. Int J Epidemiol. 2006; 35(3): 683-8

Mung'ala-Odera V, White S, Meehan R, Otieno GO, Njuguna P, Mturi N, Edwards T, Neville BG, Newton CRJC. Prevalence, incidence and risk factors of epilepsy in older children in rural Kenya. Seizure. 2008; 17(5): 396-404

Munyao TM, Othieno-Abinya NA. Cutaneous basal cell carcinoma in Kenya. East Afr Med J. 1999; 76(2): 97-100

Munyekenye OG, Githeko AK, Zhou G, Mushinzimana E, Minakawa N, Yan G. Plasmodium falciparum: Spatial Analysis, Western Kenya Highlands. Emerg Infect Dis. 2005; 11(10): 1571-7 as it appears in Malaria Atlas Project. Malaria Atlas Project Plasmodium Falciparum Parasite Rate Database. Oxford, United Kingdom: Malaria Atlas Project

Mutahi WT, Thiong'o FW. Prevalence and intensity of Schistosomiasis mansoni in irrigation and non-irrigation areas of central Kenya. East Afr Med J. 2005; 82(11): 586-91

Mutanda LN, Kangethe SK, Juma R, Lichenga EO, Gathecha C. Aetiology of diarrhoea in malnourished children at Kenyatta National Hospital. East Afr Med J. 1985; 62(12): 835-41

Mutanda LN, Kinoti SN, Gemert W, Lichenga EO. Age distribution and seasonal pattern of rotavirus infection in children in Kenya. J Diarrhoeal Dis Res. 1984; 2(3): 147-50

Mutanda LN. Epidemiology of acute gastroenteritis in early childhood in Kenya. III. Distribution of the aetiological agents. East Afr Med J. 1980; 57(5): 317-26

Mutanda LN. Epidemiology of acute gastroenteritis in early childhood in Kenya: aetiological agents. Trop Geogr Med. 1980; 32(2): 138-44

Mutero CM, Kabutha C, Kimani V, Kabuage L, Gitau G, Ssennyonga J, Githure J, Muthami L, Kaida A, Musyoka L, Kiarie E, Oganda M. A transdisciplinary perspective on the links between malaria and agroecosystems in Kenya. Addict Behav Rep. 2004; 89(2): 171-86 as it appears in Malaria Atlas Project. Malaria Atlas Project Plasmodium Falciparum Parasite Rate Database. Oxford, United Kingdom: Malaria Atlas Project

Mutero CM, Mutinga MJ, Ngindu AM, Kenya PR, Amimo FA. Visceral leishmaniasis and malaria prevalence in West Pokot District, Kenya. East Afr Med J. 1992; 69(1): 3-8 as it appears in Malaria Atlas Project. Malaria Atlas Project Plasmodium Falciparum Parasite Rate Database. Oxford, United Kingdom: Malaria Atlas Project

Mutero CM, Ouma JH, Agak BK, Wanderi JA, Copeland RS. Malaria prevalence and use of self-protection measures against mosquitoes in Suba District, Kenya. East Afr Med J. 1998; 75(1): 11-5 as it appears in Malaria Atlas Project. Malaria Atlas Project Plasmodium Falciparum Parasite Rate Database. Oxford, United Kingdom: Malaria Atlas Project

Mutinga MJ, Mnzava A, Kimokoti R, Nyamori M, Ngindu AM. Malaria prevalence and morbidity in relation to the use of permethrin-treated wall cloths in Kenya. East Afr Med J. 1993; 70(12): 756-62 as it appears in Malaria Atlas Project. Malaria Atlas Project Plasmodium Falciparum Parasite Rate Database. Oxford, United Kingdom: Malaria Atlas Project

Mutonga D, Langat D, Mwangi D, Tonui J, Njeru M, Abade A, Irura Z, Njeru I, Dahlke M. National surveillance data on the epidemiology of cholera in Kenya, 1997-2010. J Infect Dis. 2013; S55–61

Mutuku FM, King CH, Bustinduy AL, Mungai PL, Muchiri EM, Kitron U. Impact of drought on the spatial pattern of transmission of Schistosoma haematobium in coastal Kenya. Am J Trop Med Hyg. 2011; 85(6): 1065-70

Muturi EJ, Mbogo CM, Mwangangi JM, Ng'ang'a ZW, Kabiru EW, Mwandawiro C, Beier JC. Concomitant infections of Plasmodium falciparum and Wuchereria bancrofti on the Kenyan coast. Filaria J. 2006; 8 as it appears in London School of Hygiene and Tropical Medicine.

Global Atlas of Helminth Infections - Lymphatic Filariasis. London, United Kingdom: London School of Hygiene and Tropical Medicine
Mwangi I, Berkley J, Lowe B, Peshu N, Marsh K, Newton CRJC. Acute bacterial meningitis in children admitted to a rural Kenyan hospital: increasing antibiotic resistance and outcome. Pediatr Infect Dis J. 2002; 21(11): 1042-8
Mwangi J, Gatei DG. Hepatitis B virus, hepatocellular carcinoma and liver cirrhosis in Kenya. East Afr Med J. 1993; 70(4 Suppl): 34-6
Mwangi JW. Viral markers in a blood donor population. East Afr Med J. 1999; 76(1): 35-7
Mwaniki D, Omondi B, Muniu E, Thiong'o F, Ouma J, Magnussen P, Geissler PW, Michaelsen KF, Friis H. Effects on serum retinol of multi-micronutrient supplementation and multi-helminth chemotherapy: a randomised, controlled trial in Kenyan school children. Eur J Clin Nutr. 2002; 56(7): 666-73
Mwaniki MK, Gatakaa HW, Mturi FN, Chesaro CR, Chuma JM, Peshu NM, Mason L, Kager P, Marsh K, English M, Berkley JA, Newton CR. An increase in the burden of neonatal admissions to a rural district hospital in Kenya over 19 years. BMC Public Health. 2010; 10: 591
Mwenda JM, Ntoto KM, Abebe A, Enweronu-Laryea C, Amina I, Mchomvu J, Kisakye A, Mpabalwani EM, Pazvakavambwa I, Armah GE, Seheri LM, Kiulia NM, Page N, Widdowson M-A, Steele AD. Burden and epidemiology of rotavirus diarrhea in selected African countries: preliminary results from the African Rotavirus Surveillance Network. J Infect Dis. 2010; 202(Suppl): S5-11
Mwinzi SMG, Ruberti FR, Stewart JD. Epilepsy in the Kenyan Africa. Med Afr Noire. 1976; 23: 331-334
Nabakwe EC, Lichtenbelt WVM, Ngare DK, Wierik M, Westerterp KR, Owino OC. Vitamin a deficiency and anaemia in young children living in a malaria endemic district of western Kenya. East Afr Med J. 2005; 82(6): 300-6 as it appears in Malaria Atlas Project. Malaria Atlas Project Plasmodium Falciparum Parasite Rate Database. Oxford, United Kingdom: Malaria Atlas Project
Nagi S, Chadeka EA, Sunahara T, Mutungi F, Justin YK, Kaneko S, Ichinose Y, Matsumoto S, Njenga SM, Hashizume M, Shimada M, Hamano S. Risk factors and spatial distribution of Schistosoma mansoni infection among primary school children in Mbita District, Western Kenya. PLoS Negl Trop Dis. 2014; 8(7): e2991 as it appears in Malaria Atlas Project. Malaria Atlas Project Plasmodium Falciparum Parasite Rate Database. Oxford, United Kingdom: Malaria Atlas Project
Naicker S. Burden of end-stage renal disease in sub-Saharan Africa. Clin Nephrol. 2010; 74(Suppl 1): S13-6
Naicker S. End-stage renal disease in sub-Saharan and South Africa. Kidney Int Suppl. 2003; 83: S119-22
Nakata S, Gatheru Z, Ukae S, Adachi N, Kobayashi N, Honma S, Muli J, Ogaja P, Nyangao J, Kiplagat E, Tukei PM, Chiba S. Epidemiological study of the G serotype distribution of group A rotaviruses in Kenya from 1991 to 1994. J Med Virol. 1999; 58(3): 296-303
Nasrin D, Wu Y, Blackwelder WC, Farag TH, Saha D, Sow SO, Alonso PL, Breiman RF, Sur D, Faruque ASG, Zaidi AKM, Biswas K, Van Eijk AM, Walker DG, Levine MM, Kotloff KL. Health care seeking for childhood diarrhea in developing countries: evidence from seven sites in Africa and Asia. Am J Trop Med Hyg. 2013; 89(1 Suppl): 3-12
Ndegwa LK, Karimurio J, Okelo RO, Adala HS. Prevalence of visual impairment and blindness in a Nairobi urban population. East Afr Med J. 2006; 83(4): 69-72
Ndegwa LK, Katz MA, McCormick K, Nganga Z, Mungai A, Emukule G, Kollmann MKHM, Mayieka L, Otieno J, Breiman RF, Mott JA, Ellingson K. Surveillance for respiratory health care-associated infections among inpatients in 3 Kenyan hospitals, 2010-2012. Am J Infect Control. 2014; 42(9): 985–90

Neumann CG, Bwibo NO, Murphy SP, Sigman M, Whaley S, Allen LH, Guthrie D, Weiss RE, Demment MW. Animal source foods improve dietary quality, micronutrient status, growth and cognitive function in Kenyan school children: background, study design and baseline findings. J Nutr. 2003; 133(11 Suppl 2): 3941-3949 as it appears in World Health Organization (WHO). WHO Global Database on Anemia, Nutrition Landscape Information System. Geneva, Switzerland: World Health Organization (WHO)

Nevill CG, Lury JD, Mosobo MK, Watkins HM, Watkins WM. Daily chlorproguanil is an effective alternative to daily proguanil in the prevention of Plasmodium falciparum malaria in Kenya. Trans R Soc Trop Med Hyg. 1994; 88(3): 319-20 as it appears in Malaria Atlas Project. Malaria Atlas Project Plasmodium Falciparum Parasite Rate Database. Oxford, United Kingdom: Malaria Atlas Project

Nevill CG, Watkins WM, Carter JY, Munafu CG. Comparison of mosquito nets, proguanil hydrochloride, and placebo to prevent malaria. Br Med J (Clin Res Ed). 1988; 297(6645): 401-3 as it appears in Malaria Atlas Project. Malaria Atlas Project Plasmodium Falciparum Parasite Rate Database. Oxford, United Kingdom: Malaria Atlas Project

Newton CR, Warn PA, Winstanley PA, Peshu N, Snow RW, Pasvol G, Marsh K. Severe anaemia in children living in a malaria endemic area of Kenya. Trop Med Int Health. 1997; 2(2): 165-78

Ng’etich AI, Rawago FO, Jura WGZO, Mwinzi PN, Won KY, Odiere MR. A cross-sectional study on schistosomiasis and soil-transmitted helminths in Mbita district, western Kenya using different copromicroscopic techniques. Parasit Vectors. 2016; 9: 87

Ng'ang'a PM, Valderhaug J. Dental caries in primary school children in Nairobi, Kenya. Acta Odontol Scand. 1992; 50(5): 269-72

Ngare DK, Muttunga JN, Njonge E. Vitamin A deficiency in pre-school children in Kenya. East Afr Med J. 2000; 77(8): 421-4 as it appears in World Health Organization (WHO). WHO Global Database on Vitamin A Deficiency. Geneva, Switzerland: World Health Organization (WHO)

Ngugi AK, Bottomley C, Kleinschmidt I, Wagner RG, Kakooza-Mwesige A, Ae-Ngibise K, Owusu-Agyei S, Masanja H, Kamuyu G, Odhiambo R, Chengo E, Sander JW, Newton CR, SEEDS group. Prevalence of active convulsive epilepsy in sub-Saharan Africa and associated risk factors: cross-sectional and case-control studies. Lancet Neurol. 2013; 12(3): 253-63

Ngugi AK, Bottomley C, Scott JA, Mung'ala-Odera V, Bauni E, Sander JW, Kleinschmidt I, Newton CR. Incidence of convulsive epilepsy in a rural area in Kenya. Epilepsia. 2013; 54(8): 1352-9

Njenga SM, Muita M, Kirigi G, Mbugua J, Mitsui Y, Fujimaki Y, Aoki Y. Bancroftian filariasis in Kwale district, Kenya. East Afr Med J. 2000; 77(5): 245-9 as it appears in London School of Hygiene and Tropical Medicine. Global Atlas of Helminth Infections - Lymphatic Filariasis. London, United Kingdom: London School of Hygiene and Tropical Medicine

Njenga SM, Mwandawiro CS, Muniu E, Mwanje MT, Haji FM, Bockarie MJ. Adult population as potential reservoir of NTD infections in rural villages of Kwale district, Coastal Kenya: implications for preventive chemotherapy interventions policy. Parasit Vectors. 2011; 4: 175 as it appears in London School of Hygiene and Tropical Medicine. Global Atlas of Helminth Infections - Lymphatic Filariasis. London, United Kingdom: London School of Hygiene and Tropical Medicine

Njenga SM, Mwandawiro CS, Wamae CN, Mukoko DA, Omar AA, Shimada M, Bockarie MJ, Molyneux DH. Sustained reduction in prevalence of lymphatic filariasis infection in spite of missed rounds of mass drug administration in an area under mosquito nets for malaria control. Parasit Vectors. 2011; 90 as it appears in London School of Hygiene and Tropical Medicine. Global Atlas of Helminth Infections - Lymphatic Filariasis. London, United Kingdom: London School of Hygiene and Tropical Medicine

Njenga SM, Wamae CN, Njomo DW, Mwandawiro CS, Molyneux DH. Chronic clinical manifestations related to Wuchereria bancrofti infection in a highly endemic area in Kenya. Trans R Soc Trop Med Hyg. 2007; 101(5): 439-44

Njenga SM, Wamae CN, Njomo DW, Mwandawiro CS, Molyneux DH. Impact of two rounds of mass treatment with diethylcarbamazine plus albendazole on Wuchereria bancrofti infection and the sensitivity of immunochromatographic test in Malindi, Kenya. Trans R Soc Trop Med Hyg. 2008; 102(10): 1017-24

Njenga SM, Wamae CN. Evaluation of ICT filariasis card test using whole capillary blood: comparison with Knott's concentration and counting chamber methods. J Parasitol. 2001; 87(5): 1140-3

Njoroge NW, Kemoli AM, Gatheche LW. Prevalence and pattern of early childhood caries among 3-5 year olds in Kiambaa, Kenya. East Afr Med J. 2010; 87(3): 134-7

Nokes DJ, Ngama M, Bett A, Abwao J, Munywoki P, English M, Scott JAG, Cane PA, Medley GF. Incidence and Severity of Respiratory Syncytial Virus Pneumonia in Rural Kenyan Children Identified through Hospital Surveillance. Clin Infect Dis. 2009; 49(9): 1341–9

Noland GS, Hendel-Paterson B, Min XM, Moormann AM, Vulule JM, Narum DL, Lanar DE, Kazura JW, John CC. Low prevalence of antibodies to preerythrocytic but not blood-stage Plasmodium falciparum antigens in an area of unstable malaria transmission compared to prevalence in an area of stable malaria transmission. Infect Immun. 2008; 76(12): 5721-8 as it appears in Malaria Atlas Project. Malaria Atlas Project Plasmodium Falciparum Parasite Rate Database. Oxford, United Kingdom: Malaria Atlas Project

Nørmark S. Oral health among 15- and 35-44-year-olds in Sierra Leone. Tandlaegebladet. 1991; 95(4): 132-8

Nyamu PN, Otieno CF, Amayo EO, McLigeyo SO. Risk factors and prevalence of diabetic foot ulcers at Kenyatta National Hospital, Nairobi. East Afr Med J. 2003; 80(1): 36-43

Nyiro JU, Sanders EJ, Ngetsa C, Wale S, Awuondo K, Bukusi E, Price MA, Amornkul PN, Nokes DJ. Seroprevalence, predictors and estimated incidence of maternal and neonatal Herpes Simplex Virus type 2 infection in semi-urban women in Kilifi, Kenya. BMC Infect Dis. 2011; 155

Odhiambo JA, Borgdorff MW, Kiambih FM, Kibuga DK, Kwamanga DO, Ng'ang'a L, Agwanda R, Kalisvaart NA, Misljenovic O, Nagelkerke NJ, Bosman M. Tuberculosis and the HIV epidemic: increasing annual risk of tuberculous infection in Kenya, 1986-1996. Am J Public Health. 1999; 89(7): 1078–82

Odhiambo JA, Williams HC, Clayton TO, Robertson CF, Asher MI, ISAAC Phase Three Study Group. Global variations in prevalence of eczema symptoms in children from ISAAC Phase Three. J Allergy Clin Immunol. 2009; 124(6): 1251-1258

Odiere MR, Rawago FO, Ombok M, Secor WE, Karanja DM, Mwinzi PN, Lammie PJ, Won K. High prevalence of schistosomiasis in Mbita and its adjacent islands of Lake Victoria, western Kenya. Parasit Vectors. 2012; 2: 278

Ofulla AV, Moormann AM, Embury PE, Kazura JW, Sumba PO, John CC. Age-related differences in the detection of Plasmodium falciparum infection by PCR and microscopy, in an area of Kenya with holo-endemic malaria. Ann Trop Med Parasitol. 2005; 99(4): 431-5 as it appears in Malaria Atlas Project. Malaria Atlas Project Plasmodium Falciparum Parasite Rate Database. Oxford, United Kingdom: Malaria Atlas Project

Ogutu BR, Apollo OJ, McKinney D, Okoth W, Siangla J, Dubovsky F, Tucker K, Waitumbi JN, Diggs C, Wittes J, Malkin E, Leach A, Soisson LA, Milman JB, Otieno L, Holland CA, Polhemus M, Remich SA, Ockenhouse CF, Cohen J, Ballou WR, Martin SK, Angov E, Stewart VA, Lyon JA, Heppner DG Jr, Withers MR, for the MSP-1 Malaria Vaccine Working Group. Blood Stage Malaria Vaccine Eliciting High Antigen-Specific Antibody Concentrations Confers No Protection to Young Children in Western Kenya. PLoS One. 2009; 4(3): e4708 as it appears in Malaria Atlas Project.

Malaria Atlas Project Plasmodium Falciparum Parasite Rate Database. Oxford, United Kingdom: Malaria Atlas Project
Okech BA, Mwobobia IK, Kamau A, Muiruri S, Mutiso N, Nyambura J, Mwatele C, Amano T, Mwandawiro CS. Use of Integrated Malaria Management Reduces Malaria in Kenya. PLoS One. 2008; 3(12): e4050 as it appears in Malaria Atlas Project. Malaria Atlas Project Plasmodium Falciparum Parasite Rate Database. Oxford, United Kingdom: Malaria Atlas Project
Okoth F, Mbuthia J, Gatheru Z, Murila F, Kanyingi F, Mugo F, Esamai F, Alavi Z, Otieno J, Kiambati H, Wanjuki N. Seroprevalence of hepatitis B markers in pregnant women in Kenya. East Afr Med J. 2006; 83(9): 485-93
Okoth FA, Kobayashi M, Kaptich DC, Kaiguri PM, Tukei PM, Takayanagi T, Yamanaka T. Seroepidemiological study for HBV markers and anti-delta in Kenya. East Afr Med J. 1991; 68(7): 515-25
Olds GR, King C, Hewlett J, Olveda R, Wu G, Ouma J, Peters P, McGarvey S, Odhiambo O, Koech D, Liu CY, Aligui G, Gachihi G, Kombe Y, Parraga I, Ramirez B, Whalen C, Horton RJ, Reeve P. Double-blind placebo-controlled study of concurrent administration of albendazole and praziquantel in schoolchildren with schistosomiasis and geohelminths. J Infect Dis. 1999; 179(4): 996-1003
Oloo A, Githeko A, Adungo N, Karanja D, Vulule J, Kisia-Abok I, Seroney I, Ayisi J, Ondijo S, Koech DK, Abdullah MS. Field trial of permethrin impregnated sisal curtains in malaria control in western Kenya. East Afr Med J. 1996; 73(11): 735-40 as it appears in Malaria Atlas Project. Malaria Atlas Project Plasmodium Falciparum Parasite Rate Database. Oxford, United Kingdom: Malaria Atlas Project
Olsen A, Magnussen P, Ouma JH, Andreassen J, Friis H. The contribution of hookworm and other parasitic infections to haemoglobin and iron status among children and adults in western Kenya. Trans R Soc Trop Med Hyg. 1998; 92(6): 643-9
Olsen A. The proportion of helminth infections in a community in western Kenya which would be treated by mass chemotherapy of schoolchildren. Trans R Soc Trop Med Hyg. 1998; 92(2): 144-8
Omumbo JA. Developing a Risk Map of Malaria Transmission in the East and Horn of Africa [dissertation]. Oxford, United Kingdom: University of Oxford, 2004
Onyango CO, Njeru R, Kazungu S, Achilla R, Bulimo W, Welch SR, Cane PA, Gunson RN, Hammitt LL, Scott JAG, Berkley JA, Nokes DJ. Influenza surveillance among children with pneumonia admitted to a district hospital in coastal Kenya, 2007-2010. J Infect Dis. 2012; 206(Suppl 1): S61-67
O'Reilly CE, Jaron P, Ochieng B, Nyaguara A, Tate JE, Parsons MB, Bopp CA, Williams KA, Vinjé J, Blanton E, Wannemuehler KA, Vulule J, Laserson KF, Breiman RF, Feikin DR, Widdowson M-A, Mintz E. Risk factors for death among children less than 5 years old hospitalized with diarrhea in rural western Kenya, 2005-2007: a cohort study. PLoS Med. 2012; 9(7): e1001256
Otieno FO, Ndivo R, Oswago S, Pals S, Chen R, Thomas T, Kunneke E, Mills LA, McLellan-Lemal E. Correlates of prevalent sexually transmitted infections among participants screened for an HIV incidence cohort study in Kisumu, Kenya. Int J STD AIDS. 2015; 26(4): 225–37
Otieno-Nyunya B, Bennett E, Bunnell R, Dadabhai S, Gichangi A A, Mugo N, Wanyungu J, Baya I, Kaiser R, Kenya AIDS Indicator Survey Study Team. Epidemiology of syphilis in Kenya: results from a nationally representative serological survey. Sex Transm Infect. 2011; 87(6): 521-5
Ouma JH, Waithaka F. Prevalence of Schistosoma mansoni and Schistosoma haematobium in Kitui District, Kenya. East Afr Med J. 1978; 55(2): 54-60

Outbreak of Epidemic Malaria in Uasin Gishu District-1988 as it appears in Malaria Atlas Project. Malaria Atlas Project Plasmodium Falciparum Parasite Rate Database. Oxford, United Kingdom: Malaria Atlas Project

Owino RO, Masiga MA, Ng'ang'a PM, Macigo FG. Dental caries, gingivitis and the treatment needs among 12-year-olds. East Afr Med J. 2010; 87(1): 25-31

Oyoo GO, Ogola EN. Clinical and socio demographic aspects of congestive heart failure patients at Kenyatta National Hospital, Nairobi. East Afr Med J. 1999; 76(1): 23-7

Pigott DM, Bhatt S, Golding N, Duda KA, Battle KE, Brady OJ, Messina JP, Balard Y, Bastien P, Pratlong F, Brownstein JS, Freifeld CC, Mekaru SR, Gething PW, George DB, Myers MF, Reithinger R, Hay SI. Global distribution maps of the leishmaniases. Elife. 2014; 3: nan

Pinoges L, Schramm B, Poulet E, Balkan S, Szumilin E, Ferreyra C, Pujades-Rodríguez M. Risk factors and mortality associated with resistance to first-line antiretroviral therapy: multicentric cross-sectional and longitudinal analyses. J Acquir Immune Defic Syndr. 2015; 68(5): 527–35

Porter K, Zaba B. The empirical evidence for the impact of HIV on adult mortality in the developing world: data from serological studies. AIDS. 2004; 18(Suppl 2): S9-S17

Prevalence of Concomitant Infections of Plasmodium falciparum and Wuchereria bancrofti in Mosquito and Human Populations in Malindi, Kenya as it appears in Malaria Atlas Project. Malaria Atlas Project Plasmodium Falciparum Parasite Rate Database. Oxford, United Kingdom: Malaria Atlas Project

Pullan RL, Smith JL, Jasrasaria R, Brooker SJ. Global numbers of infection and disease burden of soil transmitted helminth infections in 2010 [Unpublished data]. Parasit Vectors. 2014; 7(37). [Unpublished data as provided by the Global Burden of Disease 2010 soil transmitted helminths expert group]

Radin JM, Katz MA, Tempia S, Talla Nzussouo N, Davis R, Duque J, Adedeji A, Adjabeng MJ, Ampofo WK, Ayele W, Bakamutumaho B, Barakat A, Cohen AL, Cohen C, Dalhatu IT, Daouda C, Dueger E, Francisco M, Heraud J-M, Jima D, Kabanda A, Kadjo H, Kandeel A, Bi Shamamba SK, Kasolo F, Kronmann KC, Mazaba Liwewe ML, Lutwama JJ, Matonya M, Mmbaga V, Mott JA, Muhimpundu MA, Muthoka P, Njuguna H, Randrianasolo L, Refaey S, Sanders C, Talaat M, Theo A, Valente F, Venter M, Woodfill C, Bresee J, Moen A, Widdowson M-A. Influenza surveillance in 15 countries in Africa, 2006-2010. J Infect Dis. 2012; S14–21

Reniers G, Slaymaker E, Nakiyingi-Miiro J, Nyamukapa C, Crampin AC, Herbst K, Urassa M, Otieno F, Gregson S, Sewe M, Michael D, Lutalo T, Hosegood V, Kasamba I, Price A, Nabukalu D, Mclean E, Zaba B, ALPHA Network. Mortality trends in the era of antiretroviral therapy: evidence from the Network for Analysing Longitudinal Population based HIV/AIDS data on Africa (ALPHA). AIDS. 2014; S533–42

Report on the Malaria Prevalence Survey in Malindi as it appears in Malaria Atlas Project. Malaria Atlas Project Plasmodium Falciparum Parasite Rate Database. Oxford, United Kingdom: Malaria Atlas Project

Rijpstra AC. Results of duplicated series of stool-examinations for all intestinal parasites by five different methods in school-children in East Africa with remarks on serological aspects of amoebiasis and schistosomiasis. Ann Soc Belg Med Trop. 1975; 55(5): 415-25

Rowland M, Bouma M, Ducornez D, Durrani N, Rozendaal J, Schapira A, Sondorp E. Pyrethroid-impregnated bed nets for personal protection against malaria for Afghan refugees. Trans R Soc Trop Med Hyg. 1996; 90(4): 357-61 as it appears in Malaria Atlas Project. Malaria Atlas Project Plasmodium Falciparum Parasite Rate Database. Oxford, United Kingdom: Malaria Atlas Project

Ruto JJ, Karuga JW. Temporal and spatial epidemiology of sleeping sickness and use of geographical information system (GIS) in Kenya. J Vector Borne Dis. 2009; 46(1): 18-25

Sadarangani M, Makani J, Komba AN, Ajala-Agbo T, Newton CR, Marsh K, Williams TN. An observational study of children with sickle cell disease in Kilifi, Kenya. Br J Haematol. 2009; 146(6): 675-82

Saidi SM, Iijima Y, Sang WK, Mwangudza AK, Oundo JO, Taga K, Aihara M, Nagayama K, Yamamoto H, Waiyaki PG, Honda T. Epidemiological study on infectious diarrheal diseases in children in a coastal rural area of Kenya. Microbiol Immunol. 1997; 41(10): 773-8

Sang WK, Oundo V, Schnabel D. Prevalence and antibiotic resistance of bacterial pathogens isolated from childhood diarrhoea in four provinces of Kenya. J Infect Dev Ctries. 2012; 6(7): 572–8

Satayathum SA, Muchiri EM, Ouma JH, Whalen CC, King CH. Factors affecting infection or reinfection with Schistosoma haematobium in coastal Kenya: survival analysis during a nine-year, school-based treatment program. Am J Trop Med Hyg. 2006; 75(1): 83-92

Sato K, Shimada M, Noda S, Muhoho ND, Katsumata T, Sato A, Aoki Y. Efficacy of metrifonate in a highly endemic area of urinary schistosomiasis in Kenya. Am J Trop Med Hyg. 1988; 38(1): 81-5

Schaefer KU, Khan B, Gachihi GS, Kager PA, Muller AS, Verhave JP, Mcneill KM. Splenomegaly in Baringo District, Kenya, an area endemic for visceral leishmaniasis and malaria. Trop Geogr Med. 1995; 47(3): 111–4 as it appears in Malaria Atlas Project. Malaria Atlas Project Plasmodium Falciparum Parasite Rate Database. Oxford, United Kingdom: Malaria Atlas Project

Schmeller MD W. Community health workers reduce skin diseases in East African children. Int J Dermatol. 1998; 37(5): 370-7

Schmeller W, Baumgartner S, Dzikus A. Dermatophytomycoses in children in rural Kenya: the impact of primary health care. Mycoses. 1997; 40(1-2): 55-63

Schmeller W, Dzikus A. Skin diseases in children in rural Kenya: long-term results of a dermatology project within the primary health care system. Br J Dermatol. 2001; 144(1): 118-24

Schwab L, Steinkuller PG. Visual disability and blindness secondary to refractive errors in Africa. Soc Sci Med. 1983; 17(22): 1751-4

Scott JAG, Berkley JA, Mwangi I, Ochola L, Uyoga S, Macharia A, Ndila C, Lowe BS, Mwarumba S, Bauni E, Marsh K, Williams TN. Relation between falciparum malaria and bacteraemia in Kenyan children: a population-based, case-control study and a longitudinal study. Lancet. 2011; 378(9799): 1316-23

Seal A, Creeke P, Mirghani Z, Abdalla F, McBurney R, Pratt L, Brookes D, Ruth L, Marchand E. Iron and vitamin A deficiency in long-term African refugees. J Nutr. 2005; 135(4): 808-13 as it appears in Malaria Atlas Project. Malaria Atlas Project Plasmodium Falciparum Parasite Rate Database. Oxford, United Kingdom: Malaria Atlas Project

Seedat S, Nyamai C, Njenga F, Vythilingum B, Stein DJ. Trauma exposure and post-traumatic stress symptoms in urban African schools Survey in CapeTown and Nairobi. Br J Psychiatry. 2004; 184(2): 169-75

Sexton JD, Ruebush TK, Brandling-Bennett AD, Breman JG, Roberts JM, Odera JS, Were JB. Permethrin-impregnated curtains and bed-nets prevent malaria in western Kenya. Am J Trop Med Hyg. 1990; 43(1): 11-8 as it appears in Malaria Atlas Project. Malaria Atlas Project Plasmodium Falciparum Parasite Rate Database. Oxford, United Kingdom: Malaria Atlas Project

Shanks GD, Biomndo K, Guyatt HL, Snow RW. Travel as a risk factor for uncomplicated Plasmodium falciparum malaria in the highlands of western Kenya. Trans R Soc Trop Med Hyg. 2005; 99(1): 71-4 as it appears in Malaria Atlas Project. Malaria Atlas Project Plasmodium Falciparum Parasite Rate Database. Oxford, United Kingdom: Malaria Atlas Project

Shapiro RL, Otieno MR, Adcock PM, Phillips-Howard PA, Hawley WA, Kumar L, Waiyaki P, Nahlen BL, Slutsker L. Transmission of epidemic Vibrio cholerae O1 in rural western Kenya associated with drinking water from Lake Victoria: an environmental reservoir for cholera?. Am J Trop Med Hyg. 1999; 60(2): 271–6

Shililu, Maier, Seitz, Orago. Seasonal density, sporozoite rates and entomological inoculation rates of Anopheles gambiae and Anopheles funestus in a high-altitude sugarcane growing zone in western kenya. Trop Med Int Health. 1998; 3(9): 706–10 as it appears in Malaria Atlas Project. Malaria Atlas Project Plasmodium Falciparum Parasite Rate Database. Oxford, United Kingdom: Malaria Atlas Project

Shimada M, Hirata M, Ouma JH, Wambayi E, Thiongo FW, Aoki Y. Epidemiological study of Schistosoma haematobium infection in the coastal area of Kenya. Japan J Trop Med Hyg. 1987; 15(3): 173-84

Shulman CE, Dorman EK, Talisuna AO, Lowe BS, Nevill C, Snow RW, Jilo H, Peshu N, Bulmer JN, Graham S, Marsh K. A community randomized controlled trial of insecticide-treated bednets for the prevention of malaria and anaemia among primigravid women on the Kenyan coast. Trop Med Int Health. 1998; 3(3): 197-204 as it appears in World Health Organization (WHO). WHO Global Database on Anemia, Nutrition Landscape Information System. Geneva, Switzerland: World Health Organization (WHO)

Shulman CE, Graham WJ, Jilo H, Lowe BS, New L, Obiero J, Snow RW, Marsh K. Malaria is an important cause of anaemia in primigravidae: evidence from a district hospital in coastal Kenya. Trans R Soc Trop Med Hyg. 1996; 90(5): 535-9

Shulman CE, Levene M, Morison L, Dorman E, Peshu N, Marsh K. Screening for severe anaemia in pregnancy in Kenya, using pallor examination and self-reported morbidity. Trans R Soc Trop Med Hyg. 2001; 95(3): 250-5 as it appears in World Health Organization (WHO). WHO Global Database on Anemia, Nutrition Landscape Information System. Geneva, Switzerland: World Health Organization (WHO)

Simonsen PE, Bernhard P, Jaoko WG, Meyrowitsch DW, Malecela-Lazaro MN, Magnussen P, Michael E. Filaria dance sign and subclinical hydrocoele in two east African communities with bancroftian filariasis. Trans R Soc Trop Med Hyg. 2002; 96(6): 649-53

Simonsen PE, Meyrowitsch DW, Jaoko WG, Malecela MN, Mukoko D, Pedersen EM, Ouma JH, Rwegoshora RT, Masese N, Magnussen P, Estambale BBA, Michael E. Bancroftian filariasis infection, disease, and specific antibody response patterns in a high and a low endemicity community in East Africa. Am J Trop Med Hyg. 2002; 66(5): 550-9 as it appears in London School of Hygiene and Tropical Medicine. Global Atlas of Helminth Infections - Lymphatic Filariasis. London, United Kingdom: London School of Hygiene and Tropical Medicine

Simonsen PE, Meyrowitsch DW, Mukoko DA, Pedersen EM, Malecela-Lazaro MN, Rwegoshora RT, Ouma JH, Masese N, Jaoko WG, Michael E. The effect of repeated half-yearly diethylcarbamazine mass treatment on Wuchereria bancrofti infection and transmission in two East African communities with different levels of endemicity. Am J Trop Med Hyg. 2004; 70(1): 63-71

Slutsker L, Tipple M, Keane V, McCance C, Campbell CC. Malaria in east African refugees resettling to the United States: development of strategies to reduce the risk of imported malaria. J Infect Dis. 1995; 171(2): 489-93 as it appears in Malaria Atlas Project. Malaria Atlas Project Plasmodium Falciparum Parasite Rate Database. Oxford, United Kingdom: Malaria Atlas Project

Smith DH, Warren KS, Mahmoud AA. Morbidity in schistosomiasis mansoni in relation to intensity of infection: study of a community in Kisumu, Kenya. Am J Trop Med Hyg. 1979; 28(2): 220-9

Smith JL, Brooker S. Impact of hookworm infection and deworming on anaemia in non-pregnant populations: a systematic review. Trop Med Int Health. 2010; 15(7): 776–95

Smith JS, Moses S, Hudgens MG, Parker CB, Agot K, Maclean I, Ndinya-Achola JO, Snijders PJF, Meijer CJLM, Bailey RC. Increased risk of HIV acquisition among Kenyan men with human papillomavirus infection. J Infect Dis. 2010; 201(11): 1677–85

Snow RW, Omumbo JA, Lowe B, Molyneux CS, Obiero JO, Palmer A, Weber MW, Pinder M, Nahlen B, Obonyo C, Newbold C, Gupta S, Marsh K. Relation between severe malaria morbidity in children and level of Plasmodium falciparum transmission in Africa. Lancet. 1997; 349(9066): 1650-4 as it appears in Malaria Atlas Project. Malaria Atlas Project Plasmodium Falciparum Parasite Rate Database. Oxford, United Kingdom: Malaria Atlas Project

Snow RW, Williams RE, Rogers JE, Mung'ala VO, Peshu N. The prevalence of epilepsy among a rural Kenyan population. Its association with premature mortality. Trop Geogr Med. 1994; 46(3): 175-9

Some ES. Misuse of drugs: perceptions of household heads in Kisumu district, Kenya. East Afr Med J. 1994; 71(2): 93-7

Spencer HC, Kaseje DC, Collins WE, Shehata MG, Turner A, Stanfill PS, Huong AY, Roberts JM, Villinski M, Koech DK. Community-based malaria control in Saradidi, Kenya: description of the programme and impact on parasitaemia rates and antimalarial antibodies. Ann Trop Med Parasitol. 1987; 81 Suppl 1: 13–23 as it appears in Malaria Atlas Project. Malaria Atlas Project Plasmodium Falciparum Parasite Rate Database. Oxford, United Kingdom: Malaria Atlas Project

Spencer HC, Kaseje DC, Sempebwa EK, Huong AY, Roberts JM. Malaria chemoprophylaxis to pregnant women provided by community health workers in Saradidi, Kenya. II. Effect on parasitaemia and haemoglobin levels. Ann Trop Med Parasitol. 1987; 83-9 as it appears in World Health Organization (WHO). WHO Global Database on Anemia, Nutrition Landscape Information System. Geneva, Switzerland: World Health Organization (WHO)

Stephenson LS, Kinoti SN, Latham MC, Kurz KM, Kyobe J. Single dose metrifonate or praziquantel treatment in Kenyan children I Effects on Schistosoma haematobium, hookworm, hemoglobin levels, splenomegaly, and hepatomegaly. Am J Trop Med Hyg. 1989; 41(4): 436-44

Stephenson LS, Latham MC, Crompton DWT, Schulpen TWJ, Jansen AA. Nutritional status and stool examinations for intestinal parasites in Kenyan preschool children in Machakos District. East Afr Med J. 1979; 56(1): 1-9

Stephenson LS, Latham MC, Kinoti SN, Oduori ML. Regression of Splenomegaly and Hepatomegaly in Children Treated for Schistosoma haematobium Infection. Am J Trop Med Hyg. 1985; 34(1): 119–23 as it appears in Malaria Atlas Project. Malaria Atlas Project Plasmodium Falciparum Parasite Rate Database. Oxford, United Kingdom: Malaria Atlas Project

Stephenson LS, Latham MC, Kurz KM, Kinoti SN, Oduori ML, Crompton DW. Relationships of Schistosoma hematobium, hookworm and malarial infections and metrifonate treatment to hemoglobin level in Kenyan school children. Am J Trop Med Hyg. 1985; 34(3): 519-28

Stephenson LS, Latham MC, Kurz KM, Miller D, Kinoti SN, Oduori ML. Urinary iron loss and physical fitness of Kenyan children with urinary schistosomiasis. Am J Trop Med Hyg. 1985; 34(2): 322-30

Stevens G. Global Burden of Disease 2010 Expert Group Working Document on Sense and Hearing Disorders

Stevenson JC, Stresman GH, Gitonga CW, Gillig J, Owaga C, Marube E, Odongo W, Okoth A, China P, Oriango R, Brooker SJ, Bousema T, Drakeley C, Cox J. Reliability of School Surveys in Estimating Geographic Variation in Malaria Transmission in the Western Kenyan Highlands. PLoS One. 2013; 8(10): e77641 as it appears in Malaria Atlas Project. Malaria Atlas Project

Plasmodium Falciparum Parasite Rate Database. Oxford, United Kingdom: Malaria Atlas Project
Studies on Malaria and its Vectors in Nairobi: a Review of the Distribution of the Vectors and the Prevalence of the Disease as it appears in Malaria Atlas Project. Malaria Atlas Project Plasmodium Falciparum Parasite Rate Database. Oxford, United Kingdom: Malaria Atlas Project
Sturrock RF, Kariuki HC, Thiongo FW, Gachare JW, Omondi BG, Ouma JH, Mbugua G, Butterworth AE. Schistosomiasis mansoni in Kenya: relationship between infection and anaemia in schoolchildren at the community level. Trans R Soc Trop Med Hyg. 1996; 90(1): 48-54
Suchdev PS, Davis SM, Bartoces M, Ruth LJ, Worrell CM, Kanyi H, Odero K, Wiegand RE, Njenga SM, Montgomery JM, Fox LM. Soil-transmitted helminth infection and nutritional status among urban slum children in Kenya. Am J Trop Med Hyg. 2014; 90(2): 229-305 as it appears in Malaria Atlas Project. Malaria Atlas Project Plasmodium Falciparum Parasite Rate Database. Oxford, United Kingdom: Malaria Atlas Project
Suchdev PS, Ruth LJ, Earley M, Macharia A, Williams TN. The burden and consequences of inherited blood disorders among young children in western Kenya. Matern Child Nutr. 2014; 10(1): 135-44
Swierczewski BE, Odundo EA, Koech MC, Ndonye JN, Kirera RK, Odhiambo CP, Cheruiyot EK, Shaffer DN, Ombogo AN, Oaks EV. Enteric pathogen surveillance in a case-control study of acute diarrhoea in the town of Kisii, Kenya. J Med Microbiol. 2013; 62(Pt 11): 1774-6
Talbert A, Thuo N, Karisa J, Chesaro C, Ohuma E, Ignas J, Berkley JA, Toromo C, Atkinson S, Maitland K. Diarrhoea complicating severe acute malnutrition in Kenyan children: a prospective descriptive study of risk factors and outcome. PLoS One. 2012; 7(6): e38321
Temmerman M, Mohamedali F, Fransen L. Syphilis prevention in pregnancy: an opportunity to improve reproductive and child health in Kenya. Health Policy Plan. 1993; 8(2): 122-7
Temmerman M, Gichangi P, Fonck K, Apers L, Claeys P, Van Renterghem L, Kiragu D, Karanja G, Ndinya-Achola J, Bwayo J. Effect of a syphilis control programme on pregnancy outcome in Nairobi, Kenya. Sex Transm Infect. 2000; 76(2): 117-21
Temmerman M, Plummer FA, Mirza NB, Ndinya-Achola JO, Wamola IA, Nagelkerke N, Brunham RC, Piot P. Infection with HIV as a risk factor for adverse obstetrical outcome. AIDS. 1990; 4(11): 1087-93
Tennant PWG, Pearce MS, Bythell M, Rankin J. 20-year survival of children born with congenital anomalies: a population-based study. Lancet. 2010; 375(9715): 649-56. and Congenital Heart Anomalies Mortality Risk With No Diagnosis or Care Estimates as provided by the Global Burden of Disease 2010 congenital anomaly expert group. [Unpublished]
Ter Kuile FO, Terlouw DJ, Phillips-Howard PA, Hawley WA, Friedman JF, Kolczak MS, Kariuki SK, Shi YP, Kwena AM, Vulule JM, Nahlen BL. Impact of permethrin-treated bed nets on malaria and all-cause morbidity in young children in an area of intense perennial malaria transmission in western Kenya: cross-sectional survey. Am J Trop Med Hyg. 2003; 68(4 Suppl): 100-7
The Malaria Parasite Prevalence Rates in Settled Villages of the Mwea Tebere Irrigation Scheme as it appears in Malaria Atlas Project. Malaria Atlas Project Plasmodium Falciparum Parasite Rate Database. Oxford, United Kingdom: Malaria Atlas Project
Tornheim J, Manya A, Oyando N, Kabaka S, Breiman R, Feikin D. The epidemiology of hospitalized pneumonia in rural Kenya: the potential of surveillance data in setting public health priorities. Int J Infect Dis. 2007; 6(11): 536-43
United Nations Children's Fund (UNICEF), World Health Organization (WHO). WHO and UNICEF Reported Disease Incidence Time Series. Geneva, Switzerland: World Health Organization (WHO)

United Nations Population Division. Trends in International Migrant Stock: Migrants by Destination and Origin - 2013 Revision. New York City, United States: United Nations Population Division
Van't Hoog AH, Laserson KF, Githui WA, Meme HK, Agaya JA, Odeny LO, Muchiri BG, Marston BJ, DeCock KM, Borgdorff MW. High prevalence of pulmonary tuberculosis and inadequate case finding in rural western Kenya. Am J Respir Crit Care Med. 2011; 183(9): 1245-53
Veenemans J, Andang'O PEA, Mbugi EV, Kraaijenhagen RJ, Mwaniki DL, Mockenhaupt FP, Roewer S, Olomi RM, Shao JF, Van Der Meer JWM, Savelkoul HFJ, Verhoef H. Alpha+ -thalassemia protects against anemia associated with asymptomatic malaria: evidence from community-based surveys in Tanzania and Kenya. J Infect Dis. 2008; 198(3): 401–8 as it appears in Malaria Atlas Project. Malaria Atlas Project Plasmodium Falciparum Parasite Rate Database. Oxford, United Kingdom: Malaria Atlas Project
Verani JR, Abudho B, Montgomery SP, Mwinzi PNM, Shane HL, Butler SE, Karanja DMS, Secor WE. Schistosomiasis among young children in Usoma, Kenya. Am J Trop Med Hyg. 2011; 84(5): 787–91
Verhoef H, Hodgins E, Eggelte TA, Carter JY, Lema O, West CE, Kok FJ. Anti-malarial drug use among preschool children in an area of seasonal malaria transmission in Kenya. Am J Trop Med Hyg. 1999; 61(5): 770–5 as it appears in Malaria Atlas Project. Malaria Atlas Project Plasmodium Falciparum Parasite Rate Database. Oxford, United Kingdom: Malaria Atlas Project
Vogel JP, Lee ACC, Souza JP. Maternal morbidity and preterm birth in 22 low- and middle-income countries: a secondary analysis of the WHO Global Survey dataset. BMC Pregnancy Childbirth. 2014; 56
Wafula EM, Onyango FE, Mirza WM, Macharia WM, Wamola I, Ndinya-Achola JO, Agwanda R, Waigwa RN, Musia J. Epidemiology of acute respiratory tract infections among young children in Kenya. Rev Infect Dis. 1990; 12(Suppl 8): S1035-8
Wamae CN, Gatika SM, Roberts JM, Lammie PJ. Wuchereria bancrofti in Kwale District, Coastal Kenya: patterns of focal distribution of infection, clinical manifestations and anti-filarial IgG responsiveness. Parasitology. 1998; 116 (Pt 2): 173-82 as it appears in London School of Hygiene and Tropical Medicine. Global Atlas of Helminth Infections - Lymphatic Filariasis. London, United Kingdom: London School of Hygiene and Tropical Medicine
Wamae CN, Lammie PJ. Haematuria in coastal Kenya is associated with Schistosoma haematobium but not Wuchereria bancrofti infection. Trans R Soc Trop Med Hyg. 1998; 92(1): 63-4
Wamae CN, Njenga SM, Ngugi BM, Mbui J, Njaanake HK. Evaluation of effectiveness of diethylcarbamazine/albendazole combination in reduction of Wuchereria bancrofti infection using multiple infection parameters. Addict Behav Rep. 2011; S33-38 as it appears in London School of Hygiene and Tropical Medicine. Global Atlas of Helminth Infections - Lymphatic Filariasis. London, United Kingdom: London School of Hygiene and Tropical Medicine
Wanjala CL, Waitumbi J, Zhou G, Githeko AK. Identification of malaria transmission and epidemic hotspots in the western Kenya highlands: its application to malaria epidemic prediction. Parasit Vectors. 2011; 4: 81 as it appears in Malaria Atlas Project. Malaria Atlas Project Plasmodium Falciparum Parasite Rate Database. Oxford, United Kingdom: Malaria Atlas Project
Warren KS, Mahmoud AA, Muruka JF, Whittaker LR, Ouma JH, Arap Siongok TK. Schistosomiasis haematobia in coast province Kenya. Am J Trop Med Hyg. 1979; 28(5): 864-70
Watkins WM, Howells RE, Brandling-Bennett AD, Koech DK. In Vitro Susceptibility of Plasmodium falciparum Isolates from Jilore, Kenya, to Antimalarial Drugs. Am J Trop Med Hyg. 1987; 37(3): 445–51 as it appears in Malaria Atlas Project. Malaria Atlas Project Plasmodium Falciparum Parasite Rate Database. Oxford, United Kingdom: Malaria Atlas Project

Watkins WM, Oloo JA, Lury JD, Mosoba M, Kariuki D, Mjomba M, Koech DK, Gilles HM. Efficacy of multiple-dose halofantrine in treatment of chloroquine-resistant falciparum malaria in children in Kenya. Lancet. 1988; 2(8605): 247-50 as it appears in Malaria Atlas Project. Malaria Atlas Project Plasmodium Falciparum Parasite Rate Database. Oxford, United Kingdom: Malaria Atlas Project

Weatherall D. Sickle Cell and Thalassemias Prevalence Data, Personal Correspondence with David Weatherall. [Unpublished]

Weinberger DM, Harboe ZB, Sanders EAM, Ndiritu M, Klugman KP, Rückinger S, Dagan R, Adegbola R, Cutts F, Johnson HL, O'Brien KL, Scott JA, Lipsitch M. Association of Serotype with Risk of Death Due to Pneumococcal Pneumonia: A Meta-Analysis. Clin Infect Dis. 2010; 51(6): 692-9

Weiss HA, Buvé A, Robinson NJ, Van Dyck E, Kahindo M, Anagonou S, Musonda R, Zekeng L, Morison L, Caraël M, Laga M, Hayes RJ, Study Group on Heterogeneity of HIV Epidemics in African Cities. The epidemiology of HSV-2 infection and its association with HIV infection in four urban African populations. AIDS. 2001; S97-108

Were FN, Bwibo NO. Two year neurological outcomes of Very Low Birth Weight infants. East Afr Med J. 2006; 83(5): 243-9

Whitfield R, Schwab L, Ross-Degnan D, Steinkuller P, Swartwood J. Blindness and eye disease in Kenya: ocular status survey results from the Kenya Rural Blindness Prevention Project. Br J Ophthalmol. 1990; 74(6): 333-40

WHO Department of Communicable Disease Surveillance and Response. WHO Report on Global Surveillance of Epidemic-prone Infectious Diseases 2000

Wijers DJ, Kaleli N. Bancroftian filariasis in Kenya. V. Mass treatment given by members of the local community. Ann Trop Med Parasitol. 1984; 78(4): 383-94 as it appears in London School of Hygiene and Tropical Medicine. Global Atlas of Helminth Infections - Lymphatic Filariasis. London, United Kingdom: London School of Hygiene and Tropical Medicine

Willcox MC, Beckman L. Haemoglobin variants, beta-thalassaemia and G-6-PD types in Liberia. Hum Hered. 1981; 31(6): 339-47

Williams H, Stewart A, Von Mutius E, Cookson W, Anderson HR. Is eczema really on the increase worldwide. J Allergy Clin Immunol. 2008; 121(4): 947-954

Williams TN, Mwangi TW, Wambua S, Alexander ND, Kortok M, Snow RW, Marsh K. Sickle cell trait and the risk of Plasmodium falciparum malaria and other childhood diseases. J Infect Dis. 2005; 192(1): 178-86

Wilson S, Booth M, Jones FM, Mwatha JK, Kimani G, Kariuki HC, Vennervald BJ, Ouma JH, Muchiri E, Dunne DW. Age-adjusted Plasmodium falciparum antibody levels in school-aged children are a stable marker of microgeographical variations in exposure to Plasmodium infection. BMC Infect Dis. 2007; 7(1): 67 as it appears in Malaria Atlas Project. Malaria Atlas Project Plasmodium Falciparum Parasite Rate Database. Oxford, United Kingdom: Malaria Atlas Project

Wilson S, Vennervald BJ, Kadzo H, Ireri E, Amaganga C, Booth M, Kariuki HC, Mwatha JK, Kimani G, Ouma JH, Muchiri E, Dunne DW. Hepatosplenomegaly in Kenyan schoolchildren: exacerbation by concurrent chronic exposure to malaria and Schistosoma mansoni infection. Trop Med Int Health. 2007; 12(12): 1442-9 as it appears in Malaria Atlas Project. Malaria Atlas Project Plasmodium Falciparum Parasite Rate Database. Oxford, United Kingdom: Malaria Atlas Project

World Health Organization (WHO). Dracunculiasis Eradication: Global Surveillance Summary, 2002. Wkly Epidemiol Rec. 2003; 78(18): 145-156

World Health Organization (WHO). Dracunculiasis Eradication: Global Surveillance Summary, 2001. Wkly Epidemiol Rec. 2002; 77(18): 141-152

World Health Organization (WHO). Dracunculiasis Eradication: Global Surveillance Summary, 2004. Wkly Epidemiol Rec. 2005; 80(19): 165-175
World Health Organization (WHO). Dracunculiasis Eradication: Global Surveillance Summary, 2006. Wkly Epidemiol Rec. 2007; 82(16): 133-139
World Health Organization (WHO). Dracunculiasis: Global Surveillance Summary, 2000. Wkly Epidemiol Rec. 2001; 76(18): 133-140
World Health Organization (WHO). Dracunculiasis: Global Surveillance Summary, 1996. Wkly Epidemiol Rec. 1997; 19(72): 133-140
World Health Organization (WHO). Global leprosy situation, 2004. Wkly Epidemiol Rec. 2005: 80(13): 118-24
World Health Organization (WHO). Global leprosy situation, 2006. Wkly Epidemiol Rec. 2006: 81(32): 309-16
World Health Organization (WHO). Global leprosy situation, 2010. Wkly Epidemiol Rec. 2010: 85(35): 337-48
World Health Organization (WHO). Global leprosy situation, 2012. Wkly Epidemiol Rec. 2012: 87(34): 317-28
World Health Organization (WHO). Global leprosy situation, September 1999. Wkly Epidemiol Rec. 1999: 74(38): 313-6
World Health Organization (WHO). Kenya WHO Leishmaniasis Country Profile 2014. Geneva, Switzerland: World Health Organization (WHO), 2016
World Health Organization (WHO). Kenya World Health Survey 2004. Geneva, Switzerland: World Health Organization (WHO), 2005
World Health Organization (WHO). Leprosy - Global situation. Wkly Epidemiol Rec. 2000: 75(28): 226-31
World Health Organization (WHO). Leprosy update, 2011. Wkly Epidemiol Rec. 2011: 86(36): 389-99
World Health Organization (WHO). Leprosy. Wkly Epidemiol Rec. 2001: 76(23): 173-9
World Health Organization (WHO). Progress towards leprosy elimination. Wkly Epidemiol Rec. 1997: 72(23): 165-72
World Health Organization (WHO). Progress towards the elimination of leprosy as a public health problem. Wkly Epidemiol Rec. 1996: 71(20): 149-56
World Health Organization (WHO). Progress towards the elimination of leprosy as a public health problem. Part I. Wkly Epidemiol Rec. 1995: 70(25): 177-82
World Health Organization (WHO). WHO Global Health Observatory - Cholera: Number of Reported Cases by Country. Geneva, Switzerland: World Health Organization (WHO)
World Health Organization (WHO). WHO Global Health Observatory - Number of new reported cases (T.b. rhodesiense), Data by country. Geneva, Switzerland: World Health Organization (WHO)
World Health Organization (WHO). WHO Global Health Observatory - Population Living in Trachoma Endemic Areas. Geneva, Switzerland: World Health Organization (WHO)
World Health Organization (WHO). WHO Global Health Observatory Interactive Graph - Number of Cases of Cutaneous Leishmaniasis Reported 2008. Geneva, Switzerland: World Health Organization (WHO)
World Health Organization (WHO). WHO Global Health Observatory Interactive Graph - Number of Cases of Cutaneous Leishmaniasis Reported 2015. Geneva, Switzerland: World Health Organization (WHO)
World Health Organization (WHO). WHO Global Health Observatory Interactive Graph - Number of Cases of Visceral Leishmaniasis Reported 2000. Geneva, Switzerland: World Health Organization (WHO)

World Health Organization (WHO). WHO Global Health Observatory Interactive Graph - Number of Cases of Visceral Leishmaniasis Reported 2001. Geneva, Switzerland: World Health Organization (WHO)
World Health Organization (WHO). WHO Global Health Observatory Interactive Graph - Number of Cases of Visceral Leishmaniasis Reported 2003. Geneva, Switzerland: World Health Organization (WHO)
World Health Organization (WHO). WHO Global Health Observatory Interactive Graph - Number of Cases of Visceral Leishmaniasis Reported 2004. Geneva, Switzerland: World Health Organization (WHO)
World Health Organization (WHO). WHO Global Health Observatory Interactive Graph - Number of Cases of Visceral Leishmaniasis Reported 2005. Geneva, Switzerland: World Health Organization (WHO)
World Health Organization (WHO). WHO Global Health Observatory Interactive Graph - Number of Cases of Visceral Leishmaniasis Reported 2006. Geneva, Switzerland: World Health Organization (WHO)
World Health Organization (WHO). WHO Global Health Observatory Interactive Graph - Number of Cases of Visceral Leishmaniasis Reported 2007. Geneva, Switzerland: World Health Organization (WHO)
World Health Organization (WHO). WHO Global Health Observatory Interactive Graph - Number of Cases of Visceral Leishmaniasis Reported 2008. Geneva, Switzerland: World Health Organization (WHO)
World Health Organization (WHO). WHO Global Health Observatory Interactive Graph - Number of Cases of Visceral Leishmaniasis Reported 2009. Geneva, Switzerland: World Health Organization (WHO)
World Health Organization (WHO). WHO Global Health Observatory Interactive Graph - Number of Cases of Visceral Leishmaniasis Reported 2011. Geneva, Switzerland: World Health Organization (WHO)
World Health Organization (WHO). WHO Global Health Observatory Interactive Graph - Number of Cases of Visceral Leishmaniasis Reported 2012. Geneva, Switzerland: World Health Organization (WHO)
World Health Organization (WHO). WHO Global Health Observatory Interactive Graph - Number of Cases of Visceral Leishmaniasis Reported 2013. Geneva, Switzerland: World Health Organization (WHO)
World Health Organization (WHO). WHO Global Health Observatory Interactive Graph - Number of Cases of Visceral Leishmaniasis Reported 2015. Geneva, Switzerland: World Health Organization (WHO)
World Health Organization (WHO). WHO Global Project on Anti-Tuberculosis Drug Resistance Surveillance Data 1988-2015
World Health Organization (WHO). WHO PCT Databank - Lymphatic Filariasis. Geneva, Switzerland: World Health Organization (WHO)
World Health Organization (WHO). WHO PCT Databank - Soil-transmitted Helminthiases. Geneva, Switzerland: World Health Organization (WHO)
World Health Organization (WHO). WHO Tuberculosis Case Notifications. Geneva, Switzerland: World Health Organization (WHO)
World Health Organization Regional Office for Africa (WHO-AFRO). HIV/AIDS Epidemiological Surveillance Update for the WHO African Region 2002. Brazaville , Congo: World Health Organization Regional Office for Africa (WHO-AFRO), 2003

World Health Organization. Dracunculiasis: Global Surveillance Summary, 1994. Wkly Epidemiol Rec. 1993; 70(18): 125-131

World Malaria Report 2005 as it appears in Malaria Atlas Project. Malaria Atlas Project Interventions Database

World Malaria Report 2008 as it appears in Malaria Atlas Project. Malaria Atlas Project Interventions Database

World Malaria Report 2009 as it appears in Malaria Atlas Project. Malaria Atlas Project Interventions Database

World Malaria Report 2012 as it appears in Malaria Atlas Project. Malaria Atlas Project Interventions Database

World Malaria Report 2013 as it appears in Malaria Atlas Project. Malaria Atlas Project Annual Parasite Incidence Database

World Malaria Report 2015 as it appears in Malaria Atlas Project. Malaria Atlas Project Annual Parasite Incidence Database

World Malaria Report 2016 as it appears in Malaria Atlas Project. Malaria Atlas Project Annual Parasite Incidence Database

Worldwide variations in the prevalence of asthma symptoms: the International Study of Asthma and Allergies in Childhood (ISAAC). Eur Respir J. 1998; 12(2): 315-35

Wu VK, Poenaru D, Poley MJ. Burden of surgical congenital anomalies in Kenya: a population-based study. J Trop Pediatr. 2013; 59(3): 195-202

Ye Y, Madise N, Ndugwa R, Ochola S, Snow RW. Fever treatment in the absence of malaria transmission in an urban informal settlement in Nairobi, Kenya. Malar J. 2009; 8(1): 160 as it appears in Malaria Atlas Project. Malaria Atlas Project Plasmodium Falciparum Parasite Rate Database. Oxford, United Kingdom: Malaria Atlas Project

Zucker JR, Lackritz EM, Ruebush TK, Hightower AW, Adungosi JE, Were JB, Campbell CC. Anaemia, blood transfusion practices, HIV and mortality among women of reproductive age in western Kenya. Trans R Soc Trop Med Hyg. 1994; 88(2): 173-6

Appendix Table 4. Data used in Kenya risk factor estimation, GBD 2016

Abt Associates Inc., Kenya National Bureau of Statistics, Ministry of Health (Kenya). Kenya Household Health Expenditure and Utilization Survey 2007. Nairobi, Kenya: Kenya National Bureau of Statistics

Akhwale WS, Lum JK, Kaneko A, Eto H, Obonyo C, Björkman A, Kobayakawa T. Anemia and malaria at different altitudes in the western highlands of Kenya. Addict Behav Rep. 2004; 91(2): 167-75

Aunger R, Schmidt WP, Ranpura A, Coombes Y, Maina PM, Matiko CN, Curtis V. Three kinds of psychological determinants for hand-washing behaviour in Kenya. Soc Sci Med. 2010; 70(3): 383-91

Ayah R, Joshi MD, Wanjiru R, Njau EK, Otieno CF, Njeru EK, Mutai KK. A population-based survey of prevalence of diabetes and correlates in an urban slum community in Nairobi, Kenya. BMC Public Health. 2013; 13: 371

Bill and Melinda Gates Institute for Population and Reproductive Health, Johns Hopkins Bloomberg School of Public Health, International Center for Reproductive Health (Kenya), Kenya National Bureau of Statistics, Ministry of Health (Kenya), National Council for Population and Development (Kenya). Kenya Performance, Monitoring, and Accountability Survey, Round 2 2014. Baltimore, United States: Johns Hopkins Bloomberg School of Public Health

Bill and Melinda Gates Institute for Population and Reproductive Health, Johns Hopkins Bloomberg School of Public Health, International Center for Reproductive Health (Kenya), Kenya National Bureau of Statistics, Ministry of Health (Kenya), National Council for Population and Development (Kenya), Performance Monitoring and Accountability 2020. Kenya Performance, Monitoring, and Accountability Survey, Round 3 2015. Baltimore, United States: Bill and Melinda Gates Institute for Population and Reproductive Health, Johns Hopkins Bloomberg School of Public Health

Brown I, Elliott P. SODIUM INTAKES AROUND THE WORLD [Internet]. Paris: World Health Organization;. 2006; 85

Center for Vaccine Development (Chile), Center for Vaccine Development, University of Maryland, Centers for Disease Control and Prevention (CDC), Department of Medical Microbiology and Immunology, Göteborg University, International Vaccine Institute, Kenya Medical Research Institute (KEMRI), Perry Point Cooperative Studies Program Coordinating Center, U.S. Department of Veterans Affairs, School of Medicine, University of Virginia, University of Chile. Kenya - Nyanza Global Enteric Multicenter Study 2008-2011. Baltimore, MD, United States: Center for Vaccine Development, University of Maryland

Center for Vaccine Development (Chile), Center for Vaccine Development, University of Maryland, Centers for Disease Control and Prevention (CDC), Department of Medical Microbiology and Immunology, Göteborg University, International Vaccine Institute, Kenya Medical Research Institute (KEMRI), Perry Point Cooperative Studies Program Coordinating Center, U.S. Department of Veterans Affairs, School of Medicine, University of Virginia, University of Chile. Kenya - Nyanza Global Enteric Multicenter Study 2011-2012. Baltimore, MD, United States: Center for Vaccine Development, University of Maryland

Centers for Disease Control and Prevention (CDC) and World Health Organization (WHO). Kenya Global Youth Tobacco Survey 2001. Atlanta, United States: Centers for Disease Control and Prevention (CDC)

Centers for Disease Control and Prevention (CDC) and World Health Organization (WHO). Kenya Global Youth Tobacco Survey 2007. United States: Centers for Disease Control and Prevention (CDC), 2007

Centers for Disease Control and Prevention (CDC), Central Bureau of Statistics (Kenya), Macro International, Inc, Ministry of Health (Kenya), National Council for Population and Development (Kenya). Kenya Demographic and Health Survey 2003. Calverton, United States: Macro International, Inc

Centers for Disease Control and Prevention (CDC), ICF Macro, Kenya Medical Research Institute (KEMRI), Kenya National Bureau of Statistics, Measure DHS, Ministry of Public Health and Sanitation (Kenya), Population Services International (PSI), President's Malaria Initiative (PMI), United Nations Children's Fund (UNICEF), Walter Reed Project, World Health Organization (WHO). Kenya Malaria Indicator Survey 2010. Nairobi, Kenya: Kenya National Bureau of Statistics

Centers for Disease Control and Prevention (CDC), Johns Hopkins Bloomberg School of Public Health, Kenya National Bureau of Statistics, Ministry of Health (Kenya), Research Triangle Institute, Inc. (RTI), World Health Organization (WHO). Kenya Global Adult Tobacco Survey 2014

Centers for Disease Control and Prevention (CDC), Kenya National Bureau of Statistics, United Nations Children's Fund (UNICEF). Kenya Violence Against Children Study 2010. Nairobi, Kenya: Kenya National Bureau of Statistics

Centers for Disease Control and Prevention (CDC), World Health Organization (WHO). Kenya Global School-Based Student Health Survey 2003

Central Bureau of Statistics (CBS) (Kenya), Minnesota Population Center. Kenya Population and Housing Census 1989 from the Integrated Public Use Microdata Series, International: [Machine-readable database]. Minneapolis: University of Minnesota

Central Bureau of Statistics (CBS) (Kenya), Minnesota Population Center. Kenya Population and Housing Census 1999 from the Integrated Public Use Microdata Series, International: [Machine-readable database]. Minneapolis: University of Minnesota

Central Bureau of Statistics (Kenya), Macro International, Inc, National Council for Population Development (NCPD). Kenya Demographic and Health Survey 1993. Calverton, United States: Macro International, Inc

Central Bureau of Statistics (Kenya), Macro International, Inc, National Council for Population Development (NCPD). Kenya Demographic and Health Survey 1998. Calverton, United States: Macro International, Inc

Central Bureau of Statistics (Kenya), UK Department for International Development (DFID), United States Agency for International Development (USAID), European Union (EU), Danish International Development Agency (DANIDA), World Bank (WB), United Nations Development Programme (UNDP). Kenya Integrated Household Budget Survey 2005-2006. Nairobi, Kenya: Central Bureau of Statistics (Kenya)

Central Bureau of Statistics (Kenya), UK Department for International Development (DFID), United Nations Development Programme (UNDP), United Nations Population Fund (UNFPA), United States Agency for International Development (USAID). Kenya Population and Housing Census 1999

Central Bureau of Statistics (Kenya), United Nations Children's Fund (UNICEF). Kenya Multiple Indicator Cluster Survey 2000. New York, United States: United Nations Children's Fund (UNICEF)

Central Bureau of Statistics (Kenya). Kenya Welfare Monitoring Survey III 1997. Nairobi, Kenya: Kenya National Bureau of Statistics

Erulkar AS. The Experience of Sexual Coercion among Young People in Kenya. Int Fam Plan Perspect. 2004; 30(4): 182-9

Euromonitor International. Euromonitor Passport - Cigarette Statistics. London, United Kingdom: Euromonitor International

Euromonitor International. Euromonitor Passport - Dairy Market Statistics. London, United Kingdom: Euromonitor International

Euromonitor International. Euromonitor Passport - Nuts Market Statistics. London, United Kingdom: Euromonitor International

Euromonitor International. Partially Hydrogenated Vegetable Oil Sales Database

FAO Supply Utilization Accounts 1961-2013. Personal Correspondence with Dr. Josef Schmidhuber, 2016. [Unpublished]

Food and Agriculture Organization of the United Nations (FAO). FAOSTAT Food Balance Sheets, October 2014. Rome, Italy: Food and Agriculture Organization of the United Nations (FAO)

Food and Agriculture Organization of the United Nations (FAO). FAOSTAT Food Balance Sheets, April 2015. Rome, Italy: Food and Agriculture Organization of the United Nations (FAO), 2015

Food and Agriculture Organization of the United Nations (FAO). FAOSTAT Commodity Balances - Crops Primary Equivalent. Rome, Italy: Food and Agriculture Organization of the United Nations (FAO)

Friis H, Mwaniki D, Omondi B, Muniu E, Thiong'o F, Ouma J, Magnussen P, Geissler PW, Michaelsen KF. Effects on haemoglobin of multi-micronutrient supplementation and multi-helminth chemotherapy: a randomized, controlled trial in Kenyan school children. Eur J Clin Nutr. 2003; 57(4): 573-9

Gaita SM, Boman J,Gatari MJ,Pettersson JBC,Janhäll S. Source apportionment and seasonal variation of PM 2.5 in a Sub-Saharan African city: Nairobi, Kenya . Atmos Chem Phys. 2014; 14: 9977-91 as it appears in World Health Organization (WHO). WHO Urban Ambient Air Pollution Database Draft 2016

Gewa CA, Murphy SP, Neumann CG. A comparison of weighed and recalled intakes for schoolchildren and mothers in rural Kenya. Public Health Nutr. 2009; 12(8): 1197–204

Gewa CA, Murphy SP, Weiss RE, Neumann CG. Determining minimum food intake amounts for diet diversity scores to maximize associations with nutrient adequacy: an analysis of schoolchildren’s diets in rural Kenya. Public Health Nutr. 2014; 17(12): 2667–73

Gouws E, White PJ, Stover J, Brown T. Short term estimates of adult HIV incidence by mode of transmission: Kenya and Thailand as examples. Sex Transm Infect. 2006; 82(Suppl 3): 51-55

Haregu TN, Oti S, Ngomi N, Khayeka-Wandabwa C, Egondi T, Kyobutungi C. Interlinkage among cardio-metabolic disease markers in an urban poor setting in Nairobi, Kenya. Glob Health Action. 2016; 9: 30626

ICF International, Kenya Medical Research Institute (KEMRI), Kenya National Bureau of Statistics, Ministry of Health (Kenya), National AIDS Control Council (Kenya), National Council for Population and Development (Kenya). Kenya Demographic and Health Survey 2014. Fairfax, United States: ICF International

ICF International, Kenya National Bureau of Statistics, National Malaria Control Program (NMCP) (Kenya). Kenya Malaria Indicator Survey 2015. Fairfax, United States: ICF International, 2015

ICF Macro, Kenya Medical Research Institute (KEMRI), Kenya National Bureau of Statistics, Ministry of Public Health and Sanitation (Kenya), National AIDS and STI Control Program (Kenya), National Aids Control Council (NACC), National Coordinating Agency for Population and Development (Kenya). Kenya Demographic and Health Survey 2008-2009. Calverton, United States: ICF Macro

International Labour Organization (ILO). International Labour Organization Database (ILOSTAT) - Employment to Population Ratio. International Labour Organization (ILO)
Jenson A, Omar AL, Omar MA, Rishad AS, Khoshnood K. Assessment of hypertension control in a district of Mombasa, Kenya. Glob Public Health. 2011; 6(3): 293–306
Joshi MD, Ayah R, Njau EK, Wanjiru R, Kayima JK, Njeru EK, Mutai KK. Prevalence of hypertension and associated cardiovascular risk factors in an urban slum in Nairobi, Kenya: a population-based survey. BMC Public Health. 2014; 14: 1177
Kenya Anthropometric and Micronutrient Nutrition Survey in Kakuma Camp 2001 as it appears in World Health Organization (WHO). WHO Global Database on Child Growth and Malnutrition - Historical. Geneva, Switzerland: World Health Organization (WHO)
Kenya Baseline Survey on Nutrition and Health for the Marsabit Development Program 1994 as it appears in World Health Organization (WHO). WHO Global Database on Child Growth and Malnutrition - Historical. Geneva, Switzerland: World Health Organization (WHO)
Kenya Medical Research Institute (KEMRI), Ministry of Health (Kenya), Social Sciences and Medicine Africa Network (SOMA-NET), United Nations Children's Fund (UNICEF), University of Nairobi. Kenya National Micronutrient Survey 1999
Kenya National Bureau of Statistics, Ministry of Devolution and Planning (Kenya), Ministry of Health (Kenya), National AIDS and STI Control Program (Kenya). Kenya AIDS Indicator Survey 2012-2013. Nairobi, Kenya: Kenya National Bureau of Statistics
Kenya National Bureau of Statistics, Ministry of Health (Kenya), World Health Organization (WHO). Kenya STEPS Noncommunicable Disease Risk Factors Survey 2015
Kenya National Bureau of Statistics, Population Studies and Research Institute, University of Nairobi (Kenya), United Nations Children's Fund (UNICEF). Kenya - Bungoma County Multiple Indicator Survey 2013-2014. New York, United States: United Nations Children's Fund (UNICEF), 2015
Kenya National Bureau of Statistics, Population Studies and Research Institute, University of Nairobi (Kenya), United Nations Children's Fund (UNICEF). Kenya - Kakamega County Multiple Indicator Survey 2013-2014. New York, United States: United Nations Children's Fund (UNICEF), 2015
Kenya National Bureau of Statistics, Population Studies and Research Institute, University of Nairobi (Kenya), United Nations Children's Fund (UNICEF). Kenya - Turkana County Multiple Indicator Survey 2013-2014. New York, United States: United Nations Children's Fund (UNICEF), 2015
Kenya National Bureau of Statistics, United Nations Children's Fund (UNICEF). Kenya - Eastern Province Multiple Indicator Cluster Survey 2008. Nairobi, Kenya: Kenya National Bureau of Statistics
Kenya National Bureau of Statistics, United Nations Children's Fund (UNICEF). Kenya - Coast Multiple Indicator Cluster Survey 2009. New York, United States: United Nations Children's Fund (UNICEF), 2014
Kenya National Bureau of Statistics, United Nations Children's Fund (UNICEF). Kenya - Nyanza Province Multiple Indicator Cluster Survey 2011. Nairobi, Kenya: Kenya National Bureau of Statistics
Kenya National Bureau of Statistics, United Nations Children's Fund (UNICEF). Kenya - North Eastern Province Multiple Indicator Cluster Survey 2007. Nairobi, Kenya: Kenya National Bureau of Statistics
Kenya National Bureau of Statistics, United Nations Children's Fund (UNICEF). Kenya Multiple Indicator Cluster Survey 1996

Kenya National Bureau of Statistics, USAID, United Nations Population Fund (UNFPA), United States Census Bureau. Kenya Population and Housing Census 2009

Kenya National Bureau of Statistics. Kenya Welfare Monitoring Survey II 1994

Kenya National Micronutrient Survey 2011 as it appears in Petry N, Olofin I, Hurrell RF, Boy E, Wirth JP, Moursi M, Angel MD, Rohner F. The Proportion of Anemia Associated with Iron Deficiency in Low, Medium, and High Human Development Index Countries: A Systematic Analysis of National Surveys. Nutrients. 2016; 8(11)

Kenya Nutrition and Immunization Coverage Survey in Kakuma Camp 1997 as it appears in World Health Organization (WHO). WHO Global Database on Child Growth and Malnutrition - Historical. Geneva, Switzerland: World Health Organization (WHO)

Kenya Rural Child Nutrition Survey 1987 as it appears in World Health Organization (WHO). WHO Global Database on Child Growth and Malnutrition. Geneva, Switzerland: World Health Organization (WHO)

Leenstra T, Kariuki SK, Kurtis JD, Oloo AJ, Kager PA, ter Kuile FO. Prevalence and severity of anemia and iron deficiency: cross-sectional studies in adolescent schoolgirls in western Kenya. Eur J Clin Nutr. 2004; 58(4): 681-91

Macro Systems, Inc.; Institute for Resource Development, National Council for Population Development (NCPD). Kenya Demographic and Health Survey 1988-1989. Columbia, United States: Macro Systems, Inc

Mwaniki EW, Makokha AN. Nutrition status and associated factors among children in public primary schools in Dagoretti, Nairobi, Kenya. Afr Health Sci. 2013; 13(1): 39–46

National AIDS and STI Control Program (Kenya). Kenya Most-At-Risk Populations Surveillance Report 2012. Nairobi, Kenya: National AIDS and STI Control Program (Kenya), 2012

National AIDS Control Council (Kenya), National AIDS and STI Control Program (Kenya), United Nations Office on Drugs and Crime (UNODC), University of California San Francisco. Kenya Most At Risk Populations Size Estimate Consensus Report. Nairobi, Kenya: National AIDS and STI Control Program (Kenya), 2013

Newton CR, Warn PA, Winstanley PA, Peshu N, Snow RW, Pasvol G, Marsh K. Severe anaemia in children living in a malaria endemic area of Kenya. Trop Med Int Health. 1997; 2(2): 165-78

Ngare DK, Muttunga JN, Njonge E. Vitamin A deficiency in pre-school children in Kenya. East Afr Med J. 2000; 77(8): 421-4 as it appears in World Health Organization (WHO). WHO Global Database on Vitamin A Deficiency. Geneva, Switzerland: World Health Organization (WHO)

Njoroge GK, Njagi ENM, Orinda GO, Sekadde-Kigondu CB, Kayima JK. Environmental and occupational exposure to lead. East Afr Med J. 2008; 85(6): 284-91

Okal J, Geibel S, Muraguri N, Musyoki H, Tun W, Broz D, Kuria D, Kim A, Oluoch T, Raymond HF. Estimates of the size of key populations at risk for HIV infection: men who have sex with men, female sex workers and injecting drug users in Nairobi, Kenya. Sex Transm Infect. 2013; 89(5): 366–71

Olack B, Wabwire-Mangen F, Smeeth L, Montgomery JM, Kiwanuka N, Breiman RF. Risk factors of hypertension among adults aged 35-64 years living in an urban slum Nairobi, Kenya. BMC Public Health. 2015; 15: 1251

Olewe TM, Mwanthi MA, Wang'ombe JK, Griffiths JK. Blood lead levels and potental environmental exposures among children under five years in Kibera slums, Nairobi. East Afr J Public Health. 2009; 6(1): 6-10

Olsen A, Magnussen P, Ouma JH, Andreassen J, Friis H. The contribution of hookworm and other parasitic infections to haemoglobin and iron status among children and adults in western Kenya. Trans R Soc Trop Med Hyg. 1998; 92(6): 643-9

Performance Monitoring and Accountability 2020 (PMA2020) Project, International Centre for Reproductive Health Kenya (ICRHK). 2014. Baltimore, MD: PMA2020. Bill & Melinda Gates Institute for Population and Reproductive Health, Johns Hopkins Bloomberg School of Public Health

Poulter N, Khaw KT, Hopwood BE, Mugambi M, Peart WS, Rose G, Sever PS. Blood pressure and associated factors in a rural Kenyan community. Hypertension. 1984; 6(6): 810-3

Schmidt WP, Aunger R, Coombes Y, Maina PM, Matiko CN, Biran A, Curtis V. Determinants of handwashing practices in Kenya: the role of media exposure, poverty and infrastructure. Trop Med Int Health. 2009; 14(12): 1534-41

Seedat S, Nyamai C, Njenga F, Vythilingum B, Stein DJ. Trauma exposure and post-traumatic stress symptoms in urban African schools Survey in CapeTown and Nairobi. Br J Psychiatry. 2004; 184(2): 169-75

Shulman CE, Graham WJ, Jilo H, Lowe BS, New L, Obiero J, Snow RW, Marsh K. Malaria is an important cause of anaemia in primigravidae: evidence from a district hospital in coastal Kenya. Trans R Soc Trop Med Hyg. 1996; 90(5): 535-9

Steadman Group. Kenya Formative and Baseline Survey on Handwashing with Soap 2007

Ter Kuile FO, Terlouw DJ, Phillips-Howard PA, Hawley WA, Friedman JF, Kolczak MS, Kariuki SK, Shi YP, Kwena AM, Vulule JM, Nahlen BL. Impact of permethrin-treated bed nets on malaria and all-cause morbidity in young children in an area of intense perennial malaria transmission in western Kenya: cross-sectional survey. Am J Trop Med Hyg. 2003; 68(4 Suppl): 100-7

The INTERSALT Co-operative Research Group. Appendix tables. Centre-specific results by age and sex. J Hum Hypertens 1989;3(5):331-407

U.S. Department of Agriculture (USDA). USDA Global Tobacco Database 1960-2005. Washington D.C. , United States: U.S. Department of Agriculture (USDA)

van de Vijver SJM, Oti SO, Agyemang C, Gomez GB, Kyobutungi C. Prevalence, awareness, treatment and control of hypertension among slum dwellers in Nairobi, Kenya. J Hypertens. 2013; 31(5): 1018–24

World Bank. World Development Indicators - Vitamin A Supplementation Coverage Rate. Washington DC, United States: World Bank

World Health Organization (WHO). Kenya World Health Survey 2004. Geneva, Switzerland: World Health Organization (WHO), 2005

Zucker JR, Lackritz EM, Ruebush TK, Hightower AW, Adungosi JE, Were JB, Campbell CC. Anaemia, blood transfusion practices, HIV and mortality among women of reproductive age in western Kenya. Trans R Soc Trop Med Hyg. 1994; 88(2): 173-6

Appendix Table 5 - Total all-cause all-age death rates, age-standardised death rates per 100,000, and annualised percentage change for 1990, 2006, and 2016, both sexes combined, Kenya

	All-age death rate (per 100,000)					Age-standardized death rate (per 100,000)				
	**1990**	**2006**	**2016**	**Annualized percent change 1990- 2006**	**Annualized percent change 2006-2016**	**1990**	**2006**	**2016**	**Annualised percent change 1990- 2006**	**Annualised percent change 2006-2016**
All-cause mortality	850·31 (829·76 to 871·12)	902·89 (881·64 to 924·12)	579·00 (562·15 to 595·96)	0·38 (0·36 to 0·38)	-4·44 (-5·68 to -3·44)	1,446·9 (1,402·9 to 1,493·5)	1,645·4 (1,604·7 to 1,690·5)	1,181·96 (1,142·34 to 1,221)	0·8 (0·7 to ·08)	-3·31 (-2·04 to -4·05)
HIV/AIDS-specific mortality	69·53 (59·04 to 82·5)	282·36 (268·87 to 294·9)	90·07 (84·63 to 95·58)	4·71 (4·50 to 4·88)	-21·35 (-21·77 to -20·85)	94·98 (79·61 to 113·96)	397·64 (382·28 to 412·89)	107·5 (101·96 to 113·53)	4·76 (4·52 to 4·95)	-27·0 (-27·49 to -26·37)

Appendix Table 6 - All-cause mortality rate, HIV/AIDS-specific mortality rate, and percent change for 1990, 2006 and 2016, both sexes combined, Kenya and counties

	All-cause age-standardized mortality rate (per 100,000)					HIV/AIDS-specific age-standardized mortality rate (per 100,000)				
	1990	2006	2016	Annualised percent change 1990-2006	Annualised percent change 2006-2016	1990	2006	2016	Annualised percent change 1990-2006	Annualised percent change 2006-2016
Baringo	1650 (1380 to 1920)	1560 (1360 to 1790)	1120 (911 to 1340)	-0.4 (-1.6 to 0.8)	-3.3 (-5.3 to -1.3)	99 (36.5 to 198)	363 (305 to 433)	100 (78.4 to 129)	8.1 (4.3 to 13.9)	-12.9 (-14.6 to -10.5)
Bomet	1120 (939 to 1310)	1160 (987 to 1370)	928 (780 to 1130)	0.2 (-1.2 to 1.5)	-2.2 (-4.2 to -0.2)	78.6 (26.2 to 175)	312 (252 to 382)	89.6 (69.9 to 114)	8.6 (4.1 to 15.1)	-12.5 (-14.1 to -10.1)
Bungoma	1320 (1050 to 1600)	1250 (1050 to 1500)	994 (814 to 1220)	-0.3 (-1.7 to 1.2)	-2.3 (-4.2 to -0.5)	122 (53 to 230)	356 (282 to 436)	92.3 (67.5 to 125)	6.7 (3.3 to 11.7)	-13.5 (-16 to -11.4)
Busia	1500 (1200 to 1810)	1690 (1350 to 2100)	1300 (1020 to 1600)	0.7 (-0.8 to 2.3)	-2.6 (-4.8 to -0.6)	124 (57.8 to 227)	392 (318 to 482)	99 (71.3 to 131)	7.2 (3.8 to 11.7)	-13.8 (-16.4 to -11.8)
Elgeyo-Marakwet	1530 (1280 to 1770)	1570 (1340 to 1790)	1230 (999 to 1490)	0.1 (-1 to 1.4)	-2.4 (-4.3 to -0.3)	93 (29.9 to 196)	342 (282 to 410)	96.9 (74.1 to 125)	8.1 (4 to 14.6)	-12.6 (-14.4 to -10.3)
Embu	1150 (949 to 1360)	1330 (1060 to 1620)	1000 (790 to 1260)	0.9 (-0.6 to 2.5)	-2.8 (-5.1 to -0.6)	29.6 (7.08 to 85.4)	238 (188 to 302)	70.9 (51.9 to 95.6)	13.0 (6.8 to 21.8)	-12.1 (-14.4 to -9.6)
Garissa	1560 (1330 to 1810)	1620 (1390 to 1890)	1310 (1090 to 1570)	0.2 (-1 to 1.4)	-2.1 (-3.8 to -0.4)	14.9 (7.21 to 26.7)	67.7 (54.9 to 81.8)	37.4 (30.1 to 47.4)	9.4 (5.8 to 14.5)	-5.9 (-7.6 to -4.1)
Homa Bay	2120 (1800 to 2500)	2920 (2540 to 3290)	1860 (1470 to 2220)	2.0 (0.9 to 3.2)	-4.5 (-6.8 to -2.5)	123 (51 to 262)	923 (782 to 1100)	222 (169 to 280)	12.6 (8.4 to 17.7)	-14.2 (-16.7 to -11.3)
Isiolo	1580 (1370 to 1820)	1610 (1290 to 1980)	1290 (1010 to 1580)	0.1 (-1.4 to 1.4)	-2.2 (-4.4 to 0)	39.7 (10.3 to 104)	261 (201 to 330)	79.8 (55.8 to 113)	11.8 (6.1 to 20)	-11.9 (-14.6 to -9.1)
Kajiado	1430 (1150 to 1700)	1610 (1390 to 1880)	1180 (949 to 1420)	0.7 (-0.7 to 2.2)	-3.1 (-5.3 to -1)	103 (39 to 206)	383 (324 to 454)	105 (77.3 to 135)	8.2 (4.3 to 14)	-12.9 (-15.5 to -10.5)
Kakamega	1450 (1190 to 1740)	1640 (1400 to 1900)	1230 (1010 to 1480)	0.8 (-0.5 to 2)	-2.8 (-4.5 to -1)	137 (62.4 to 241)	401 (325 to 487)	104 (71.3 to 141)	6.7 (3.6 to 11.2)	-13.5 (-16.8 to -11.2)
Kericho	1210 (956 to 1480)	1300 (1090 to 1630)	920 (675 to 1180)	0.4 (-1 to 2.2)	-3.5 (-5.8 to -1)	89.4 (34.1 to 189)	350 (289 to 421)	105 (78.1 to 143)	8.5 (4.3 to 14.2)	-12.1 (-14.2 to -9.4)
Kiambu	1410 (1170 to 1640)	1690 (1490 to 1880)	1150 (924 to 1340)	1.1 (-0.1 to 2.3)	-3.8 (-5.8 to -2.2)	60.3 (20.7 to 128)	292 (240 to 353)	101 (73.9 to 137)	9.9 (5.5 to 16.1)	-10.6 (-12.8 to -8.6)
Kilifi	1480 (1170 to 1800)	1610 (1330 to 1910)	1130 (937 to 1320)	0.6 (-0.9 to 2.1)	-3.5 (-5.7 to -1.4)	126 (35.5 to 278)	388 (300 to 477)	103 (77 to 132)	7.0 (2.7 to 14.2)	-13.3 (-15.3 to -10.8)
Kirinyaga	1230 (978 to 1510)	1510 (1190 to 1910)	1100 (817 to 1390)	1.3 (-0.3 to 2.9)	-3.2 (-5.6 to -0.8)	55.5 (17.7 to 119)	272 (219 to 335)	97 (67.4 to 132)	9.9 (5.5 to 16.5)	-10.3 (-12.6 to -8.1)
Kisii	1360 (1090 to 1700)	1890 (1650 to 2150)	1140 (894 to 1390)	2.0 (0.5 to 3.4)	-5.0 (-7.5 to -2.8)	127 (42.5 to 270)	808 (660 to 976)	186 (144 to 241)	11.6 (7.4 to 18)	-14.7 (-16.8 to -11.4)
Kisumu	1690 (1370 to 2030)	2450 (2160 to 2800)	1540 (1220 to 1880)	2.3 (1.1 to 3.5)	-4.6 (-7.2 to -2.3)	135 (47.1 to 292)	878 (723 to 1050)	229 (179 to 292)	11.7 (7.3 to 17.8)	-13.4 (-15.5 to -10.4)
Kitui	1510 (1300 to 1690)	1390 (1210 to 1600)	1090 (898 to 1290)	-0.5 (-1.6 to 0.8)	-2.5 (-4.4 to -0.7)	32.1 (9.36 to 84.2)	254 (206 to 319)	68.4 (51.7 to 93.3)	12.9 (7 to 20.6)	-13.1 (-15.2 to -10.8)
Kwale	1560 (1200 to 1930)	1680 (1400 to 1970)	1240 (1000 to 1490)	0.4 (-1 to 2.2)	-3.0 (-5.2 to -1)	143 (48.4 to 299)	409 (342 to 487)	116 (93 to 147)	6.6 (2.4 to 13)	-12.6 (-14.2 to -10.5)
Laikipia	1170 (873 to 1450)	1340 (1060 to 1690)	899 (687 to 1180)	0.8 (-0.8 to 2.7)	-4.0 (-6.3 to -1.5)	87.8 (27 to 190)	347 (275 to 417)	97.5 (74.9 to 123)	8.6 (4.3 to 15.7)	-12.7 (-14.6 to -10.3)
Lamu	1470 (1080 to 1890)	1630 (1300 to 2060)	1220 (960 to 1520)	0.7 (-1.1 to 2.6)	-2.9 (-5.2 to -0.7)	146 (45 to 308)	409 (343 to 486)	112 (86.3 to 146)	6.4 (2.2 to 13.4)	-12.9 (-15.2 to -10.7)
Machakos	1220 (1020 to 1390)	1340 (1140 to 1550)	1060 (871 to 1230)	0.6 (-0.7 to 1.9)	-2.4 (-4.3 to -0.6)	31.8 (7.42 to 89.4)	234 (181 to 301)	65.4 (47.6 to 89.9)	12.5 (6.6 to 21.5)	-12.7 (-14.9 to -10.6)
Makueni	1430 (1220 to 1670)	1480 (1260 to 1700)	1120 (877 to 1340)	0.2 (-1.1 to 1.5)	-2.8 (-5.1 to -1)	38.8 (11 to 90.5)	265 (218 to 328)	81.5 (60.9 to 110)	12.0 (6.8 to 20)	-11.8 (-14.1 to -9)
Mandera	1630 (1400 to 1880)	1660 (1470 to 1890)	1400 (1180 to 1650)	0.1 (-0.9 to 1.2)	-1.7 (-3.1 to -0.2)	19.1 (9.99 to 32.8)	75 (61 to 89.2)	44.4 (36.7 to 53.2)	8.6 (5.2 to 13)	-5.2 (-6.9 to -3.5)
Marsabit	1460 (1200 to 1760)	1650 (1340 to 2010)	1330 (1070 to 1600)	0.8 (-0.6 to 2.3)	-2.2 (-4 to -0.4)	34.3 (9.22 to 91.2)	240 (185 to 306)	71.1 (51 to 98.9)	12.1 (6.2 to 20.4)	-12.1 (-14.5 to -9.7)
Meru	1330 (1140 to 1520)	1450 (1220 to 1670)	1190 (1010 to 1400)	0.5 (-0.8 to 1.7)	-1.9 (-3.6 to -0.2)	34.4 (9.12 to 92.4)	252 (201 to 314)	74.6 (54.8 to 101)	12.4 (6.5 to 21)	-12.2 (-14.3 to -10)
Migori	1860 (1550 to 2200)	2480 (2190 to 2850)	1610 (1270 to 1950)	1.8 (0.6 to 3.1)	-4.3 (-6.7 to -2.1)	129 (52.6 to 283)	906 (755 to 1080)	220 (176 to 273)	12.2 (7.9 to 17.7)	-14.2 (-16.1 to -11.2)
Mombasa	1350 (1010 to 1680)	1530 (1210 to 1870)	1040 (834 to 1270)	0.8 (-1 to 2.6)	-3.9 (-6.2 to -1.8)	137 (41.6 to 308)	388 (322 to 468)	110 (83.9 to 140)	6.5 (2.1 to 13.8)	-12.6 (-14.5 to -10.4)
Murang'a	1120 (871 to 1340)	1370 (1200 to 1550)	968 (796 to 1160)	1.3 (0 to 2.8)	-3.5 (-5.4 to -1.7)	55 (18.3 to 123)	278 (229 to 336)	96.2 (69.4 to 127)	10.1 (5.5 to 16.9)	-10.6 (-12.8 to -9)
Nairobi	1310 (1000 to 1640)	1640 (1380 to 1880)	1100 (878 to 1300)	1.4 (-0.2 to 3.3)	-4.0 (-6.4 to -2)	167 (60.5 to 327)	438 (366 to 511)	105 (76.7 to 135)	6.0 (2.0 to 12.3)	-14.3 (-16.9 to -12.3)
Nakuru	1390 (1140 to 1610)	1390 (1220 to 1600)	899 (719 to 1080)	0.0 (-1.1 to 1.2)	-4.3 (-6.2 to -2.7)	90 (26.8 to 193)	340 (279 to 407)	99.7 (75.9 to 128)	8.3 (4.1 to 15.3)	-12.3 (-14 to -10.2)
Nandi	1380 (1140 to 1630)	1460 (1270 to 1670)	1070 (875 to 1280)	0.3 (-1 to 1.6)	-3.1 (-5.2 to -1.3)	95.7 (32.7 to 197)	344 (287 to 414)	98.8 (78.1 to 124)	8.0 (4 to 14.4)	-12.5 (-14.1 to -10.2)
Narok	1720 (1490 to 1960)	1760 (1490 to 2080)	1310 (1090 to 1550)	0.2 (-1.1 to 1.3)	-3 (-5 to -1.1)	89.5 (32.6 to 189)	367 (297 to 443)	103 (82.3 to 128)	8.8 (4.6 to 14.7)	-12.7 (-14.1 to -10.6)
Nyamira	1330 (991 to 1650)	2280 (2000 to 2650)	1530 (1230 to 1840)	3.4 (2 to 5)	-4 (-6.5 to -1.9)	123 (42.6 to 274)	851 (699 to 1020)	205 (162 to 259)	12.1 (7.6 to 18.3)	-14.3 (-16.3 to -11.5)
Nyandarua	1430 (1190 to 1680)	1790 (1580 to 2030)	1450 (1170 to 1700)	1.4 (0.2 to 2.5)	-2.1 (-4.1 to -0.4)	60.4 (22.6 to 123)	303 (240 to 365)	111 (81.2 to 143)	10.1 (6 to 15.9)	-10.1 (-12.3 to -8.4)
Nyeri	1530 (1260 to 1810)	1640 (1440 to 1890)	1270 (1050 to 1490)	0.5 (-0.8 to 1.8)	-2.6 (-4.5 to -0.8)	56 (17.1 to 119)	296 (248 to 354)	98.8 (73 to 129)	10.4 (6 to 17.5)	-11 (-13.1 to -9.1)
Samburu	1420 (1080 to 1730)	1600 (1250 to 1940)	1320 (1060 to 1610)	0.7 (-0.7 to 2.3)	-1.9 (-4 to 0.3)	101 (29.6 to 206)	357 (286 to 438)	99.6 (74.3 to 129)	7.9 (4.1 to 14.7)	-12.8 (-14.8 to -10.3)
Siaya	1730 (1430 to 2080)	2310 (1980 to 2660)	1190 (945 to 1420)	1.8 (0.5 to 3)	-6.7 (-9.1 to -4.4)	118 (42 to 255)	823 (674 to 996)	179 (140 to 232)	12.1 (7.8 to 18)	-15.3 (-17.3 to -12.2)
Taita-Taveta	1560 (1240 to 1930)	1780 (1490 to 2100)	1410 (1170 to 1650)	0.8 (-0.6 to 2.4)	-2.3 (-4.4 to -0.3)	130 (39.2 to 283)	412 (343 to 491)	109 (82.1 to 141)	7.2 (3 to 14.4)	-13.3 (-15.8 to -10.7)
Tana River	1940 (1490 to 2380)	2170 (1790 to 2570)	1700 (1400 to 2010)	0.7 (-0.7 to 2.4)	-2.5 (-4.4 to -0.4)	138 (43.9 to 319)	438 (362 to 526)	128 (96 to 165)	7.2 (2.7 to 13.8)	-12.3 (-14.2 to -10.2)

Tharaka-Nithi	1690<br>(1420 to 1970)	1600<br>(1370 to 1860)	1220<br>(990 to 1450)	-0.3<br>(-1.6 to 1)	-2.7<br>(-4.6 to -0.9)	39.2<br>(9.53 to 93.4)	279<br>(226 to 347)	83.5<br>(62 to 108)	12.3<br>(6.9 to 21.2)	-12.1<br>(-14.5 to -9.8)
Trans Nzoia	1140<br>(854 to 1440)	1280<br>(1050 to 1590)	970<br>(761 to 1260)	0.7<br>(-0.8 to 2.7)	-2.8<br>(-5.1 to -0.4)	95.6<br>(31.5 to 200)	351<br>(291 to 420)	101<br>(78.6 to 127)	8.1<br>(4.2 to 14.8)	-12.4<br>(-14.2 to -10)
Turkana	1670<br>(1430 to 1940)	1840<br>(1560 to 2140)	1430<br>(1200 to 1710)	0.6<br>(-0.6 to 1.7)	-2.5<br>(-4.2 to -0.7)	104<br>(41.1 to 202)	369<br>(306 to 439)	102<br>(82.1 to 129)	7.9<br>(4.3 to 13)	-12.8<br>(-14.4 to -10.6)
Uasin Gishu	1200<br>(977 to 1420)	1230<br>(1080 to 1430)	896<br>(731 to 1090)	0.2<br>(-1.1 to 1.6)	-3.2<br>(-5.4 to -1.1)	90.5<br>(32.8 to 189)	339<br>(281 to 405)	96.3<br>(74.9 to 122)	8.3<br>(4.2 to 14.4)	-12.6<br>(-14.4 to -10.2)
Vihiga	1600<br>(1230 to 1930)	1730<br>(1460 to 2130)	1390<br>(1100 to 1700)	0.5<br>(-1.1 to 2.1)	-2.2<br>(-4.4 to -0.1)	119<br>(51.2 to 224)	407<br>(336 to 491)	108<br>(76.1 to 146)	7.7<br>(4.1 to 12.7)	-13.3<br>(-16.3 to -11)
Wajir	1590<br>(1360 to 1850)	1750<br>(1530 to 1990)	1460<br>(1220 to 1740)	0.6<br>(-0.4 to 1.7)	-1.8<br>(-3.3 to -0.3)	19.5<br>(11.2 to 32.4)	77.7<br>(62.6 to 93.1)	50.1<br>(39 to 62.9)	8.6<br>(5.4 to 12.7)	-4.4<br>(-6.5 to -2.4)
West Pokot	1630<br>(1330 to 1910)	1580<br>(1320 to 1870)	1190<br>(956 to 1420)	-0.2<br>(-1.5 to 1.4)	-2.8<br>(-4.8 to -1)	101<br>(36.5 to 200)	362<br>(298 to 433)	100<br>(74 to 126)	8.0<br>(4.1 to 13.9)	-12.8<br>(-14.6 to -10.7)

Appendix Table 7 - Under-5 mortality rate, maternal mortality rate, and annualised percent change for 1990, 2006 and 2016, both sexes combined, Kenya and counties										
	Under-5 mortality rate (per 1,000 live births)					Maternal mortality rate (per 100,000 live births)				
	**1990**	**2006**	**2016**	**Percent change 1990-2006**	**Percent change 2006-2016**	**1990**	**2006**	**2016**	**Percent change 1990- 2006**	**Percent Change 2006-2016**
**Kenya**	95.42<br>90.12 to 101.34	70.1<br>64.34 to 76.62	43.44<br>36.88 to 51.17	-1.9<br>(-2.6 to -1.3)	-4.8<br>(-6.6 to -3.1)	315.72<br>(242.91 to 399.41)	341.71<br>(271.98 to 422.83)	257.64<br>(195.09 to 335.32)	0.5<br>(-0.4 to 1.6)	-2.8<br>(-4.5 to -1.3)
Baringo	155.56<br>142.9 to 169.01	67.89<br>61.37 to 74.87	35.04<br>28.45 to 42.55	-5.2<br>(-6 to -4.4)	-6.7<br>(-8.7 to -4.6)	131.08<br>(77.83 to 203.17)	98.43<br>(56.4 to 168.67)	55.48<br>(27.63 to 111.11)	-1.8<br>(-5 to 1.7)	-5.7<br>(-11 to -0.6)
Bomet	30.48<br>27.36 to 33.71	36.08<br>30.78 to 41.77	33.75<br>28.8 to 39.6	1.0<br>(-0.2 to 2.3)	-0.7<br>(-2.4 to 1)	95.86<br>(54.98 to 163.47)	86.1<br>(49.69 to 156.3)	80.39<br>(42.11 to 155.31)	-0.7<br>(-3.9 to 2.8)	-0.7<br>(-6.2 to 4.5)
Bungoma	101.26<br>88.02 to 114.68	62.36<br>56.01 to 68.44	45.06<br>39.66 to 51.21	-3.0<br>(-4.1 to -2)	-3.3<br>(-4.8 to -1.6)	370.07<br>(213.82 to 568.95)	283.06<br>(170.59 to 510.45)	321.3<br>(176.22 to 534.04)	-1.7<br>(-5.2 to 2.4)	1.3<br>(-3.5 to 5.7)
Busia	130.8<br>111.59 to 151.02	107.27<br>94.54 to 121.46	65.94<br>57.13 to 75.93	-1.2<br>(-2.6 to 0.1)	-4.9<br>(-6.7 to -3.1)	319.25<br>(184.86 to 497.94)	334.63<br>(163.65 to 577.48)	314.97<br>(173.33 to 525.35)	0.3<br>(-3.8 to 4.0)	-0.6<br>(-5.4 to 4.8)
Elgeyo-Marakwet	83.47<br>76.35 to 91.7	51.36<br>45.98 to 56.98	25.74<br>20.79 to 31.43	-3.0<br>(-3.9 to -2.1)	-7.0<br>(-9 to -4.9)	141.8<br>(82.75 to 224.87)	128.83<br>(68.93 to 216.85)	128.05<br>(61.95 to 217.21)	-0.6<br>(-4.0 to 2.8)	-0.1<br>(-5 to 5.0)
Embu	60.37<br>53.68 to 67.34	53.16<br>46.06 to 60.86	40.93<br>32.81 to 50.51	-0.8<br>(-1.9 to 0.3)	-2.7<br>(-4.9 to -0.3)	74.46<br>(43.64 to 127.99)	121.41<br>(61.33 to 212.43)	72.71<br>(33.6 to 142.57)	3.1<br>(-0.4 to 6.4)	-5.1<br>(-11.2 to 0.0)
Garissa	116.69<br>99.75 to 133.96	66.06<br>49.79 to 85.39	35.59<br>28.74 to 42.99	-3.6<br>(-5.6 to -1.7)	-6.1<br>(-8.7 to -3.9)	340.78<br>(205.59 to 506.76)	313.49<br>(183.13 to 459.14)	237.49<br>(135.17 to 374.87)	-0.5<br>(-2.7 to 1.9)	-2.8<br>(-6.3 to 0.7)
Homa Bay	194.58<br>179.56 to 210.22	158.55<br>142.93 to 174.28	77.25<br>67.61 to 87.83	-1.3<br>(-2.2 to -0.3)	-7.2<br>(-9 to -5.4)	549.56<br>(343.4 to 803.06)	662.95<br>(420.65 to 1020.17)	410.42<br>(210.12 to 662.22)	1.2<br>(-1.7 to 4.3)	-4.8<br>(-10.7 to 0.6)
Isiolo	212.62<br>193.3 to 234.19	99.89<br>88.1 to 112.02	49.44<br>42.08 to 58.29	-4.7<br>(-5.6 to -3.9)	-7.0<br>(-8.6 to -5.5)	214.45<br>(124.58 to 350.52)	237.41<br>(130.29 to 380.72)	148.77<br>(77.86 to 250.57)	0.6<br>(-2.7 to 4.2)	-4.7<br>(-9.4 to 0.3)
Kajiado	52.39<br>46.62 to 58.41	52.65<br>44.64 to 61.53	27.4<br>22.14 to 33.44	0.0<br>(-1.1 to 1.1)	-6.6<br>(-9 to -4.3)	157.38<br>(88.01 to 252.64)	185.24<br>(107.13 to 298.35)	121.9<br>(57.56 to 201.02)	1.0<br>(-3.0 to 4.6)	-4.2<br>(-11.1 to 1.6)
Kakamega	131.21<br>118.65 to 145.06	106.53<br>96.56 to 118.01	62.45<br>50.44 to 76.47	-1.3<br>(-2.3 to -0.4)	-5.4<br>(-7.7 to -3.2)	406.4<br>(240.01 to 616.88)	418.36<br>(236.59 to 689.55)	455.41<br>(234.85 to 738.5)	0.2<br>(-3.4 to 3.4)	0.8<br>(-4.5 to 5.9)
Kericho	48.08<br>43.89 to 52.74	49.79<br>44.77 to 55.14	35.84<br>29.27 to 43.27	0.2<br>(-0.6 to 1.1)	-3.3<br>(-5.4 to -1.3)	168.77<br>(100.74 to 265.29)	170.3<br>(105.41 to 289.71)	110.36<br>(49.79 to 215.75)	0.1<br>(-2.5 to 3.1)	-4.3<br>(-10.2 to 0.7)
Kiambu	44.47<br>37.97 to 51.67	42.89<br>38.17 to 48.13	27.81<br>23.71 to 32.53	-0.2<br>(-1.3 to 0.9)	-4.3<br>(-6.1 to -2.4)	234.22<br>(138.05 to 359.1)	326.25<br>(205.6 to 470.57)	195.36<br>(98.86 to 325.06)	2.1<br>(-0.9 to 5.2)	-5.1<br>(-10.5 to -1.0)
Kilifi	132.08<br>120.96 to 143.69	80.29<br>72.86 to 88.57	44.43<br>39.23 to 50.35	-3.1<br>(-3.9 to -2.3)	-5.9<br>(-7.3 to -4.5)	416.65<br>(241.47 to 616.98)	387.56<br>(248.34 to 598.19)	217.13<br>(128.74 to 322.39)	-0.5<br>(-3.2 to 2.5)	-5.8<br>(-10.8 to -1.4)
Kirinyaga	60.71<br>48.14 to 74.68	56.12<br>49.35 to 63.29	39.9<br>34.03 to 46.62	-0.5<br>(-2.1 to 1.3)	-3.4<br>(-5.3 to -1.5)	168.58<br>(95.22 to 274.7)	370.61<br>(197.8 to 636.36)	226.21<br>(99.75 to 419.15)	4.9<br>(1.5 to 8.4)	-4.9<br>(-10.7 to 0.0)
Kisii	120.24<br>106.8 to 134.41	65.72<br>57.64 to 75.29	37.68<br>27.23 to 51.01	-3.8<br>(-5 to -2.6)	-5.7<br>(-9.3 to -2.0)	383.41<br>(206.36 to 583.24)	478.11<br>(311.69 to 760.88)	347.72<br>(143 to 602.92)	1.4<br>(-1.6 to 5.0)	-3.2<br>(-11 to 1.6)
Kisumu	151.6<br>137.6 to 167.73	111.16<br>97.69 to 125.66	58.69<br>42.48 to 79.24	-1.9<br>(-3.1 to -0.9)	-6.5<br>(-9.5 to -3.4)	300.61<br>(157.97 to 469.51)	373.38<br>(234.82 to 567.25)	224.5<br>(95.99 to 409.34)	1.4<br>(-1.9 to 5.2)	-5.1<br>(-12 to 0.9)
Kitui	78.69<br>69.35 to 88.75	57.74<br>51.39 to 64.44	31.7<br>25.38 to 39.17	-1.9<br>(-2.9 to -1.0)	-6.0<br>(-8.6 to -3.5)	329.57<br>(212.57 to 488.45)	197<br>(136.28 to 317.18)	131.22<br>(71.47 to 244.1)	-3.2<br>(-6 to -0.4)	-4.1<br>(-8.8 to 0.1)
Kwale	126.3<br>112.7 to 141.79	77.9<br>68.64 to 87.65	44.65<br>35.27 to 55.51	-3.0<br>(-4.1 to -2.0)	-5.6<br>(-7.6 to -3.5)	599.83<br>(373.15 to 854.76)	367.64<br>(251.18 to 546.96)	252.08<br>(141.52 to 417.25)	-3.1<br>(-5.4 to 0.1)	-3.8<br>(-9.3 to 0.9)
Laikipia	48.14<br>44.3 to 52.36	52.22<br>43.96 to 61.05	25<br>20.19 to 30.52	0.5<br>(-0.4 to 1.4)	-7.4<br>(-9.9 to -5.1)	167.61<br>(97.46 to 262.96)	217.37<br>(114.88 to 395.52)	109.02<br>(50.67 to 203.5)	1.6<br>(-1.6 to 5.0)	-6.9<br>(-12.3 to -2.0)
Lamu	138.78<br>126.92 to 151.31	88.42<br>79.46 to 98	59.22<br>51.81 to 67.61	-2.8<br>(-3.5 to -2.1)	-4.0<br>(-5.3 to -2.7)	222.55<br>(121.33 to 348.14)	231.73<br>(136.09 to 379.37)	159.15<br>(80.5 to 258.56)	0.3<br>(-3 to 3.8)	-3.8<br>(-9.6 to 0.7)
Machakos	61.1<br>53.99 to 68.44	51.68<br>43.41 to 60.88	32.45<br>25.48 to 40.79	-1.1<br>(-2.3 to 0.2)	-4.7<br>(-7.9 to -1.6)	349.7<br>(241.9 to 500.92)	424.66<br>(271.81 to 634.29)	315.55<br>(184.15 to 490.36)	1.2<br>(-1.3 to 3.8)	-3.0<br>(-7.2 to 0.9)
Makueni	87.03<br>76.49 to 98.57	54.08<br>46.77 to 61.52	31.85<br>26.46 to 38.74	-3.0<br>(-4.1 to -1.9)	-5.3<br>(-7.2 to -3.3)	198.85<br>(126.81 to 300.47)	168.41<br>(110.67 to 265.32)	108.93<br>(52.47 to 213.3)	-1.0<br>(-3.7 to 1.8)	-4.4<br>(-10.1 to 0.2)
Mandera	130.17<br>113.36 to 147.12	76.82<br>57.95 to 99.43	57.76<br>38.74 to 81.87	-3.3<br>(-5.4 to -1.3)	-2.9<br>(-6.1 to 0.1)	348.9<br>(217.44 to 532.91)	321.25<br>(201.39 to 471.38)	252.74<br>(145.84 to 392.29)	-0.5<br>(-2.5 to 1.9)	-2.4<br>(-6.2 to 1.1)
Marsabit	103.87<br>93.35 to 115.95	61.54<br>53.85 to 69.5	36.66<br>31.1 to 43.43	-3.3<br>(-4.2 to -2.3)	-5.2<br>(-6.8 to -3.6)	130.68<br>(77.28 to 209.28)	125.81<br>(70.36 to 215.06)	74.15<br>(37.82 to 132.37)	-0.2<br>(-3.2 to 3.1)	-5.3<br>(-10.2 to -0.5)
Meru	52.69<br>47.36 to 58.74	54.45<br>48.20 to 60.99	53.51<br>45.39 to 62.61	0.2<br>(-0.7 to 1.2)	-0.2<br>(-1.8 to 1.5)	408.37<br>(268.68 to 591.58)	426.75<br>(269.83 to 657.29)	384.29<br>(217.22 to 584.35)	0.3<br>(-2.3 to 2.9)	-1.0<br>(-5.1 to 2.7)
Migori	251.73<br>226.64 to 278.47	152.6<br>137.18 to 168.73	80.94<br>66.83 to 95.64	-3.1<br>(-4.2 to -2.2)	-6.4<br>(-7.8 to -5.0)	303.65<br>(167.97 to 478.05)	438.28<br>(280.29 to 658.79)	270.5<br>(140.61 to 436.14)	2.3<br>(-1.0 to 6.3)	-4.8<br>(-11.3 to -0.1)
Mombasa	85.04<br>74.7 to 96.57	62.88<br>53.4 to 73.23	44.68<br>33.95 to 57.63	-1.9<br>(-3.1 to -0.6)	-3.5<br>(-6.8 to -0.2)	297.83<br>(180.02 to 424.02)	303.19<br>(182.99 to 482.41)	177.6<br>(93.45 to 282.18)	0.1<br>(-2.9 to 3.3)	-5.3<br>(-10.5 to -0.8)
Murang'a	39.01<br>33.12 to 45.48	47.46<br>40.55 to 55.11	36.46<br>30.99 to 42.76	1.2<br>(0.1 to 2.4)	-2.6<br>(-4.9 to -0.5)	163.37<br>(87.83 to 290.01)	287.13<br>(185.69 to 447.9)	222.54<br>(111.05 to 409.08)	3.5<br>(0.4 to 7.2)	-2.5<br>(-7.9 to 2.0)
Nairobi	64.99<br>59.55 to 71.02	43.41<br>39.05 to 48.19	20.7<br>17.46 to 24.45	-2.5<br>(-3.4 to -1.6)	-7.4<br>(-9.3 to -5.4)	574.98<br>(366.96 to 792.82)	676.15<br>(380.76 to 1072.53)	523.78<br>(251.86 to 843.8)	1.0<br>(-2.5 to 4.3)	-2.6<br>(-9.5 to 3.5)
Nakuru	47.5<br>42.62 to 52.55	63.69<br>57.64 to 70.38	57.01<br>46.48 to 68.92	1.8<br>(1.0 to 2.8)	-1.1<br>(-3.3 to 1.0)	259.05<br>(165.26 to 380.1)	246.15<br>(155.66 to 393.75)	181.97<br>(83.33 to 333)	-0.3<br>(-3.2 to 2.6)	-3.0<br>(-9.2 to 1.5)

Nandi	55.95<br>51.06 to 61.4	41.53<br>37.33 to 45.98	27.51<br>22.41 to 33.29	-1.9<br>(-2.7 to -1.0)	-4.2<br>(-6.2 to -2.1)	186.29<br>(119.99 to 290.87)	124.05<br>(78.1 to 210.75)	125.1<br>(62.57 to 231.18)	-2.5<br>(-5.3 to 0.3)	0.1<br>(-5.5 to 4.2)
Narok	78.89<br>72.07 to 86.16	46.77<br>42.31 to 51.62	28.69<br>23.32 to 34.79	-3.3<br>(-4.1 to -2.4)	-4.9<br>(-7.0 to -2.9)	288.7<br>(176.21 to 438.16)	293.12<br>(152.49 to 471.03)	165.9<br>(91.47 to 280.29)	0.1<br>(-3.5 to 3.4)	-5.7<br>(-11.1 to 0.2)
Nyamira	58.39<br>52.61 to 64.4	51.64<br>45.57 to 57.54	32.81<br>28.49 to 37.72	-0.8<br>(-1.8 to 0.3)	-4.5<br>(-6.4 to -2.7)	214.4<br>(116.33 to 340.97)	433.33<br>(252.98 to 839.89)	386.33<br>(192.2 to 621.16)	4.4<br>(0.8 to 9.2)	-1.1<br>(-8.0 to 4.4)
Nyandarua	42.33<br>33.87 to 52.88	59.38<br>52.73 to 66.66	51.12<br>42.54 to 61.15	2.1<br>(0.7 to 3.6)	-1.5<br>(-3.7 to 0.9)	173.26<br>(93.5 to 292.93)	300.22<br>(179.11 to 478.14)	220.95<br>(111.83 to 379.17)	3.4<br>(0.4 to 7)	-3.1<br>(-8.3 to 1.2)
Nyeri	40.22<br>32.67 to 48.88	51.74<br>46.26 to 58.06	39.76<br>33.83 to 46.59	1.6<br>(0.0 to 3.3)	-2.7<br>(-4.4 to -0.8)	555.46<br>(323.06 to 831.03)	913.54<br>(579.49 to 1366.62)	739.31<br>(448.12 to 1113.63)	3.1<br>(-0.1 to 6.4)	-2.1<br>(-7.2 to 2.6)
Samburu	89.62<br>80.42 to 99.66	52.46<br>46.63 to 58.77	29.92<br>23.26 to 37.97	-3.3<br>(-4.3 to -2.3)	-5.7<br>(-8.2 to -3.2)	111<br>(60.55 to 183.31)	98.78<br>(46.41 to 176.52)	78.39<br>(41.44 to 138.8)	-0.7<br>(-4.4 to 2.7)	-2.3<br>(-7.7 to 3.3)
Siaya	209.45<br>184.8 to 234.93	158.88<br>142.26 to 176.84	74.25<br>60.93 to 90.14	-1.7<br>(-2.8 to -0.5)	-7.6<br>(-10.1 to -5.1)	386.57<br>(202.18 to 632.58)	460.78<br>(229.48 to 776.57)	202.38<br>(92.37 to 343.4)	1.1<br>(-2.6 to 4.9)	-8.2<br>(-14.5 to -2.4)
Taita-Taveta	84.48<br>77.33 to 92	64.24<br>57.02 to 71.88	37.67<br>30.26 to 46.15	-1.7<br>(-2.5 to -0.8)	-5.4<br>(-7.0 to -3.6)	405.77<br>(244.6 to 617.84)	529.4<br>(340.19 to 847.57)	555.46<br>(330.49 to 842.86)	1.7<br>(-1.5 to 4.8)	0.5<br>(-4.9 to 5.6)
Tana River	69.85<br>62.05 to 78.03	85.94<br>74.24 to 98.87	60.99<br>49.67 to 73.86	1.3<br>(0.0 to 2.5)	-3.5<br>(-5.3 to -1.5)	399.17<br>(211.73 to 648.86)	242.98<br>(135.15 to 398.65)	198.35<br>(105.85 to 331.04)	-3.1<br>(-6.2 to 0.1)	-2<br>(-7 to 3.3)
Tharaka-Nithi	88.23<br>76.28 to 102.37	58.87<br>49.35 to 69.63	37.37<br>30.51 to 45.2	-2.5<br>(-3.9 to -1.1)	-4.6<br>(-6.7 to -2.4)	182.91<br>(113.6 to 279.82)	136.35<br>(81.8 to 220.49)	83.83<br>(36.85 to 162.01)	-1.8<br>(-5 to 1.2)	-4.9<br>(-10.7 to -0.1)
Trans Nzoia	62.59<br>57.17 to 68.59	43.2<br>38.78 to 47.85	31.84<br>25.88 to 38.61	-2.3<br>(-3.2 to -1.4)	-3.1<br>(-5.2 to -1.1)	147.88<br>(86.17 to 238.61)	140.05<br>(84.66 to 248.68)	105.43<br>(52.07 to 205.9)	-0.3<br>(-3.3 to 2.8)	-2.8<br>(-8.2 to 1.4)
Turkana	145.42<br>131.68 to 159.5	86.61<br>74.24 to 99.95	45.23<br>37.26 to 55.06	-3.2<br>(-4.4 to -2.0)	-6.5<br>(-8.6 to -4.6)	211.71<br>(119.38 to 346.66)	190.89<br>(106.06 to 319.5)	125.53<br>(72.45 to 220.89)	-0.6<br>(-4.1 to 2.5)	-4.2<br>(-8.9 to 1.2)
Uasin Gishu	51.93<br>47.43 to 57.05	42.64<br>38.33 to 47.23	37.79<br>30.74 to 45.78	-1.2<br>(-2.1 to -0.3)	-1.2<br>(-3.3 to 0.7)	237.47<br>(144.24 to 370.9)	174.1<br>(108.87 to 285.12)	143.18<br>(72.42 to 262.67)	-1.9<br>(-5 to 1.1)	-2.0<br>(-7.8 to 2.3)
Vihiga	94.35<br>82.03 to 108.13	97.41<br>80 to 116.62	70.63<br>54.51 to 90.77	0.2<br>(-1.3 to 1.7)	-3.3<br>(-6 to -0.4)	408.57<br>(224.36 to 640.95)	379.12<br>(206.2 to 628.02)	405.73<br>(201.06 to 681.96)	-0.5<br>(-4.1 to 2.9)	0.7<br>(-4.6 to 5.9)
Wajir	145.72<br>125.84 to 165.39	92.53<br>69.36 to 119.92	48.53<br>32.85 to 68.61	-2.9<br>(-5 to -0.9)	-6.5<br>(-9.7 to -3.6)	405.12<br>(250.72 to 611.91)	401.35<br>(262.82 to 587.36)	329.62<br>(189.96 to 510.9)	-0.1<br>(-2.1 to 2.3)	-2.0<br>(-5.4 to 1.4)
West Pokot	135.24<br>123.99 to 147.25	44.01<br>39.7 to 48.62	24.65<br>19.99 to 29.95	-7.0<br>(-7.8 to -6.2)	-5.8<br>(-7.9 to -3.8)	107.79<br>(62.6 to 174.27)	68.94<br>(35.74 to 122.6)	37.29<br>(18.87 to 70.88)	-2.8<br>(-6.7 to 0.9)	-6.1<br>(-11.8 to -0.6)

Appendix Table 8: Cause atributable changes in life expectancy from 1990 - 2006, 2006 - 2016, and 1990 - 2016 for Kenya and counties, both sexes															
	Life expectancy (years)			Overall change in life expectancy due to all causes (years)			Change in life expectancy attributable to each level 1 cause (years)								
							1990 to 2006			2006 to 2016			1990 to 2016		
Location	1990	2006	2016	1990 to 2006	2006 to 2016	1990 to 2016	CMNN	NCD	Injuries	CMNN	NCD	Injuries	CMNN	NCD	Injuries
Kenya	61.4 (60.8 to 62.0)	58.5 (57.9 to 59.1)	66.8 (66.1 to 67.6)	-3.0	8.4	5.4	-3.05	0.03	0.07	8.12	0.16	0.09	5.13	0.16	0.13
Baringo	56.3 (53.7 to 59.3)	59.7 (57.5 to 62.0)	68.4 (65.7 to 71.3)	3.5	8.6	12.1	2.54	0.58	0.35	8.28	0.22	0.13	10.47	1.04	0.59
Bomet	68.8 (66.2 to 71.6)	66.0 (63.4 to 68.4)	71.1 (68.4 to 73.5)	-2.8	5.1	2.2	-3.88	0.90	0.12	5.74	-0.54	-0.12	1.99	0.25	-0.01
Bungoma	62.3 (58.8 to 65.9)	63.3 (60.2 to 66.2)	69.5 (66.7 to 72.3)	1.0	6.2	7.2	0.35	0.47	0.18	6.96	-0.66	-0.12	6.54	0.39	0.25
Busia	58.9 (55.6 to 62.9)	56.5 (52.9 to 60.0)	64.5 (61.3 to 68.2)	-2.4	8.0	5.6	-2.84	0.36	0.10	8.48	-0.43	-0.02	5.76	-0.16	0.05
Elgeyo-Marakwet	61.3 (58.7 to 64)	60.6 (58.5 to 63.1)	67.6 (65 to 70.3)	-0.7	7.0	6.3	1.27	-1.55	-0.42	7.24	-0.32	0.05	6.02	0.09	0.14
Embu	67.5 (64.7 to 70.2)	63.8 (60.9 to 67.1)	69.7 (66.3 to 73)	-3.6	5.9	2.2	-3.08	-0.49	-0.08	5.72	0.18	-0.02	2.53	-0.22	-0.08
Garissa	59.6 (56.9 to 62.3)	61.4 (58.7 to 64.1)	66.6 (63.9 to 69.5)	1.7	5.2	7.0	2.05	-0.28	-0.02	4.59	0.27	0.36	6.77	-0.12	0.31
Homa Bay	49.9 (47.1 to 52.8)	43.0 (40.7 to 45.6)	57.0 (54.4 to 60.5)	-6.9	14.0	7.2	-6.41	-0.32	-0.12	13.38	0.35	0.28	6.99	0.03	0.14
Isiolo	55.0 (52.3 to 57.7)	58.5 (55.5 to 61.9)	65.6 (62.4 to 69)	3.5	7.1	10.6	3.54	0.04	-0.07	7.01	-0.33	0.38	10.61	-0.27	0.24
Kajiado	63.9 (60.9 to 67.2)	59.9 (57.4 to 62.3)	67.9 (65.3 to 70.8)	-4.0	8.0	4.0	-4.14	0.16	-0.01	8.03	-0.05	0.04	3.90	0.10	0.04
Kakamega	59.2 (56.3 to 62.4)	56.8 (54.1 to 59.7)	65.3 (62.7 to 68.2)	-2.4	8.4	6.0	-2.62	0.05	0.14	8.72	-0.33	0.04	6.16	-0.29	0.13
Kericho	66.7 (63.3 to 70.4)	63.3 (59.6 to 66.2)	70.8 (67.4 to 74.7)	-3.4	7.5	4.1	-3.89	0.47	0.03	7.47	0.05	0.01	3.64	0.46	0.04
Kiambu	64.8 (62.3 to 67.6)	60.3 (58.3 to 62.2)	68 (65.8 to 71.1)	-4.5	7.7	3.2	-3.55	-0.75	-0.21	6.34	1.11	0.28	2.80	0.34	0.07
Kilifi	59.0 (55.4 to 62.7)	58.5 (55.9 to 61.6)	67.6 (65.4 to 70.2)	-0.4	9.1	8.6	-0.47	0.13	-0.07	9.09	0.00	-0.02	8.80	-0.24	0.08
Kirinyaga	66.1 (63.0 to 69.4)	61.5 (57.9 to 65.3)	68.2 (64.8 to 72.2)	-4.6	6.7	2.1	-3.54	-0.88	-0.16	5.91	0.63	0.13	2.30	-0.18	-0.01
Kisii	60.7 (56.8 to 64.4)	53.9 (50.9 to 57.1)	67.0 (64 to 70.5)	-6.7	13.1	6.4	-7.45	0.29	0.45	13.01	0.00	0.06	5.76	0.20	0.41
Kisumu	55.5 (52 to 59.2)	47.4 (44.9 to 50.3)	60.5 (57 to 64)	-8.1	13.1	5.0	-7.41	-0.60	-0.08	12.68	0.25	0.14	5.26	-0.32	0.05
Kitui	62.3 (60.3 to 64.7)	62.6 (60.3 to 64.9)	69.0 (66.7 to 71.7)	0.3	6.4	6.7	0.02	0.25	0.05	6.52	-0.13	-0.01	5.67	0.86	0.16
Kwale	58.2 (54.7 to 62.3)	57.9 (55.5 to 60.6)	66.0 (63.3 to 69.0)	-0.2	8.1	7.9	-0.12	-0.07	-0.03	8.38	-0.25	-0.02	7.78	0.01	0.08
Laikipia	67.2 (63.9 to 71.3)	62.8 (59.4 to 66.4)	71.8 (68.2 to 75.4)	-4.4	9.0	4.6	-4.51	0.14	-0.01	8.55	0.27	0.14	4.06	0.40	0.12
Lamu	58.7 (54.5 to 63.6)	57.9 (54.3 to 61.3)	65.5 (62.1 to 69.0)	-0.8	7.7	6.9	-1.74	1.27	-0.35	8.13	-0.36	-0.09	7.53	-0.66	-0.01
Machakos	66.5 (64.2 to 68.9)	63.8 (61.4 to 66.2)	69.5 (67.1 to 72)	-2.7	5.7	3.0	-2.44	-0.26	-0.02	6.04	-0.27	-0.10	3.46	-0.41	-0.10
Makueni	62.5 (59.9 to 64.9)	61.9 (59.6 to 64.4)	68.6 (65.9 to 71.8)	-0.5	6.7	6.2	0.35	-0.63	-0.26	6.42	0.21	0.05	5.37	0.58	0.20

Mandera	58.3 (55.8 to 60.9)	60.3 (57.9 to 62.8)	64.3 (61.4 to 67.2)	2.0	3.9	6.0	2.16	-0.14	0.02	3.33	0.39	0.23	5.57	0.19	0.23
Marsabit	61.4 (58.4 to 64.3)	60.0 (56.9 to 63.2)	65.9 (62.9 to 69)	-1.4	5.9	4.5	-0.59	-0.20	-0.66	5.42	0.07	0.44	4.42	-0.04	0.10
Meru	65.6 (63.4 to 67.9)	62.5 (59.9 to 65.1)	66.6 (64.2 to 69.1)	-3.1	4.2	1.1	-3.24	0.19	-0.01	4.52	-0.24	-0.13	1.23	-0.05	-0.10
Migori	49.4 (46.1 to 52.8)	45.7 (42.9 to 48.7)	59.2 (56.0 to 63.0)	-3.8	13.6	9.8	-3.97	-0.24	0.44	13.55	-0.07	0.08	9.80	-0.30	0.29
Mombasa	62.8 (58.9 to 67.1)	60.2 (57.2 to 63.8)	68.7 (65.7 to 72)	-2.6	8.5	5.9	-2.39	-0.20	0.03	8.04	0.39	0.05	5.67	0.19	0.05
Murang'a	68.7 (65.8 to 72)	63.4 (61.3 to 65.7)	70.3 (67.8 to 72.9)	-5.2	6.8	1.6	-4.61	-0.49	-0.14	5.97	0.69	0.18	1.39	0.17	0.05
Nairobi	64.1 (60.1 to 68.3)	59.8 (57.7 to 62.1)	69.3 (66.8 to 72.1)	-4.3	9.6	5.2	-3.55	-0.77	-0.02	8.68	0.63	0.24	5.16	-0.14	0.20
Nakuru	64.6 (62 to 67.5)	61.6 (59.2 to 63.9)	70.2 (67.6 to 73.2)	-3.0	8.6	5.6	-4.46	1.29	0.19	7.59	0.88	0.12	3.30	2.03	0.28
Nandi	63.9 (61.1 to 67)	61.6 (59.3 to 64)	69 (66.3 to 71.8)	-2.3	7.4	5.0	-2.47	0.17	-0.03	6.88	0.34	0.14	4.50	0.44	0.11
Narok	59.6 (57.2 to 62)	58.9 (56.6 to 61.4)	66.2 (63.7 to 69)	-0.8	7.4	6.6	2.53	-2.36	-0.94	7.14	0.21	0.03	5.82	0.61	0.19
Nyamira	64.2 (60.5 to 68.5)	51.6 (48.8 to 54.4)	62.7 (59.6 to 66)	-12.6	11.1	-1.5	-11.17	-1.18	-0.28	11.23	-0.16	0.05	1.19	-2.32	-0.38
Nyandarua	64.7 (62.1 to 67.6)	58.6 (56.6 to 60.8)	63.3 (60.8 to 66.2)	-6.0	4.7	-1.4	-5.29	-0.59	-0.17	4.59	0.13	-0.03	-0.53	-0.58	-0.24
Nyeri	64 (61.1 to 67)	60.5 (58.3 to 62.6)	66.5 (64 to 69.2)	-3.5	5.9	2.5	-4.01	0.44	0.11	5.71	0.23	0.00	1.83	0.56	0.09
Samburu	62.2 (58.9 to 66)	60.2 (57.2 to 64.1)	66.2 (63.3 to 69.4)	-2.0	6.0	4.0	-2.40	0.19	0.19	6.50	-0.47	-0.05	4.23	-0.32	0.05
Siaya	52.5 (49 to 56.1)	46.8 (43.9 to 49.9)	64.7 (61.9 to 68.2)	-5.7	17.9	12.2	-6.05	0.11	0.20	16.66	0.92	0.31	10.72	0.98	0.46
Taita Taveta	60.5 (56.7 to 64.1)	57.7 (55.2 to 60.5)	64.5 (62 to 67.4)	-2.8	6.8	4.1	-2.27	-0.41	-0.07	7.51	-0.58	-0.10	5.04	-0.82	-0.14
Tana River	57.5 (54.1 to 61.5)	53.5 (50.9 to 56.4)	60.3 (57.4 to 63.1)	-4.1	6.8	2.8	-4.40	0.41	-0.08	6.98	-0.18	0.03	2.60	0.20	-0.04
Tharaka Nithi	59.9 (57.4 to 62.7)	60.3 (57.9 to 62.8)	66.8 (64.3 to 70.0)	0.4	6.5	6.9	0.05	0.32	0.08	6.40	0.08	0.00	5.68	1.03	0.22
Trans Nzoia	66.8 (63.2 to 70.9)	63.8 (60.5 to 67.1)	70.4 (66.6 to 73.6)	-2.9	6.5	3.6	-3.11	0.15	0.06	6.86	-0.27	-0.07	3.76	-0.13	-0.02
Turkana	56.9 (54.1 to 59.7)	56.3 (53.9 to 58.8)	64.1 (61.5 to 66.7)	-0.6	7.8	7.3	-0.21	-0.21	-0.15	7.65	0.02	0.18	6.84	0.16	0.27
Uasin Gishu	66.7 (63.8 to 69.6)	64.6 (62.2 to 66.8)	71.3 (68.6 to 74)	-2.1	6.7	4.6	-3.37	1.10	0.19	6.83	-0.11	-0.02	3.88	0.64	0.10
Vihiga	59.7 (56.6 to 63.6)	56.4 (53.1 to 59.6)	63.2 (60.2 to 66.8)	-3.2	6.7	3.5	-4.28	0.82	0.24	7.23	-0.49	0.00	3.09	0.23	0.19
Wajir	57.9 (55.2 to 60.7)	58.7 (56.1 to 61.5)	64.1 (61.2 to 67)	0.8	5.4	6.2	1.15	-0.30	0.00	4.80	0.32	0.26	6.15	-0.17	0.24
West Pokot	57.6 (55 to 60.7)	60.7 (58.1 to 63.6)	67.9 (65.5 to 71)	3.1	7.2	10.3	2.40	0.46	0.24	7.20	0.01	0.00	9.30	0.68	0.33

Appendix Table 9:
Expected and observed HALE (healthy life expectancy) for Kenya counties for both sexes, 1990 and 2016

Location	1990		2016	
	Expected (On the basis of SDI)	Observed	Expected (On the basis of SDI)	Observed
Kenya	53.0	54.1 (51.8 to 56.1)	59.6	58.6 (56 to 60.7)
Baringo	50.6	49.5 (46.6 to 52.6)	57.9	59.9 (56.5 to 63.3)
Bomet	50.1	60.7 (57.5 to 63.8)	59.6	62.4 (59.2 to 65.4)
Bungoma	52.1	54.9 (51.5 to 58.5)	59.2	60.9 (57.7 to 64.1)
Busia	52.7	51.5 (48 to 55.3)	58.2	56.4 (53.1 to 60)
Elgeyo-Marakwet	50.6	54.3 (51.4 to 57.2)	59.3	59.6 (56.6 to 62.9)
Embu	53.8	59.4 (56.3 to 62.5)	60.7	61 (57.3 to 64.4)
Garissa	35.9	52.3 (49.4 to 55.4)	47.2	58.4 (55.1 to 61.6)
Homa Bay	51.0	43.8 (40.8 to 46.6)	58.8	49.9 (46.7 to 53.3)
Isiolo	50.1	48.3 (45.7 to 51.1)	54.6	57.6 (54.2 to 61.3)
Kajiado	52.5	56.1 (52.9 to 59.4)	60.2	59.4 (56.2 to 62.6)
Kakamega	52.7	52.1 (48.9 to 55.5)	58.8	57.2 (53.8 to 60.4)
Kericho	48.8	58.7 (55 to 62.4)	60.1	62 (58.4 to 65.6)
Kiambu	57.0	57.6 (54.7 to 60.6)	61.9	60.1 (57.1 to 63.2)
Kilifi	43.4	51.7 (48.2 to 54.9)	55.7	59.1 (56 to 62.3)
Kirinyaga	55.1	58.4 (55.3 to 61.9)	61.1	60 (56.5 to 63.8)
Kisii	52.3	53.5 (49.7 to 57.1)	60.7	58.7 (55.2 to 62)
Kisumu	53.2	48.5 (44.9 to 51.9)	60.2	52.6 (49 to 56)
Kitui	49.5	55.1 (52.4 to 57.9)	57.7	60.5 (57.4 to 63.5)
Kwale	47.7	50.9 (47.2 to 54.6)	55.9	57.5 (54.3 to 60.8)
Laikipia	53.2	59.2 (55.5 to 63)	61.4	62.9 (58.9 to 66.5)
Lamu	49.7	51.7 (47.9 to 56.2)	57.3	57.5 (54 to 61.2)
Machakos	52.7	58.8 (55.9 to 61.5)	60.2	61.1 (58.1 to 64.3)
Makueni	51.5	55.2 (52.4 to 57.8)	58.4	60.4 (57.2 to 63.9)
Mandera	35.4	51.4 (48.6 to 54.3)	38.7	56.5 (53 to 59.7)
Marsabit	48.3	54.3 (51.3 to 57.1)	53.2	58 (54.6 to 61.6)
Meru	52.3	57.8 (54.9 to 60.6)	59.3	58.5 (55.5 to 61.4)

Migori	51.2	43.3<br>(40.2 to 46.5)	58.8	51.6<br>(48.2 to 55.2)
Mombasa	54.0	55.1<br>(51.2 to 58.9)	61.1	59.9<br>(56.5 to 63.2)
Murang'a	55.3	60.8<br>(57.5 to 63.9)	60.7	61.8<br>(58.5 to 64.9)
Nairobi	59.8	56.5<br>(52.4 to 60.1)	64.3	60.8<br>(57.6 to 63.9)
Nakuru	52.3	57.1<br>(54.3 to 60)	61.0	61.5<br>(58.1 to 64.8)
Nandi	50.1	56.1<br>(52.9 to 59.2)	59.6	60.3<br>(57 to 63.3)
Narok	48.3	52.6<br>(49.8 to 55.3)	57.0	58.2<br>(55.2 to 61.1)
Nyamira	55.0	56.9<br>(53.2 to 60.6)	61.8	55.4<br>(52.3 to 58.5)
Nyandarua	55.0	57.4<br>(54.4 to 60.6)	61.0	56<br>(53.1 to 58.9)
Nyeri	56.3	57<br>(53.9 to 60.2)	61.5	58.9<br>(56 to 61.9)
Samburu	47.5	54.9<br>(51.3 to 58.5)	52.1	58.3<br>(55.1 to 61.6)
Siaya	49.9	46<br>(42.3 to 49.4)	59.2	56.1<br>(52.8 to 59.7)
Taita Taveta	50.4	53.5<br>(50 to 57.1)	59.6	57<br>(53.9 to 60.1)
Tana River	39.9	50.3<br>(46.8 to 53.8)	50.1	52.4<br>(48.9 to 55.5)
Tharaka Nithi	52.1	53.2<br>(50.5 to 56)	60.1	59<br>(55.7 to 62.3)
Trans Nzoia	50.6	58.7<br>(54.9 to 62.3)	59.5	61.7<br>(58.3 to 65.2)
Turkana	48.0	49.9<br>(46.9 to 52.8)	51.0	56.2<br>(53.1 to 59.3)
Uasin Gishu	52.7	58.7<br>(55.4 to 61.8)	61.1	62.4<br>(59.1 to 65.6)
Vihiga	54.0	52.8<br>(49.6 to 56)	59.5	55.7<br>(52.2 to 59.1)
Wajir	35.4	51.2<br>(48.2 to 54.1)	41.5	56.7<br>(53.3 to 60.1)
West Pokot	48.3	50.8<br>(47.8 to 53.8)	54.6	59.6<br>(56.5 to 62.9)

Appendix Table 10: Age-standardised DALY rates (per 100,000) attributable to top ten leading risk factors for Kenya and provinces, both sexes, 1990 and 2016

Locations	Unsafe water, sanitation, and handwashing		Child and maternal malnutrition		Unsafe Sex		Air pollution		High systolic blood pressure		Dietary risks		Tobacco		Occupational risks		High fasting plasma glucose		Alcohol use	
	1990	2016	1990	2016	1990	2016	1990	2016	1990	2016	1990	2016	1990	2016	1990	2016	1990	2016	1990	2016
Kenya	10600 (7410 to 14800)	6250 (4210 to 8840)	8970 (8210 to 9700)	4490 (4160 to 4860)	4530 (3850 to 5370)	5130 (4800 to 5460)	4300 (3460 to 5150)	2570 (2090 to 3020)	2480 (1950 to 3000)	2490 (2090 to 2930)	2050 (1520 to 2670)	2020 (1600 to 2510)	1400 (1080 to 1760)	1140 (928 to 1360)	1310 (1060 to 1570)	1270 (1040 to 1520)	1100 (863 to 1370)	1370 (1120 to 1690)	1090 (786 to 1430)	1320 (993 to 1630)
Baringo	13800 (8980 to 20400)	8030 (4740 to 12100)	15500 (13300 to 17900)	4310 (3370 to 5380)	4380 (1990 to 8300)	4650 (3740 to 5850)	5990 (4470 to 7720)	2210 (1560 to 3020)	2490 (1770 to 3380)	2080 (1430 to 2860)	2030 (1350 to 2880)	1640 (1110 to 2400)	875 (583 to 1250)	395 (264 to 579)	1330 (933 to 1760)	1250 (971 to 1550)	1100 (798 to 1470)	1060 (769 to 1450)	1100 (493 to 2010)	1020 (487 to 1780)
Bomet	9310 (5900 to 13200)	6180 (3650 to 9310)	3090 (2360 to 3830)	3740 (2550 to 5300)	3850 (1650 to 7890)	4150 (3330 to 5250)	2230 (1530 to 3170)	1920 (1340 to 2580)	1740 (1250 to 2300)	1660 (1170 to 2300)	1410 (950 to 2010)	1290 (877 to 1890)	503 (325 to 756)	566 (367 to 825)	1240 (899 to 1610)	1190 (932 to 1480)	787 (592 to 1030)	857 (636 to 1140)	703 (336 to 1300)	869 (411 to 1510)
Bungoma	10100 (6130 to 14800)	6780 (3890 to 10300)	7920 (6370 to 9770)	4640 (3860 to 5460)	5590 (2740 to 10100)	4400 (3320 to 5930)	3720 (2680 to 4900)	2240 (1620 to 2910)	1790 (1210 to 2510)	1710 (1210 to 2360)	1450 (969 to 2140)	1330 (900 to 1950)	964 (638 to 1400)	687 (477 to 980)	1240 (891 to 1650)	1210 (941 to 1530)	785 (566 to 1060)	842 (623 to 1140)	857 (366 to 1620)	847 (413 to 1470)
Busia	10400 (6240 to 15700)	8450 (4860 to 13400)	9950 (7920 to 12400)	6880 (5610 to 8260)	5540 (2900 to 9860)	4700 (3520 to 6160)	4520 (3380 to 5990)	3390 (2460 to 4460)	2090 (1430 to 3030)	2420 (1650 to 3410)	1720 (1120 to 2610)	1960 (1310 to 2810)	1070 (707 to 1570)	886 (601 to 1280)	1290 (949 to 1680)	1290 (1000 to 1640)	962 (690 to 1320)	1220 (860 to 1680)	953 (424 to 1840)	1170 (501 to 2110)
Elgeyo-Marakwet	12200 (7860 to 18300)	7020 (3870 to 11200)	8940 (7440 to 10300)	3140 (2470 to 3930)	4400 (1930 to 8410)	4680 (3690 to 5900)	4450 (3370 to 5680)	2640 (1920 to 3500)	2680 (1880 to 3550)	2810 (1980 to 3780)	2220 (1500 to 3130)	2250 (1550 to 3130)	1180 (799 to 1710)	835 (540 to 1210)	1270 (924 to 1670)	1260 (958 to 1560)	1090 (791 to 1430)	1340 (964 to 1790)	1030 (427 to 1870)	1250 (559 to 2160)
Embu	9380 (5930 to 13800)	5130 (2850 to 8290)	5980 (4950 to 7050)	4260 (3270 to 5460)	1630 (700 to 3880)	3350 (2580 to 4410)	3300 (2410 to 4270)	2480 (1830 to 3260)	2120 (1500 to 2840)	2280 (1620 to 3160)	1720 (1140 to 2510)	1830 (1230 to 2600)	1010 (691 to 1410)	999 (680 to 1460)	1300 (964 to 1640)	1250 (961 to 1550)	908 (670 to 1210)	1230 (885 to 1680)	879 (408 to 1590)	1110 (487 to 1980)
Garissa	11700 (7710 to 17500)	7180 (4290 to 11000)	11300 (8690 to 13800)	4530 (3420 to 5720)	1040 (649 to 1620)	1990 (1590 to 2540)	5580 (4090 to 7470)	3340 (2450 to 4410)	2410 (1620 to 3150)	2760 (2000 to 3590)	2150 (1330 to 3010)	2410 (1660 to 3250)	2750 (1860 to 3860)	2610 (1830 to 3620)	1170 (819 to 1620)	1480 (1150 to 1840)	1070 (763 to 1400)	1440 (1040 to 1870)	827 (333 to 1650)	1170 (458 to 2150)
Homa Bay	15800 (10800 to 23200)	9750 (6020 to 15000)	17700 (14500 to 21200)	6720 (5390 to 8250)	5770 (2880 to 11400)	10700 (8370 to 13200)	8460 (6460 to 10700)	4700 (3430 to 5930)	3390 (2460 to 4430)	3840 (2640 to 5170)	2880 (1980 to 4000)	3240 (2130 to 4530)	1920 (1350 to 2640)	1740 (1110 to 2600)	1550 (1200 to 1980)	1340 (1040 to 1680)	1620 (1150 to 2160)	2220 (1610 to 2970)	1720 (733 to 3160)	2530 (1120 to 4520)
Isiolo	13000 (9020 to 18400)	7080 (4300 to 10900)	22900 (18400 to 28200)	5640 (4570 to 6840)	1800 (709 to 4260)	3770 (2770 to 5200)	8660 (6040 to 11600)	3250 (2400 to 4250)	2230 (1610 to 3020)	2850 (1970 to 3870)	1840 (1240 to 2710)	2360 (1540 to 3340)	2420 (1610 to 3420)	1400 (909 to 2000)	1120 (786 to 1520)	1250 (955 to 1570)	905 (671 to 1180)	1300 (933 to 1790)	849 (367 to 1550)	1350 (553 to 2490)
Kajiado	8240 (5040 to 12900)	4120 (2590 to 6050)	5690 (4750 to 6700)	3250 (2440 to 4280)	5040 (2390 to 9440)	5000 (3790 to 6320)	3400 (2360 to 4600)	1920 (1320 to 2630)	2970 (2090 to 3910)	2940 (2220 to 3760)	2430 (1630 to 3430)	2320 (1690 to 3120)	1390 (925 to 1950)	1250 (856 to 1730)	1360 (988 to 1770)	1300 (1010 to 1630)	1260 (910 to 1670)	1610 (1200 to 2070)	1150 (511 to 2100)	1440 (666 to 2460)
Kakamega	10500 (6680 to 15600)	8140 (4930 to 12300)	11300 (9410 to 13700)	6280 (4920 to 7930)	6150 (3140 to 10600)	4900 (3530 to 6580)	5170 (3910 to 6610)	3190 (2340 to 4180)	2000 (1390 to 2800)	2380 (1710 to 3260)	1630 (1060 to 2390)	1920 (1340 to 2730)	1370 (907 to 1970)	1130 (787 to 1520)	1290 (938 to 1680)	1300 (1020 to 1630)	907 (652 to 1240)	1270 (949 to 1690)	946 (423 to 1760)	1170 (523 to 2030)
Kericho	9050 (5700 to 14000)	4950 (2830 to 8220)	5100 (4240 to 5950)	4030 (3150 to 5020)	4260 (1940 to 8350)	4770 (3660 to 6340)	2790 (1950 to 3760)	1970 (1370 to 2700)	1940 (1320 to 2640)	1720 (1150 to 2450)	1580 (1030 to 2300)	1340 (864 to 2000)	705 (453 to 1050)	548 (326 to 848)	1270 (894 to 1750)	1200 (918 to 1520)	855 (616 to 1150)	965 (670 to 1310)	762 (350 to 1470)	967 (450 to 1660)
Kiambu	8730 (5210 to 13400)	3650 (2000 to 5660)	4510 (3420 to 5730)	2790 (2220 to 3480)	3020 (1350 to 5800)	4790 (3650 to 6440)	3440 (2520 to 4390)	2020 (1460 to 2620)	3200 (2330 to 4120)	2940 (2190 to 3640)	2640 (1840 to 3610)	2370 (1720 to 3100)	1630 (1110 to 2290)	1340 (877 to 1820)	1390 (1080 to 1780)	1280 (995 to 1570)	1510 (1110 to 1990)	1850 (1400 to 2340)	1600 (765 to 2710)	1640 (783 to 2740)
Kilifi	12000 (7970 to 17400)	5990 (4110 to 8500)	13600 (11500 to 15600)	5110 (4410 to 5880)	5800 (2180 to 11800)	4950 (3910 to 6390)	5000 (3590 to 6590)	2560 (1940 to 3300)	2130 (1340 to 2960)	2430 (1770 to 3120)	1790 (1050 to 2700)	1960 (1370 to 2670)	1160 (768 to 1690)	978 (725 to 1290)	1100 (830 to 1450)	1300 (1010 to 1620)	900 (619 to 1220)	1180 (881 to 1530)	711 (318 to 1430)	1110 (515 to 1860)
Kirinyaga	8860 (5460 to 13200)	4780 (2360 to 8110)	5940 (4260 to 8060)	3800 (3000 to 4780)	2640 (1110 to 5190)	4540 (3240 to 6120)	3570 (2580 to 4750)	2510 (1760 to 3350)	2340 (1590 to 3160)	2550 (1710 to 3600)	1930 (1270 to 2770)	2070 (1340 to 3010)	1420 (945 to 2040)	1240 (784 to 1860)	1340 (1020 to 1720)	1240 (956 to 1570)	1030 (734 to 1400)	1470 (1060 to 2050)	1110 (509 to 1990)	1290 (568 to 2320)
Kisii	11100 (7120 to 16400)	6820 (3950 to 10500)	12400 (10500 to 14600)	4460 (3190 to 6100)	6050 (2550 to 12000)	9090 (7340 to 11500)	5320 (4040 to 6950)	2250 (1530 to 3010)	1900 (1290 to 2750)	2020 (1320 to 2850)	1500 (953 to 2260)	1560 (984 to 2330)	1110 (742 to 1600)	560 (352 to 818)	1280 (951 to 1690)	1190 (933 to 1490)	813 (586 to 1120)	998 (734 to 1330)	840 (375 to 1530)	1000 (475 to 1680)
Kisumu	14000 (9520 to 19900)	6060 (3860 to 9180)	14600 (12400 to 17200)	5050 (3760 to 6740)	6280 (2660 to 12900)	10900 (8790 to 13500)	5510 (4120 to 7030)	3380 (2380 to 4610)	2610 (1710 to 3500)	3240 (2260 to 4430)	2240 (1420 to 3210)	2770 (1890 to 3870)	1840 (1150 to 2640)	1770 (1010 to 2830)	1370 (1010 to 1800)	1280 (989 to 1610)	1450 (970 to 1970)	2240 (1580 to 2970)	1500 (626 to 2740)	2320 (962 to 4280)
Kitui	14800 (10400 to 20900)	8110 (5130 to 11900)	8830 (7300 to 10500)	3690 (2860 to 4810)	1800 (851 to 3830)	3290 (2540 to 4410)	3890 (2870 to 5070)	2140 (1520 to 2860)	2720 (1970 to 3580)	2220 (1590 to 2950)	2310 (1580 to 3270)	1860 (1270 to 2590)	1240 (850 to 1750)	1060 (710 to 1480)	1200 (866 to 1580)	1220 (959 to 1520)	1080 (799 to 1410)	1180 (850 to 1580)	930 (405 to 1690)	1060 (449 to 1900)
Kwale	12600 (8110 to 18700)	7120 (4400 to 10700)	12500 (10400 to 14600)	5280 (4170 to 6690)	6530 (2790 to 13000)	5460 (4480 to 6850)	4950 (3590 to 6550)	2580 (1850 to 3400)	2460 (1560 to 3450)	2560 (1850 to 3390)	2050 (1240 to 3040)	2120 (1500 to 2890)	1450 (915 to 2100)	1340 (932 to 1890)	1200 (866 to 1570)	1330 (1040 to 1660)	982 (673 to 1360)	1350 (986 to 1820)	822 (355 to 1580)	1280 (563 to 2310)
Laikipia	7570 (4500 to 12100)	3830 (2040 to 6300)	4860 (4140 to 5540)	2530 (1940 to 3230)	4180 (1740 to 8410)	4550 (3620 to 5700)	3000 (2110 to 4080)	1670 (1150 to 2370)	2090 (1380 to 2900)	1920 (1320 to 2720)	1730 (1090 to 2460)	1510 (991 to 2230)	1760 (1110 to 2560)	1390 (900 to 2060)	1270 (945 to 1690)	1190 (915 to 1500)	942 (650 to 1280)	1130 (802 to 1570)	904 (407 to 1670)	1010 (481 to 1720)
Lamu	11100 (6920 to 16800)	6150 (3880 to 9120)	12600 (10500 to 15000)	6190 (5200 to 7170)	6640 (2560 to 13300)	5180 (4090 to 6680)	5200 (3630 to 7190)	3200 (2370 to 4140)	2090 (1300 to 3080)	2610 (1790 to 3620)	1730 (1010 to 2720)	2160 (1440 to 3070)	1410 (907 to 2100)	1340 (913 to 1910)	1070 (734 to 1500)	1230 (952 to 1570)	894 (619 to 1240)	1380 (969 to 1900)	781 (313 to 1550)	1250 (547 to 2260)
Machakos	10100 (6600 to 14400)	4900 (3100 to 7180)	6480 (5420 to 7640)	3550 (2690 to 4560)	1740 (730 to 4150)	3170 (2450 to 4310)	3050 (2290 to 3880)	2150 (1570 to 2740)	2250 (1590 to 2950)	2540 (1920 to 3180)	1840 (1250 to 2620)	2060 (1500 to 2740)	1130 (780 to 1540)	1170 (843 to 1540)	1270 (969 to 1620)	1290 (1030 to 1580)	932 (691 to 1230)	1350 (1000 to 1760)	866 (400 to 1480)	1230 (589 to 2050)
Makueni	12100 (7610 to 17600)	7450 (4370 to 11400)	8320 (6640 to 10100)	3220 (2110 to 4730)	2040 (893 to 4160)	3860 (3010 to 5070)	3950 (2960 to 4970)	2310 (1610 to 3090)	2580 (1840 to 3460)	2300 (1600 to 3100)	2160 (1480 to 2990)	1900 (1290 to 2630)	1260 (866 to 1730)	886 (596 to 1260)	1350 (1010 to 1730)	1270 (984 to 1580)	1140 (836 to 1510)	1210 (859 to 1640)	1110 (475 to 1980)	1060 (484 to 1950)
Mandera	12800 (8160 to 19400)	11000 (6950 to 16200)	13600 (10800 to 16500)	6740 (3990 to 10600)	1200 (755 to 1790)	2210 (1830 to 2680)	5070 (3620 to 6800)	3480 (2420 to 4850)	2200 (1490 to 2890)	2230 (1530 to 2910)	1940 (1220 to 2730)	1990 (1290 to 2700)	763 (518 to 1080)	660 (439 to 962)	1080 (783 to 1440)	1500 (1080 to 1980)	1040 (745 to 1400)	1210 (869 to 1620)	835 (353 to 1650)	1030 (371 to 2040)
Marsabit	11800 (7310 to 17700)	9750 (5930 to 14400)	10300 (8070 to 12200)	4350 (3280 to 5560)	1820 (780 to 4120)	3450 (2580 to 4650)	4570 (3310 to 6020)	2830 (2020 to 3750)	2360 (1640 to 3230)	2580 (1810 to 3450)	1980 (1310 to 2870)	2180 (1470 to 3020)	796 (525 to 1200)	655 (428 to 969)	1370 (945 to 1810)	1510 (1150 to 1850)	974 (705 to 1310)	1280 (923 to 1730)	888 (387 to 1750)	1230 (508 to 2330)
Meru	9520 (6240 to 14000)	6140 (3430 to 9380)	5720 (4680 to 6810)	5440 (4300 to 6710)	1940 (861 to 4340)	3510 (2680 to 4670)	3370 (2530 to 4360)	3200 (2430 to 4010)	2730 (2020 to 3500)	2760 (1970 to 3610)	2250 (1590 to 3090)	2240 (1610 to 3080)	1430 (1010 to 1960)	1400 (987 to 1890)	1370 (1050 to 1760)	1350 (1060 to 1660)	1060 (805 to 1410)	1360 (992 to 1800)	987 (476 to 1760)	1250 (521 to 2150)
Migori	15600 (10400 to 22500)	9120 (5630 to 14000)	25300 (21000 to 30200)	8040 (6300 to 9750)	5760 (2700 to 11700)	10500 (8610 to 13000)	10300 (7490 to 13600)	4050 (2970 to 5250)	2140 (1480 to 2980)	2980 (1980 to 4070)	1760 (1150 to 2640)	2450 (1580 to 3540)	2160 (1380 to 3070)	1250 (820 to 1890)	1320 (949 to 1750)	1310 (1020 to 1640)	959 (692 to 1300)	1640 (1150 to 2200)	978 (446 to 1840)	1760 (758 to 3110)
Mombasa	7260 (4450 to 11800)	3160 (1830 to 5620)	8620 (7200 to 10200)	4620 (3510 to 5990)	6420 (2570 to 13700)	5110 (4060 to 6440)	3630 (1990 to 5140)	2010 (1390 to 2760)	2590 (1770 to 3610)	2440 (1730 to 3290)	2110 (1340 to 3160)	1920 (1320 to 2720)	1640 (1080 to 2470)	1150 (788 to 1640)	1300 (960 to 1710)	1210 (954 to 1500)	1080 (783 to 1490)	1440 (1050 to 1950)	1020 (448 to 1890)	1320 (616 to 2300)
Murang'a	8050 (4530 to 12200)	4740 (2510 to 7430)	3370 (2600 to 4330)	3230 (2500 to 4080)	2710 (1170 to 5510)	4520 (3400 to 5920)	2670 (1890 to 3560)	2270 (1690 to 2920)	2040 (1360 to 2720)	1990 (1440 to 2720)	1650 (1070 to 2320)	1570 (1090 to 2250)	904 (565 to 1310)	766 (560 to 1050)	1230 (901 to 1620)	1190 (927 to 1470)	893 (648 to 1180)	1060 (785 to 1420)	953 (446 to 1680)	958 (475 to 1660)
Nairobi	4820 (3400 to 6540)	1440 (885 to 2110)	6860 (5750 to 8060)	2310 (1840 to 2880)	7550 (3270 to 14500)	5080 (3880 to 6470)	1990 (1030 to 3240)	1000 (696 to 1380)	2820 (2020 to 3730)	2880 (2200 to 3560)	2220 (1490 to 3150)	2160 (1570 to 2840)	1570 (962 to 2370)	1600 (1040 to 2210)	1260 (916 to 1670)	1150 (893 to 1430)	1350 (958 to 1850)	1810 (1370 to 2320)	1580 (741 to 2660)	1920 (974 to 3010)
Nakuru	8670 (5430 to 13000)	4430 (2780 to 6550)	4600 (3810 to 5400)	5650 (4360 to 7140)	4290 (1690 to 8600)	4430 (3490 to 5660)	3460 (2550 to 4480)	2370 (1720 to 3120)	2720 (1970 to 3480)	1650 (1160 to 2220)	2290 (1590 to 3060)	1270 (849 to 1770)	2030 (1370 to 2920)	1130 (782 to 1550)	1490 (1140 to 1870)	1170 (908 to 1470)	1250 (932 to 1670)	897 (646 to 1190)	1280 (583 to 2330)	924 (434 to 1590)
Nandi	9910 (6010 to 15200)	6320 (3710 to 9690)	5800 (4680 to 6840)	3390 (2690 to 4240)	4430 (1870 to 8480)	4540 (3710 to 5670)	3340 (2420 to 4350)	2140 (1520 to 2860)	2270 (1620 to 2990)	2110 (1520 to 2780)	1910 (1280 to 2640)	1720 (1200 to 2360)	1380 (873 to 2020)	1070 (694 to 1600)	1500 (1110 to 1980)	1230 (966 to 1540)	1160 (831 to 1580)	1310 (969 to 1730)	1080 (472 to 2080)	1250 (593 to 2230)
Narok	13100 (8220 to 19700)	8630 (5060 to 13000)	8390 (6750 to 10100)	3410 (2530 to 4320)	4320 (2030 to 8290)	4860 (4010 to 5950)	4320 (3180 to 5630)	2660 (1930 to 3490)	3110 (2220 to 4170)	2730 (2010 to 3540)	2590 (1740 to 3680)	2210 (1570 to 2950)	1710 (1180 to 2380)	1320 (865 to 1870)	1520 (1080 to 2030)	1340 (1070 to 1650)	1300 (942 to 1740)	1360 (1010 to 1780)	1170 (553 to 2210)	1310 (582 to 2320)
Nyamira	9920 (5750 to 15500)	7450 (4440 to 11500)	5950 (4600 to 7340)	3810 (2850 to 4910)	5980 (2550 to 12400)	10000 (8170 to 12400)	3110 (2120 to 4130)	3080 (2190 to 3980)	2190 (1430 to 3020)	3390 (2350 to 4440)	1780 (1110 to 2570)	2750 (1830 to 3710)	1220 (754 to 1810)	1450 (897 to 2200)	1280 (950 to 1660)	1330 (1040 to 1670)	948 (645 to 1260)	1860 (1320 to 2450)	1090 (483 to 1970)	1930 (952 to 3340)
Nyandarua	9870 (5710 to 15500)	7570 (4210 to 11700)	4470 (3230 to 6020)	5190 (4030 to 6520)	3010 (1440 to 5530)	5160 (3870 to 6690)	3400 (2430 to 4540)	3570 (2710 to 4610)	2970 (2090 to 3920)	3390 (2390 to 4510)	2480 (1730 to 3430)	2820 (1920 to 3850)	1350 (876 to 2050)	1740 (1140 to 2510)	1440 (1110 to 1830)	1330 (1050 to 1640)	1380 (985 to 1880)	2050 (1490 to 2720)	1480 (718 to 2580)	1950 (850 to 3360)
Nyeri	8850 (5260 to 13900)	4230 (2110 to 6640)	4030 (3020 to 5190)	3510 (2690 to 4440)	3050 (1430 to 5760)	4810 (3710 to 6240)	4000 (3010 to 5140)	3150 (2400 to 4010)	3800 (2740 to 4940)	3460 (2630 to 4400)	3140 (2170 to 4380)	2740 (1980 to 3680)	1600 (1080 to 2230)	1200 (870 to 1650)	1380 (1060 to 1700)	1290 (1010 to 1590)	1510 (1110 to 2080)	1630 (1210 to 2110)	1500 (707 to 2620)	1430 (688 to 2430)
Samburu	11700 (6890 to 17800)	9160 (5320 to 13700)	10300 (8480 to 12000)	3430 (2460 to 4660)	4660 (1710 to 8950)	4770 (3730 to 6050)	4760 (3460 to 6370)	2710 (1860 to 3700)	2100 (1320 to 3000)	2490 (1760 to 3390)	1730 (1030 to 2630)	2080 (1420 to 2940)	1350 (817 to 2070)	1280 (807 to 1840)	1180 (842 to 1600)	1410 (1090 to 1770)	881 (614 to 1220)	1200 (867 to 1660)	835 (348 to 1700)	1240 (503 to 2450)
Siaya	12600 (6570 to 20600)	6620 (3020 to 11600)	16800 (13500 to 20400)	6450 (4680 to 8640)	5560 (2440 to 11500)	8700 (7070 to 10800)	7820 (5610 to 10500)	2830 (1920 to 3910)	1900 (1210 to 2810)	1560 (1020 to 2200)	1660 (987 to 2620)	1300 (810 to 1900)	1940 (1220 to 2940)	1030 (646 to 1570)	1160 (818 to 1590)	1140 (857 to 1460)	1080 (741 to 1540)	1090 (762 to 1460)	947 (398 to 1930)	982 (423 to 1870)
Taita Taveta	9980 (6570 to 15100)	4900 (3010 to 7200)	8480 (7240 to 9810)	3380 (2620 to 4310)	6220 (2470 to 12600)	5340 (4210 to 6850)	4480 (3330 to 5900)	3290 (2400 to 4230)	3120 (2160 to 4230)	3950 (3000 to 4960)	2650 (1730 to 3820)	3290 (2410 to 4330)	1630 (1080 to 2380)	1660 (1190 to 2230)	1450 (1060 to 1880)	1500 (1190 to 1850)	1250 (896 to 1690)	2080 (1590 to 2630)	1150 (476 to 2190)	1680 (716 to 2920)
Tana River	14500 (9300 to 22300)	10900 (6830 to 16700)	8110 (6690 to 9470)	7090 (5680 to 8720)	6640 (2870 to 14000)	6010 (4650 to 7620)	4490 (2980 to 6340)	4010 (2930 to 5270)	3520 (2220 to 4950)	3420 (2450 to 4480)	3130 (1870 to 4550)	3000 (2120 to 4040)	1540 (860 to 2420)	1470 (965 to 2050)	1350 (882 to 1900)	1430 (1140 to 1790)	1490 (986 to 2080)	1860 (1320 to 2490)	1240 (469 to 2460)	1670 (663 to 3200)
Tharaka Nithi	12500 (8330 to 18200)	6930 (3930 to 10600)	8660 (7110 to 10400)	3780 (3210 to 4430)	2160 (903 to 4540)	3930 (3000 to 5090)	5240 (3930 to 6660)	2930 (2150 to 3770)	3630 (2650 to 4730)	2920 (2100 to 3870)	3140 (2170 to 4320)	2460 (1740 to 3290)	2120 (1480 to 2940)	1510 (1000 to 2160)	1590 (1260 to 2010)	1320 (1030 to 1640)	1570 (1120 to 2130)	1680 (1250 to 2230)	1450 (617 to 2640)	1460 (625 to 2560)
Trans Nzoia	8830 (5160 to 14000)	6210 (3600 to 9810)	5690 (4540 to 6810)	3020 (2250 to 3940)	4370 (1780 to 8630)	4660 (3710 to 5900)	2950 (2100 to 4020)	1980 (1380 to 2730)	1610 (1070 to 2350)	1740 (1190 to 2510)	1300 (802 to 2000)	1360 (912 to 2040)	929 (585 to 1440)	703 (440 to 1090)	1210 (861 to 1640)	1210 (926 to 1520)	760 (535 to 1040)	946 (676 to 1330)	692 (316 to 1390)	967 (441 to 1780)
Turkana	15600 (10300 to 22700)	10900 (6580 to 16100)	17500 (14600 to 20200)	5860 (4550 to 7320)	4470 (2050 to 8360)	4740 (3910 to 5870)	6470 (4780 to 8440)	3090 (2260 to 4120)	2230 (1580 to 3000)	2380 (1730 to 3190)	1870 (1240 to 2670)	2010 (1430 to 2800)	1190 (797 to 1750)	730 (489 to 1070)	1330 (950 to 1780)	1430 (1080 to 1770)	1010 (745 to 1330)	1180 (852 to 1600)	985 (413 to 1880)	1290 (529 to 2460)
Uasin Gishu	8630 (5280 to 13200)	4820 (2840 to 7320)	5070 (4240 to 5910)	3850 (3020 to 4850)	4350 (1980 to 8330)	4440 (3560 to 5560)	2990 (2200 to 3900)	1920 (1390 to 2510)	2080 (1470 to 2740)	1710 (1210 to 2330)	1650 (1140 to 2350)	1290 (874 to 1870)	1120 (752 to 1570)	889 (638 to 1230)	1170 (855 to 1560)	1190 (912 to 1490)	887 (654 to 1170)	874 (662 to 1150)	783 (370 to 1460)	870 (428 to 1490)
Vihiga	11000 (6340 to 17300)	9100 (4930 to 14400)	7910 (6010 to 9950)	6830 (4420 to 9970)	5620 (2920 to 10100)	5110 (3700 to 6810)	4410 (3280 to 5680)	3670 (2530 to 5080)	2940 (1910 to 4050)	2850 (1870 to 3960)	2430 (1510 to 3490)	2320 (1480 to 3390)	2000 (1300 to 2910)	1640 (1080 to 2300)	1340 (1040 to 1710)	1320 (1020 to 1660)	1200 (843 to 1620)	1360 (966 to 1870)	1210 (553 to 2200)	1300 (581 to 2300)
Wajir	14400 (9470 to 21000)	11000 (6770 to 16400)	16900 (13500 to 20300)	5510 (3300 to 8410)	1190 (774 to 1770)	2480 (1930 to 3120)	5660 (4080 to 7680)	3280 (2300 to 4560)	2000 (1360 to 2730)	2420 (1660 to 3190)	1740 (1090 to 2560)	2120 (1390 to 2920)	1260 (864 to 1840)	1040 (690 to 1550)	1080 (784 to 1470)	1510 (1110 to 1940)	917 (657 to 1210)	1220 (874 to 1640)	706 (289 to 1390)	1100 (380 to 2190)
West Pokot	15800 (10200 to 22300)	8990 (5380 to 13400)	13400 (11200 to 15800)	3080 (2450 to 3870)	4580 (2030 to 8390)	4750 (3690 to 5960)	5310 (3870 to 6900)	2220 (1510 to 2950)	2460 (1670 to 3400)	2120 (1500 to 2880)	2010 (1270 to 2950)	1720 (1180 to 2390)	1560 (1050 to 2250)	919 (591 to 1320)	1220 (877 to 1650)	1300 (1010 to 1650)	990 (704 to 1310)	1060 (772 to 1420)	914 (379 to 1710)	1040 (492 to 1920)

Appendix Table 11: Level 1 cause attributable DALY rates (per 100,000) for Kenya and 47 counties in 1990 and 2016, age-standardised rate, both sexes						
Locations	1990			2016		
	Communicable, maternal, neonatal, and nutritional diseases	Non-communicable diseases	Injuries	Communicable, maternal, neonatal, and nutritional diseases	Non-communicable diseases	Injuries
Kenya	35100 (32700 - 37800)	17700 (14700 - 20800)	3240 (2650 - 3850)	23100 (21400 - 25100)	17400 (14900 - 20400)	3000 (2560 - 3460)
Baringo	46700 (40800 - 53700)	18100 (14300 - 22100)	3890 (2950 - 4940)	22200 (18800 - 26100)	15700 (12200 - 19400)	2440 (1760 - 3290)
Bomet	21700 (17800 - 27200)	14800 (11700 - 18200)	2260 (1620 - 3130)	18700 (15500 - 22300)	14400 (11500 - 17900)	2390 (1780 - 3150)
Bungoma	36600 (30800 - 43200)	15300 (11900 - 18900)	2790 (1960 - 3820)	22000 (18700 - 26000)	14500 (11500 - 18000)	2300 (1730 - 2980)
Busia	44700 (37900 - 53000)	16500 (13100 - 20400)	3010 (2230 - 4030)	30300 (25400 - 35500)	16900 (13200 - 21000)	2930 (2160 - 3910)
Elgeyo-Marakwet	35500 (30500 - 41900)	18300 (14500 - 22400)	3050 (2250 - 4020)	20500 (17200 - 24900)	18100 (14200 - 22200)	2740 (1990 - 3640)
Embu	24000 (20200 - 28500)	16100 (12700 - 19700)	2690 (2030 - 3480)	18800 (15700 - 22700)	16700 (13100 - 20600)	2980 (2260 - 3880)
Garissa	39400 (34300 - 45600)	18000 (14000 - 21900)	3700 (2770 - 4790)	22700 (18700 - 27100)	18500 (14900 - 22400)	2990 (2260 - 3920)
Homa Bay	62300 (54000 - 72300)	21200 (17200 - 25500)	5870 (4520 - 7370)	41800 (36300 - 47900)	21500 (16800 - 26500)	5540 (4130 - 7410)
Isiolo	55100 (47600 - 63600)	17200 (14000 - 20700)	3730 (2880 - 4700)	25600 (21600 - 30100)	17900 (14000 - 22000)	3050 (2290 - 3930)
Kajiado	28000 (23500 - 33300)	19100 (15100 - 23100)	3150 (2290 - 4120)	19100 (16300 - 21900)	19000 (15400 - 22800)	3130 (2440 - 3990)
Kakamega	42900 (36900 - 49400)	16300 (12600 - 20000)	3270 (2420 - 4300)	27500 (23400 - 31900)	16800 (13600 - 21000)	2940 (2260 - 3770)
Kericho	25800 (21300 - 32000)	15500 (12100 - 19600)	2530 (1780 - 3570)	19000 (16100 - 22700)	14800 (11400 - 18600)	2560 (1830 - 3490)
Kiambu	23800 (19600 - 28900)	19900 (16100 - 24100)	3520 (2600 - 4560)	17500 (15100 - 20400)	19300 (15700 - 23000)	3430 (2620 - 4210)
Kilifi	44500 (38500 - 52800)	16500 (12700 - 20700)	2810 (2120 - 3630)	23000 (20300 - 26100)	16900 (13700 - 20000)	2640 (2100 - 3270)
Kirinyaga	25200 (20500 - 30800)	17000 (13200 - 20900)	3130 (2250 - 4220)	19800 (16600 - 23700)	17500 (13400 - 22100)	3260 (2380 - 4360)
Kisii	39500 (33600 - 47900)	16100 (12600 - 20200)	3360 (2550 - 4350)	26100 (22400 - 30600)	15500 (12300 - 19300)	2330 (1720 - 3080)
Kisumu	49200 (43000 - 57600)	19600 (15100 - 24600)	4120 (2920 - 5400)	34500 (29500 - 40200)	20900 (16500 - 26100)	4080 (2930 - 5290)
Kitui	33100 (28500 - 38100)	17900 (14300 - 21800)	2820 (2140 - 3660)	20300 (17000 - 24100)	16100 (12800 - 19700)	2530 (1880 - 3280)
Kwale	45200 (38400 - 53600)	17200 (13200 - 21400)	2930 (2130 - 3870)	25900 (22200 - 30200)	17400 (14000 - 20900)	2800 (2130 - 3640)
Laikipia	24000 (19500 - 29700)	16300 (12600 - 20300)	2720 (1930 - 3700)	15700 (13100 - 18900)	15600 (12100 - 19700)	2540 (1890 - 3420)
Lamu	44800 (37700 - 54100)	16400 (12500 - 20800)	3110 (2190 - 4230)	25800 (22400 - 29900)	17900 (14000 - 22100)	3150 (2330 - 4140)
Machakos	24900 (21400 - 29100)	16400 (13100 - 20000)	2530 (1910 - 3280)	17500 (15000 - 20200)	17400 (14100 - 20800)	2880 (2280 - 3610)
Makueni	32600 (27800 - 38000)	17700 (14100 - 21400)	3110 (2330 - 3980)	20400 (16400 - 25000)	16500 (12800 - 20100)	2660 (1950 - 3470)
Mandera	43700 (37800 - 49800)	17100 (13400 - 20700)	3380 (2480 - 4350)	29700 (23600 - 37100)	16800 (13300 - 20900)	2880 (2130 - 3820)
Marsabit	35400 (30100 - 41600)	17100 (13500 - 21100)	3110 (2310 - 4100)	24900 (20300 - 30200)	17400 (13500 - 21300)	2940 (2130 - 3850)
Meru	25500 (22000 - 29900)	17800 (14300 - 21400)	2700 (2040 - 3460)	23000 (19500 - 26500)	18100 (14600 - 22000)	3120 (2410 - 3910)
Migori	68800 (59600 - 78300)	17800 (14100 - 21800)	4400 (3250 - 5720)	40400 (34900 - 46100)	19000 (14900 - 23400)	3540 (2570 - 4680)
Mombasa	32600 (26800 - 41000)	17800 (14000 - 22200)	2940 (2160 - 3890)	20200 (17500 - 23700)	17500 (14000 - 21100)	2890 (2230 - 3670)
Murang'a	20300 (16400 - 25100)	15900 (12400 - 19900)	2640 (1810 - 3600)	18400 (15800 - 21400)	15700 (12600 - 19300)	2690 (2100 - 3420)
Nairobi	26800 (21500 - 34400)	19600 (15500 - 24100)	3550 (2650 - 4700)	14900 (12900 - 17000)	19900 (16400 - 23400)	3250 (2540 - 4020)
Nakuru	26200 (21600 - 31700)	18800 (15100 - 22600)	3220 (2310 - 4210)	20600 (17900 - 23500)	15200 (12200 - 18400)	2790 (2190 - 3480)
Nandi	30300 (25700 - 36700)	17100 (13500 - 20900)	3180 (2270 - 4280)	20200 (17100 - 23800)	16200 (13000 - 19900)	2950 (2210 - 3840)
Narok	37900 (32300 - 44300)	19600 (15600 - 23600)	3490 (2610 - 4510)	23600 (19800 - 28200)	18000 (14500 - 21800)	3030 (2240 - 3850)

Nyamira	29500 (23700 - 37200)	16600 (12600 - 20400)	2970 (2020 - 3990)	29000 (24700 - 33900)	20400 (16100 - 24900)	3660 (2640 - 4780)
Nyandarua	24900 (20100 - 30600)	19200 (15300 - 23400)	3390 (2450 - 4480)	26600 (22500 - 31400)	20600 (16300 - 25000)	3990 (3020 - 5130)
Nyeri	24300 (19600 - 30100)	21500 (17100 - 25900)	3450 (2580 - 4390)	20000 (17100 - 23200)	20300 (16400 - 24300)	3320 (2600 - 4100)
Samburu	36800 (31300 - 43700)	16200 (12500 - 20600)	2810 (1980 - 3900)	25000 (20600 - 30500)	17100 (13500 - 21000)	2670 (1930 - 3520)
Siaya	59800 (51500 - 69800)	17500 (13500 - 22600)	3860 (2790 - 5230)	32500 (27300 - 38400)	15100 (11400 - 19500)	2670 (1840 - 3730)
Taita Taveta	35000 (28900 - 43300)	19600 (15200 - 23900)	3330 (2450 - 4440)	22300 (19300 - 25700)	22000 (18000 - 26200)	3830 (3020 - 4790)
Tana River	43800 (35700 - 54200)	21000 (15700 - 26500)	3520 (2250 - 4810)	36300 (31100 - 42400)	20500 (16300 - 25100)	3750 (2840 - 4780)
Tharaka Nithi	34900 (29800 - 40500)	21200 (17100 - 25300)	3860 (3000 - 4810)	21100 (17700 - 25200)	18700 (14800 - 22700)	3420 (2600 - 4330)
TransNzoia	27600 (22800 - 34100)	14600 (11300 - 18500)	2450 (1700 - 3380)	19200 (16000 - 23400)	14800 (11600 - 18600)	2520 (1810 - 3420)
Turkana	50000 (44100 - 57000)	17000 (13800 - 20800)	3480 (2650 - 4560)	30900 (26300 - 36400)	16700 (13300 - 20500)	2710 (1990 - 3560)
Uasin Gishu	25600 (21400 - 31300)	15900 (12500 - 19400)	2480 (1850 - 3290)	18300 (15800 - 21400)	14800 (11800 - 18100)	2320 (1780 - 2930)
Vihiga	38900 (32400 - 46500)	18700 (14500 - 23400)	3220 (2330 - 4250)	31300 (24700 - 38600)	18100 (14200 - 22700)	2720 (2010 - 3460)
Wajir	46300 (40400 - 52300)	16400 (12800 - 20200)	3300 (2440 - 4300)	29700 (23600 - 36400)	17000 (13300 - 21100)	2710 (1990 - 3610)
West Pokot	44900 (39000 - 51700)	17400 (13600 - 21500)	3190 (2370 - 4160)	22600 (18800 - 26800)	15800 (12600 - 19600)	2420 (1740 - 3230)

Appendix Table 12: YLD and YLL age-standardised rates (per 100,000) for LRI (lower respiratory infections), diarrhoeal disease, and malaria in 1990 and 2016 for Kenya and 47 counties, both sexes

Locations	Lower respiratory infections				Diarrheal diseases				Malaria			
	YLDs		YLLs		YLDs		YLLs		YLDs		YLLs	
	1990	2016	1990	2016	1990	2016	1990	2016	1990	2016	1990	2016
Kenya	9.7 (6.5 to 13.8)	8.3 (5.6 to 11.8)	5033.9 (4175 to 6063.4)	2715.8 (2231.2 to 3172.6)	299.3 (206.4 to 408.1)	244.2 (169.4 to 333.1)	10051 (6655.1 to 14489.5)	5968.9 (3796.8 to 8757.4)	137.7 (100.4 to 182)	62 (48 to 78)	2176.3 (1634.6 to 2789.9)	543.9 (372.9 to 743)
Baringo	10.5 (7 to 15)	7.7 (5.2 to 11.1)	7564.5 (5700.2 to 9792.6)	2077.7 (1411.4 to 2821.5)	303.6 (209 to 417.1)	247.9 (171.2 to 337.1)	12914.2 (7998.5 to 19892.6)	7912 (4456.2 to 12277.2)	98.5 (74 to 125.8)	44.1 (34.3 to 55.7)	1672.3 (955 to 2699.6)	191.1 (101.3 to 325.1)
Bomet	8.9 (5.8 to 12.5)	7.8 (5.1 to 11.2)	1923.6 (1218.2 to 3055.8)	1926.9 (1284.4 to 2671)	319.3 (220.9 to 434.6)	258.3 (178.5 to 353)	9099.3 (5504.4 to 13346.5)	5963.2 (3314.7 to 9311.3)	105 (76.9 to 140.2)	38.9 (29 to 50.6)	685.3 (427.1 to 1047.3)	144 (77.8 to 234.7)
Bungoma	9.6 (6.3 to 13.8)	7.9 (5.2 to 11)	4482.7 (3268.2 to 5991.8)	2405 (1705.9 to 3168.1)	300.3 (207.8 to 413.7)	258.8 (180.2 to 356)	9543.9 (5528 to 14419)	6515 (3505.8 to 10095.4)	308.1 (207.4 to 440.2)	82.7 (59.3 to 110)	5466.6 (3993.1 to 6995.7)	891.3 (539.9 to 1335.9)
Busia	9.4 (6.3 to 13.2)	8.2 (5.5 to 11.7)	5485.2 (4117.4 to 7288)	3617 (2627.8 to 4754.5)	313.9 (216.3 to 427.5)	270.1 (185.8 to 371.7)	9722.1 (5286.3 to 15275.6)	8052 (4336.5 to 13244.8)	484.3 (329.7 to 678.7)	187.7 (132.6 to 255.2)	9372.7 (7470.8 to 11540.1)	2735 (1867.7 to 3818.1)
Elgeyo-Marakwet	9.8 (6.5 to 13.9)	8.1 (5.4 to 11.6)	4714.2 (3553.2 to 6221.1)	2106.3 (1542.6 to 2794.4)	340.6 (235 to 464.4)	260.7 (180.1 to 356.5)	11677.5 (7147.8 to 17916.6)	6856.7 (3460.1 to 11236.9)	75.8 (57.4 to 95)	39.1 (29.4 to 50)	772 (465.7 to 1112.5)	107.1 (58.7 to 175.6)
Embu	9.5 (6.2 to 13.5)	8.7 (5.8 to 12.3)	3806.3 (2780.8 to 5026.4)	2879.9 (2102.9 to 3836.1)	315.7 (217.1 to 430.9)	268.2 (186.4 to 370.8)	8929.9 (5397.7 to 13501.3)	4766.3 (2304.3 to 8038.7)	105.3 (77.2 to 140.2)	45.3 (34.3 to 57.3)	1044.8 (637.7 to 1592.6)	271.5 (152.7 to 435.9)
Garissa	11.2 (7.5 to 16.1)	9.2 (6.1 to 13.2)	6464.5 (4699.8 to 9229.2)	2873.7 (1961.8 to 4286.3)	303.9 (209.8 to 415.2)	248.5 (172.6 to 338.9)	10892.6 (6899.6 to 17035.2)	6928.6 (3910.7 to 10962.2)	97.7 (74.8 to 127.2)	44.4 (33.6 to 55.4)	1033.2 (551.3 to 1681.7)	152.3 (81.2 to 248.6)
Homa Bay	10.1 (6.7 to 14.4)	8.2 (5.5 to 11.6)	10692.4 (8215.5 to 13395)	4640.5 (3514 to 5818.2)	305.9 (212.4 to 416.2)	246.2 (171.6 to 335.5)	14490.3 (9328.1 to 21985.3)	9269.8 (5376.3 to 14735.6)	220.9 (155.1 to 304.7)	112.4 (83 to 150.1)	4576 (2843.7 to 6696.1)	1915.2 (1180.8 to 2795.2)
Isiolo	10.4 (6.9 to 15)	8.4 (5.6 to 11.9)	13042.7 (9782.1 to 17064.5)	3155.5 (2316.8 to 4135.9)	289.6 (200 to 395)	242.3 (168.4 to 332.5)	11286.1 (7464.3 to 16587.6)	6826.6 (3880.1 to 10839.2)	92.1 (69.3 to 119.8)	46.8 (36.2 to 58.7)	1919.6 (970.3 to 3506.6)	263.4 (155 to 409.7)
Kajiado	10.1 (6.7 to 14.3)	8.8 (5.8 to 12.5)	3248.8 (2336.5 to 4525.5)	2260.6 (1677.7 to 2911.5)	290.7 (199.1 to 401.4)	215.9 (149 to 294.8)	7933 (4614.9 to 13047.2)	3836.3 (2241.5 to 5918.8)	90.2 (69 to 116.2)	43.7 (33 to 55.2)	696.4 (412.6 to 1045.8)	239 (144.9 to 364.6)
Kakamega	9.6 (6.5 to 13.8)	8.2 (5.5 to 11.7)	6682.1 (5104.6 to 8589)	3446.3 (2598.9 to 4478.4)	296.3 (205.4 to 405.6)	276.7 (191.7 to 376.6)	9670.8 (5613.8 to 15062.1)	7758 (4372.2 to 12139.3)	228.1 (159.7 to 316.2)	110.6 (80.5 to 149.1)	5049 (3294.2 to 6872.2)	1545.1 (943 to 2206.9)
Kericho	8.9 (5.9 to 12.6)	7.8 (5.1 to 11.2)	2600.9 (1784 to 3748.7)	2033.9 (1384.6 to 2770.8)	287.6 (198.2 to 390.9)	248.5 (172.9 to 338.1)	8764.5 (5367.4 to 13958.9)	4690.6 (2485 to 8205.9)	119.7 (88.6 to 160.8)	49 (37.3 to 62.8)	1094.3 (716.8 to 1570.2)	313.4 (174.5 to 503.1)
Kiambu	9.7 (6.4 to 13.9)	8.9 (5.9 to 12.8)	3202.8 (2440.3 to 4085.1)	2378.6 (1776.9 to 2990.9)	297.5 (203.4 to 406.6)	213.6 (147.5 to 294.6)	8425.3 (4699.2 to 13262.2)	3432.2 (1629 to 5594.6)	65.6 (50.5 to 83.3)	37.8 (28 to 48.6)	388.2 (233 to 606.7)	88.5 (53.8 to 135.1)
Kilifi	9.3 (6.3 to 13.2)	7.8 (5.2 to 11.2)	6424.6 (4922.9 to 8556.9)	2700.4 (2031.6 to 3480.5)	280.9 (195.2 to 383.5)	230.7 (159.7 to 318.4)	11238.7 (7109.1 to 16627.3)	5685.2 (3794.9 to 8314.3)	215.8 (151.7 to 305.6)	72.5 (54.5 to 94.5)	3843.6 (2731.4 to 5148.7)	531.5 (339.6 to 788.6)
Kirinyaga	9.8 (6.4 to 14)	8.8 (5.8 to 12.6)	4069 (2938.5 to 5387.3)	2713.1 (1979.6 to 3567.5)	311.3 (214.3 to 427.4)	234.9 (161.9 to 323.5)	8384 (4838.9 to 13184.7)	4491.2 (1906.3 to 7925.1)	85.4 (64.2 to 111.3)	44.5 (33.2 to 56.5)	1207.4 (747.6 to 1834.6)	345.5 (191.7 to 547.6)
Kisii	10.1 (6.6 to 14.4)	7.2 (4.8 to 10.4)	7042.9 (5468 to 8970.5)	2242.9 (1542.4 to 3048.2)	291.5 (201.5 to 401.4)	235.8 (163.2 to 321.8)	10181.8 (5985.8 to 15562.4)	6581.8 (3618 to 10357.8)	125.5 (92 to 166.6)	56.4 (42.4 to 71.3)	1732.1 (1058.7 to 2552.3)	291.7 (171.3 to 464.9)
Kisumu	10.3 (6.9 to 14.7)	8.3 (5.6 to 12)	6894.5 (5438.5 to 8645.4)	3518.2 (2388 to 4836.3)	301.4 (207.9 to 416.3)	216.4 (150.7 to 294.1)	13219.7 (8578 to 19360.5)	5665.9 (3357.9 to 8942.3)	246.8 (171.6 to 341.7)	124.9 (91.2 to 165)	5379.6 (3748.6 to 7177.8)	1825.4 (1077.8 to 2825.2)
Kitui	10 (6.7 to 14.2)	8 (5.4 to 11.6)	3857.3 (2808.3 to 5207.2)	1909.5 (1322.3 to 2606.1)	380.4 (262.2 to 522.4)	346.2 (240.3 to 472.9)	14454.2 (9604.7 to 20571.5)	7890 (4756.3 to 11910.6)	95.7 (72.6 to 124.7)	42.5 (31.9 to 54)	793.5 (480.7 to 1255.7)	133.6 (77.5 to 221.8)
Kwale	10 (6.7 to 14.2)	8 (5.3 to 11.4)	6002.5 (4508.6 to 7968.7)	2634.9 (1915.6 to 3483.6)	316.8 (220.1 to 432.1)	260.7 (179.9 to 359.1)	11967.5 (7339.3 to 18464.8)	6847.8 (4080.3 to 10502)	180 (128.2 to 249.3)	76.9 (57.8 to 100.2)	2570.6 (1802.9 to 3562.5)	562.3 (355.9 to 838.1)
Laikipia	9.5 (6.3 to 13.6)	8 (5.4 to 11.5)	3098.5 (2249.6 to 4326)	1690.7 (1173.8 to 2372.7)	291 (201.1 to 397.2)	223.8 (154.8 to 307.4)	7187.3 (3912.7 to 12083.1)	3596.4 (1723 to 6168.6)	66.1 (50.6 to 83.4)	37.6 (28.1 to 47.7)	416 (233.9 to 686.9)	90.8 (49.4 to 152.5)
Lamu	10.2 (6.8 to 14.8)	8.8 (5.9 to 12.6)	7237.7 (5168.4 to 10061.7)	3743.6 (2876.8 to 4844.8)	294.6 (203.4 to 401)	229.6 (159.1 to 315.6)	10205.9 (5826.3 to 16325.6)	5736.5 (3380.1 to 9027.6)	144.1 (100.7 to 199.4)	44.4 (33.6 to 56.7)	4269.2 (2481.2 to 6151.9)	507.9 (268.9 to 833.8)
Machakos	9.3 (6.2 to 13.6)	8.4 (5.5 to 12)	3081.8 (2287.8 to 4014.6)	2200.5 (1687.8 to 2793.4)	300.2 (207.4 to 415.3)	231.1 (159.1 to 315.3)	9771.2 (6215.9 to 14350.6)	4619.3 (2735.1 to 7056.8)	95.4 (71.4 to 125)	43.2 (32.3 to 55.6)	879.3 (559.3 to 1323)	199.7 (120.5 to 310)
Makueni	9.7 (6.3 to 13.8)	8.1 (5.4 to 11.7)	4244.9 (3189.9 to 5497.9)	2231.2 (1542.4 to 3024.7)	323.6 (224.2 to 441)	262.4 (181.2 to 359.2)	11602.4 (6807.8 to 17405.9)	7217.9 (3982.9 to 11353.8)	103 (77.7 to 135.6)	43.7 (33.3 to 56.6)	1131.7 (661.9 to 1835.8)	171.1 (93.6 to 286.3)
Mandera	10.3 (6.9 to 14.6)	9.3 (6.2 to 13.2)	5678.7 (3898.4 to 8048.5)	3249.2 (1952 to 5061.4)	308.4 (214.7 to 421.9)	275.1 (189.6 to 374.1)	12226.9 (7428 to 19251.3)	10747.6 (6662.3 to 16315.2)	67.7 (50.7 to 86.2)	43.9 (33.5 to 55.1)	275.9 (109.1 to 544.6)	72.8 (33.7 to 138.2)
Marsabit	10.3 (6.9 to 14.8)	8.7 (5.8 to 12.3)	5286.7 (3809.1 to 7229.5)	2480.9 (1711.6 to 3448.9)	325.2 (226.4 to 443.1)	277.7 (192.1 to 379.4)	11177.8 (6415.8 to 17253.9)	9579.3 (5583.3 to 14375.7)	86.7 (65 to 113.9)	44.8 (33.6 to 57.2)	752.1 (353.1 to 1466)	113.5 (53 to 215.9)
Meru	9.3 (6.2 to 13.1)	8.9 (5.9 to 12.7)	3076.8 (2292.4 to 4112.7)	3472.6 (2649.9 to 4361.7)	285.1 (197.1 to 388.3)	232 (160.5 to 321.4)	9194.6 (5742.3 to 13921)	5804 (2860.6 to 9232.9)	116.7 (85.5 to 156.2)	51.4 (38.6 to 66.4)	1069.2 (679.7 to 1576.2)	481 (257.9 to 805.8)
Migori	10.7 (7.1 to 15.3)	8.4 (5.6 to 12)	15091.3 (11440.5 to 19946.5)	4357.8 (3325.4 to 5545)	306.6 (214.7 to 415.6)	253.6 (176.8 to 349.6)	13586.1 (8190.6 to 20861.7)	8651.7 (5103 to 13696.2)	138.2 (102 to 183)	94.9 (71.6 to 123.9)	4920 (2795.9 to 7736.7)	1945.9 (1185.5 to 2882.7)
Mombasa	10.1 (6.7 to 14.4)	8.7 (5.8 to 12.4)	4886 (3631.3 to 6449)	3002.5 (2239.1 to 3890.9)	257.2 (177.5 to 351.5)	224.7 (156.5 to 307.3)	6644.1 (3790.2 to 11526.8)	2705 (1339.1 to 5387.6)	93.9 (69.9 to 122)	69.1 (51.3 to 91.7)	1344.7 (847.5 to 1982.3)	684.8 (398.4 to 1032.1)
Murang'a	8.7 (5.8 to 12.4)	7.9 (5.3 to 11.5)	2732 (1911.6 to 3750.8)	2647.3 (2034.1 to 3347.6)	314.5 (217.2 to 430.7)	237.6 (162.6 to 328.2)	7690.4 (4018 to 12100.7)	4413.3 (2117.2 to 7181.8)	77.2 (57.6 to 100.5)	41.7 (30.7 to 52.7)	758.1 (443.2 to 1198.3)	290.6 (167.4 to 470.5)
Nairobi	10 (6.7 to 14.4)	9.1 (6.1 to 12.9)	4184.7 (3302.7 to 5235.5)	2015.2 (1545.8 to 2550)	196.5 (135.9 to 268.9)	160.2 (111.5 to 217.5)	4549.1 (3197.1 to 6306.4)	1246.9 (650.9 to 1984)	47.3 (36.4 to 60.4)	34.7 (25.2 to 45)	16.8 (9.9 to 27.7)	2.7 (1.5 to 4.1)
Nakuru	9.8 (6.5 to 13.9)	8.3 (5.5 to 12)	3391.6 (2543 to 4509.8)	3437.6 (2473.4 to 4582.2)	281.3 (195.5 to 382.6)	237 (164.6 to 322.9)	8324.6 (4922.9 to 12998.8)	3961 (2325.1 to 6291.1)	63.9 (49.3 to 80.2)	37.5 (28 to 48.4)	373.6 (206.6 to 594.5)	133.6 (69.4 to 230.2)
Nandi	9.1 (6 to 12.8)	7.8 (5.2 to 11.2)	3136.8 (2232 to 4270.3)	1904.2 (1350.8 to 2558.4)	280 (192.6 to 380.1)	230 (158.7 to 313.7)	9580.5 (5526.7 to 15511)	6158.8 (3385 to 9696.5)	131.1 (96.5 to 173.9)	57.7 (42.9 to 74.6)	1437.4 (949.4 to 2021.7)	405.7 (239.7 to 618.6)
Narok	9.7 (6.4 to 13.9)	8.1 (5.4 to 11.6)	3974.6 (2972.6 to 5356.5)	2227.1 (1612.9 to 2929.2)	330.9 (229.4 to 450)	283.9 (196.4 to 388.9)	12679 (7696.4 to 19419.5)	8453.9 (4776.1 to 13138.1)	74.2 (56.7 to 92.4)	41.8 (32 to 53.4)	558.3 (348.5 to 853.1)	126.2 (72.8 to 202.2)
Nyamira	9.1 (6 to 13)	8.3 (5.5 to 11.8)	3190.1 (2253.4 to 4274.3)	2611.9 (1898.7 to 3401.5)	299.8 (207.9 to 411.6)	249.2 (172.3 to 342.1)	9555.3 (5164.8 to 15377.1)	7227.8 (4083.6 to 11475.6)	111.6 (81.2 to 149.7)	49.5 (37.6 to 63.5)	1402.9 (915.1 to 2020.6)	273 (165 to 422.1)

Nyandarua	9.4 (6.3 to 13.6)	9.1 (6 to 13.1)	2972.8 (2068.6 to 3985.4)	3661.1 (2813.8 to 4642)	316.8 (217.1 to 431.3)	266.8 (182.9 to 362.3)	9568.1 (5204.4 to 15505.8)	7251.1 (3690.7 to 11657.2)	47 (34.8 to 60.2)	34.9 (25.9 to 44.6)	8.8 (4.6 to 15)	2.1 (0 to 4)
Nyeri	10.3 (6.8 to 14.6)	9.1 (6.1 to 12.9)	3455.5 (2614.1 to 4390.3)	3317.4 (2637.8 to 4024)	306.4 (209.7 to 421.9)	222.2 (152.8 to 303.1)	8469.3 (4648.6 to 13696.7)	3924 (1674.5 to 6506.5)	65.4 (49.2 to 81.7)	39.9 (30.1 to 50.7)	506.9 (317.2 to 763.6)	207.6 (118 to 330.5)
Samburu	9.4 (6.3 to 13.5)	8.3 (5.5 to 11.7)	5847.4 (4388.5 to 7770.4)	2337.3 (1553.7 to 3293.5)	313.6 (218 to 423)	270.7 (187.4 to 370.9)	10968.3 (6139.8 to 17601.2)	8991.3 (5075.3 to 14013.2)	104.3 (77 to 137.3)	45.3 (34.7 to 58.2)	646.1 (307.8 to 1274.7)	115.4 (63.8 to 191)
Siaya	10.2 (6.7 to 14.7)	7.2 (4.7 to 10.3)	10824.5 (8236.5 to 14240.7)	3500.3 (2424.4 to 4763.3)	371.1 (257.4 to 504)	364.7 (254.2 to 495)	11069.2 (4716 to 19186.3)	6045.8 (2259.1 to 11158.9)	373.4 (256.9 to 519.7)	209.9 (147.3 to 289.2)	12228.5 (9223.1 to 16000.3)	3953.1 (2645.4 to 5387.8)
Taita Taveta	10.5 (6.9 to 15)	9.6 (6.3 to 13.6)	4663.6 (3561.3 to 6121.2)	3380.3 (2662.7 to 4216.2)	274.9 (189.1 to 376.3)	199.7 (137.1 to 272.6)	9446.8 (5971.5 to 14758.6)	4503.2 (2581.7 to 6945.6)	103.5 (76.6 to 136.1)	47.1 (36 to 59.5)	1033.5 (667.5 to 1499)	346.6 (203.7 to 537)
Tana River	9.9 (6.6 to 14)	9.2 (6.2 to 12.9)	3890.4 (2671.7 to 5679.7)	3831.2 (2751.3 to 5104.9)	312.8 (217.3 to 426.8)	269.1 (185.4 to 370)	14173.5 (8852.2 to 22195.3)	10646.9 (6439.7 to 16850.3)	174.9 (125.2 to 235.8)	49.2 (37.5 to 62.2)	1210.7 (762.6 to 1733.8)	255.6 (144.8 to 407.1)
Tharaka Nithi	10.7 (7 to 15.3)	8.6 (5.7 to 12.4)	5403.9 (4112.7 to 6872.9)	2871.3 (2180.9 to 3640.1)	320.4 (220.8 to 437.7)	251.8 (174.8 to 344.9)	11893 (7454.3 to 17936.4)	6654.7 (3574.2 to 10731.8)	119.9 (86.8 to 160.7)	48.2 (36.8 to 61.8)	1355.1 (820.9 to 2134.4)	289.5 (164.6 to 459.4)
Trans Nzoia	8.9 (6 to 12.7)	7.8 (5.2 to 11)	3297.6 (2333.8 to 4605.8)	1938.7 (1325.3 to 2630.5)	293.4 (203.6 to 398.9)	273.7 (189.7 to 373.8)	8419.4 (4614.8 to 14047.5)	5993.2 (3244.8 to 9949.7)	137.4 (99.7 to 188)	48.3 (36.4 to 61.4)	1739 (1086.4 to 2526)	283.1 (165.1 to 465.1)
Turkana	9.9 (6.6 to 14.1)	8.3 (5.5 to 11.7)	8393.3 (6121.6 to 11313.7)	2795.1 (1958.6 to 3962.6)	323.2 (224.1 to 444.4)	278.6 (193.9 to 380.8)	14679.7 (9117.1 to 22136)	10764.3 (6213.8 to 16400)	101.8 (77.1 to 132.6)	66.6 (50.7 to 87)	649.8 (350.7 to 1072.4)	252.6 (141.8 to 416)
Uasin Gishu	9.1 (6.1 to 12.8)	7.8 (5.3 to 11.2)	3034.2 (2201.2 to 4094.4)	2211.4 (1606.8 to 2911.7)	278.4 (192.7 to 382.5)	217.8 (149.9 to 299.6)	8299.7 (4849.9 to 13153.1)	4563.1 (2468.7 to 7257.8)	86.2 (64.6 to 111.4)	41.6 (31.3 to 52.8)	1031.1 (656.4 to 1502.1)	330.2 (190 to 513)
Vihiga	9.6 (6.4 to 13.7)	8.4 (5.6 to 12.2)	4515.3 (3360 to 5920.4)	3730.5 (2495.4 to 5392)	313.7 (217.3 to 432.1)	268.9 (185.8 to 366.8)	10512.3 (5555.6 to 17225.1)	8729.4 (4381 to 14266.1)	264.4 (181.3 to 372.2)	141.7 (100.1 to 193.6)	4432.7 (3180.7 to 5878.3)	2307.2 (1409.8 to 3606.7)
Wajir	10.2 (6.8 to 14.5)	8.9 (5.8 to 12.9)	6913.9 (4725.8 to 9653.5)	2883.4 (1827.8 to 4614.5)	316.1 (218.9 to 430.6)	276.7 (191.2 to 377.5)	13641.5 (8392.4 to 20424.6)	10779.7 (6335.4 to 16413.2)	67.2 (51.7 to 85.2)	44.6 (34.5 to 56.1)	425.4 (190.9 to 864.9)	90 (44.8 to 166)
West Pokot	10.2 (6.7 to 14.5)	7.6 (5.1 to 10.9)	6302 (4608.9 to 8359)	1753.7 (1138.2 to 2516.5)	358 (245.8 to 487.7)	275.7 (189.6 to 377.8)	15137.2 (9341.5 to 21885.5)	8878.9 (5063.2 to 13400.5)	169.9 (122.7 to 230.9)	67.4 (50.2 to 87.2)	2421.9 (1386.7 to 3745.1)	294.9 (180.8 to 454.2)
